# Triarylpyrylium-based fluorescent DNA-binding dyes – facile synthesis, substituent effects, and sensing mechanism

**DOI:** 10.1039/d5ra04750a

**Published:** 2025-08-22

**Authors:** Farkas Domahidy, Levente Cseri, Gábor Turczel, Blanka Huszár, Balázs J. Rózsa, Zoltán Mucsi, Ervin Kovács

**Affiliations:** a BrainVisionCenter Liliom utca 43-45 H-1094 Budapest Hungary kovacs.ervin@ttk.hu; b Hevesy György PhD School of Chemistry, Eötvös Loránd University Pázmány Péter sétány 1/A H-1117 Budapest Hungary; c NMR Research Laboratory, HUN-REN Research Centre for Natural Sciences Magyar tudósok körútja 2 H-1117 Budapest Hungary; d Laboratory of 3D Functional Network and Dendritic Imaging, HUN-REN Institute of Experimental Medicine Szigony utca 43 H-1083 Budapest Hungary; e Two-Photon Measurement Technology Research Group, Pázmány Péter Catholic University Práter utca 50/a H-1083 Budapest Hungary; f Faculty of Material and Chemical Sciences, University of Miskolc H-3515 Miskolc Hungary; g Institute of Materials and Environmental Chemistry, HUN-REN Research Centre for Natural Sciences Magyar tudósok körútja 2 H-1117 Budapest Hungary

## Abstract

Novel pyrylium-based fluorescent DNA-binding dyes were developed, containing *p*-aminoaryl groups at the *α* positions of the pyrylium moiety. The amines are substituted with aminoalkyl groups to enhance the dye's water solubility and DNA binding affinity. With this structural modification triarylpyryliums exhibit up to 7.6 times higher fluorescence enhancement upon binding to DNA, rendering them promising candidates for fluorogenic DNA sensors. The photochemical characteristics of the dyes, including the radiationless deexcitation pathways necessary for the DNA-induced fluorogenicity were studied using both spectroscopical experiments and theoretical calculations. Particular attention was given to the possibility of twisted intramolecular charge transfer (TICT) states as a quenching pathway in the unbound state. These findings support the rational design of structurally dynamic, environment-sensitive fluorophores for nucleic acid detection and molecular diagnostics.

## Abstract 2

A series of novel fluorescent DNA-binding pyrylium dyes is developed, and the mechanism of their fluorogenicity is explored computationally.

## Graphical abstract

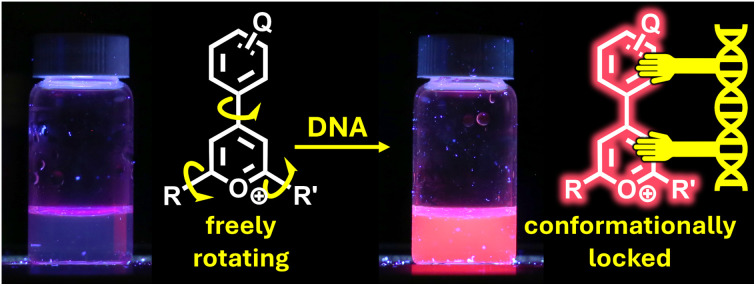

## Introduction

In recent years, pyrylium salts have garnered increasing attention due to their wide range of applications in light-driven processes. They have been utilised as visible-light-induced photocatalysts and polymerisation photoinitiators, typically acting as photooxidants, generating a radical cation type intermediate.^1–3^ Triarylpyryliums and -thiopyryliums have also been tested as prospective photosensitisers for the *ex-vivo* decontamination of blood-borne pathogens in blood. These molecules bind to deoxyribonucleic acid (DNA), which was considered to be advantageous as it could provide selectivity towards the pathogens over red blood cells.^4,5^ Several pyrylium-based dyes have been utilized as fluorescent sensor molecules for the detection of various analytes *in vitro* and *in vivo*. Most of these sensor molecules harness the tendency of pyryliums to react with nucleophiles, including cyanide,^6^ sulfide,^7^ ammonia, and various amines.^8,9^ Due to the different absorption band of the resulting product from that of the original pyrylium, the analyte can be detected colorimetrically. Pyrylium-based fluorophores are also becoming increasingly utilised for intracellular imaging. The apolar-cationic character of styrylpyrylium dyes could be harnessed to stain mitochondria in hepatoblastoma cells.^10^ Far-red to near-infrared fluorescent probes based on a conformationally locked triarylpyrylium scaffold were also developed, including a nitroreductase sensor for the *in vivo* detection of cell hypoxia.^11^ The monitoring of intracellular pH during apoptosis was also achieved using a conformationally locked 2,6-bis(hydroxyaryl)pyrylium fluorophore with pH-responsive fluorescence.^12^

The DNA binding and environment-sensitive fluorescent properties of pyrylium dyes were also harnessed in several applications, such as the detection of double-stranded DNA (dsDNA) in gel electrophoresis using 2,4-bis(4-dimethylaminophenyl)-6-methylpyrylium^13,14^ An NMR study proposed that this compound binds to DNA intercalatively.^15^ Pyrylium dyes were also used in Fluorescence *In Situ* Hybridization (FISH) for the detection of specific DNA sequences by covalently linking the fluorophore to a single-stranded DNA containing the complementary sequence. Upon hybridization the pendant fluorophore can non-covalently bind to the resulting double helix, resulting in increased fluorescence.^16^

Our objective was to design and synthesize pyrylium-based compounds that are well-suited for the detection of minute quantities of DNA and exhibit favourable aqueous solubility. The latter required the incorporation of polar auxiliary groups; considering the structural resemblance of triarylpyryliums to other donor–acceptor–donor type DNA-binding dyes, such as Miami Yellow and Green,^17^*N*′-methylpiperazine-*N*-yl groups were chosen as a starting point in the substituent optimisation.

## Results and discussion

### Synthesis

**Fig. 1:**
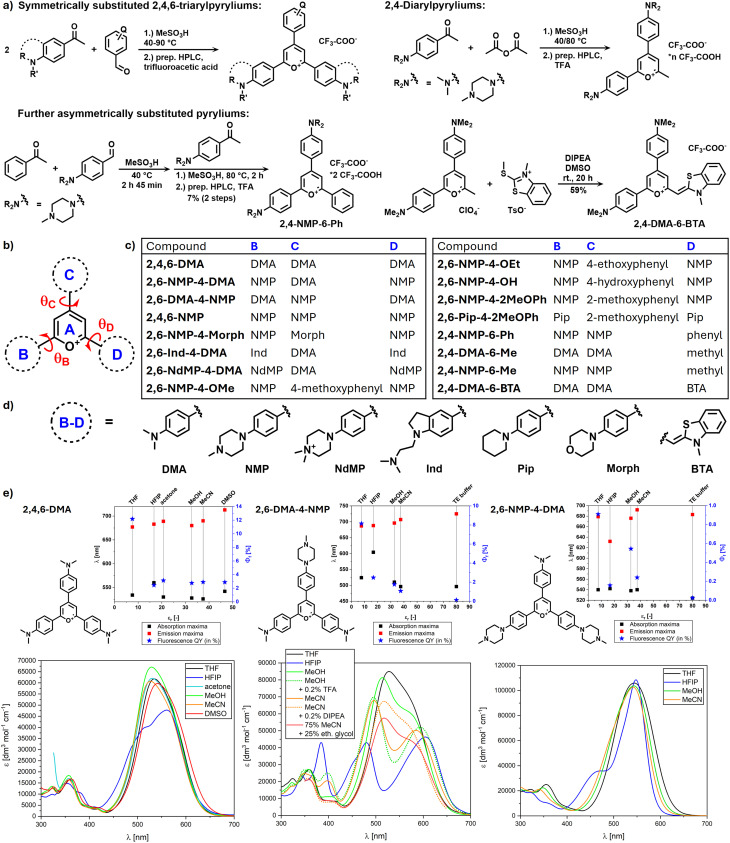
(a) Synthesis of the pyrylium dyes. (b) General formula of 2,4,6-trisubstituted pyryliums with the relevant dihedral angles (red). (c) Substituents of the synthesized pyrylium dyes. (d) Abbreviations of the substituents. (e) Spectroscopic properties and absorption spectra of 2,4,6-DMA (left), 2,6-DMA-4-NMP (middle), and 2,6-NMP-4-DMA (right) in various organic solvents (HFIP: 1,1,1,3,3,3-hexafluoroisopropanol).

The 2,4,6-triarylpyrylium salts were synthesized from the corresponding ketones and aldehydes *via* condensation in methanesulfonic acid (Fig. 1a). During this condensation a dihydropyran ring is formed, which is oxidized to pyrylium *in situ*, presumably by atmospheric oxygen. The 4-dialkylamino-substituted benzaldehyde and acetophenone intermediates were prepared from the S_N_Ar-type reaction of secondary amines and 4-fluorobenzaldehyde or 4′-fluoroacetophenone. An exception is the ketone intermediate of 2,6-Ind-4-DMA, synthesized from indole *via* a three-step route (Fig. S2 in SI). The asymmetrically substituted pyrylium, 2,4-NMP-6-Ph was prepared in a one-pot reaction where the aldehyde and one of the ketones were reacted at 40 °C and the resulting chalcone was reacted with the other ketone at 80 °C. Diarylpyryliums were synthesized in the acid-catalysed condensation of 4-dialkylamino-substituted acetophenones with acetic anhydride, as previously reported.^13^ In one of the asymmetrical derivatives a (3-methylbenzo[*d*]thiazol-2(3*H*)-ylidene)methyl donor group – also present in thiazole orange dyes – was incorporated into the fluorophore, replacing one of the aryl donor groups (2,4-DMA-6-BTA). This molecule was prepared from the diarylpyrylium 2,4-DMA-6-Me by reacting it with 3-methyl-2-methylthiobenzothiazolium *p*-toluenesulfonate. All synthesized pyrylium dyes were obtained as trifluoroacetate salts following purification with preparative HPLC.

### Spectroscopical properties

**Fig. 2:**
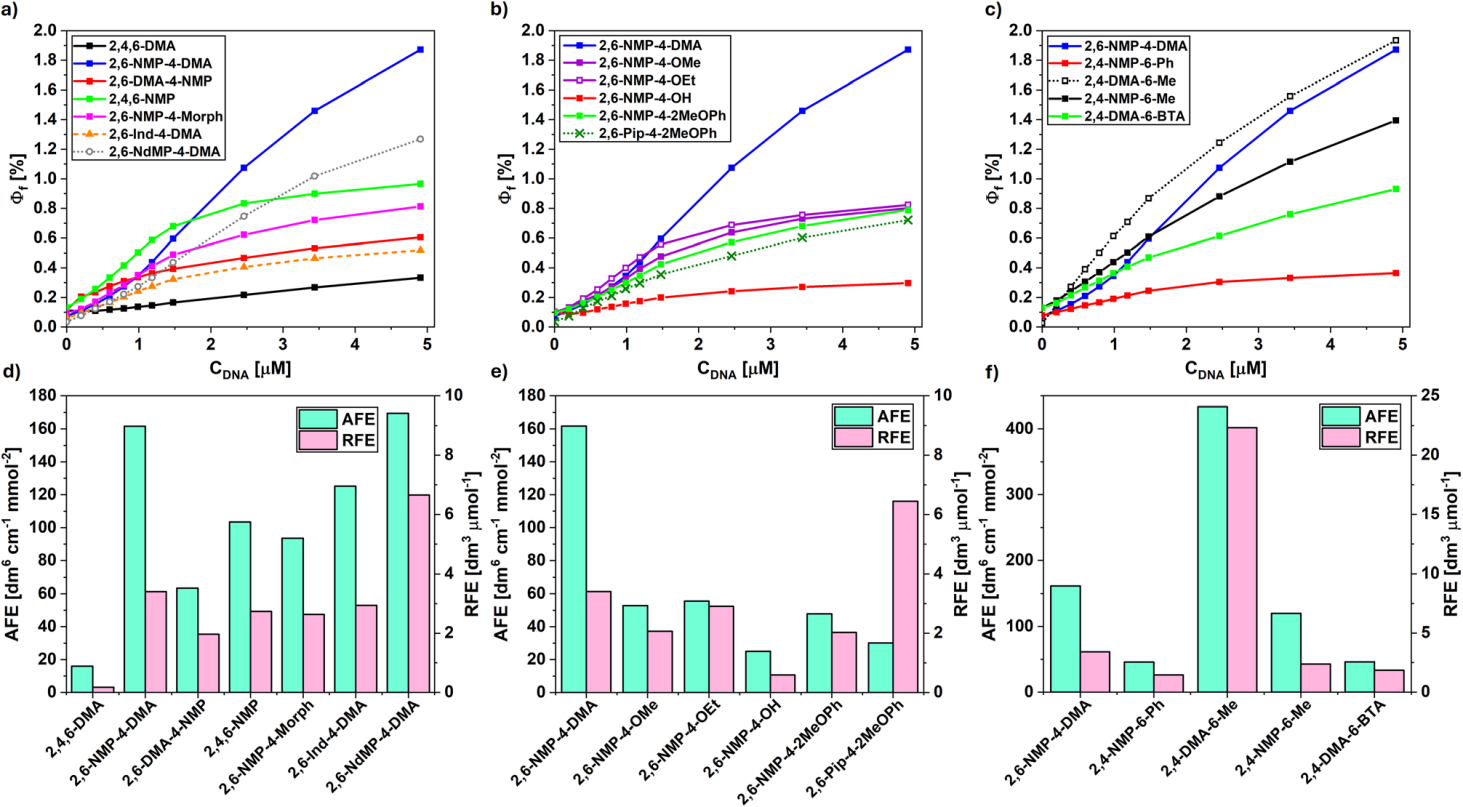
(a–c) Dependence of the effective fluorescence quantum yield (*Φ*_f_, in percents) on the concentration of plasmid DNA. (d–f) Absolute (AFE) and relative fluorescence enhancements (RFE) of the synthesized pyrylium derivatives.

The absorption and emission spectra of the pyrylium dyes were recorded in Tris–EDTA (TE) buffer (pH = 7.4). The dependence of fluorescent emission on the concentration of dsDNA (*C*_DNA_) was measured by titrating a solution of each dye in TE buffer with a stock solution of cloned plasmid DNA (6882 base pairs). Fluorescence quantum yield of the DNA-bound form (*Φ*_f_^DNA^) was measured in the presence of 50 μM plasmid DNA (Table 1 and Fig. 2). Furthermore, the emission of 2,4,6-DMA, 2,6-DMA-4-NMP, 2,6-NMP-4-DMA were recorded in various organic solvents (Fig. 1e).

**Table 1:** Spectroscopical properties of the prepared chemosensors. Extinction coefficients (*ε*^TE^, dm^3^ mol^−1^ cm^−1^) absorption maxima (*λ*_abs_, nm) emission maximum wavelengths in solution and in dsDNA (*λ*^TE^_em_, *λ*^DNA^_em_, nm); fluorescence quantum yields in solution and in dsDNA (*Φ*^0^_f_, *Φ*^DNA^_f_, percent) in pH = 7.4 TE buffer. Absolute fluorescence enhancements (AFE, dm^6^ cm^−1^ mmol^−2^) and relative fluorescence enhancements (RFE, dm^3^ μmol^−1^); the highest AFE/RFE values of di- and triarylpyryliums are underscored. Calculated torsional angles between the A (pyrylium) ring and the B, C, or D rings (*θ*_B_, *θ*_C,_*θ*_D_). (n.c.: not calculated)

Compounds	*ε* ^TE^	*λ*_abs_	*λ* ^TE^_em_/*λ*^DNA^_em_	*Φ* ^0^_f_ [%]	*Φ* ^DNA^_f_ [%]	AFE	RFE	*θ*_B_	*θ*_C_	*θ*_D_
2,4,6-DMA	82 800	534	733/699	0.061	0.29	31.5	0.62	13.3	20.5	13.3
2,6-NMP-4-DMA	64 500	530	702/629	0.074	**3.56**	162	3.42	18.2	17.2	15.8
2,6-DMA-4-NMP	27 500	496	725/707	0.117	0.79	63.4	1.97	n.c.	n.c.	n.c.
2,4,6-NMP	28 700	514	684/670	0.131	1.37	103	2.74	14.1	22.8	15.1
2,6-NMP-4-Morph	37 900	518	683/665	0.094	1.08	93.6	2.64	16.2	18.6	15.0
2,6-Ind-4-DMA	69 800	524	714/702	0.061	0.78	125	2.94	13.3	19.8	17.1
2,6-NdMP-4-DMA	72 000	535	640/628	0.035	2.19	**169**	**6.66**	15.9	17.5	19.0
2,6-NMP-4-OMe	24 900	540	680/670	0.103	1.11	52.8	2.07	13.8	25.1	15.1
2,6-NMP-4-OEt	19 100	540	684/667	0.100	1.03	55.5	2.91	n.c.	n.c.	n.c.
2,6-NMP-4-OH	44 100	510	674/669	0.096	0.45	25.1	0.59	n.c.	n.c.	n.c.
2,6-NMP-4-2MeOPh	24 300	550	691/671	0.097	0.87	47.8	2.03	15.3	34.3	16.4
2,6-Pip-4-2MeOPh	13 200	594	739/711	0.035	1.58	30.1	6.44	n.c.	n.c.	n.c.
2,4-NMP-6-Ph	40 300	528	685/668	0.043	0.48	37.9	2.19	n.c.	n.c.	n.c.
2,4-DMA-6-Me	72 300	562	656/637	0.027	**3.79**	**434**	**22.3**	3.7	16.5	—
2,4-NMP-6-Me	39 000	508	616/622	0.085	2.34	103	3.11	3.6	16.4	—
2,4-DMA-6-BTA	19 400	484	773/684	0.129	0.97	46.2	1.85	n.c.	n.c.	n.c.

The simplest of the model compounds, 2,4,6-DMA exhibits little variation in absorption maxima in organic solvents: the absorption peak generally shifts in the hypsochromic direction with the increase of solvent polarity (from 534 nm in tetrahydrofuran to 526 nm in acetonitrile), with the exception of DMSO (*λ*_abs_ = 542 nm) and 1,1,1,3,3,3-hexafluoroisopropanol (HFIP), where the main absorption peak splits into two. In water, this compound aggregates above 1 μM, as demonstrated by the concentration dependence of its area-normalized absorption spectra (Fig. S143 in SI). The lack of significant solvatochromism implies that the dipole moment of the molecule does not change significantly during vertical excitation. Fluorescence quantum yield decreases with increasing solvent polarity, from 12.2% in THF through 2–4% in more polar organic solvents to 0.06% in TE buffer (although aggregation might influence this latter result). This renders triarylpyryliums promising candidates for *in vivo* polarity sensing.^18,19^ The dye exhibits large Stokes shift (Δ*λ* = 150–200 nm) and despite this and the varying *Φ*_f_ values, the emission maxima are distributed in a relatively narrow range (677–713 nm). This implies that in each of the investigated solvents the excited molecule (and its solvent environment) undergoes significant geometry reorganization before the emission occurs from an intramolecular charge transfer (ICT) state. The fluorescence lifetime of 2,4,6-DMA was also measured in methanol and THF. In both cases biexponential fitting provided the lowest *χ*^2^ values (Table S10 in SI). While in methanol the average lifetime of the two components are in the hundred-picosecond range (*τ*_1_ = 331 ps, *f*_1_ = 93% and *τ*_2_ = 670 ps, *f*_2_ = 7%), in THF both increase over 1 ns, while the fractional contribution of the long component increases (*τ*_1_ = 1.19 ns, *f*_1_ = 47% and *τ*_2_ = 1.48 ns, *f*_2_ = 54%).

With changing the dimethylamino groups to *N*′-methylpiperazin-*N*-yl (NMP), the fluorescence quantum yields (*Φ*_f_) generally decrease in each solvent (a notable exception is water, where very faint luminescence was measured in each case). In 2,6-DMA-4-NMP the shape of the absorption spectrum is highly dependent on the solvent (while for 2,6-NMP-4-DMA the solvent effects are not nearly this pronounced). In THF and methanol the main absorption band of 2,6-DMA-4-NMP consists of two overlapping peaks that shift to the opposite directions in MeCN, in acidified methanol, and an even greater shift was observed in HFIP. In MeCN, the splitting of the main absorption peak is reversed by the addition of a base (0.2% diisopropylethylamine, DIPEA), or 25% ethylene glycol. The independence of the emission spectrum and *Φ*_f_ of the excitation wavelength (measured in HFIP) indicates that the two absorption peaks originate from the same species. This solvatochromicity can be explained by the perturbation of orbital energies by the proximity of a positively charged trialkylammonium group. This effect is influenced both by the acid-base equilibrium of the amine and the extent of H-bonding stabilization of the trialkylammonium ion by the surrounding solvent molecules. According to our TD-DFT modelling of the fluorophore (*vide infra*) the main absorption peak corresponds to the *S*_0_ → *S*_1_ (HOMO → LUMO) and *S*_0_ → *S*_2_ (HOMO-1 → LUMO) excitations, which are similar in energy and comparable in oscillatory strength. HOMO-1 has considerable electron density on the C ring, unlike the HOMO, which is mostly concentrated on the B, A, and D rings, while the electron density of LUMO is highest on the central pyrylium and smaller on the three aryl moieties (see Fig. S171b in SI). Therefore, the presence of a positive charge on the trialkylamine nitrogen increases the energy needed for the HOMO-1 → LUMO excitation by stabilizing the HOMO more than the LUMO and decreases the HOMO → LUMO gap by stabilizing the LUMO more than the HOMO. The presence of hydrogen bond acceptors decreases this effect (hence the disappearance of peak splitting in MeCN, when an amine base or ethylene glycol is added to the solution). On the other hand, in HFIP the H-bonding stabilization of the ammonium ion is weaker than in other polar solvents, because HFIP is both a poor H-bond acceptor and a very strong H-bond donor that saturates the available acceptor sites.^20^

Measurements of *Φ*_f_ in mixtures of MeCN and ethylene glycol (a pair of solvents with different viscosity and almost identical relative permittivity^21^) demonstrated that while viscosity influences the *Φ*_f_ of 2,4,6-DMA minimally, the influence of viscosity is larger for 2,6-DMA-4-NMP and even larger for 2,6-NMP-4-DMA and 2,4,6-NMP (Fig. S140 in SI).

DNA-binding-induced fluorogenicity was quantitatively characterized using two metrics introduced in our previous work.^22^

Absolute Fluorescence Enhancement (AFE) is defined as the product of the extinction coefficient and the derivative of the effective fluorescence quantum yield of the partially DNA-bound dye (*Φ*_f_) with respect to *C*_DNA_ at *C*_DNA_ = 0:
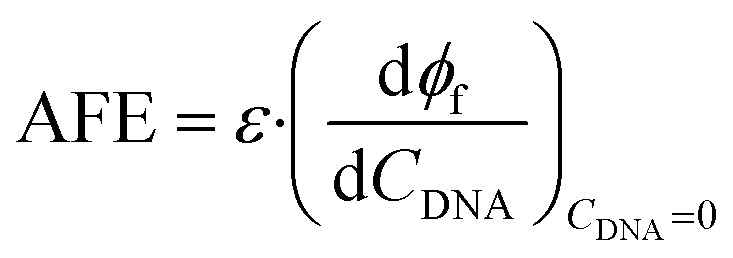


During this measurement the concentration of the dye is fixed at 1 μM. The second factor of the product was approximated by linear regression in the 0–0.8 μM range. Since *ε*·*ϕ*_f_ is expected to be proportional to the flux of the emitted light (*i.e.* the measured parameter in a fluorescence-based quantification of *C*_DNA_), the ratio of AFE values gives us the relative sensitivity provided by different fluorescent dyes in a given experimental setup.

Relative Fluorescence Enhancement (RFE) shows the ratio of the increase of *Φ*_f_ (d*Φ*_f_/d*C*_DNA_) and the fluorescence quantum yield of the non-bound dye (*Φ*^0^_f_):
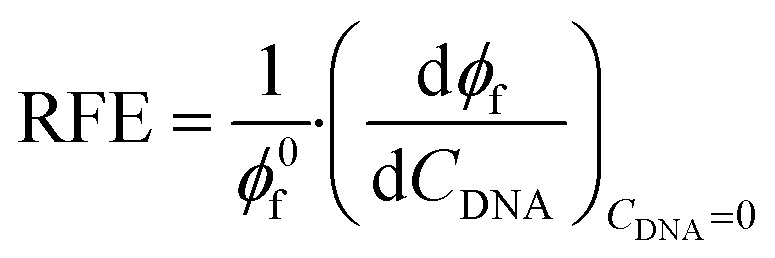


Compared to the previously known reference compound 2,4,6-DMA, the introduction of amine-containing side groups provides 2.7–12.3 times larger *Φ*_f_^DNA^ and similar *Φ*^0^_f_. The highest AFE, RFE, and *Φ*_f_^DNA^ among NMP-substituted triarylpyryliums were measured for 2,6-NMP-4-DMA, which provides an order of magnitude lower limit of detection for DNA sensing than 2,4,6-DMA (see Table S9 in SI). These values are not increased by the introduction of further cyclic ether or tertiary amino groups on the C ring's substituent (2,6-NMP-4-Morph, 2,4,6-NMP), nor by introducing only one NMP substituent on the C ring (2,6-DMA-4-NMP). Tethering the diamino substituents of the B and D rings to the aromatic ring through an ethylene linkage to prevent excited state rotations of the amino groups (2,6-Ind-4-DMA) results in similar Φf0 and AFE values, but lower *Φ*_f_^DNA^ compared to 2,6-NMP-4-DMA. However, changing the tertiary amino groups to quaternary dimethylpiperazinium (2,6-NdMP-4-DMA) slightly increases the AFE, and due to its fainter fluorescence in the absence of DNA its RFE is also higher. Changing the 4-amino substituent of the C ring to less electron-donating substituents (2,6-NMP-4-OMe, 2,6-NMP-4-OEt, 2,6-NMP-4-OH, 2,6-NMP-4-2MeOPh) leads to lower *ε* and AFE. Changing the NMP group of the latter compound to 1-piperidinyl (2,6-Pip-4-2MeOPh, Fig. 2b) results in lower AFE, but its RFE is still higher than that of 2,4,6-DMA. Two further asymmetrical derivatives, 2,4-NMP-6-Ph, and the benzothiazole-bearing 2,4-DMA-6-BTA have also been synthesized and measured, but their AFE and RFE fall short of those of most other pyrylium dyes.

The previously known^13^ diarylpyrylium dye, 2,4-DMA-6-Me exhibits higher AFE and RFE than the triarylpyryliums, mostly due to its larger fluorescence quantum yield in DNA (*Φ*_f_^DNA^ = 3.79%). Unlike in triarylpyryliums, here the change of dimethylamino groups to NMP (2,4-NMP-6-Me) does not ameliorate its DNA sensing ability. These results indicate that the best-performing pyrylium dyes (2,6-NMP-4-DMA, 2,6-NdMP-4-DMA, 2,4-DMA-6-Me) are applicable for the spectroscopical detection of minute quantities of DNA, although they compare unfavourably to thiazole orange-based DNA dyes^22^ according to these metrics (Table S8 in SI).

The Scatchard plots obtained from the DNA titration experiments do not follow the McGhee-von Hippel model of DNA binding (Fig. S163 in SI). This deviation from the expected behaviour is probably due to multimodal binding,^23^ as implied by the somewhat irregular concave shape of some of the DNA titration curves (*e.g.*2,6-NMP-4-DMA, see Fig. 2a). The only exception from this is 2,4-DMA-6-Me, where the binding constant was determined to be *K*_b_ = 2.5 × 10^5^ M^−1^ with a binding site size of 1.4 base pairs.

### Applicability in agarose gel electrophoresis

**Fig. 3:**
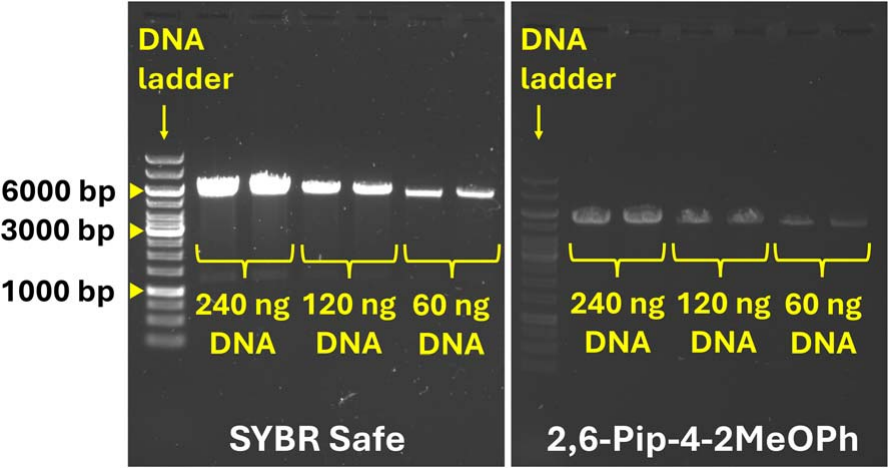
Comparison of SYBR Safe and 2,6-Pip-4-2MeOPh for agarose gel staining using dsDNA samples in different concentrations and GeneRuler 1 kb DNA ladder as reference (middle section of the photograph was cropped for better visibility).

The applicability of the fluorescent pyrylium dyes as gel stains was tested on DNA samples. PCR products were run on an agarose gel with 2,4,6-DMA, 2,6-NMP-4-DMA, 2,6-NdMP-4-DMA, 2,6-Pip-4-2MeOPh, and SYBR Safe (as reference). As observed in the agarose gel electrophoresis images, the tested dyes did not exhibit any nonspecific background fluorescence, but they provided lower emission intensity compared to SYBR Safe (*I*_2,6-Pip-4-2MeOPh_/*I*_SYBR Safe_ = 0.082–0.11 in different lanes, see Fig. S168–169 in SI). 2,6-NMP-4-DMA and 2,6-NdMP-4-DMA were deemed unsuitable for DNA visualization, as they restricted the migration of DNA fragments. However, 2,6-Pip-4-2MeOPh and 2,4,6-DMA did not interfere with gel electrophoresis quality, therefore validating their use for DNA visualization in agarose gels (see Fig. 3 and S165–168 in SI).

### Theoretical study of fluorescence quenching mechanism

There are numerous fluorescence quenching mechanisms that can lead to a decrease or complete loss of emission upon irradiation. These mechanisms include photoinduced electron transfer (PET), twisted intramolecular charge transfer (TICT), triplet state quenching, aggregation-induced quenching, or collisional quenching. In the case of fluorescent sensor molecules, this quenching process can be suppressed in the presence of a specific analyte, resulting in fluorescence activation (“turn-on” response) and enabling quantitative analysis. Based on previously reported results and prevailing understanding, it is reasonable to assume that the DNA-dependent turn-on fluorescence response can be attributed to the steric hindrance imposed by the host macromolecule that restricts the intramolecular rotations of the chromophore. These conformational changes would otherwise enable radiationless relaxation and consequently quench fluorescence. This may occur *via* a non-emissive twisted intramolecular charge transfer (TICT) state, analogously to the well-established DNA sensing mechanism of Thiazole Orange derivatives.^22,24–26^ The assumption that intramolecular twisting is necessary for efficient fluorescence quenching is underpinned by the fact that the *Φ*_f_ values reported for other 2,4,6-triarylpyrylium dyes containing fixed 2- and 6-aryl substituents are two orders of magnitude higher than the *Φ*_f_ of their rotationally free counterparts.^11^ A further argument for the involvement of a TICT intermediate can be found in a study by Lampre *et al.*, in which the transient dichroic spectrum of a postulated TICT intermediate was detected in Kerr ellipsometry experiments conducted using a solution of 4-(4-dimethylaminophenyl)-2,6-diphenylpyrylium in glycerol, ethylene glycol, and ethanol.^27^

**Fig. 4:**
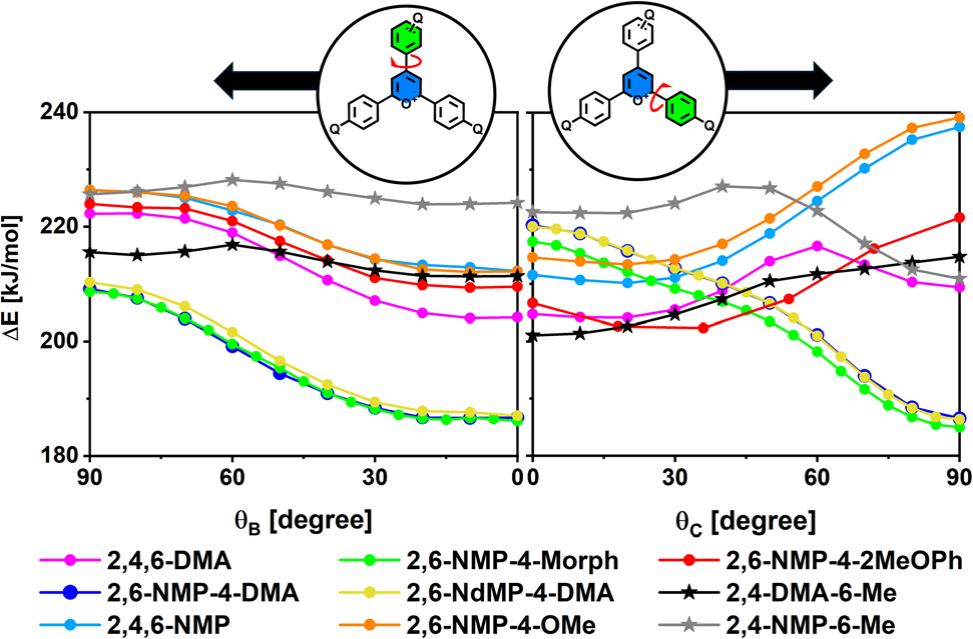
Calculated *S*_1_ scanning energies of different pyrylium dyes as a function of the *θ*_B_ (left) and *θ*_C_ (right) dihedral angles, given relative to the *S*_0_ energy optimum.

Fluorescence quenching through a TICT state would require the excited state energy minimum to lie at a near perpendicularly twisted conformation with broken conjugation between the pyrylium acceptor and one of the aryl donor groups – either the B (or D) ring (*θ*_B_ = 90°, TICT_B_ conformation) or the C ring (*θ*_C_ = 90°, TICT_C_ conformation). This was tested by DFT scanning the *S*_0_, *S*_1_ and *T*_1_ energy surfaces of nine model compounds for the expected rotations using polarizable continuum model (PCM) method to model the effect of the aqueous environment (Fig. 4). One parameter (*θ*_B_ or *θ*_C_) was pre-set at a time and all the other internal parameters were re-optimised in each step of the scan. The result is a Δ*E*(*θ*_i_) function, where the Δ*E* potential energy is given relative to the S_0_ energy minimum. To further validate the applied computational method, the same calculations were performed with 4-(4-dimethylaminophenyl)-2,6-diphenylpyrylium, a pyrylium dye with an experimentally established TICT intermediate. In this case the calculations predicted an S_1_ energy minimum at *θ*_C_ = 90°, consistently with the reported experimental results.^27^

In ground state (*S*_0_) the energy barriers of both aryl rotations (B and C) are calculated to be within the range of 15–40 kJ mol^−1^, with an energy maximum at *θ*_i_ = 90°, referring to a weakly hindered rotation. At the optimal geometry the *θ*_B_, *θ*_C_ and *θ*_D_ dihedral angles range between 10° and 20° with the exception of 2,6-NMP-4-2MeOPh, where the steric repulsion between the methoxy group and the pyrylium C–H forces the A and C ring into a more twisted conformation (*θ*_C_ = 34°, Table 1). Likewise, the energy optima in *T*_1_ lie close to a planar geometry and the perpendicular twisted conformation of the aryl groups is energetically unfavorable (Table S19–21 in SI).

This is not the case, however, in *S*_1_ where the global minimum of Δ*E*(*θ*_C_) at *θ*_C_ = 90° predicts an energetically favorable TICT_C_ geometry for four of the modeled molecules: 2,6-NMP-4-DMA, 2,6-NMP-4-Morph, 2,6-NdMP-4-DMA, and 2,4-NMP-6-Me. Zero calculated oscillator strengths predict these TICT states to be non-emissive. For the perpendicular rotation of the B (or D) rings, the calculations do not predict any global energy minima at around *θ*_B_ = 90°, although the two diarylpyryliums (2,4-DMA-6-Me, 2,4-NMP-6-Me) and 2,6-NMP-4-OMe exhibit small local minima with almost negligible energy barriers (Fig. 4). The simultaneous rotation of two aryl rings, as well as rotations about the aryl–N bonds (*θ*_B_’ and *θ*_C_’), were also investigated for 2,4,6-DMA, but no further energy minima were found (see Table S23 and Fig. S174–S179 in SI). The latter finding is in accordance with the experimental observation that conformationally locking the arylamine groups does not enhance *Φ*^0^_f_ significantly (*Φ*^0^_f_ = 0.061% for 2,6-Ind-4-DMA in comparison to 0.024% for 2,6-NMP-4-DMA), which suggests that aryl-N rotations play a negligible role in fluorescence quenching.

**Fig. 5:**
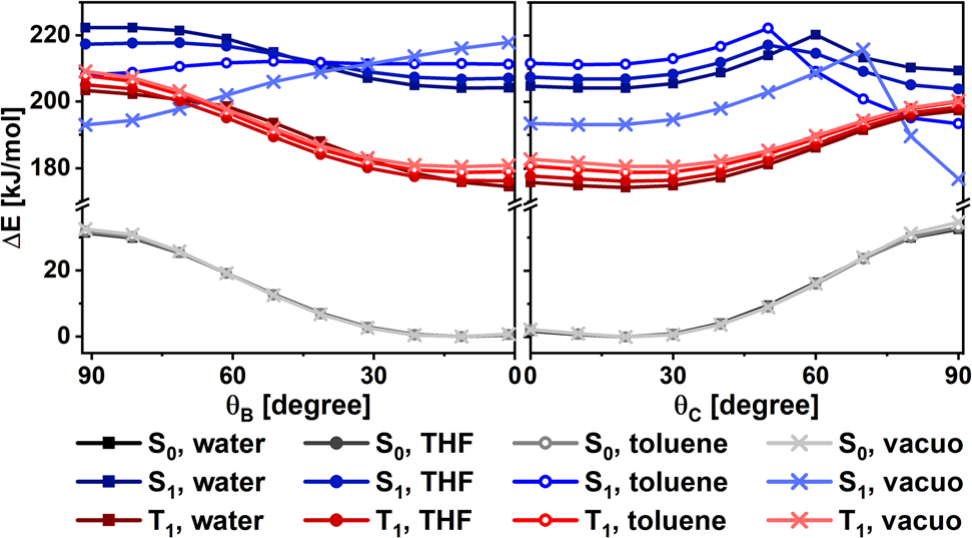
*S*_0_, *S*_1_, and *T*_1_ scanning energies of 2,4,6-DMA as a function of the *θ*_B_ and *θ*_C_ dihedral angles, given relative to the *S*_0_ energy optimum, calculated using different PCM solvent models.

The effect of solvent polarity was also investigated for 2,4,6-DMA using PCM method to model the effect of four media (water, THF, toluene, and vacuum). The obtained Δ*E*(*θ*_i_) curves indicate that by decreasing the polarity of the environment (from water to vacuum) both the TICT_B_ and TICT_C_ conformations become favorable, with the latter being a global energy minimum in THF, toluene, and vacuum (Fig. 5).

### Sensing mechanism inferred from computation and experiment

If TICT quenching is hypothesized to be the dominant radiationless relaxation pathway of di- and triarylpyryliums, one should expect higher fluorescence quantum yields (*Φ*^0^_f_) for the non-bound compounds where TICT is energetically unfavoured. However, the experimentally measured *Φ*^0^_f_ values of 2,4,6-DMA, 2,4,6-NMP, 2,6-NMP-4-OMe, 2,6-NMP-4-2MeOPh, and 2,4-DMA-6-Me are comparable to those of the compounds where TICT is predicted to be favoured. Another counterargument to TICT-based quenching concerns the calculated S_1_ potential surface of 2,4,6-DMA in different media. While the formation of a TICT intermediate is predicted to become less favourable with increasing solvent polarity, the spectroscopical results display almost the opposite tendency, with the highest *Φ*_f_ measured in the least polar solvent (THF). However, it must be noted that our computational approach employs a simple model of the possible solvent interactions (polarisable continuum model: PCM) and excited state dynamics. Effects unaccounted for include specific solvent–fluorophore interactions (*e.g.* hydrogen bonding), as well as the effect of the excited state intramolecular charge shift on hydrogen bonding and protonation equilibria.

Assuming that the applied computational model is reliable, we are prompted to conclude that TICT is likely not the dominant fluorescence quenching mechanism. In this case the overwhelming majority of excited-state molecules must de-excite *via* other relaxation pathways, a significant part of which involves aryl–aryl torsion (as implied by the large *Φ*_f_ of rotationally locked derivatives^11^).

Apart from TICT, other possible radiationless deexcitation pathways were also considered. Attributing the high radiationless relaxation rate in aqueous media to intramolecular PET seems implausible as the pyrylium compounds without potential PET donor groups (*e.g.*2,4,6-DMA, 2,6-Pip-4-2MeOPh) do not exhibit higher *Φ*^0^_f_ and the *Φ*^0^_f_ does not significantly decrease with the number of *N*-methylpiperazinyl groups (*Φ*^0^_f_ = 0.061, 0.117, 0.074, 0.131% for 2,4,6-DMA, 2,6-DMA-4-NMP, 2,4-NMP-6-DMA, and 2,4,6-NMP, respectively). Moreover, at pH = 7.4 tertiary aliphatic amino groups are mostly protonated, and therefore unable to act as PET donors. A further counterargument against PET quenching comes from the theoretical modelling of 2,4,6-NMP. Even if none of the amino groups are protonated, the highest-energy occupied orbital with considerable electron density on the aliphatic amino groups is HOMO-2, therefore electron transfer from this orbital to HOMO (or HOMO-1) would not be favourable (Fig. S172 in SI). Collisional quenching with dissolved oxygen was also considered as a potential contributor for the high radiationless deexcitation rates. However, since the *Φ*_f_ of 2,6-NdMP-4-DMA does not depend significantly on the presence or absence of oxygen (see Fig. S160 in SI), collisional quenching must play only a complementary role. Therefore, the most probable explanation for such radiationless de-excitation mechanisms is internal conversion involving aryl–aryl torsion.

## Conclusions

In conclusion, 14 novel pyrylium-based DNA chemosensors were synthesized and characterized, including 10 symmetrical and one asymmetrical 2,4,6-triarylpyryliums (mostly easily accesible by a one-pot reaction), one 2,4-diarylpyrylium, and one benzothiazole-cyanine-like pyrylium derivative. These dyes fluoresce weakly in aqueous solution and exhibit a fluorescence enhancement upon DNA binding that allows their use as DNA chemosensors (*e.g.* in gel electrophoresis). Their red emission and large Stokes shifts (90–150 nm) are advantageous for fluorescence-based bioimaging by minimizing interference.

The mechanism of their fluorogenicity was studied by DFT methods to investigate the mechanism of fluorescence quenching necessary for the DNA-induced fluorescence enhancement. Although TICT-mediated quenching was initially considered dominant in di- and triarylpyryliums, our computational model did not support this, suggesting TICT plays only a complementary role.

## Author contributions

F. Domahidy: conceptualization, data curation, formal analysis, investigation methodology, (synthesis, spectroscopy), project administration, supervision, visualization, writing – original draft, writing – review & editing. L. Cseri: conceptualization, methodology, writing – review & editing. G. Turczel: investigation, data curation, formal analysis (NMR). B. Huszár: investigation, data curation, methodology (gel electrophoresis testing). B. J. Rózsa: resources, funding acquisition. Z. Mucsi: investigation, data curation, formal analysis, writing – original draft (theoretical calculations); resources, funding acquisition. E. Kovács: conceptualization, formal analysis, investigation (synthesis, spectroscopy), methodology, project administration, resources, supervision, writing – review & editing.

## Conflicts of interest

F. Domahidy, E. Kovács, B. J. Rózsa, Z. Mucsi, and L. Cseri have patent HUP2300414 issued to BrainVisionCenter. The other authors declare that no conflict of interest exists.

## Supplementary Material

RA-015-D5RA04750A-s001

## Data Availability

The data underlying this study are available in the published article and its SI. See DOI: https://doi.org/10.1039/d5ra04750a. NMR FIDs and theoretical raw data can be found at Mendeley Data in Dataset S1[DETAILS].^28^ Additional raw data are available from the corresponding authors upon request.
