# Supplementary material for: Triarylpyrylium-based fluorescent DNA-binding dyes – facile synthesis, substituent effects, and sensing mechanism
Source: RSC Adv. 2025 Aug 22;15(36):29952–9. doi: 10.1039/d5ra04750a (PMC12377229; doi:10.1039/d5ra04750a)
Supplement: RA-015-D5RA04750A-s001 [file RA-015-D5RA04750A-s001.pdf]

Supplementary Information (SI) for RSC Advances.
This journal is © The Royal Society of Chemistry 2025

# Supporting Information

# for

# Triarylpyrylium-based fluorescent DNA-binding dyes – facile synthesis, substituent effects, and sensing mechanism

Farkas Domahidy,[a,b] Levente Cseri,[a] Gábor Turczel,[c] Blanka Huszár,[a] Balázs J. Rózsa,[a,d,e] Zoltán Mucsi,[a,f] and Ervin Kovács[a,g]

[a] BrainVisionCenter
Liliom utca 43-45, H-1094 Budapest, Hungary

[b] Hevesy György PhD School of Chemistry, Eötvös Loránd University
Pázmány Péter sétány 1/A, H-1117 Budapest, Hungary

[c] NMR Research Laboratory, HUN-REN Research Centre for Natural Sciences
Magyar tudósok körútja 2, H-1117 Budapest, Hungary

[d] Two-Photon Measurement Technology Research Group, Pázmány Péter Catholic University
Práter utca 50/a, H-1083 Budapest, Hungary

[e] Laboratory of 3D functional network and dendritic imaging, HUN-REN Institute of Experimental Medicine
Szigony utca 43, H-1083 Budapest, Hungary

[f] Faculty of Materials and Chemical Sciences, University of Miskolc
H-3515 Miskolc, Hungary

[g] Institute of Materials and Environmental Chemistry, HUN-REN Research Centre for Natural Sciences
Magyar tudósok körútja 2, H-1117 Budapest, Hungary
E-mail: kovacs.ervin@ttk.hu

## Table of Contents

1. General remarks ........ 2
2. Synthetic procedures ........ 3
3. NMR and HRMS spectra of the prepared compounds ........ 21
4. Spectroscopic characterization ........ 91
5. Testing in agarose gel electrophoresis ........ 112
6. Theoretical studies ........ 116
7. References ........ 187

# 1. General remarks

All reagents and solvents were purchased from Merck, Fluorochem, or VWR. Deionized water (DI) was prepared using a Milli-Q RiOs-DI-3UV system (>10 MΩ cm).

Microwave-assisted reactions were conducted in an Anton-Paar Monowave 450 microwave reactor.

Thin layer chromatography (TLC) was performed on commercially available pre-coated TLC plates (Merck Silica gel 60 $F_{254}$ aluminium sheets) and spots were visualized by UV light or by exposing it with the aqueous solution of $(NH_4)_6Mo_7O_{24}$, $Ce(SO_4)_2$ and sulfuric acid.

Flash column chromatography was performed by an Interchim puriFlash xs 520Plus flash chromatograph using gradient elution on normal phase mode (Silica gel 60, 40 – 63 µm from VWR).

Gradient elution preparative RP-HPLC purifications were conducted on a Teledyne ISCO ACCQPrep® HP150 chromatography system equipped with a Phenomenex Gemini 10 µm NX-C18 110 Å column.

For reaction monitoring, a Shimadzu LC-40D XR UPLC-MS system was used equipped with a SIL-40C XR autosampler, SPD-M40 photodiode array detector and an LCMS-2020 DUIS Mass Spectrometer operated in alternating negative and positive modes. An Ascentis Express C18, 2 µm UHPLC column (L × I.D. 5 cm × 2.1 mm) was used at 40 °C provided by a CTO-40s column oven. Gradient elution was used either using 0.1% v/v TFA in water (A) and MeCN (B). NMR spectra were obtained on Varian Unity INOVA spectrometers operating at an equivalent $^1H$ frequency of 300, 400, 500 and 600 MHz. Spectra were acquired at room temperature unless noted otherwise. Notation for the $^1H$ NMR spectral splitting patterns includes singlet (s), doublet (d), triplet (t), quartet (q), broad (br) and multiplet/overlapping peaks (m). Chemical shifts of the resonances are given as δ values in ppm, the coupling constants (*J*) are expressed in Hz. Individual resonances were assigned based on 2D NMR experiments shown in this document. Accurate mass measurements were carried out on a high-resolution Q-Exactive Focus hybrid quadrupole-orbitrap mass spectrometer (Thermo Fisher Scientific, Bremen, Germany) used with a heated electrospray ionization source. Samples were dissolved in acetonitrile-water 1:1 (v/v) solvent mixture containing 0.1% (v/v) formic acid. Flow injection analysis was performed using a 50 µL $min^{-1}$ eluent flow provided by a Thermo Scientific UPLC. The cationic molecules were detected in positive ionization ESI.

Absorption spectra were recorded on a Shimadzu UV-1900i UV-Vis spectrophotometer in 1 × 1 cm polymethacrylate cuvettes. Emission spectra were recorded on a Shimadzu RF-6000 spectrofluorimeter. UVASol. DMSO (Supelco) was used for the preparation of stock solutions.

## 2. Synthetic procedures

### 2.1. Overview of the synthetic procedures

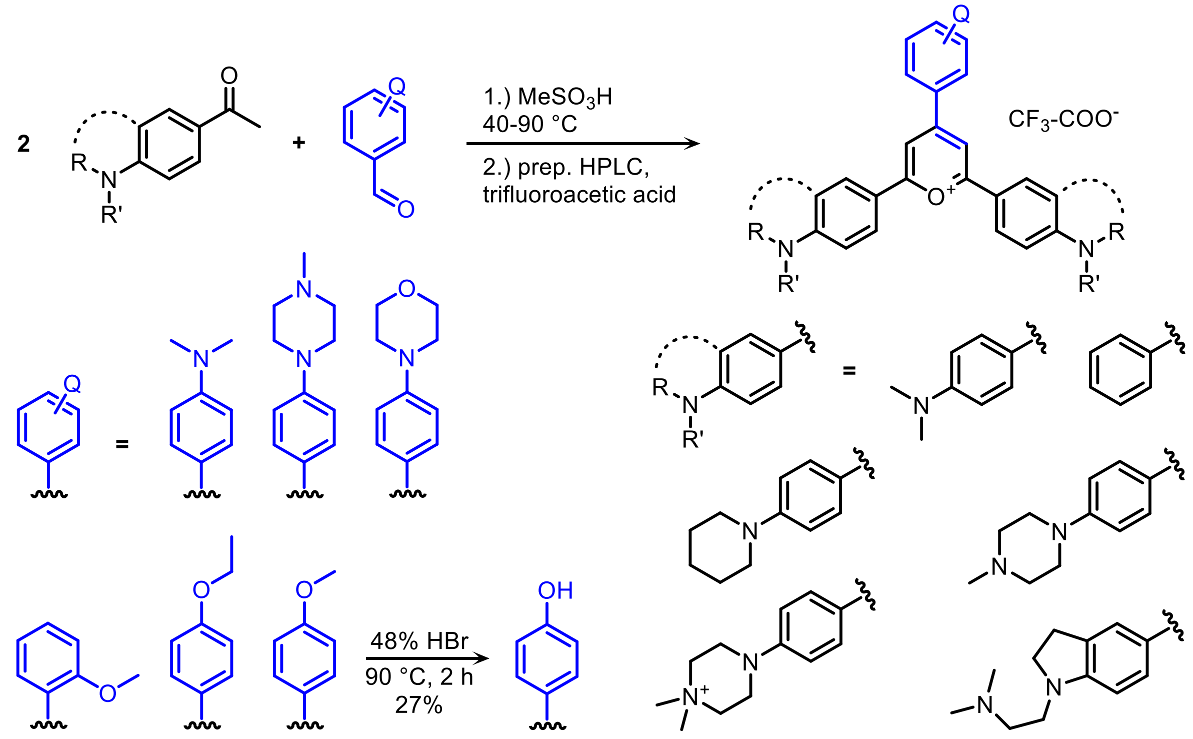


*Figure S1 – Synthesis of triarylpyrylium dyes*

### 2.2. Synthesis of intermediates

**Overview**

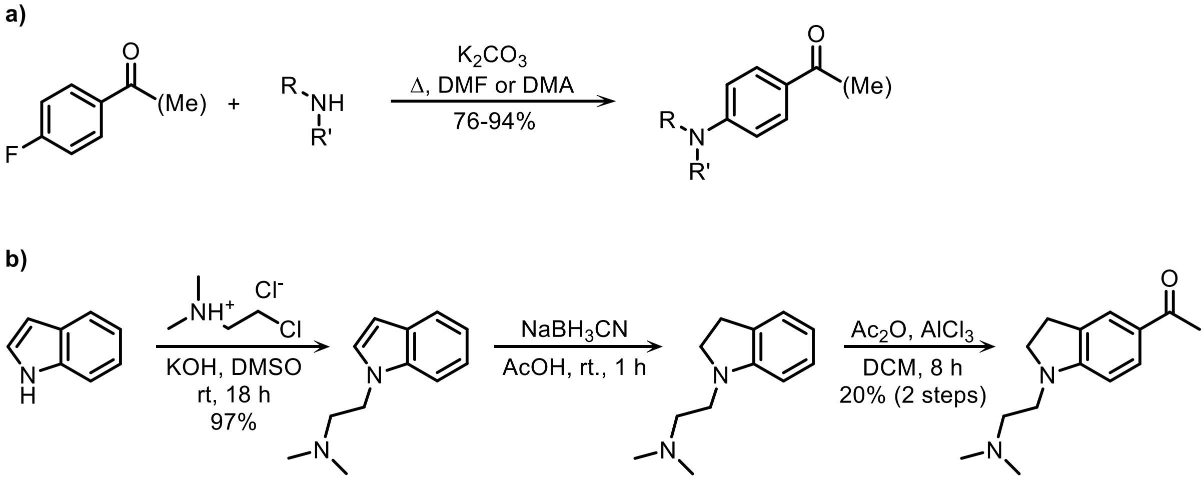


*Figure S2 – Synthesis of intermediates. a) Synthesis of 4-amino substituted benzaldehydes and acetophenones. b) Synthesis of 1-(1-(2-(dimethylamino)ethyl)indolin-5-yl)ethan-1-one*

**4-Morpholinobenzaldehyde**

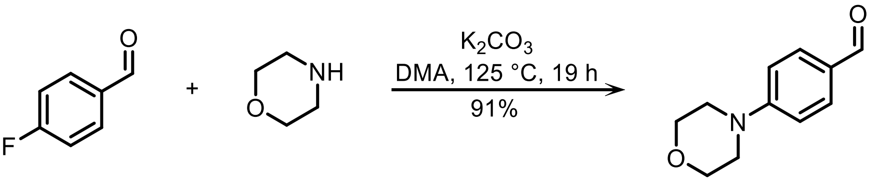


$K_2CO_3$ (1,28 g, 9.27 mmol, 2 eq.), morpholine (530 µL, 525 mg, 6.02 mmol, 3 eq.) and 4-fluorobenzaldehyde (500 µL, 575 mg, 4.63 mmol, 1 eq.) were dissolved in 10 mL anhydrous N,N-dimethylacetamide and stirred at 125 °C for 19 h. The solvent and the starting materials were removed by evaporation at 90 °C. The residue was dissolved in DCM, washed with distilled water and cc. NaCl, dried over $MgSO_4$, and concentrated *in vacuo*. The product (803.2 mg, 91%) is a yellowish oil that crystallized overnight.

$^1$H NMR (400 MHz, $CDCl_3$) δ (ppm): 9.80 (s, 1H), 7.77 (d, J=8.8 Hz, 2H), 6.92 (d, J=8.8 Hz, 2H), 3.86 (t, J=4.9 Hz, 4H), 3.35 (t, J=4.9 Hz, 4H).

$^{13}$C{$^1$H} NMR (101 MHz, $CDCl_3$) δ (ppm): 190.7, 155.3, 131.9, 127.8, 113.6, 66.6, 47.4.

Data is in accordance with literature reports.[1]

**4-(4-Methylpiperazin-1-yl)benzaldehyde**

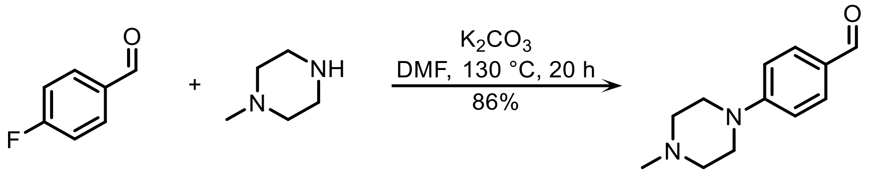


$K_2CO_3$ (1.11 g, 8.06 mmol, 2 eq.), 1-methylpiperazine (447 µL, 404 mg, 4.03 mmol, 1 eq.) and 4-fluorobenzaldehyde (435 µL, 500 mg, 4.04 mmol, 1 eq.) were dissolved in 10 mL anhydrous dimethylformamide and stirred at 130 °C for 20 h. The solvent and the starting materials were removed by evaporation at 90 °C. The residue was dissolved in DCM, washed with distilled water and cc. NaCl, dried over $MgSO_4$, and concentrated *in vacuo*. The product is 709.5 mg (86%) yellowish oil that crystallized overnight.

$^1$H NMR (400 MHz, $CDCl_3$) δ (ppm): 9.77 (s, 1H), 7.77-7.73 (m, 2H), 6.94-6.90 (m, 2H), 3.43-3.39 (m, 4H), 2.57-2.53 (m, 4H), 2.35 (s, 3H).

$^{13}$C{$^1$H} NMR (101 MHz, $CDCl_3$) δ (ppm): 190.6, 155.1, 132.0, 127.2, 113.7, 54.8, 47.2, 46.2.

Data is in accordance with literature reports.[2]

**1-(4-(Piperidin-1-yl)phenyl)ethan-1-one**

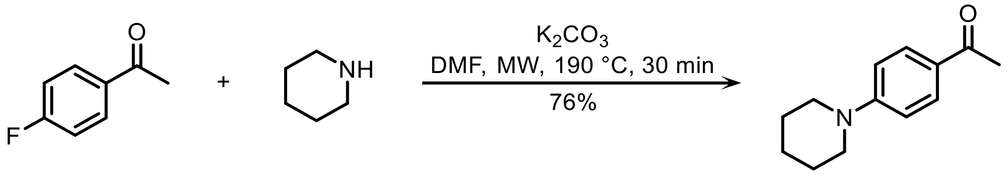


$K_2CO_3$ (1131 mg, 8.18 mmol, 2 eq.), piperidine (404 µL, 348 mg, 4.09 mmol, 1 eq.) and 4-fluoroacetophenone (500 µL, 565 mg, 4.09 mmol, 1 eq.) were dissolved in 8 mL anhydrous dimethylformamide and heated to 190 °C for 30 minutes in microwave reactor. The solvent and the starting materials were removed by evaporation at 90 °C. The residue was dissolved in DCM, washed with distilled water and cc. NaCl, dried over $MgSO_4$, and concentrated *in vacuo*. The product is 635.1 mg (76%) light brown crystalline solid.

$^{1}$H NMR (400 MHz, $CDCl_3$) δ (ppm): 7.85 (d, J=9.0 Hz, 2H), 6.85 (d, J=9.0 Hz, 2H), 3.38-3.34 (m, 4H), 2.51 (s, 3H), 1.70-1.33 (m, 6H)

$^{13}$C{$^{1}$H} NMR (101 MHz, $CDCl_3$) δ (ppm): 196.6, 154.5, 150.8, 130.6, 126.8, 113.3, 48.7, 26.2, 25.5, 24.5.

Data is in accordance with literature reports.[3]

**1-(4-(4-Methylpiperazin-1-yl)phenyl)ethan-1-one**

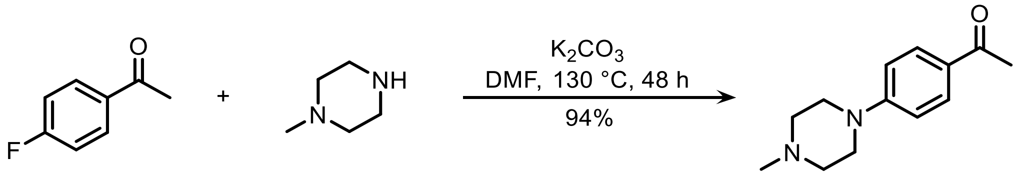


$K_2CO_3$ (2.26 g, 16.4 mmol, 2 eq.), 1-methylpiperazine (998 µL, 901 mg, 9.0 mmol, 1.1 eq.) and 4-fluoroacetophenone (1000 µL, 1130 mg, 8.18 mmol, 1 eq.) were dissolved in 12 mL dimethylformamide and stirred at 130 °C for 2 days. The solvent and the starting materials were removed by evaporation at 90 °C. The residue was dissolved in DCM, washed with distilled water and cc. NaCl, dried over $MgSO_4$, and concentrated *in vacuo*. The product is 1677 mg (94%) light brown crystalline solid.

$^{1}$H NMR (400 MHz, $CDCl_3$) δ (ppm): δ= 7.86 (d, J=9.0 Hz, 2H), 6.86 (d, J=9.0 Hz, 2H), 3.39-3.34 (m, 4H), 2.56-2.52 (m, 4H), 2.51 (s, 3H, s), 2.34 (s, 3H).

Data is in accordance with literature reports.[4]

**4-(4-Acetylphenyl)-1,1-dimethylpiperazin-1-ium iodide**

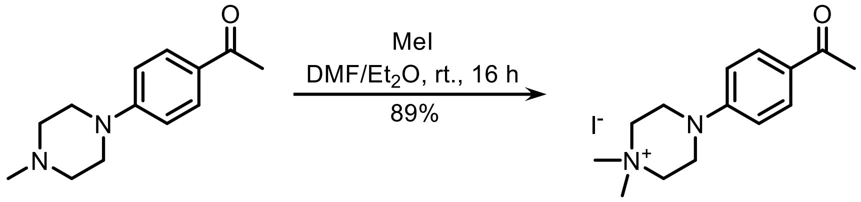


1-(4-(4-Methylpiperazin-1-yl)phenyl)ethan-1-one (448 mg, 2.05 mmol, 1 eq.) and methyl iodide (320 µL, 728 mg, 2.5 eq.) were dissolved in the mixture of diethyl ether (5 mL) and DMF (2.5 mL) and stirred in darkness at room temperature. After 16 h the product was precipitated by the addition of 50 mL diethyl ether and filtered. The product is 656.8 mg (89%) yellowish white powder.

$^{1}$H NMR (400 MHz, DMSO-$d_6$) δ (ppm): 7.87 (d, J=8.5 Hz, 2H, $CH_3$-(CO)-C=C*H*), 7.08 (d, J=8.5 Hz, 2H, N-C=C*H*), 3.73-3.69 (m, 4H, $(CH_3)_2N^+(-CH_2-CH_2-)_2$), 3.57-3.52 (m, 4H, $(CH_3)_2$N+$(-CH_2-CH_2-)_2$), 3.21 (s, 3H, $N^+(-CH_3)_2$), 2.48 (s, 3H, (CO)-$CH_3$).

$^{13}$C{$^{1}$H} NMR (101 MHz, DMSO-$d_6$) δ (ppm): 195.9 (C=O), 152.5 (N-$C_{Ar}$), 130.0 (CH3-(CO)-C=CH), 127.8 ((C=O)-$C_{Ar}$), 113.7 (N-C=CH), 59.8 ($(CH_3)_2$N+$(-CH_2-CH_2-)_2$), 50.3 ($N^+(-CH_3)_2$), 40.7 ($(CH_3)_2$N+$(-CH_2-CH_2-)_2$), 26.3 ((CO)-$CH_3$).

**2-(1H-indol-1-yl)-N,N-dimethylethan-1-amine**

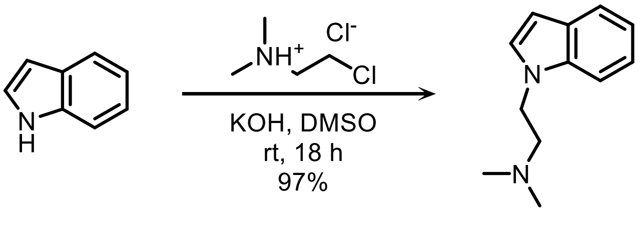

Under nitrogen atmosphere, indole (2.00 g, 17.1 mmol, 1 eq.) was dissolved in 12 mL DMSO. To this solution KOH (4.31 g, 76.8 mmol, 4.5 eq.) was added. The reaction mixture was then cooled to 0 °C and dimethylaminoethyl chloride hydrochloride (2.705 g, 18.8 mmol, 1.1 eq.) was added. The solution was then stirred at room temperature. After 18 h the mixture was diluted with distilled water and diethyl ether. The phases were separated and the aqueous phase was extracted by ether three times. The organic phases were washed with distilled water and cc. NaCl, dried over $MgSO_4$, and concentrated *in vacuo*. The crude product (3.123 g, 97%, pale yellow oil) was used without further purification.

$^1$H NMR (400 MHz, $CDCl_3$) δ (ppm): 7.65 (d, J=7.9 Hz, 1H), 7.37 (dd, $J_1$=8.2 Hz, $J_2$=0.9 Hz, 1H), 7.26-7.20 (m, J=3.1 Hz, 1H), 7.15 (d, J=3.1 Hz, 1H), 7.14-7.09 (m, 1H), 6.51 (dd, $J_1$=3.1 Hz, $J_2$=0.9 Hz 1H), 4.25 (t, J=7.2 Hz, 2H), 2.71 (t, J=7.2 Hz, 2H), 2.32 (s, 6H).

$^{13}$C{$^1$H} NMR (101 MHz, $CDCl_3$) δ (ppm): 135.9, 128.6, 128.0, 121.5, 121.0, 119.3, 109.2, 101.3, 59.0, 45.8, 44.8.

Data is in accordance with literature reports.[5]

**2-(Indolin-1-yl)-N,N-dimethylethan-1-amine**

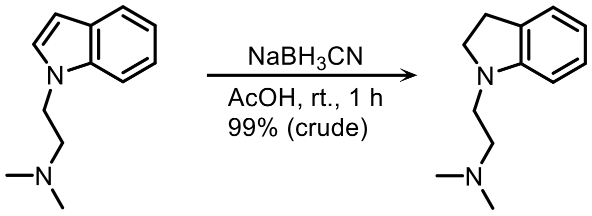


Crude 2-(1H-indol-1-yl)-N,N-dimethylethan-1-amine (1496 mg., 7.95 mmol, 1 eq) was dissolved in 30 mL acetic acid under $N_2$. Sodium cyanoborohydride (1498 mg, 23.8 mmol, 3 eq.) was added in small portions. The solution was stirred at room temperature for 1 h and poured into an excess of 5% NaOH/$H_2O$. This was then extracted by DCM; the organic phase was washed with distilled water and cc. NaCl, dried over $MgSO_4$, and concentrated *in vacuo*. 1.499 g (yellowish liquid; yield: 99%; NMR purity: 90%) crude product was obtained. The crude product was used without further purification.

$^1$H NMR (400 MHz, $CDCl_3$) δ (ppm): 7.09-7.04 (m, 2H), 6.64 (t, J=7.3 Hz, 1H), 6.50 (d, J=7.9 Hz, 1H), 3.38 (t, J=8.3 Hz, 1H), 3.20 (t, J=7.2 Hz, 1H), 2.97 (t, J=8.4 Hz, 1H), 2.54 (t, J=7.2 Hz, 1H), 2.32 (s, 6H).

$^{13}$C{$^1$H} NMR (101 MHz, $CDCl_3$) δ (ppm): 152.6, 130.1, 127.4, 124.5, 117.6, 107.0, 57.1, 53.7, 48.0, 46.0, 28.7.

Data is in accordance with literature reports.[6]

**1-(1-(2-(Dimethylamino)ethyl)indolin-5-yl)ethan-1-one**

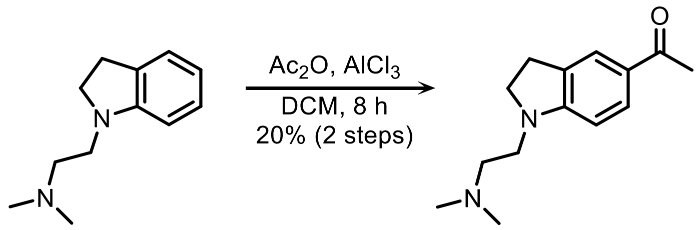


In a pre-dried flask (8.4 g, 63.1 mmol, 12 eq.) $AlCl_3$ was suspended in 18 mL dry DCM. At 0 °C acetic anhydride (1.49 mL, 1.61 g, 15.8 mmol, 3 eq.) was added dropwise. The reaction mixture was stirred at 20 °C for 20 minutes. In a separate dried flask, (1.00 g, 5.26 mmol, 1 eq.) 2-(indolin-1-yl)-N,N-dimethylethan-1-amine was dissolved in 12 mL dry DCM and it was added to the acetic anhydride. The

mixture was stirred at room temperature for 8 h and poured on ice. The solution was then stirred vigorously and basified with the dropwise addition of 40% $NaOH/H_2O$ until the initially precipitating $Al(OH)_3$ dissolved. This was then extracted by DCM; the organic phase was washed with distilled water and cc. NaCl, dried over $MgSO_4$, and concentrated *in vacuo*.

The crude product was purified by flash chromatography (on silica gel; eluent: DCM-MeOH 0-15%) and subsequently by preparative RP-HPLC (stationary phase: C18 silica; eluent: 0.4 g/l $NH_4HCO_3/H_2O$ – MeCN gradient). 242 mg (20%) pure product was obtained as a yellowish liquid.

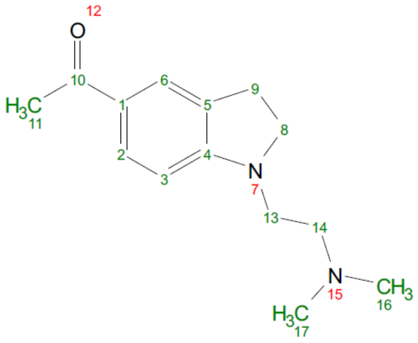

$^1$H NMR (500 MHz, $CDCl_3$) δ (ppm): 7.73 (dd, J = 8.3, 1.8 Hz, 1H, *2*), 7.68 – 7.63 (m, 1H, *6*), 6.34 (d, J = 8.3 Hz, 1H, *3*), 3.58 (t, J = 8.6 Hz, 2H, *8*), 3.30 (t, J = 7.1 Hz, 2H, *13*), 3.06 – 2.98 (m, 2H, *9*), 2.52 (t, J = 7.1 Hz, 2H, *14*), 2.47 (s, 3H, *11*), 2.29 (s, 6H, *16*, *17*).

$^{13}$C{$^1$H} NMR (101 MHz, $CDCl_3$) δ (ppm): 196.2 (*10*), 156.2 (*4*), 130.9 (*2*), 129.7 (*5*), 127.0 (*1*), 124.8 (*6*), 104.2 (*3*), 56.7 (*14*), 52.9 (*8*), 46.1 (*13*), 45.9 (*16*, *17*), 27.7 (*9*), 26.2 (*11*).

HRMS (ESI positive mode): m/z calculated for $C_{14}H_{21}N_2O^+$ $[M]^+$: 233.1649, found: 233.1645.

**3-Methyl-2-methylthiobenzothiazolium *p*-toluenesulfonate**

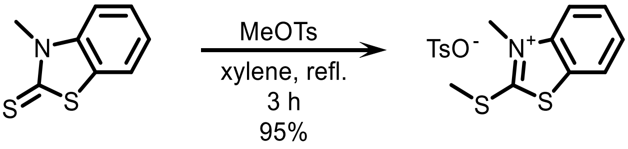


3-Methylbenzothiazole-2-(3*H*)-thione (1.5 g, 8.27 mmol, 1 eq.) was dissolved in 15 mL *p*-xylene. Methyl-*p*-toluenesulfonate (2.37 mL, 15.7 mmol, 1.9 eq.) was added and the solution was refluxed for 3 hours. The solvent was evaporated, and the crude product was suspended in diethyl ether. The yellowish precipitate was filtered, washed with diethyl ether, and dried *in vacuo*. Yield: 2.89 g (95%).

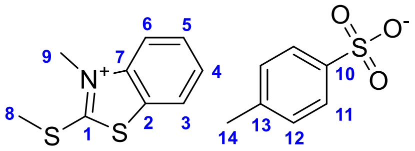

$^1$H NMR (500 MHz, $CD_3OD$) δ (ppm): 8.23 (dd, $J$ = 8.3, 1.1 Hz, 1H, *3*), 8.12 – 8.06 (m, 1H, *6*), 7.87 (ddt, $J$ = 8.4, 7.5, 1.0 Hz, 1H, *5*), 7.75 (ddd, $J$ = 8.2, 7.3, 0.9 Hz, 1H, *4*), 7.70 (d, $J$ = 7.9 Hz, 2H, *11*), 7.22 (d, $J$ = 8.3 Hz, 2H, *12*), 4.17 (d, $J$ = 0.8 Hz, 3H, *9*), 3.14 (d, $J$ = 0.9 Hz, 3H, *8*), 2.37 (s, 3H, *14*).

$^{13}$C{$^1$H} NMR (126 MHz, $CD_3OD$) δ (ppm): 181.9 (*1*), 142.8 (*7*) 142.3 (*10*), 140.2 (*13*), 129.4 (*5*), 128.5 (2), 128.4 (*12*), 127.2 (*4*), 125.6 (*11*), 123.2 (*3*), 115.1 (*6*), 35.5 (*9*), 19.9 (*14*), 17.1 (*8*).

HRMS (ESI positive mode): m/z calculated for $C_{21}H_{21}N_2S^+$ $[M]^+$: 196.0250, found: 196.0249.

Data is in accordance with literature reports.[7]

## 2.3. Synthesis of diaryl- and triarylpyrylium dyes

### 2,4,6-DMA - 2,4,6-tris(4-(dimethylamino)phenyl)pyrylium trifluoroacetate

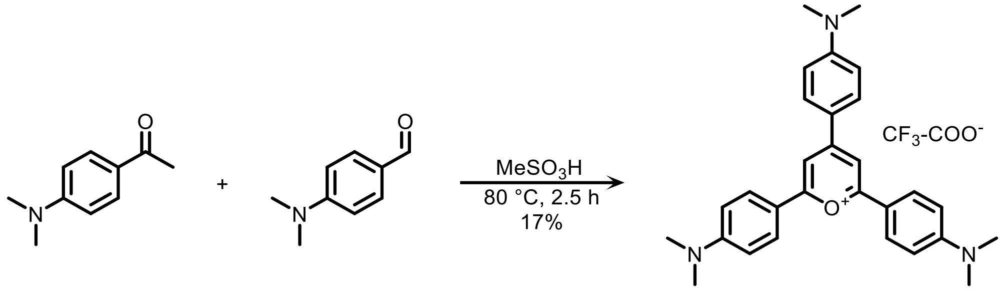


4-Dimethylaminobenzaldehyde (91.4 mg, 0.613 mmol, 1 eq.) and 4-dimethylaminoacetophenone (200 mg, 1.23 mmol, 2 eq.) were dissolved in 1 mL methanesulfonic acid and stirred at 80 °C for 2.5 h. The solution was poured into 30 mL distilled water and extracted by DCM, washed with distilled water, dried over $MgSO_4$, and concentrated. The crude product was purified by RP-HPLC (stationary phase: C18 silica; eluent: 0.2% TFA/$H_2O$ – MeCN gradient). 58.4 mg (17%) dark purple crystalline product was obtained.

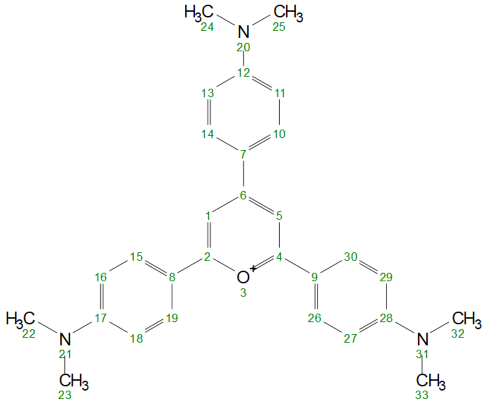


$^1$H NMR (400 MHz, DMSO-$d_6$) δ (ppm): 8.31-8.27 (m, 2H, *10, 14*), 8.13-8.09 (m, 6H, *15, 19, 26, 30*), 8.09 (s, 6H, *1, 5*), 6.84-6.81 (m, 6H, *11, 13, 16, 18, 27, 29*), 3.15 (s, 6H, *24, 25*), 3.09 (s, 12H, *22, 23, 32, 33*).

$^{13}$C{$^1$H} NMR (101 MHz, DMSO-$d_6$) δ (ppm): 165.2 (*2, 4*), 157.1 (*6*), 154.4 (*12*), 153.6 (*17, 28*), 131.6 (*10, 14*), 129.3 (*15, 19, 26, 30*), 118.8 (*7*), 115.7 (*8, 9*), 112.1 (*11, 13*), 111.9 (*16, 18, 27, 29*), 105.2 (*1, 5*), 39.6 (*24, 25*), 39.5 (*22, 23, 32, 33*).

HRMS (ESI positive mode): m/z calculated for $C_{29}H_{32}N_3O^+$ [M]$^+$: 438.2540, found: 438.2579

### 2,6-NMP-4-DMA - 4,4'-((4-(4-(dimethylamino)phenyl)pyrylium-2,6-diyl)bis(4,1-phenylene))bis(1-methylpiperazin-1-ium) tris(trifluoroacetate)

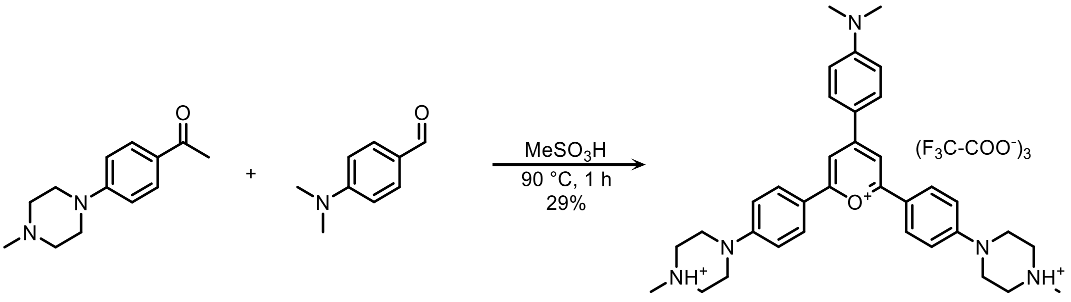


1-(4-(4-Methylpiperazin-1-yl)phenyl)ethan-1-one (125 mg, 0.57 mmol, 2 eq.) and 4-dimethylaminobenzaldehyde (43 mg, 0.29 mmol, 1.0 eq.) were dissolved in 1.0 mL methanesulfonic acid and stirred at 90 °C for 1 h. The solution was added to 20 mL diethyl ether and stirred. The ether phase was then discarded and the viscous precipitate was washed with ether two times. The crude product was purified by RP-HPLC (stationary phase: C18 silica; eluent: 0.2% TFA/$H_2O$ – MeCN gradient). 73 mg (29%) purple product (tris(trifluoroacetate) salt) was obtained.

Structure was elucidated from mono(trifluoroacetate) salt.

$^1$H NMR (400 MHz, $CDCl_3$) δ (ppm): 8.34 (d, J=9.1 Hz, 2H, 28, 32), 8.12 (d, J=9.0 Hz, 4H, 8, 18, 33, 36), 7.98 (s, 2H, 3, 5), 6.94 (d, J=8.9 Hz, 4H, 9, 19, 35, 37), 6.64 (d, J=9.0 Hz, 2H, 29, 31), 3.38 (broad t, J≈4.7 Hz, 8H, 12, 16, 22, 26), 2.94 (s, 6H, 40, 41), 2.54 (broad t, J≈4.7 Hz, 8H, 13, 15, 23, 25), 2.36 (s, 6H, 35, 38).

$^{13}$C{1H} NMR (101 MHz, $CDCl_3$) δ (ppm): 164.7 (2, 6), 157.6 (4), 154.4 (30), 153.8 (10, 20), 132.3 (28, 32), 129.4 (8, 18, 33, 36), 119.0 (27), 118.2 (7, 17), 114.2 (9, 19, 34, 37), 112.5 (29, 31), 106.8 (3, 5), 54.5 (13, 15, 23, 25), 46.7 (12, 16, 22, 26), 46.0 (35, 38), 40.0 (40, 41).

HRMS (ESI positive mode): m/z calculated for $C_{35}H_{42}N_5O^+$ $[M]^+$: 548.3384, found: 548.3395.

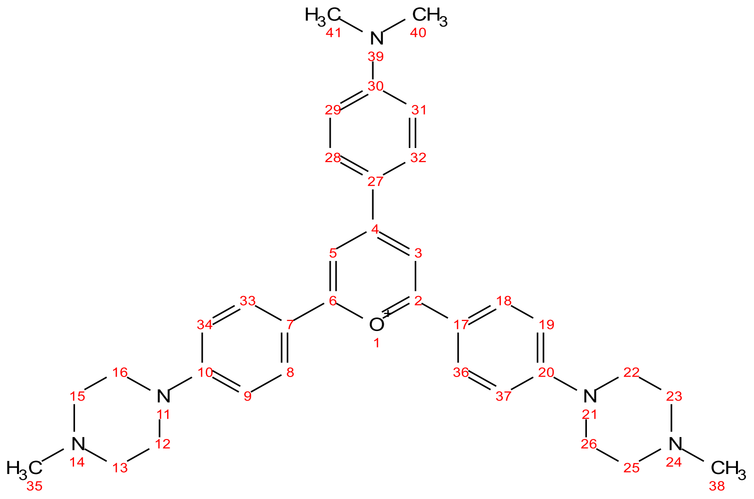

**2,6-DMA-4-NMP - 2,6-bis(4-(dimethylamino)phenyl)-4-(4-(4-methylpiperazin-1-yl)phenyl)pyrylium**

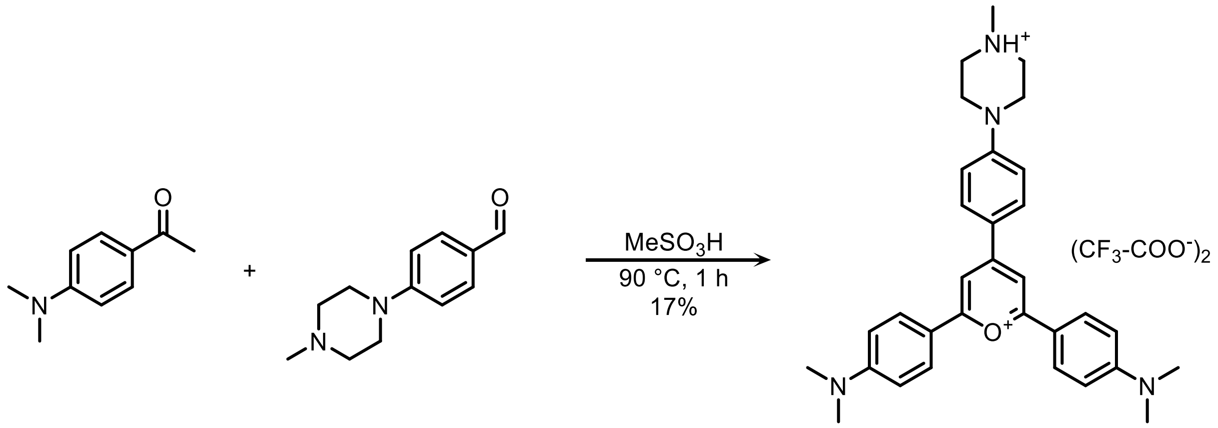


4-Dimethylaminoacetophenone (250 mg, 1.53 mmol, 2 eq.) and 4-(4-methylpiperazin-1-yl)benzaldehyde (156 mg, 0.766 mmol, 1 eq.) were dissolved in 1.5 mL methanesulfonic acid and stirred at 80 °C for 3 h 10 min. The solution was poured on ice, extracted with DCM, washed with $NaHCO_3$ and cc. NaCl, dried over $MgSO_4$, and concentrated *in vacuo*. The crude product was redissolved in MeCN, precipitated by the addition of diethyl either, filtered, and washed with ether. The precipitate was purified and isolated as a trifluoroacetate salt by RP-HPLC (stationary phase: C18 silica; eluent: 0.2% TFA/$H_2O$ – MeCN gradient). The product in 93.7 mg (17%) amorphous purple solid.

$^1$H NMR (300 MHz, DMSO-$d_6$) δ (ppm): 8.43 – 8.34 (m, 2H, *20, 33*), 8.22 (s, 2H, *3, 5*), 8.20-8.14 (m, 4H, *8, 12, 14, 18*), 7.22-7.16 (m, 2H, *21, 34*), 6.88-6.81 (m, 4H, *9, 11, 15, 17*), 4.30 (brs, 2H, *24a, 28a*), 3.58 (brs, 2H, *25a, 27a*), 3.23 (brs, 4H, *24b, 25b, 27b, 28b*), 3.10 (s, 12H, *30, 32, 36, 37*), 2.90 (s, 3H, *35*).

$^{13}C\{^1H\}$ NMR (75 MHz, DMSO-$d_6$) δ (ppm): 166.0 (*2, 6*), 157.3 (*4*), 153.6 (*10, 16*), 153.2 (*22*), 131.2 (*20, 33*), 129.5 (*8, 12, 14, 18*), 122.0 (*19*), 115.4 (*7, 13*), 114.3 (*21, 34*), 111.9 (*9, 11, 15, 17*), 106.3 (*3, 5*), 51.9 (*25, 27*), 43.7 (*24, 28*), 42.1 (*35*), 39.7 (*30, 32, 36, 37*).

HRMS (ESI positive mode): m/z calculated for $C_{32}H_{37}N_4O^+$ $[M]^+$: 494.2962, found: 494.2991.

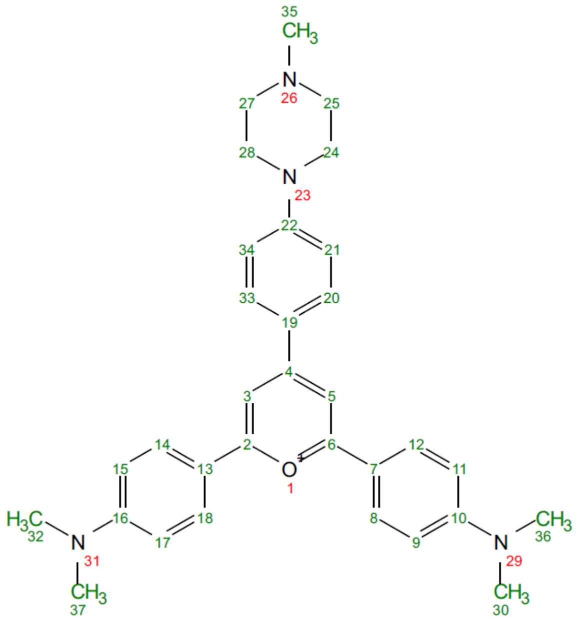

**2,4,6-NMP - 4,4',4''-(pyrylium-2,4,6-triyltris(benzene-4,1-diyl))tris(1-methylpiperazin-1-ium) tetrakis(trifluoroacetate)**

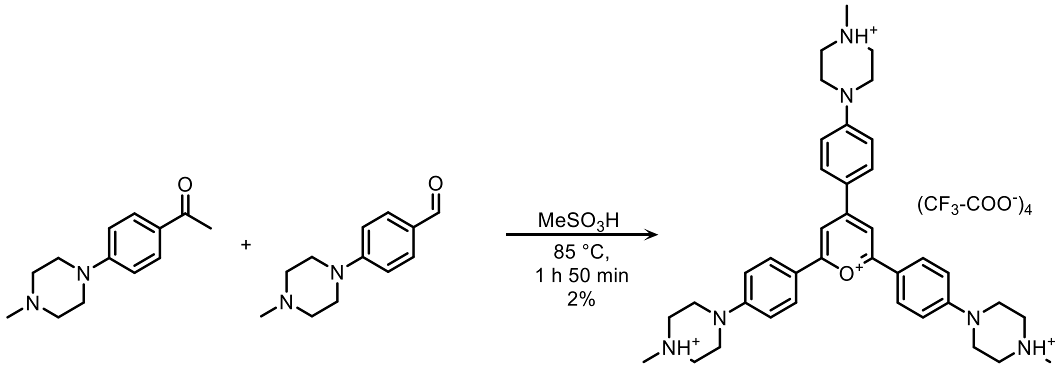


4-(4-Methylpiperazin-1-yl)benzaldehyde (94 mg, 0.458 mmol, 1 eq.) and 1-(4-(4-methylpiperazin-1-yl)phenyl)ethan-1-one (200 mg, 0.916 mmol, 2 eq.) were dissolved in 1 mL methanesulfonic acid and stirred at 85 °C for 2 h 40 min. The solution was poured on ice, extracted with DCM, washed with $NaHCO_3$ and cc. NaCl, dried over $MgSO_4$, and concentrated *in vacuo*. The crude product was redissolved in MeCN, precipitated by the addition of diethyl either, filtered, and washed with ether. The precipitate was purified and isolated as a trifluoroacetate salt by RP-HPLC (stationary phase: C18 silica; eluent: 0.2% TFA/$H_2O$ – MeCN gradient). The product is 9.9 mg (2%) purplish solid.

$^1H$ NMR (600 MHz, DMSO-$d_6$) δ (ppm): 8.54 (s, 2H, *3, 5*), 8.54-8.52 (m, 2H, *28, 43*), 8.36-8.33 (m, 4H, *8, 18, 37, 40*), 7.29-7.26 (m, 4H, *9, 19, 38, 41*), 7.27-7.24 (m, 2H, *29, 44*), 4.49-2.97 (brm, 24H, *12, 13, 15, 16, 22, 23, 25, 26, 32, 33, 35, 36*), 2.89 (s, 9H, *39, 42, 45*).

$^{13}C\{^1H\}$ NMR (151 MHz, DMSO-$d_6$) δ (ppm): 166.2 (*2, 6*), 159.0 (*4*), 153.8 (*30*), 153.1 (*10, 20*), 132.0 (*28, 43*), 129.8 (*8, 18, 37, 40*), 121.4 (*27*), 118.8 (*7, 17*), 114.6 (*9, 19, 38, 41*), 114.3 (*29, 44*), 108.1 (*3, 5*), 51.92 (*33, 35*), 51.90 (*13, 15, 23, 25*), 43.8 (*12, 16, 22, 26*), 43.6 (*32, 36*), 42.1 (*39, 42, 45*).

HRMS (ESI positive mode): m/z calculated for $C_{38}H_{47}N_6O^+$ $[M]^+$: 603.3806, found: 603.3799.

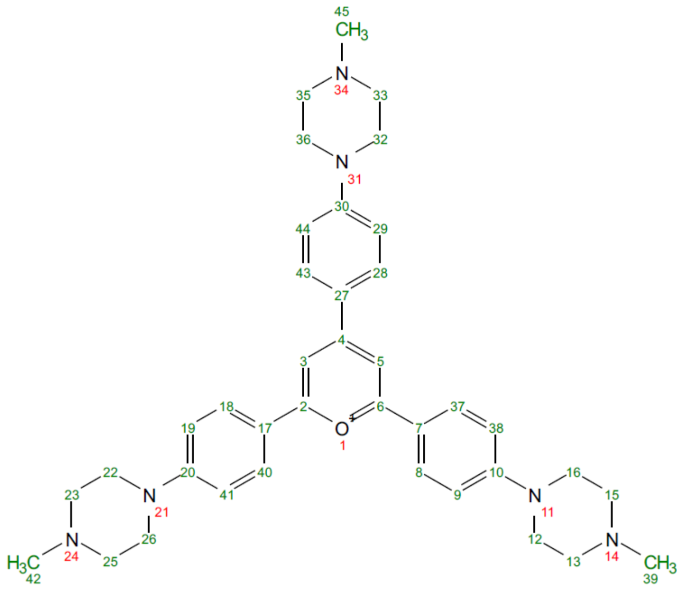

**2,6-NMP-4-Morph - 4,4'-((4-(4-morpholinophenyl)pyrylium-2,6-diyl)bis(4,1-phenylene))bis(1-methylpiperazin-1-ium) tris(trifluoroacetate)**

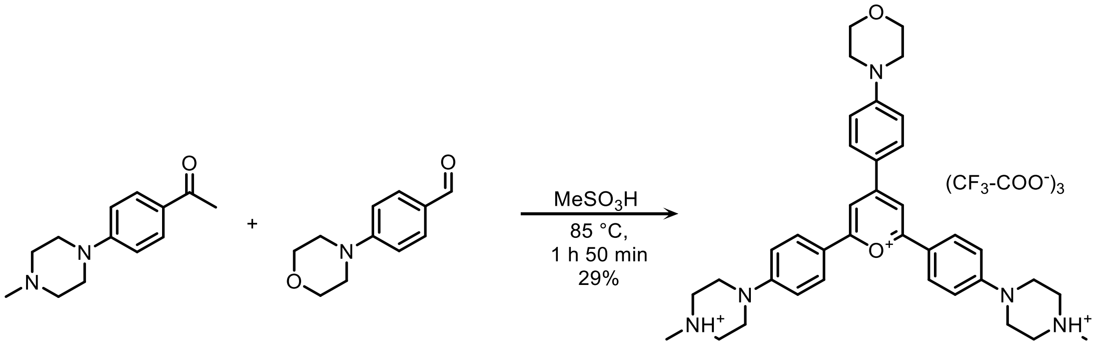


4-Morpholinobenzaldehyde (67.5 mg, 0.353 mmol, 1 eq.) and 1-(4-(4-methylpiperazin-1-yl)phenyl)ethan-1-one (154 mg, 0.706 mmol, 2 eq.) were dissolved in 1 mL methanesulfonic acid and stirred at 85 °C for 1 h 50 min. The solution was precipitated with diethyl ether. Purification by RP-HPLC (stationary phase: C18 silica; eluent: 0.2% TFA/$H_2O$ – MeCN gradient) yielded 96.7 mg (29%) product.

$^1$H NMR (500 MHz, DMSO-$d_6$) δ (ppm): 8.50 (d, J = 8.8 Hz, 2H, *10, 14*), 8.48 (s, 2H, *1, 5*), 8.33 (d, J = 8.7 Hz, 4H, *15, 19, 20, 24*), 7.26 (d, J = 9.0 Hz, 4H, *16, 18, 21, 23*), 7.17 (d, J = 8.9 Hz, 2H, *11, 13*), 4.26 (s, 4H, *28, 32, 38, 42*), 3.78 (t, J = 4.9 Hz, 4H), 3.59 (brs, 4H, *29, 31, 39, 41*), 3.57 (t, J = 5.0 Hz, 4H, *33, 37*), 3.27 (brs, 4H, *28, 32, 38, 42*), 3.18 (brs, 4H, *29, 31, 39, 41*), 2.90 (s, 6H, *43, 44*).

$^{13}$C{$^1$H} NMR (126 MHz, DMSO-$d_6$) δ (ppm): 165.7 (*2, 4*), 158.6 (*6*), 155.0 (*12*), 153.0 (*17, 22*), 132.1 (*10, 14*), 129.6 (*15, 19, 20, 24*), 120.3 (*9*), 119.0 (*7, 8*), 114.6 (*16, 18, 21, 23*), 113.5 (*11, 13*), 107.6 (*1, 5*), 65.8 (*34, 36*), 51.9 (*29, 31, 39, 41*), 46.3 (*33, 37*), 43.7 (*28, 32, 38, 42*), 42.1 (*43, 44*).

HRMS (ESI positive mode): m/z calculated for $C_{28}H_{35}N_4O^+$ $[M]^+$: 590.3490, found: 590.3490.

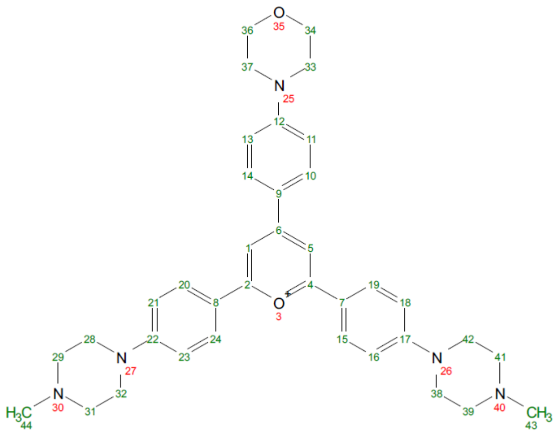

**2,6-Ind-4-DMA - 4-(4-(dimethylamino)phenyl)-2,6-bis(1-(2-(dimethylammonio)ethyl)indolin-5-yl)pyrylium tris(trifluoroacetate)**

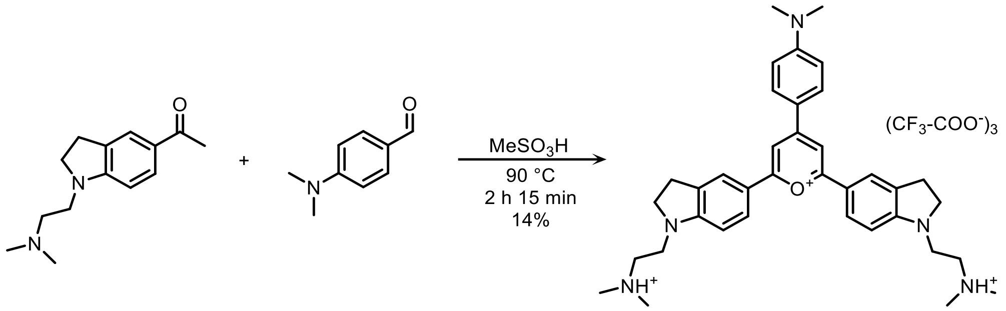


1-(1-(2-(Dimethylamino)ethyl)indolin-5-yl)ethan-1-one (100.5 mg, 0.433 mmol, 2 eq.) and 4-diemthylaminobenzaldehyde (32.8 mg, 0.220 mmol, 1.02 eq.) were dissolved in in 1 mL methanesulfonic acid and stirred at 90 °C for 2 h 15 min. The solution was precipitated with diethyl ether. Purification by RP-HPLC (stationary phase: C18 silica; eluent: 0.2% TFA/H2O – MeCN gradient) yielded 26.9 mg (14%) purple solid product.

$^{1}$H NMR (400 MHz, DMSO-*d*$_6$) δ (ppm): 10.16 (s, 2H, *38, 41 N*$^+$*-H*), 8.36 (d, J = 8.9 Hz, 2H, *10, 14*), 8.20 (s, 2H, *1, 5*), 8.13 (d, J = 8.5 Hz, 2H, *15, 24*), 8.03 (s, 2H, *19, 20*), 6.91 (d, J = 8.9 Hz, 2H, *11, 13*), 6.86 (d, J = 8.6 Hz, 2H, *16, 23*), 3.71 (t, J = 7.6 Hz, 8H, *29, 32, 34, 36*), 3.39 (t, J = 6.7 Hz, 4H, *35, 37*), 3.18 (s, 6H, *26, 27*), 3.23 – 3.11 (m, 4H, *28, 33*), 2.88 (s, 12H, *39, 40, 42, 43*).

$^{13}$C{$^{1}$H} NMR (101 MHz, DMSO-*d*$_6$) δ (ppm): 165.2 (*2, 4*), 157.1 (*6*), 155.9 (*17, 22*), 154.3 (*12*), 131.1 (*18, 21*), 129.3 (*15, 24*), 123.2 (*19, 20*), 118.6 (*9*), 117.7 (*7, 8*), 112.0 (*11, 13*), 106.1 (*16, 23*), 105.6 (*1, 5*), 53.2 (*35, 37*), 51.7 (*29, 32*), 42.6 (*39, 40, 42, 43*), 42.0 (*34, 36*), 39.5 (*26, 27*), 26.8 (*28, 33*).

HRMS (ESI positive mode): m/z calculated for $C_{37}H_{46}N_5O^+$ [M]$^+$: 576.3697, found: 576.3703.

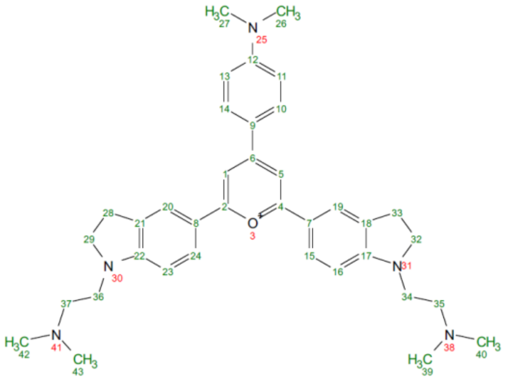

**2,6-NdMP-4-DMA - 4,4'-((4-(4-(dimethylamino)phenyl)pyrylium-2,6-diyl)bis(4,1-phenylene))bis(1,1-dimethylpiperazin-1-ium) tris(trifluoroacetate)**

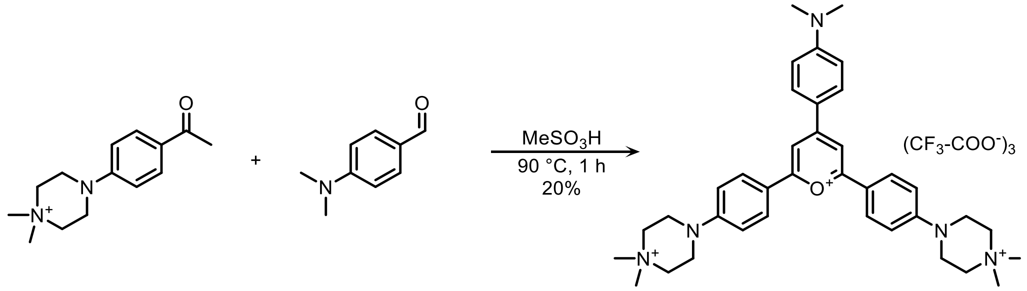


4-(4-Acetylphenyl)-1,1-dimethylpiperazin-1-ium iodide (150 mg, 0.416 mmol, 2 eq.) and 4-dimethylaminobenzaldehyde (37 mg, 0.25 mmol, 1.2 eq.) were dissolved in 1 mL methanesulfonic acid and stirred at 90 °C for 2 h 15 min. The crude product was precipitated with diethyl ether. The ether phase was then discarded and the viscous precipitate was washed with ether two times. Purification by RP-HPLC (stationary phase: C18 silica; eluent: 0.2% TFA/$H_2O$ – MeCN gradient) yielded 37.2 mg (20%) purplish black solid product.

$^{1}$H NMR (400 MHz, DMSO-$d_6$) δ (ppm): 8.50 (d, J=9.2 Hz, 2H, *20, 24*), 8.44 (s, 2H, *1, 5*), 8.34 (d, J=9.0 Hz, 4H, *10, 14, 15, 19*), 7.27 (d, J=9.0 Hz, 4H, *11, 13, 16, 18*), 6.95 (d, J=9.0 Hz, 2H, *21, 23*), 3.85 (broad t, J≈4.7 Hz, 8H, *27, 31, 32, 36*), 3.61 (broad t, J≈4.7 Hz, 8H, *28, 30, 33, 35*), 3.25 (s, 12H, *40, 41, 42, 43*), 3.21 (s, 6H, *38, 39*).

$^{13}$C{$^{1}$H} NMR (101 MHz, DMSO-$d_6$) δ (ppm): 165.0 (*2, 4*), 158.1 (*6*), 157.7 (t, J = 30.5 Hz, *trifluoroacetate carboxyl*), 154.9 (*22*), 152.9 (*12, 17*), 132.4 (*20, 24*), 129.4 (*10, 14, 15, 19*), 119.2 (*7, 8*), 118.4 (*9*), 114.5 (*11, 13, 16, 18*), 112.4 (*21, 23*), 106.9 (*1, 5*), 59.8 (*28, 30, 33, 35*), 50.3 (*40, 41, 42, 43*), 40.5 (*28, 30, 32, 36*).

HRMS (ESI positive mode): m/z calculated for $C_{37}H_{48}N_5O^{3+}$ $[M]^+$: 192.7947, found: 192.7942.

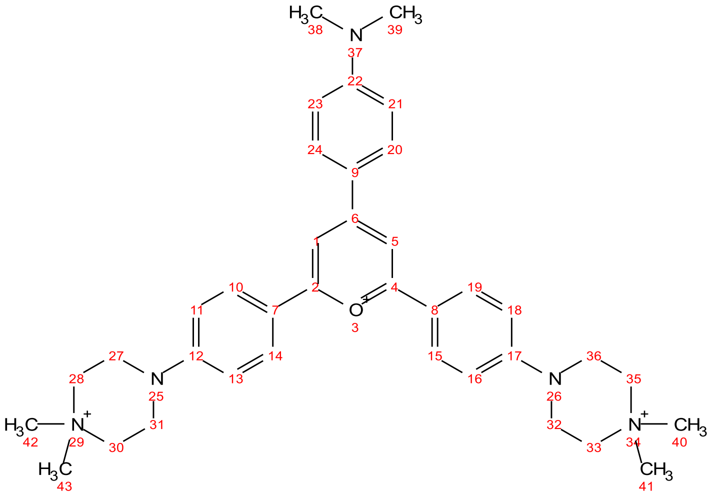

**2,6-NMP-4-OMe - 4,4'-((4-(4-methoxyphenyl)pyrylium-2,6-diyl)bis(4,1-phenylene))bis(1-methylpiperazin-1-ium) tris(trifluoroacetate)**

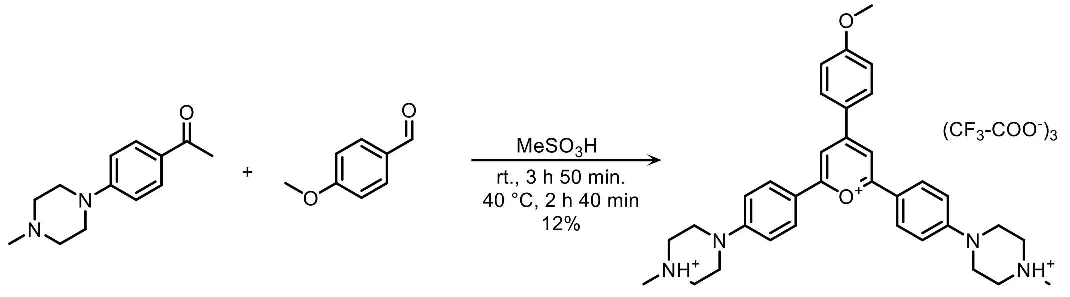


4-Methoxybenzaldehyde (250 mg, 1.82 mmol, 1 eq.) and 1-(4-(4-methylpiperazin-1-yl)phenyl)ethan-1-one (800 mg, 3.66 mmol, 2 eq.) were dissolved in 2.5 mL methanesulfonic acid and stirred at 90 °C for 3 h 50 min. The solution was diluted with distilled water, neutralized with the addition of $NaHCO_3$, extracted with DCM, and concentrated. Purification by RP-HPLC (stationary phase: C18 silica; eluent: 0.2% $TFA/H_2O$ – MeCN gradient) yielded 199.4 mg (12%) product.

$^1$H NMR (400 MHz, DMSO-$d_6$) δ (ppm): 1H NMR (400 MHz, dmso) δ 10.48 (br, 2H, *30, 35 $N^+$-H*), 8.61 (s, 2H, *1, 5*), 8.55 (d, J = 9.0 Hz, 2H, *10, 14*), 8.38 (d, J = 8.9 Hz, 4H, *15, 19, 20, 24*), 7.28 (d, J = 9.0 Hz, 4H, *16, 18, 21, 23*), 7.26 (d, J = 8.9 Hz, 2H, *11, 13*), 4.30 (br, 4H, *28, 32, 33, 37*), 3.96 (s, 3H, *40*), 3.59 (br, 4H, *29, 31, 34, 36*), 3.29 (br, 4H, *28, 32, 33, 37*), 3.19 (br, 4H, *29, 31, 34, 36*), 2.90 (s, 6H, *38, 39*).

$^{13}$C{$^1$H} NMR (101 MHz, DMSO-$d_6$) δ (ppm): 167.3 (*2, 4*), 164.6 (*12*), 160.1 (*6*), 153.4 (*17, 22*), 131.9 (*10, 14*), 130.2 (*15, 19, 20, 24*), 125.0 (*9*), 118.5 (*7, 8*), 115.2 (*11, 13*), 114.5 (*16, 18, 21, 23*), 109.5 (*1, 5*), 56.0 (*40*), 51.9 (*29, 31, 34, 36*), 43.7 (*28, 32, 33, 37*), 42.1 (*38, 39*).

HRMS (ESI positive mode): m/z calculated for $C_{34}H_{39}N_4O_2^+$ $[M]^+$: 535.3068, found: 535.3073.

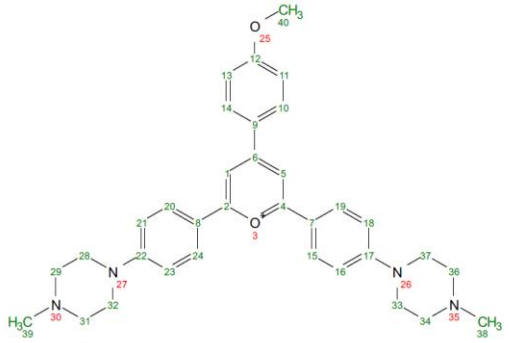

**2,6-NMP-4-OEt - 4,4'-((4-(4-ethoxyphenyl)pyrylium-2,6-diyl)bis(4,1-phenylene))bis(1-methylpiperazin-1-ium) tris(trifluoroacetate)**

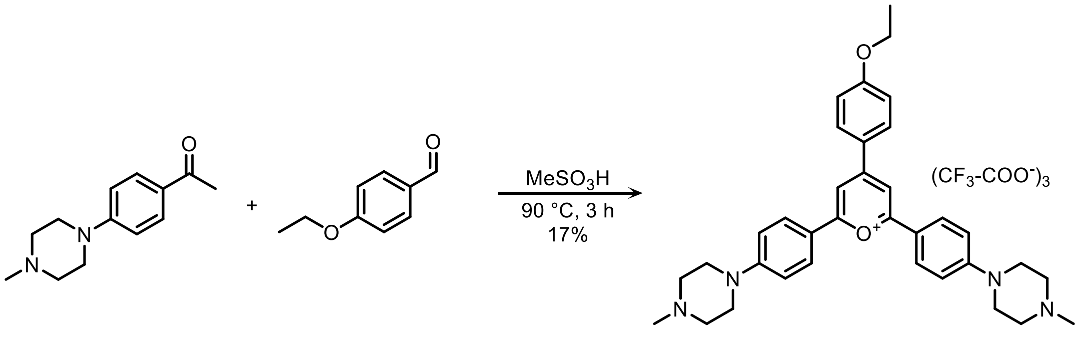

4-Ethoxybenzaldehyde (47.8 µL, 51.6 mg, 0.344 mmol, 1 eq.) and 1-(4-(4-methylpiperazin-1-yl)phenyl)ethan-1-one (150 mg, 0.687 mmol, 2 eq.) were dissolved in 1 mL methanesulfonic acid and stirred at 90 °C for 3 h. The solution was precipitated with diethyl ether. Purification by RP-HPLC (stationary phase: C18 silica; eluent: 0.2% TFA/$H_2O$ – MeCN gradient) yielded 51.4 mg (17%) product.

$^1$H NMR (600 MHz, DMSO-$d_6$) δ (ppm): 10.50 (brs, 2H), 8.60 (s, 2H, *1, 5*), 8.54 (d, J = 9.0 Hz, 2H, *10, 14*), 8.37 (d, J = 8.9 Hz, 4H, *15, 19, 20, 24*), 7.28 (d, J = 9.3 Hz, 4H, *16, 18, 21, 23*), 7.24 (d, J = 8.9 Hz, 2H, *11, 13*), 4.30 (brs, 4H, *27, 31, 32, 36*), 4.25 (q, J = 7.0 Hz, 2H, *40*), 3.59 (brs, 4H, *28, 30, 33, 35*), 3.30 (brs, 4H, *27, 31, 32, 36*), 3.18 (brs, 4H, *28, 30, 33, 35*), 2.90 (s, 6H, *37, 38*), 1.41 (t, J = 7.0 Hz, 3H, *41*).

$^{13}$C{$^1$H} NMR (151 MHz, DMSO-$d_6$) δ (ppm): 167.3 (*2, 4*), 164.0 (*12*), 160.1 (*6*), 153.3 (*17, 22*), 131.9 (*10, 14*), 130.2 (*15, 19, 20, 24*), 124.9 (*9*), 118.5 (*7, 8*), 115.5 (*11, 13*), 114.5 (*16, 18, 21, 23*), 109.5 (*1, 5*), 64.1 (*40*), 51.9 (*28, 30, 33, 35*), 43.7 (*27, 31, 32, 36*), 42.1 (*37, 38*), 14.4 (*41*).

HRMS (ESI positive mode): m/z calculated for $C_{35}H_{41}N_4O_2^+$ [M]$^+$: 549.3225, found: 549.3228.

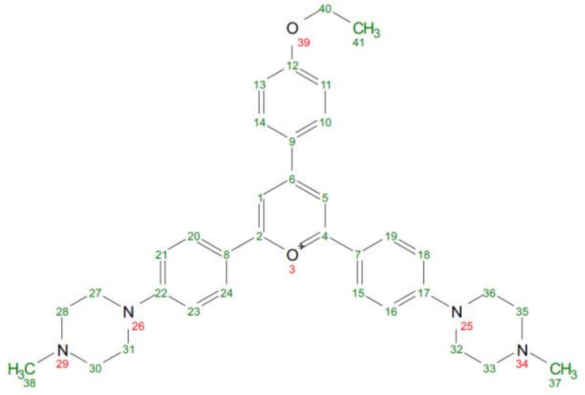

**2,6-NMP-4-OH - 4-(4-hydroxyphenyl)-2,6-bis(4-(4-methylpiperazin-1-yl)phenyl)pyrylium tris(trifluoroborate)**

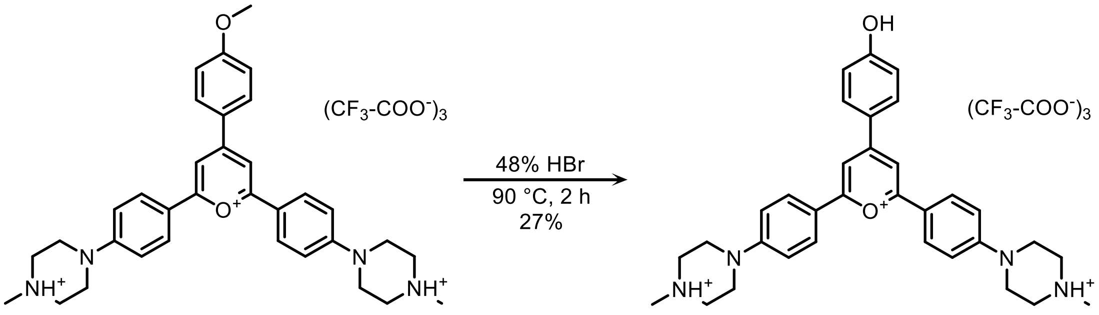


**2,6-NMP-4-OMe** (51.3 mg, 0.0585 mmol, 1 eq.) was dissolved in 2.5 mL 48% HBr solution and stirred at 100 °C for 18.5h. The solution was diluted with distilled water; DCM was added and the aqueous phase was neutralized with the careful addition of cc. $NaHCO_3$ until the product could be extracted into the organic phase. The phases were separated and the aqueous phase was extracted with further DCM. The collected organic phases were washed with cc. NaCl, dried over $MgSO_4$, and concentrated in vacuo.

Purification by RP-HPLC (stationary phase: C18 silica; eluent: 0.2% TFA/$H_2O$ – MeCN gradient) yielded 13.5 mg (27%) product.

$^1$H NMR (600 MHz, DMSO-$d_6$) δ (ppm): 11.19 (1H, br), 10.36 (2H, br), 8.55 (s, 2H, *1, 5*), 8.50 – 8.41 (m, Hz, 2H, *10, 14*), 8.41 – 8.31 (m, 4H, *15, 19, 20, 24*), 7.36 – 7.18 (m, 4H, *16, 18, 21, 23*), 7.12 – 6.97 (m, 2H, *11, 13*), 3.85 (br m, 16H, *28, 29, 31, 32, 33, 34, 36, 37*), 2.89 (s, 6H, *38, 39*).

$^{13}C\{^1H\}$ NMR (151 MHz, DMSO-$d_6$) δ (ppm): 166.9 (*2, 4*), 164.2 (*12*), 160.2 (*6*), 153.3 (*17, 22*), 132.3 (*10, 14*), 130.0 (*15, 19, 20, 24*), 123.3 (*9*), 118.5 (*7, 8*), 116.7 (*11, 13*), 114.5 (*16, 18, 21, 23*), 109.0 (*1, 5*), 51.9 (*29, 31, 34, 36*), 43.7 (*28, 32, 33, 37*), 42.1 (*38, 39*).

HRMS (ESI positive mode): m/z calculated for $C_{33}H_{37}N_4O_2^+$ $[M]^+$: 521,2911, found: 521.2909.

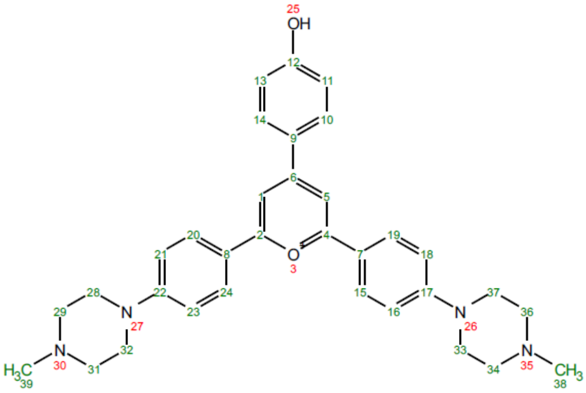

**2,6-NMP-4-2MeOPh - 4,4'-((4-(2-methoxyphenyl)pyrylium-2,6-diyl)bis(4,1-phenylene))bis(1-methylpiperazin-1-ium) tris(trifluoroacetate)**

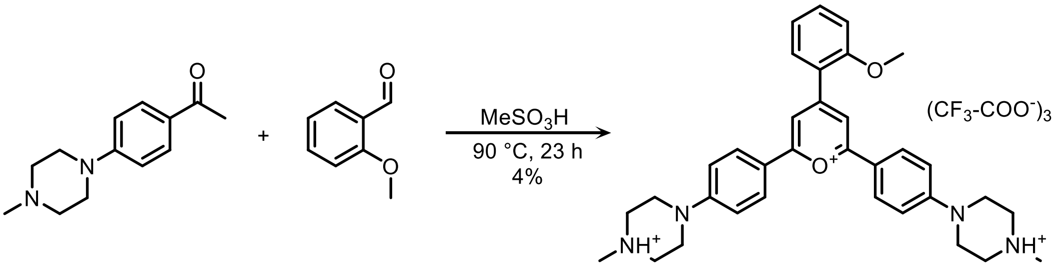


1-(4-(4-Methylpiperazin-1-yl)phenyl)ethan-1-one (150 mg, 0.687 mmol, 2 eq.) and 2-methoxybenzaldehyde (46.8 mg, 0.344 mmol, 1 eq.) were dissolved in 1 mL methanesulfonic acid and stirred at 90 °C for 23 h. The solution was precipitated with diethyl ether. Purification by RP-HPLC (stationary phase: C18 silica; eluent: 0.2% TFA/$H_2O$ – MeCN gradient) yielded 13.1 mg (4.3%) product.

$^1H$ NMR (500 MHz, DMSO-$d_6$) δ (ppm): 8.50 (s, 2H, *1, 5*), 8.29 (d, J = 8.8 Hz, 4H, *15, 19, 20, 24*), 7.91 (dd, J = 7.8, 1.7 Hz, 1H, *14*), 7.73 – 7.67 (m, 1H, *19*), 7.35 (d, J = 8.5 Hz, 1H, *11*), 7.27 (d, J = 9.0 Hz, 4H, *16, 18, 21, 23*), 7.25 (t, J = 7.4 Hz, 1H, *13*), 3.95 (s, 3H, *38*), 3.38 (brs, 32H, *27, 28, 30, 31, 32, 33, 35, 36*), 2.89 (s, 6H, *39, 40*).

$^{13}C\{^1H\}$ NMR (126 MHz, DMSO-$d_6$) δ (ppm): 167.3 (*2, 4*), 160.9 (*6*), 158.0 (*10*), 153.4 (*17, 22*), 134.6 (*12*), 131.3 (*14*), 130.2 (*15, 19, 20, 24*), 123.4 (*9*), 121.2 (*13*), 118.2 (*7, 8*), 114.6 (*16, 18, 21, 23*), 114.1 (*1, 5*), 112.6 (*11*), 56.2 (*38*), 51.8 (*28, 30, 33, 35*), 43.6 (*27, 31, 32, 36*), 42.1 (*39, 40*).

HRMS (ESI positive mode): m/z calculated for $C_{34}H_{39}N_4O_2^+$ $[M]^+$: 535.3068, found: 535.3079.

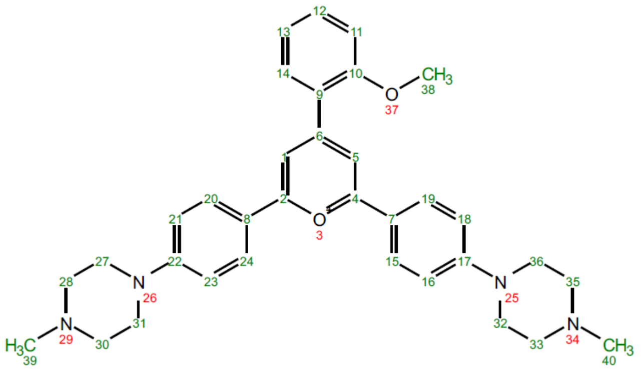

**2,6-Pip-4-2MeOPh - 4-(2-methoxyphenyl)-2,6-bis(4-(piperidin-1-yl)phenyl)pyrylium trifluoroacetate**

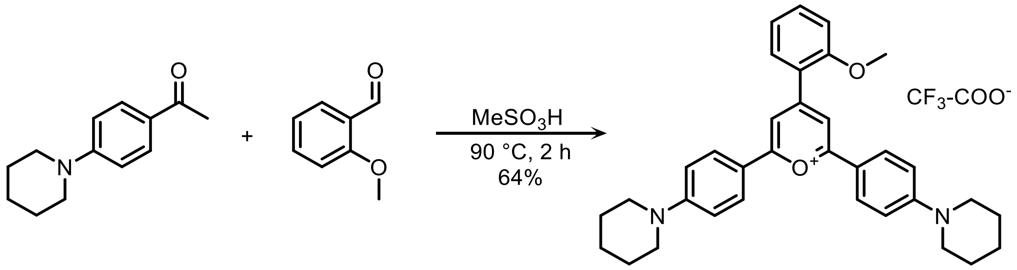


1-(4-(4-Methylpiperazin-1-yl)phenyl)ethan-1-one (80 mg, 0.394 mmol, 2 eq.) and 2-methoxybenzaldehyde (34.8 mg, 0.256 mmol, 1.3 eq.) were dissolved in 1 mL methanesulfonic acid and stirred at 90 °C for 2 h. The solution was poured on ice, extracted with DCM, washed with $NaHCO_3$ and cc. NaCl, dried over $MgSO_4$, and concentrated *in vacuo*. The product is 63.9 mg greenish black powder, 64%.

The product can be further purified and isolated as a trifluoroacetate salt by RP-HPLC (stationary phase: C18 silica; eluent: 0.2% TFA/$H_2O$ – MeCN gradient).

$^1$H NMR (500 MHz, DMSO-$d_6$) δ (ppm): 7.98 (d, J = 8.8 Hz, 4H, *15, 19, 20, 24*), 7.86 (s, 2H, *1, 5*), 7.69 (d, J = 7.6 Hz, 1H, *14*), 7.58 (t, J = 7.8 Hz, 1H, *12*), 7.19 (t, J = 7.5 Hz, 1H, *13*), 7.10 (d, J = 8.4 Hz, 1H, *11*), 7.00 (d, J = 8.8 Hz, 4H, *16, 18, 21, 23*), 3.97 (s, 3H, *38*), 3.59 – 3.43 (m, 8H, *27, 31, 32, 36*), 1.73 (brs, 12H, *28, 29, 30, 33, 34, 35*).

$^{13}$C{$^1$H} NMR (126 MHz, DMSO-$d_6$) δ (ppm): 167.3 (*2, 4*), 159.5 (*6*), 158.1 (*10*), 154.9 (*17, 22*), 134.6 (*12*), 130.7 (*14*), 130.2 (*15, 19, 20, 24*), 123.9 (*9*), 122.1 (*13*), 115.9 (*7, 8*), 114.1 (*16, 18, 21, 23*), 112.4 (*11*), 112.2 (*1, 5*), 56.6 (*38*), 48.4 (*27, 31, 32, 36*), 25.6 (*28, 30, 33, 35*), 24.4 (*29*).

HRMS (ESI positive mode): m/z calculated for $C_{34}H_{37}N_2O_2^+$ [M]$^+$: 505.2850, found: 505.2853

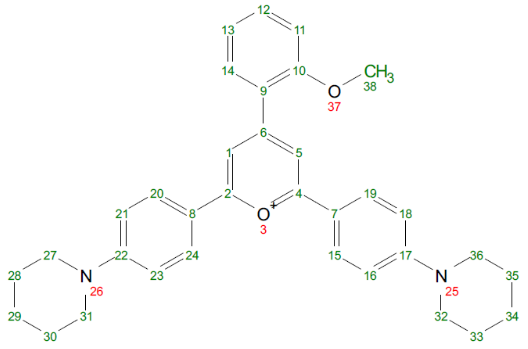

**2,4-NMP-6-Ph - 4,4'-((6-phenylpyrylium-2,4-diyl)bis(4,1-phenylene))bis(1-methylpiperazin-1-ium)**

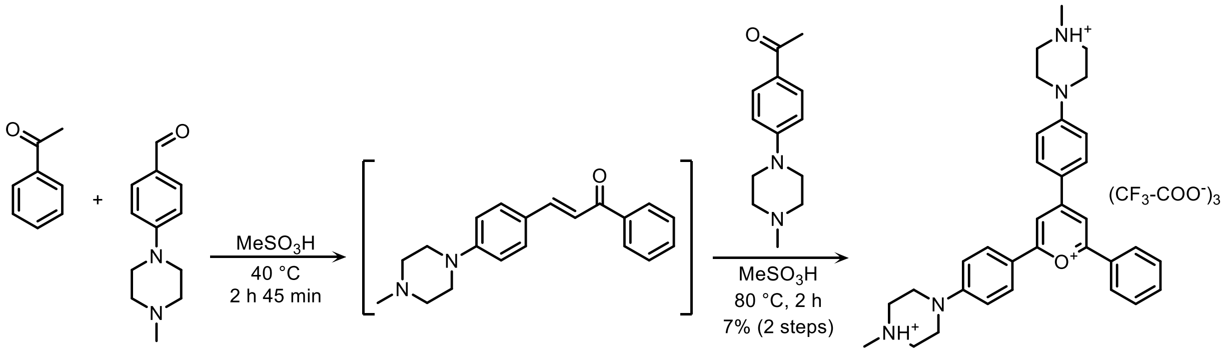


Acetophenone (80.2 µL, 82,6 mg, 0,687 mmol, 1 eq.) and 4-(4-methylpiperazin-1-yl)benzaldehyde (140 mg 0,687 mmol, 1 eq.) were dissolved in 1.5 mL methanesulfonic acid and stirred at 40 °C for 2 h 45 min. 1-(4-(4-Methylpiperazin-1-yl)phenyl)ethan-1-one (150 mg, 0.687 mmol, 1 eq.) was added and the solution was stirred at 80 °C for 2 h. The solution was precipitated with diethyl ether. Purification by RP-HPLC (stationary phase: C18 silica; eluent: 0.2% TFA/$H_2O$ – MeCN gradient) yielded 40.2 mg (7%) product.

$^1$H NMR (500 MHz, DMSO-$d_6$) δ (ppm): 10.56 (s, 1H, *28 or 35 $N^+$-H*), 10.15 (s, 1H, *28 or 35 $N^+$-H*), 8.71 (d, J = 1.7 Hz, 1H, *5*), 8.68 (d, J = 1.7 Hz, 1H, *1*), 8.59 (d, J = 8.9 Hz, 2H, *10, 14*), 8.44 – 8.42 (m, 2H, *20, 24*), 8.42 – 8.40 (m, 2H, *15, 19*), 7.80 – 7.76 (m, 1H, *22*), 7.75 – 7.69 (m, 2H, *21, 23*), 7.28 (d, J = 9.0 Hz, 2H, *16, 18*), 7.27 (d, J = 9.2 Hz, 2H, *11, 13*), 4.39 (brs, 2H, *33, 37*), 4.30 (brs, 2H, *26, 30*), 3.60 (brs, 4H, *27, 29, 34, 36*), 3.34 (brs, 4H, *26, 30, 33, 37*), 3.19 (brs, 4H, *27, 29, 34, 36*), 2.90 (s, 6H, *31, 38*).

$^{13}$C{$^1$H} NMR (126 MHz, DMSO-$d_6$) δ (ppm): 167.7 (*4*), 165.3 (*2*), 159.7 (*6*), 154.2 (*12*), 153.4 (*17*), 133.5 (*22*), 132.5 (10, 14), 130.4 (*15, 19*), 129.8 (*8*), 129.5 (*21, 23*), 127.6 (*20, 24*), 121.0 (*9*), 118.4 (*7*), 114.5 (*16, 18*), 114.3 (*11, 13*), 109.7 (*1*), 109.5 (*5*), 51.9 (*27, 29, 34, 36*), 43.6 (*26, 30*), 43.5 (*33, 37*), 42.1 (*31, 35*).

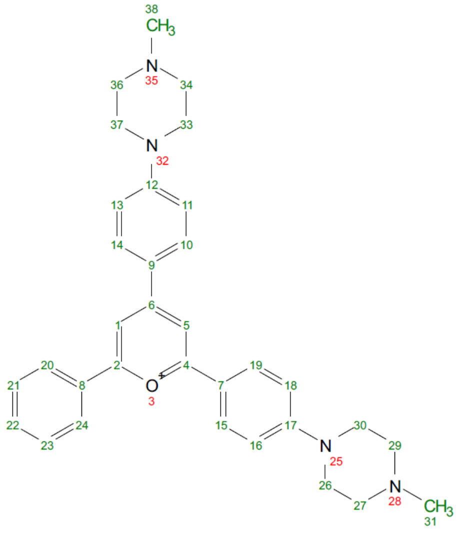

HRMS (ESI positive mode): m/z calculated for $C_{33}H_{37}N_4O^+$ [M]$^+$: 505.2962, found: 535.3073.

**2,4-DMA-6-Me - 2,4-bis(4-(dimethylamino)phenyl)-6-methylpyrylium trifluoroacetate (or perchlorate)**

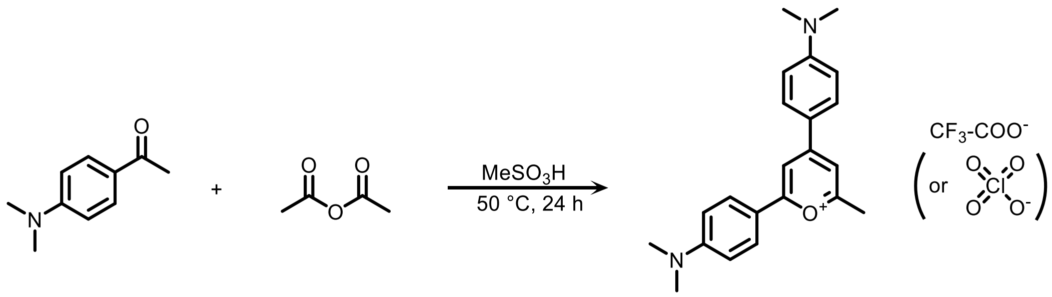


4-Dimethylaminoacetophenone (100 mg, 1.23 mmol, 2 eq.) was dissolved in the mixture of acetic anhydride (100 µL, 108 mg, 1.06 mmol, 1.73 eq.) and 600 µL methanesulfonic acid and stirred at 50 °C for 24 h. The solution was added to 20 mL diethyl ether and stirred. The ether phase was then discarded

and the viscous precipitate was washed with 10 mL ether. The crude product was purified by RP-HPLC (stationary phase: C18 silica; eluent: 0.2% TFA/$H_2O$ – MeCN gradient). 47.3 mg (35%) purplish brown product was obtained.

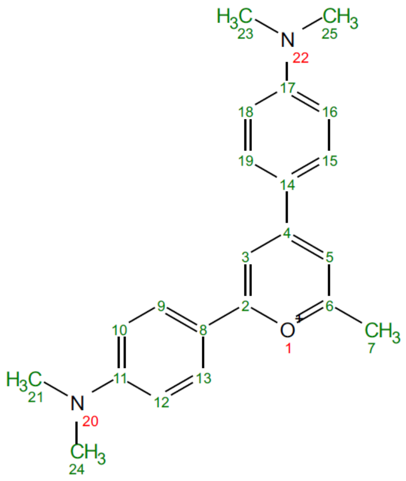

The product can be also isolated as a perchlorate salt for synthetic purposes by pouring the reaction mixture on ice and precipitating the pyrylium salt by the addition of 70% perchloric acid (2 mL for a reaction mixture initially containing 600 mg 4-dimethylaminoacetophenone, with a crude yield of 73%).

$^{1}$H NMR (400 MHz, DMSO-$d_6$) δ (ppm): 8.34 (d, J=1.5 Hz, 1H, *3*), 8.25 (d, J=9.5 Hz, 2H, *15, 19*), 8.16 (d, J=9.2 Hz, 2H, *9, 13*), 7.86 (s, 1H, *5*), 6.91 (d, J=9.5, 2H, *16, 18*), 6.88 (d, J=9.4, 2H, *10, 12*), 3.17 (s, 6H, *23, 25*), 3.12 (s, 6H, *21, 24*), 2.67 (s, 3H, *7*).

$^{13}$C{$^{1}$H} NMR (101 MHz, DMSO-$d_6$) δ (ppm): 168.5 (*6*), 167.7 (*2*), 157.9 (*4*), 154.8 (*17*), 153.9 (*11*), 131.8 (*15, 19*), 129.7 (*9, 13*), 117.7 (*14*), 115.0 (*8*), 112.4 (*16, 18*), 111.9 (*10, 12*), 110.4 (*5*), 106.5 (*3*), 39.8 (*23, 25*), 39.7 (*21, 24*), 20.1 (*7*).

HRMS (ESI positive mode): m/z calculated for $C_{22}H_{25}N_2O^+$ $[M]^+$: 333.1961, found: 333.1952.

**2,4-NMP-6-Me - 4,4'-((6-methylpyrylium-2,4-diyl)bis(4,1-phenylene))bis(1-methylpiperazin-1-ium) tris(trifluoroacetate)**

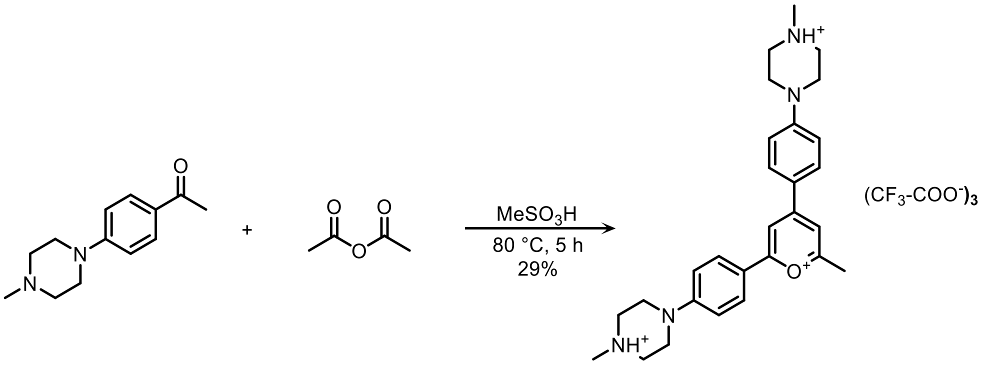


DF-490

1-(4-(4-Methylpiperazin-1-yl)phenyl)ethan-1-one (100 mg, 0.458 mmol, 2 eq.) was dissolved in the mixture of acetic anhydride (100 µL, 108 mg, 1.06 mmol, 4.62 eq.) and 600 µL methanesulfonic acid and stirred at 80 °C for 5 h. The solution was added to 20 mL diethyl ether and stirred. The ether phase was then discarded and the viscous precipitate was washed with 10 mL ether. The crude product was purified by RP-HPLC (stationary phase: C18 silica; eluent: 0.2% TFA/$H_2O$ – MeCN gradient). 52.2 mg (29%) purplish brown product was obtained.

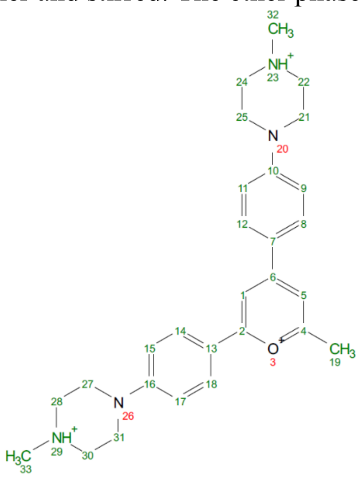

$^{1}$H NMR (400 MHz, DMSO-$d_6$) δ (ppm): 10.52 (s, 2H, *23, 29*), 8.64 (d, J = 1.9 Hz, 1H, *1*), 8.41 – 8.37 (m, 2H, *8, 12*), 8.33 – 8.29 (m, 2H, *14, 18*), 8.15 (d, J = 1.8 Hz, 1H, *5*), 7.27 – 7.24 (m, 2H, *9, 11*), 7.26 – 7.22 (m, 2H, *15, 17*), 4.38 (s, 2H, *27, 31*), 4.29 (s, 2H, *21, 25*), 3.60 – 3.57 (m, 4H, *22, 24, 28, 30*), 3.34 (s, 2H, *27, 31*), 3.28 (s, 2H, *21, 25*), 3.16 (s, 4H, *22, 24, 28, 30*), 2.89 (s, 3H, *32*), 2.88 (s, 3H, *33*), 2.77 (s, 3H, *19*).

$^{13}C\{^1H\}$ NMR (101 MHz, DMSO-*d*$_6$) δ (ppm): 171.1 (*4*), 168.6 (*2*), 159.8 (*6*), 154.2 (*10*), 153.4 (*16*), 132.2 (*8, 12*), 130.1 (*14, 18*), 120.4 (*4*), 118.2 (*13*), 114.4 (*9, 11, 15, 17*), 112.4 (*5*), 108.8 (*1*), 51.9 (22, *24, 28, 30*), 43.6 (*21, 25*), 43.5 (*27, 31*), 42.1 (*32, 33*), 20.4 (*19*).

HRMS (ESI positive mode): m/z calculated for $C_{28}H_{35}N_4O^+$ $[M]^+$: 443.2805, found: 443.2805.

**2,4-DMA-6-BTA - (Z)-2,4-bis(4-(dimethylamino)phenyl)-6-((3-methylbenzo[d]thiazol-2(3H)-ylidene)methyl)pyrylium trifluoroacetate**

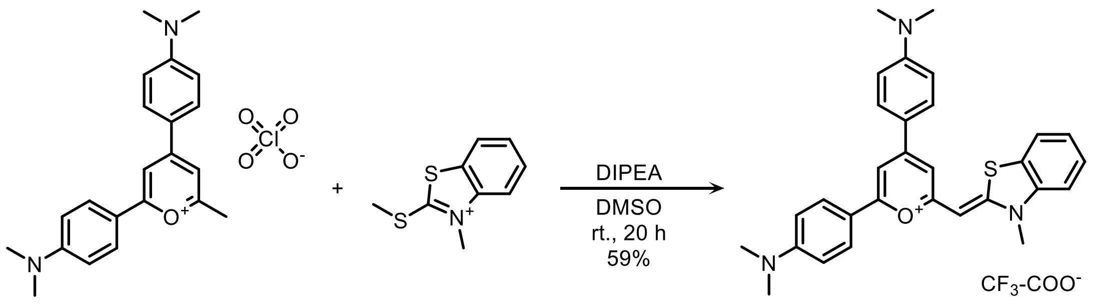


Under $N_2$ 2,4-DMA-6-Me (perchlorate salt, 69.9 mg, 0.161 mmol, 1 eq.) and (59.9 mg, 0.161 mmol, 1 eq.) 3-methyl-2-methylthiobenzothiazolium p-toluenesulfonate was dissolved in 1 mL DMSO. Diisopropylethylamine (0.141 µL, 104 mg, 0.807 mmol, 5 eq.) was added to the solution and the reaction was stirred for 20 h. Purification by RP-HPLC (stationary phase: C18 silica; eluent: 0.2% TFA/$H_2O$ – MeCN gradient) yielded 56.4 mg (59%) product.

$^1H$ NMR (400 MHz, DMSO-*d*$_6$) δ (ppm): 8.03 (d, J = 8.1 Hz, 2H, *15, 19*), 7.99 (d, J = 7.8 Hz, 1H, *35*), 7.79 (d, J = 8.5 Hz, 2H, *10, 14*), 7.61 (d, J = 8.33, 1H, *32*), 7.53 (t, J=7.6 Hz, 1H, *33*), 7.49 (s, 1H, *1*), 7.37 (t, J=7.5 Hz, 1H, *34*), 7.32 (s, 1H, *5*), 6.72 (d, J = 8.6 Hz, 2H, *16, 18*), 6.63 (d, J = 8.6 Hz, 2H, *11, 13*), 6.29 (s, 1H, *7*), 3.68 (s, 3H, *31*), 3.00 (s, 12H, *22, 23, 24, 25*).

$^{13}C\{^1H\}$ NMR (101 MHz, DMSO-*d*$_6$) δ (ppm): 165.3 (*4*), 162.0 (*2*), 157.4 (*26*), 152.8 (*12*), 152.2 (*17*), 150.3 (*6*), 140.3 (*28*), 129.1 (*10, 14*), 128.0 (*15, 19, 33*), 125.0 (*29*), 124.3 (*34*), 123.0 (*35*), 119.0 (*9*), 116.4 (*8*), 112.8 (*32*), 111.7 (*11, 13*), 111.5 (*16, 18*), 109.1 (*5*), 101.8 (*1*), 86.8 (*7*), 39.6 (*22, 23, 24, 25*), 33.3 (*31*),

HRMS (ESI positive mode): m/z calculated for $C_{30}H_{30}N_3OS^+$ $[M]^+$: 480.2105, found: 480.210

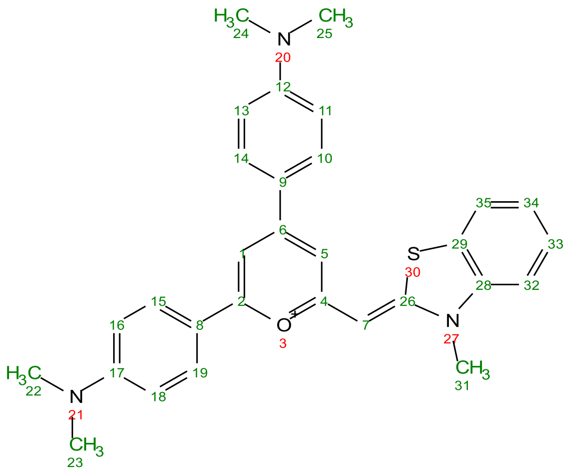

# 3. NMR and HRMS spectra of the prepared compounds

## 3.1. 4-Morpholinobenzaldehyde

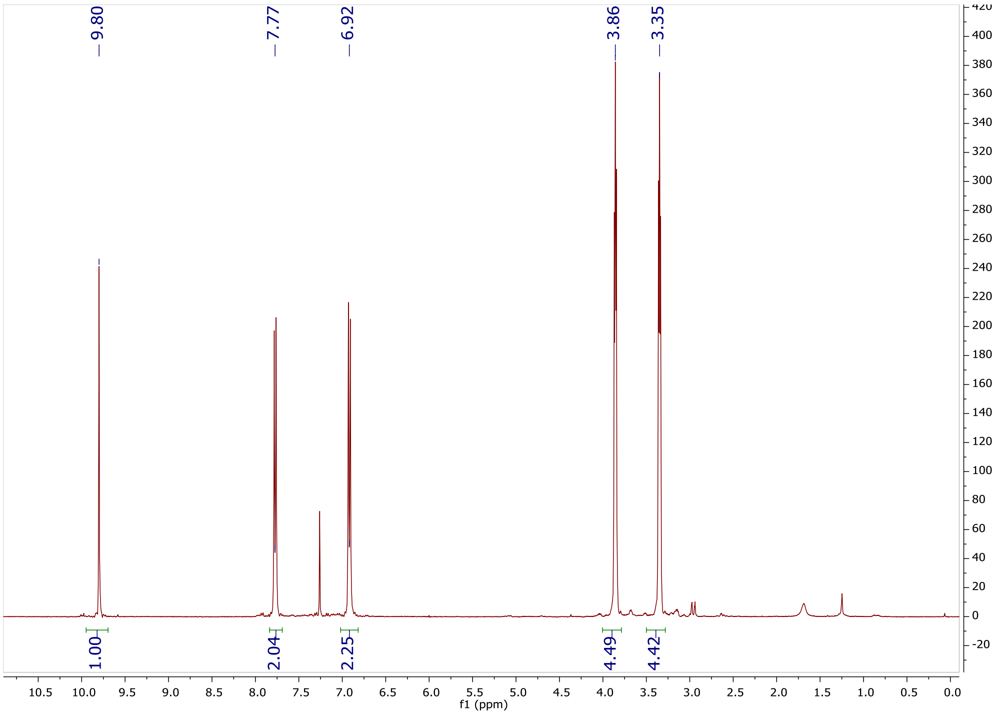


*Figure S3: $^{1}$H-NMR spectrum of 4-morpholinobenzaldehyde, measured at 293 K in $CDCl_3$*

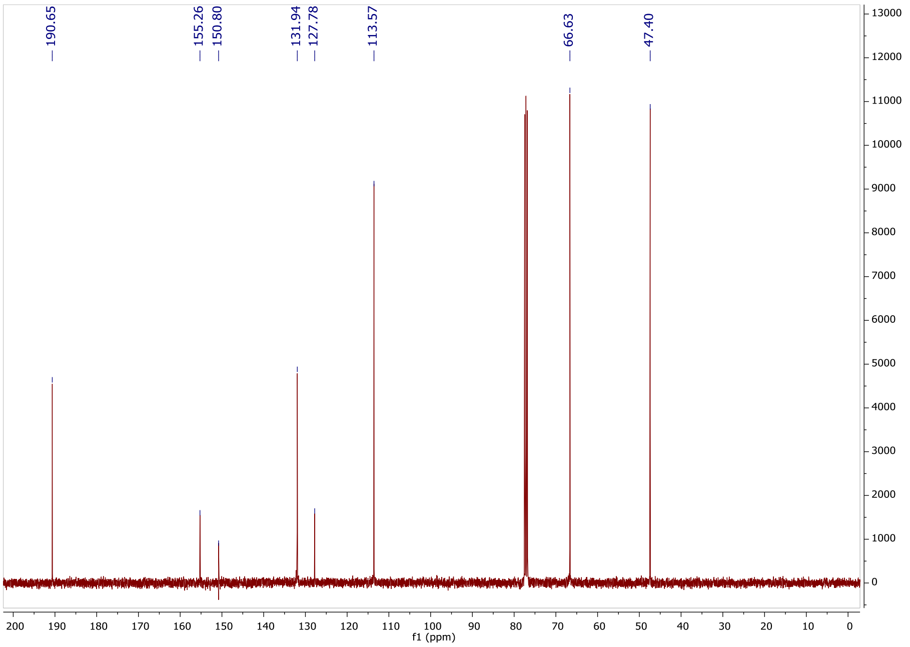


*Figure S4: $^{13}$C-NMR spectrum of 4-morpholinobenzaldehyde, measured at 293 K in $CDCl_3$*

## 3.2. 4-(4-Methylpiperazin-1-yl)benzaldehyde

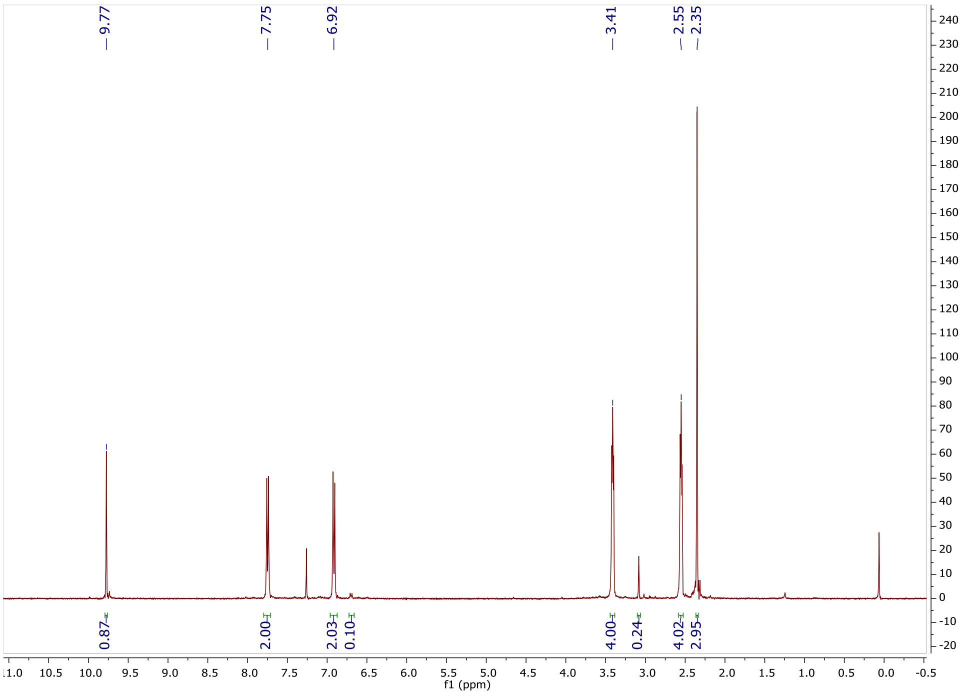


*Figure S5: $^{1}$H-NMR spectrum of 4-(4-methylpiperazin-1-yl)benzaldehyde, measured at 293 K in $CDCl_3$*

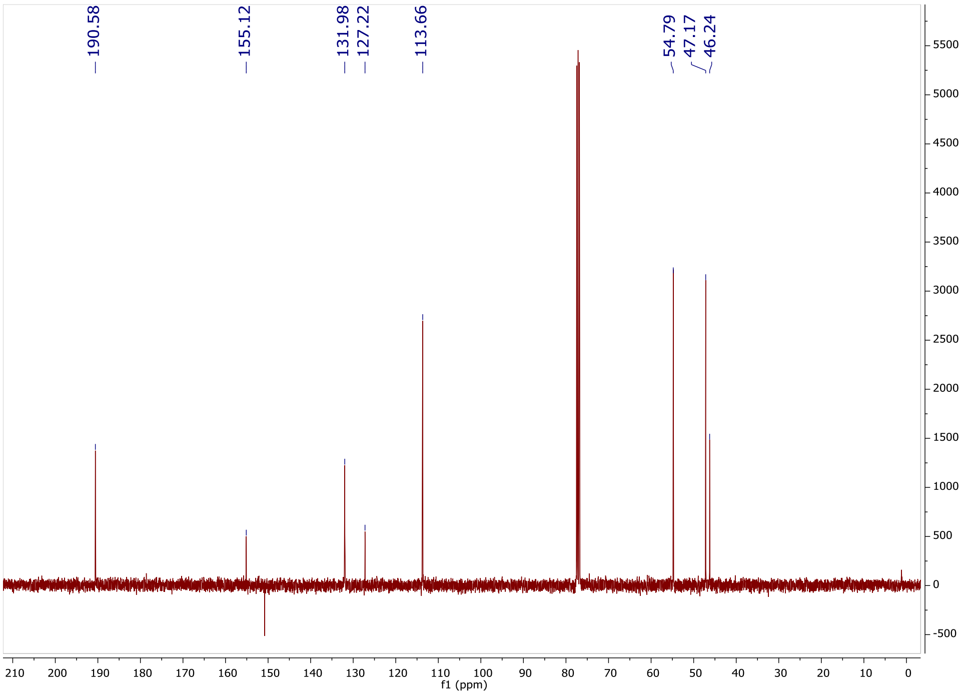


*Figure S6: $^{13}$C-NMR spectrum of 4-(4-methylpiperazin-1-yl)benzaldehyde, measured at 293 K in $CDCl_3$*

### 3.3. 1-(4-(Piperidin-1-yl)phenyl)ethan-1-one

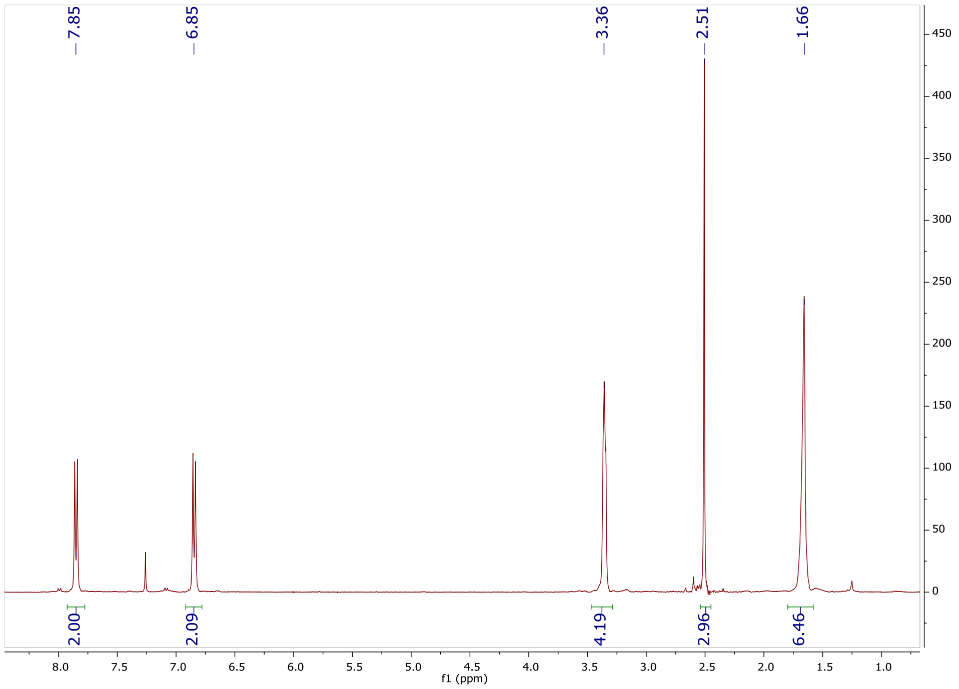


*Figure S7: $^{1}$H-NMR spectrum of 1-(4-(piperidin-1-yl)phenyl)ethan-1-one, measured at 293 K in $CDCl_3$*

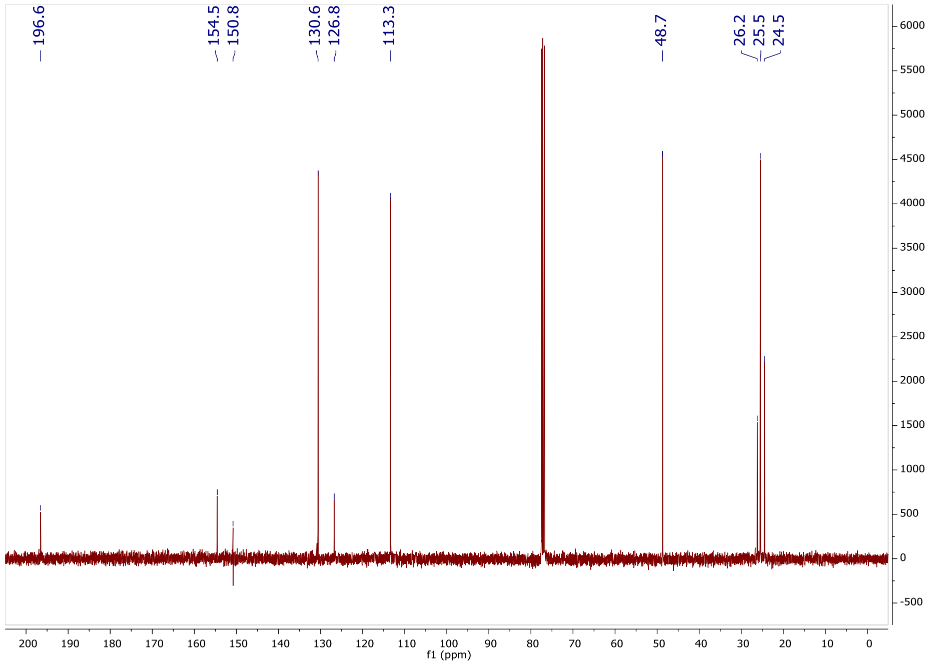


*Figure S8: $^{13}$C-NMR spectrum of 1-(4-(piperidin-1-yl)phenyl)ethan-1-one, measured at 293 K in $CDCl_3$*

## 3.4. 1-(4-(4-Methylpiperazin-1-yl)phenyl)ethan-1-one

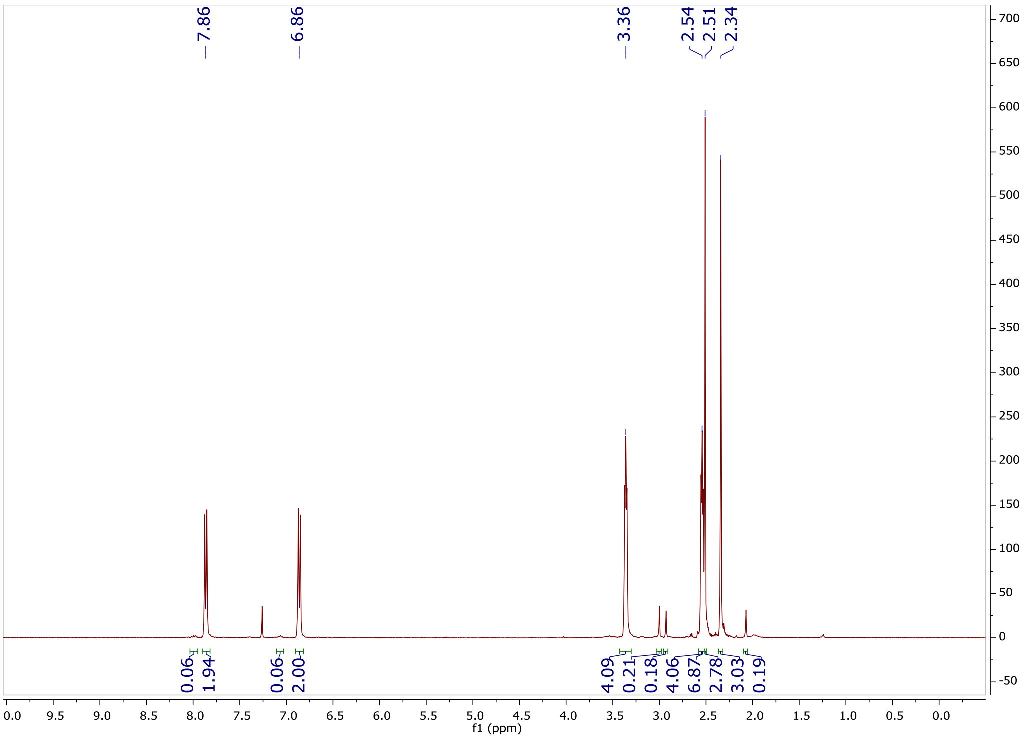


*Figure S9: $^{1}$H-NMR spectrum of 1-(4-(4-methylpiperazin-1-yl)phenyl)ethan-1-one, measured at 293 K in $CDCl_3$*

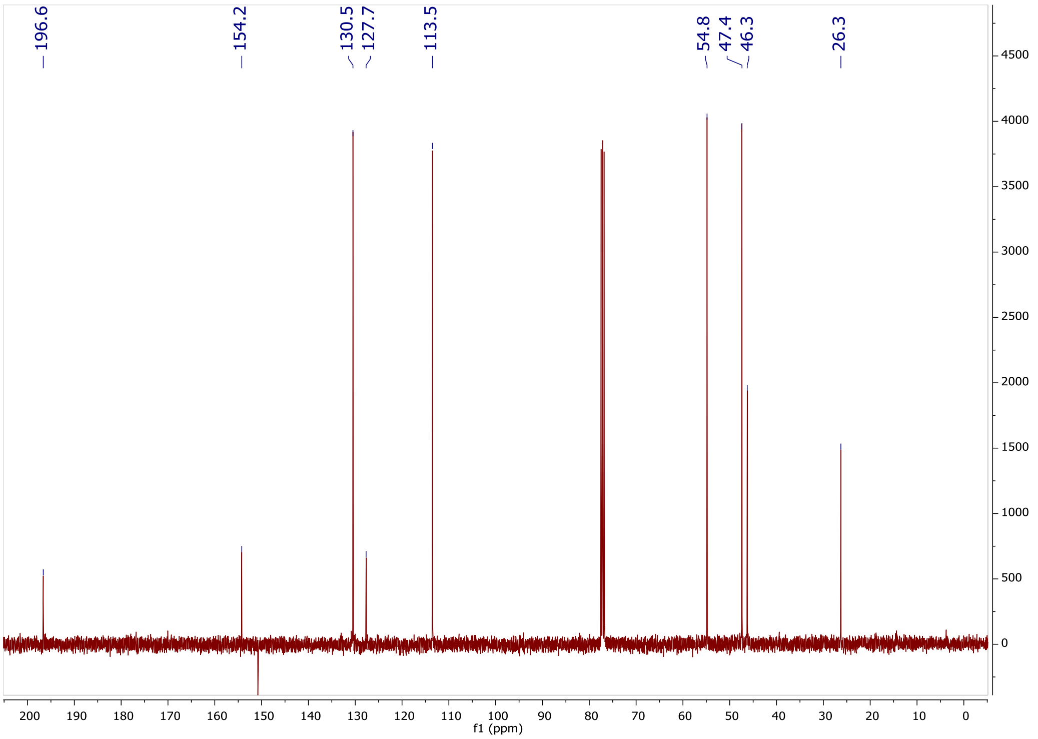


*Figure S10: $^{13}$C-NMR spectrum of 1-(4-(4-methylpiperazin-1-yl)phenyl)ethan-1-one, 400 MHz, measured at 293 K in $CDCl_3$*

## 3.5. 4-(4-Acetylphenyl)-1,1-dimethylpiperazin-1-ium iodide

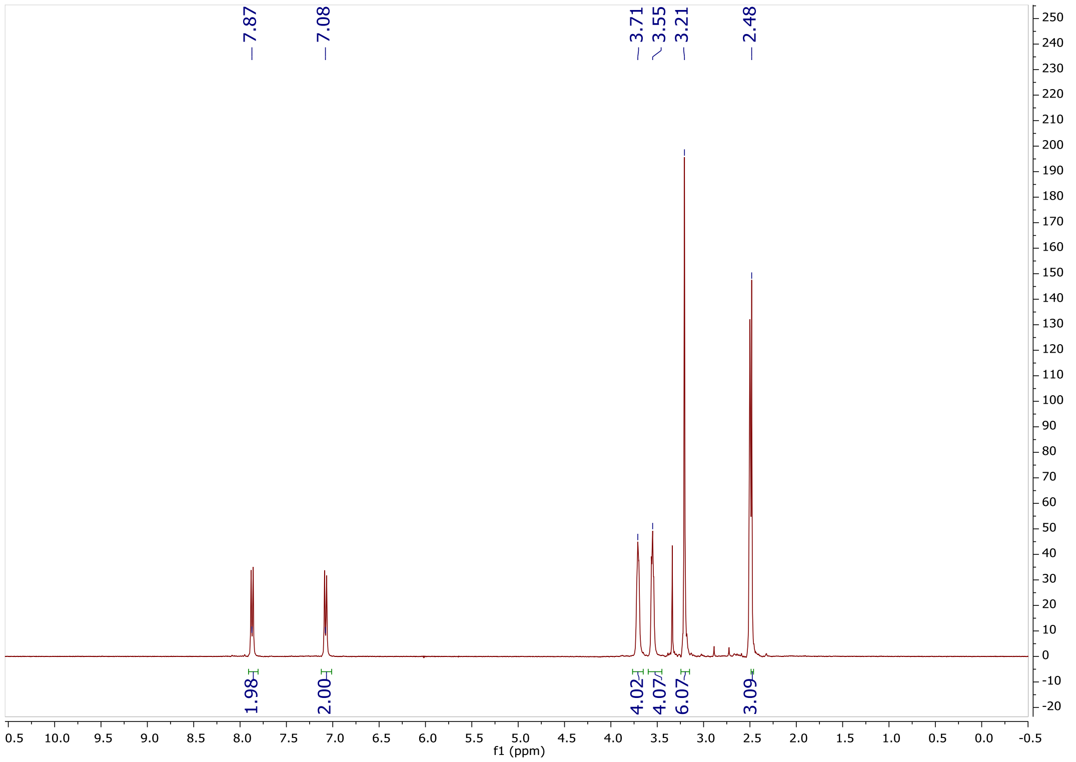


*Figure S11: $^{1}$H-NMR spectrum of 4-(4-acetylphenyl)-1,1-dimethylpiperazin-1-ium iodide, measured at 293 K in DMSO-$d_6$*

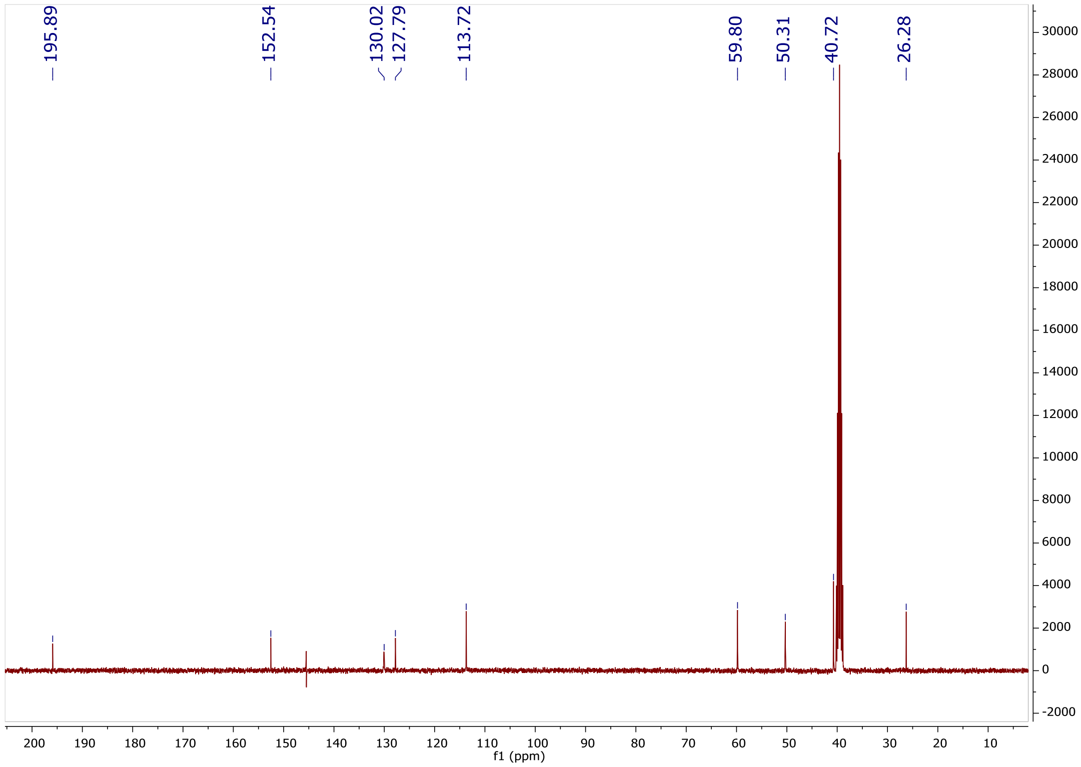


*Figure S12: $^{13}$C-NMR spectrum of 4-(4-acetylphenyl)-1,1-dimethylpiperazin-1-ium iodide, measured at 293 K in DMSO-$d_6$*

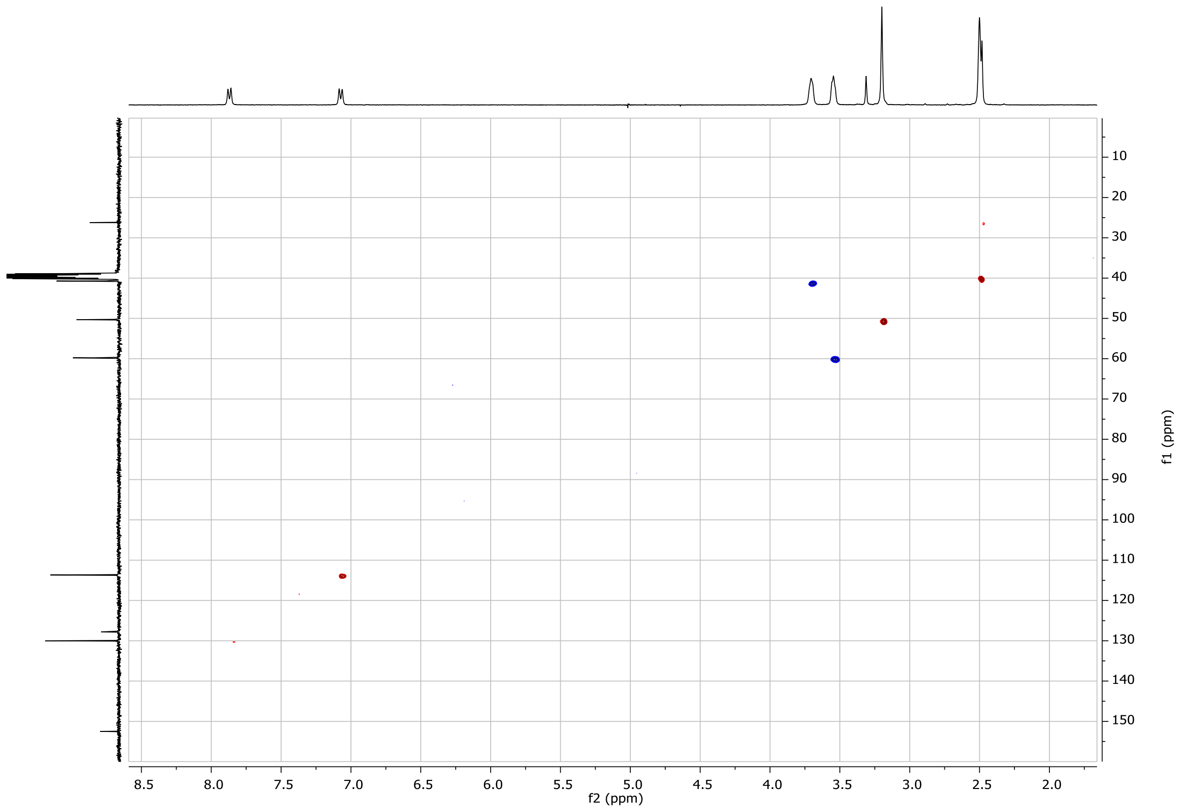


*Figure S13: HSQC spectrum of 4-(4-acetylphenyl)-1,1-dimethylpiperazin-1-ium iodide, measured at 293 K in DMSO-$d_6$*

## 3.6. 3-Methyl-2-methylthiobenzothiazolium *p*-toluenesulfonate

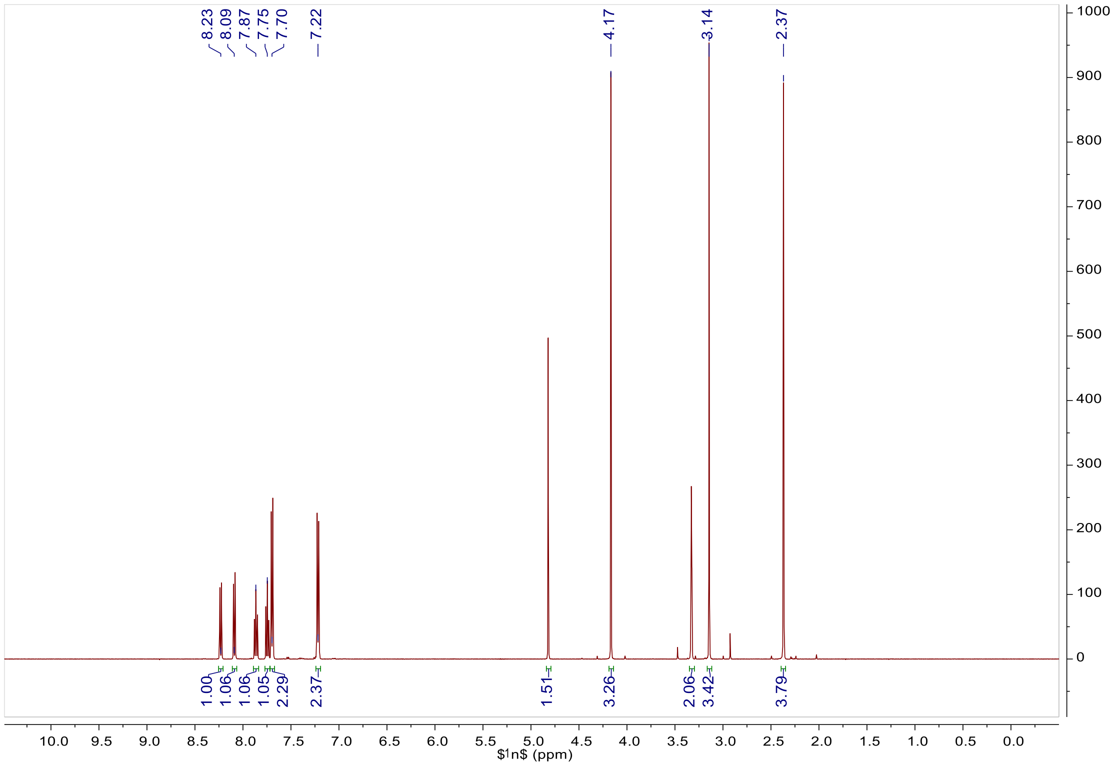


*Figure S14: $^{1}$H-NMR spectrum of 3-Methyl-2-methylthiobenzothiazolium p-toluenesulfonate, measured at 293 K in, methanol-$d_4$.*

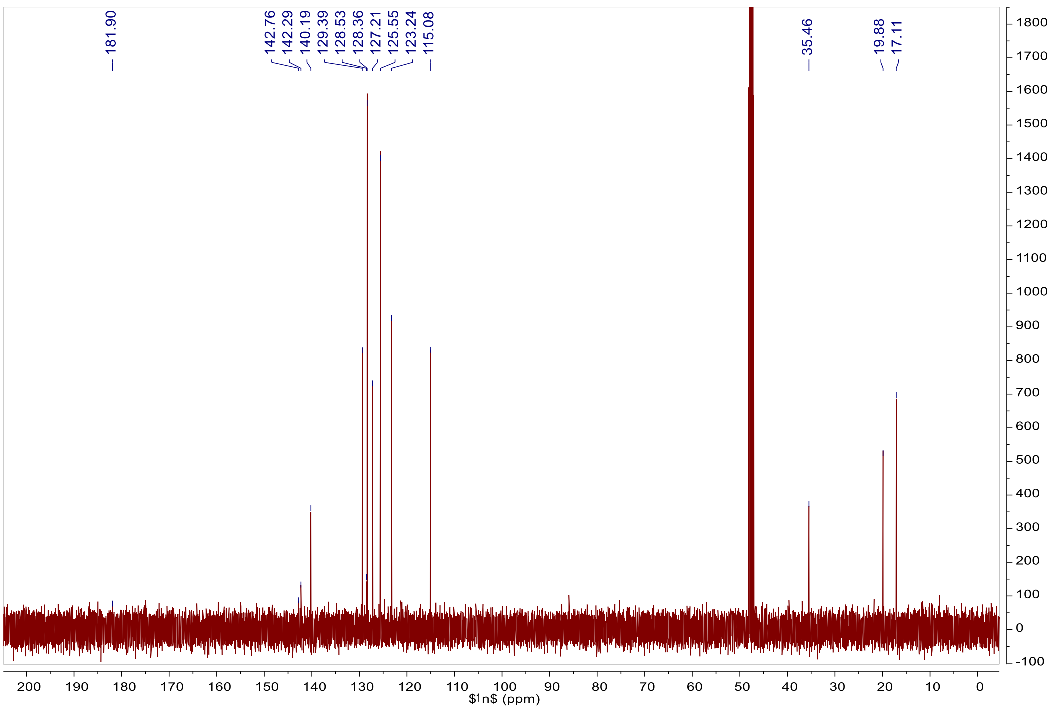


*Figure S15: $^{13}$C-NMR spectrum of 3-Methyl-2-methylthiobenzothiazolium p-toluenesulfonate, measured at 293 K in methanol-$d_4$.*

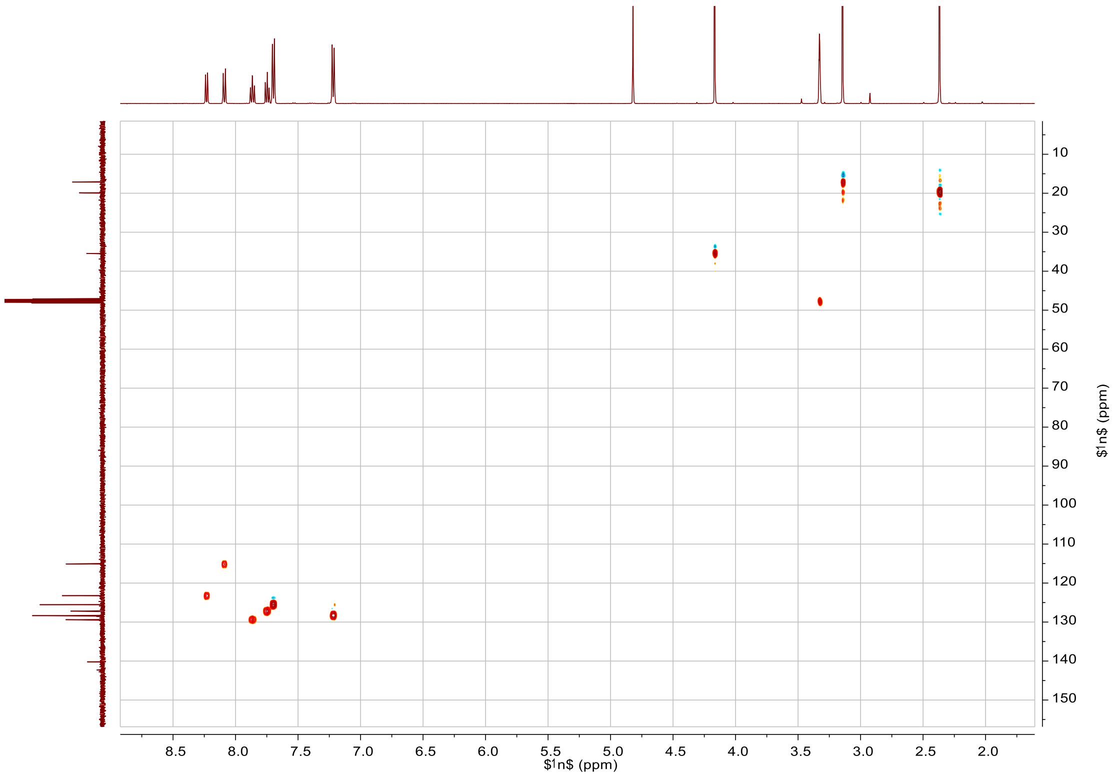


*Figure S16: HSQC spectrum of 3-Methyl-2-methylthiobenzothiazolium p-toluenesulfonate, measured at 293 K in methanol-d4.*

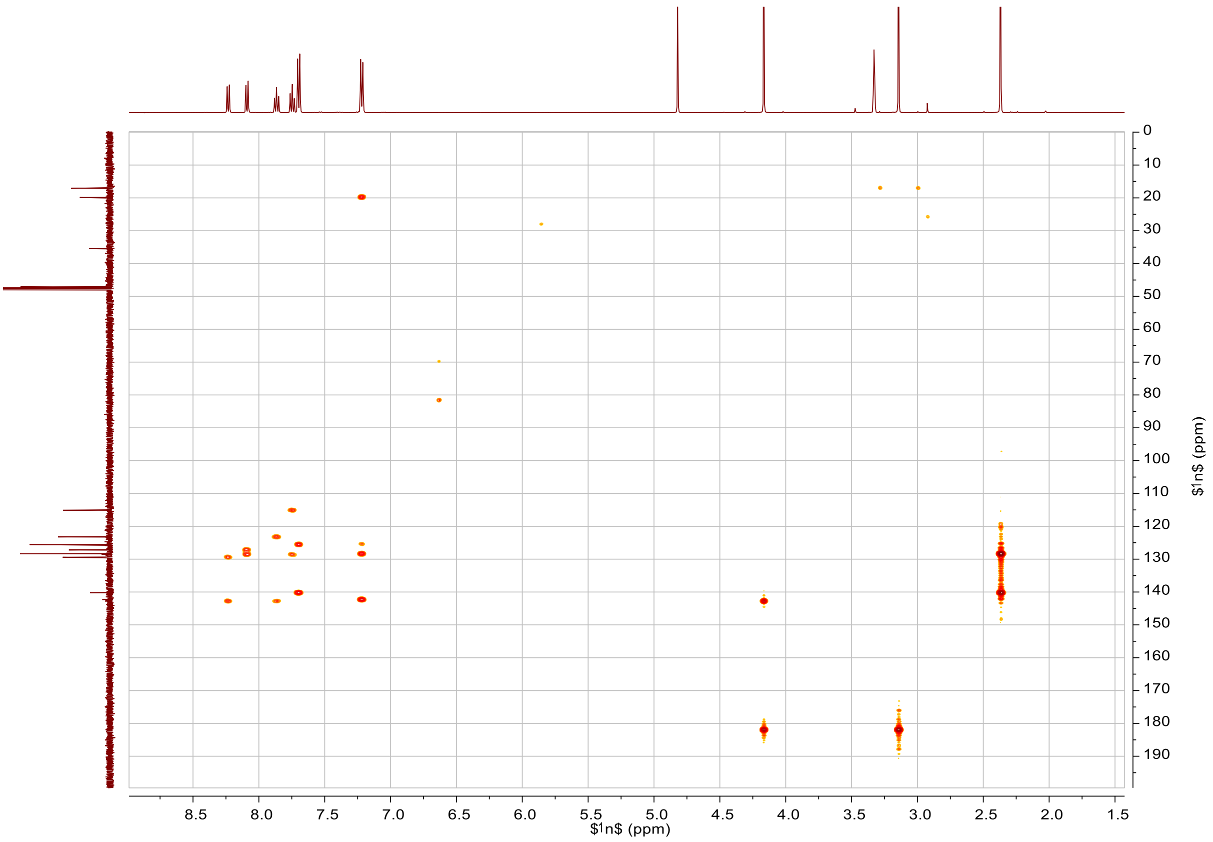


*Figure S17: HMBC spectrum of 3-Methyl-2-methylthiobenzothiazolium p-toluenesulfonate, measured at 293 K in methanol-d4.*

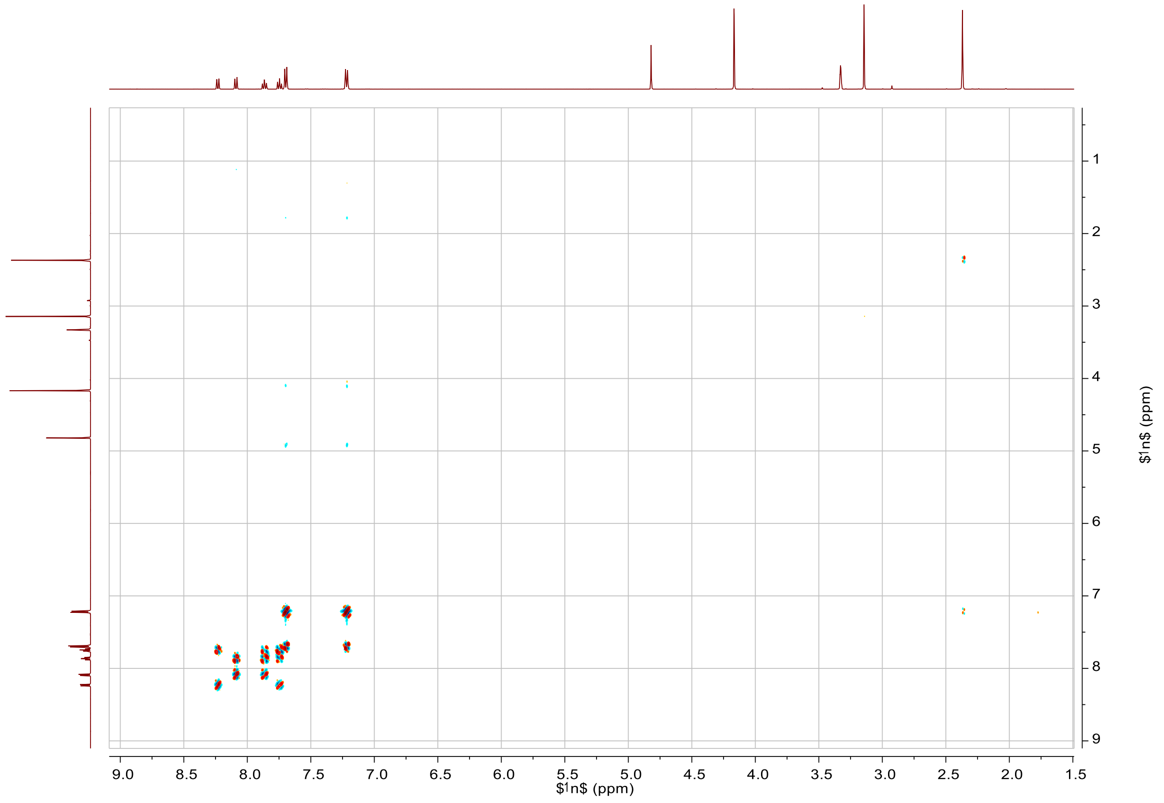


*Figure S18: COSY spectrum of 3-Methyl-2-methylthiobenzothiazolium p-toluenesulfonate, measured at 293 K in methanol-d4.*

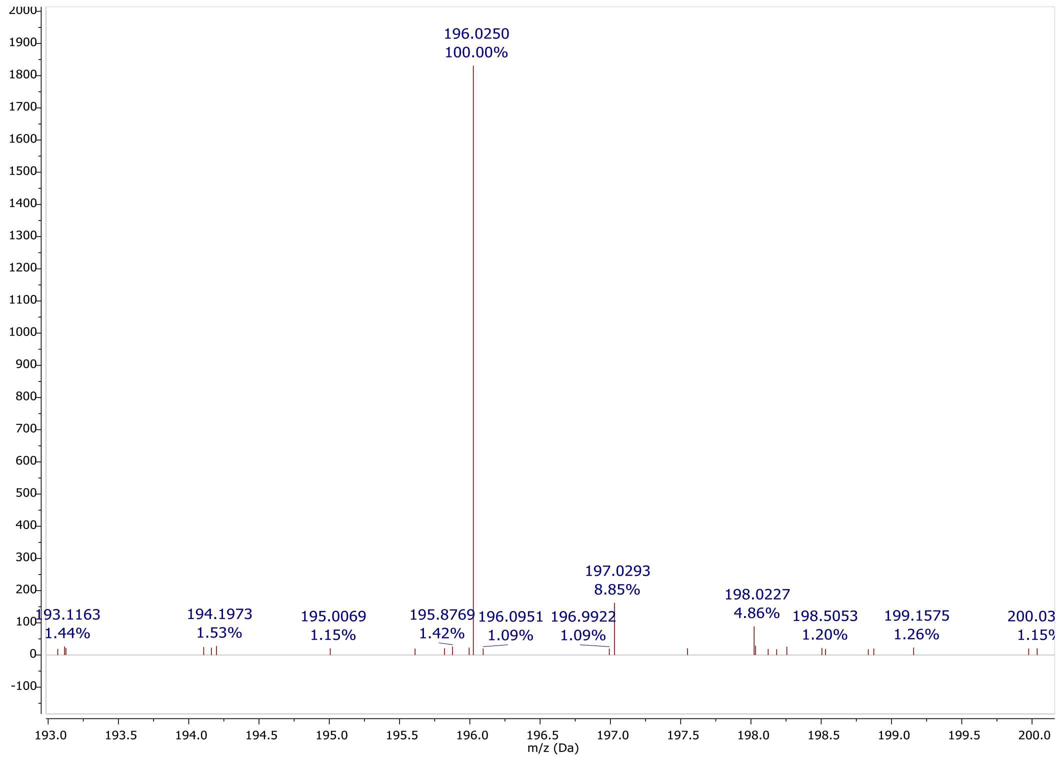


*Figure S19: HRMS spectrum of 3-Methyl-2-methylthiobenzothiazolium p-toluenesulfonate*

## 3.7. 2-(1H-Indol-1-yl)-N,N-dimethylethan-1-amine

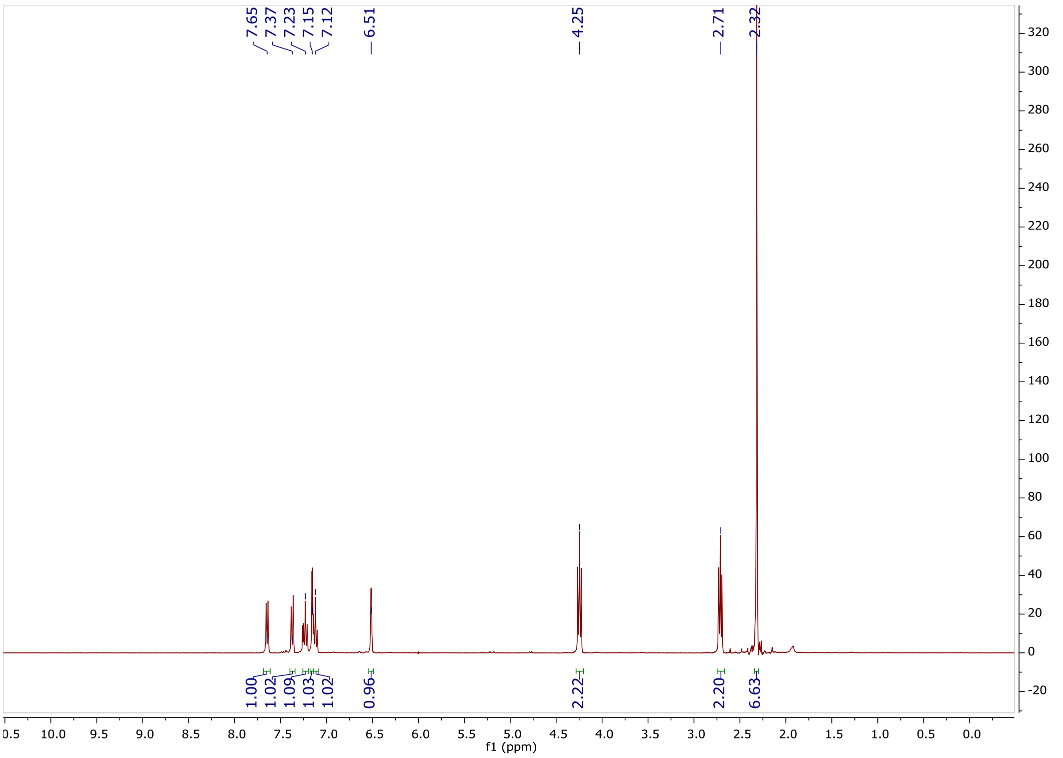


*Figure S20: $^{1}$H-NMR spectrum of 2-(1H-indol-1-yl)-N,N-dimethylethan-1-amine, measured at 293 K in $CDCl_3$*

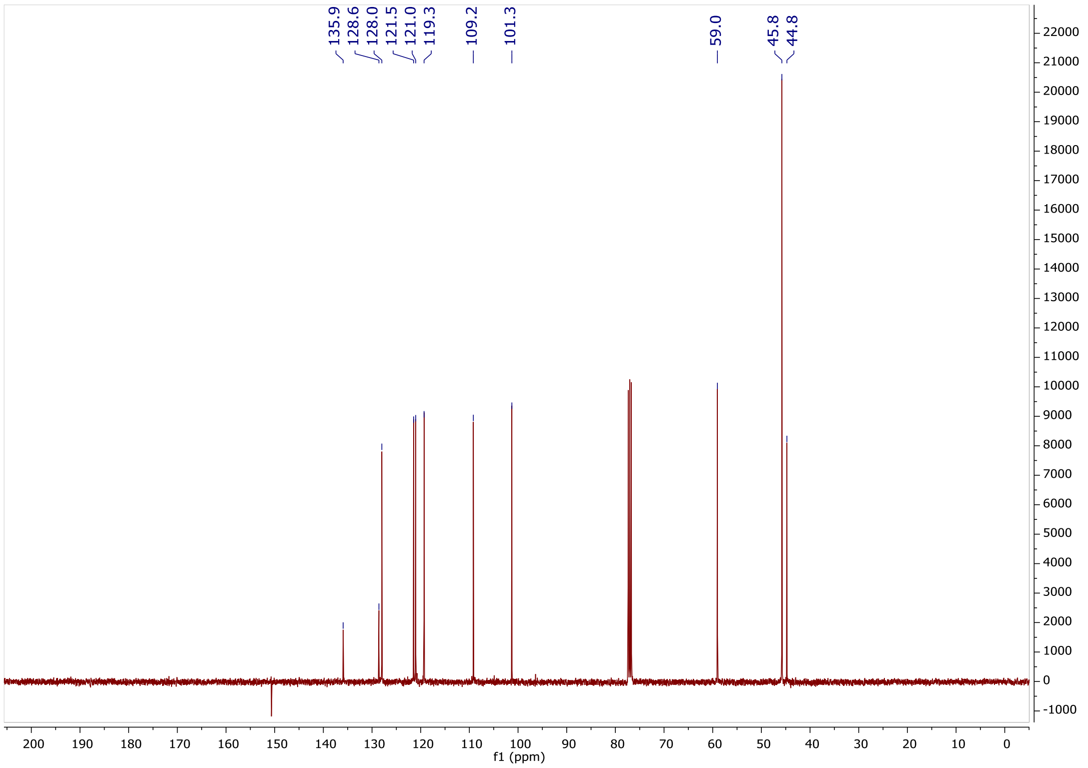


*Figure S21: $^{13}$C-NMR spectrum of 2-(1H-indol-1-yl)-N,N-dimethylethan-1-amine, measured at 293 K in $CDCl_3$*

## 3.8. 2-(Indolin-1-yl)-N,N-dimethylethan-1-amine

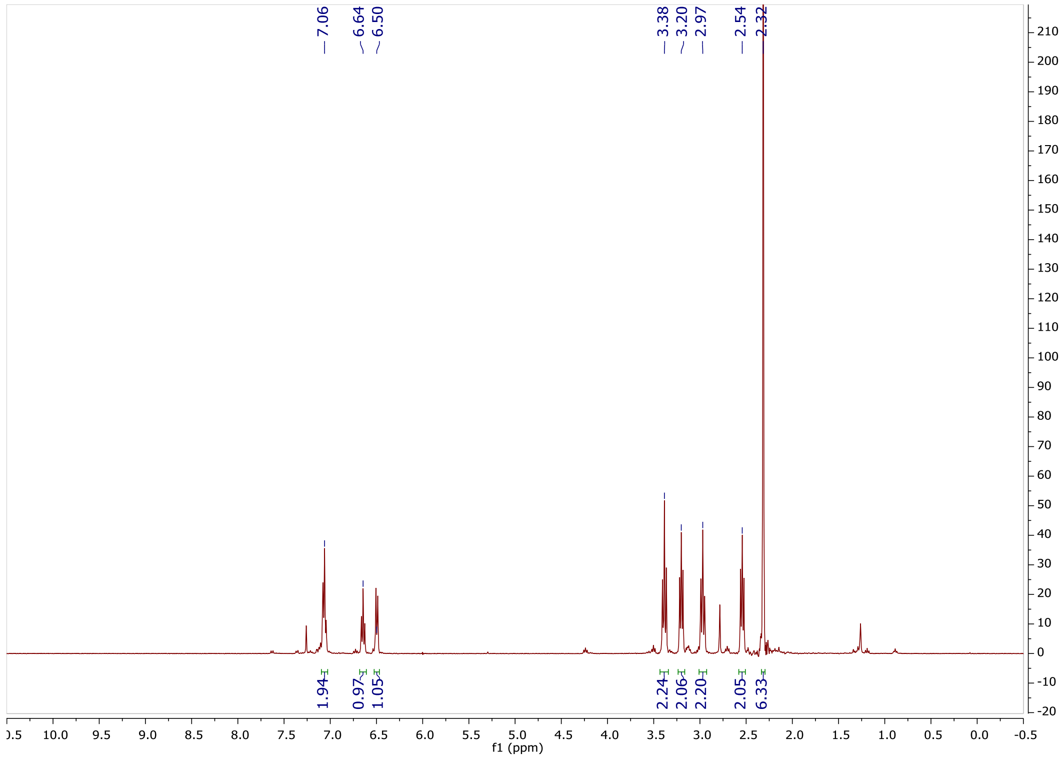


*Figure S22: $^{1}$H-NMR spectrum of 2-(indolin-1-yl)-N,N-dimethylethan-1-amine, measured at 293 K in $CDCl_3$*

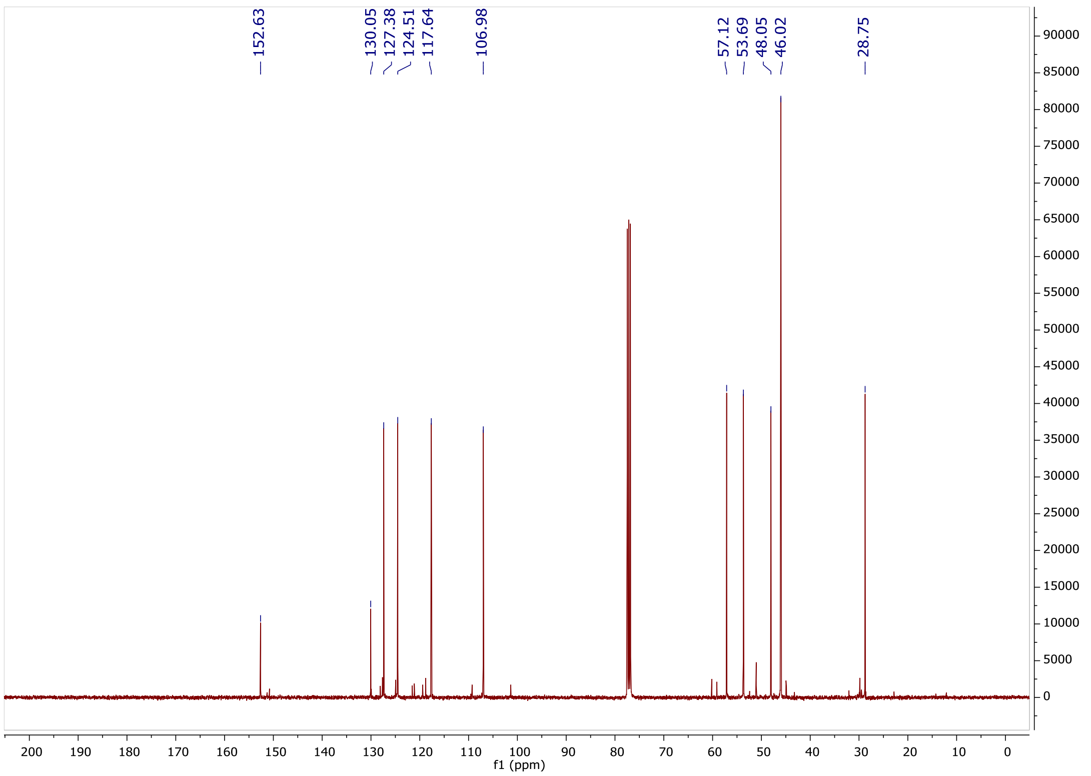


*Figure S23: $^{13}$C-NMR spectrum of 2-(indolin-1-yl)-N,N-dimethylethan-1-amine, measured at 293 K in $CDCl_3$*

## 3.9. 1-(1-(2-(Dimethylamino)ethyl)indolin-5-yl)ethan-1-one

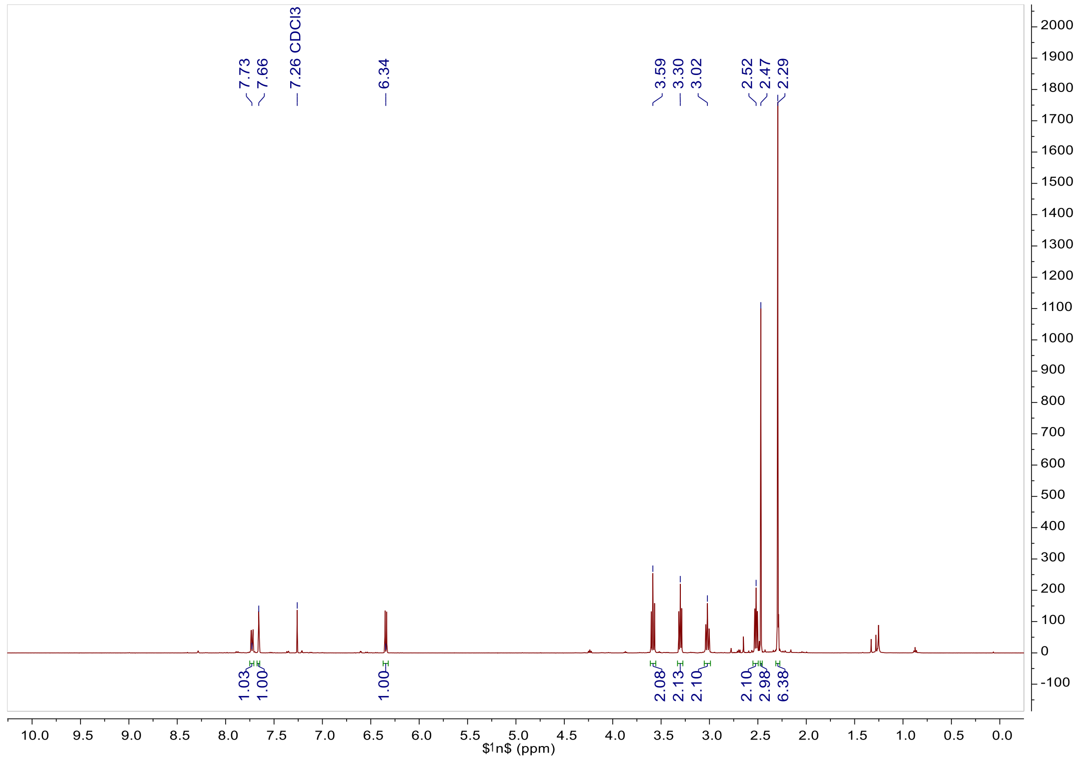


*Figure S24: $^{1}$H-NMR spectrum of 1-(1-(2-(dimethylamino)ethyl)indolin-5-yl)ethan-1-one, measured at 293 K in $CDCl_3$*

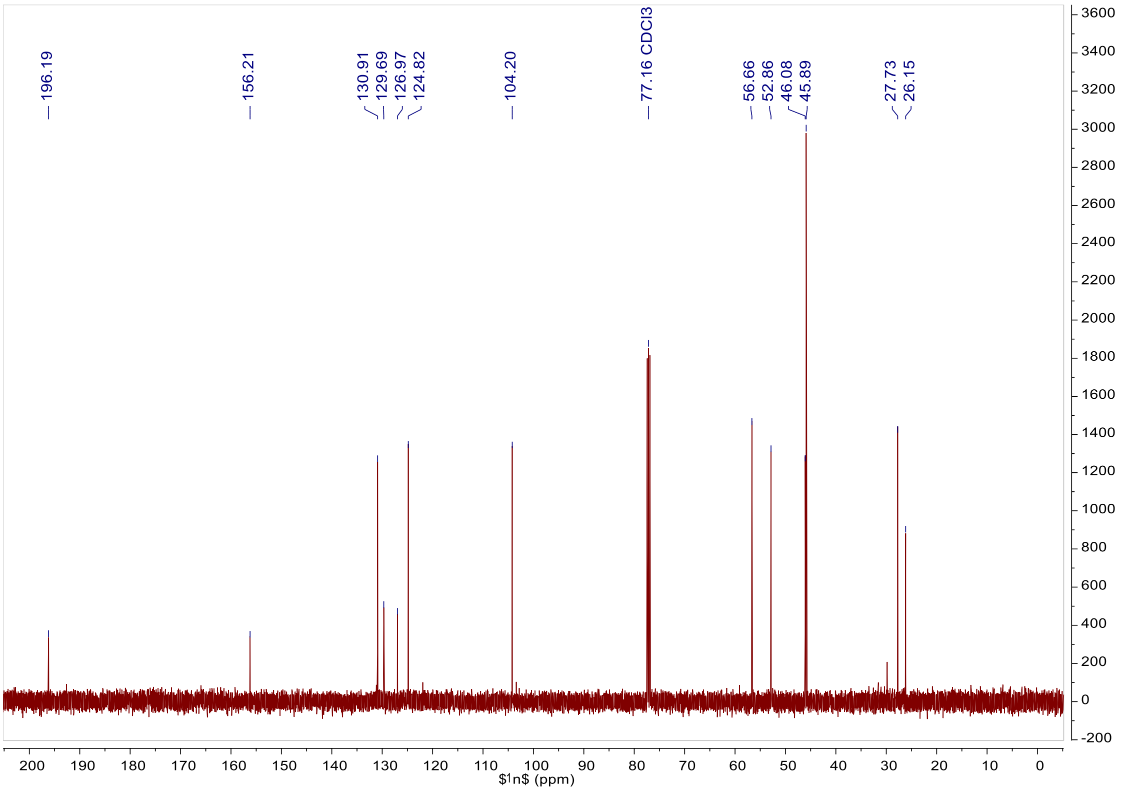


*Figure S25: $^{13}$C-NMR spectrum of 1-(1-(2-(dimethylamino)ethyl)indolin-5-yl)ethan-1-one, measured at 293 K in $CDCl_3$*

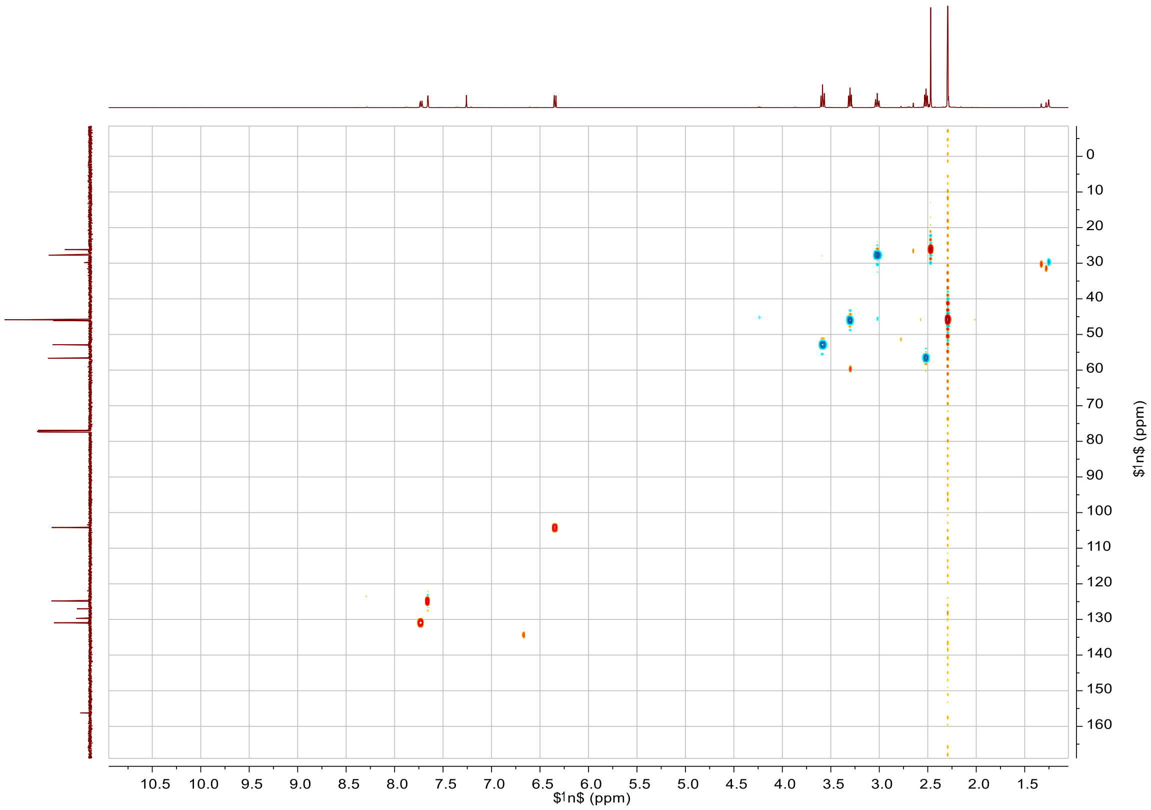


*Figure S26: HSQC spectrum of 1-(1-(2-(dimethylamino)ethyl)indolin-5-yl)ethan-1-one, measured at 293 K in $CDCl_3$*

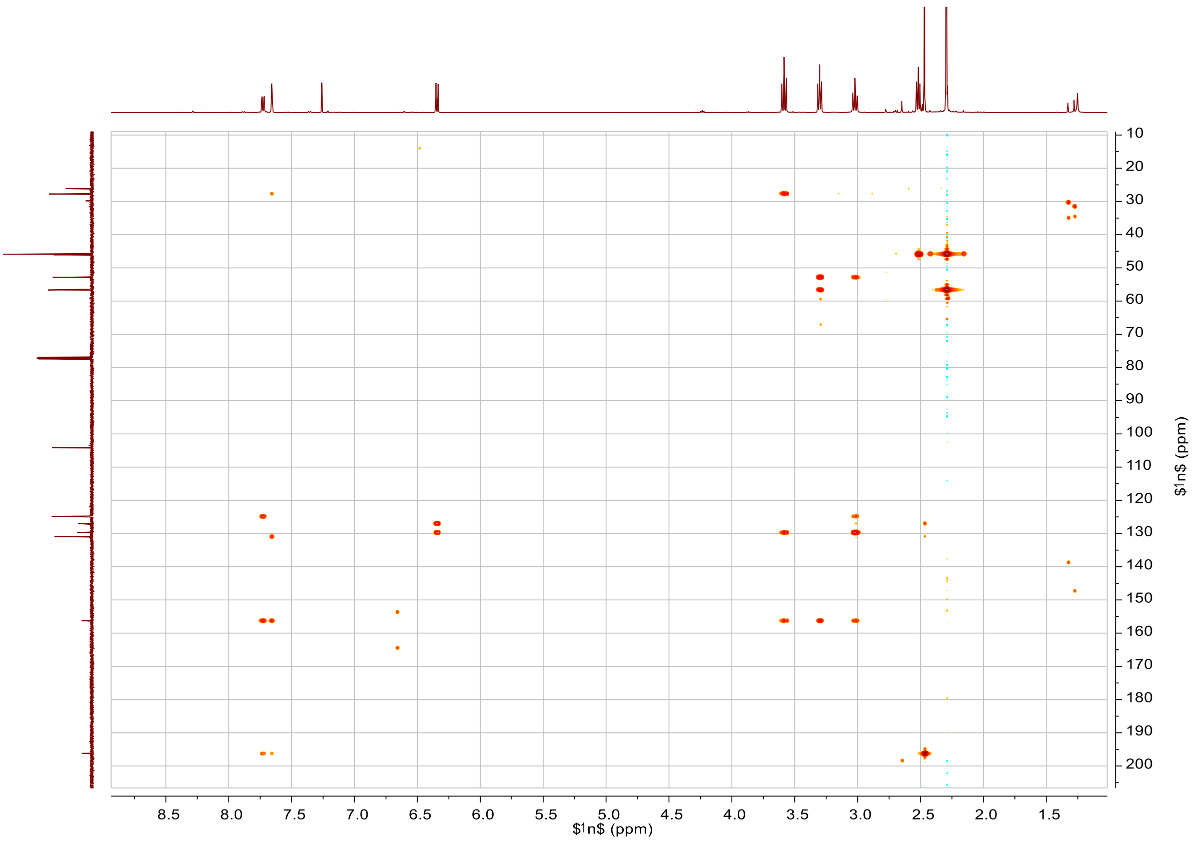


*Figure S27: HMBC spectrum of 1-(1-(2-(dimethylamino)ethyl)indolin-5-yl)ethan-1-one, measured at 293 K in $CDCl_3$*

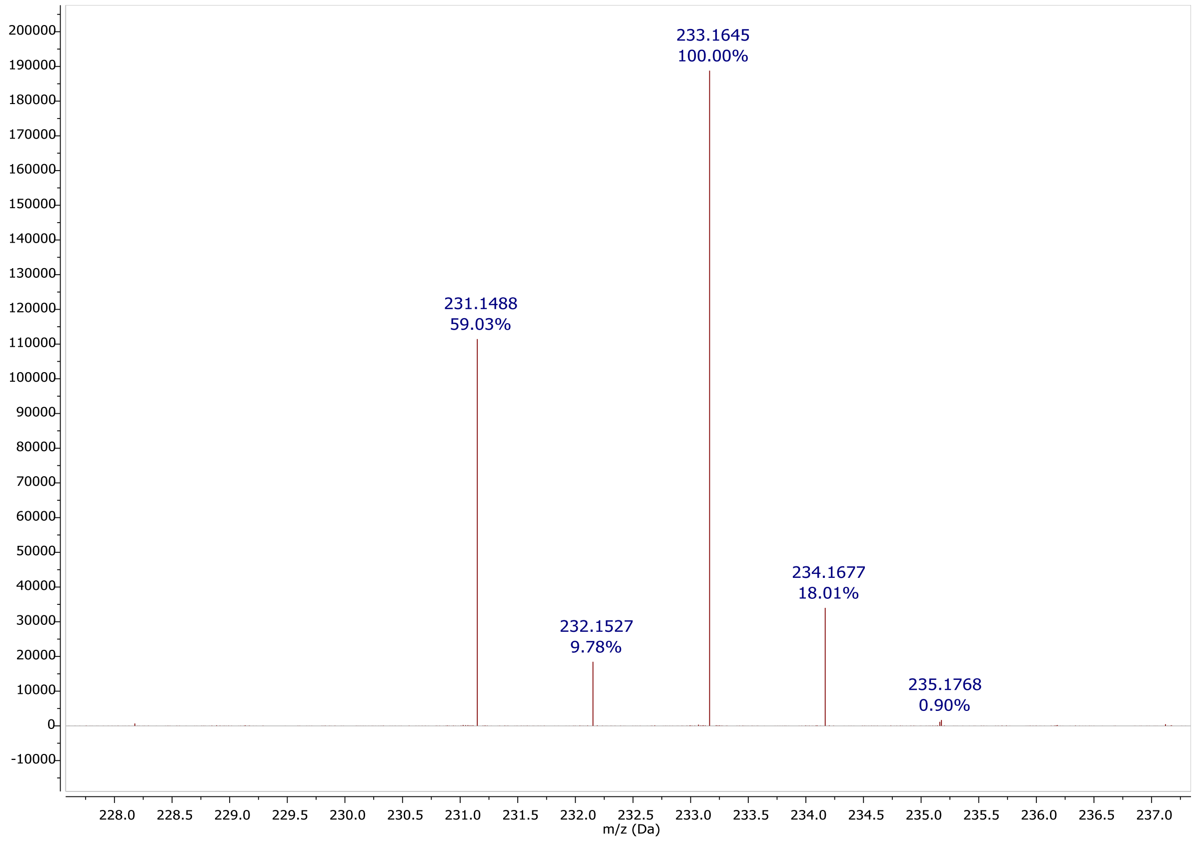


*Figure S28: HRMS spectrum of 1-(1-(2-(dimethylamino)ethyl)indolin-5-yl)ethan-1-one*

## 3.10. 2,4,6-DMA - 2,4,6-tris(4-(dimethylamino)phenyl)pyrylium trifluoroacetate

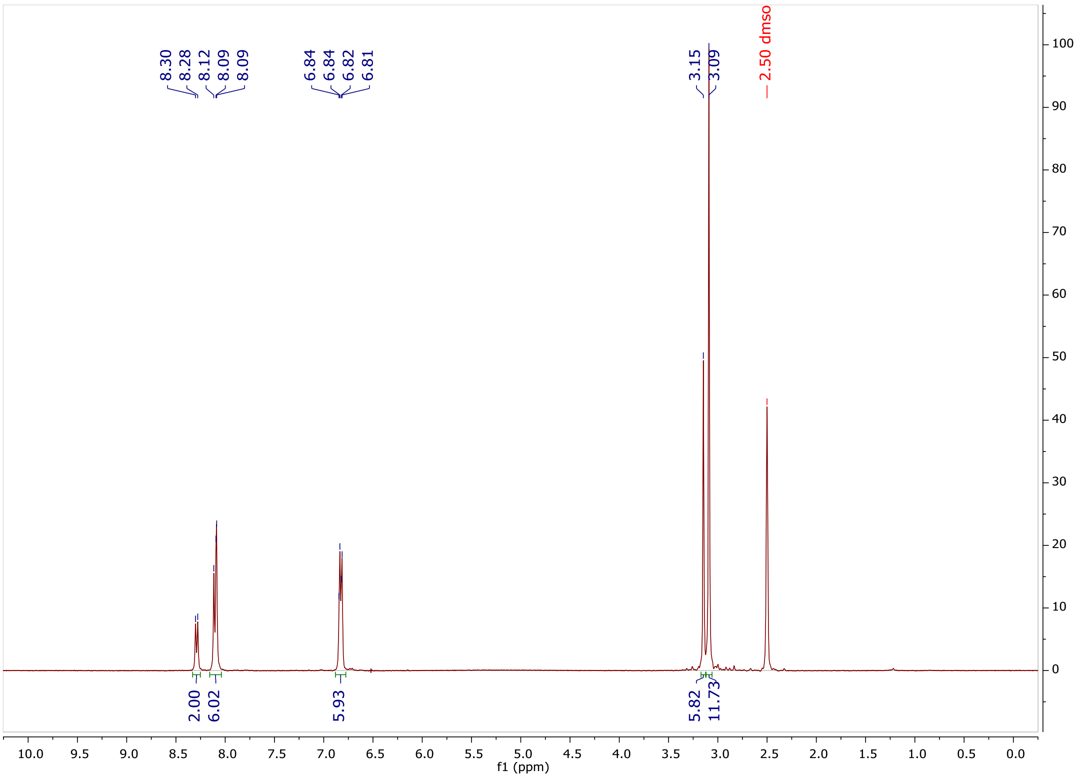


*Figure S29: $^{1}$H-NMR spectrum of 2,4,6-tris(4-(dimethylamino)phenyl)pyrylium trifluoroacetate, measured at 293 K in DMSO-$d_6$*

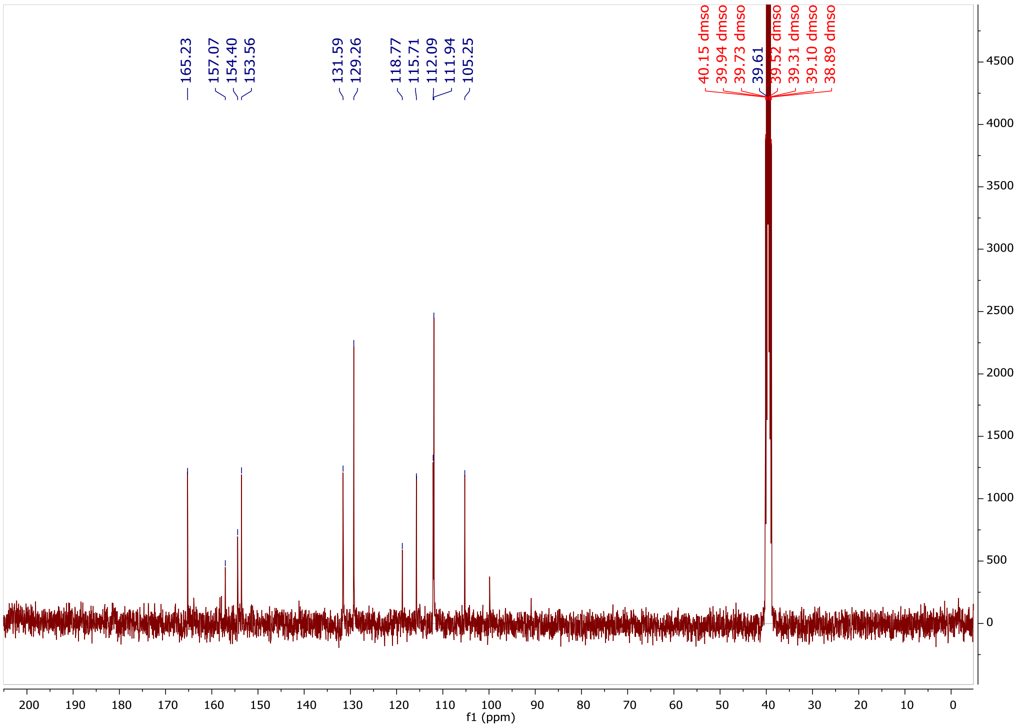


*Figure S30: $^{13}$C-NMR spectrum of 2,4,6-tris(4-(dimethylamino)phenyl)pyrylium trifluoroacetate, measured at 293 K in DMSO-$d_6$*

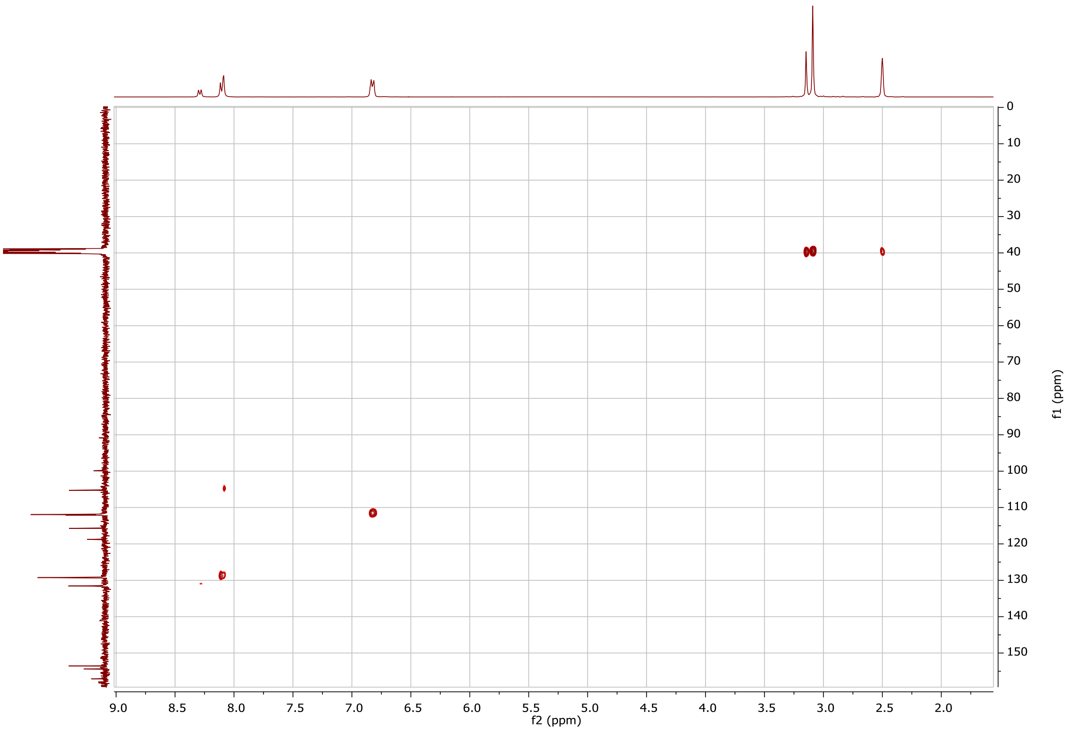


*Figure S31: HSQC spectrum of 2,4,6-tris(4-(dimethylamino)phenyl)pyrylium trifluoroacetate, measured at 293 K in DMSO-$d_6$*

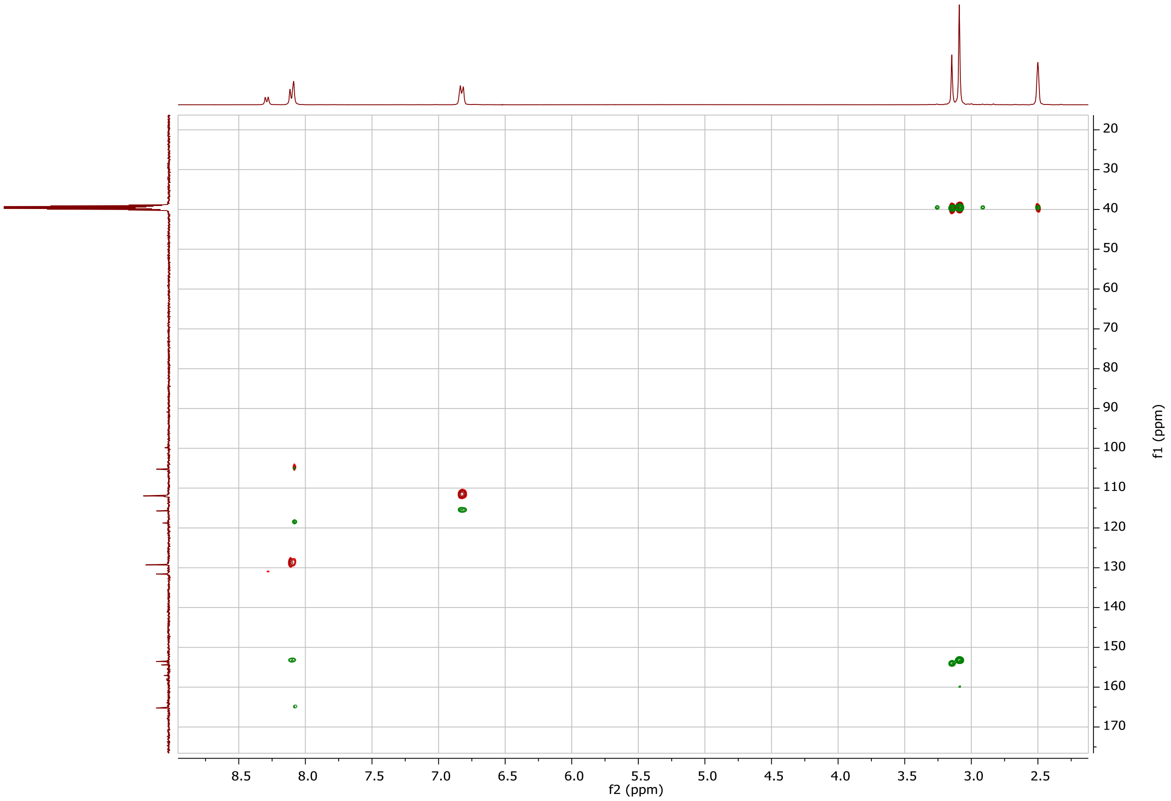


*Figure S32: HMBC spectrum of 2,4,6-tris(4-(dimethylamino)phenyl)pyrylium trifluoroacetate, measured at 293 K in DMSO-$d_6$*

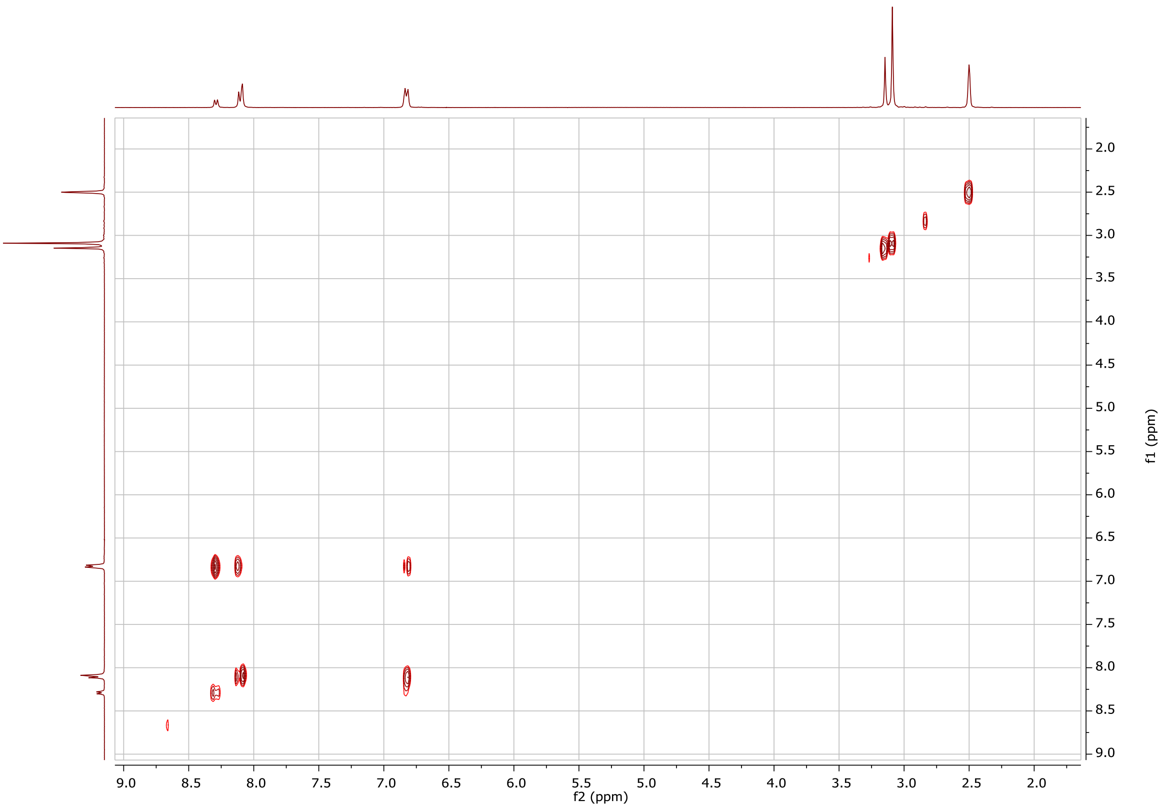


*Figure S33: COSY spectrum of 2,4,6-tris(4-(dimethylamino)phenyl)pyrylium trifluoroacetate, measured at 293 K in DMSO-d$_6$*

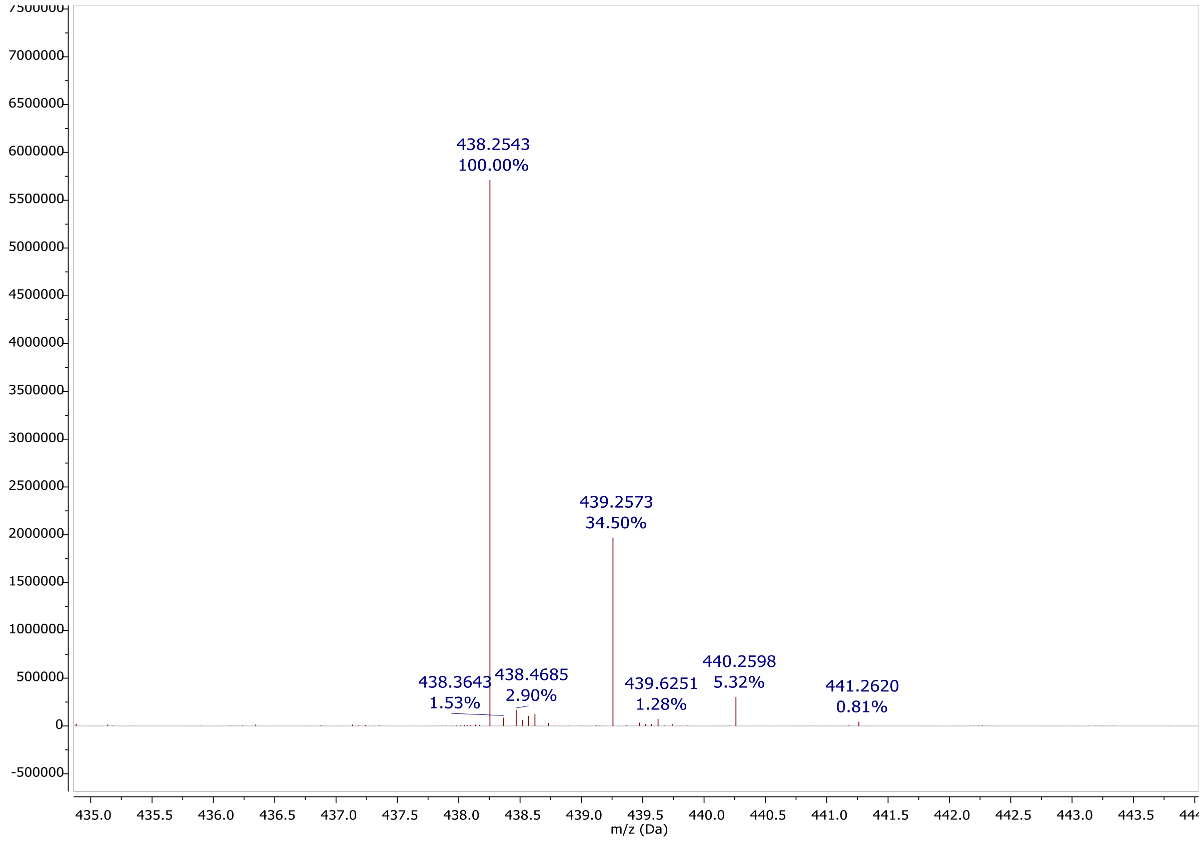


*Figure S34: HRMS spectrum of 2,4,6-tris(4-(dimethylamino)phenyl)pyrylium trifluoroacetate*

### 3.11. 2,6-NMP-4-DMA - 4,4'-((4-(4-(dimethylamino)phenyl)pyrylium-2,6-diyl)bis(4,1-phenylene))bis(1-methylpiperazin-1-ium) tris(trifluoroacetate)

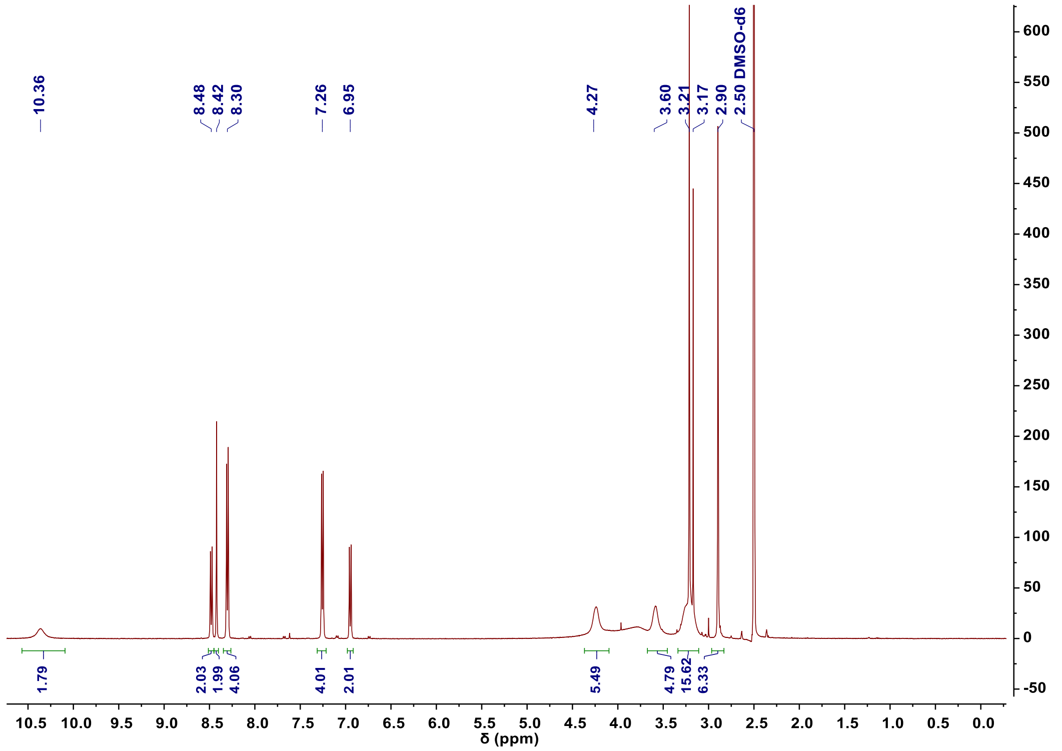


*Figure S35: $^{1}$H-NMR spectrum of 4,4'-((4-(4-(dimethylamino)phenyl)pyrylium-2,6-diyl)bis(4,1-phenylene))bis(1-methylpiperazin-1-ium) tris(trifluoroacetate), measured at 293 K in DMSO-$d_6$. This NMR spectrum was recorded from the same sample that was used for spectroscopical purposes.*

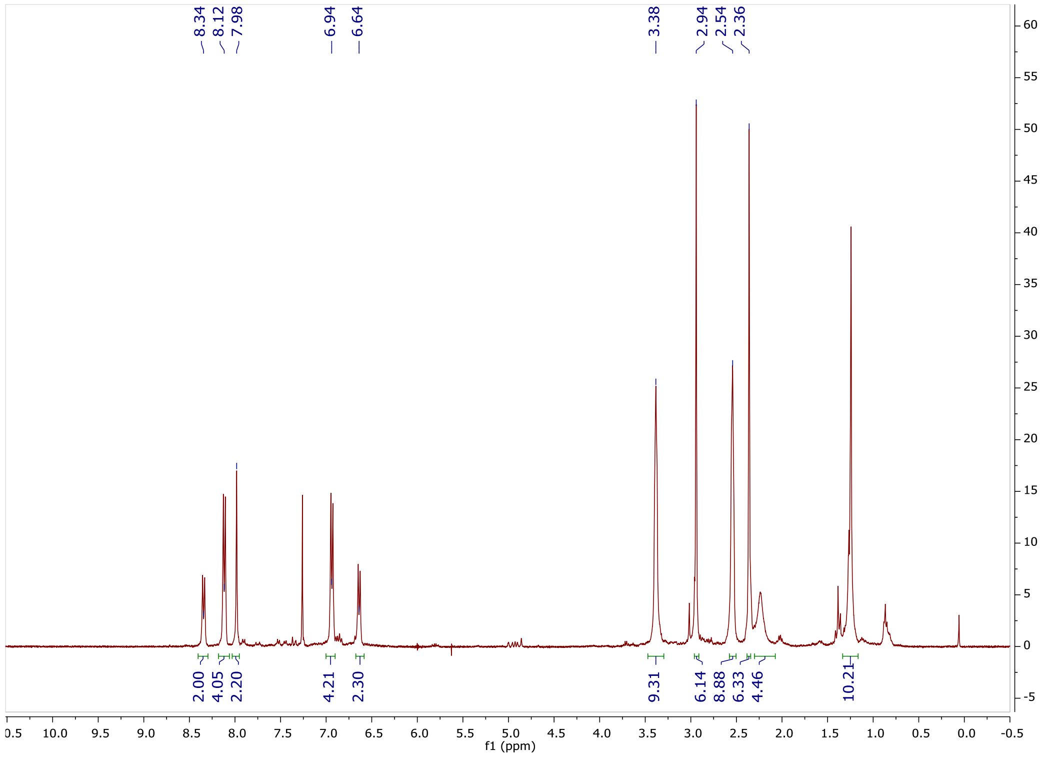


*Figure S36: $^{1}$H-NMR spectrum of 4-(4-(dimethylamino)phenyl)-2,6-bis(4-(4-methylpiperazin-1-yl)phenyl)pyrylium trifluoroacetate, measured at 293 K in $CDCl_3$*

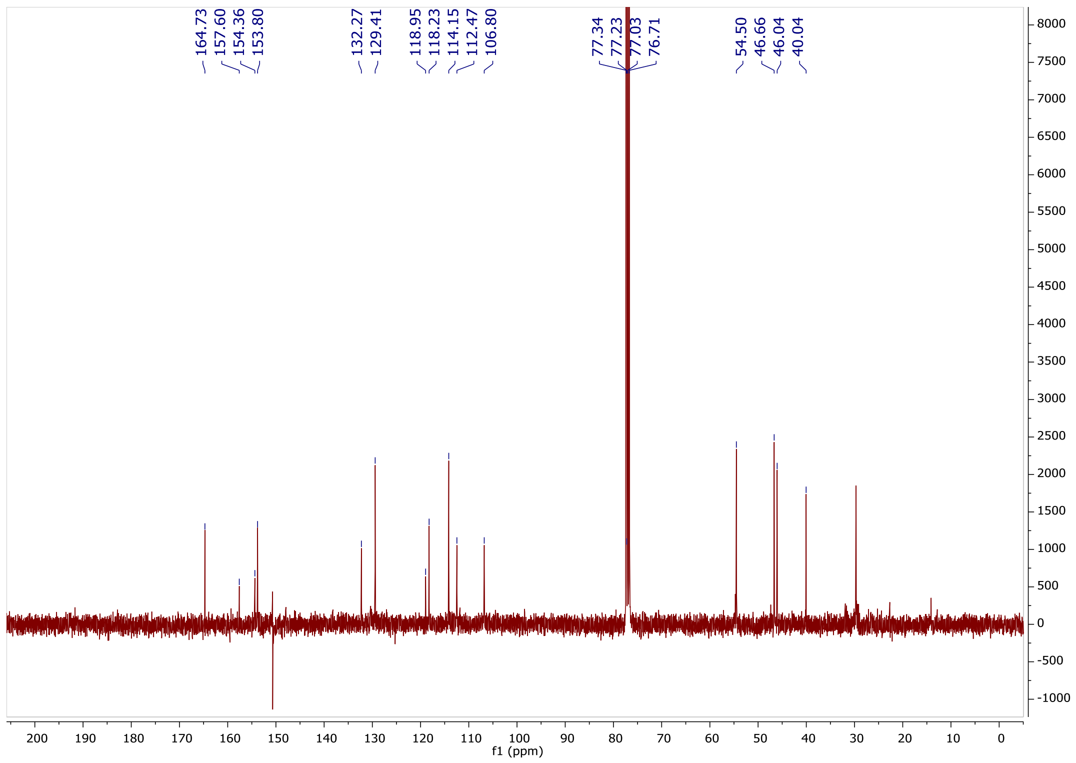


*Figure S37: $^{13}$C-NMR spectrum of 4-(4-(dimethylamino)phenyl)-2,6-bis(4-(4-methylpiperazin-1-yl)phenyl)pyrylium trifluoroacetate, measured at 293 K in $CDCl_3$*

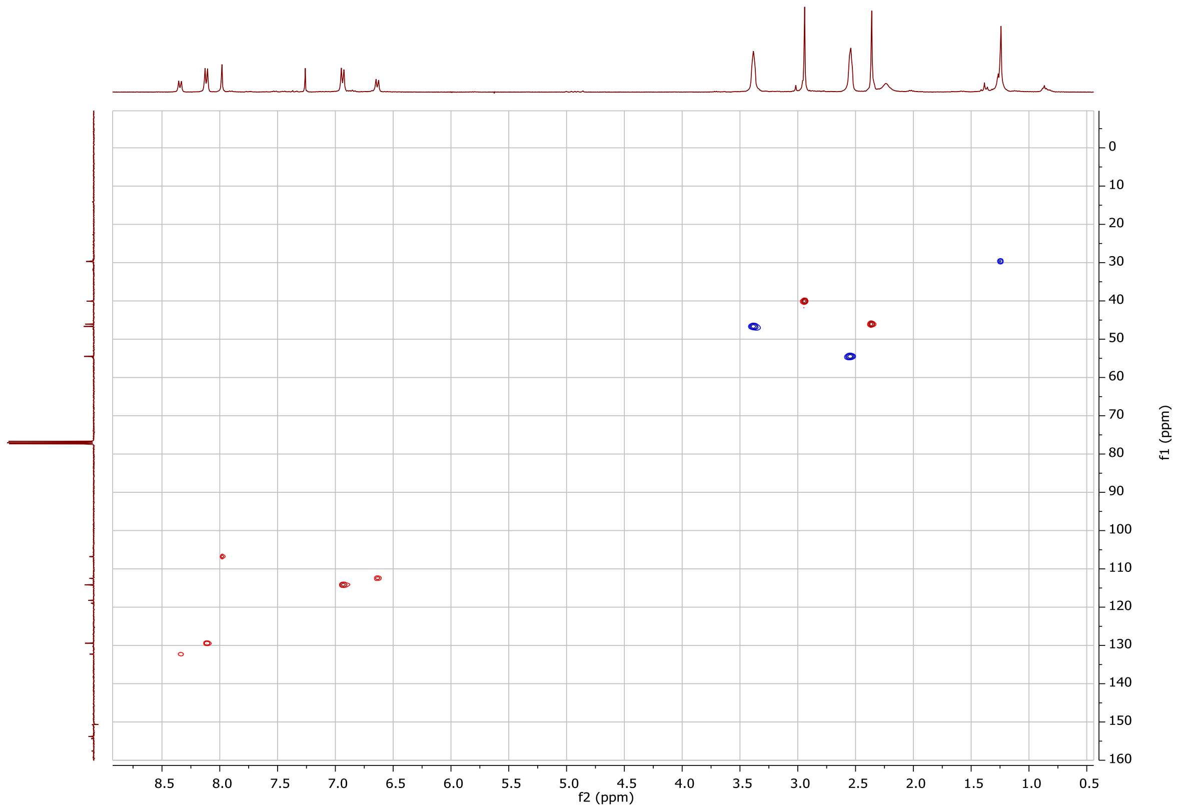


*Figure S38: HSQC spectrum of 4-(4-(dimethylamino)phenyl)-2,6-bis(4-(4-methylpiperazin-1-yl)phenyl)pyrylium trifluoroacetate, measured at 293 K in $CDCl_3$*

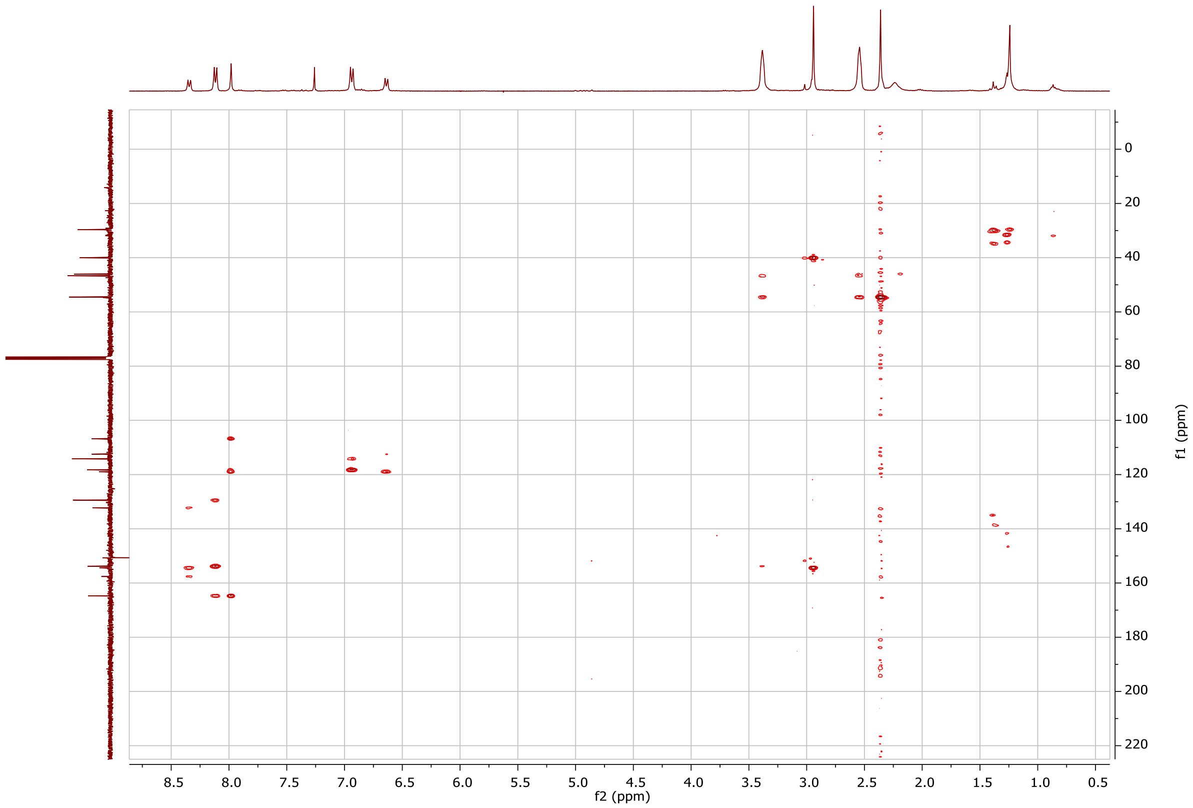


*Figure S39: HMBC spectrum of 4-(4-(dimethylamino)phenyl)-2,6-bis(4-(4-methylpiperazin-1-yl)phenyl)pyrylium trifluoroacetate, measured at 293 K in $CDCl_3$*

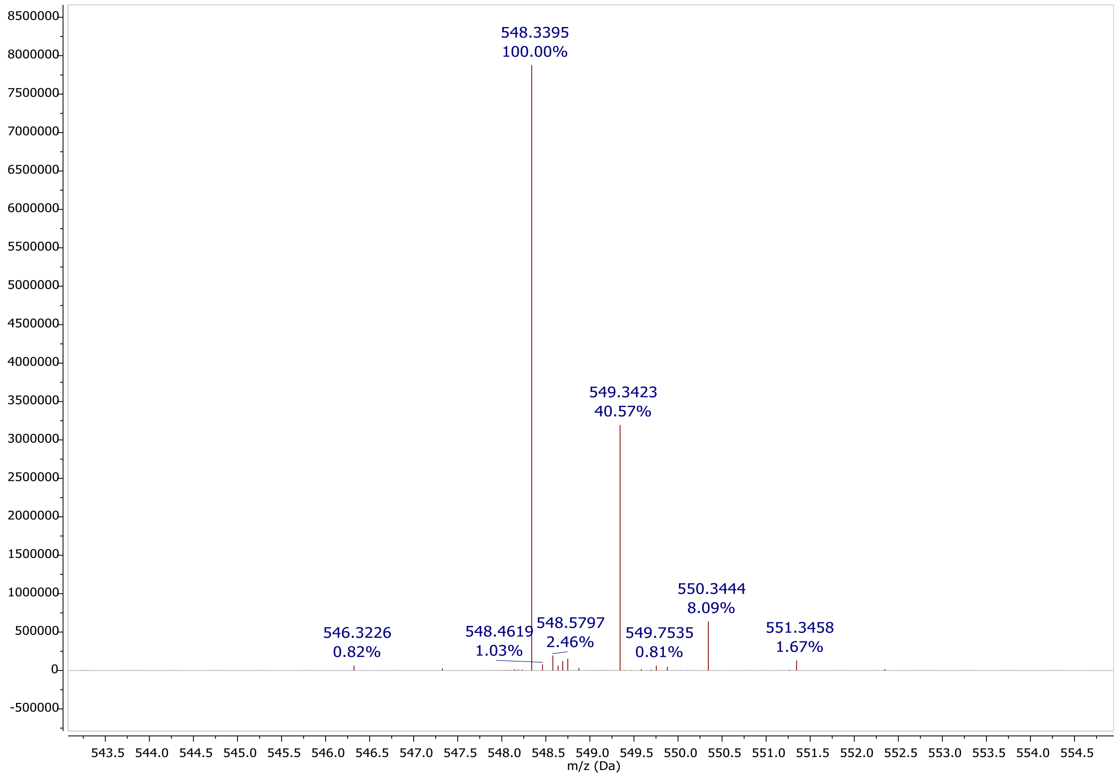


*Figure S40: HRMS spectrum of 4-(4-(dimethylamino)phenyl)-2,6-bis(4-(4-methylpiperazin-1-yl)phenyl)pyrylium trifluoroacetate.*

### 3.12. 2,6-DMA-4-NMP - 4-(4-(2,6-bis(4-(dimethylamino)phenyl)pyrylium-4-yl)phenyl)-1-methylpiperazin-1-ium bis(trifluoroacetate)

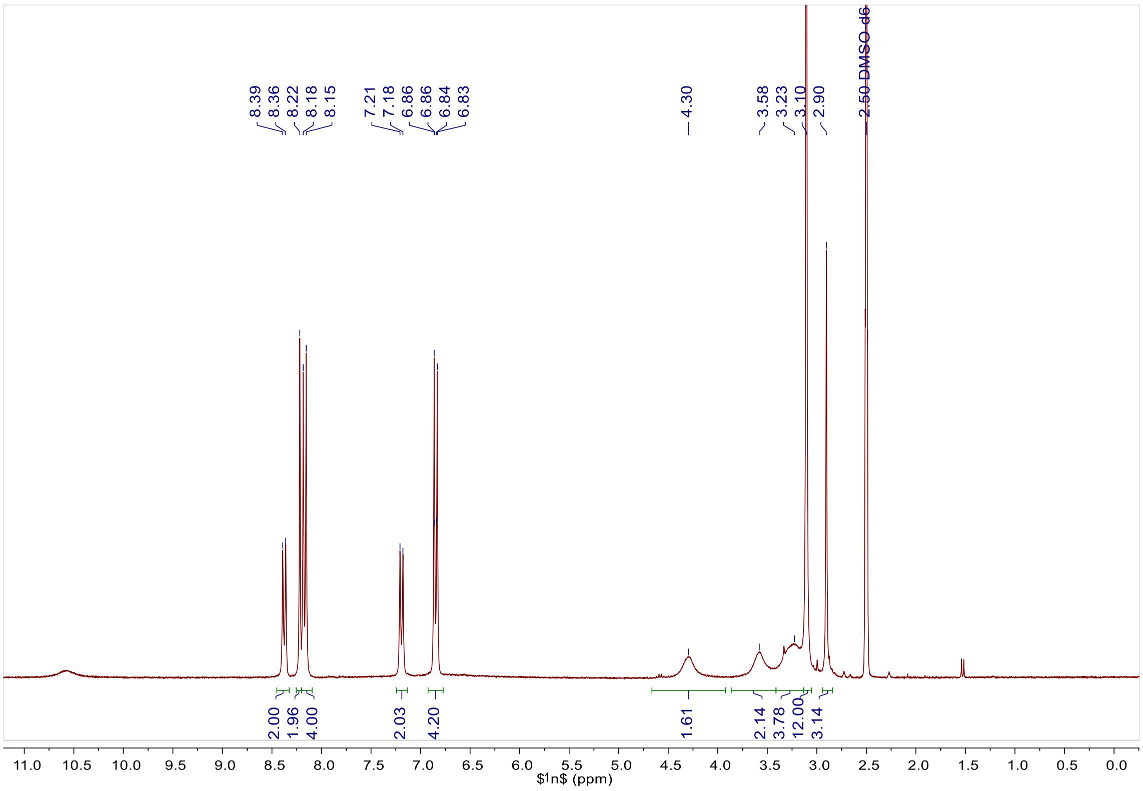

*Figure S41: $^{1}$H-NMR spectrum of 4-(4-(2,6-bis(4-(dimethylamino)phenyl)pyrylium-4-yl)phenyl)-1-methylpiperazin-1-ium bis(trifluoroacetate), measured at 293 K in DMSO-$d_6$*

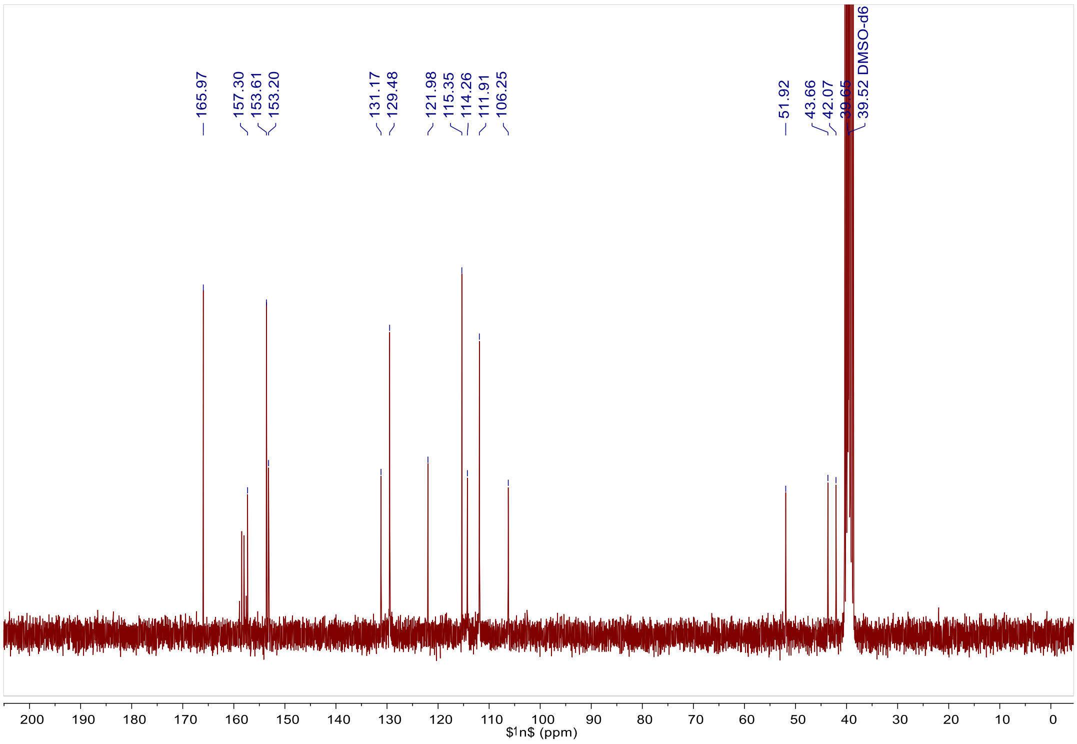

*Figure S42: $^{13}$C-NMR spectrum of 4-(4-(2,6-bis(4-(dimethylamino)phenyl)pyrylium-4-yl)phenyl)-1-methylpiperazin-1-ium bis(trifluoroacetate, measured at 293 K in DMSO-$d_6$*

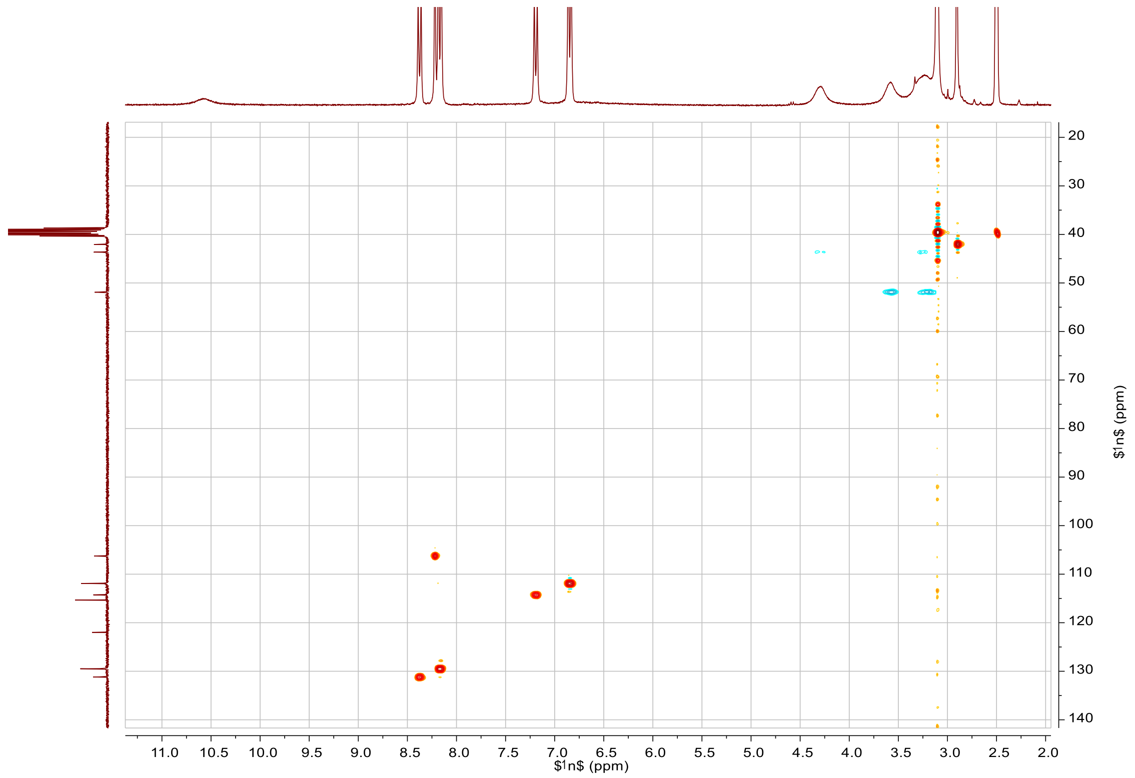


*Figure S43: HSQC spectrum of 4-(4-(2,6-bis(4-(dimethylamino)phenyl)pyrylium-4-yl)phenyl)-1-methylpiperazin-1-ium bis(trifluoroacetate), measured at 293 K in DMSO-$d_6$*

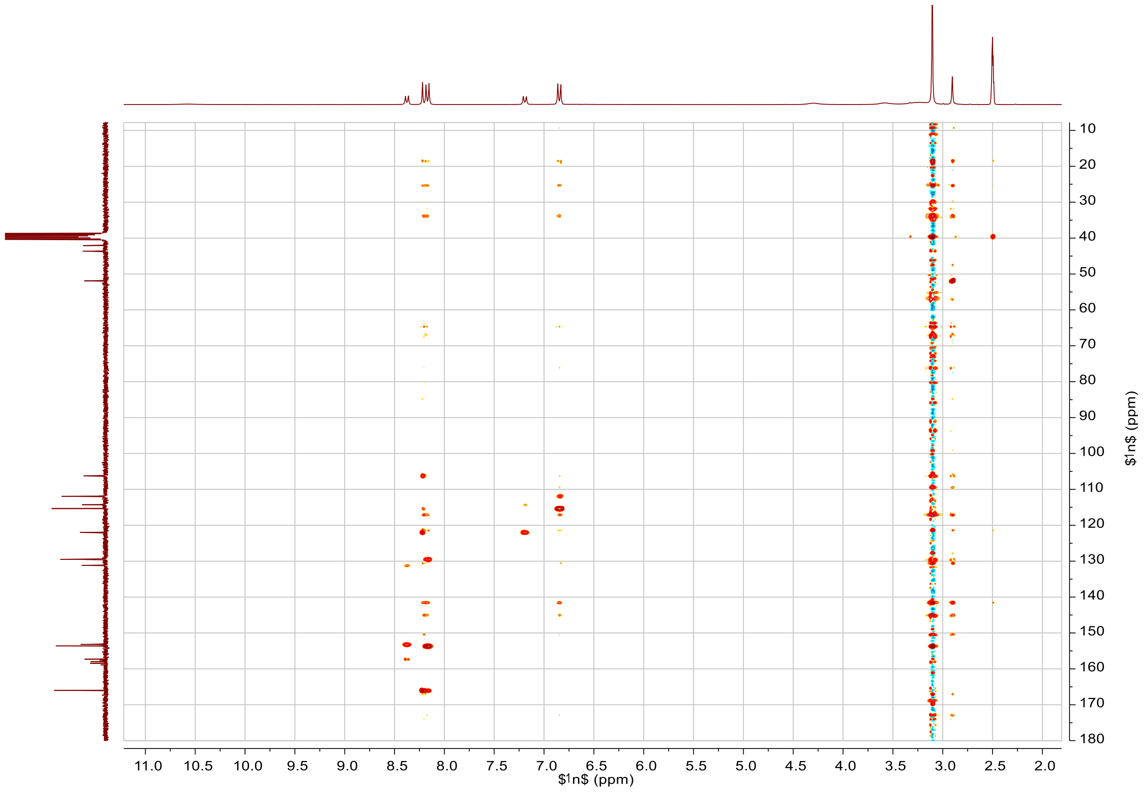


*Figure S44: HMBC spectrum of 4-(4-(2,6-bis(4-(dimethylamino)phenyl)pyrylium-4-yl)phenyl)-1-methylpiperazin-1-ium bis(trifluoroacetate), measured at 293 K in DMSO-$d_6$*

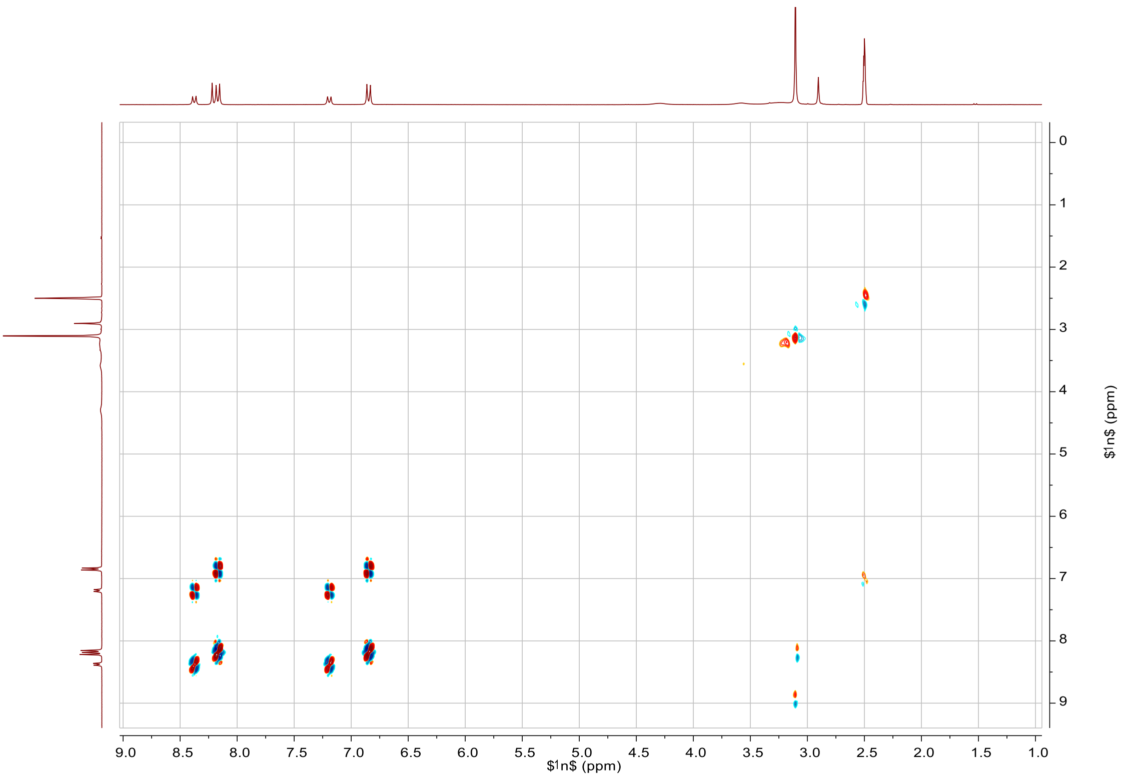


*Figure S45: COSY spectrum of 4-(4-(2,6-bis(4-(dimethylamino)phenyl)pyrylium-4-yl)phenyl)-1-methylpiperazin-1-ium bis(trifluoroacetate), measured at 293 K in DMSO-$d_6$*

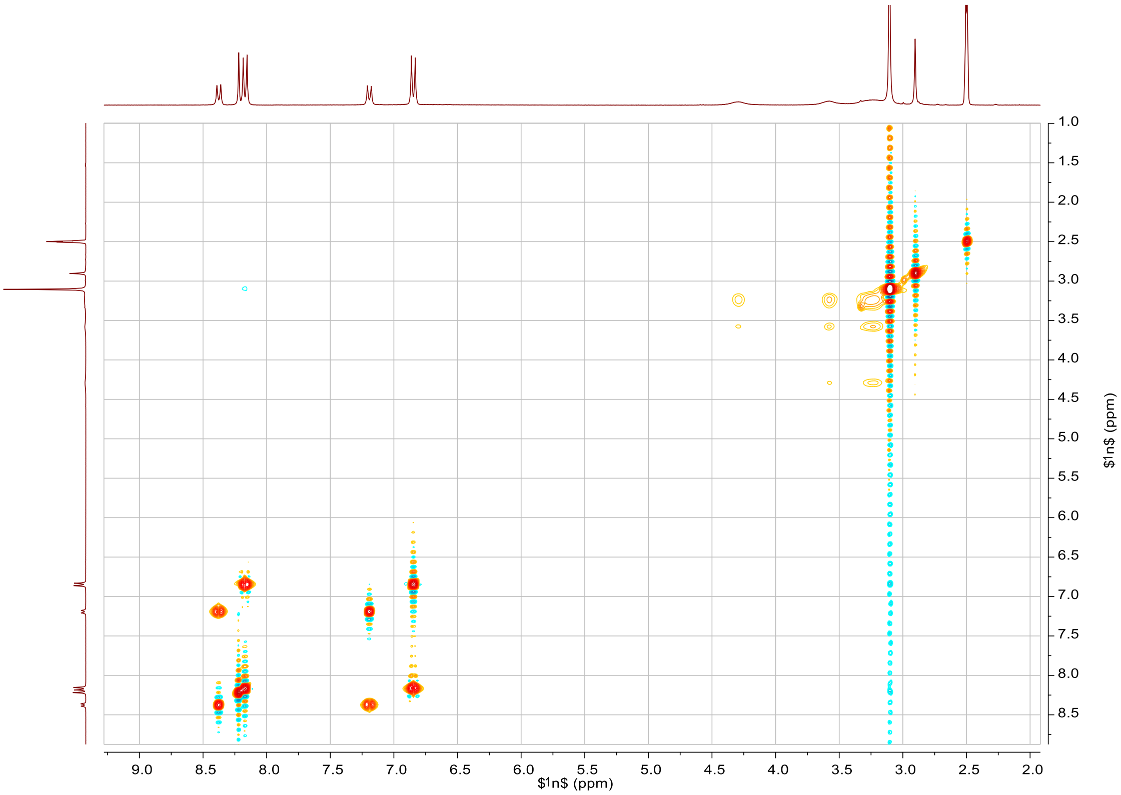


*Figure S46: TOCSY spectrum of 4-(4-(2,6-bis(4-(dimethylamino)phenyl)pyrylium-4-yl)phenyl)-1-methylpiperazin-1-ium bis(trifluoroacetate), measured at 293 K in DMSO-$d_6$*

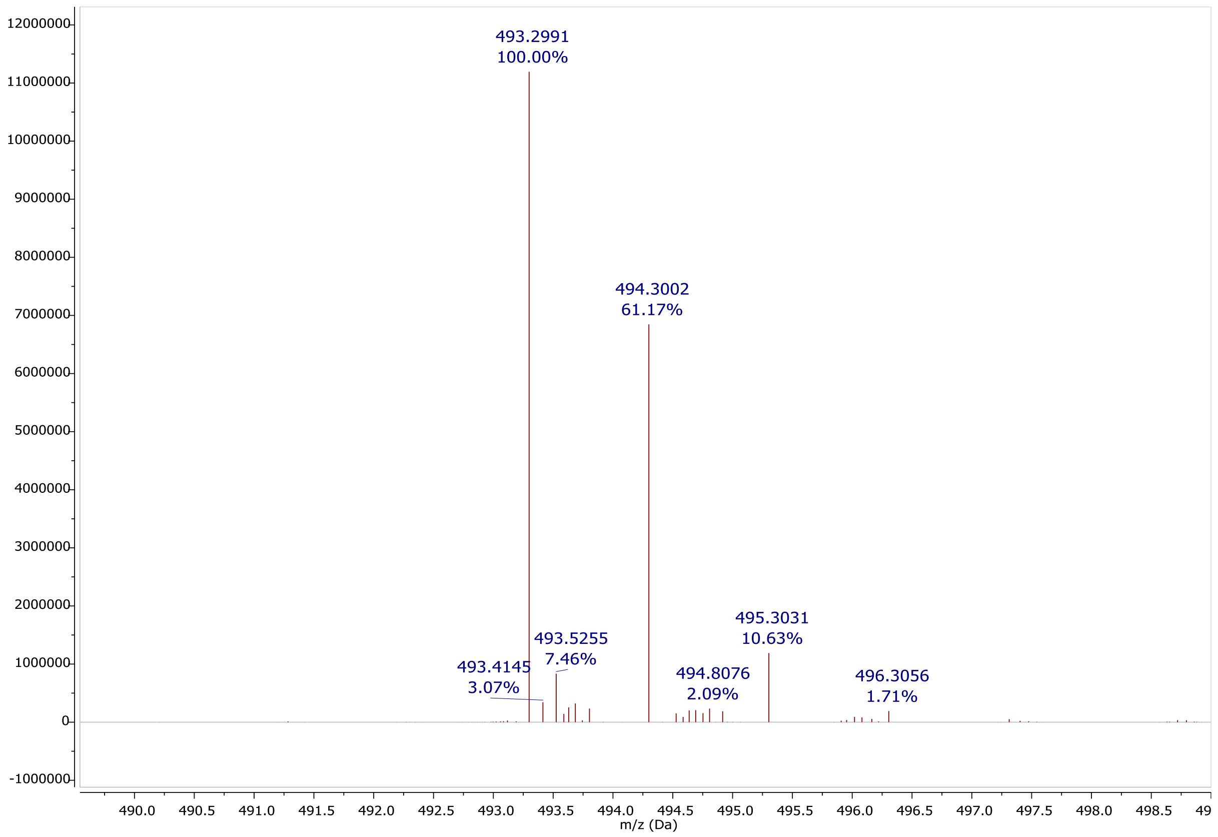


*Figure S47: HRMS spectrum of 4-(4-(2,6-bis(4-(dimethylamino)phenyl)pyrylium-4-yl)phenyl)-1-methylpiperazin-1-ium bis(trifluoroacetate)*

### 3.13. 2,4,6-NMP - 4,4',4''-(pyrylium-2,4,6-triyltris(benzene-4,1-diyl))tris(1-methylpiperazin-1-ium) tetrakis(trifluoroacetate)

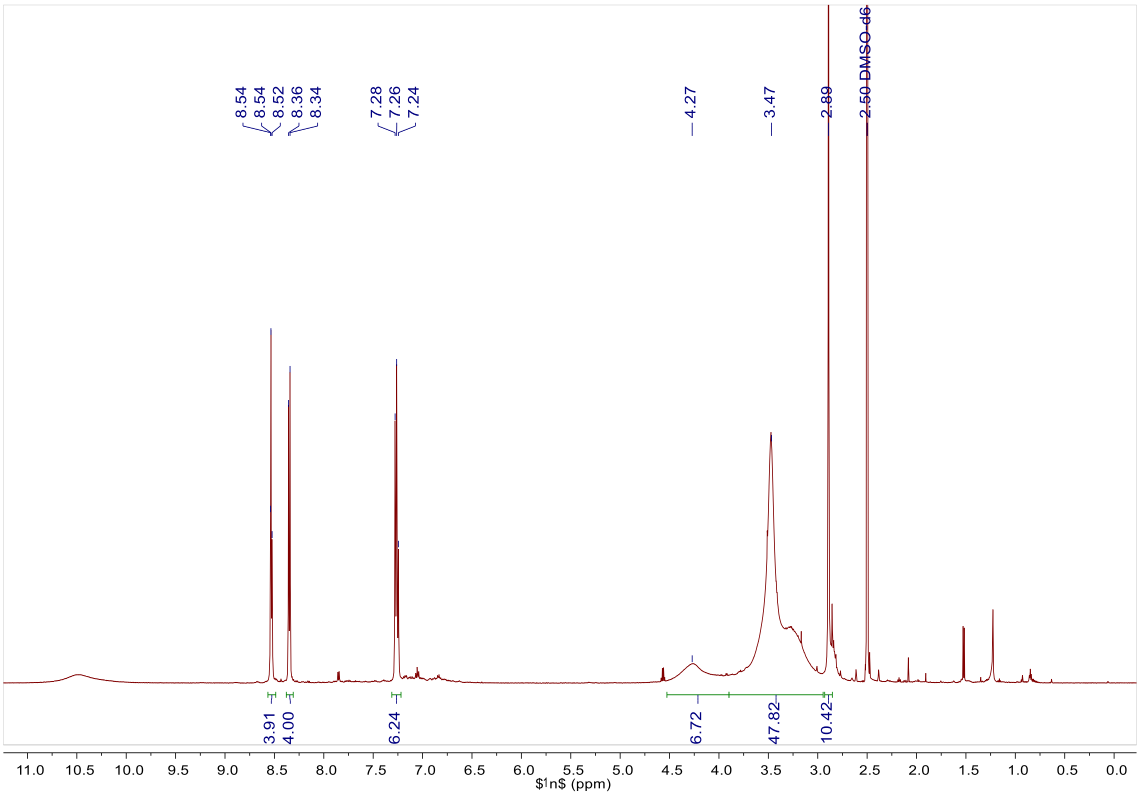


*Figure S48: $^{1}$H-NMR spectrum of 4,4',4''-(pyrylium-2,4,6-triyltris(benzene-4,1-diyl))tris(1-methylpiperazin-1-ium) tetrakis(trifluoroacetate), measured at 293 K in DMSO-$d_6$*

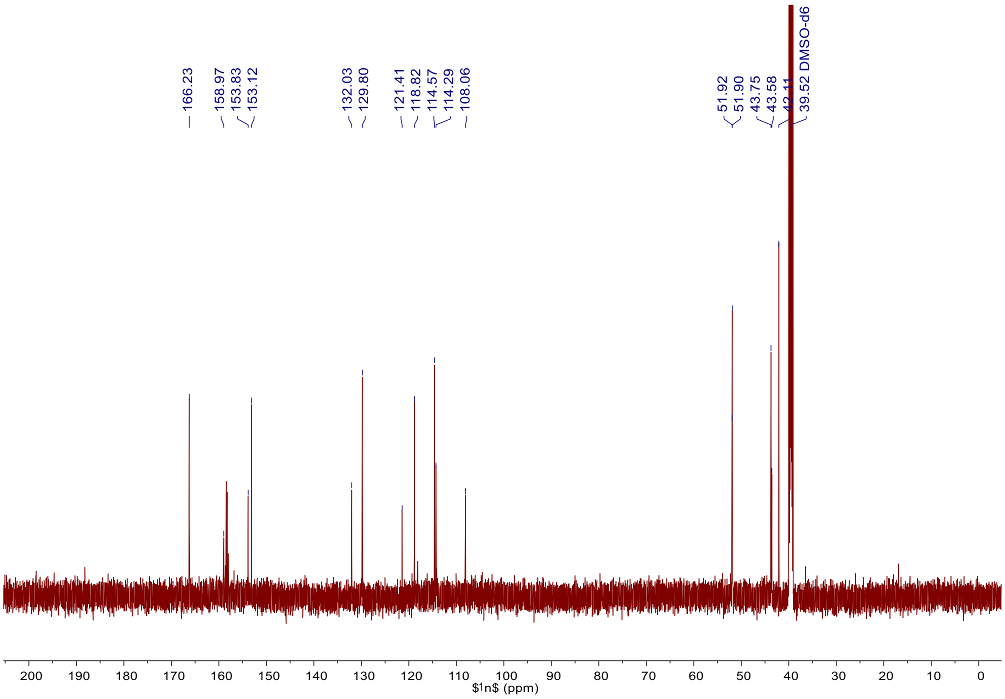


*Figure S49: $^{13}$C-NMR spectrum of 4,4',4''-(pyrylium-2,4,6-triyltris(benzene-4,1-diyl))tris(1-methylpiperazin-1-ium) tetrakis(trifluoroacetate), measured at 293 K in DMSO-$d_6$*

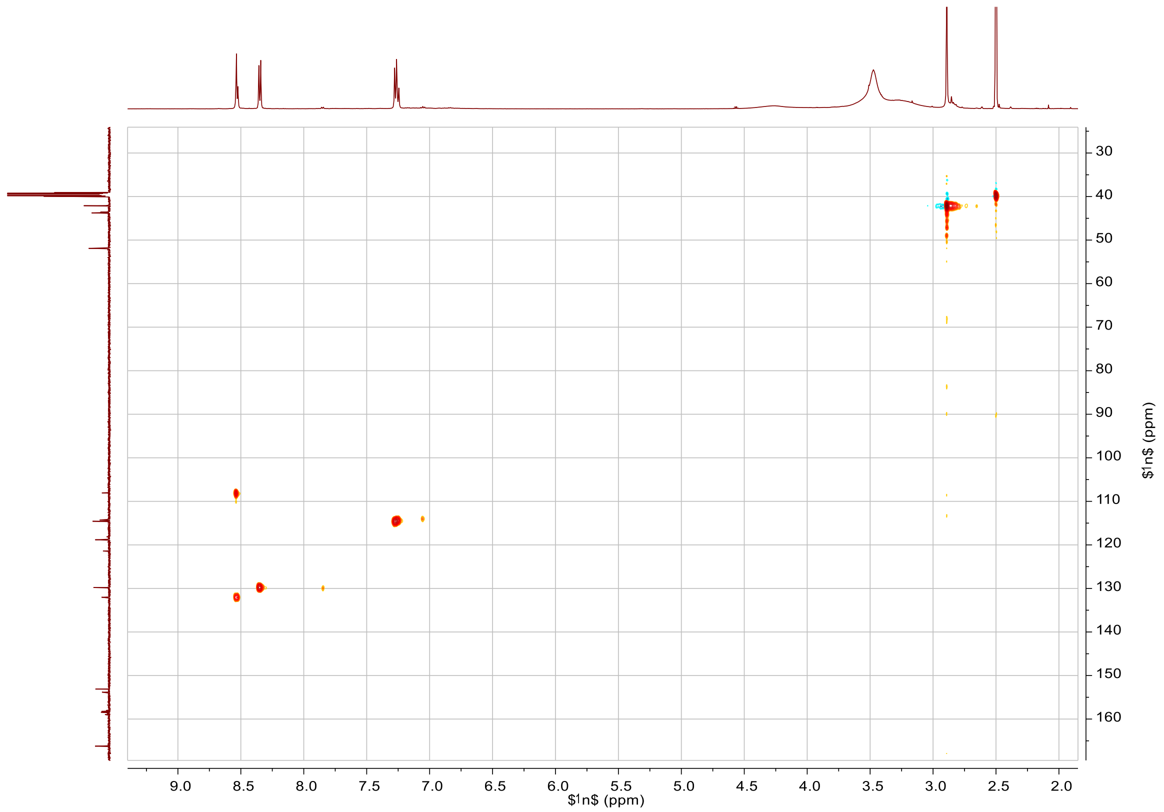


*Figure S50: HSQC spectrum of 4,4',4''-(pyrylium-2,4,6-triyltris(benzene-4,1-diyl))tris(1-methylpiperazin-1-ium) tetrakis(trifluoroacetate), measured at 293 K in DMSO-d*$_6$

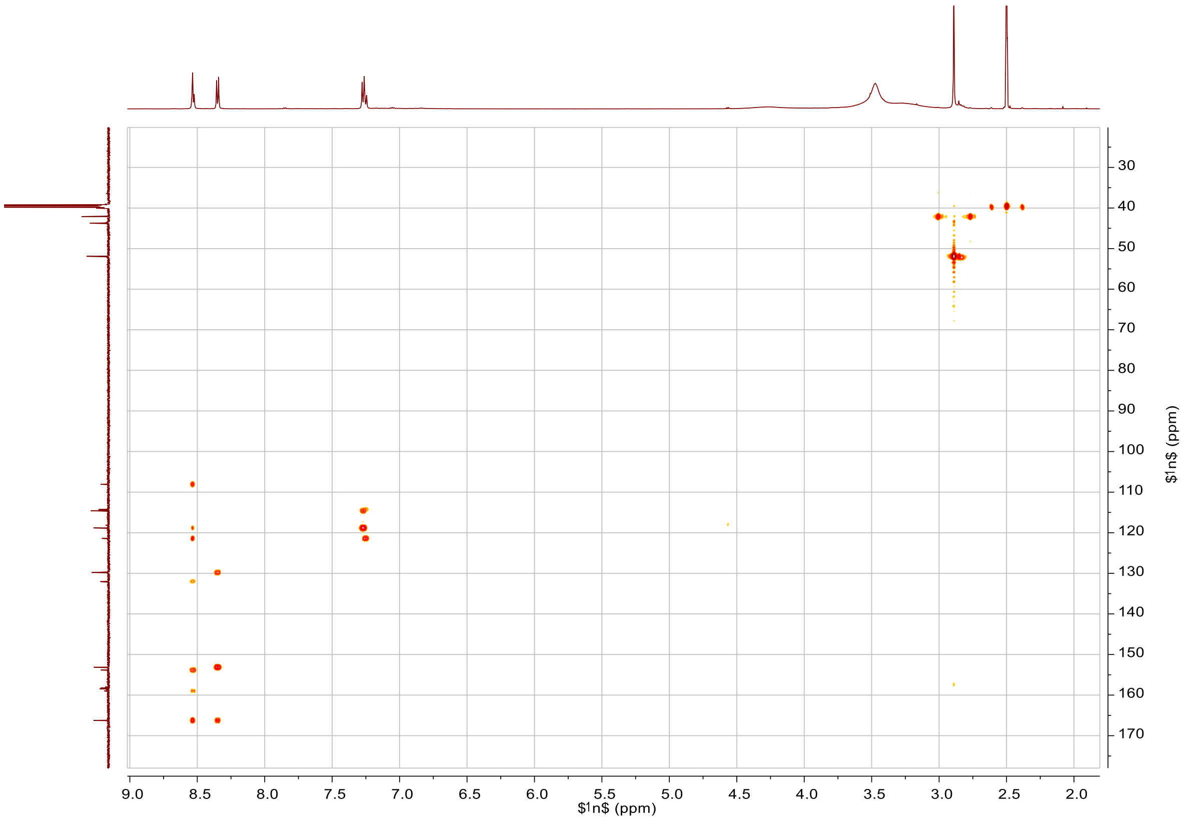


*Figure S51: HMBC spectrum of 4,4',4''-(pyrylium-2,4,6-triyltris(benzene-4,1-diyl))tris(1-methylpiperazin-1-ium) tetrakis(trifluoroacetate), measured at 293 K in DMSO-d*$_6$

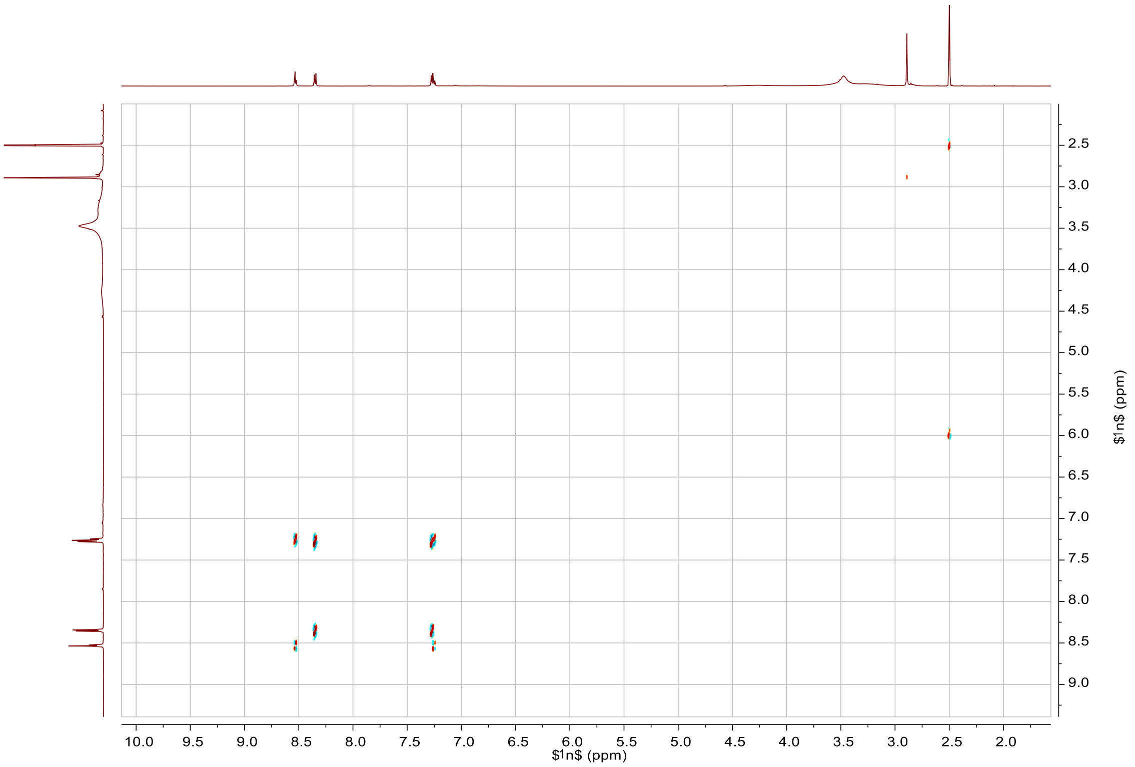


*Figure S52:COSY spectrum of 4,4',4''-(pyrylium-2,4,6-triyltris(benzene-4,1-diyl))tris(1-methylpiperazin-1-ium) tetrakis(trifluoroacetate), measured at 293 K in DMSO-$d_6$*

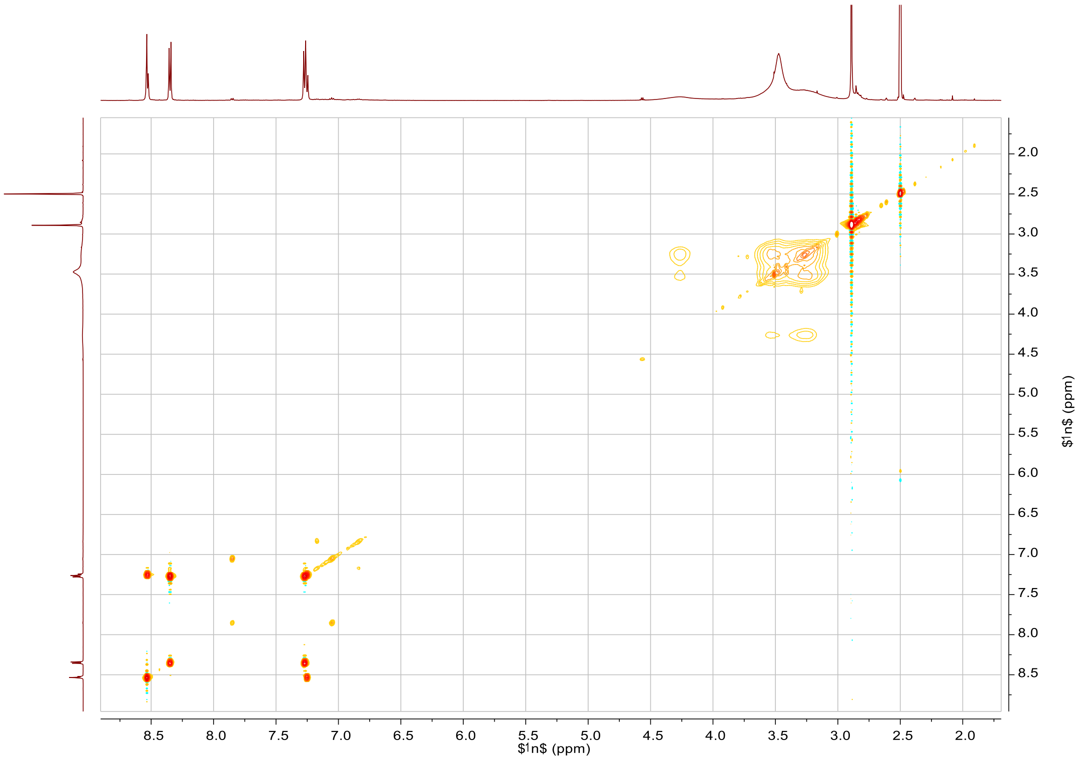


*Figure S53: TOCSY spectrum of 4,4',4''-(pyrylium-2,4,6-triyltris(benzene-4,1-diyl))tris(1-methylpiperazin-1-ium) tetrakis(trifluoroacetate), measured at 293 K in DMSO-$d_6$*

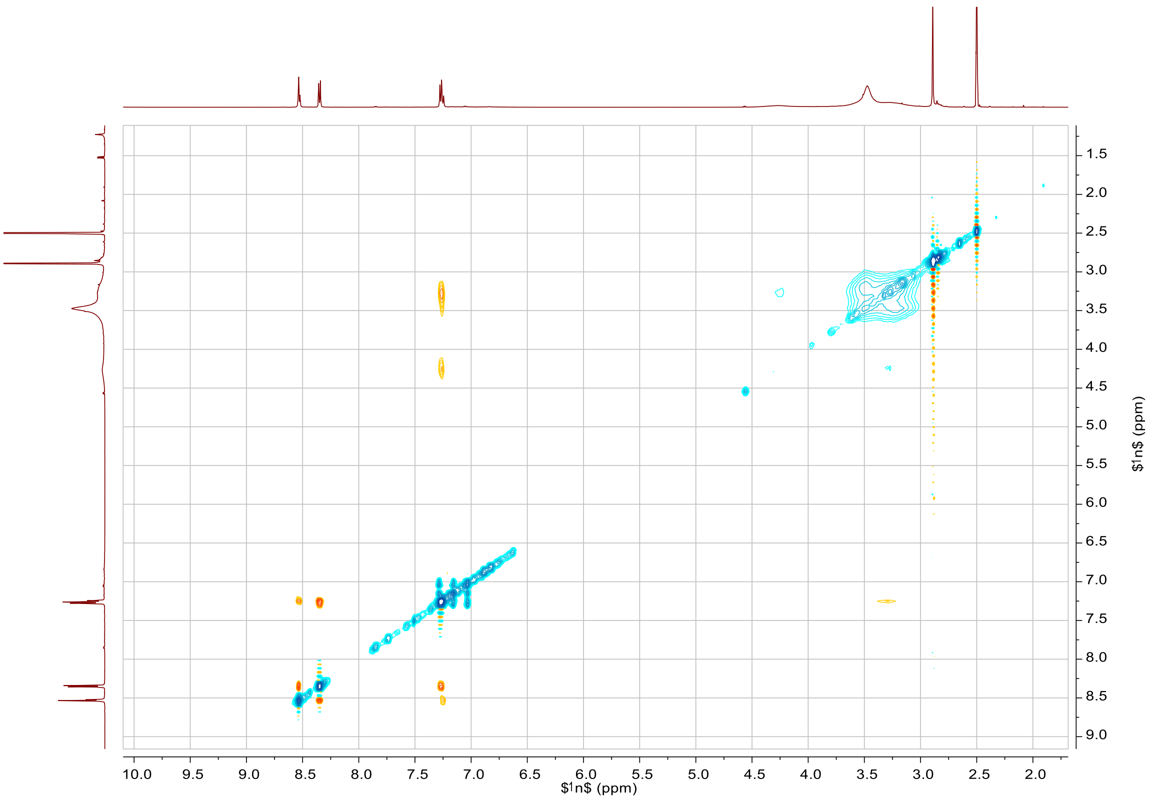


*Figure S54: ROESY spectrum of 4,4',4''-(pyrylium-2,4,6-triyltris(benzene-4,1-diyl))tris(1-methylpiperazin-1-ium) tetrakis(trifluoroacetate), measured at 293 K in DMSO-$d_6$*

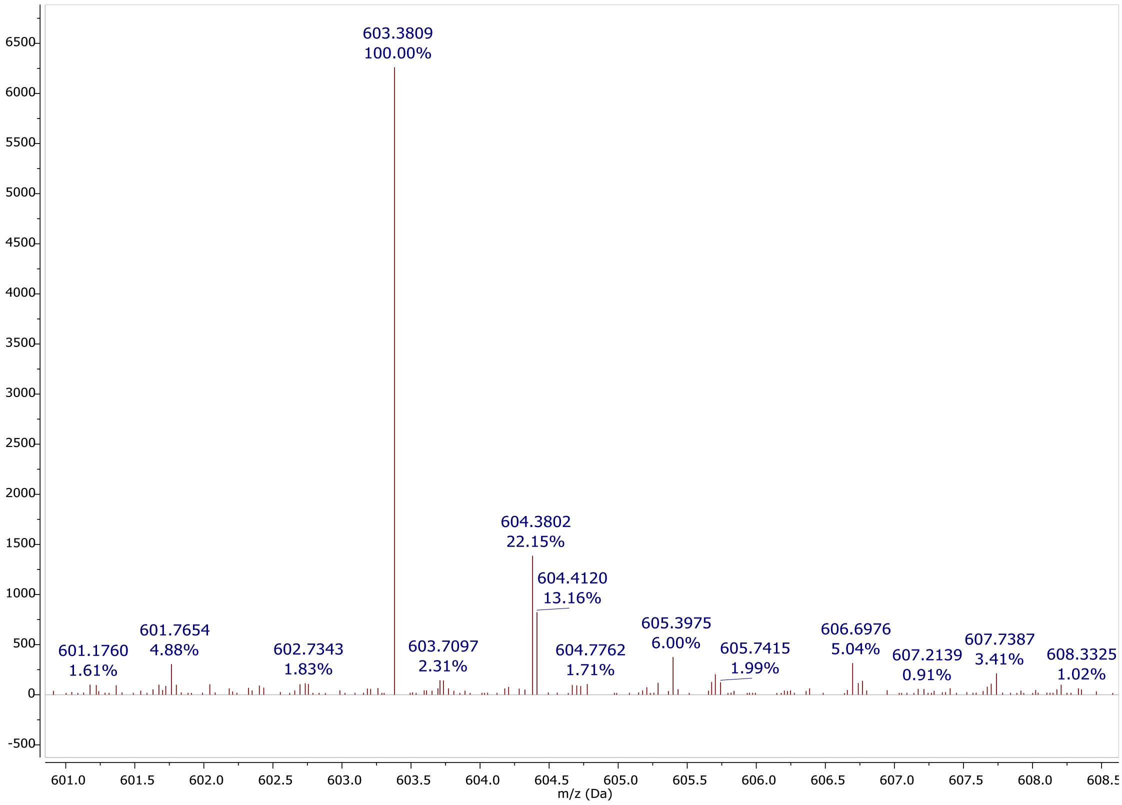


*Figure S55:- HRMS spectrum of 4,4',4''-(pyrylium-2,4,6-triyltris(benzene-4,1-diyl))tris(1-methylpiperazin-1-ium) tetrakis(trifluoroacetate)*

### 3.14. 2,6-NMP-4-Morph - 4,4'-((4-(4-morpholinophenyl)pyrylium-2,6-diyl)bis(4,1-phenylene))bis(1-methylpiperazin-1-ium) tris(trifluoroacetate)

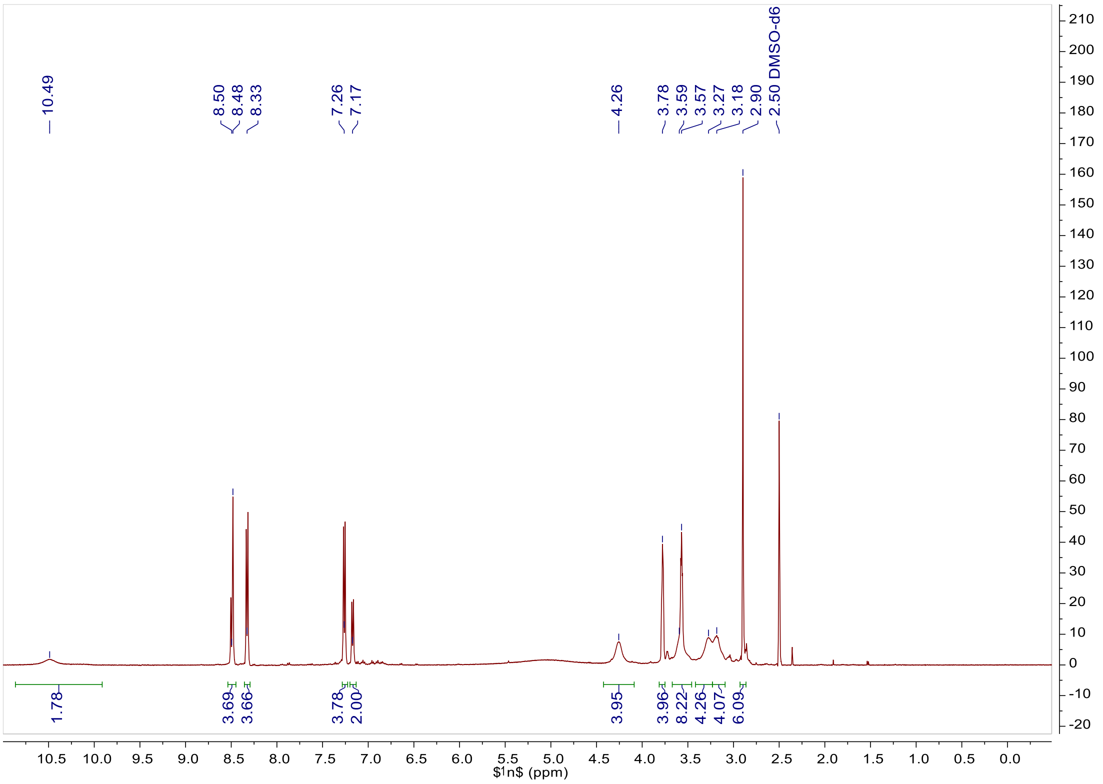


*Figure S56: $^{1}$H-NMR spectrum of 4,4'-((4-(4-morpholinophenyl)pyrylium-2,6-diyl)bis(4,1-phenylene))bis(1-methylpiperazin-1-ium) tris(trifluoroacetate), measured at 293 K in DMSO-$d_6$*

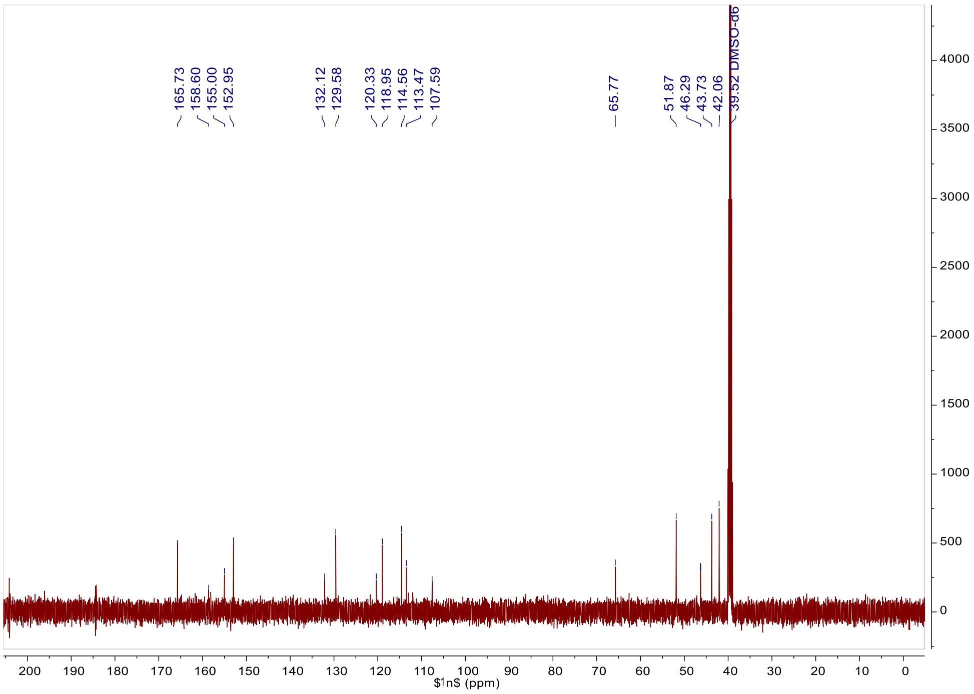


*Figure S57: $^{13}$C-NMR spectrum of 4,4'-((4-(4-morpholinophenyl)pyrylium-2,6-diyl)bis(4,1-phenylene))bis(1-methylpiperazin-1-ium) tris(trifluoroacetate), measured at 293 K in DMSO-$d_6$*

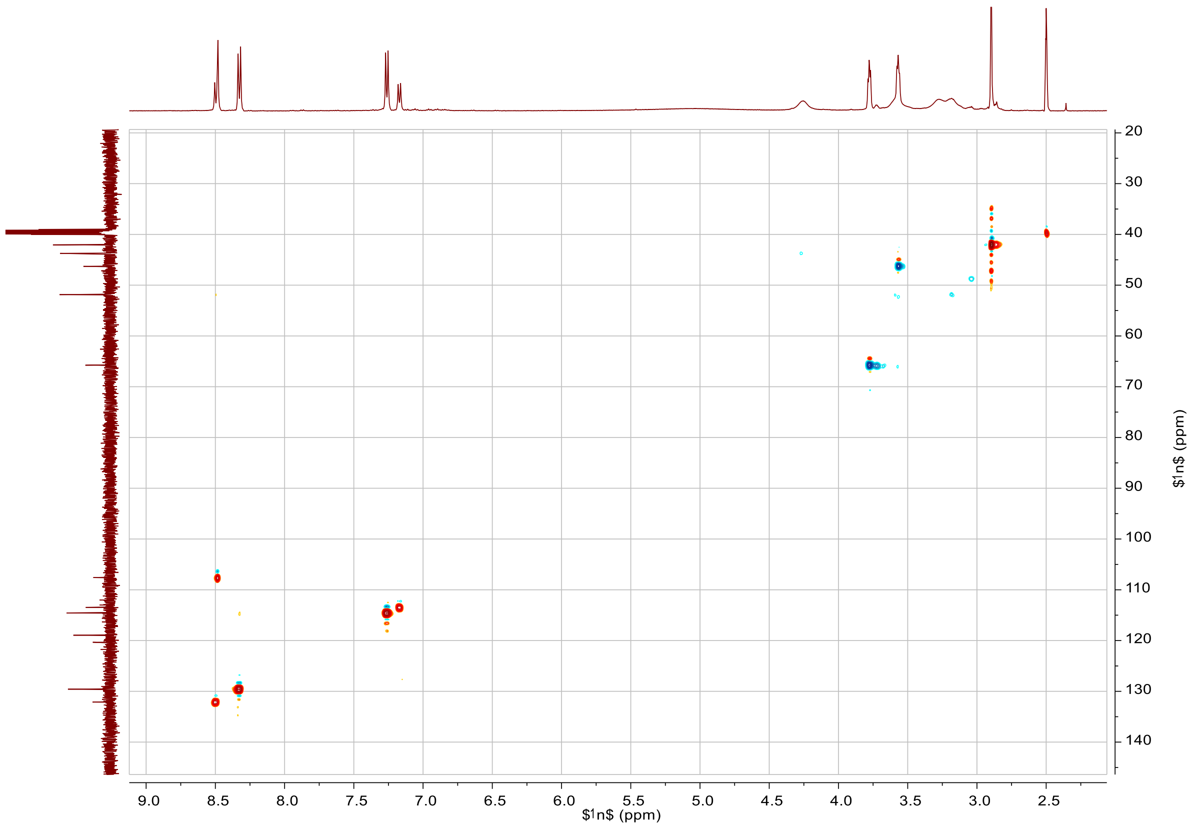


*Figure S58: HSQC spectrum of 4,4'-((4-(4-morpholinophenyl)pyrylium-2,6-diyl)bis(4,1-phenylene))bis(1-methylpiperazin-1-ium) tris(trifluoroacetate), measured at 293 K in DMSO-$d_6$*

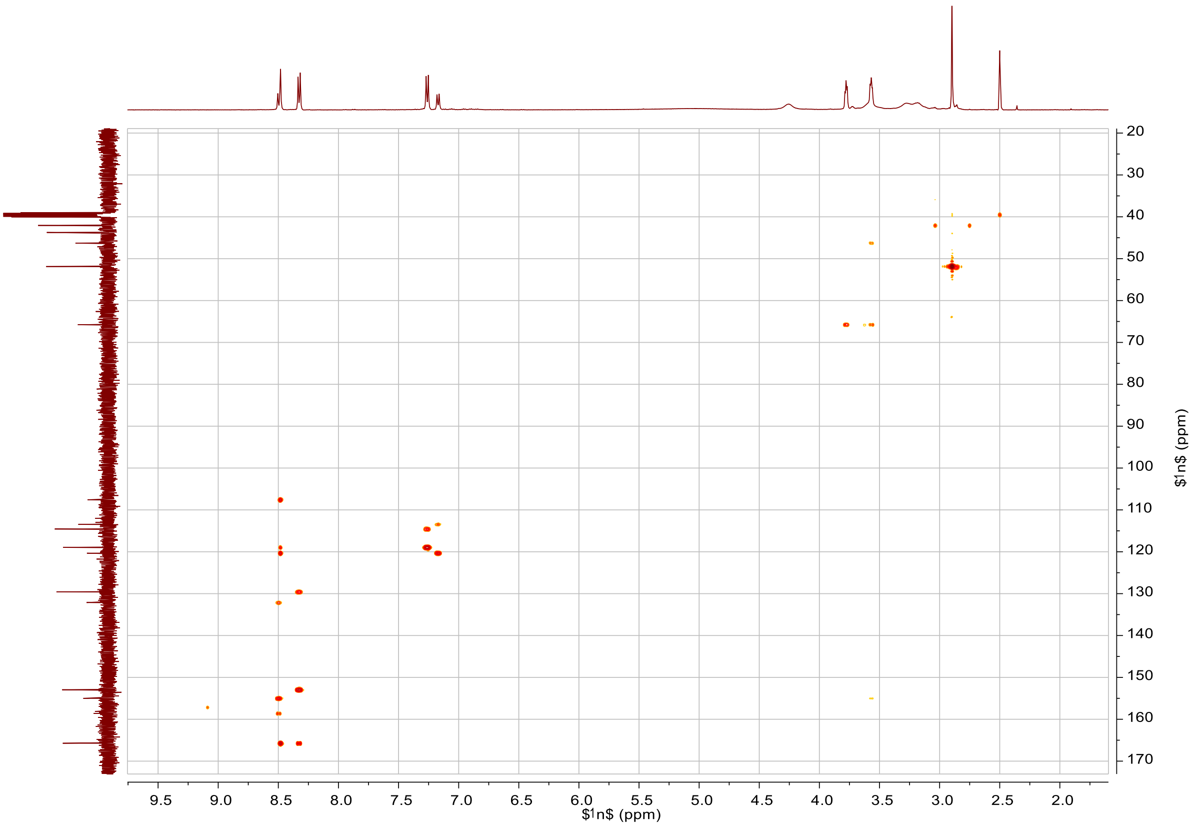


*Figure S59: HMBC spectrum of 4,4'-((4-(4-morpholinophenyl)pyrylium-2,6-diyl)bis(4,1-phenylene))bis(1-methylpiperazin-1-ium) tris(trifluoroacetate), measured at 293 K in DMSO-$d_6$*

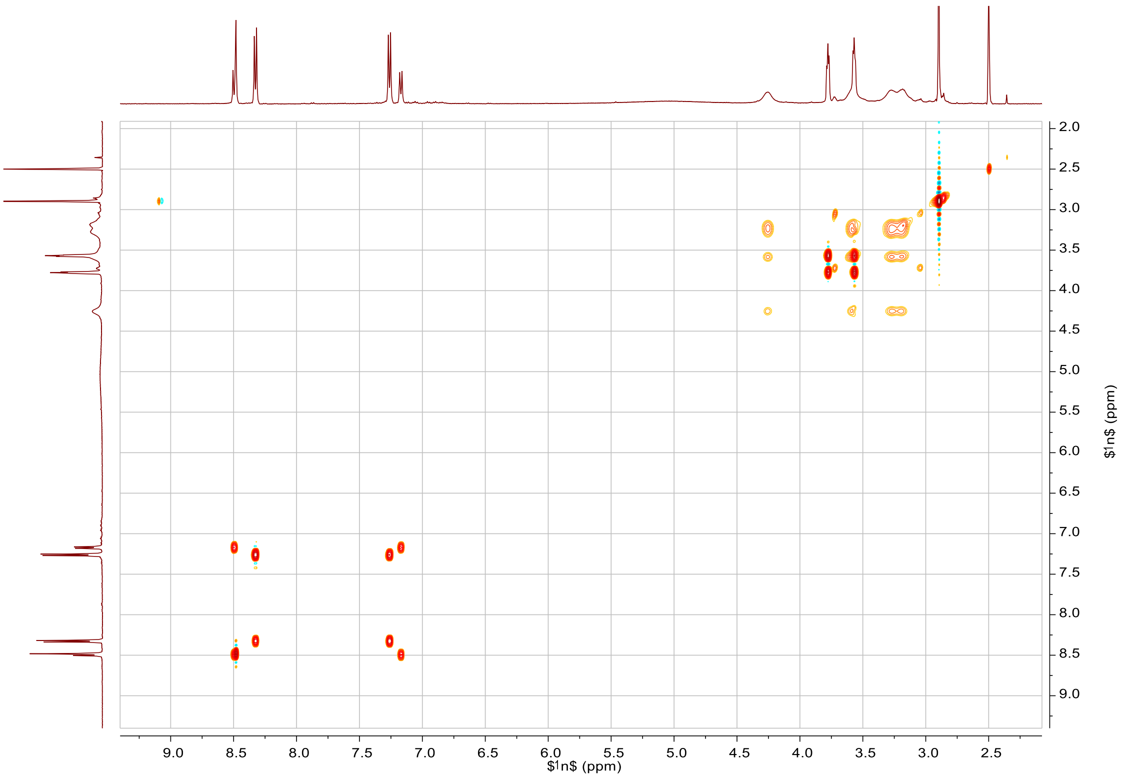


*Figure S60: TOCSY spectrum of 4,4'-((4-(4-morpholinophenyl)pyrylium-2,6-diyl)bis(4,1-phenylene))bis(1-methylpiperazin-1-ium) tris(trifluoroacetate), measured at 293 K in DMSO-d$_6$*

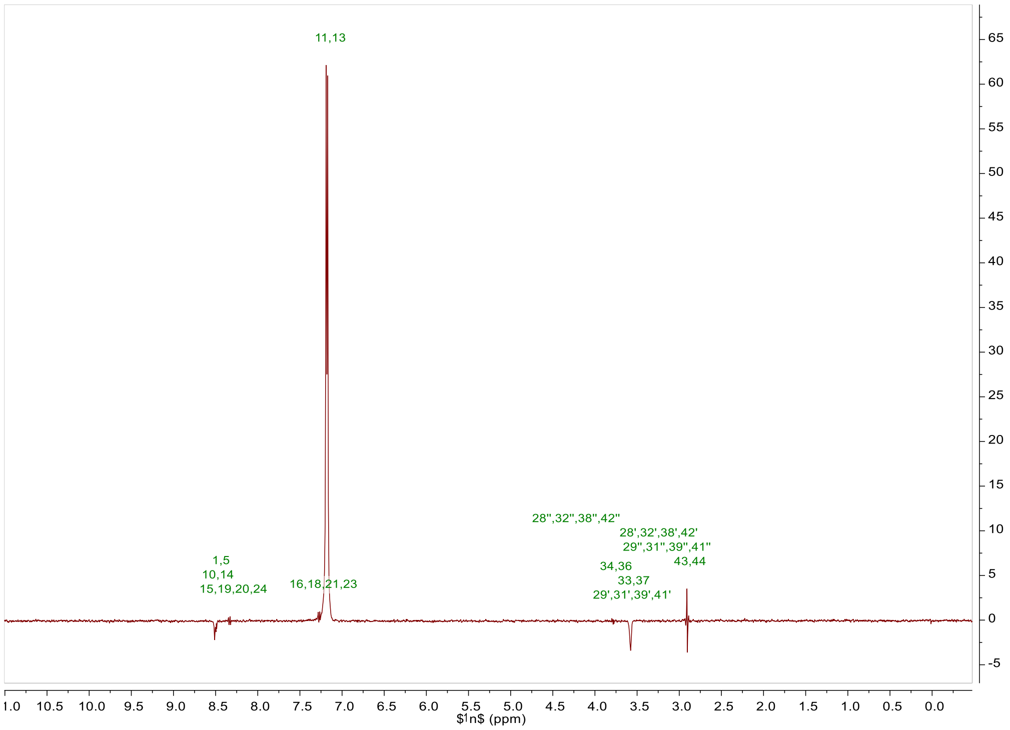


*Figure S61: 1D-ROESY spectrum of 4,4'-((4-(4-morpholinophenyl)pyrylium-2,6-diyl)bis(4,1-phenylene))bis(1-methylpiperazin-1-ium) tris(trifluoroacetate), 7.18 ppm (width: 30.4 Hz), measured at 293 K in DMSO-d$_6$*

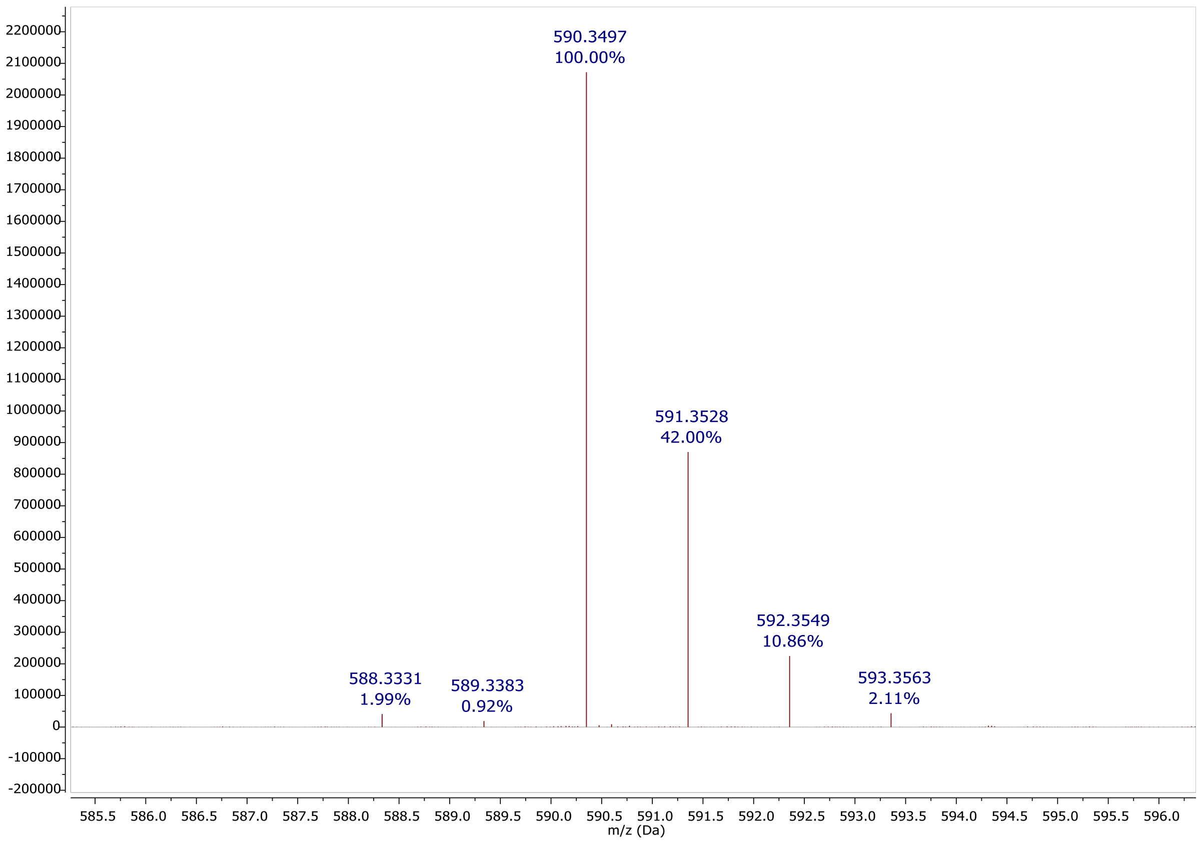


*Figure S62: HRMS spectrum of 4,4'-((4-(4-morpholinophenyl)pyrylium-2,6-diyl)bis(4,1-phenylene))bis(1-methylpiperazin-1-ium) tris(trifluoroacetate)*

### 3.15. 2,6-Ind-4-DMA - 4-(4-(dimethylamino)phenyl)-2,6-bis(1-(2-(dimethylammonio)ethyl)indolin-5-yl)pyrylium tris(trifluoroacetate)

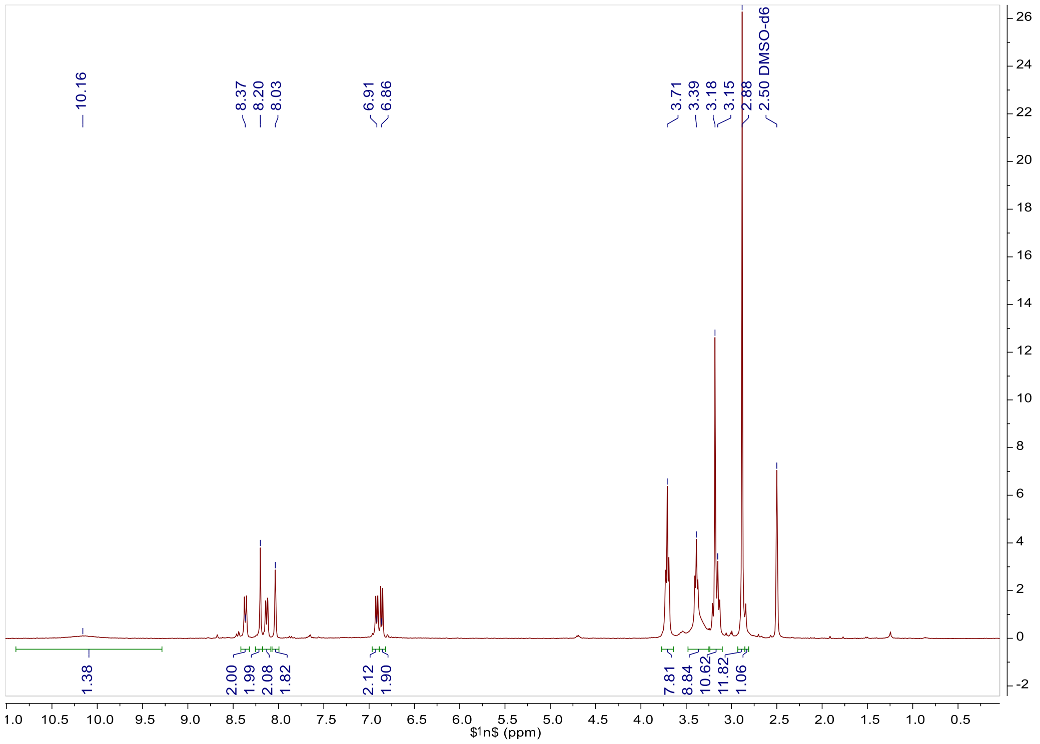


*Figure S63: $^{1}$H-NMR spectrum of 4-(4-(dimethylamino)phenyl)-2,6-bis(1-(2-(dimethylammonio)ethyl)indolin-5-yl)pyrylium tris(trifluoroacetate), 400 MHz, measured at 293 K in DMSO-$d_6$*

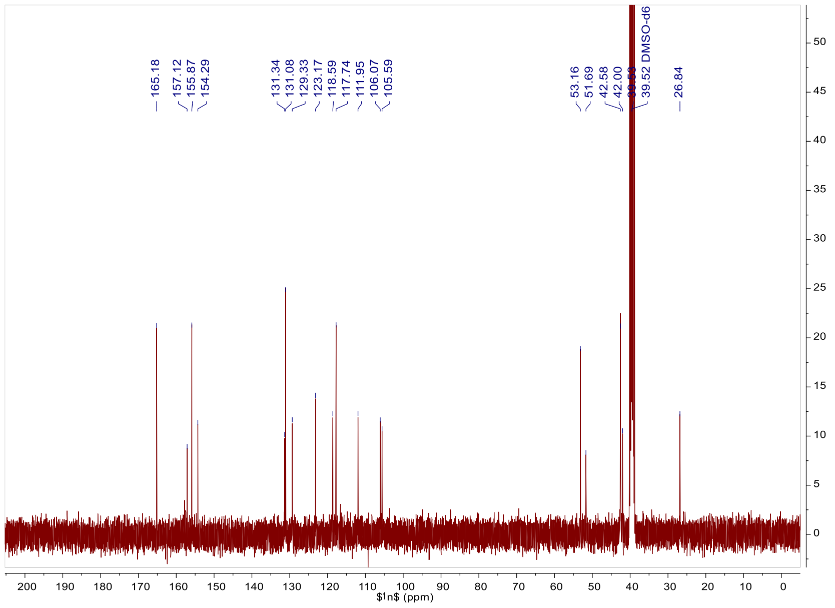


*Figure S64: $^{13}$C-NMR spectrum of 4-(4-(dimethylamino)phenyl)-2,6-bis(1-(2-(dimethylammonio)ethyl)indolin-5-yl)pyrylium tris(trifluoroacetate), 101 MHz, measured at 293 K in DMSO-$d_6$*

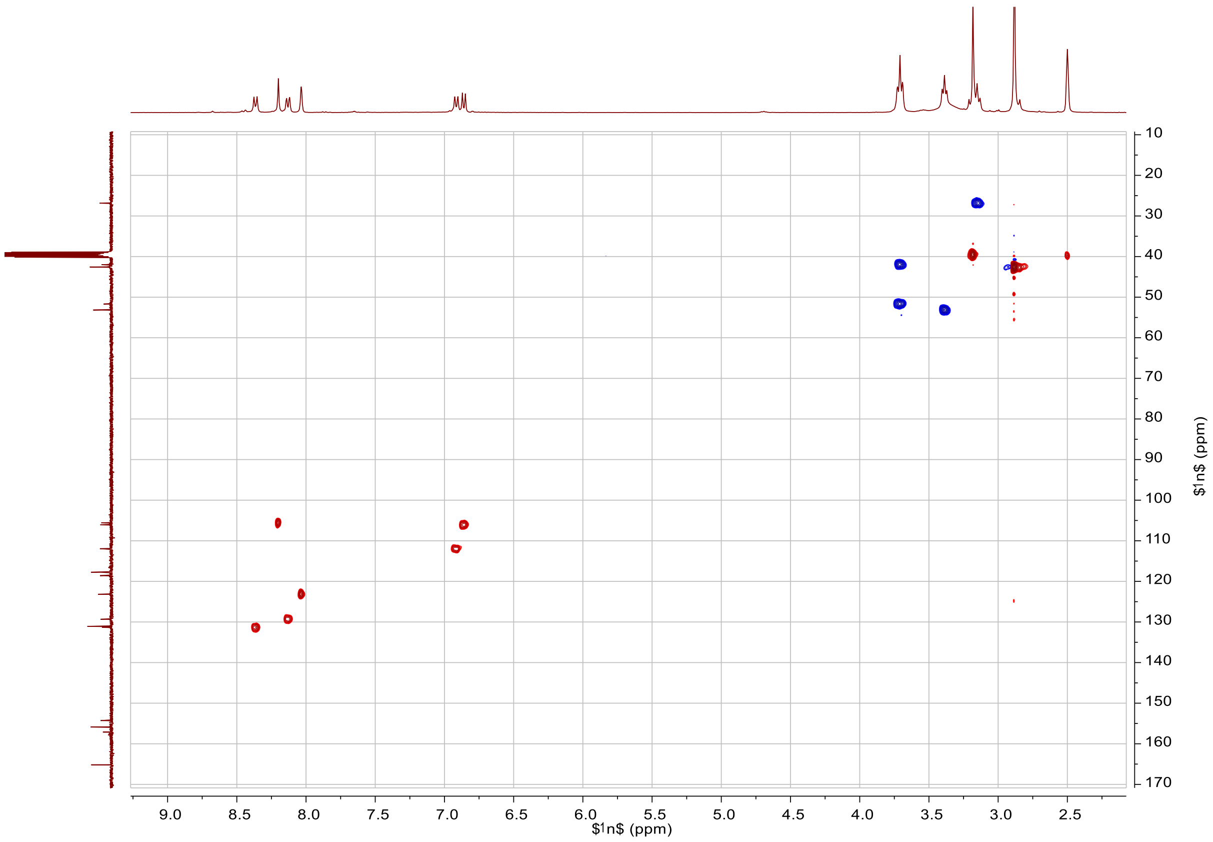


*Figure S65: HSQC spectrum of 4-(4-(dimethylamino)phenyl)-2,6-bis(1-(2-(dimethylammonio)ethyl)indolin-5-yl)pyrylium tris(trifluoroacetate), measured at 293 K in DMSO-$d_6$*

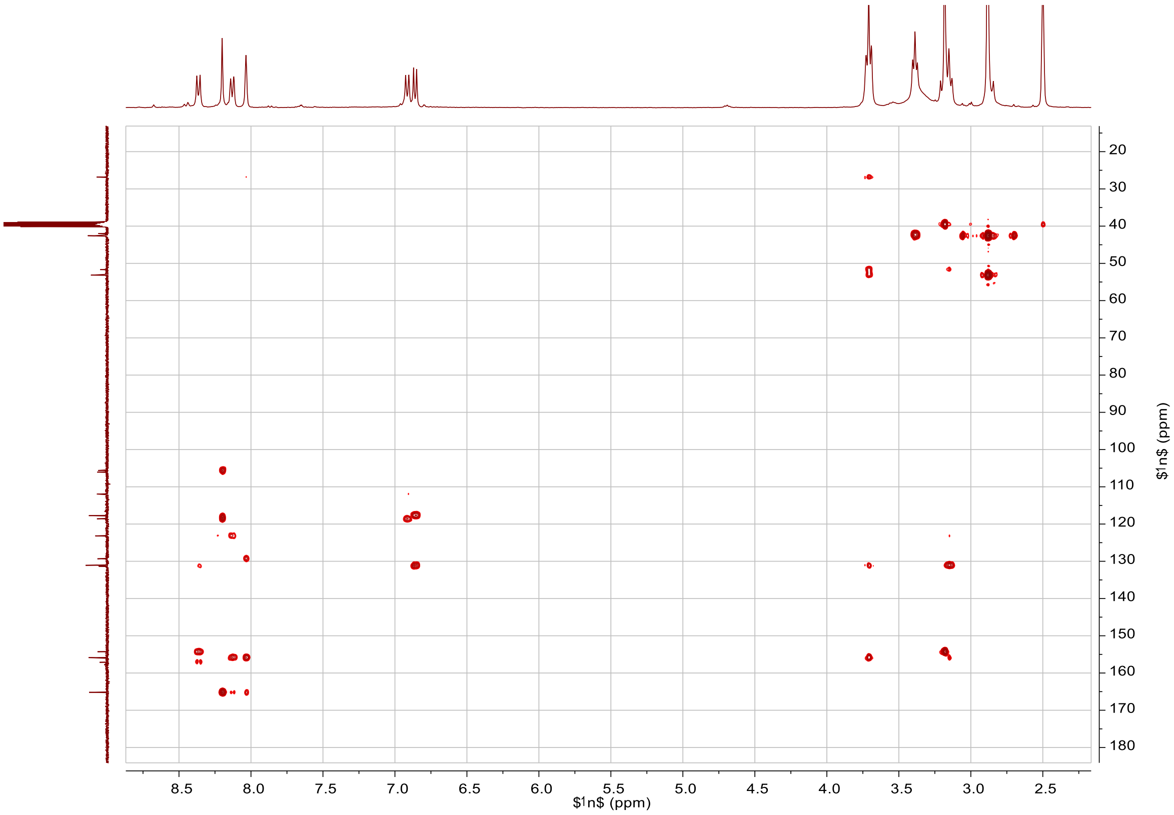


*Figure S66: HMBC spectrum of 4-(4-(dimethylamino)phenyl)-2,6-bis(1-(2-(dimethylammonio)ethyl)indolin-5-yl)pyrylium tris(trifluoroacetate), measured at 293 K in DMSO-$d_6$*

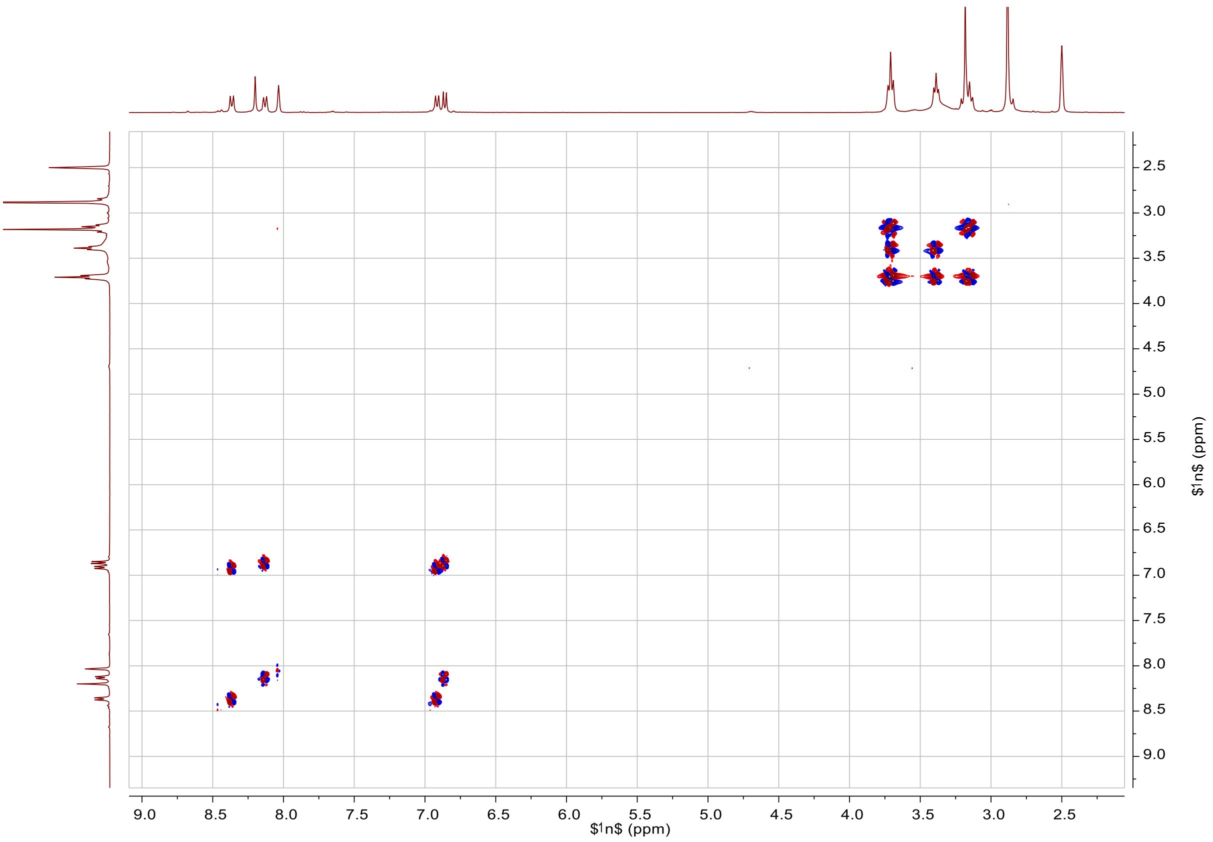


*Figure S67: COSY spectrum of 4-(4-(dimethylamino)phenyl)-2,6-bis(1-(2-(dimethylammonio)ethyl)indolin-5-yl)pyrylium tris(trifluoroacetate), measured at 293 K in DMSO-$d_6$*

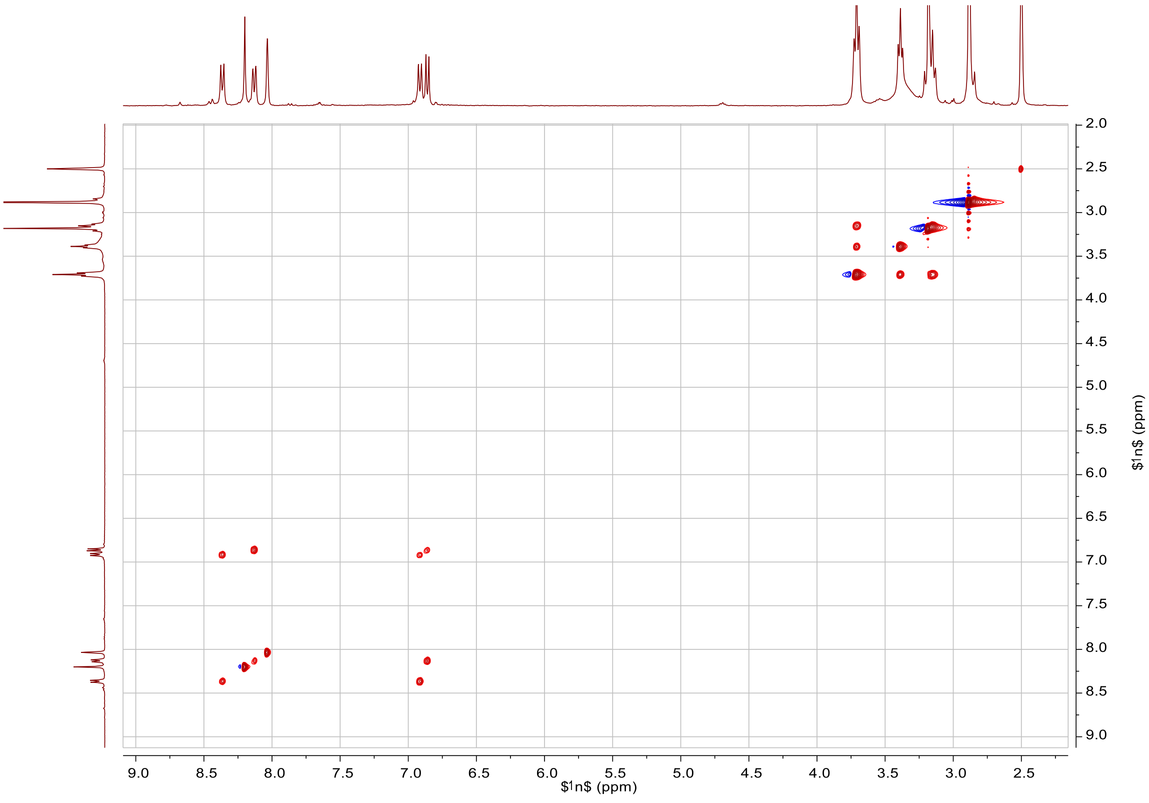


*Figure S68: TOCSY spectrum of 4-(4-(dimethylamino)phenyl)-2,6-bis(1-(2-(dimethylammonio)ethyl)indolin-5-yl)pyrylium tris(trifluoroacetate), measured at 293 K in DMSO-$d_6$*

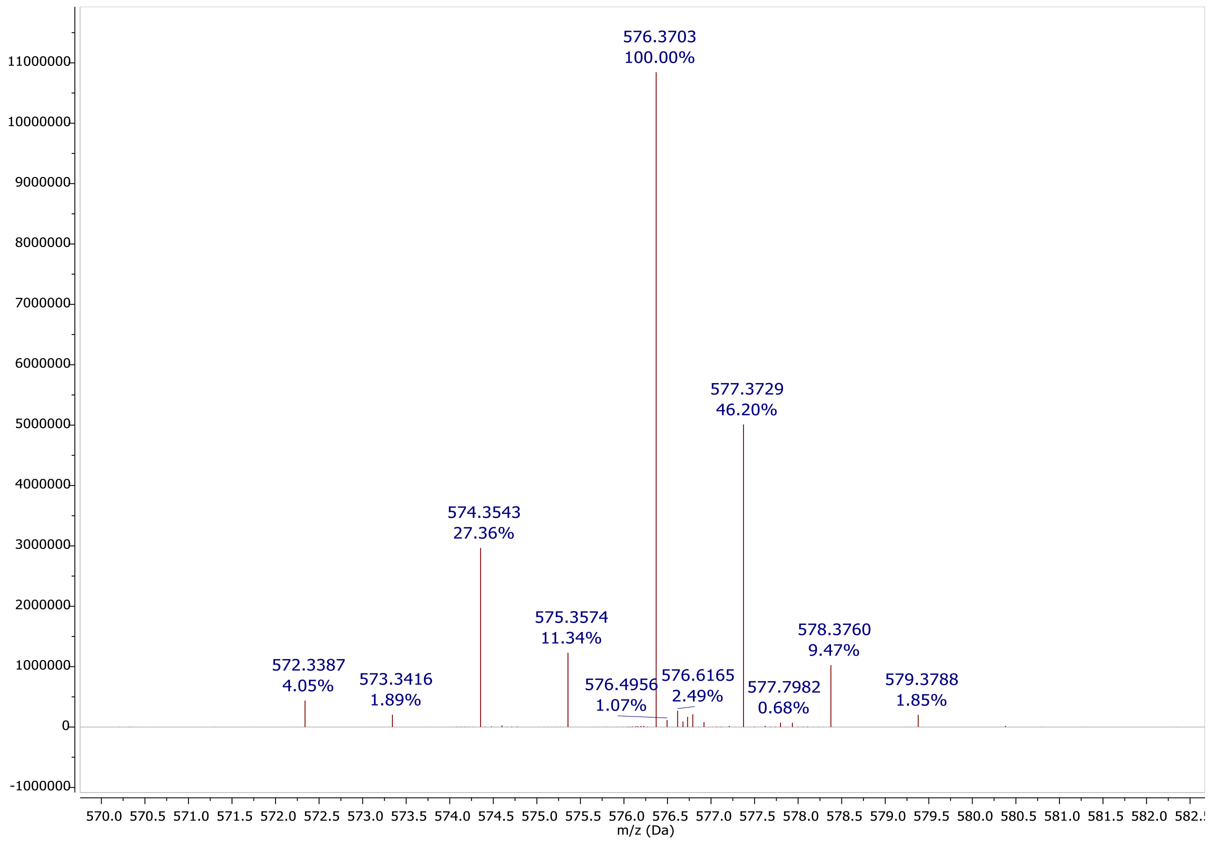


*Figure S69: HRMS spectrum of 4-(4-(dimethylamino)phenyl)-2,6-bis(1-(2-(dimethylammonio)ethyl)indolin-5-yl)pyrylium tris(trifluoroacetate)*

## 3.16. 2,6-NdMP-4-DMA 4,4'-((4-(4-(dimethylamino)phenyl)pyrylium-2,6-diyl)bis(4,1-phenylene))bis(1,1-dimethylpiperazin-1-ium) tris(trifluoroacetate)

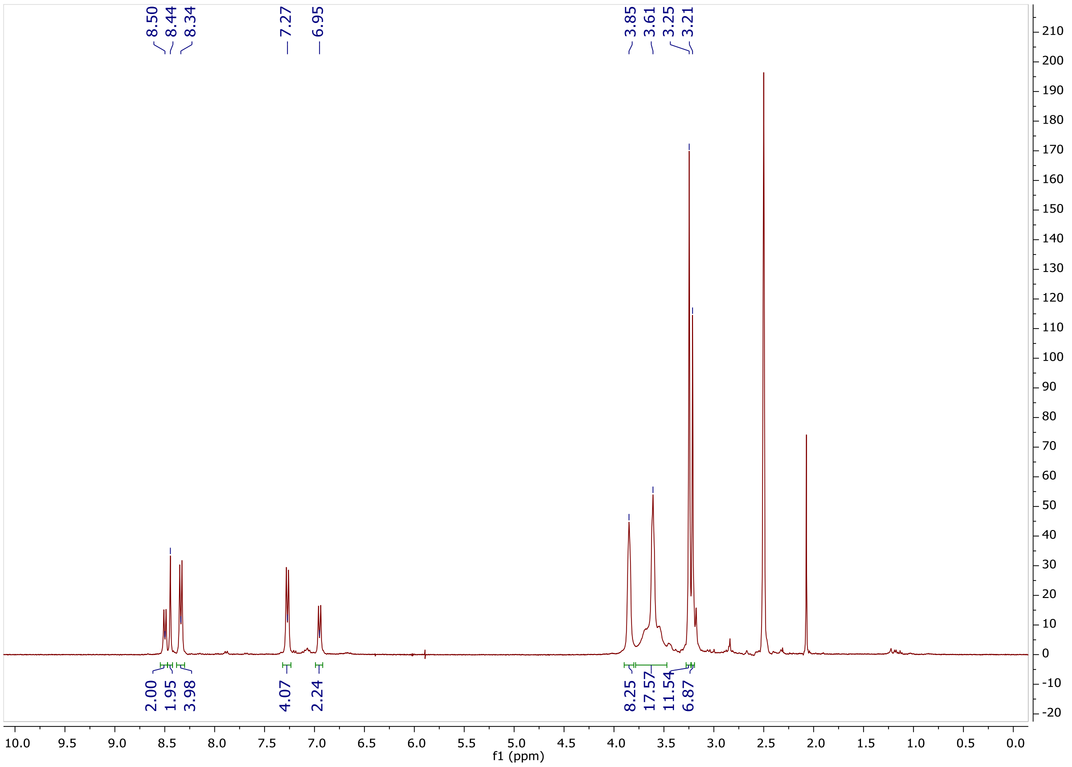


*Figure S70: $^{1}$H-NMR spectrum of 4,4'-((4-(4-(dimethylamino)phenyl)pyrylium-2,6-diyl)bis(4,1-phenylene))bis(1,1-dimethylpiperazin-1-ium) tris(trifluoroacetate), measured at 293 K in DMSO-$d_6$*

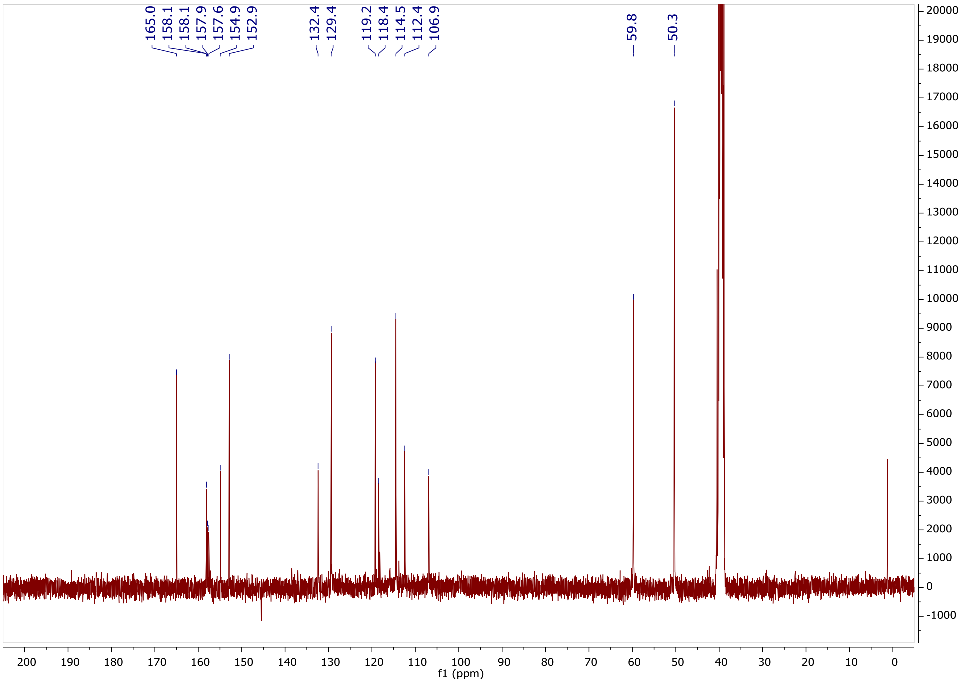


*Figure S71: $^{13}$C-NMR spectrum of 4,4'-((4-(4-(dimethylamino)phenyl)pyrylium-2,6-diyl)bis(4,1-phenylene))bis(1,1-dimethylpiperazin-1-ium) tris(trifluoroacetate), measured at 293 K in DMSO-$d_6$*

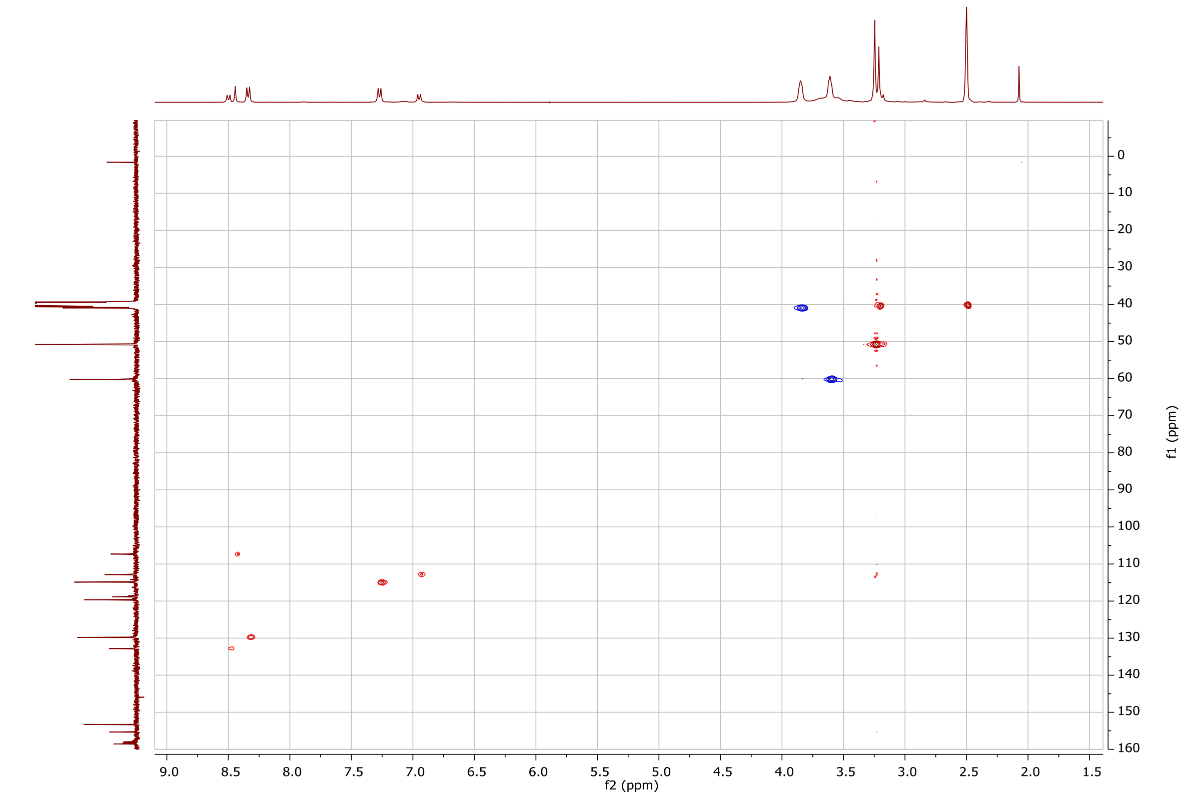


*Figure S72: HSQC spectrum of 4,4'-((4-(4-(dimethylamino)phenyl)pyrylium-2,6-diyl)bis(4,1-phenylene))bis(1,1-dimethylpiperazin-1-ium) tris(trifluoroacetate), measured at 293 K in DMSO-$d_6$*

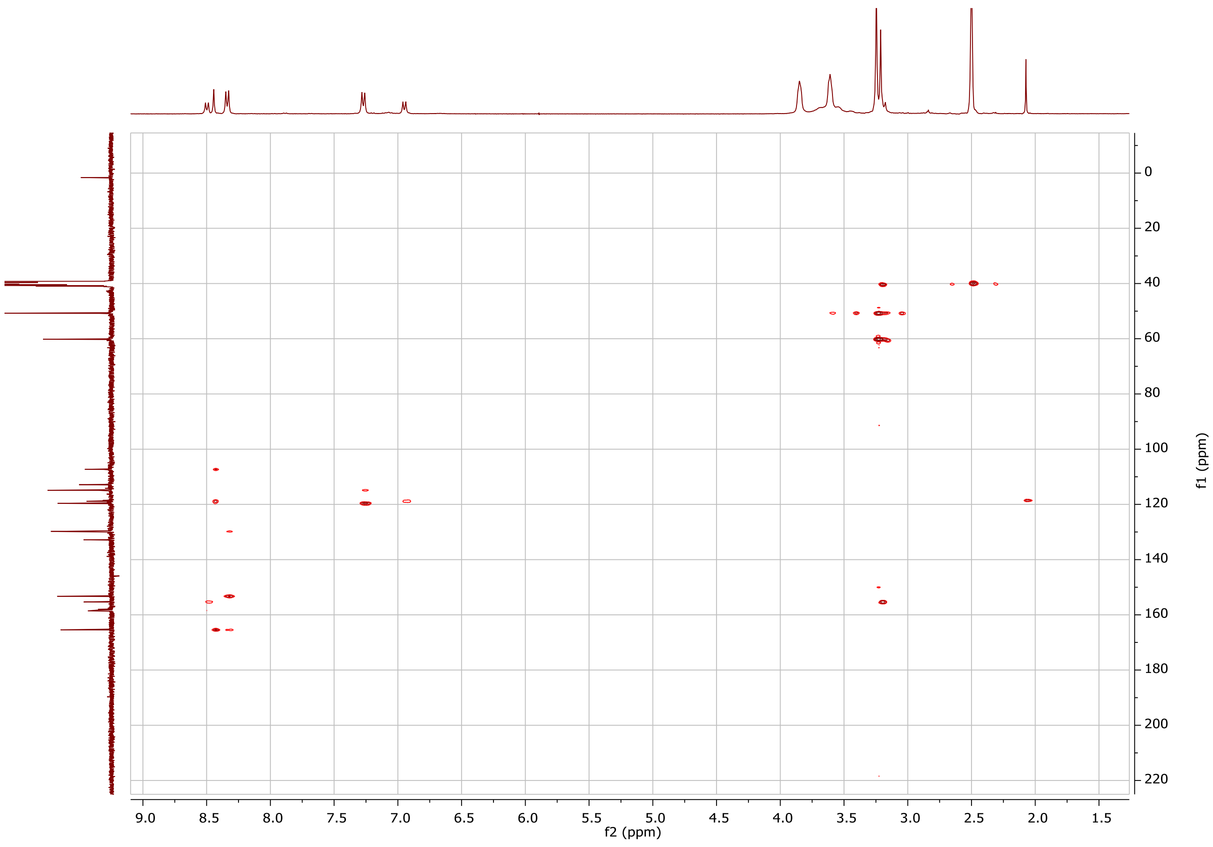


*Figure S73: HMBC spectrum of 4,4'-((4-(4-(dimethylamino)phenyl)pyrylium-2,6-diyl)bis(4,1-phenylene))bis(1,1-dimethylpiperazin-1-ium) tris(trifluoroacetate), measured at 293 K in DMSO-$d_6$*

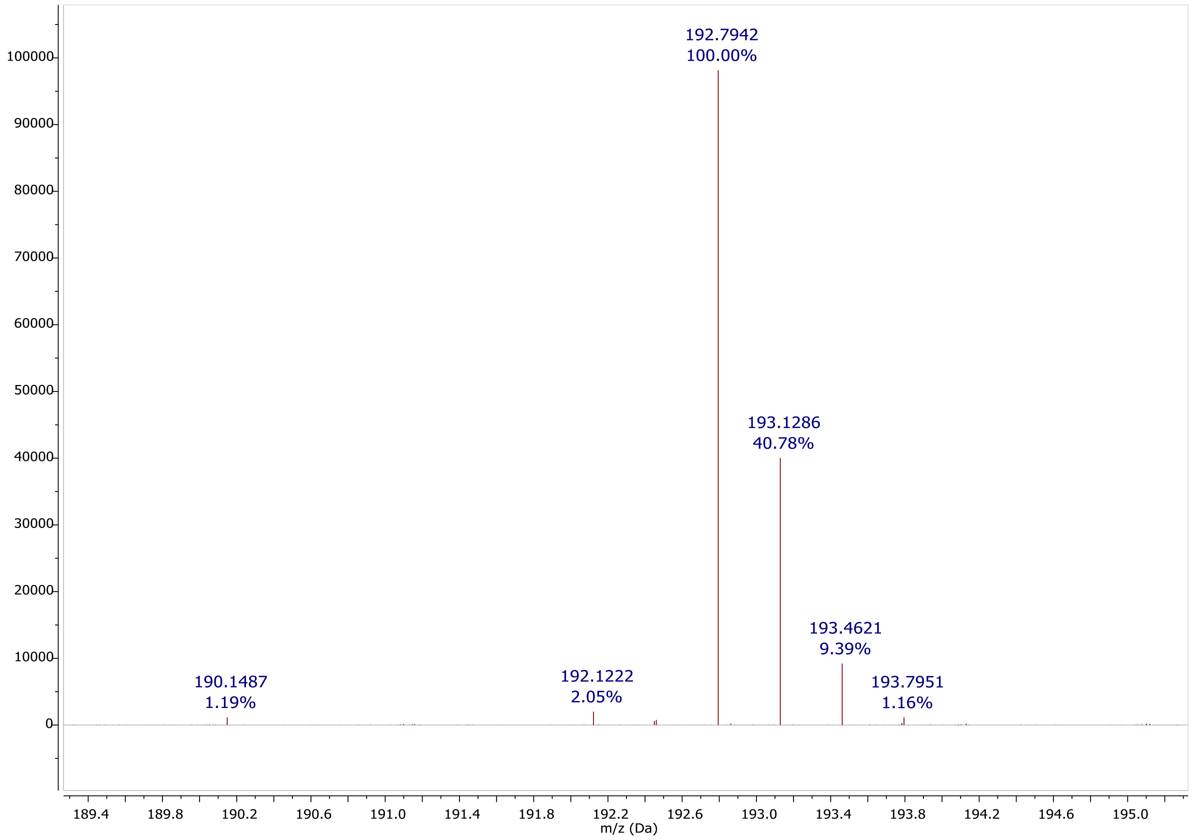


*Figure S74: HRMS spectrum of 4,4'-((4-(4-(dimethylamino)phenyl)pyrylium-2,6-diyl)bis(4,1-phenylene))bis(1,1-dimethylpiperazin-1-ium) tris(trifluoroacetate)*

### 3.17. 2,6-NMP-4-OMe - 4,4'-((4-(4-methoxyphenyl)pyrylium-2,6-diyl)bis(4,1-phenylene))bis(1-methylpiperazin-1-ium) tris(trifluoroacetate)

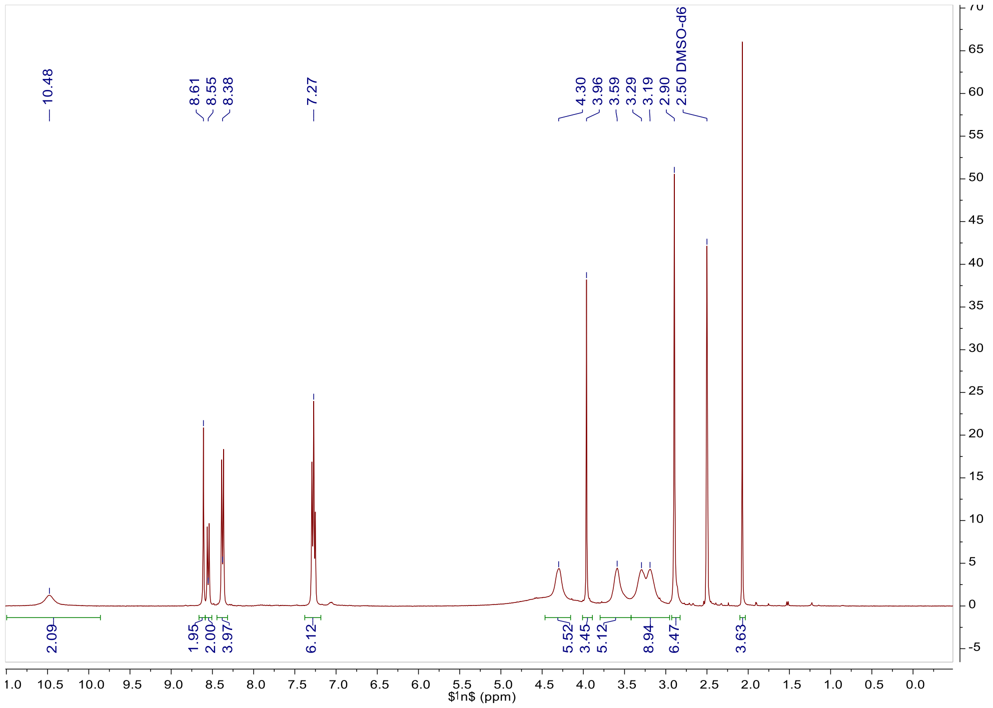


*Figure S75: $^{1}$H-NMR spectrum of 4,4'-((4-(4-methoxyphenyl)pyrylium-2,6-diyl)bis(4,1-phenylene))bis(1-methylpiperazin-1-ium) tris(trifluoroacetate), measured at 293 K in DMSO-$d_6$*

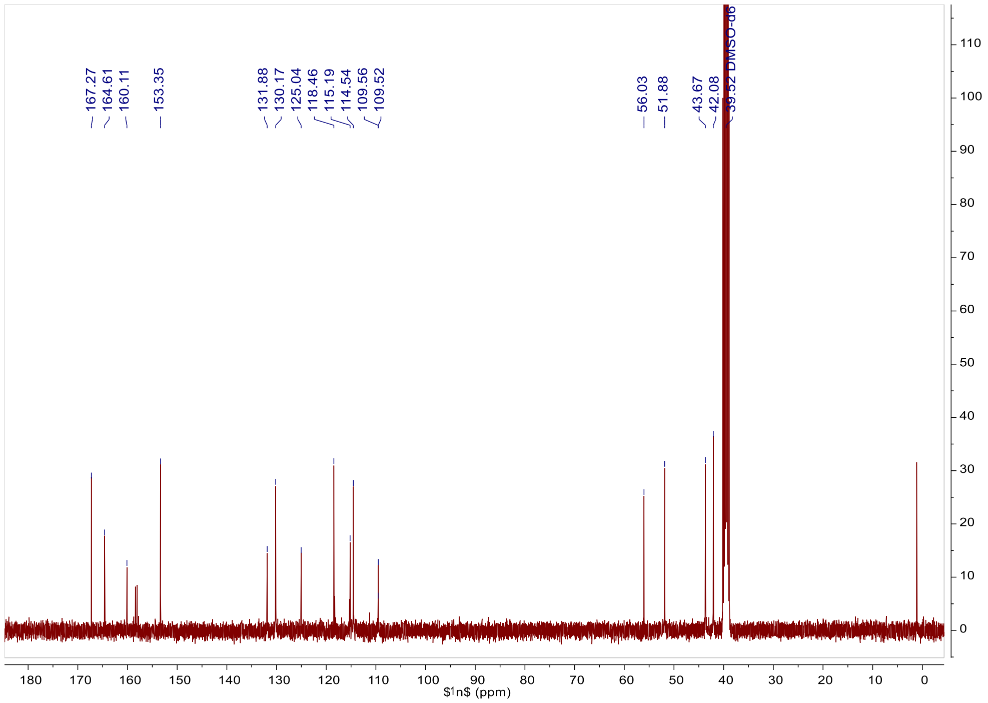


*Figure S76: $^{13}$C-NMR spectrum of 4,4'-((4-(4-methoxyphenyl)pyrylium-2,6-diyl)bis(4,1-phenylene))bis(1-methylpiperazin-1-ium) tris(trifluoroacetate), measured at 293 K in DMSO-$d_6$*

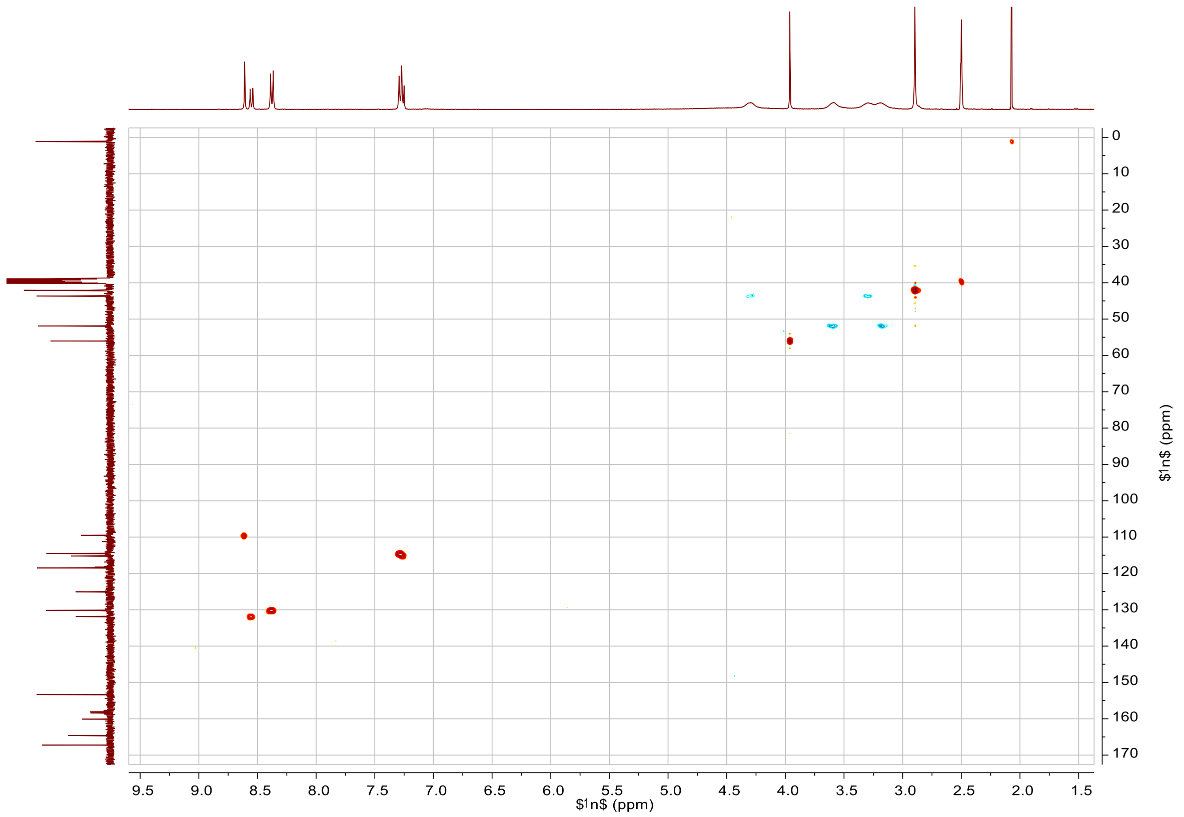


*Figure S77: HSQC spectrum of 4,4'-((4-(4-methoxyphenyl)pyrylium-2,6-diyl)bis(4,1-phenylene))bis(1-methylpiperazin-1-ium) tris(trifluoroacetate), measured at 293 K in DMSO-$d_6$*

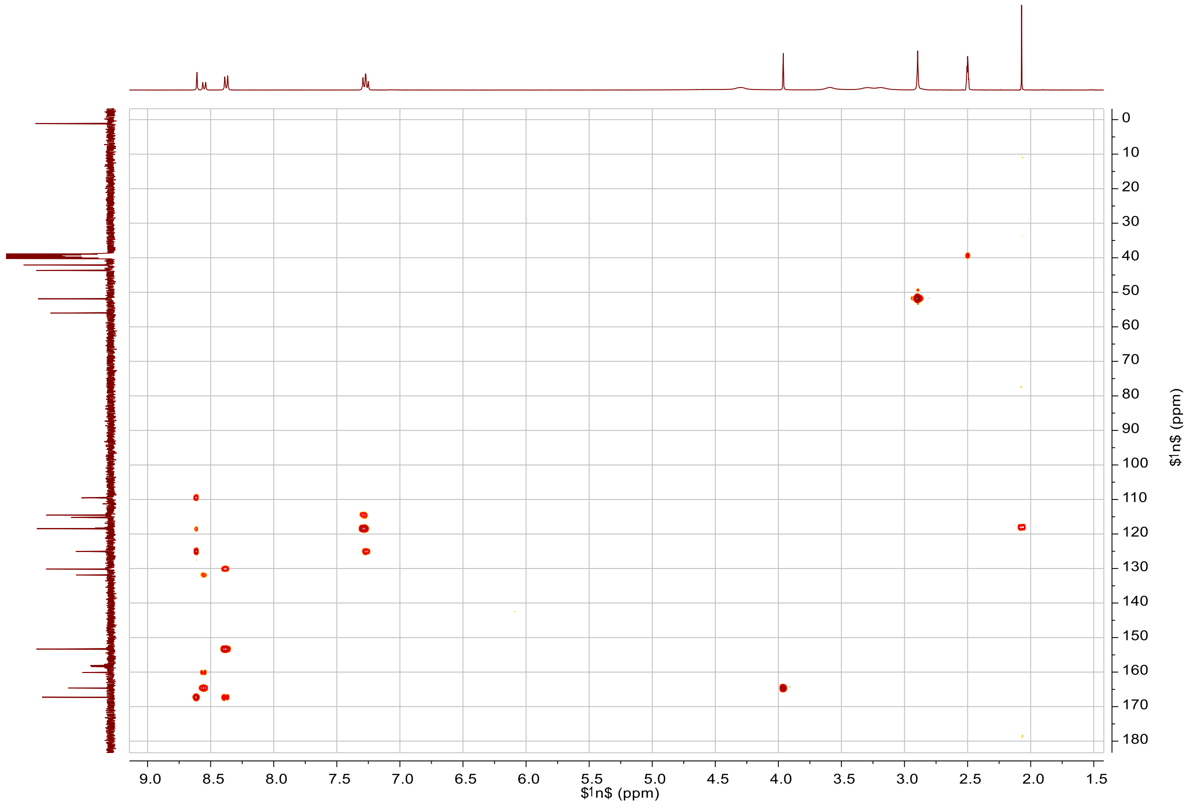


*Figure S78: HMBC spectrum of 4,4'-((4-(4-methoxyphenyl)pyrylium-2,6-diyl)bis(4,1-phenylene))bis(1-methylpiperazin-1-ium) tris(trifluoroacetate), measured at 293 K in DMSO-$d_6$*

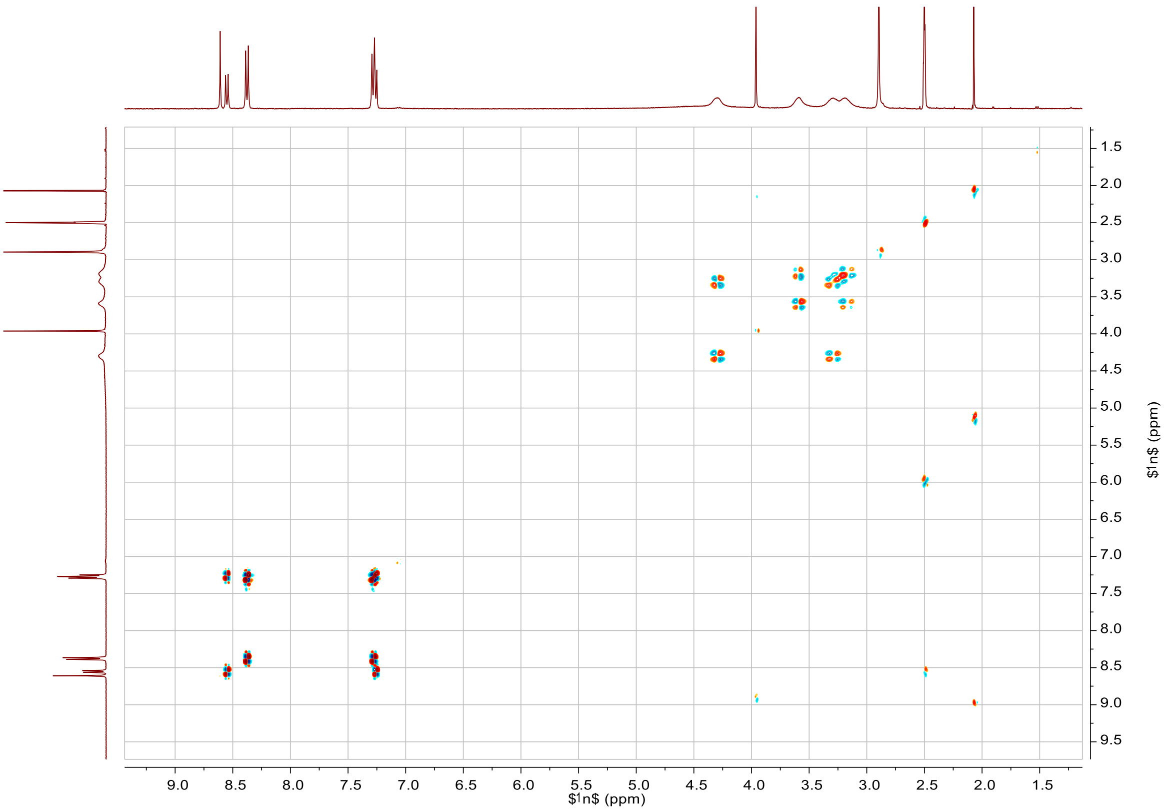


*Figure S79: COSY spectrum of 4,4'-((4-(4-methoxyphenyl)pyrylium-2,6-diyl)bis(4,1-phenylene))bis(1-methylpiperazin-1-ium) tris(trifluoroacetate), measured at 293 K in DMSO-$d_6$*

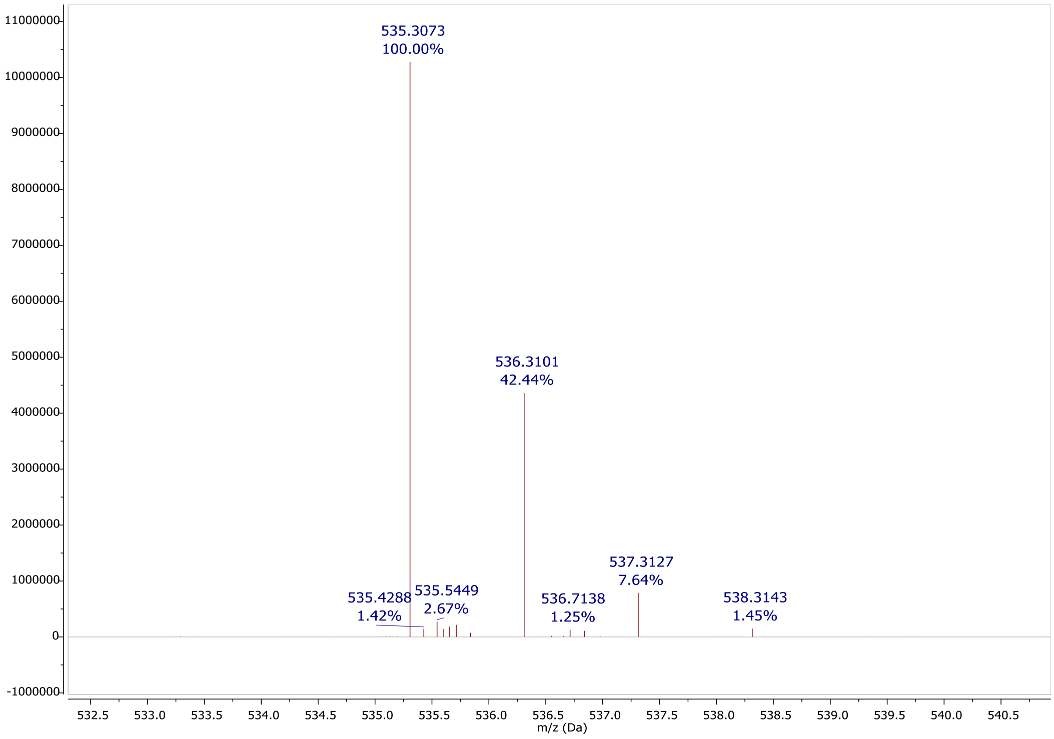


*Figure S80: HRMS spectrum of 4,4'-((4-(4-methoxyphenyl)pyrylium-2,6-diyl)bis(4,1-phenylene))bis(1-methylpiperazin-1-ium) tris(trifluoroacetate)*

## 3.18. 2,6-NMP-4-OEt 4,4'-((4-(4-ethoxyphenyl)pyrylium-2,6-diyl)bis(4,1-phenylene))bis(1-methylpiperazin-1-ium) tris(trifluoroacetate)

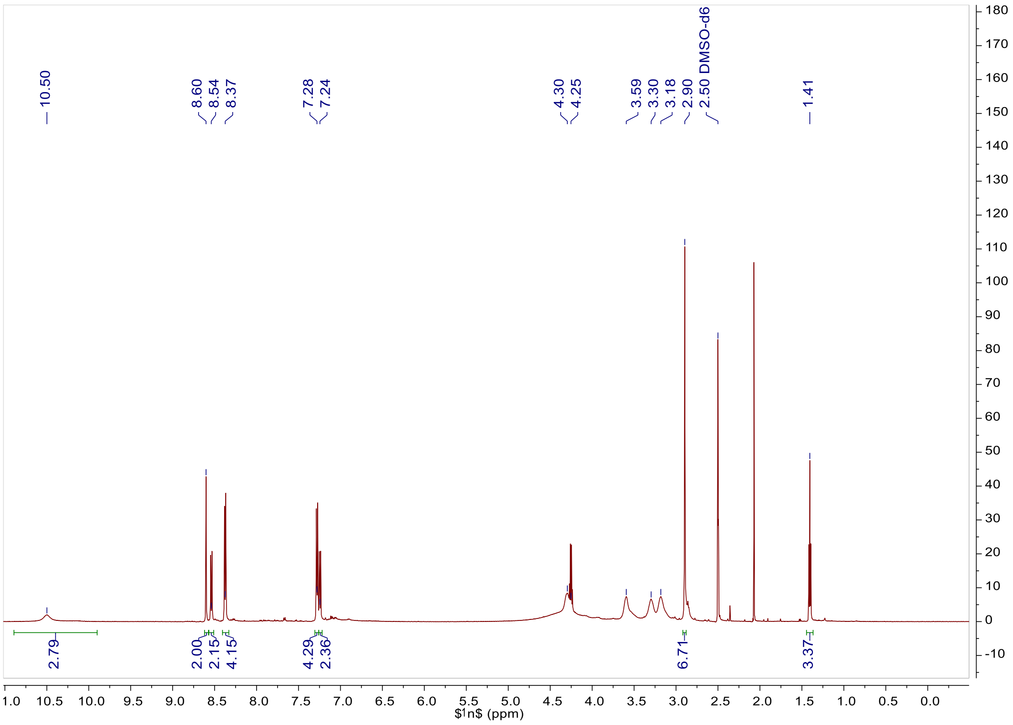


*Figure S81: $^{1}$H-NMR spectrum of 4,4'-((4-(4-ethoxyphenyl)pyrylium-2,6-diyl)bis(4,1-phenylene))bis(1-methylpiperazin-1-ium) tris(trifluoroacetate), measured at 293 K in DMSO-$d_6$*

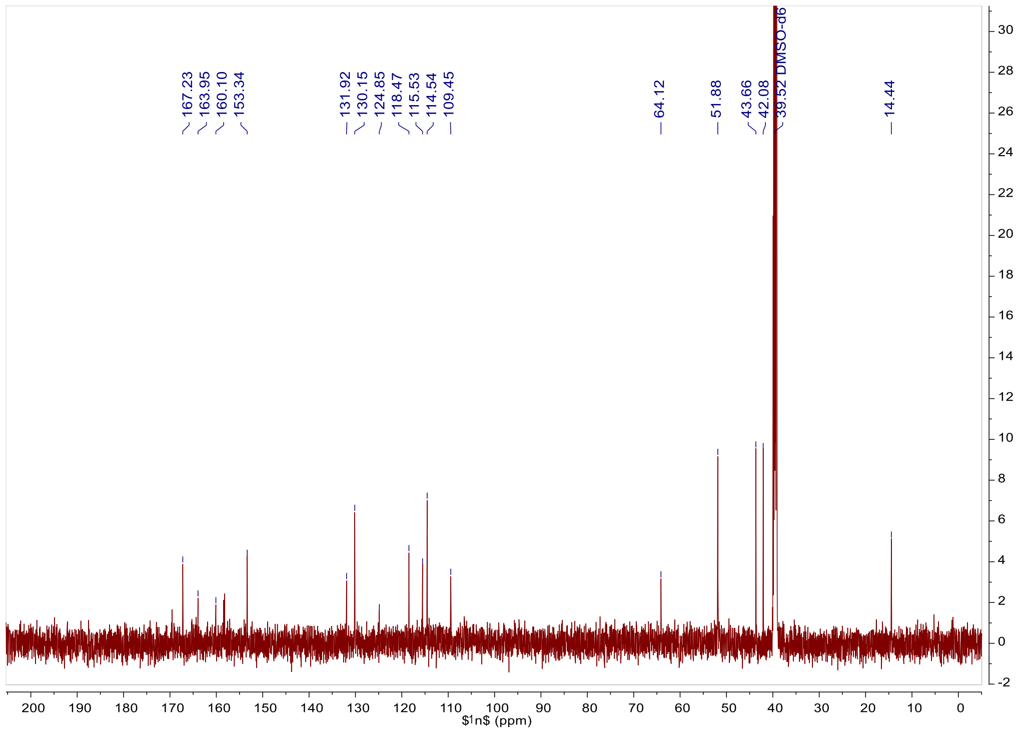


*Figure S82: $^{13}$C-NMR spectrum of 4,4'-((4-(4-ethoxyphenyl)pyrylium-2,6-diyl)bis(4,1-phenylene))bis(1-methylpiperazin-1-ium) tris(trifluoroacetate), measured at 293 K in DMSO-$d_6$*

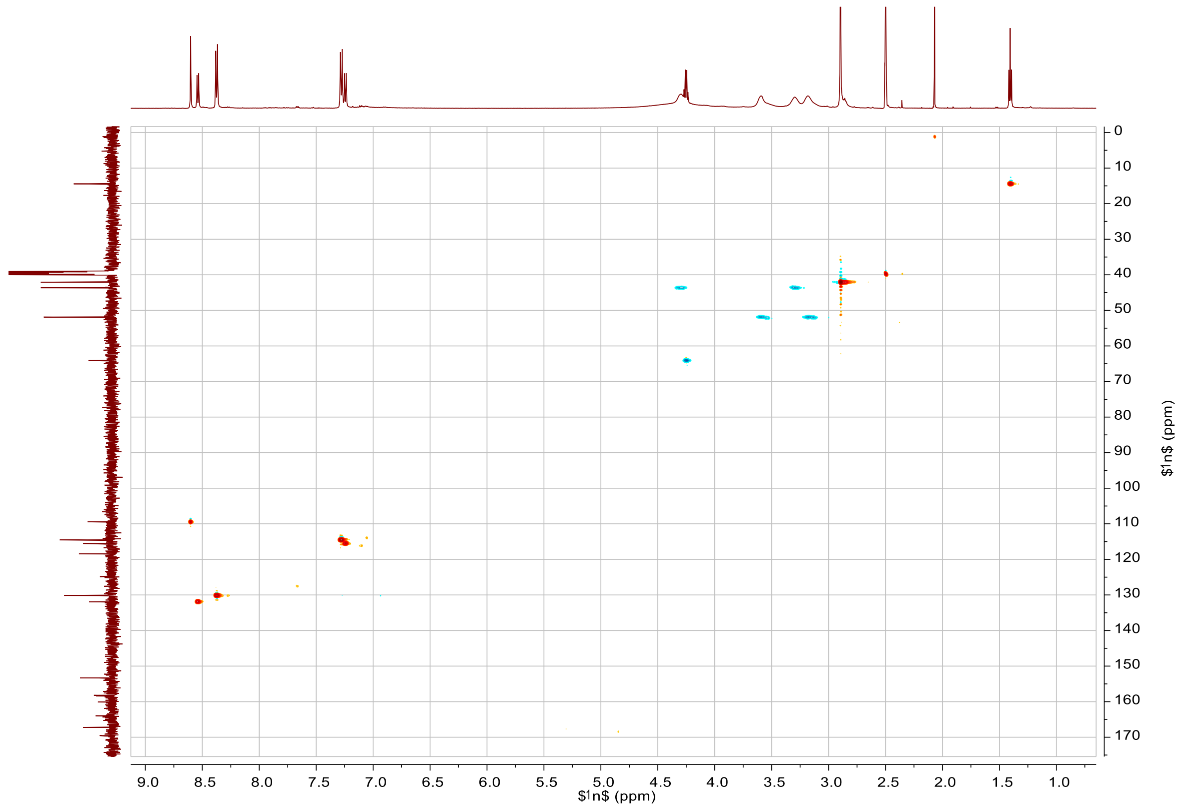


*Figure S83: HSQC spectrum of 4,4'-((4-(4-ethoxyphenyl)pyrylium-2,6-diyl)bis(4,1-phenylene))bis(1-methylpiperazin-1-ium) tris(trifluoroacetate), measured at 293 K in DMSO-$d_6$*

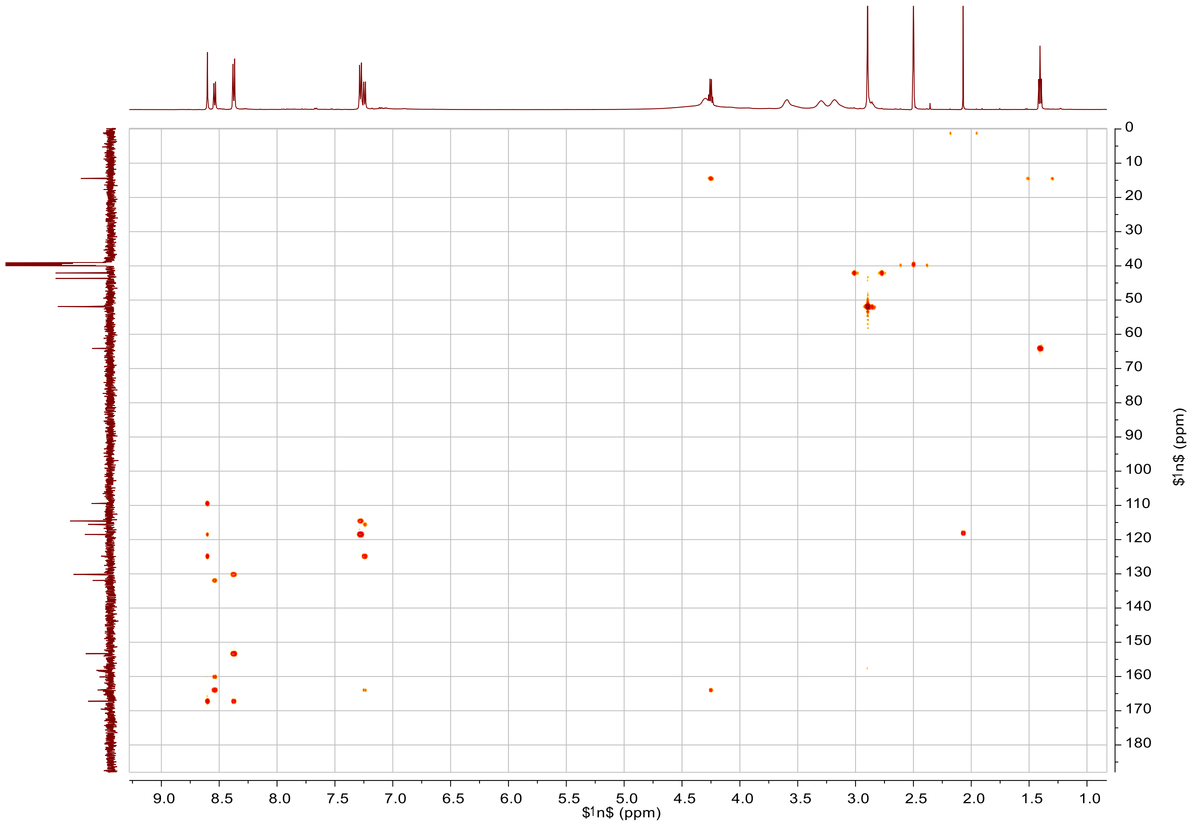


*Figure S84: HMBC spectrum of 4,4'-((4-(4-ethoxyphenyl)pyrylium-2,6-diyl)bis(4,1-phenylene))bis(1-methylpiperazin-1-ium) tris(trifluoroacetate), measured at 293 K in DMSO-$d_6$*

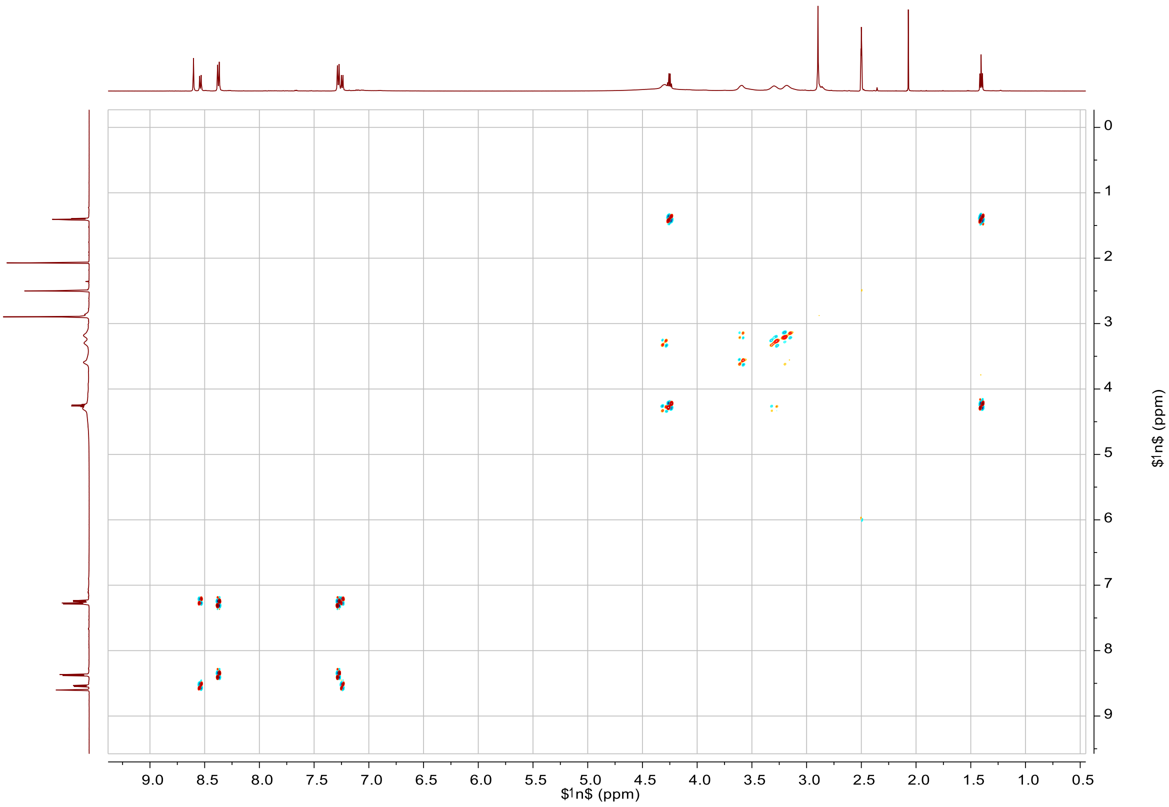


*Figure S85: COSY spectrum of 4,4'-((4-(4-ethoxyphenyl)pyrylium-2,6-diyl)bis(4,1-phenylene))bis(1-methylpiperazin-1-ium) tris(trifluoroacetate), measured at 293 K in DMSO-$d_6$*

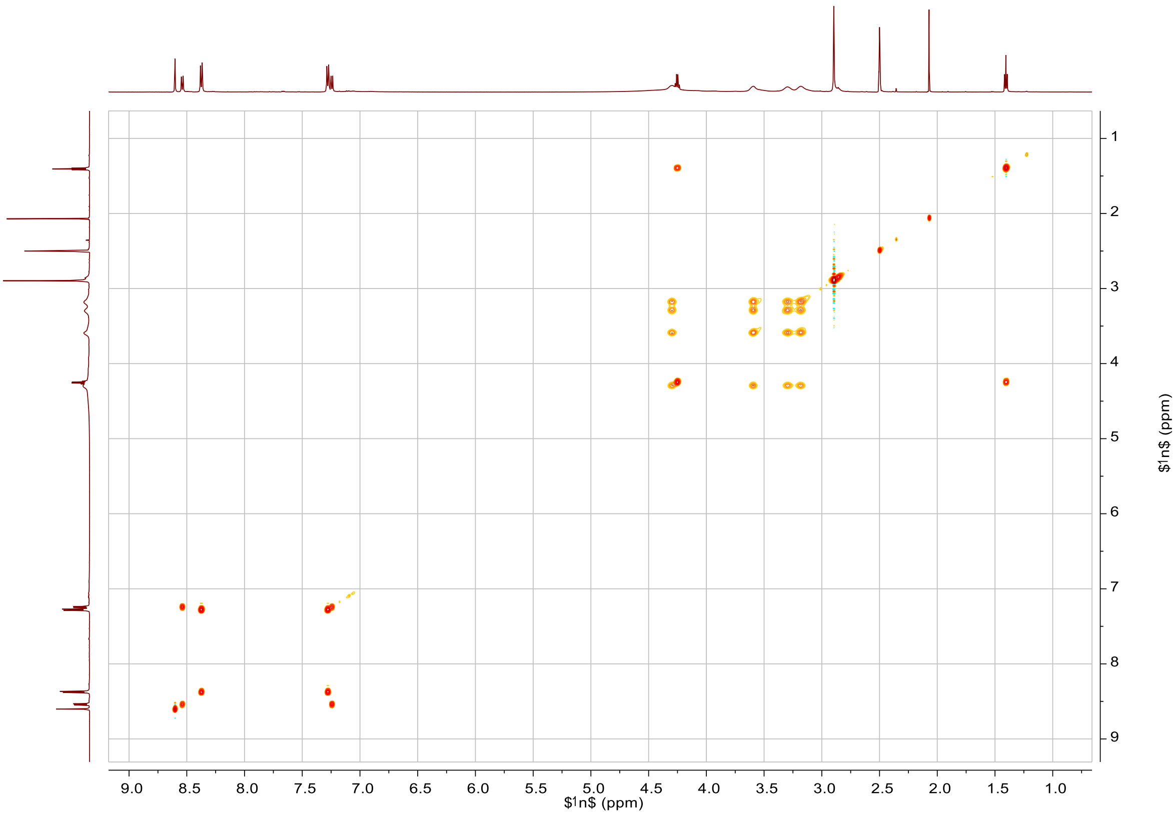


*Figure S86: TOCSY spectrum of 4,4'-((4-(4-ethoxyphenyl)pyrylium-2,6-diyl)bis(4,1-phenylene))bis(1-methylpiperazin-1-ium) tris(trifluoroacetate), measured at 293 K in DMSO-$d_6$*

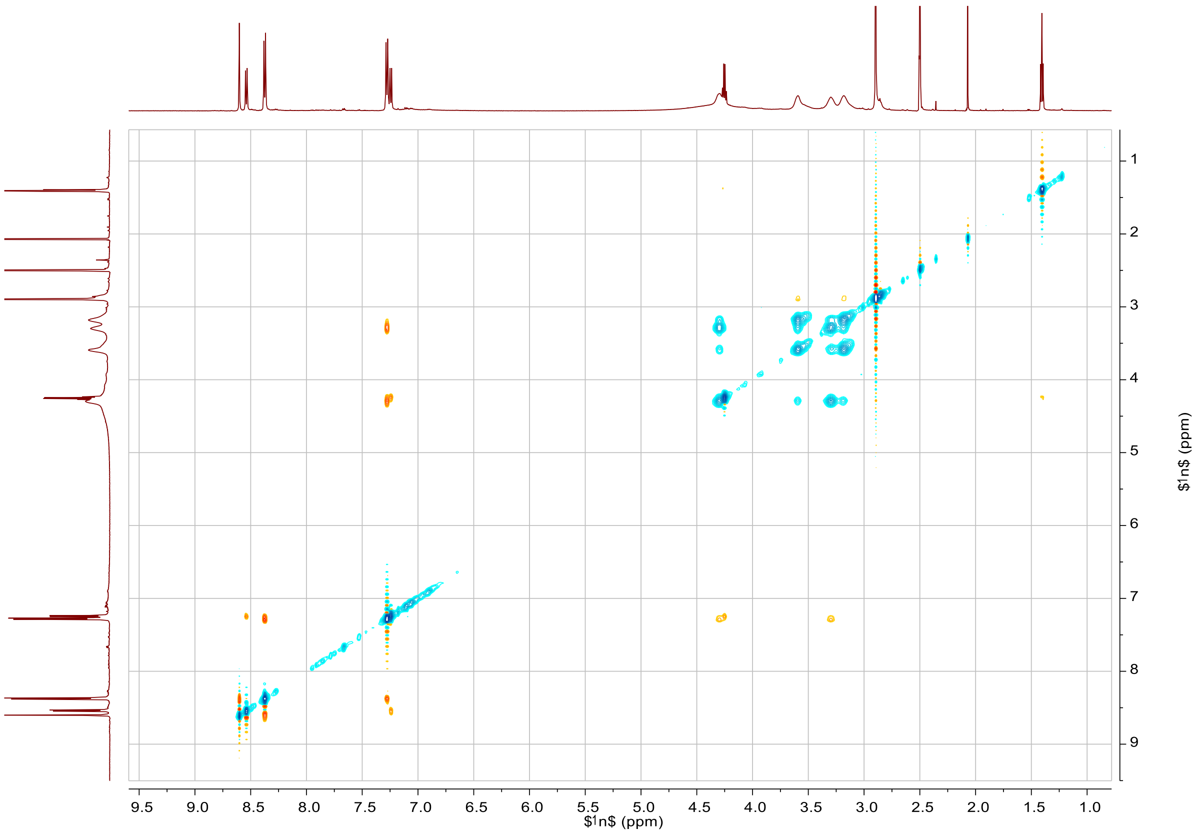


*Figure S87: ROESY spectrum of 4,4'-((4-(4-ethoxyphenyl)pyrylium-2,6-diyl)bis(4,1-phenylene))bis(1-methylpiperazin-1-ium) tris(trifluoroacetate), measured at 293 K in DMSO-$d_6$*

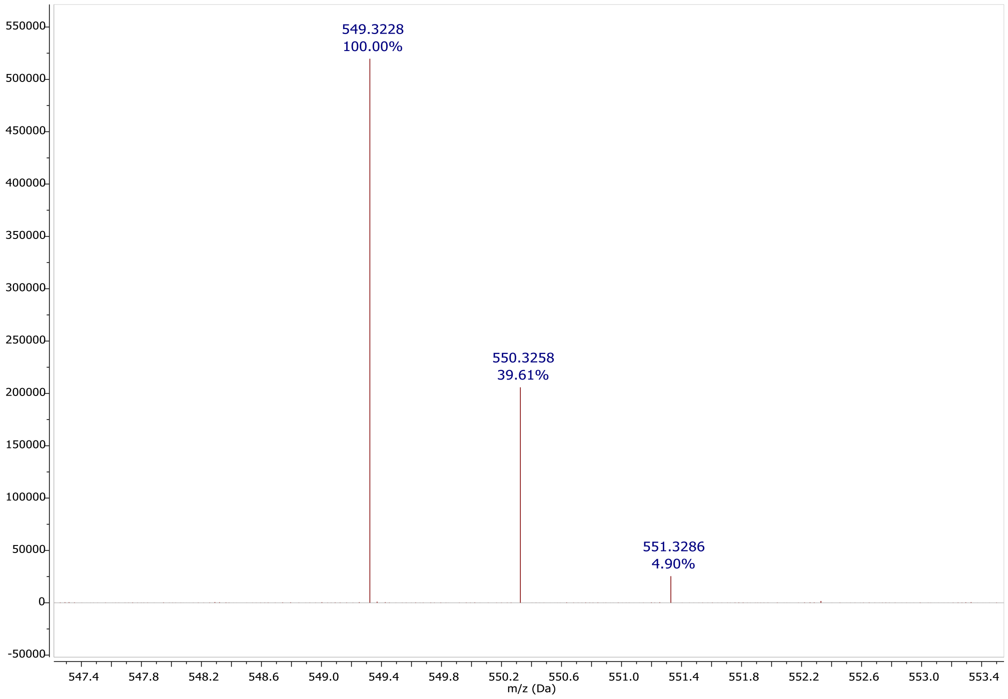


*Figure S88: HRMS spectrum of 4,4'-((4-(4-ethoxyphenyl)pyrylium-2,6-diyl)bis(4,1-phenylene))bis(1-methylpiperazin-1-ium) tris(trifluoroacetate)*

### 3.19. 2,6-NMP-4-OH - 4-(4-hydroxyphenyl)-2,6-bis(4-(4-methylpiperazin-1-yl)phenyl)pyrylium tris(trifluoroborate)

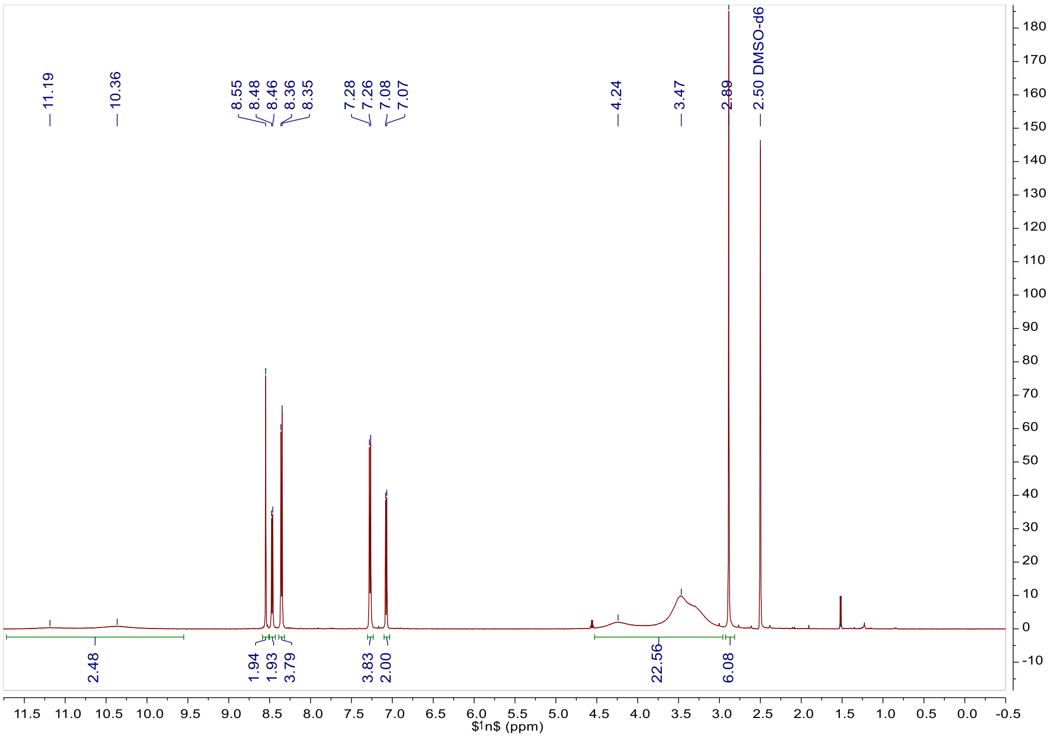


*Figure S89: $^{1}$H-NMR spectrum of 4-(4-hydroxyphenyl)-2,6-bis(4-(4-methylpiperazin-1-yl)phenyl)pyrylium tris(trifluoroborate), measured at 293 K in DMSO-$d_6$*

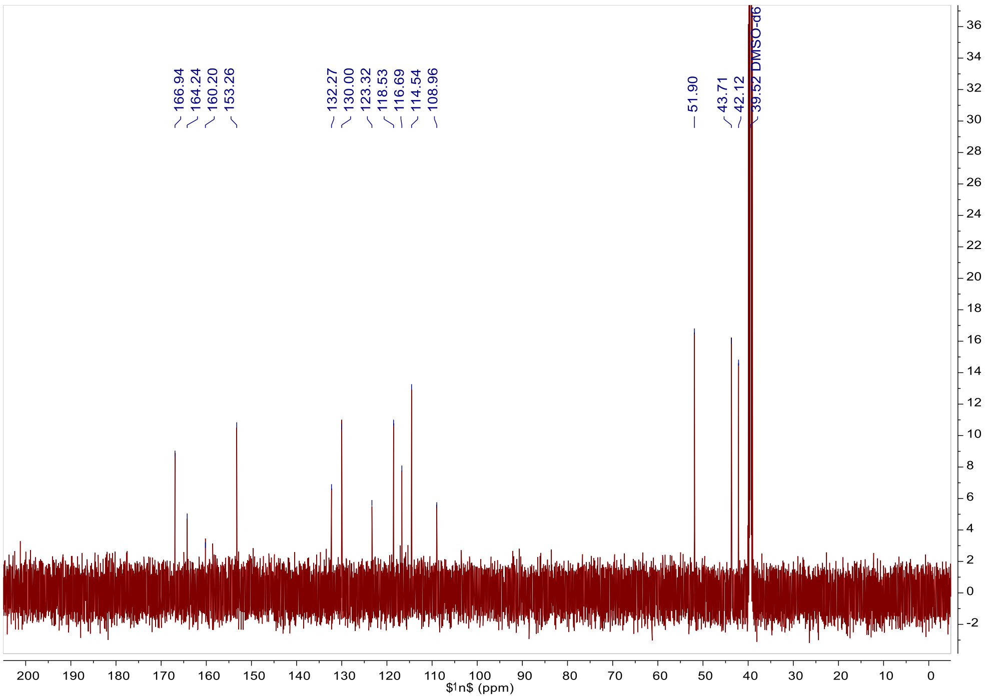


*Figure S90: $^{13}$C-NMR spectrum of 4-(4-hydroxyphenyl)-2,6-bis(4-(4-methylpiperazin-1-yl)phenyl)pyrylium tris(trifluoroborate), measured at 293 K in DMSO-$d_6$*

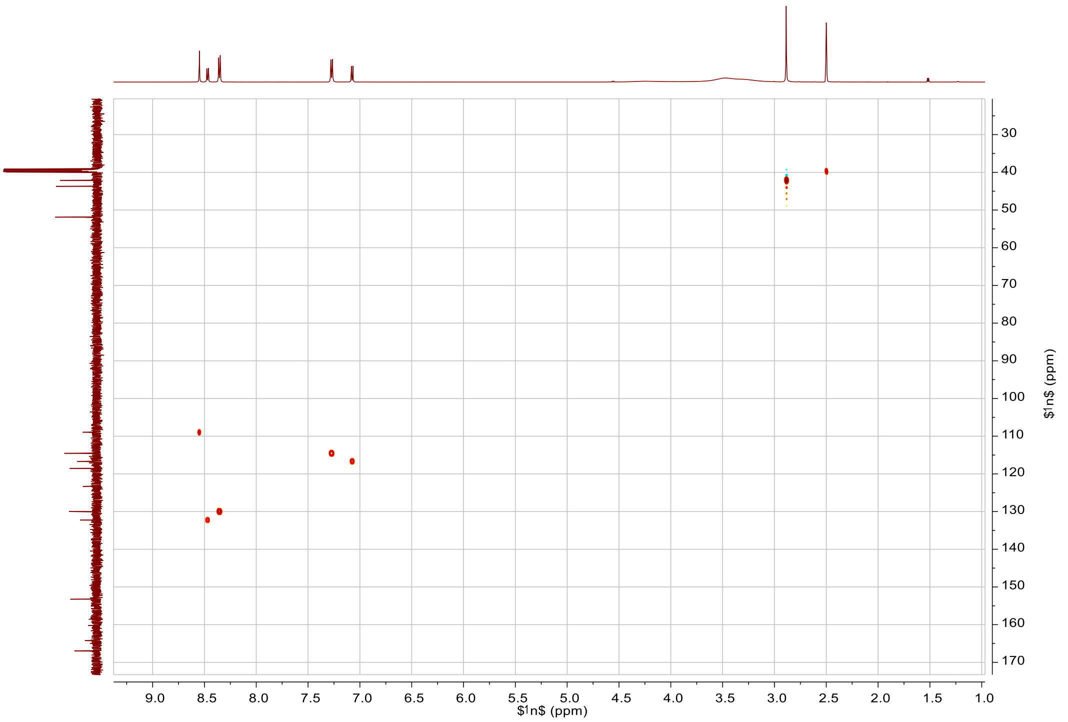


*Figure S91: HSQC spectrum of 4-(4-hydroxyphenyl)-2,6-bis(4-(4-methylpiperazin-1-yl)phenyl)pyrylium tris(trifluoroborate) measured at 293 K in DMSO-$d_6$*

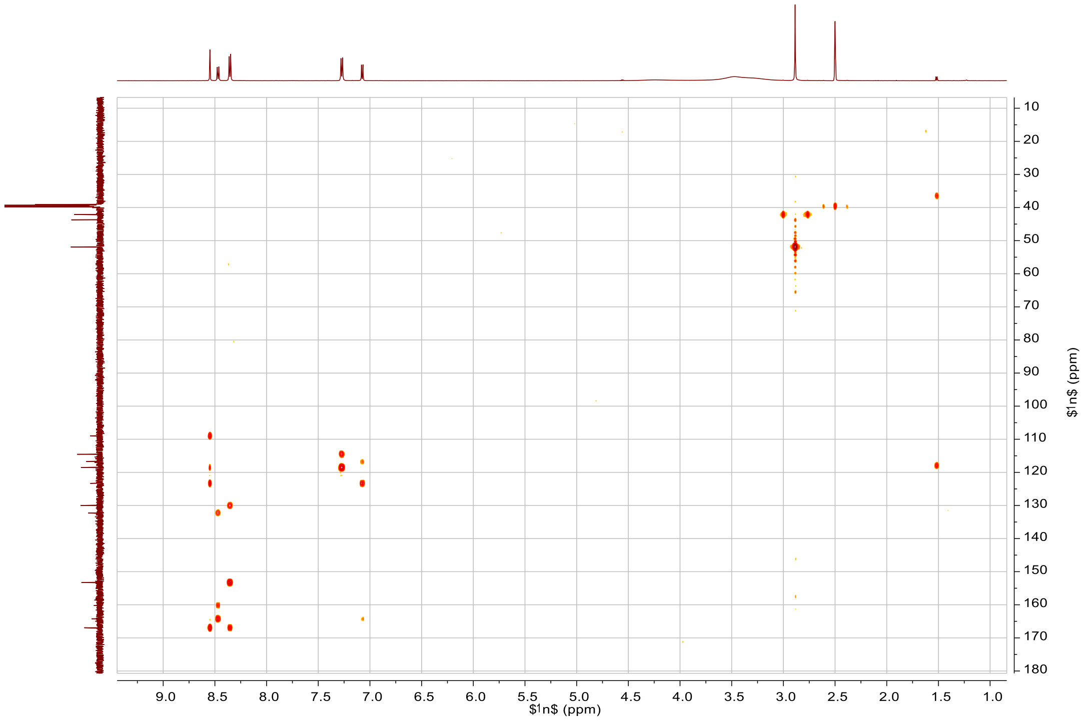


*Figure S92: HMBC spectrum of 4-(4-hydroxyphenyl)-2,6-bis(4-(4-methylpiperazin-1-yl)phenyl)pyrylium tris(trifluoroborate) measured at 293 K in DMSO-$d_6$*

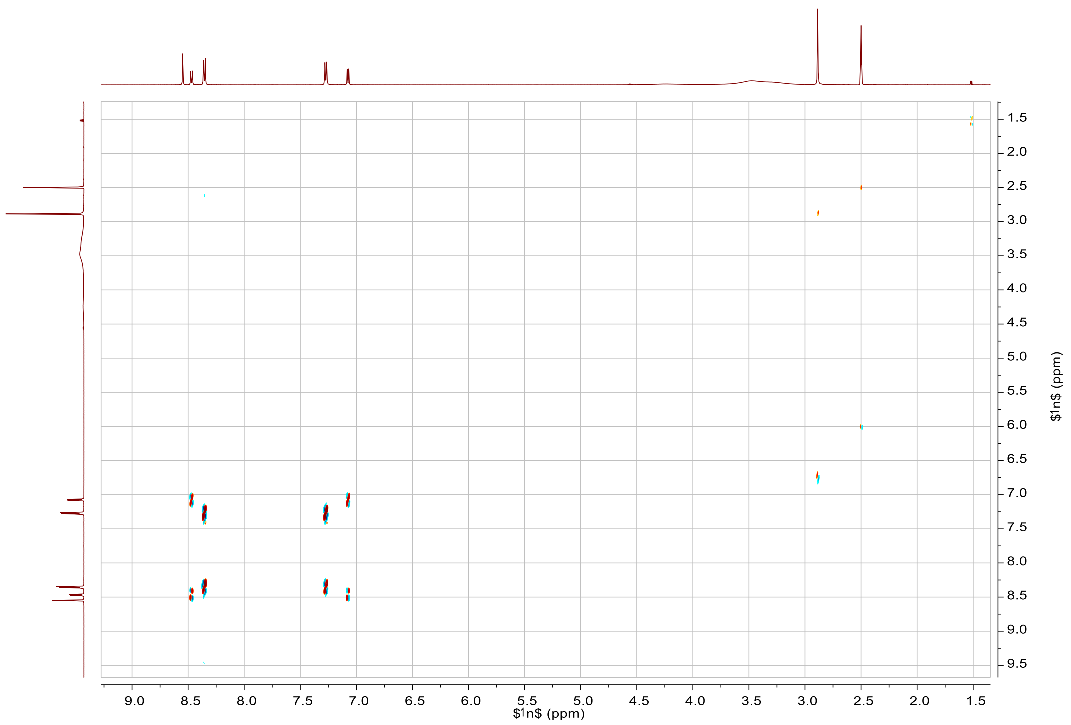


*Figure S93: COSY spectrum of 4-(4-hydroxyphenyl)-2,6-bis(4-(4-methylpiperazin-1-yl)phenyl)pyrylium tris(trifluoroborate) measured at 293 K in DMSO-$d_6$*

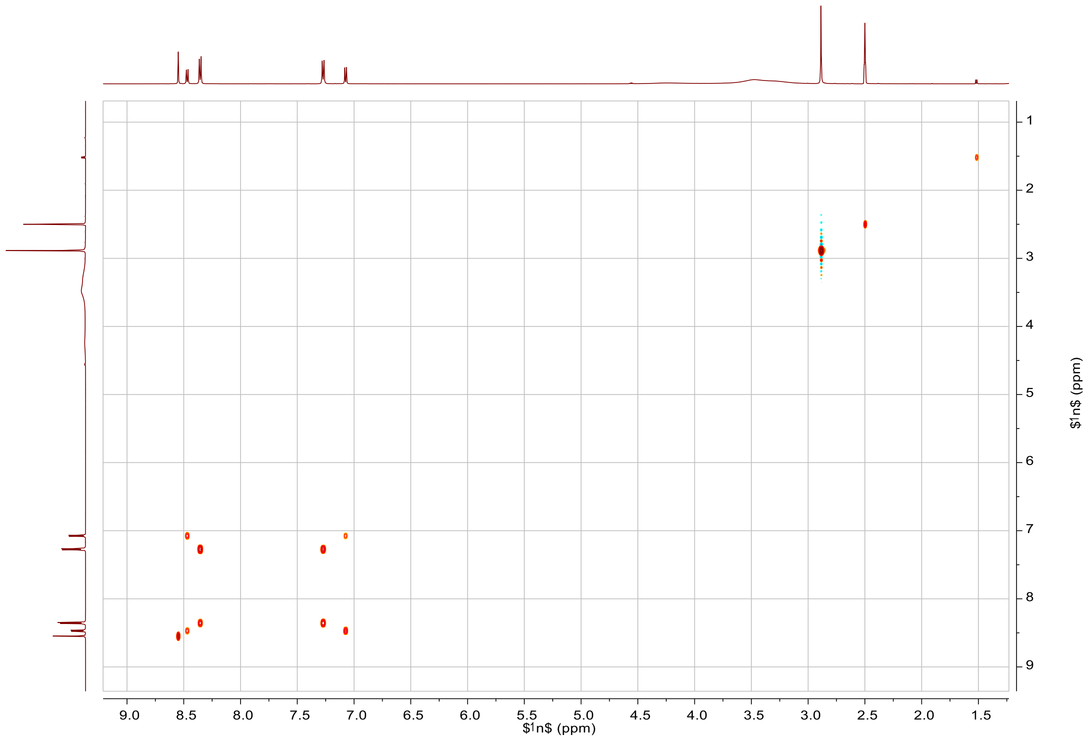


*Figure S94: TOCSY spectrum of 4-(4-hydroxyphenyl)-2,6-bis(4-(4-methylpiperazin-1-yl)phenyl)pyrylium tris(trifluoroborate) measured at 293 K in DMSO-$d_6$*

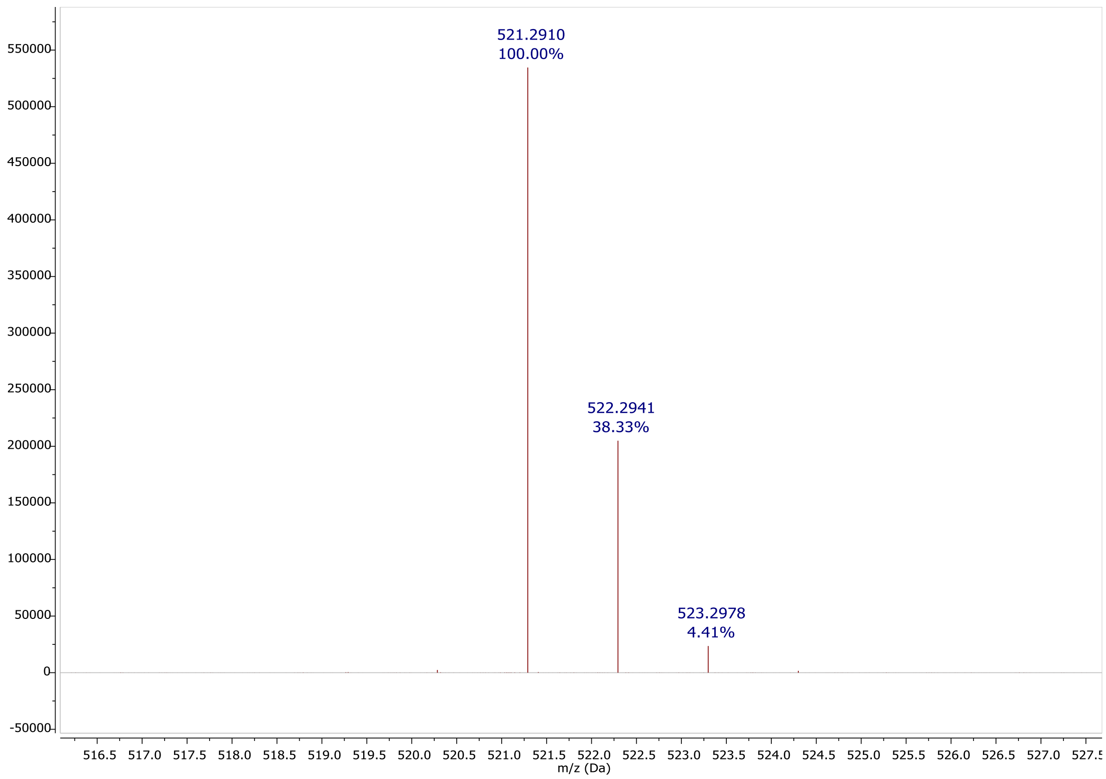


*Figure S95: HRMS spectrum of 4-(4-hydroxyphenyl)-2,6-bis(4-(4-methylpiperazin-1-yl)phenyl)pyrylium tris(trifluoroborate)*

## 3.20. 2,6-NMP-4-2MeOPh - 4,4'-((4-(2-methoxyphenyl)pyrylium-2,6-diyl)bis(4,1-phenylene))bis(1-methylpiperazin-1-ium) tris(trifluoroacetate)

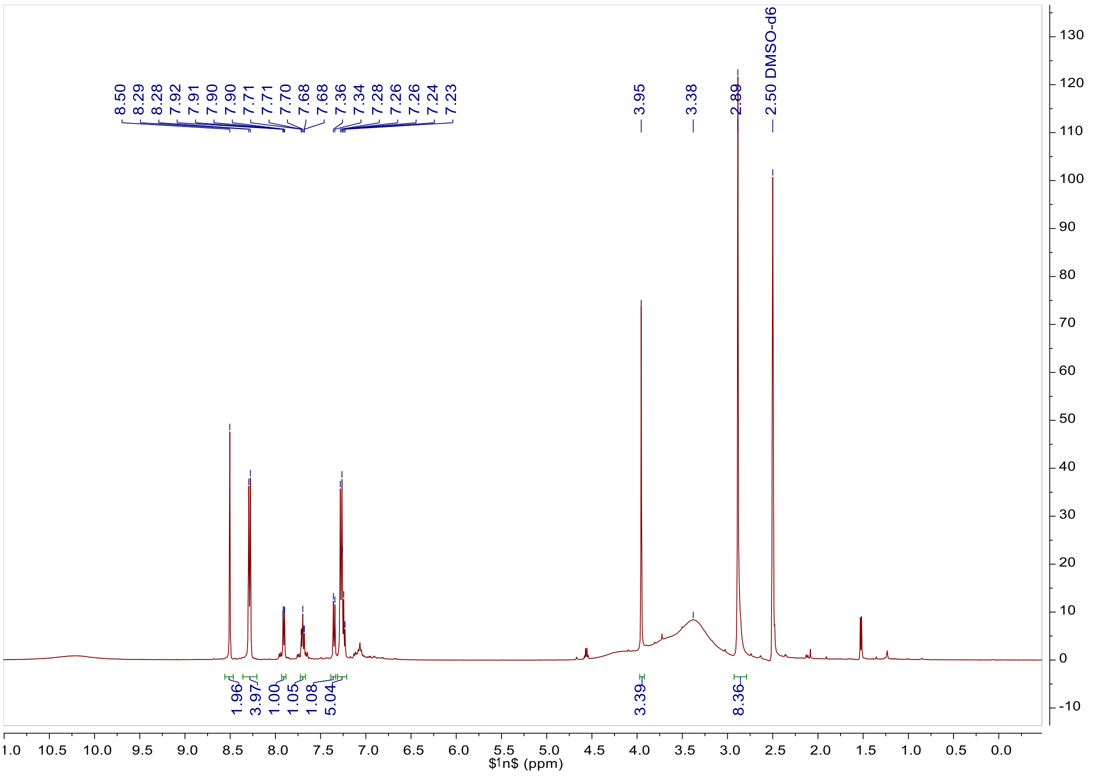


*Figure S96: $^{1}$H-NMR spectrum of 4,4'-((4-(2-methoxyphenyl)pyrylium-2,6-diyl)bis(4,1-phenylene))bis(1-methylpiperazin-1-ium) tris(trifluoroacetate), measured at 293 K in DMSO-$d_6$*

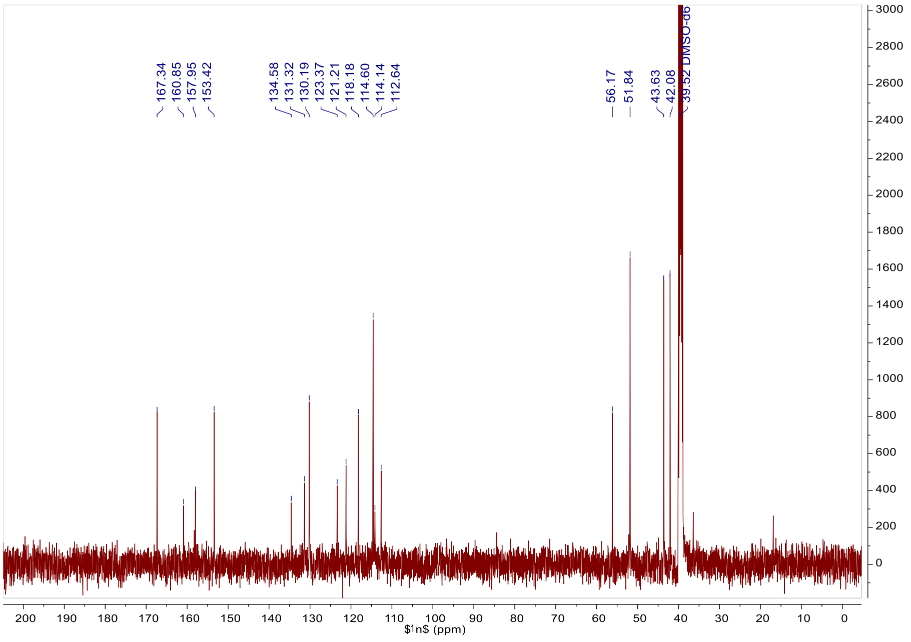


*Figure S97: $^{13}$C-NMR spectrum of 4,4'-((4-(2-methoxyphenyl)pyrylium-2,6-diyl)bis(4,1-phenylene))bis(1-methylpiperazin-1-ium) tris(trifluoroacetate), measured at 293 K in DMSO-$d_6$*

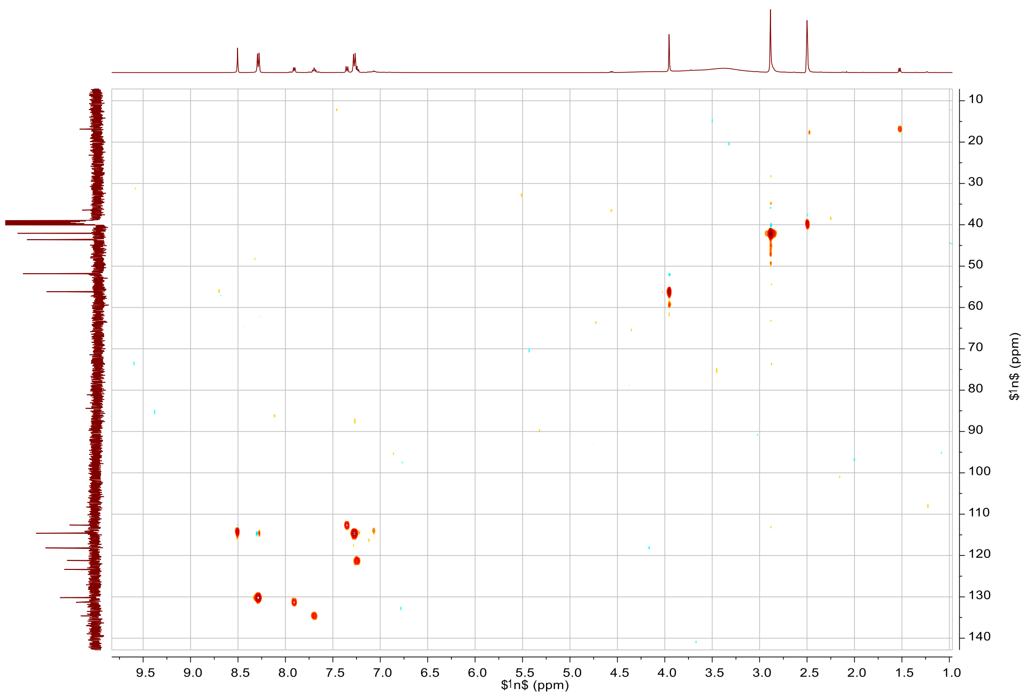


*Figure S98: HSQC spectrum of 4,4'-((4-(2-methoxyphenyl)pyrylium-2,6-diyl)bis(4,1-phenylene))bis(1-methylpiperazin-1-ium) tris(trifluoroacetate) measured at 293 K in DMSO-$d_6$*

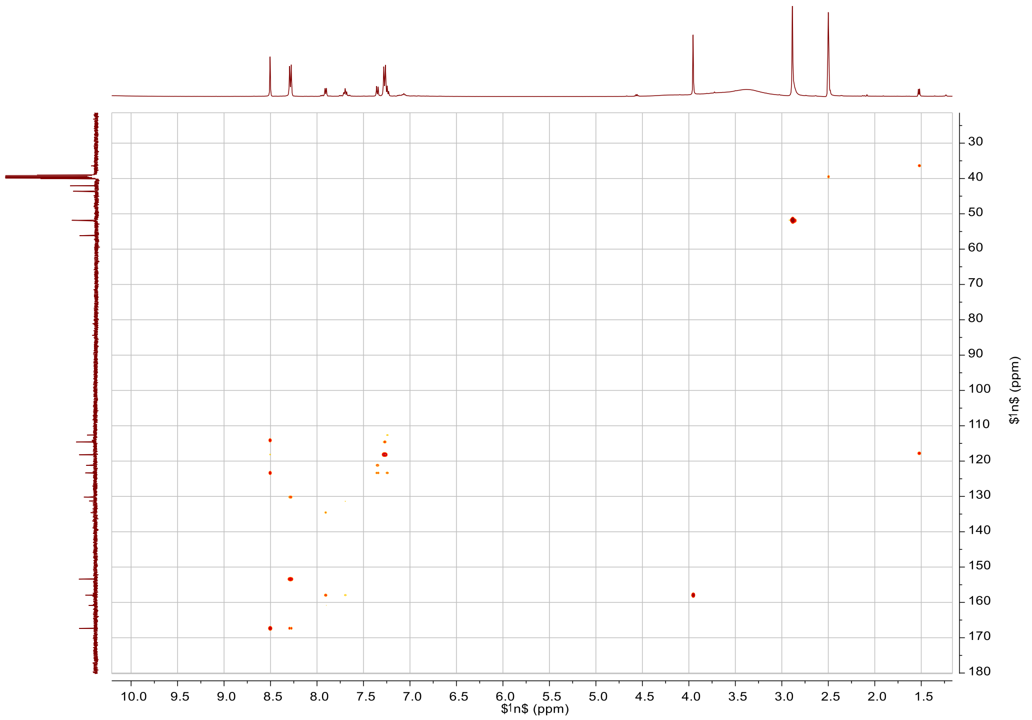


*Figure S99: HMBC spectrum of 4,4'-((4-(2-methoxyphenyl)pyrylium-2,6-diyl)bis(4,1-phenylene))bis(1-methylpiperazin-1-ium) tris(trifluoroacetate) measured at 293 K in DMSO-$d_6$*

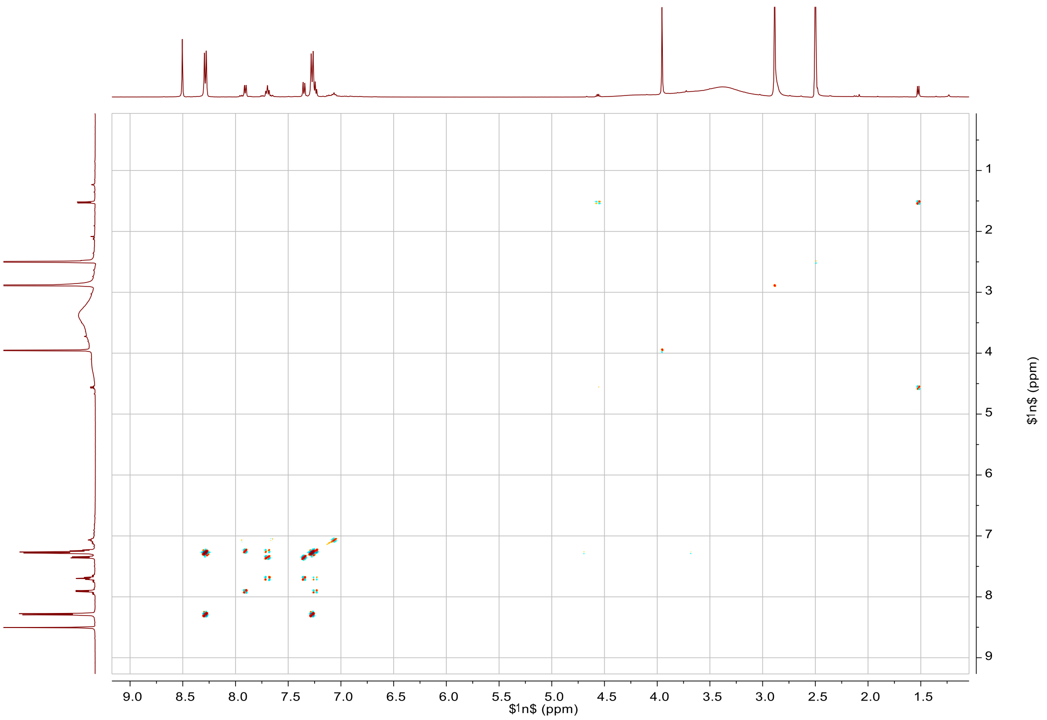


*Figure S100: COSY spectrum of 4,4'-((4-(2-methoxyphenyl)pyrylium-2,6-diyl)bis(4,1-phenylene))bis(1-methylpiperazin-1-ium) tris(trifluoroacetate) measured at 293 K in DMSO-$d_6$*

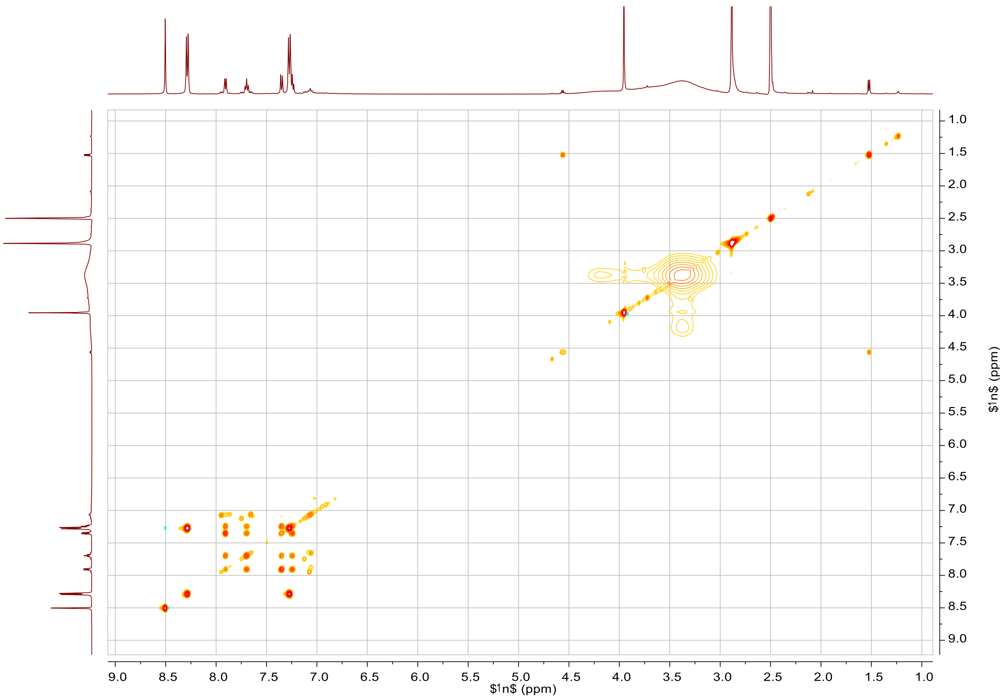


*Figure S101: TOCSY spectrum of 4,4'-((4-(2-methoxyphenyl)pyrylium-2,6-diyl)bis(4,1-phenylene))bis(1-methylpiperazin-1-ium) tris(trifluoroacetate) measured at 293 K in DMSO-$d_6$*

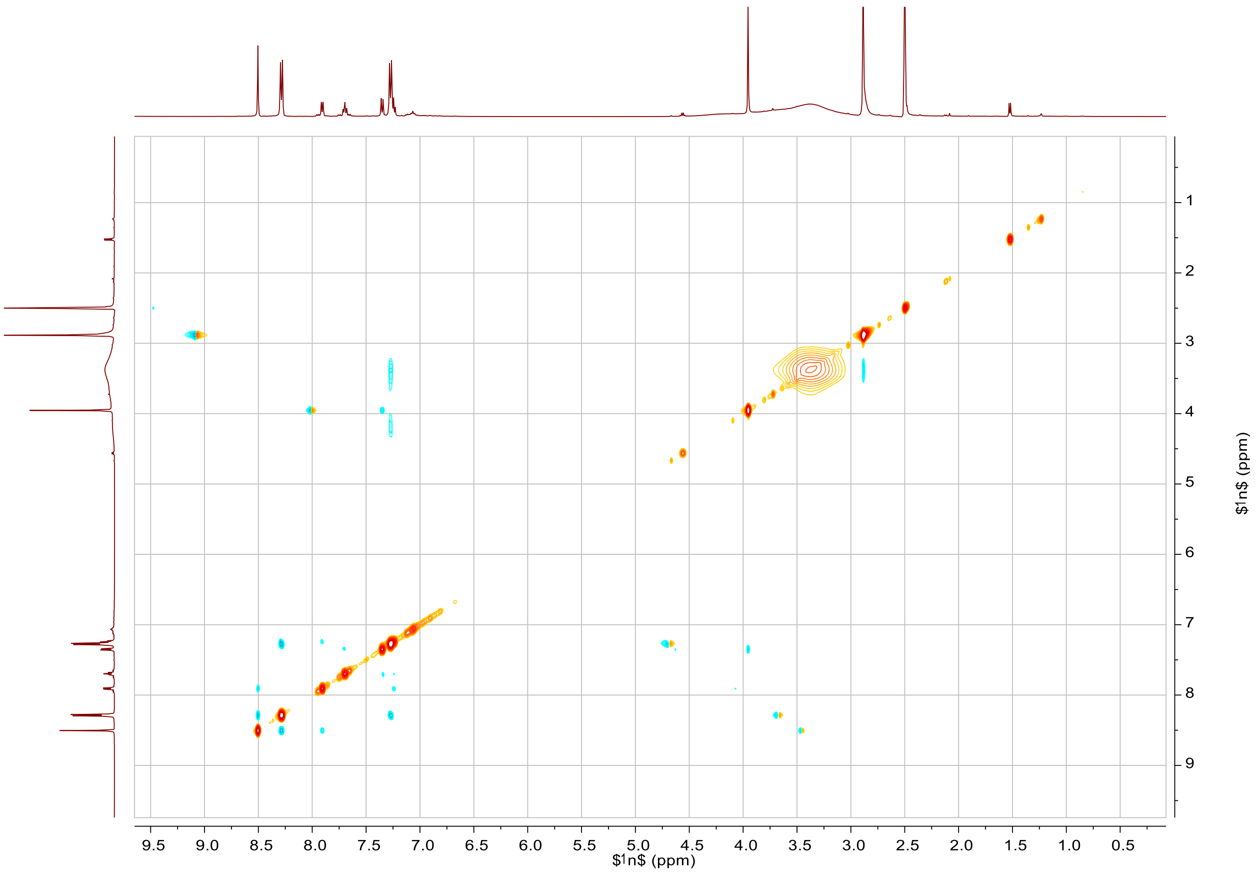


*Figure S102: ROESY spectrum of 4,4'-((4-(2-methoxyphenyl)pyrylium-2,6-diyl)bis(4,1-phenylene))bis(1-methylpiperazin-1-ium) tris(trifluoroacetate) measured at 293 K in DMSO-$d_6$*

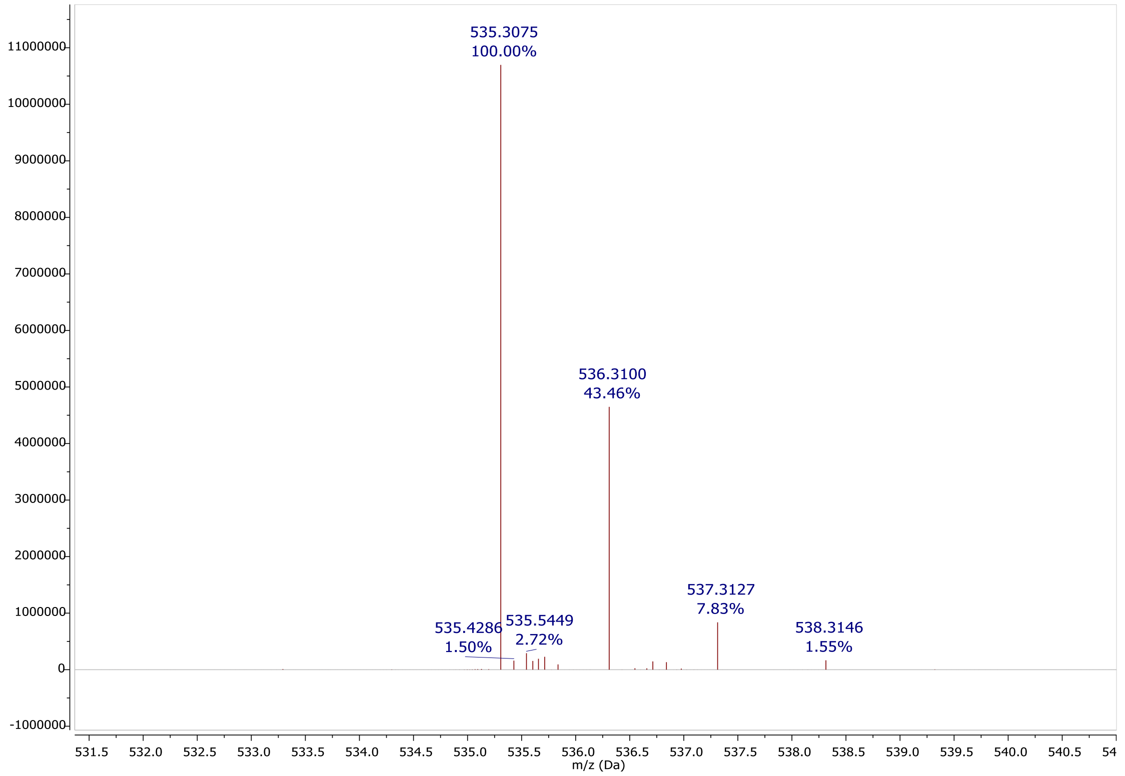


*Figure S103: HRMS spectrum of 4,4'-((4-(2-methoxyphenyl)pyrylium-2,6-diyl)bis(4,1-phenylene))bis(1-methylpiperazin-1-ium) tris(trifluoroacetate)*

### 3.21. 2,6-Pip-4-2MeOPh - 4-(2-methoxyphenyl)-2,6-bis(4-(piperidin-1-yl)phenyl)pyrylium trifluoroacetate

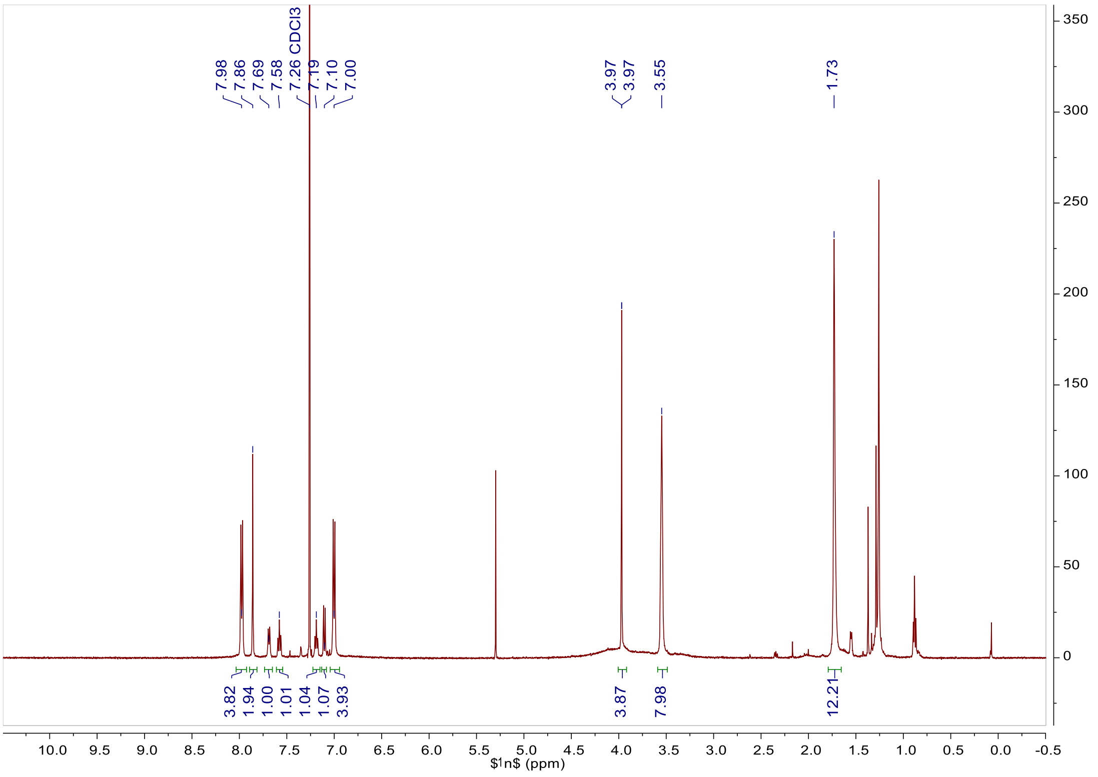


*Figure S104: $^{1}$H-NMR spectrum of 4-(2-methoxyphenyl)-2,6-bis(4-(piperidin-1-yl)phenyl)pyrylium trifluoroacetate, 500 MHz, measured at 293 K in DMSO-$d_6$*

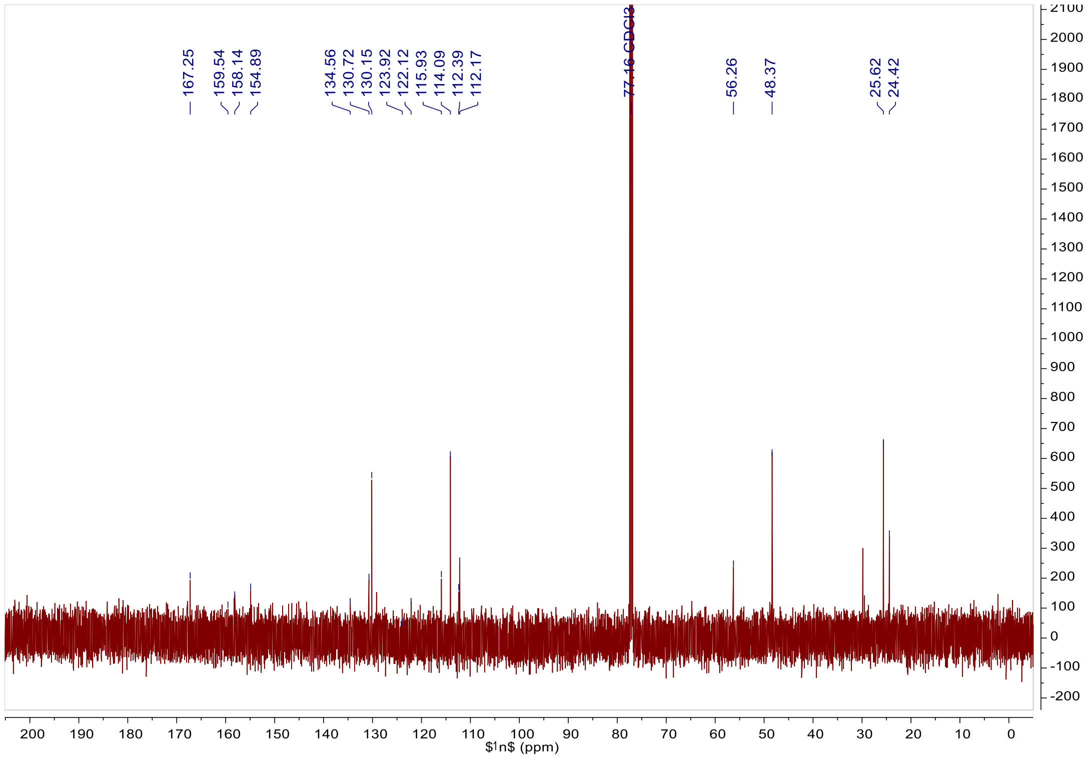


*Figure S105: $^{13}$C-NMR spectrum of 4-(2-methoxyphenyl)-2,6-bis(4-(piperidin-1-yl)phenyl)pyrylium trifluoroacetate, 126 MHz, measured at 293 K in DMSO-$d_6$*

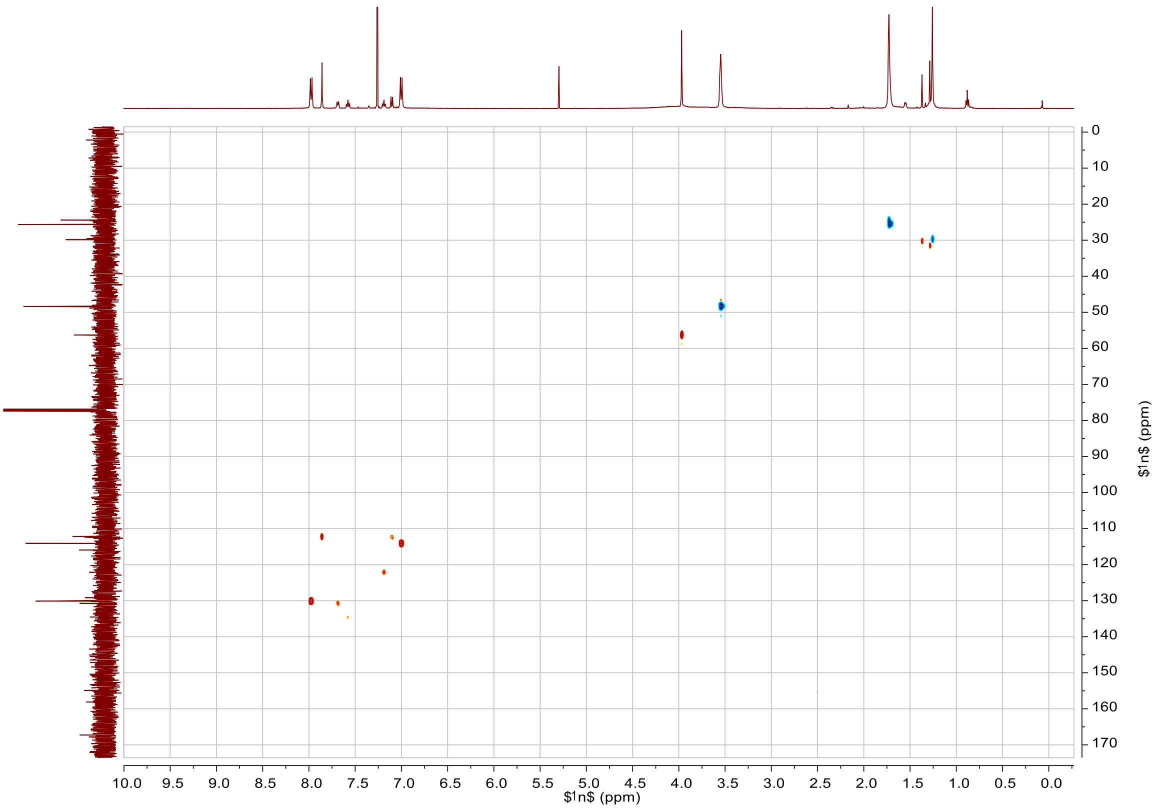


*Figure S106: HSQC spectrum of 4-(2-methoxyphenyl)-2,6-bis(4-(piperidin-1-yl)phenyl)pyrylium trifluoroacetate, measured at 293 K in DMSO-$d_6$*

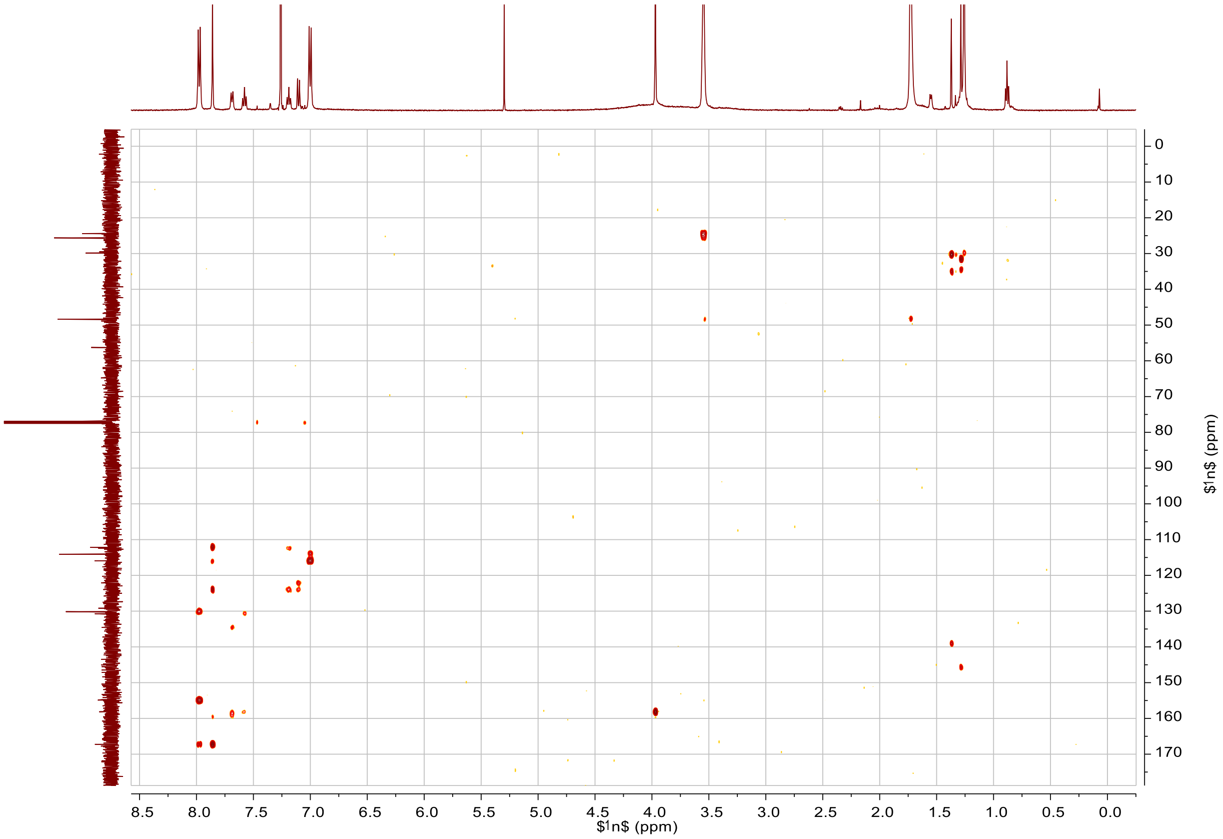


*Figure S107: HMBC spectrum of 4-(2-methoxyphenyl)-2,6-bis(4-(piperidin-1-yl)phenyl)pyrylium trifluoroacetate, measured at 293 K in DMSO-$d_6$*

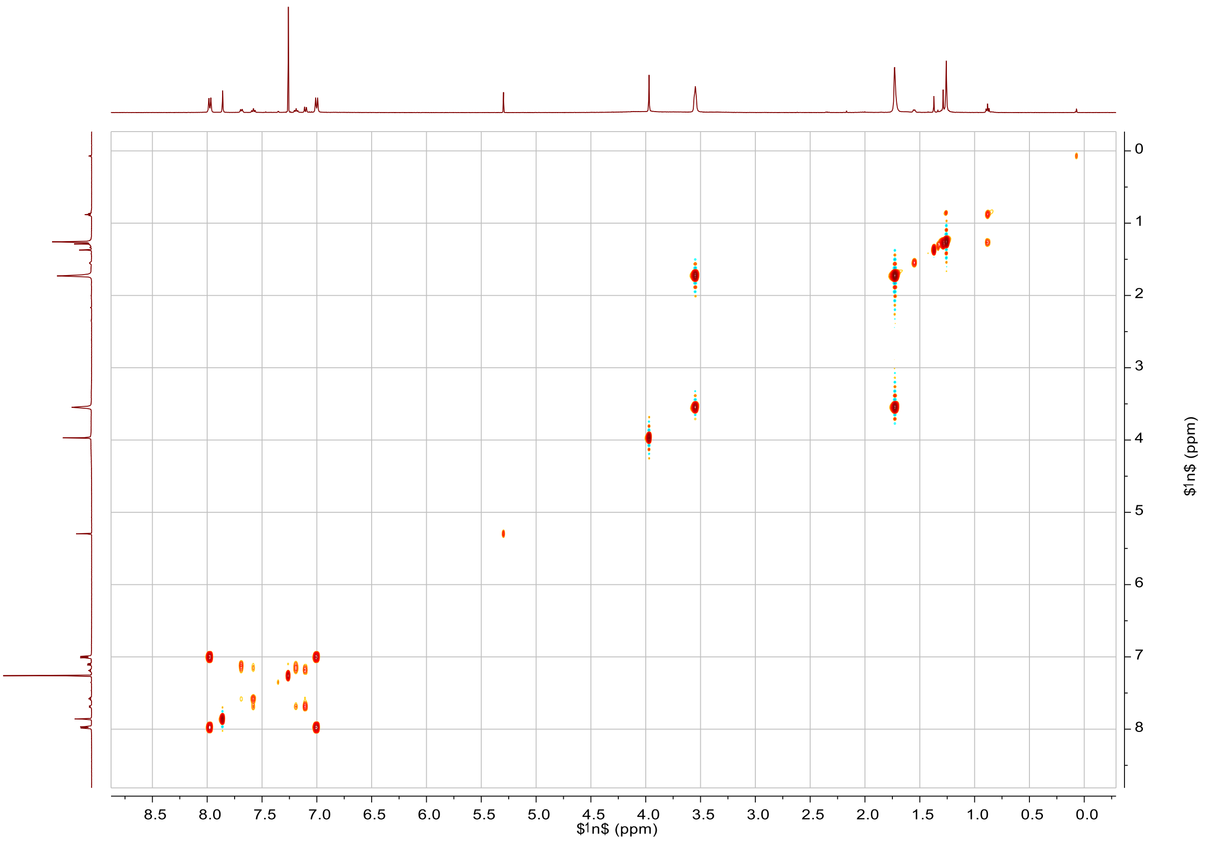


*Figure S108: TOCSY spectrum of 4-(2-methoxyphenyl)-2,6-bis(4-(piperidin-1-yl)phenyl)pyrylium trifluoroacetate, measured at 293 K in DMSO-d$_6$*

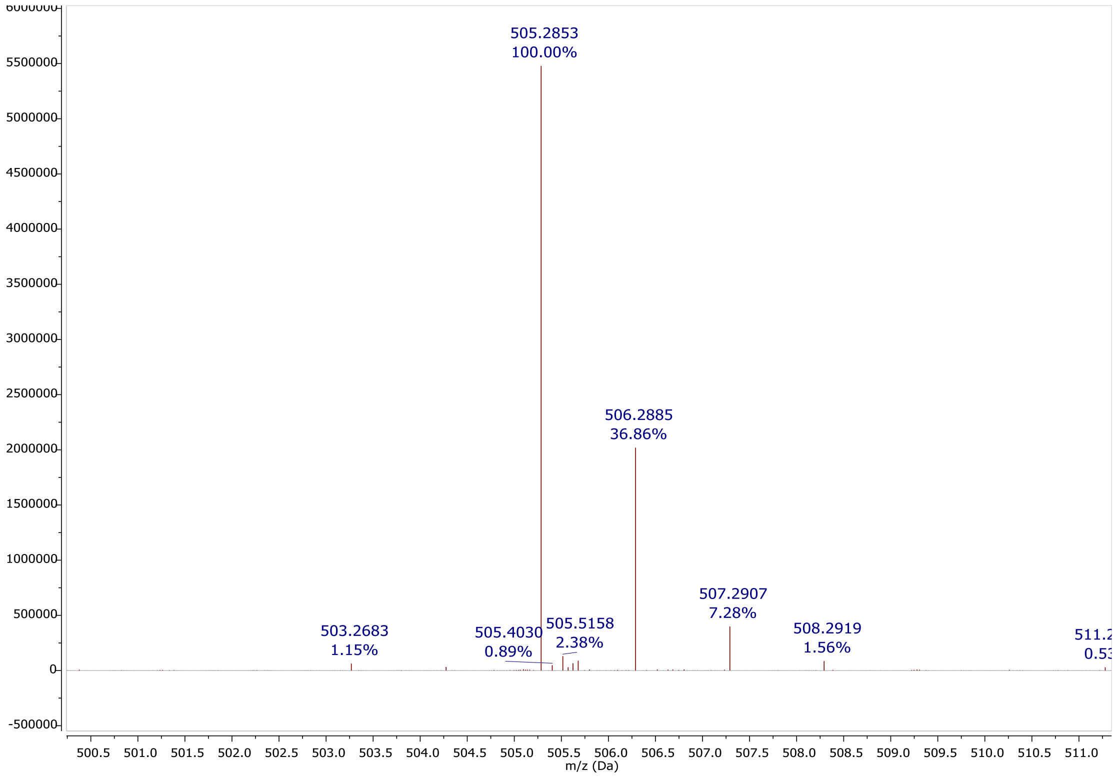


*Figure S109: HRMS spectrum of 4-(2-methoxyphenyl)-2,6-bis(4-(piperidin-1-yl)phenyl)pyrylium trifluoroacetate*

### 3.22. 2,4-NMP-6-Ph - 4,4'-((6-phenylpyrylium-2,4-diyl)bis(4,1-phenylene))bis(1-methylpiperazin-1-ium)

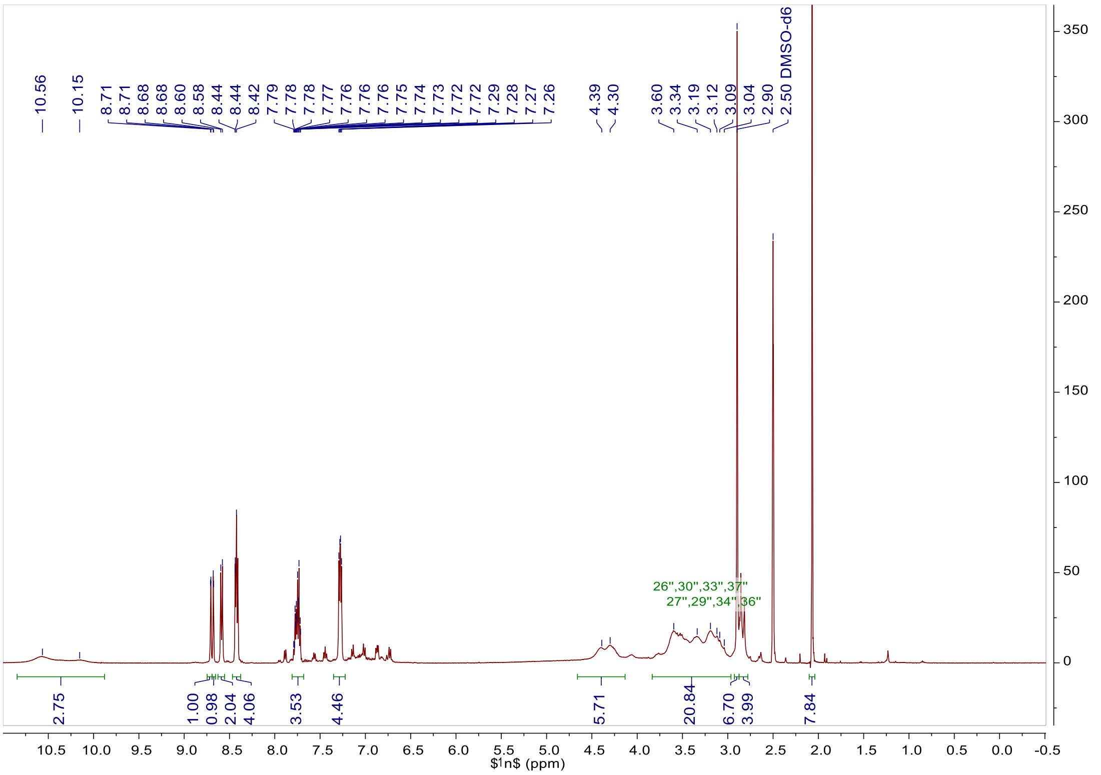

*Figure S110: $^{1}$H-NMR spectrum of 4,4'-((6-phenylpyrylium-2,4-diyl)bis(4,1-phenylene))bis(1-methylpiperazin-1-ium), measured at 293 K in DMSO-$d_6$*

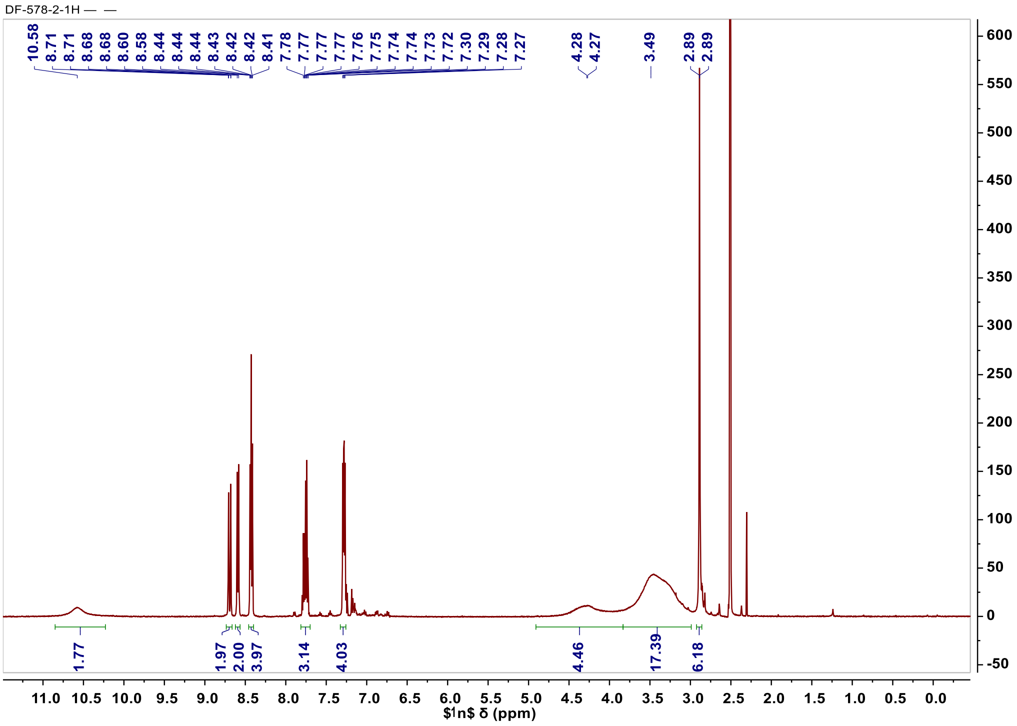

*Figure S111: $^{1}$H-NMR spectrum of 4,4'-((6-phenylpyrylium-2,4-diyl)bis(4,1-phenylene))bis(1-methylpiperazin-1-ium) (further purified for spectroscopical purposes), measured at 293 K in DMSO-$d_6$*

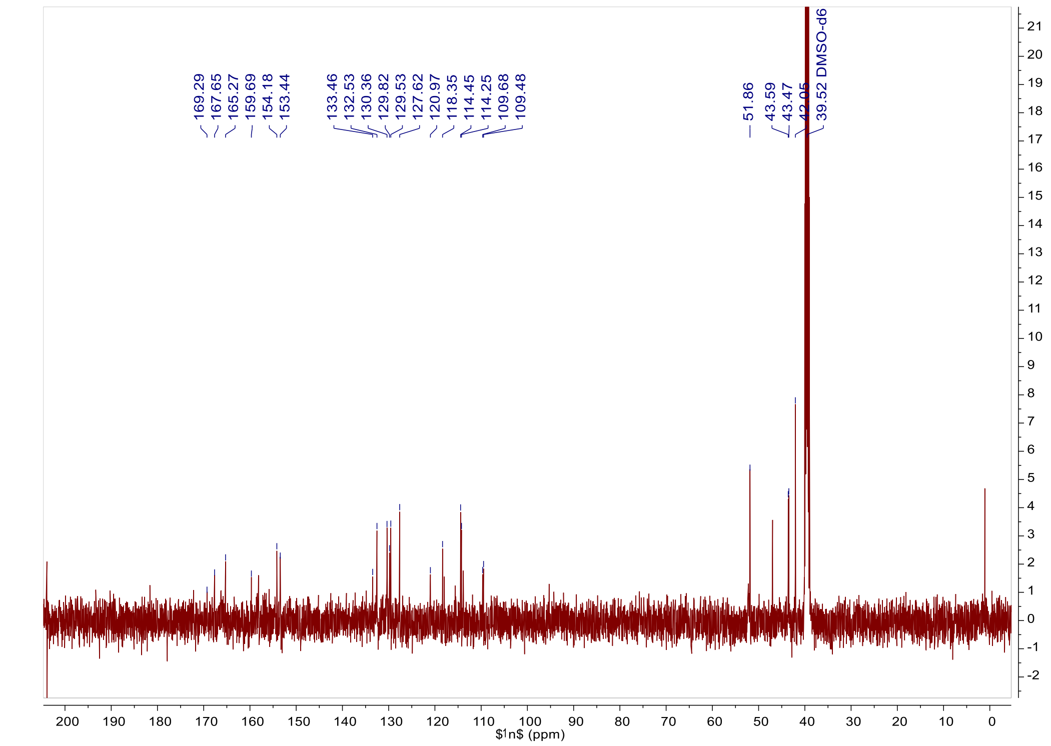


*Figure S112: $^{13}$C-NMR spectrum of 4,4'-((6-phenylpyrylium-2,4-diyl)bis(4,1-phenylene))bis(1-methylpiperazin-1-ium), measured at 293 K in DMSO-$d_6$*

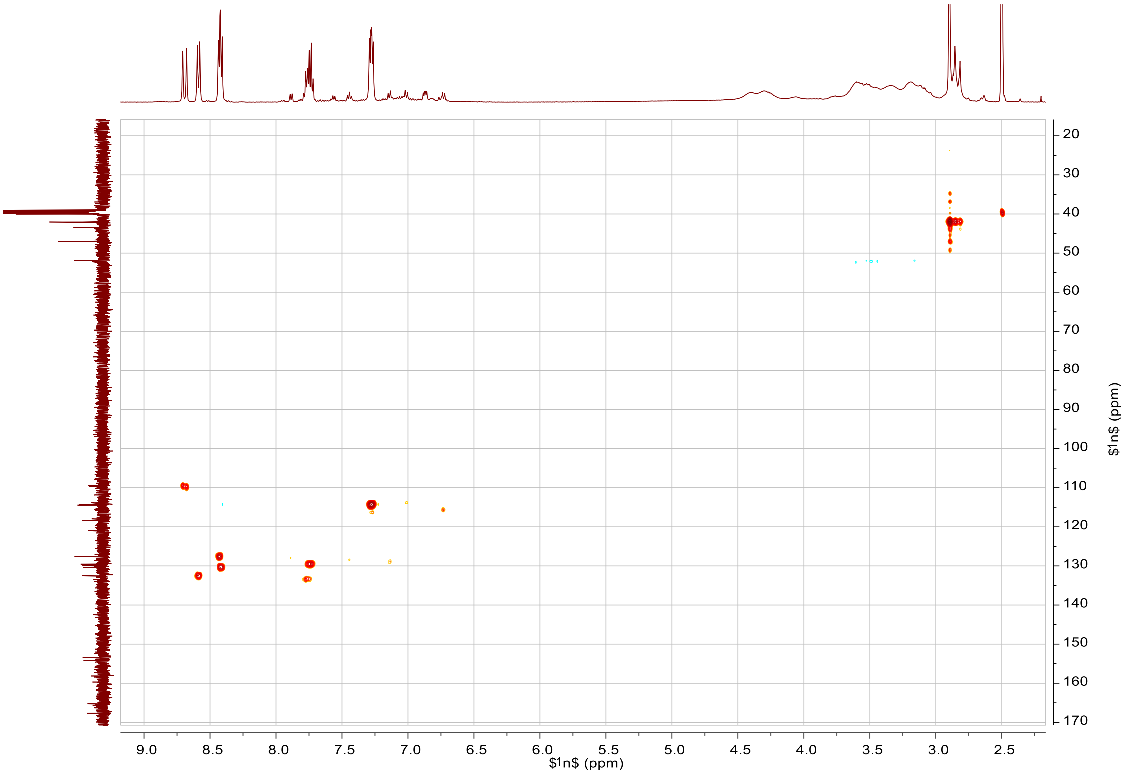


*Figure S113: HSQC spectrum of 4,4'-((6-phenylpyrylium-2,4-diyl)bis(4,1-phenylene))bis(1-methylpiperazin-1-ium), measured at 293 K in DMSO-$d_6$*

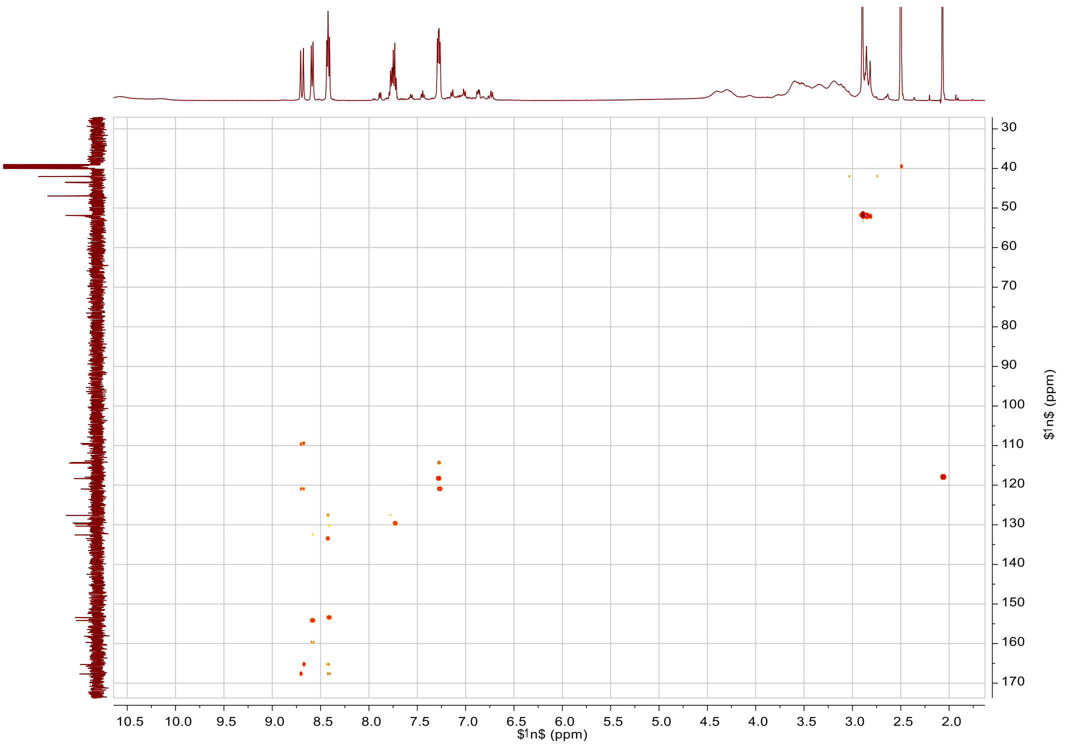


*Figure S114: HMBC spectrum of 4,4'-((6-phenylpyrylium-2,4-diyl)bis(4,1-phenylene))bis(1-methylpiperazin-1-ium), measured at 293 K in DMSO-$d_6$*

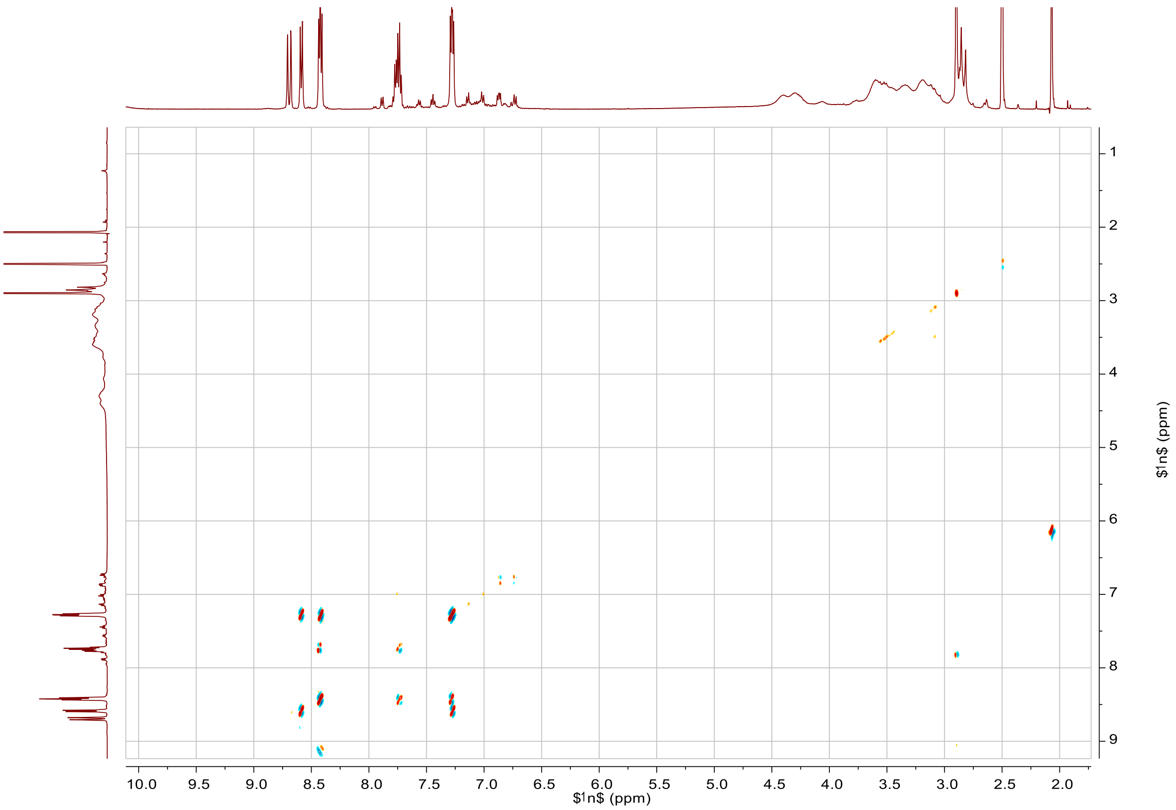


*Figure S115: COSY spectrum of 4,4'-((6-phenylpyrylium-2,4-diyl)bis(4,1-phenylene))bis(1-methylpiperazin-1-ium), measured at 293 K in DMSO-$d_6$*

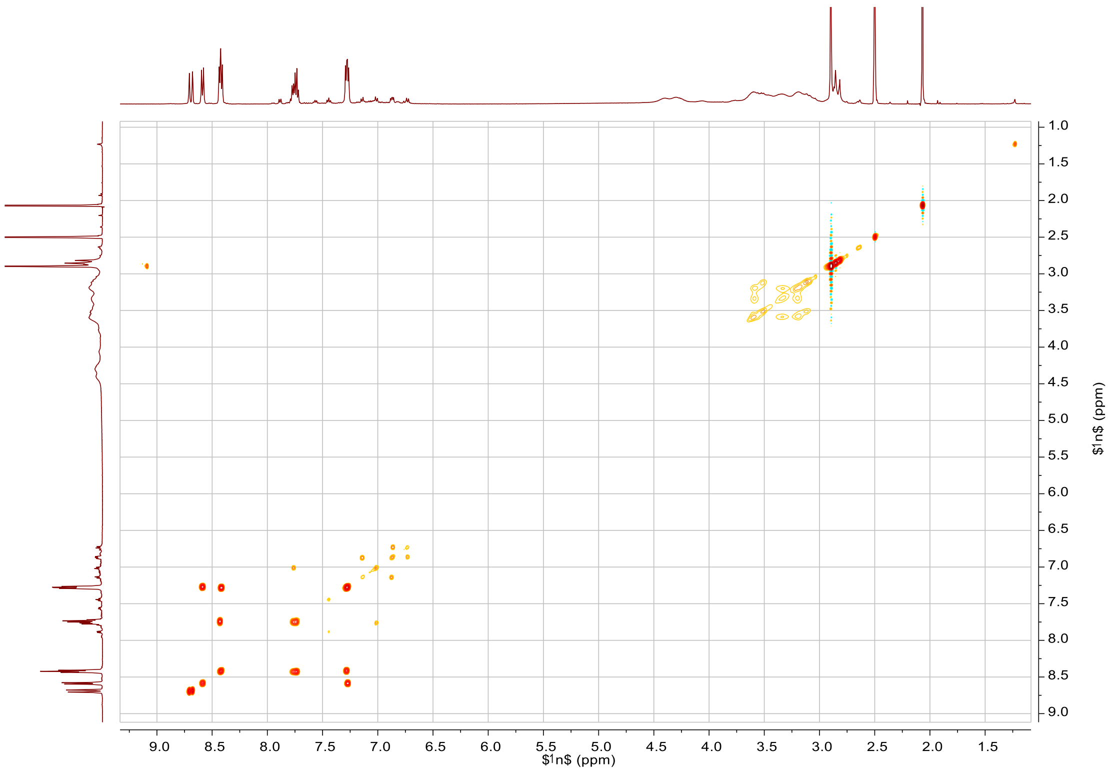


*Figure S116: TOCSY spectrum of 4,4'-((6-phenylpyrylium-2,4-diyl)bis(4,1-phenylene))bis(1-methylpiperazin-1-ium), measured at 293 K in DMSO-$d_6$*

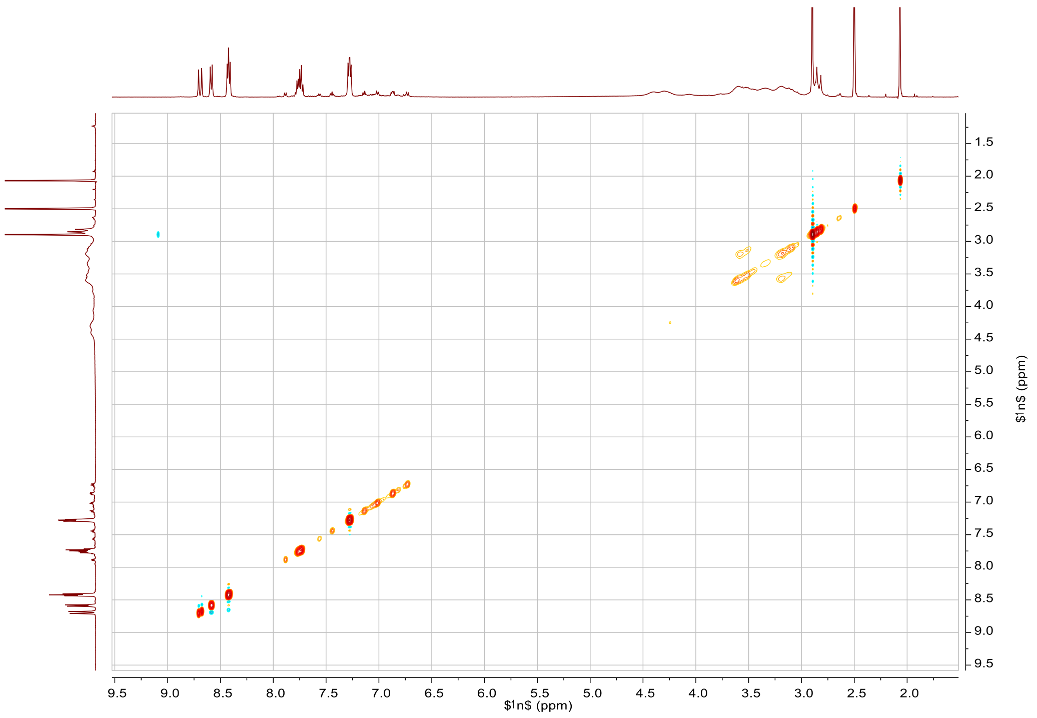


*Figure S117: ROESY spectrum of 4,4'-((6-phenylpyrylium-2,4-diyl)bis(4,1-phenylene))bis(1-methylpiperazin-1-ium), measured at 293 K in DMSO-$d_6$*

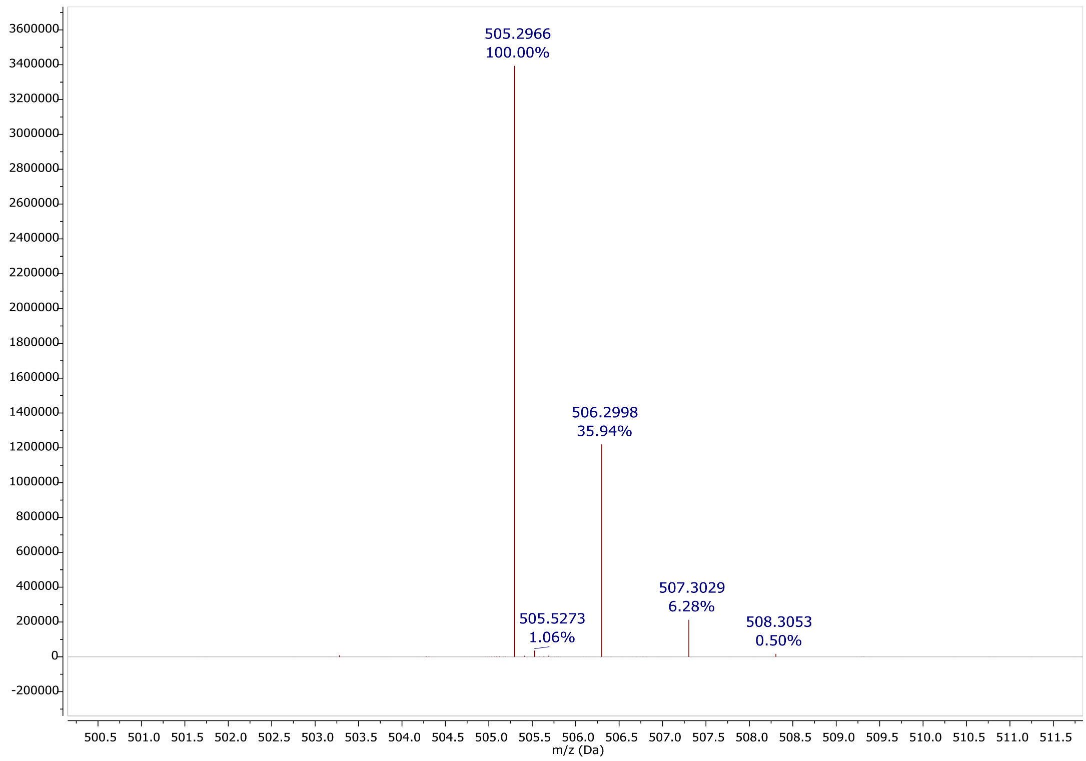


*Figure S118: HRMS spectrum of 4,4'-((6-phenylpyrylium-2,4-diyl)bis(4,1-phenylene))bis(1-methylpiperazin-1-ium)*

## 3.23. 2,4-DMA-6-Me - 2,4-bis(4-(dimethylamino)phenyl)-6-methylpyrylium trifluoroacetate (or perchlorate)

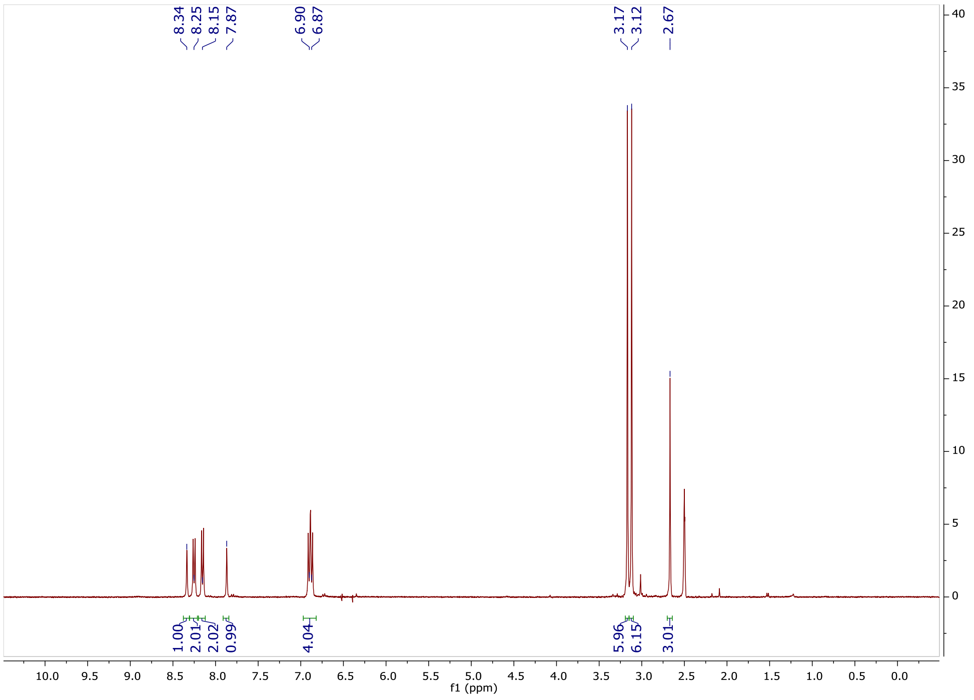


*Figure S119: $^{1}$H-NMR spectrum of 2,4-bis(4-(dimethylamino)phenyl)-6-methylpyrylium trifluoroacetate, measured at 293 K in $CDCl_3$*

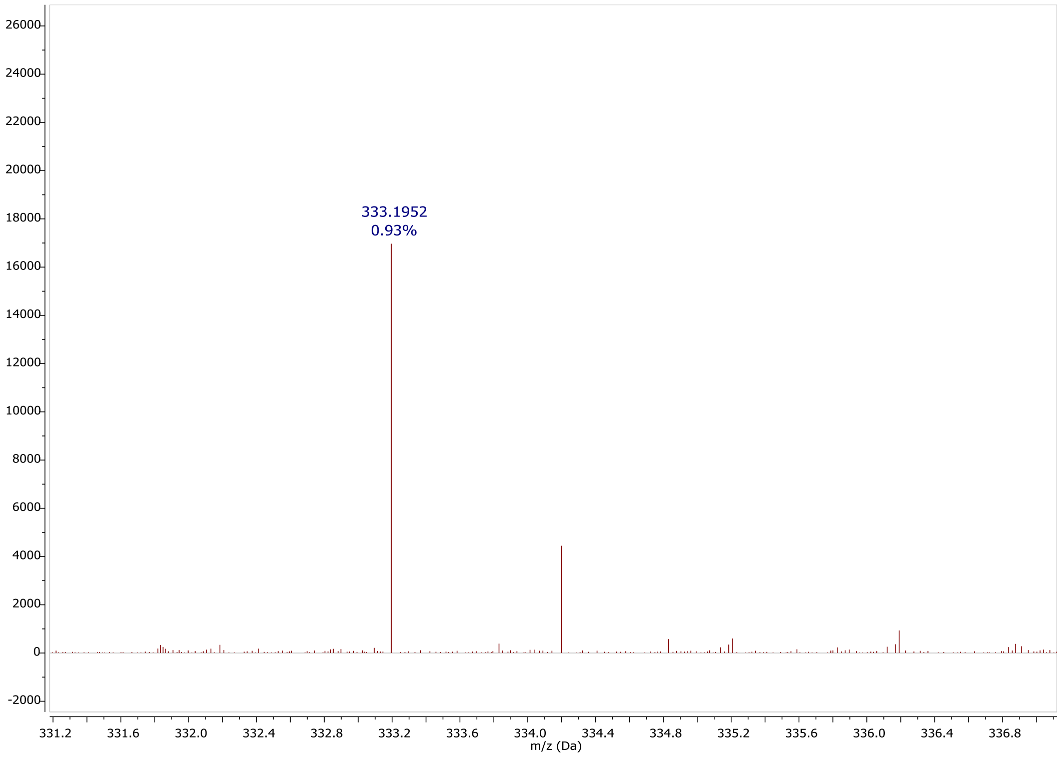


*Figure S120: HRMS spectrum of 2,4-bis(4-(dimethylamino)phenyl)-6-methylpyrylium trifluoroacetate*

## 3.24. 2,4-NMP-6-Me - 4,4'-((6-methylpyrylium-2,4-diyl)bis(4,1-phenylene))bis(1-methylpiperazin-1-ium) tris(trifluoroacetate)

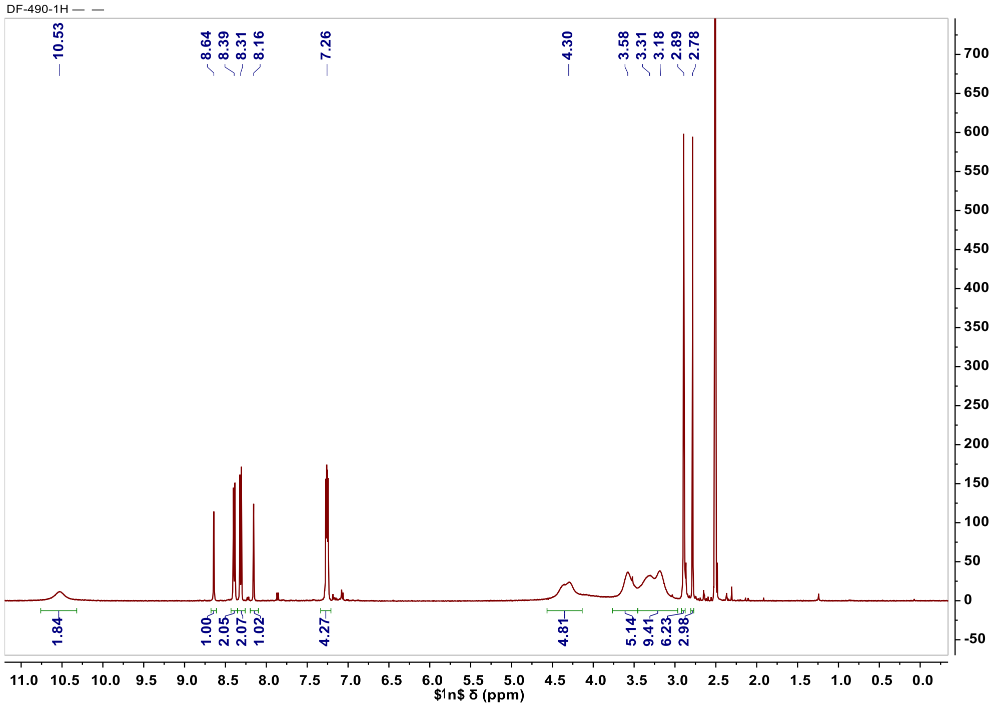


*Figure S121: $^{1}$H-NMR spectrum of 4,4'-((6-methylpyrylium-2,4-diyl)bis(4,1-phenylene))bis(1-methylpiperazin-1-ium) tris(trifluoroacetate), measured at 293 K in DMSO-$d_6$*

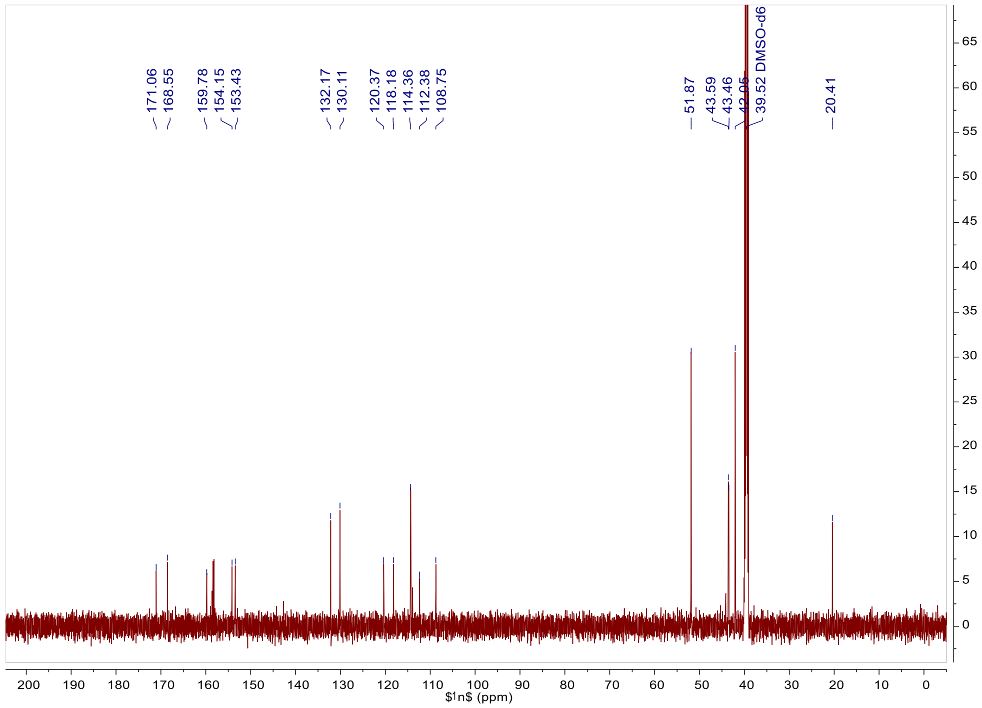


*Figure S122: $^{13}$C-NMR spectrum of 4,4'-((6-methylpyrylium-2,4-diyl)bis(4,1-phenylene))bis(1-methylpiperazin-1-ium) tris(trifluoroacetate), measured at 293 K in DMSO-$d_6$*

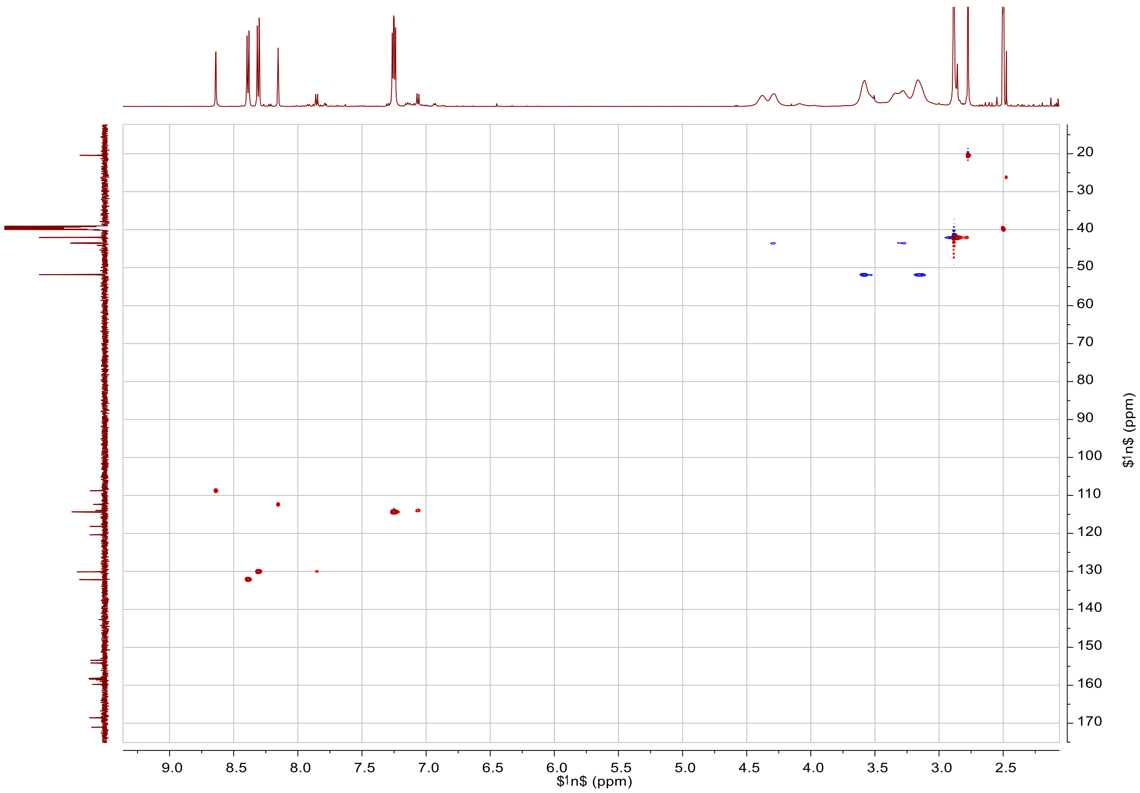


*Figure S123: HSQC spectrum of 4,4'-((6-methylpyrylium-2,4-diyl)bis(4,1-phenylene))bis(1-methylpiperazin-1-ium) tris(trifluoroacetate), measured at 293 K in DMSO-$d_6$*

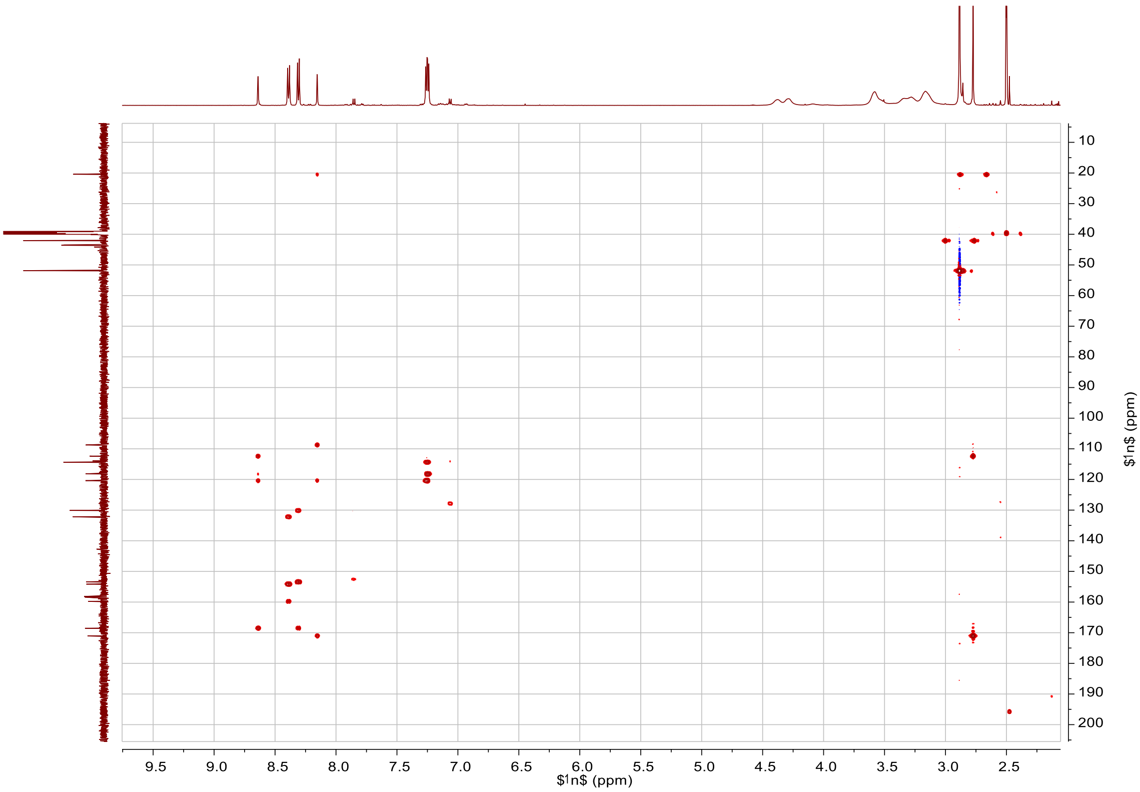


*Figure S124: HMBC spectrum of 4,4'-((6-methylpyrylium-2,4-diyl)bis(4,1-phenylene))bis(1-methylpiperazin-1-ium) tris(trifluoroacetate), measured at 293 K in DMSO-$d_6$*

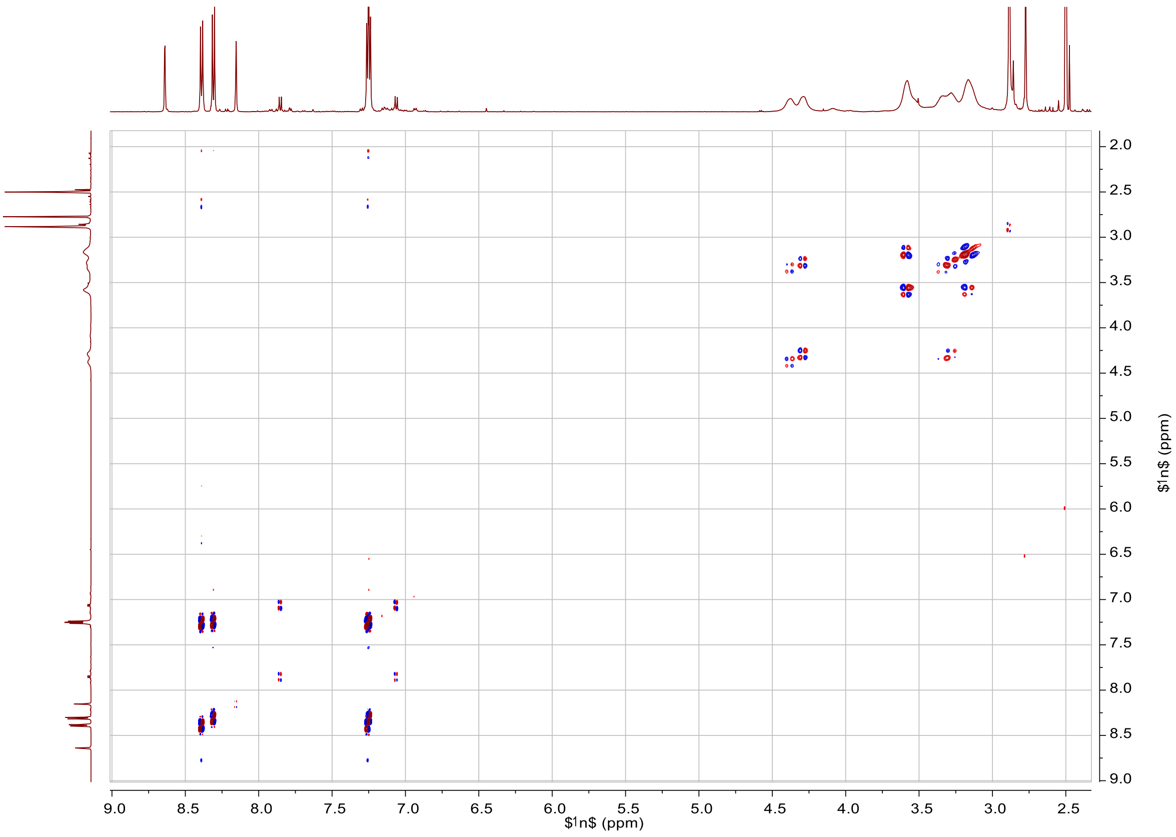


*Figure S125: COSY spectrum of 4,4'-((6-methylpyrylium-2,4-diyl)bis(4,1-phenylene))bis(1-methylpiperazin-1-ium) tris(trifluoroacetate), measured at 293 K in DMSO-$d_6$*

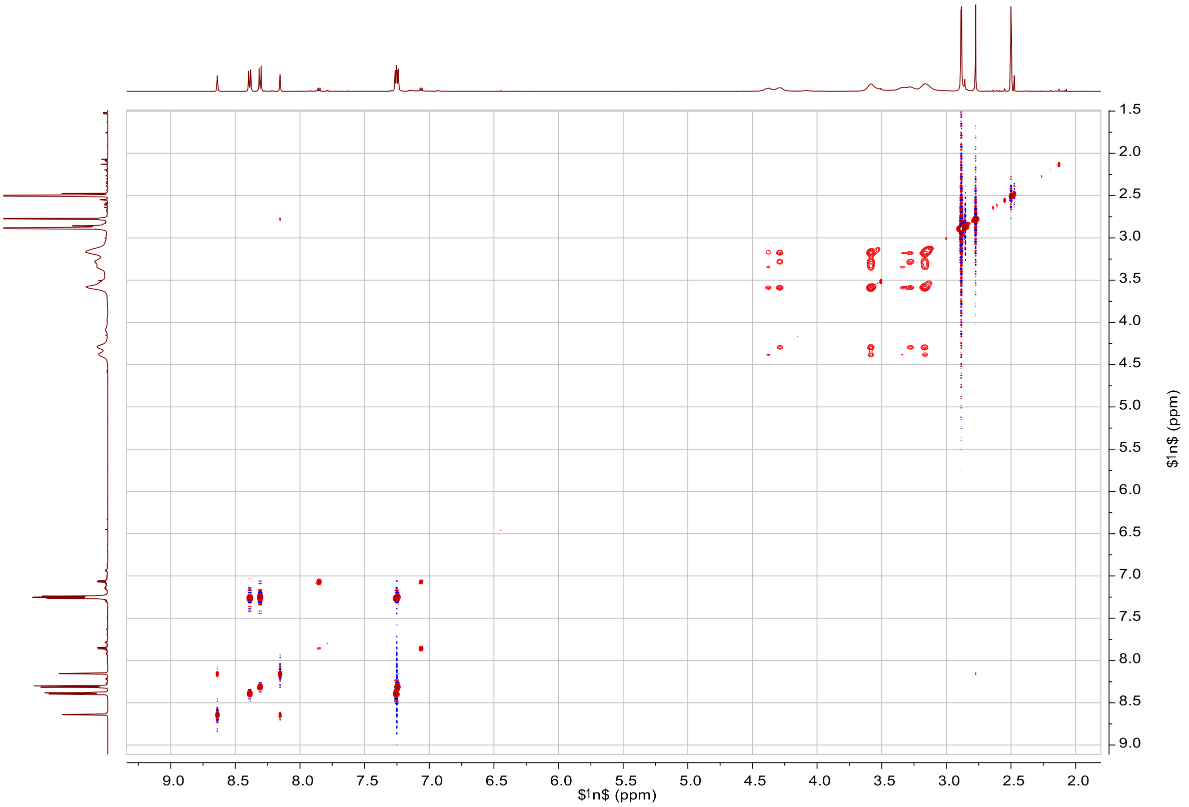


*Figure S126: TOCSY spectrum of 4,4'-((6-methylpyrylium-2,4-diyl)bis(4,1-phenylene))bis(1-methylpiperazin-1-ium) tris(trifluoroacetate), measured at 293 K in DMSO-$d_6$*

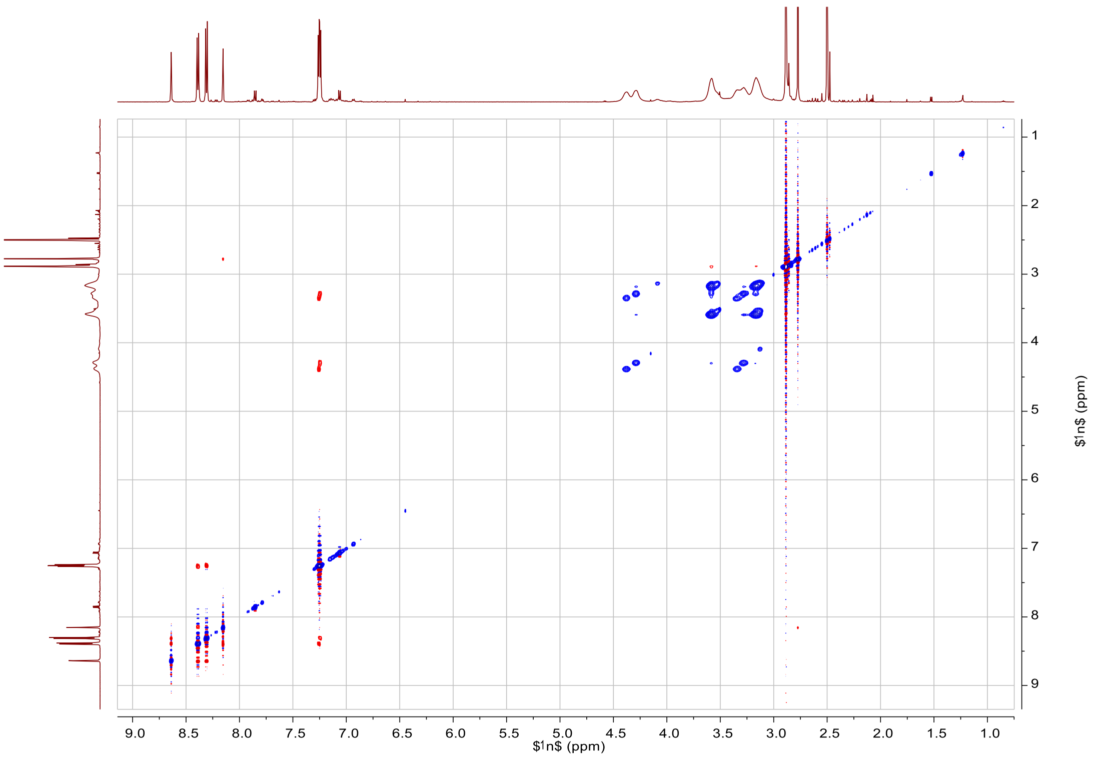


*Figure S127: ROESY spectrum of 4,4'-((6-methylpyrylium-2,4-diyl)bis(4,1-phenylene))bis(1-methylpiperazin-1-ium) tris(trifluoroacetate), measured at 293 K in DMSO-$d_6$*

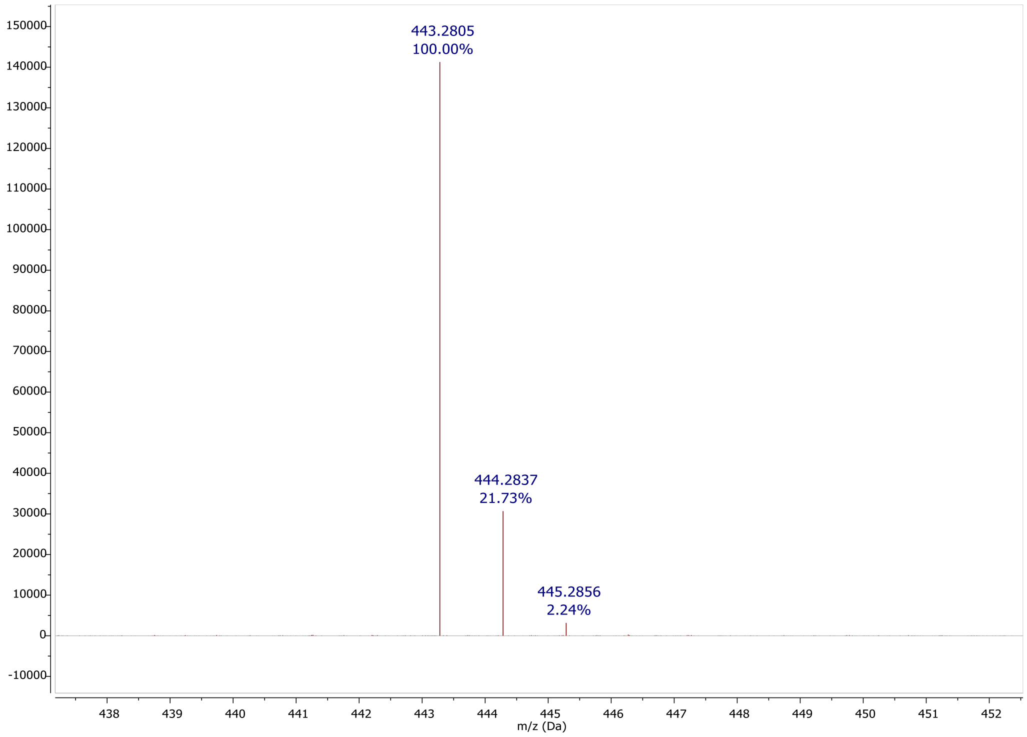


*Figure S128: HRMS spectrum of 4,4'-((6-methylpyrylium-2,4-diyl)bis(4,1-phenylene))bis(1-methylpiperazin-1-ium) tris(trifluoroacetate)*

### 3.25. 2,4-DMA-6-BTA - (Z)-2,4-bis(4-(dimethylamino)phenyl)-6-((3-methylbenzo[d]thiazol-2(3H)-ylidene)methyl)pyrylium trifluoroacetate

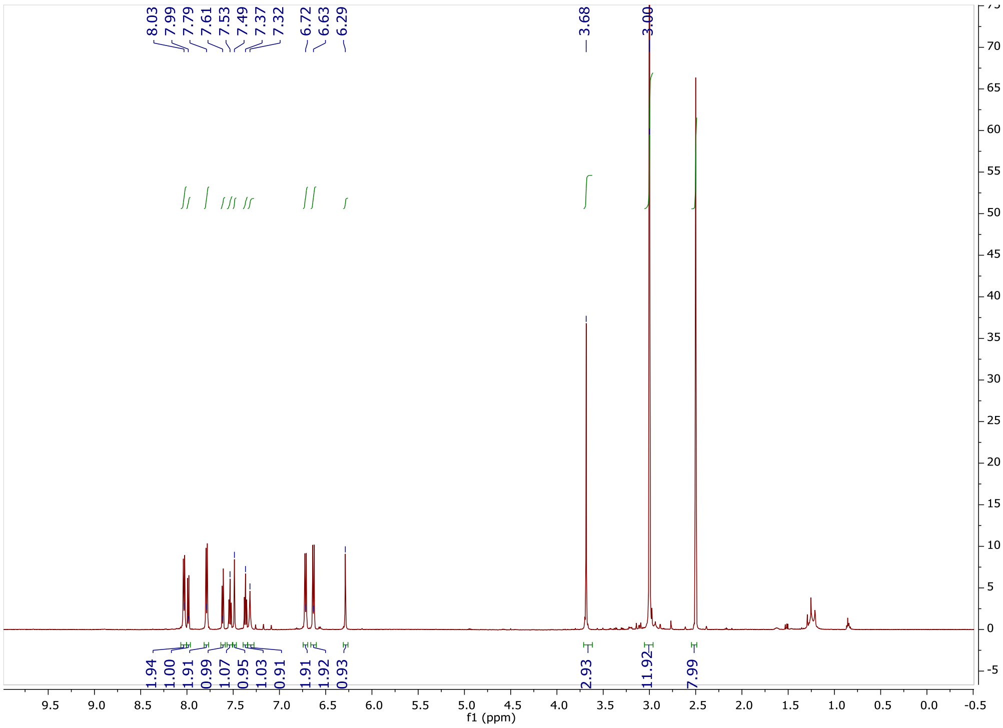

*Figure S129: ¹H-NMR spectrum of (Z)-2,4-bis(4-(dimethylamino)phenyl)-6-((3-methylbenzo[d]thiazol-2(3H)-ylidene)methyl)pyrylium, measured at 293 K in DMSO-$d_6$*

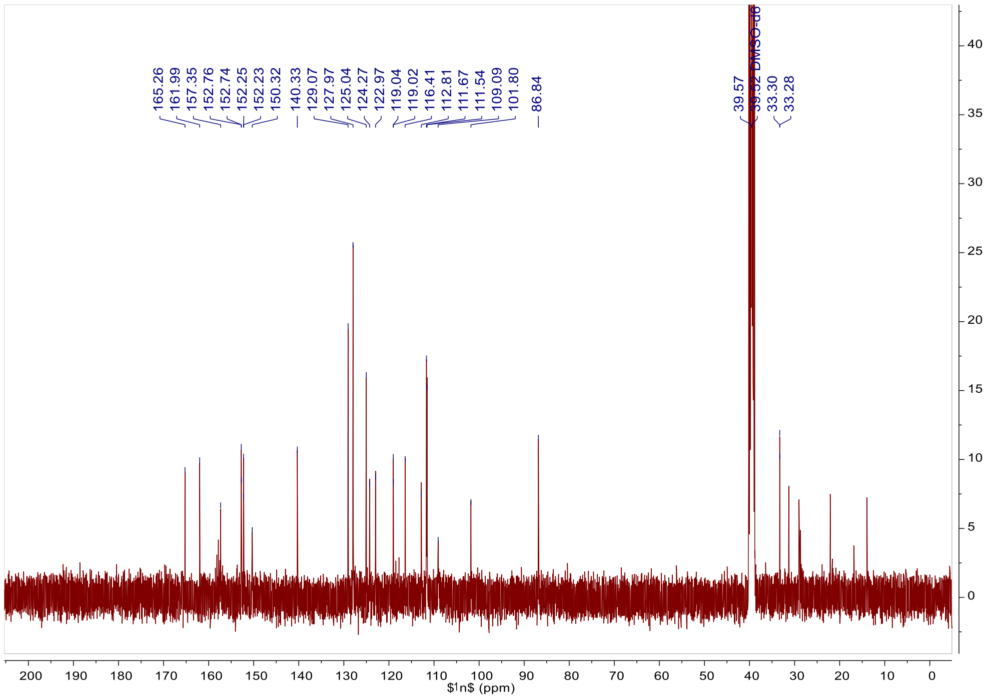

*Figure S130: ¹³C-NMR spectrum of (Z)-2,4-bis(4-(dimethylamino)phenyl)-6-((3-methylbenzo[d]thiazol-2(3H)-ylidene)methyl)pyrylium, measured at 293 K in DMSO-$d_6$*

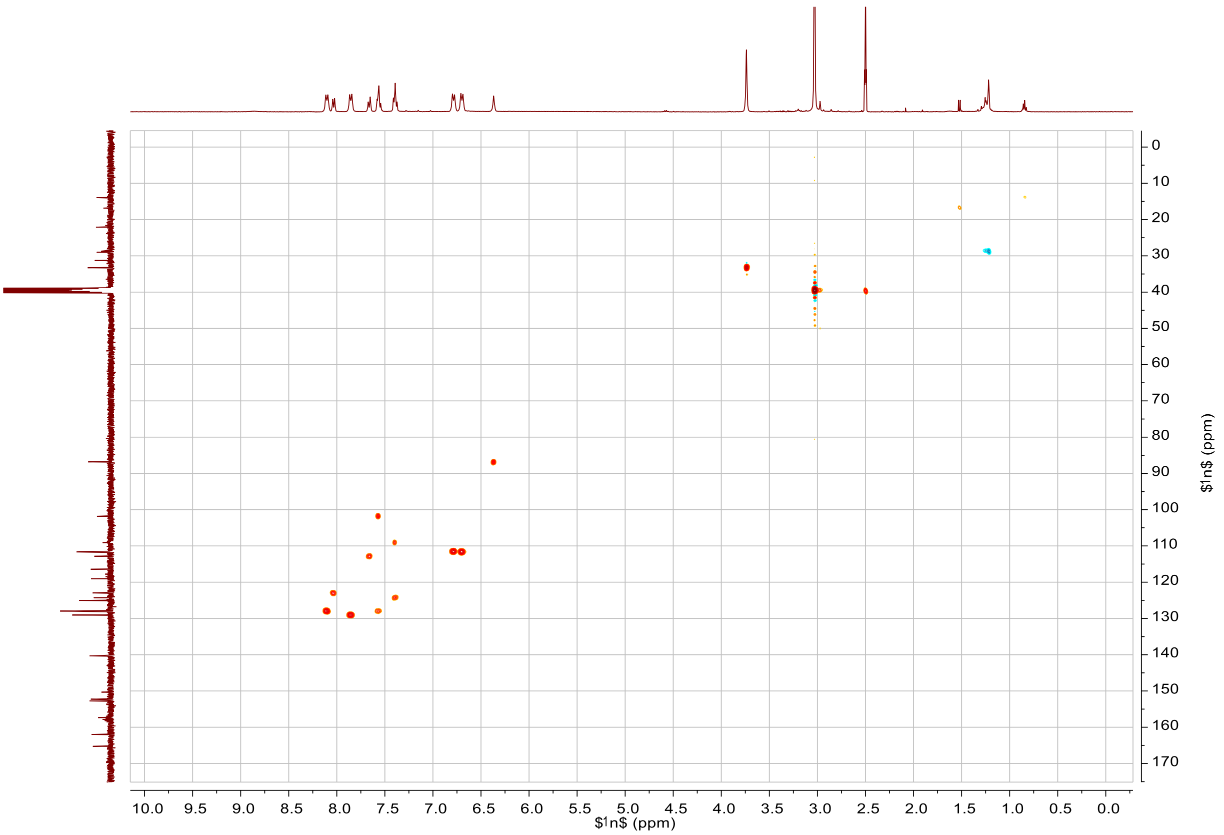


*Figure S131: HSQC spectrum of (Z)-2,4-bis(4-(dimethylamino)phenyl)-6-((3-methylbenzo[d]thiazol-2(3H)-ylidene)methyl)pyrylium, measured at 293 K in DMSO-$d_6$*

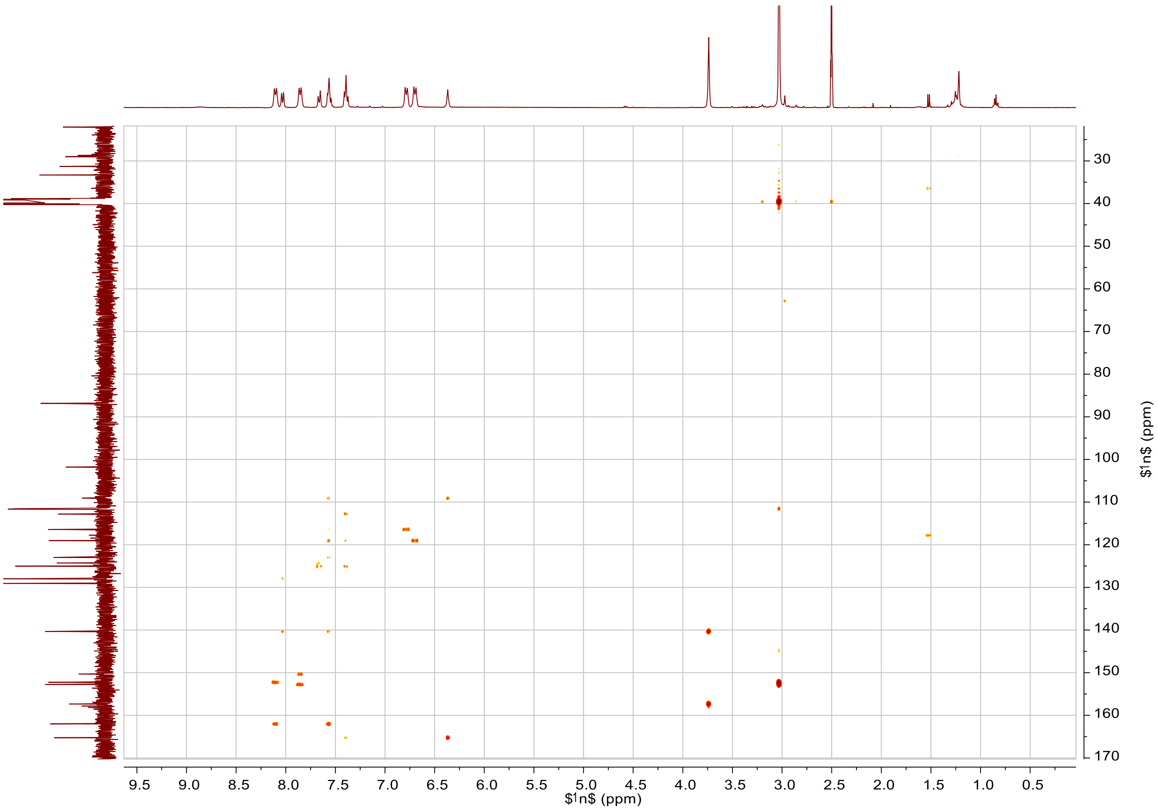


*Figure S132: HMBC spectrum of (Z)-2,4-bis(4-(dimethylamino)phenyl)-6-((3-methylbenzo[d]thiazol-2(3H)-ylidene)methyl)pyrylium, measured at 293 K in DMSO-$d_6$*

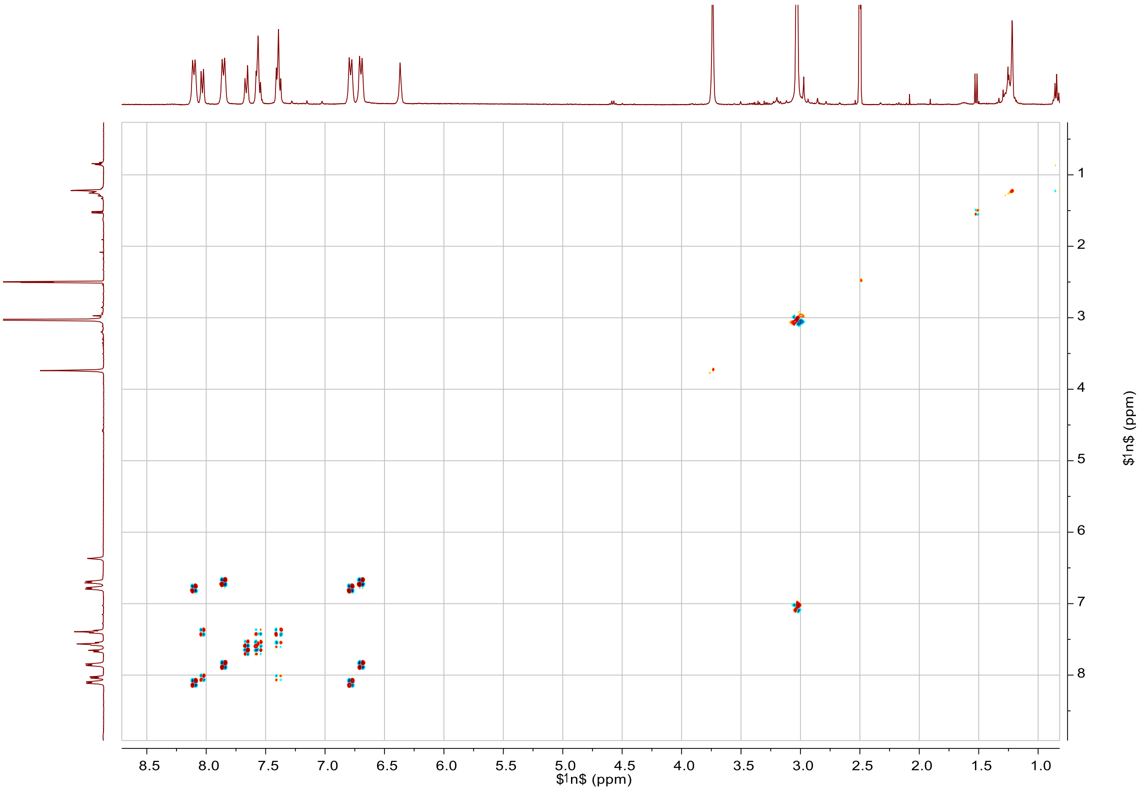


*Figure S133: COSY spectrum of (Z)-2,4-bis(4-(dimethylamino)phenyl)-6-((3-methylbenzo[d]thiazol-2(3H)-ylidene)methyl)pyrylium, measured at 293 K in DMSO-$d_6$*

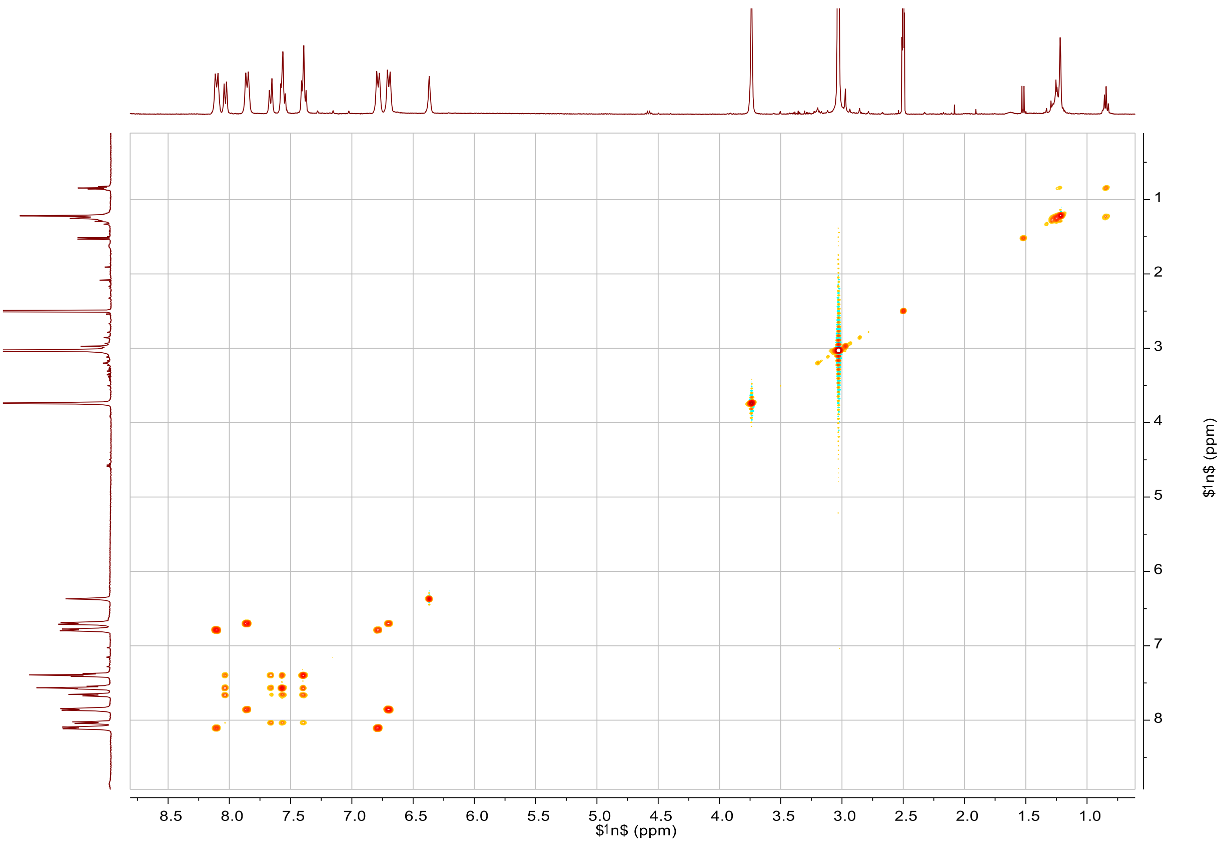


*Figure S134: TOCSY spectrum of (Z)-2,4-bis(4-(dimethylamino)phenyl)-6-((3-methylbenzo[d]thiazol-2(3H)-ylidene)methyl)pyrylium, measured at 293 K in DMSO-$d_6$*

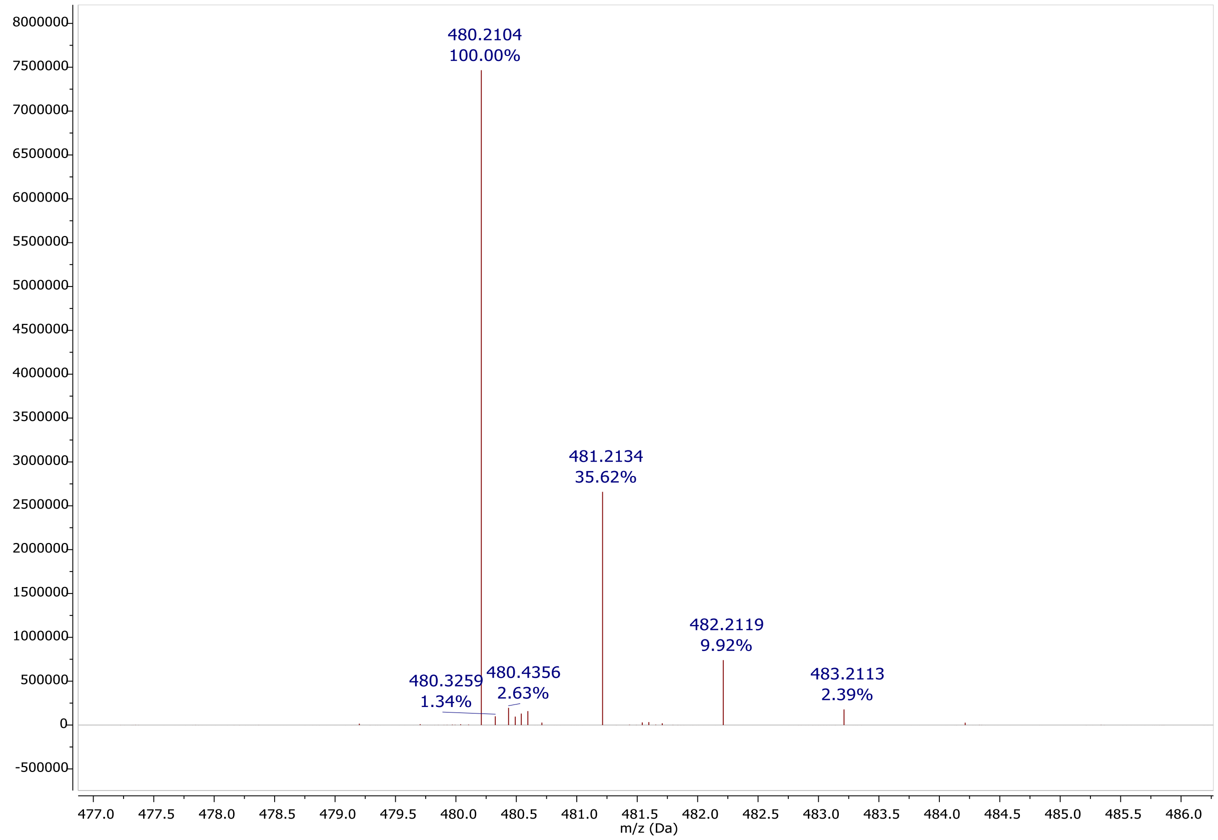


*Figure S135: HRMS spectrum of (Z)-2,4-bis(4-(dimethylamino)phenyl)-6-((3-methylbenzo[d]thiazol-2(3H)-ylidene)methyl)pyrylium*

# 4. Spectroscopic characterization

## 4.1. Summary

Absorption spectra were recorded on a Shimadzu UV-1900i UV-Vis spectrophotometer in 1 cm quartz cuvettes. During the measurements a 2 mM stock solution of the dyes was added to 2 mL TE buffer (Tris-EDTA, pH=7.4, 10 mM Tris-HCl, 1 mM EDTA) in 2 µl increments. The extinction coefficient was calculated from the linear regression of 6 measurements in TE buffer, 4 measurements in acetonitrile, ethylene glycol, methanol, and THF, and from 1 measurement in HFIP, acetone, DMSO, and acetonitrile-ethylene glycol mixtures. In these latter cases the emission spectra were recorded from the same sample to minimise measurement errors during the determination of quantum yield.

Emission spectra were recorded on a Shimadzu RF-6000 spectrofluorimeter. Emission of the dyes in the absence of DNA was measured during the DNA titration experiments (*vide infra*), using 1.0 uM solutions of the dyes in TE buffer (pH = 7.4). Emission of the dye-DNA complex was recorded from a 1 µM solution of the dyes in a 50 µM solution of plasmid DNA in TE buffer (pH = 7.4).

**Table S1:** Extinction coefficients ($\varepsilon^{TE}$, $dm^3$ $mol^{-1}$ $cm^{-1}$), absorption maximum ($\lambda_{abs}$, nm) and emission maximum wavelengths in solution and in dsDNA ($\lambda_{em}^{TE}$, $\lambda_{em}^{DNA}$, nm); fluorescence quantum yields in solution and in dsDNA ($\Phi_f^0$, $\Phi_f^{DNA}$) in pH=7.4 TE buffer. Absolute fluorescence enhancement (AFE, $dm^6$ $cm^{-1}$ $mmol^{-2}$) and relative fluorescence enhancement (RFE, $dm^3$ $\mu mol^{-1}$). Calculated torsional angles between the A (pyrylium) ring and the B, C, or D rings ($\theta_B$, $\theta_C$, $\theta_D$). (n.c.: not calculated)

| Compounds | $\varepsilon^{TE}$ | $\lambda_{abs}$ | $\lambda_{em}^{TE}$ / $\lambda_{em}^{DNA}$ | $\Phi_f^0$ | $\Phi_f^{DNA}$ | AFE | RFE | $\theta_B$ | $\theta_C$ | $\theta_D$ |
|---|---|---|---|---|---|---|---|---|---|---|
| **2,4,6-DMA** | 82 800 | 534 | 733/699 | 0.061 | 0.29 | 31.5 | 0.62 | 13.3 | 20.5 | 13.3 |
| **2,6-NMP-4-DMA** | 64 500 | 530 | 702/629 | 0.074 | 3.56 | 162 | 3.41 | 18.2 | 17.2 | 15.8 |
| **2,6-DMA-4-NMP** | 27 500 | 496 | 725/707 | 0.117 | 0.79 | 63.4 | 1.97 | n.c. | n.c. | n.c. |
| **2,4,6-NMP** | 28 700 | 514 | 684/670 | 0.131 | 1.37 | 103 | 2.74 | 14.1 | 22.8 | 15.1 |
| **2,6-NMP-4-Morph** | 37 900 | 518 | 683/665 | 0.094 | 1.08 | 93.6 | 2.64 | 16.2 | 18.6 | 15.0 |
| **2,6-Ind-4-DMA** | 69 800 | 524 | 714/702 | 0.061 | 0.78 | 125 | 2.94 | 13.3 | 19.8 | 17.1 |
| **2,6-NdMP-4-DMA** | 72 000 | 535 | 640/628 | 0.035 | 2.19 | 169 | 6.66 | 15.9 | 17.5 | 19.0 |
| **2,6-NMP-4-OMe** | 24 900 | 540 | 680/670 | 0.103 | 1.11 | 52.8 | 2.07 | 13.8 | 25.1 | 15.1 |
| **2,6-NMP-4-OEt** | 19 100 | 540 | 684/667 | 0.100 | 1.03 | 55.5 | 2.91 | n.c. | n.c. | n.c. |
| **2,6-NMP-4-OH** | 44 100 | 510 | 674/669 | 0.096 | 0.45 | 25.1 | 0.59 | n.c. | n.c. | n.c. |
| **2,6-NMP-4-2MeOPh** | 24 300 | 550 | 691/671 | 0.097 | 0.87 | 47.8 | 2.03 | 15.3 | 34.3 | 16.4 |
| **2,6-Pip-4-2MeOPh** | 13 200 | 594 | 739/711 | 0.035 | 1.58 | 30.1 | 6.44 | n.c. | n.c. | n.c. |
| **2,4-NMP-6-Ph** | 40 300 | 528 | 685/668 | 0.043 | 0.48 | 37.9 | 2.19 | n.c. | n.c. | n.c. |
| **2,4-DMA-6-Me** | 72 300 | 562 | 656/637 | 0.027 | 3.79 | 434 | 22.3 | 3.7 | 16.5 | - |
| **2,4-NMP-6-Me** | 39 000 | 508 | 616/622 | 0.085 | 2.34 | 103 | 3.11 | 3.6 | 16.4 | - |
| **2,4-DMA-6-BTA** | 19 400 | 484 | 773/684 | 0.129 | 0.97 | 46.2 | 1.85 | n.c. | n.c. | n.c. |

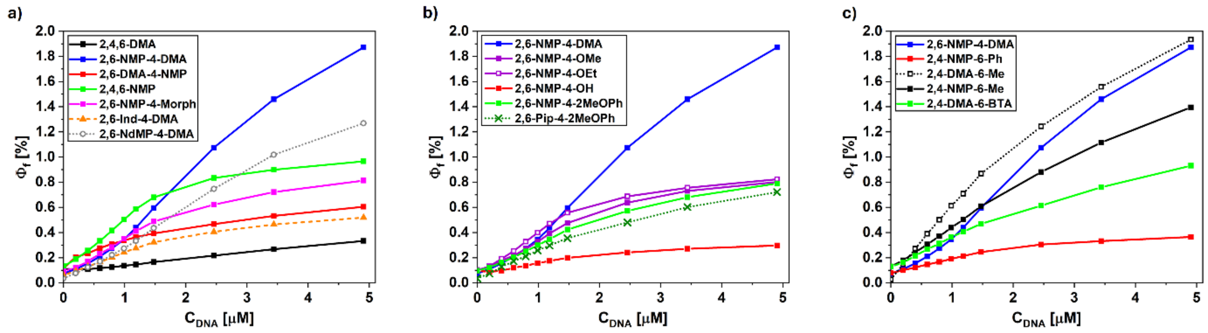


*Figure S136: Dependence of the effective fluorescence quantum yield ($\Phi_f$) of the synthesized pyrylium dyes on the concentration of plasmid DNA.*

**Table S2:** Spectroscopical properties of **2,4,6-DMA** and **2,6-DMA-4-NMP** in various solvents (EG=ethylene glycol; HFIP= 1,1,1,3,3,3-hexafluoropropan-2-ol). Excitation wavelength was 534 nm for **2,4,6-DMA** and 496 nm for **2,6-DMA-4-NMP**, unless stated otherwise.

| | **2,4,6-DMA** | | | | **2,6-DMA-4-NMP** | | | |
|---|---|---|---|---|---|---|---|---|
| | $\lambda_{abs}$ [nm] | $\lambda_{em}$ [nm] | ε [dm$^3$ mol$^{-1}$ cm$^{-1}$] | $\Phi_f$ [%] | $\lambda_{abs}$ [nm] | $\lambda_{em}$ [nm] | ε [dm$^3$ mol$^{-1}$ cm$^{-1}$] | $\Phi_f$ [%] |
| aq. buffer, pH=5 | 512 | 768 | 33200 | 0.108 | 492 | 728 | 49300 | 0.103 |
| aq. buffer, pH=7 | 560 | 765 | 36100 | 0.144 | 494 | 728 | 46200 | 0.103 |
| aq. buffer, pH=8 | 512 | 752 | 37900 | 0.105 | 498 | 727 | 45400 | 0.115 |
| aq. buffer, pH=9 | 568 | 745 | 35500 | 0.057 | 498 | 724 | 46100 | 0.104 |
| aq. buffer, pH=10 | 518 | 761 | 26800 | 0.077 | 500 | 726 | 45700 | 0.101 |
| MeCN | 526 | 690 | 60800 | 2.9 | 496/624 | 707 | 67900 | 1.07 |
| MeCN + 0.2% DIPEA | - | - | 62800 | - | 518 | 700 | 67400 | 1.71 |
| MeCN/EG 3:1 | 530 | 696 | 63400 | 2.59 | 518 | 704 | 57500 | 1.56 |
| MeCN/EG 2:2 | 532 | 695 | 65700 | 2.47 | 518 | 705 | 72300 | 1.66 |
| MeCN/EG 3:1 | 536 | 696 | 61000 | 2.47 | 518 | 706 | 60500 | 1.64 |
| EG | 540 | 692 | 59500 | 3.394 | 520 | 704 | 68500 | 2.43 |
| MeOH | 528 | 680 | 67100 | 2.758 | 514 | 696 | 81300 | 1.76 |
| MeOH + 0.2% TFA | - | - | - | - | 494/594 | 704 | 69900 | 1.26 |
| MeOH + 0.2% DIPEA | - | - | - | - | 496 | 693 | 81600 | 1.94 |
| THF | 534 | 677 | 61800 | 12.18 | 528 | 687 | 85000 | 8.15 |
| acetone | 530 | 689 | 62000 | 3.127 | - | - | - | - |
| DMSO | 542 | 713 | 59700 | 2.885 | - | - | - | - |
| HFIP | 560 | 683 | 47800 | 2.439 | 480/604 | 685/688 [1] | 43000/46300 | 2.63/2.48 [1] |

[1] $\lambda_{ex}$=480/604 nm

**Table S3:** Spectroscopical properties of **2,6-NMP-4-DMA** and **2,4,6-NMP** in various solvents (EG=ethylene glycol; HFIP= 1,1,1,3,3,3-hexafluoropropan-2-ol). Excitation wavelength was 524 nm for **2,6-NMP-4-DMA** and 514 nm for **2,4,6-NMP**, unless stated otherwise.

| | **2,6-NMP-4-DMA** | | | | **2,4,6-NMP** | | | |
|---|---|---|---|---|---|---|---|---|
| | $\lambda_{abs}$ [nm] | $\lambda_{em}$ [nm] | ε [$dm^3$ $mol^{-1}$ $cm^{-1}$] | $\Phi_f$ [%] | $\lambda_{abs}$ [nm] | $\lambda_{em}$ [nm] | ε [$dm^3$ $mol^{-1}$ $cm^{-1}$] | $\Phi_f$ [%] |
| aq. buffer, pH=5 | 534 | 665 | 62800 | 0.064 | - | - | - | - |
| aq. buffer, pH=7 | 532 | 678 | 67900 | 0.059 | - | - | - | - |
| aq. buffer, pH=8 | 522 | 699 | 58300 | 0.053 | - | - | - | - |
| aq. buffer, pH=9 | 516 | 699 | 61300 | 0.062 | - | - | - | - |
| aq. buffer, pH=10 | 516 | 701 | 57400 | 0.046 | - | - | - | - |
| MeCN | 542 | 692 | 102700 | 0.240 | 514 | 677 | 37400 | 2.58 |
| MeCN + 0.2% DIPEA | 538 | 698 | 101312 | 0.227 | 530 | 706 | 40400 | 0.175 |
| MeCN/EG 3:1 | 544 | 696 | 113147 | 0.475 | 536 | 706 | 43700 | 0.377 |
| MeCN/EG 2:2 | 546 | 694 | 111997 | 0.690 | 536 | 703 | 44900 | 0.598 |
| MeCN/EG 3:1 | 550 | 688 | 114825 | 1.08 | 540 | 703 | 44800 | 0.985 |
| EG | 552 | 684 | 107333 | 1.70 | 542 | 696 | 41500 | 1.54 |
| MeOH | 544 | 676 | 103700 | 0.544 | - | - | - | - |
| THF | 544 | 679 | 105900 | 0.910 | - | - | - | - |
| HFIP | 548 | 632[1] | 108600 | 0.156[1] | - | - | - | - |

[1]$\lambda_{ex}$=540 nm

## 4.2. Absorption and emission spectra in organic solvents

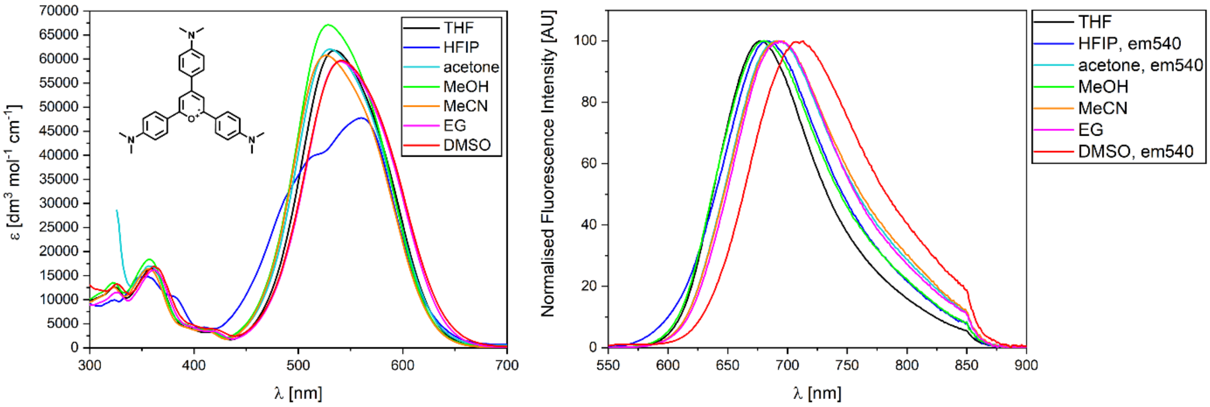


*Figure S137 Absorption and normalised emission spectra of **2,4,6-DMA** in various organic solvents (EG = ethylene glycol; HFIP = 1,1,1,3,3,3-hexafluoroisopropanol).*

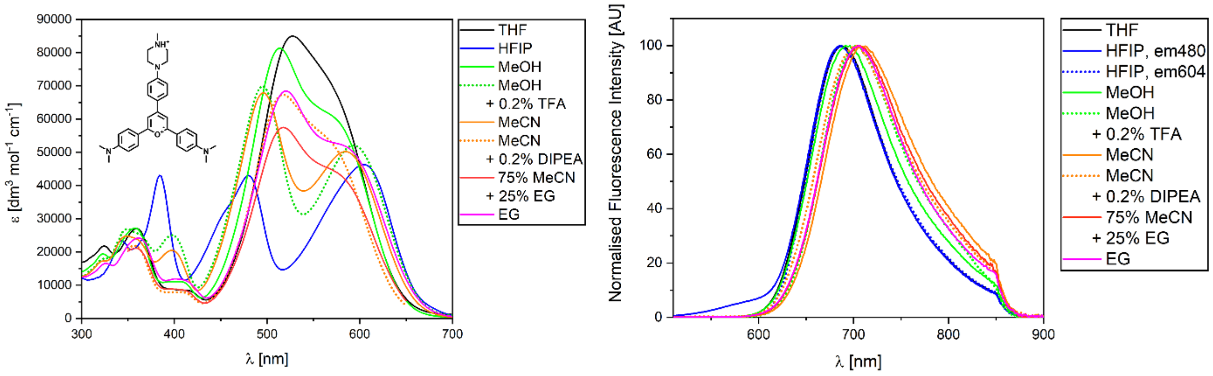


*Figure S138: Absorption and normalised emission spectra of* ***2,6-DMA-4-NMP*** *in various organic solvents (DIPEA = diisopropylethylamine; EG = ethylene glycol; HFIP = 1,1,1,3,3,3-hexafluoroisopropanol; TFA=trifluoroacetic acid).*

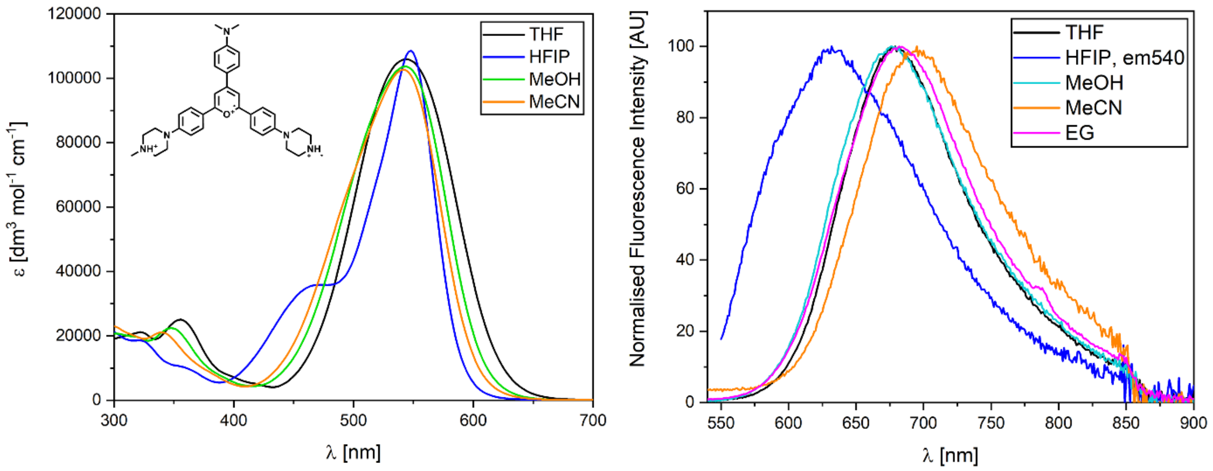


*Figure S139: Absorption and normalised emission spectra of* ***2,6-NMP-4-DMA*** *in various organic solvents (EG = ethylene glycol; HFIP = 1,1,1,3,3,3-hexafluoroisopropanol).*

## Viscosity dependence of the fluorescence quantum yield

The dependence of fluorescence quantum yield ($\Phi_f$) of **2,4,6-DMA**, **2,6-DMA-4-NMP**, **2,6-NMP-4-DMA**, and **2,4,6-NMP** on solvent viscosity was measured in mixtures of MeCN and ethylene glycol, a pair of solvents with different viscosity and almost identical relative permittivity[8] (see also Table S2–3). Quantum yields were also measured in MeCN containing 0.2% diisopropylethylamine (DIPEA).

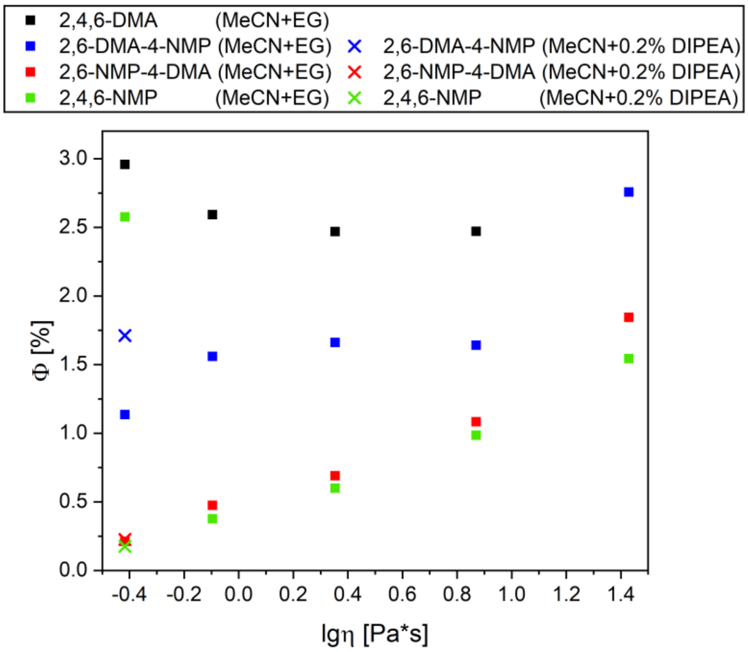


*Figure S140: Dependence of fluorescence quantum yields ($\Phi_f$) of **2,4,6-DMA**, **2,6-DMA-4-NMP**, **2,6-NMP-4-DMA**, and **2,4,6-NMP** on the dynamic viscosity acetonitrile-ethylene glycol mixtures (η, depicted here on a logarithmic scale).*

## 4.3. The effect of pH on spectroscopic properties

Absorbtion and emission spectra were recorded from 1, 2, 3, and 4 µM solutions in the following buffers:

- pH=5: acetic acid/potassium acetate buffer (ionic strength: 100 mM; measured pH: 5.01)
- pH=7: HEPES/HEPES potassium salt buffer (ionic strength: 100 mM; measured pH: 7.02)
- pH=8: HEPES/HEPES potassium salt buffer (ionic strength: 100 mM; measured pH: 8.05)
- pH=9: boric acid/K borate buffer (ionic strength: 100 mM; measured pH: 9.02)
- pH=10: boric acid/K borate buffer (ionic strength: 100 mM; measured pH: 10.05)

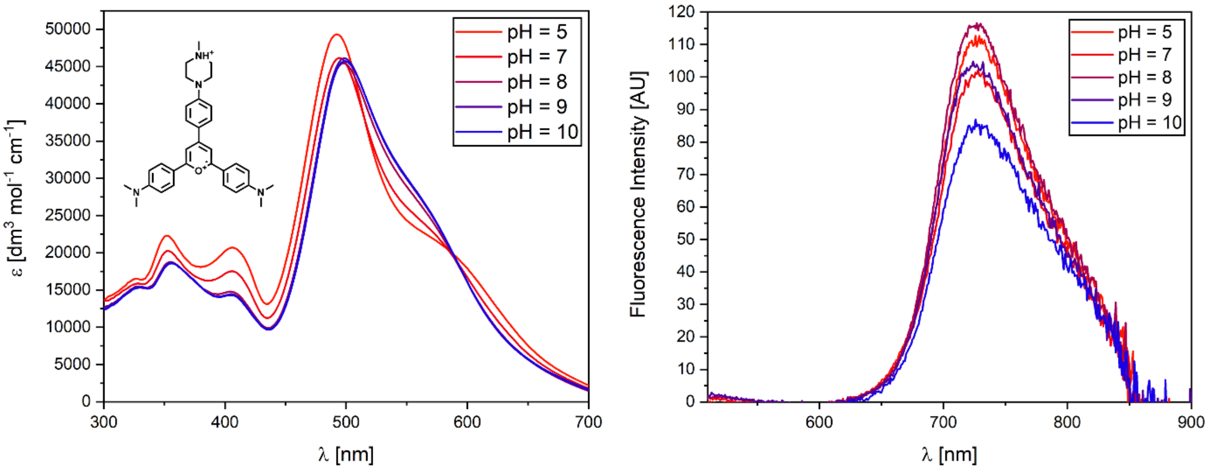


*Figure S141: Absorption and normalised emission spectra of **2,6-DMA-4-NMP** in aqueous buffers with varying pH.*

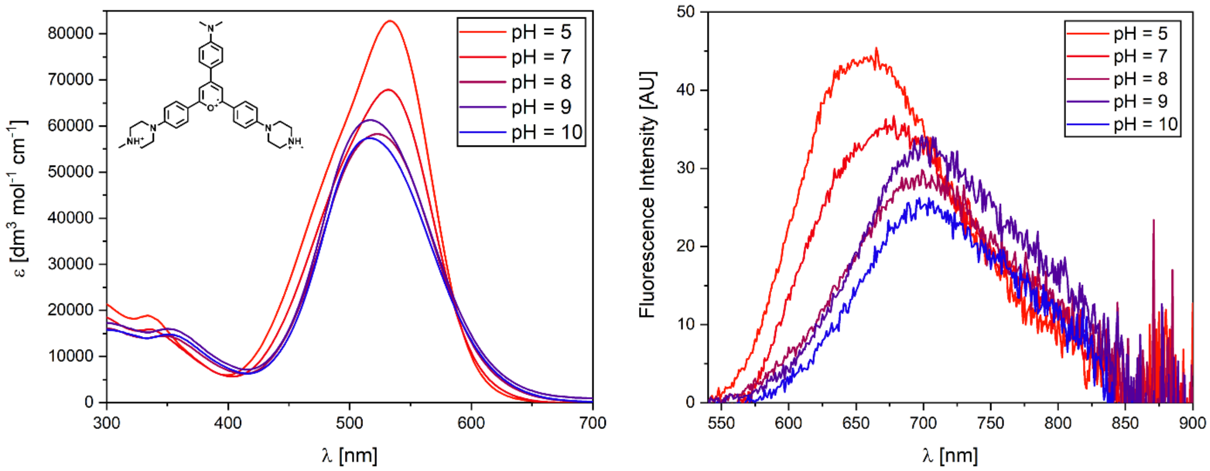


*Figure S142: Absorption and normalised emission spectra of* ***2,6-NMP-4-DMA*** *in aqueous buffers with varying pH.*

## 4.4. Study of aggregation (concentration dependence of absorption spectra in TE buffer)

The concentration dependence of the absorption spectra of **2,4,6-DMA**, **2,6-DMA-4-NMP**, and **2,6-NMP-4-DMA** was measured in pH=7.4 TE buffer in the 1–5 μM concentration range. While the spectrum of **2,4,6-DMA** exhibited significant batochromic shift, the spectra of **2,6-DMA-4-NMP** and **2,6-NMP-4-DMA** displayed slighter change, referring to a lower concentration of aggregates.

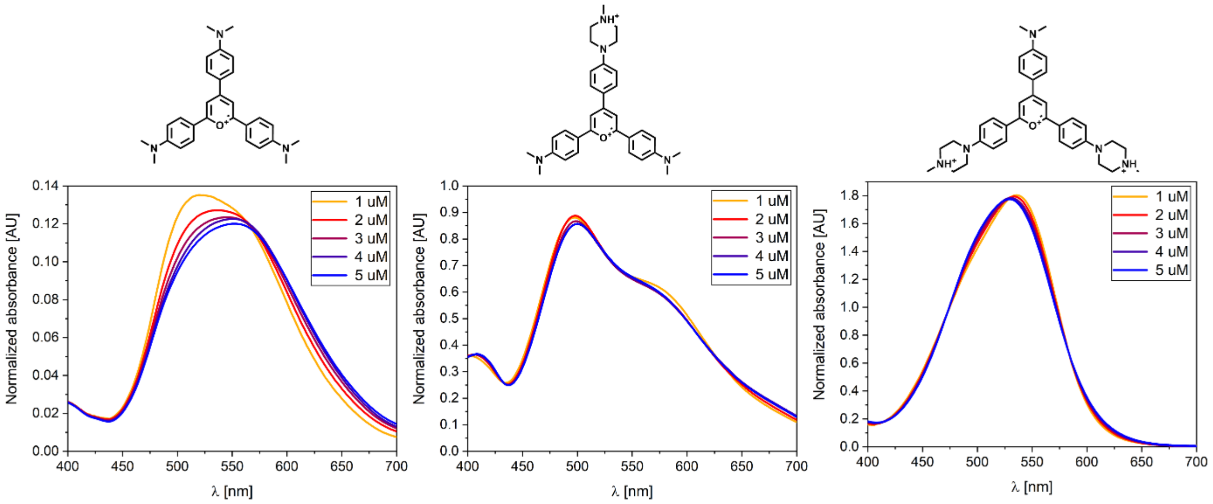


*Figure S143. Concentration dependence of the area-normalized absorption spectra of* ***2,4,6-DMA*** *(left)* ***2,6-DMA-4-NMP*** *(middle) and* ***2,6-NMP-4-DMA*** *(right).*

## 4.5. Absorption and emission spectra in TE buffer and in the presence of DNA

### 2,4,6-DMA

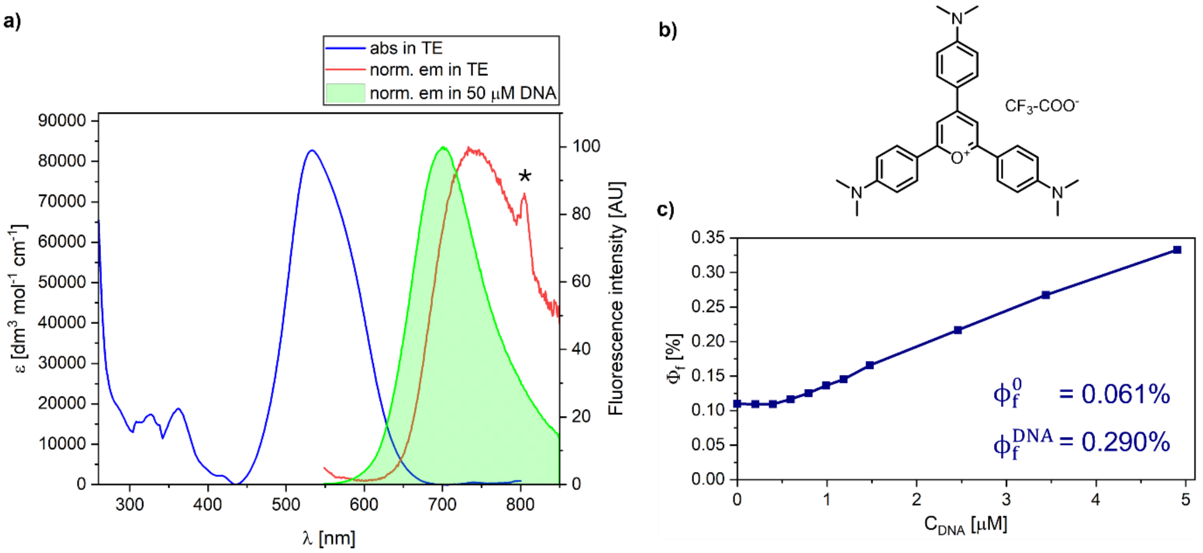


*Figure S144:* ***2,4,6-DMA*** */ 2,4,6-tris(4-(dimethylamino)phenyl)pyrylium trifluoroacetate. a) Absorption in pH=7.4 TE-SDS buffer, normamised emission in TE buffer and in the presence of 50 µM plasmid DNA ($\lambda_{ex}$ = 534 nm). The peak marked with an asterisk at $\lambda = 3\lambda_{ex}/2$ is an aritifact due to the scattering of stray light from the monochromator. b) Structural formula. c) Fluorescence quantum yield in the presence of plasmid DNA in varying concentrations; $\phi_f^0$ and $\phi_f^{DNA}$ are the fluorescence quantum yield at 0 and 50 µM plasmid DNA, respectively.*

### 2,6-NMP-4-DMA

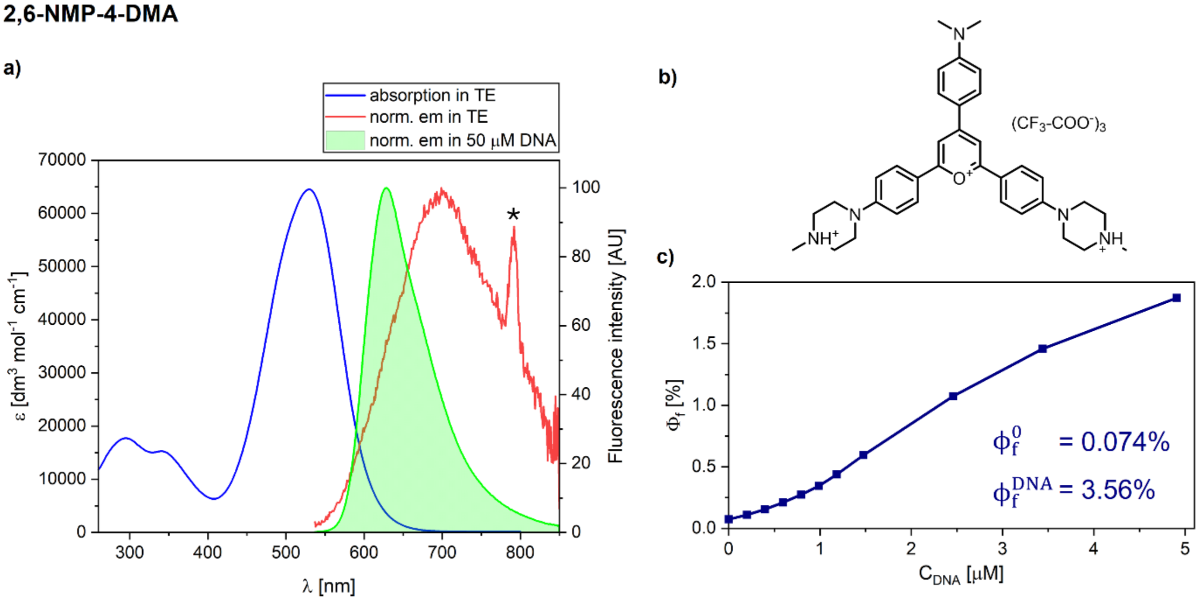


*Figure S145:* ***2,6-NMP-4-DMA*** */ 4,4'-((4-(4-(dimethylamino)phenyl)pyrylium-2,6-diyl)bis(4,1-phenylene))bis(1-methylpiperazin-1-ium) tris(trifluoroacetate). a) Absorption in pH=7.4 TE buffer, normalised emission in TE buffer and in the presence of 50 µM plasmid DNA ($\lambda_{ex}$ = 524 nm). The peak marked with an asterisk at $\lambda = 3\lambda_{ex}/2$ is an aritifact due to the scattering of stray light from the monochromator. b) Structural formula. c) Fluorescence quantum yield in the presence of plasmid DNA in varying concentrations; $\phi_f^0$ and $\phi_f^{DNA}$ are the fluorescence quantum yield at 0 and 50 µM plasmid DNA, respectively.*

**2,6-DMA-4-NMP**

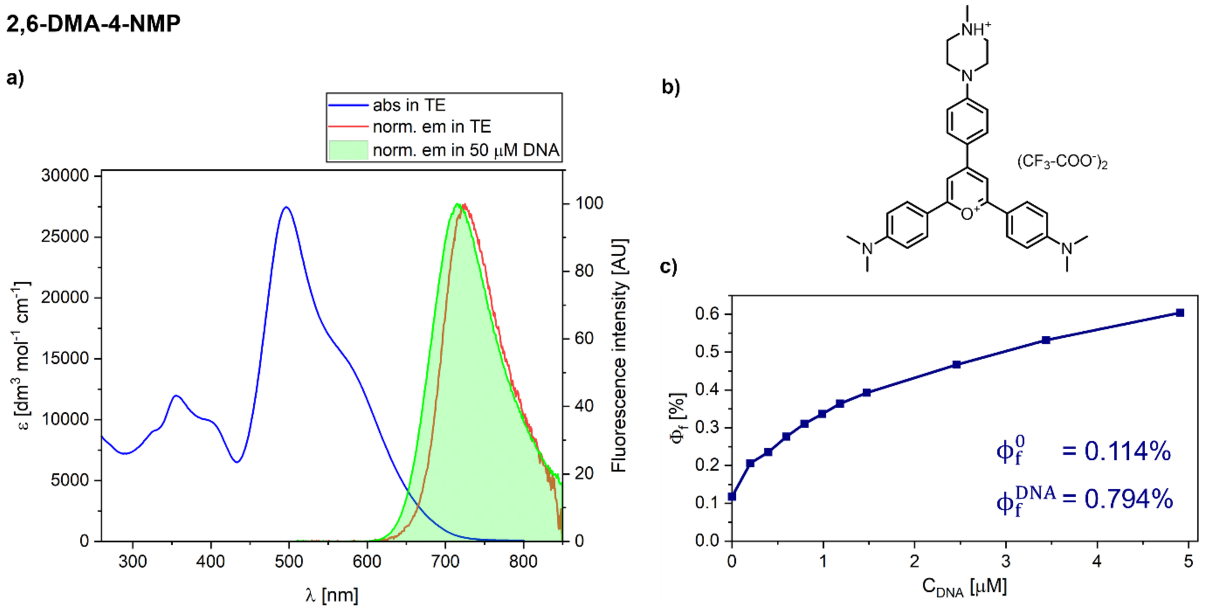


*Figure S146: **2,6-DMA-4-NMP** / 2,6-bis(4-(dimethylamino)phenyl)-4-(4-(4-methylpiperazin-1-yl)phenyl)pyrylium. a) Absorption in pH=7.4 TE buffer, normalised emission in TE buffer and in the presence of 50 µM plasmid DNA ($\lambda_{ex}$ = 496 nm). b) Structural formula. c) Fluorescence quantum yield in the presence of plasmid DNA in varying concentrations; $\phi_f^0$ and $\phi_f^{DNA}$ are the fluorescence quantum yield at 0 and 50 µM plasmid DNA, respectively.*

**2,4,6-NMP**

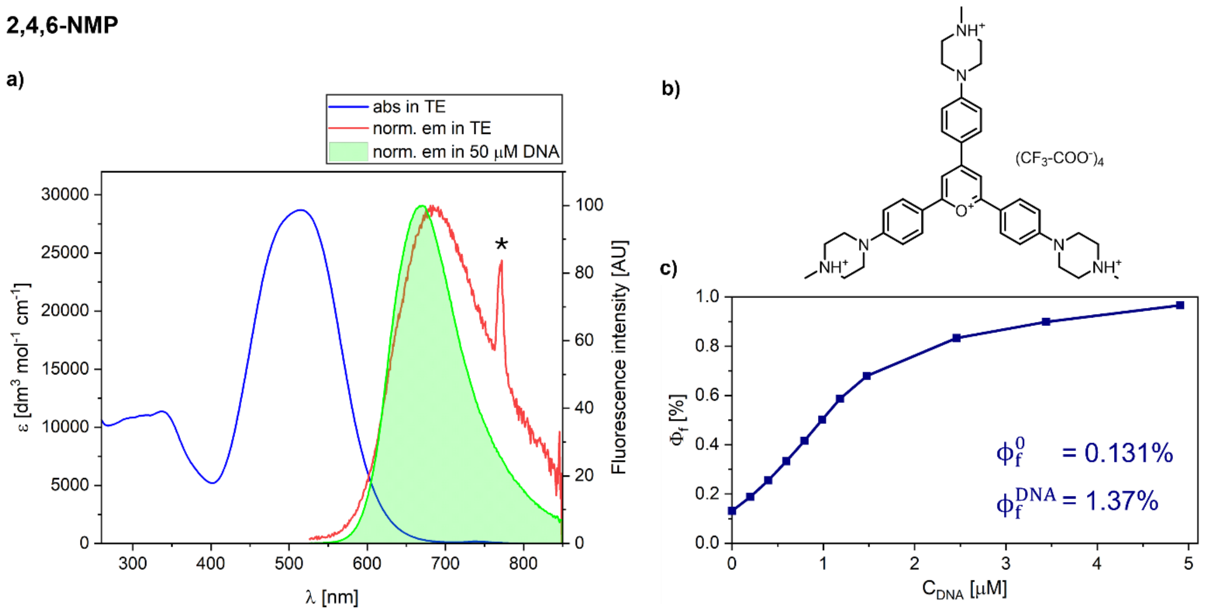


*Figure S147: **2,4,6-NMP** / 4,4',4''-(pyrylium-2,4,6-triyltris(benzene-4,1-diyl))tris(1-methylpiperazin-1-ium) tetrakis(trifluoroacetate). a) Absorption in pH=7.4 TE buffer, normalised emission in TE buffer and in the presence of 50 µM plasmid DNA ($\lambda_{ex}$ = 514 nm). The peak marked with an asterisk at $\lambda = 3\lambda_{ex}/2$ is an aritifact due to the scattering of stray light from the monochromator. b) Structural formula. c) Fluorescence quantum yield in the presence of plasmid DNA in varying concentrations; $\phi_f^0$ and $\phi_f^{DNA}$ are the fluorescence quantum yield at 0 and 50 µM plasmid DNA, respectively.*

**2,6-NMP-4-Morph**

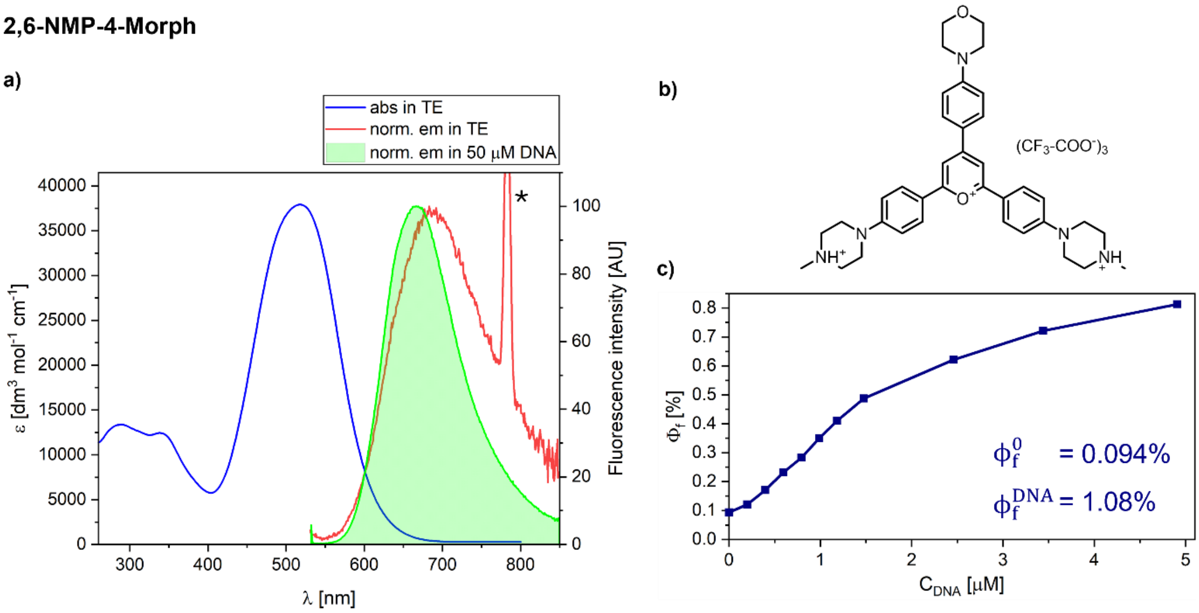


*Figure S148:* ***2,6-NMP-4-Morph*** */ 4,4'-((4-(4-morpholinophenyl)pyrylium-2,6-diyl)bis(4,1-phenylene))bis(1-methylpiperazin-1-ium) tris(trifluoroacetate). a) Absorption in pH=7.4 TE buffer, normalised emission in TE buffer and in the presence of 50 µM plasmid DNA ($\lambda_{ex}$ = 518 nm). The peak marked with an asterisk at $\lambda = 3\lambda_{ex}/2$ is an aritifact due to the scattering of stray light from the monochromator. b) Structural formula. c) Fluorescence quantum yield in the presence of plasmid DNA in varying concentrations; $\phi_f^0$ and $\phi_f^{DNA}$ are the fluorescence quantum yield at 0 and 50 µM plasmid DNA, respectively.*

**2,6-Ind-4-DMA**

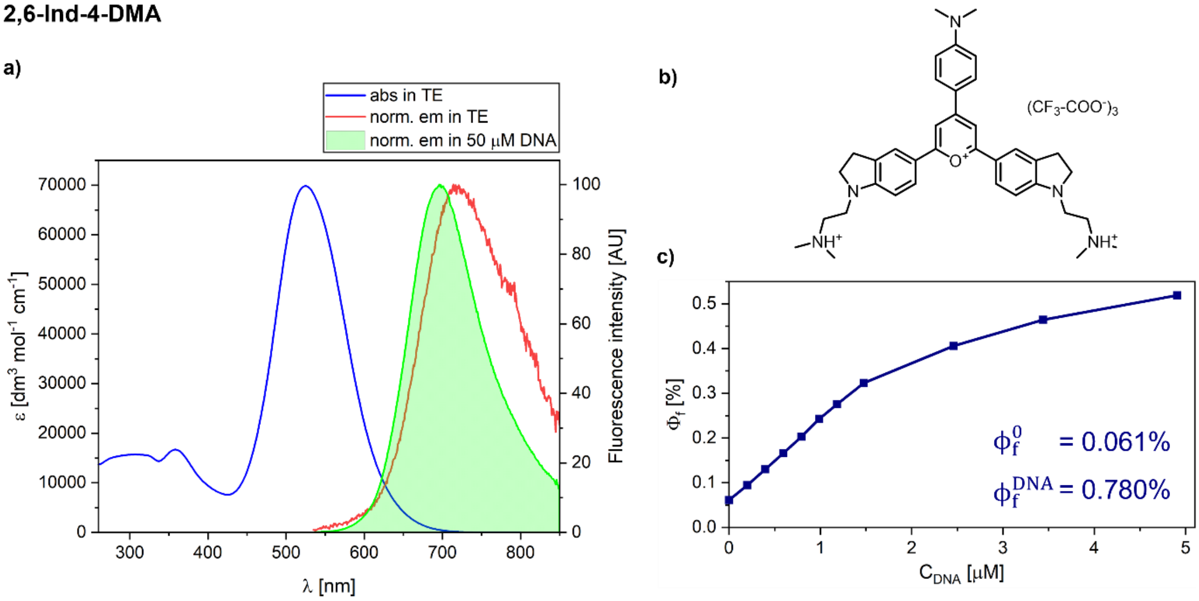


*Figure S149:* ***2,6-Ind-4-DMA*** */ 4-(4-(dimethylamino)phenyl)-2,6-bis(1-(2-(dimethylammonio)ethyl)indolin-5-yl)pyrylium tris(trifluoroacetate). a) Absorption in pH=7.4 TE buffer, normalised emission in TE buffer and in the presence of 50 µM plasmid DNA ($\lambda_{ex}$ = 524 nm). b) Structural formula. c) Fluorescence quantum yield in the presence of plasmid DNA in varying concentrations; $\phi_f^0$ and $\phi_f^{DNA}$ are the fluorescence quantum yield at 0 and 50 µM plasmid DNA, respectively.*

## 2,6-NdMP-4-DMA

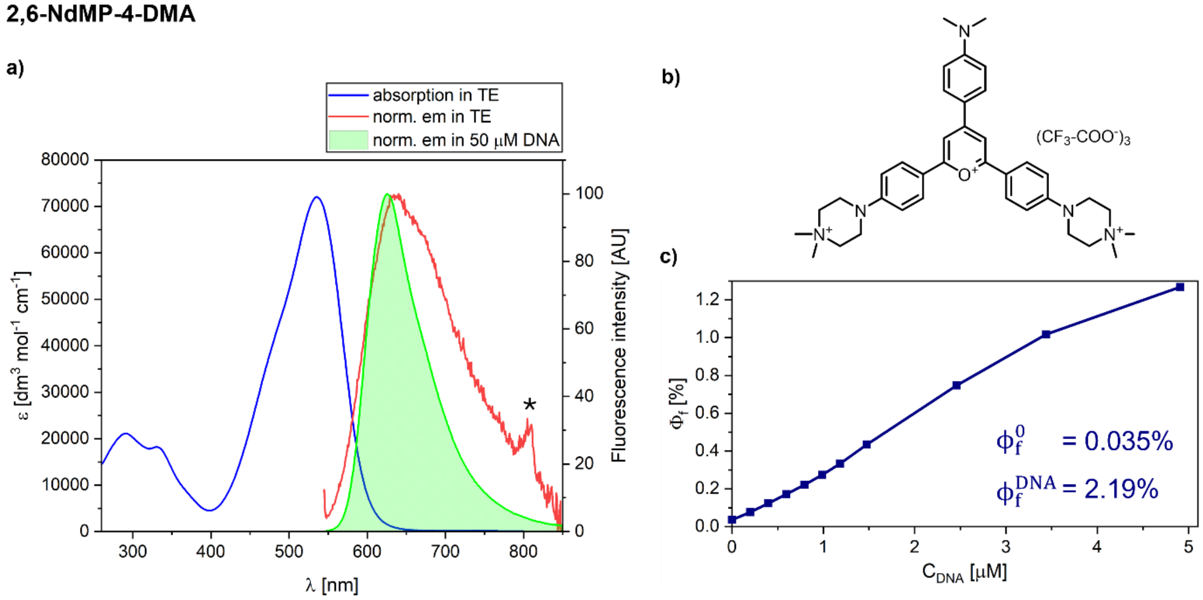


*Figure S150: **2,6-NdMP-4-DMA** / 4,4'-((4-(4-(dimethylamino)phenyl)pyrylium-2,6-diyl)bis(4,1-phenylene))bis(1,1-dimethylpiperazin-1-ium) tris(trifluoroacetate). a) Absorption in pH=7.4 TE buffer, normalised emission in TE buffer and in the presence of 50 µM plasmid DNA ($\lambda_{ex}$ = 535 nm). The peak marked with an asterisk at $\lambda = 3\lambda_{ex}/2$ is an aritifact due to the scattering of stray light from the monochromator. b) Structural formula. c) Fluorescence quantum yield in the presence of plasmid DNA in varying concentrations; $\phi_f^0$ and $\phi_f^{DNA}$ are the fluorescence quantum yield at 0 and 50 µM plasmid DNA, respectively.*

## 2,6-NMP-4-OMe

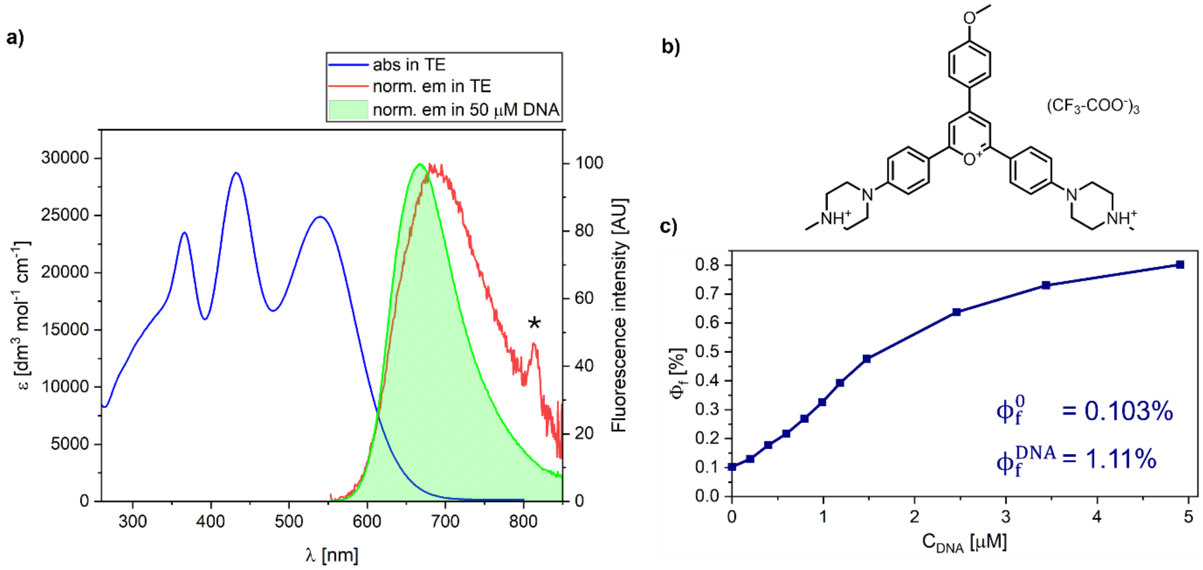


*Figure S151: **2,6-NMP-4-OMe** / 4,4'-((4-(4-methoxyphenyl)pyrylium-2,6-diyl)bis(4,1-phenylene))bis(1-methylpiperazin-1-ium) tris(trifluoroacetate). a) Absorption in pH=7.4 TE buffer, normalised emission in TE buffer and in the presence of 50 µM plasmid DNA ($\lambda_{ex}$ = 540 nm). The peak marked with an asterisk at $\lambda = 3\lambda_{ex}/2$ is an aritifact due to the scattering of stray light from the monochromator. b) Structural formula. c) Fluorescence quantum yield in the presence of plasmid DNA in varying concentrations; $\phi_f^0$ and $\phi_f^{DNA}$ are the fluorescence quantum yield at 0 and 50 µM plasmid DNA, respectively.*

**2,6-NMP-4-OEt**

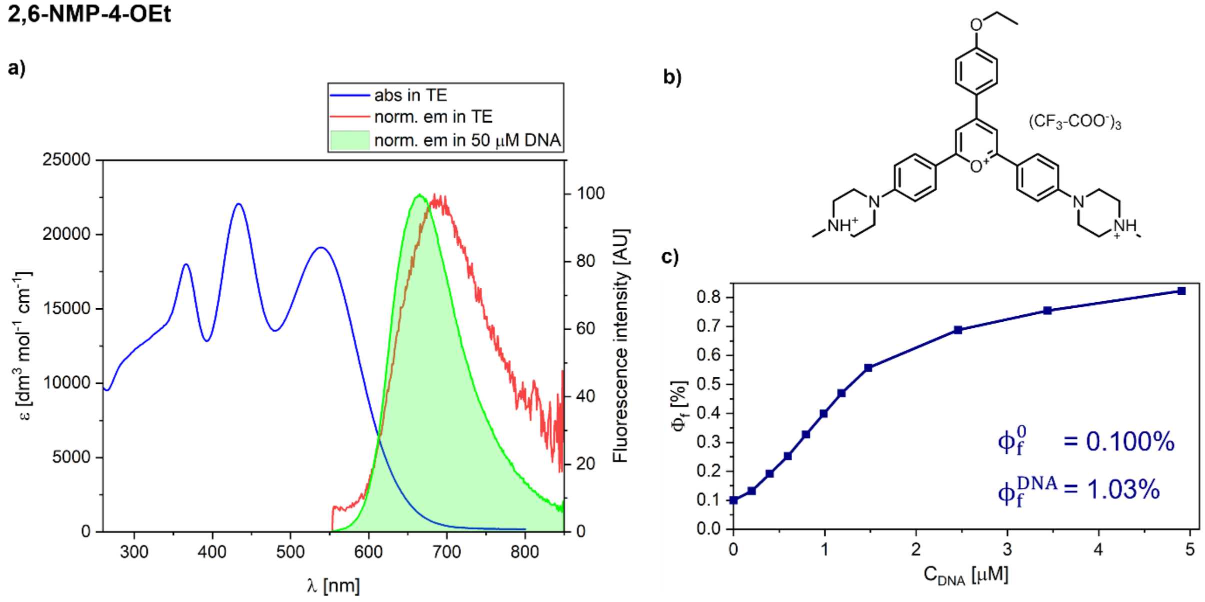


*Figure S152: **2,6-NMP-4-OEt** / 4,4'-((4-(4-ethoxyphenyl)pyrylium-2,6-diyl)bis(4,1-phenylene)). a) Absorption in pH=7.4 TE buffer, normalised emission in TE buffer and in the presence of 50 µM plasmid DNA ($\lambda_{ex}$ = 540 nm). b) Structural formula. c) Fluorescence quantum yield in the presence of plasmid DNA in varying concentrations; $\phi_f^0$ and $\phi_f^{DNA}$ are the fluorescence quantum yield at 0 and 50 µM plasmid DNA, respectively.*

**2,6-NMP-4-OH**

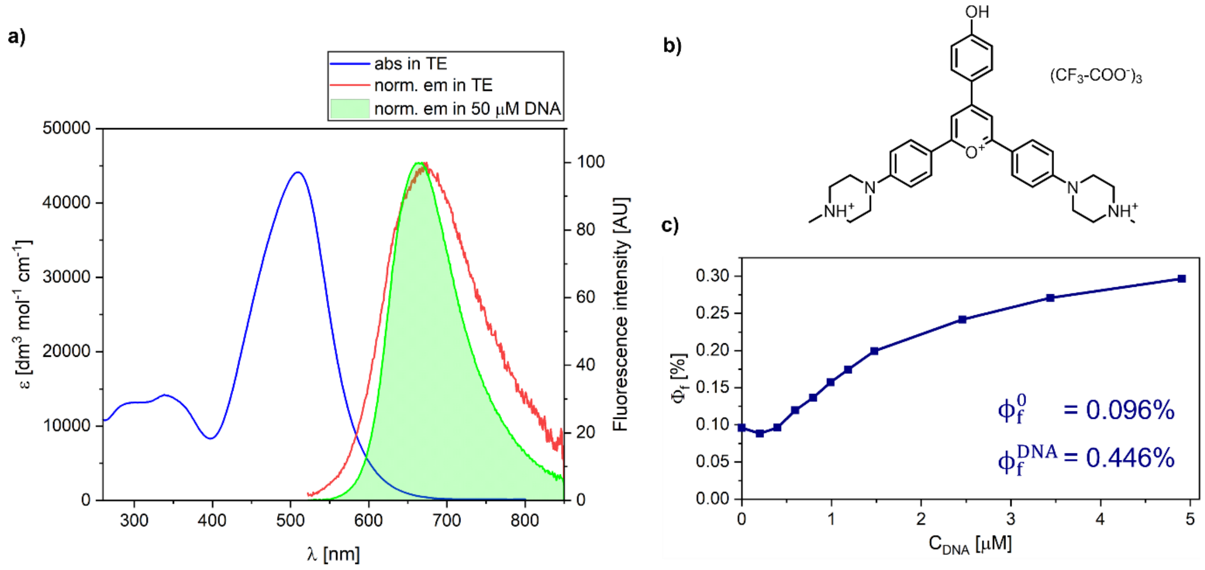


*Figure S153: **2,6-NMP-4-OH** / 4-(4-hydroxyphenyl)-2,6-bis(4-(4-methylpiperazin-1-yl)phenyl)pyrylium tris(trifluoroborate). a) Absorption in pH=7.4 TE buffer, normalised emission in TE buffer and in the presence of 50 µM plasmid DNA ($\lambda_{ex}$ = 510 nm). b) Structural formula. c) Fluorescence quantum yield in the presence of plasmid DNA in varying concentrations; $\phi_f^0$ and $\phi_f^{DNA}$ are the fluorescence quantum yield at 0 and 50 µM plasmid DNA, respectively.*

**2,6-NMP-4-2MeOPh**

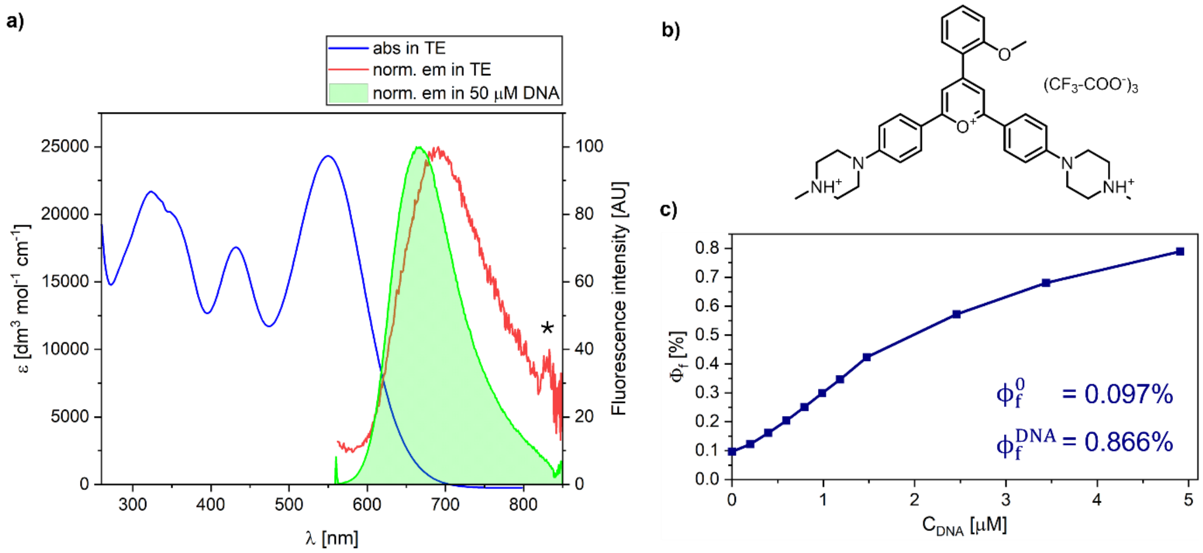


*Figure S154:* ***2,6-NMP-4-2MeOPh*** */ 4,4'-((4-(2-methoxyphenyl)pyrylium-2,6-diyl)bis(4,1-phenylene))bis(1-methylpiperazin-1-ium) tris(trifluoroacetate). a) Absorption in pH=7.4 TE buffer, normalised emission in TE buffer and in the presence of 50 μM plasmid DNA ($\lambda_{ex}$ = 550 nm). b) Structural formula. c) Fluorescence quantum yield in the presence of plasmid DNA in varying concentrations; $\phi_f^0$ and $\phi_f^{DNA}$ are the fluorescence quantum yield at 0 and 50 μM plasmid DNA, respectively.*

**2,6-Pip-4-2MeOPh**

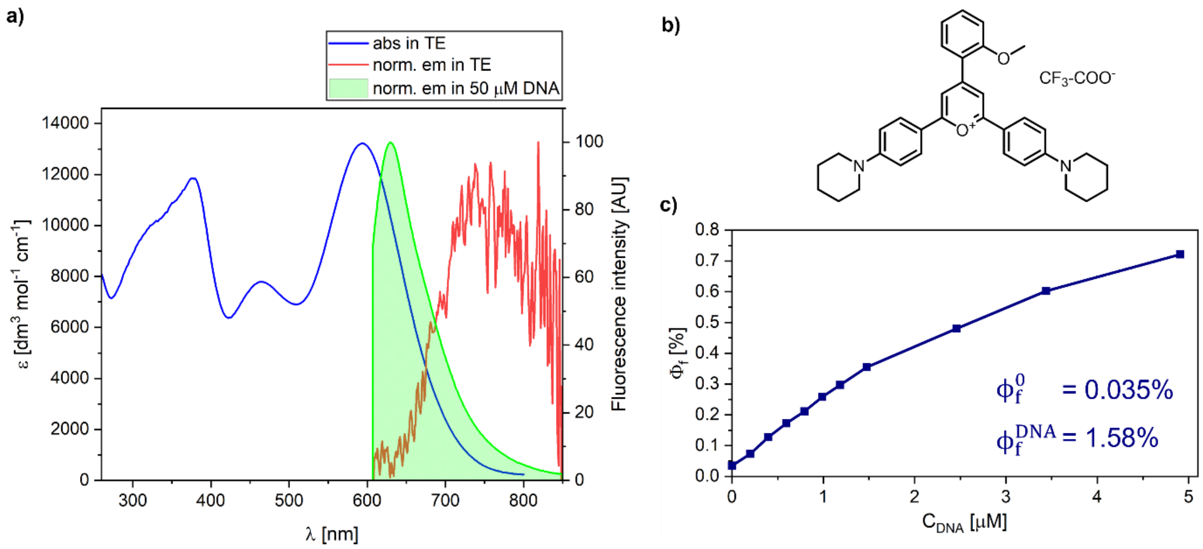


*Figure S155:* ***2,6-Pip-4-2MeOPh*** */ 4-(2-methoxyphenyl)-2,6-bis(4-(piperidin-1-yl)phenyl)pyrylium trifluoroacetate. a) Absorption in pH=7.4 TE buffer, normalised emission in TE buffer and in the presence of 50 μM plasmid DNA ($\lambda_{ex}$ = 594 nm). b) Structural formula. c) Fluorescence quantum yield in the presence of plasmid DNA in varying concentrations; $\phi_f^0$ and $\phi_f^{DNA}$ are the fluorescence quantum yield at 0 and 50 μM plasmid DNA, respectively.*

**2,4-NMP-6-Ph**

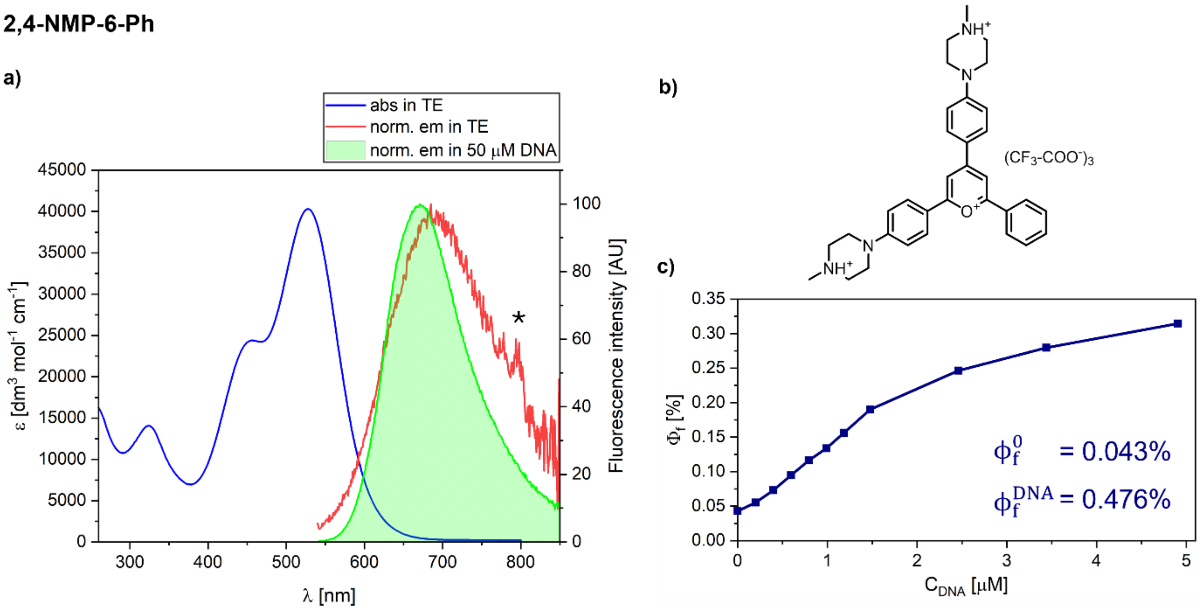


*Figure S156:* ***2,4-NMP-6-Ph*** */ 4,4'-((6-phenylpyrylium-2,4-diyl)bis(4,1-phenylene))bis(1-methylpiperazin-1-ium). a) Absorption in pH=7.4 TE buffer, normalised emission in TE buffer and in the presence of 50 µM plasmid DNA ($\lambda_{ex}$ = 528 nm). The peak marked with an asterisk at $\lambda = 3\lambda_{ex}/2$ is an aritifact due to the scattering of stray light from the monochromator. b) Structural formula. c) Fluorescence quantum yield in the presence of plasmid DNA in varying concentrations; $\phi_f^0$ and $\phi_f^{DNA}$ are the fluorescence quantum yield at 0 and 50 µM plasmid DNA, respectively.*

**2,4-DMA-6-Me**

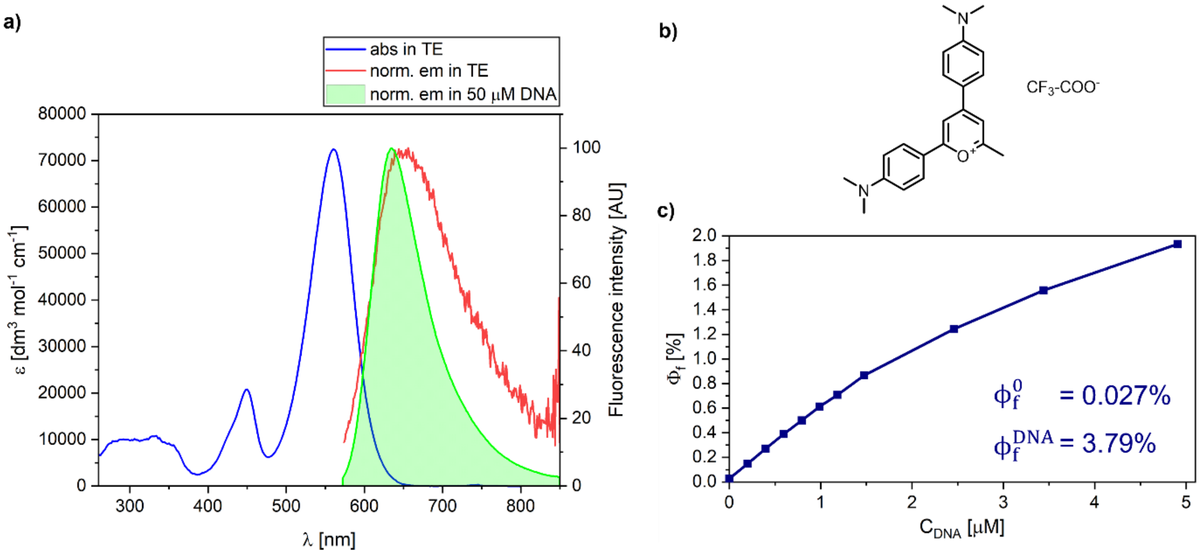


*Figure S157:* ***2,4-DMA-6-Me*** */ 2,4-bis(4-(dimethylamino)phenyl)-6-methylpyrylium trifluoroacetate. a) Absorption in pH=7.4 TE buffer, normalised emission in TE buffer and in the presence of 50 µM plasmid DNA ($\lambda_{ex}$ = 562 nm). b) Structural formula. c) Fluorescence quantum yield in the presence of plasmid DNA in varying concentrations; $\phi_f^0$ and $\phi_f^{DNA}$ are the fluorescence quantum yield at 0 and 50 µM plasmid DNA, respectively.*

**2,4-NMP-6-Me**

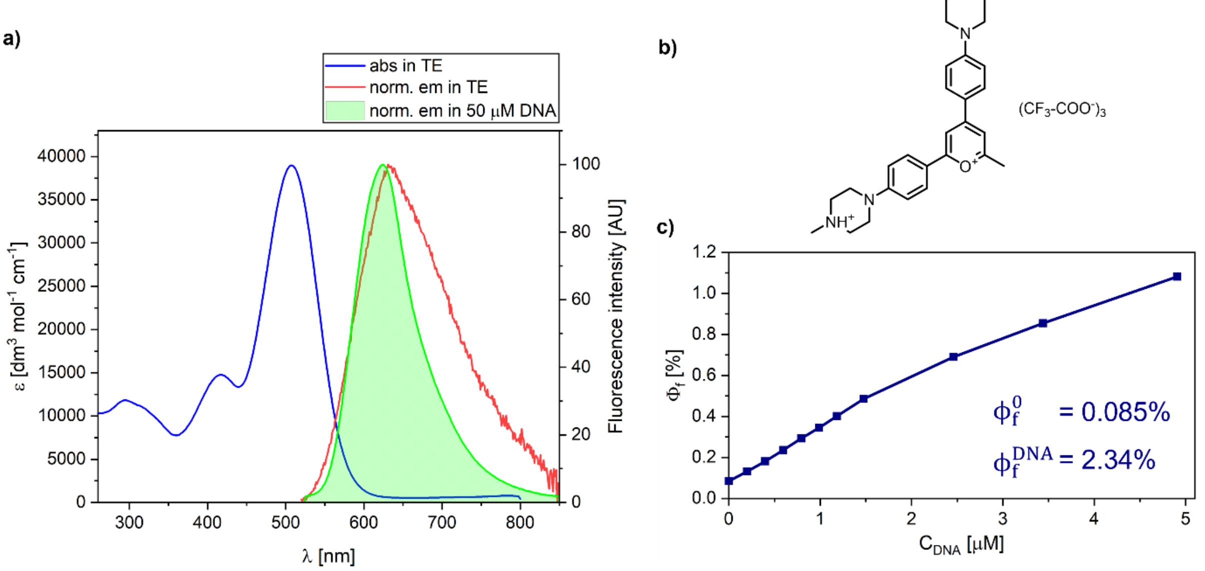


*Figure S158:* ***2,4-NMP-6-Me*** */ 4,4'-((6-methylpyrylium-2,4-diyl)bis(4,1-phenylene))bis(1-methylpiperazin-1-ium) tris(trifluoroacetate). a) Absorption in pH=7.4 TE buffer, normalised emission in TE buffer and in the presence of 50 µM plasmid DNA ($\lambda_{ex}$ = 508 nm). b) Structural formula. c) Fluorescence quantum yield in the presence of plasmid DNA in varying concentrations; $\phi_f^0$ and $\phi_f^{DNA}$ are the fluorescence quantum yield at 0 and 50 µM plasmid DNA, respectively.*

**2,4-DMA-6-BTA**

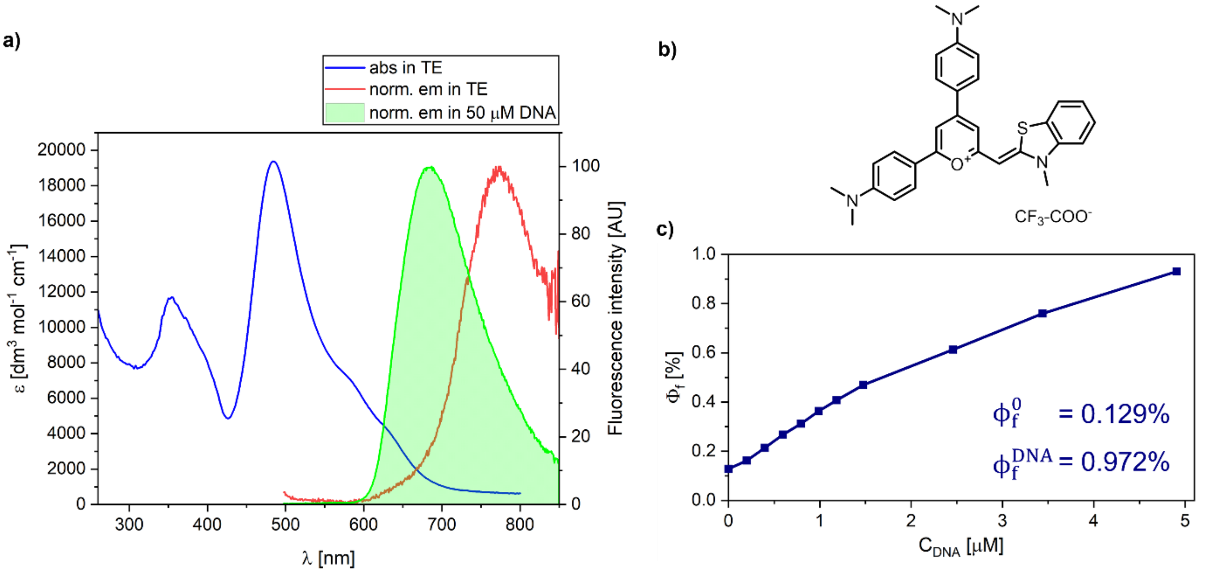


*Figure S159:* ***2,4-DMA-6-BTA*** */ (Z)-2,4-bis(4-(dimethylamino)phenyl)-6-((3-methylbenzo[d]thiazol-2(3H)-ylidene)methyl)pyrylium trifluoroacetate. a) Absorption in pH=7.4 TE buffer, normalised emission in TE buffer and in the presence of 50 µM plasmid DNA ($\lambda_{ex}$ = 484 nm). b) Structural formula. c) Fluorescence quantum yield in the presence of plasmid DNA in varying concentrations; $\phi_f^0$ and $\phi_f^{DNA}$ are the fluorescence quantum yield at 0 and 50 µM plasmid DNA, respectively.*

## 4.6. Emission measurements in nitrogen atmosphere

The emission spectrum of **2,6-NdMP-4-DMA** was recorded from a 1 µM solution in pH=7.4 TE buffer and in a 5 µM plasmid DNA solution in the same buffer. After the measurement the solution was flushed with nitrogen for 10 minutes and the spectra were re-recorded. Fluorescence quantum yields ($\Phi_f$) were determined as described above.

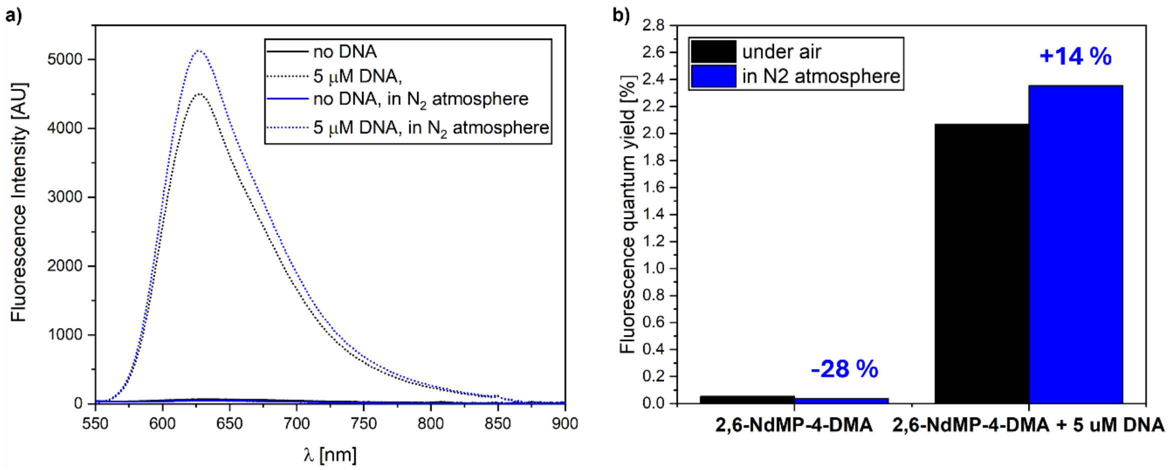


*Figure S160: a) Emission spectra of* ***2,6-NdMP-4-DMA*** *in pH=7.4 TE buffer and in the presence of 5 µM plasmid DNA measured under air and in nitrogen atmosphere. b) Effecive fluorescence quantum yields determined from these measurements and the change in $\Phi_f$ caused by the exclusion of oxygen.*

## 4.7. DNA titration experiments

Fluorescence enhancement of the dyes was measured using pAAV-hSyn-DIO-ChrimsonR-mRuby3-ST plasmid dsDNA containing 6882 base pairs with a 53.8% GC content. The plasmid DNA was purified using the EndoFree Plasmid Maxi Kit (Qiagen), following the manufacturer's instructions. Concentration of the purified plasmid was measured using NanoDrop™ 2000/2000c Spectrophotometers (Thermo Scientific™). Exact sequence of the plasmid was the following:

aagggcgatcggtgcgggcctcttcgctattacgccagctgcgcgctcgctcgctcactgaggccgcccgggcaaagcccgggcgtcgggcgacctttggtcgcccggcctcagtgagcgagcgagcg
cgcagagagggagtggccaactccatcactaggggttccttgtagttaatgattaacccgccatgctacttatctacgtagccatgctctaggaagatctctgcagagggccctgcgtatgagtgcaagtgggtt
ttaggaccaggatgaggcggggtgggggtgcctacctgacgaccgaccccgacccactggacaagcacccaacccccattccccaaattgcgcatcccctatcagagagggggaggggaaacaggatg
cggcgaggcgcgtgcgcactgccagcttcagcaccgcggacagtgccttcgcccccgcctggcggcgcgcgccaccgccgcctcagcactgaaggcgcgctgacgtcactcgccggtcccccgcaaa
ctccccttcccggccaccttggtcgcgtccgcgccgccgccggcccagccggaccgcaccacgcgaggcgcgagatagggggcacgggcgcgaccatctgcgctgcggcgccggcgactcagcgc
tgcctcagtctgcggtgggcagcggaggagtcgtgtcgtgcctgagagcgcagtcgaattcaagctgatagcaaggatcctctagagtcgactccggaataacttcgtataggatactttatacgaagttatgc
agaatggtagctggattgtagctgctattagcaatatgaaacctcttaataacttcgtatagcatacattatacgaagttatggcgcgccttagaatctggtggcttcagggaagtccgtggcacagctgatgaagc
tgtcaatgctggacatgctccgcatgtcgatgacgccctccgtgcgtgcgggcagaggggccacggggctgggcatgctgctcatctccagctcctctggaggcttgctctgcggggccatctccttggtgtt
gaggatgggctgggactgctctagggccttgtacagctcgtccatcccaccaccaaggttgctgtatttggcaactgccacttctctttgcactacgtaggtttcattgtcactctcctcgatcctttccaggcggtg
atcaacggcatggacaccgggcatcttgatgttcccgacggtctttttgacctgtaagttgtcacgaagttgcagtgcagatggccaccaccatcaactttcagtgcgatgtcagtgtatcctctcagaccaccat
ctgctggatacatcatctctgtattaggctcccaacccttggtcttcttctgcatcacgggaccattggagggaaagtttacccctctgaccttgacgttgtagacgagctcgccatcctcaaggctggtgtcctgc
gtgacggtgacgactccaccatcttcgtatctcgtaactctttcccaagtaaaaccctcaggaaaggactgtttaaagaaatcagggatgtcggccgggtacttgataaaggtacggctgccatacatgaacga
cgtggcaagaatgtcaaaggcaaatggcaggggtcctccctcgatgactttgatcctcatggtttgcactccctcgtacggtctgccttctccttcacctgtgcatttgaattggtggccgttgaccgaaccttccat
gaccaccttcatacgcatattttccttgatcagctcttcgcccttagacaccattaccggtgccgccactgtgtcctcgtcctcctcctccacgaactcttccacttccacctcctcgcctccgatctccatcttggtgg
tcttccggatgtcgccgtggatcaggatgtgctcgtggatcttgatcctcaggtggtgggccaggaaggtccaaaactccttggcgatgatgtcgcagatgctgtggccgatgctgttggcgtaagggctcagc
ttcagcagtccttctggtcccactgcccagaggattgggtagctgccccaagaggcgaagtaagcgtaggccatcagcttcacgaccatgcggcaatggcctttaggcacgctgtggttggcttccacgtagc
acttggcggcctggaagtacatgtagccgccgtagatgcaagacacgatatacagcagccacttgagccaatcggtagccagtcctgcggccatgccgaacacgatcattcccacgcaagacacgatcag
gcccatggtccgcttgctgtagtcgttcttcaggccgctcaggttgctcagtctgatcaggatcacggggcaagacagcagccactcgaagtagcgcaggcaataggcgtggttgccggtagacaggtacac
tgtggcggggctgctgaactccttgaagatctccagggtcacgaacagcacctcgacgcaacagacgtagacctcctcccaaccgcaagtggccttccaggcgctgaagccgtagaatgtcagcagggcg
atggcgatgctgaaagcaatccactggcagacctgggcgccgatcttttctcctggtgtgcctctgctgtgcagccaagcctgcttcttctccactcccacgaccaggtagccgcctgtggcctccacgaagca
atatccgtatccggcgtcgctcagtccgtaagaagggtcacaccaccactcggtgctgaagcactcgcctgtaggtgtcattcctccacagcccatgtcctcgtggtggtaggggttaggccaagggttgatgc
ctccggcggcaaacagagatctggtggcgctgctgatcagctcagccatggtgctagcataacttcgtataaagtatcctatacgaagttatttgccttaacccagaaattatcactgttattctttagaatggtgca
aagaataacttcgtataatgtatgctatacgaagttatgaattcgatatcaagcttatcgataatcaacctctggattacaaaatttgtgaaagattgactggtattcttaactatgttgctccttttacgctatgtggatac
gctgctttaatgcctttgtatcatgctattgcttcccgtatggctttcattttctcctccttgtataaatcctggttgctgtctctttatgaggagttgtggcccgttgtcaggcaacgtggcgtggtgtgcactgtgtttgct
gacgcaacccccactggttggggcattgccaccacctgtcagctcctttccgggactttcgctttccccctccctattgccacggcggaactcatcgccgcctgccttgcccgctgctggacaggggctcggct
gttgggcactgacaattccgtggtgttgtcggggaaatcatcgtcctttccttggctgctcgcctgtgttgccacctggattctgcgcgggacgtccttctgctacgtcccttcggccctcaatccagcggaccttc
cttcccgcggcctgctgccggctctgcggcctcttccgcgtcttcgccttcgccctcagacgagtcggatctccctttgggccgcctccccgcatcgataccgtcgacctcgacccgggcggccgcttcgagc
agacatgataagatacattgatgagtttggacaaaccacaactagaatgcagtgaaaaaaatgctttatttgtgaaatttgtgatgctattgctttatttgtaaccattataagctgcaataaacaagttaacaacaaca
attgcattcattttatgtttcaggttcagggggagatgtgggaggttttttaaagcaagtaaaacctctacaaatgtggtaaaatcgataaggatcttcctagagcatggctacgtagataagtagcatggcgggtta
atcattaactacaaggaacccctagtgatggagttggccactccctctctgcgcgctcgctcgctcactgaggccgggcgaccaaaggtcgcccgacgcccgggctttgcccgggcggcctcagtgagcga
gcgagcgcgcagctggcgtaatagcgaagaggcccgcaccgatcgcccttcccaacagttgcgcagcctgaatggcgaatgggacgcgccctgtagcggcgcattaagcgcggcgggtgtggtggtta
cgcgcagcgtgaccgctacacytgccagcgccctagcgcccgctcctttcgctttcttcccttcctttctcgccacgttcgccggctttccccgtcaagctctaaatcgggggctccctttagggttccgatttagt
gctttacggcacctcgaccccaaaaaacttgattagggtgatggctgtccattaattgcgttgcgctcactgcccgctttccagtcgggaaacctgtcgtgccagctgcattaatgaatcggccaacgcgcggg
gagaggcggtttgcgtattgggcgctcttccgcttcctcgctcactgactcgctgcgctcggtcgttcggctgcggcgagcggtatcagctcactcaaaggcggtaatacggttatccacagaatcaggggata
acgcaggaaagaacatgtgagcaaaaggccagcaaaaggccaggaaccgtaaaaaggccgcgttgctggcgtttttccataggctccgcccccctgacgagcatcacaaaaatcgacgctcaagtcaga
ggtggcgaaacccgacaggactataaagataccaggcgtttccccctggaagctccctcgtgcgctctcctgttccgaccctgccgcttaccggatacctgtccgcctttctcccttcgggaagcgtggcgcttt
ctcatagctcacgctgtaggtatctcagttcggtgtaggtcgttcgctccaagctgggctgtgtgcacgaaccccccgttcagcccgaccgctgcgccttatccggtaactatcgtcttgagtccaacccggtaa
gacacgacttatcgccactggcagcagccactggtaacaggattagcagagcgaggtatgtaggcggtgctacagagttcttgaagtggtggcctaactacggctacactagaagaacagtatttggtatctg
cgctctgctgaagccagttaccttcggaaaaagagttggtagctcttgatccggcaaacaaaccaccgctggtagcggtggtttttttgtttgcaagcagcagattacgcgcagaaaaaaaggatctcaagaag
atcctttgatcttttctacggggtctgacgctcagtggaacgaaaactcacgttaagggattttggtcatgagattatcaaaaaggatcttcacctagatccttttaaattaaaaatgaagttttaaatcaatctaaagta

tatatgagtaaacttggtctgacagttaccaatgcttaatcagtgaggcacctatctcagcgatctgtctatttcgttcatccatagttgcctgactccccgtcgtgtagataactacgatacgggagggcttaccatc
tggccccagtgctgcaatgataccgcgagacccacgctcaccggctccagatttatcagcaataaaccagccagccggaagggccgagcgcagaagtggtcctgcaactttatccgcctccatccagtctat
taattgttgccgggaagctagagtaagtagttcgccagttaatagtttgcgcaacgttgttgccattgctacaggcatcgtggtgtcacgctcgtcgtttggtatggcttcattcagctccggttcccaacgatcaag
gcgagttacatgatcccccatgttgtgcaaaaaagcggttagctccttcggtcctccgatcgttgtcagaagtaagttggccgcagtgttatcactcatggttatggcagcactgcataattctcttactgtcatgcc
atccgtaagatgcttttctgtgactggtgagtactcaaccaagtcattctgagaatagtgtatgcggcgaccgagttgctcttgcccggcgtcaatacgggataataccgcgccacatagcagaactttaaaagtg
ctcatcattggaaaacgttcttcggggcgaaaactctcaaggatcttaccgctgttgagatccagttcgatgtaacccactcgtgcacccaactgatcttcagcatcttttactttcaccagcgtttctgggtgagca
aaaacaggaaggcaaaatgccgcaaaaaagggaataagggcgacacggaaatgttgaatactcatactcttcctttttcaatattattgaagcatttatcagggttattgtctcatgagcggatacatatttgaatgt
atttagaaaaataaacaaataggggttccgcgcacatttccccgaaaagtgccacctaaattgtaagcgttaatattttgttaaaattcgcgttaaatttttgttaaatcagctcattttttaaccaataggccgaaatcg
gcaaaatcccttataaatcaaaagaatagaccgagatagggttgagtgttgttccagtttggaacaagagtccactattaaagaacgtggactccaacgtcaaagggcgaaaaaccgtctatcagggcgatgg
cccactacgtgaaccatcaccctaatcaagttttttggggtcgaggtgccgtaaagcactaaatcggaaccctaaagggagcccccgatttagagcttgacggggaaagccggcgaacgtggcgagaaagg
aagggaagaaagcgaaaggagcgggcgctagggcgctggcaagtgtagcggtcacgctgcgcgtaaccaccacacccgccgcgcttaatgcgccgctacagggcgcgtcccattcgccattcaggctg
cgcaactgttggg

To perform the DNA titration experiments the following stack solutions were prepared:

- 1 mM solution of the dyes in DMSO
- 1 mM and 0.1 mM solution of dsDNA (these values refer to the concentration of base pairs) in TE buffer

To avoid photobleaching, the dye stock solutions were kept in darkness before and after use.

Each measurement started with recording an emission spectrum from 2.0 mL blank TE buffer. To this buffer was added:

- 2 μL 1 mM solution of the given dye
- 6 × 4 μL + 1 × 6 μL 0.1 mM dsDNA solution
- 2 + 2 + 3 μL 1 mM dsDNA solution

After each addition the solution was homogenized using and an emission spectrum was recorded using the absorption maximum of the given free dye as excitation wavelength (1 background + 10 data points).

For each spectrum an effective quantum yield was calculated using the relative quantum yield method (Anal. Chem. 2011, 83, 1232–1242). The word *effective* stands for the fact that the measured fluorescence quantum yield is the weighted average of the $\Phi_f$ of the free and the DNA-bound molecules ($\Phi_f^0$ and $\Phi_f^{DNA}$). During this calculation, the extinction coefficient of the DNA-bound dye was approximated with the extinction coefficient of the free dye. The first six points of the titration curve were used to calculate the AFE and RFE.

**Table S4:** Effective fluorescence quantum yields of the dyes in the presence of varying concentrations of dsDNA; absolute and relative fluorescence enhancements (AFE, RFE) calculated from the first five points of the measurement.

| C(DNA) [uM] | 2,4,6-DMA | 2,6-NMP-4-DMA | 2,6-DMA-4-NMP | 2,4,6-NMP |
|---|---|---|---|---|
| **0.000** | 0.061 | 0.074 | 0.117 | 0.131 |
| **0.200** | 0.057 | 0.110 | 0.206 | 0.188 |
| **0.398** | 0.064 | 0.155 | 0.235 | 0.256 |
| **0.596** | 0.076 | 0.210 | 0.277 | 0.333 |
| **0.793** | 0.090 | 0.274 | 0.311 | 0.416 |
| **0.990** | 0.101 | 0.346 | 0.336 | 0.503 |
| **1.185** | 0.114 | 0.438 | 0.364 | 0.587 |
| **1.477** | 0.129 | 0.595 | 0.393 | 0.680 |
| **2.459** | 0.165 | 1.074 | 0.466 | 0.833 |
| **3.440** | 0.198 | 1.460 | 0.532 | 0.899 |
| **4.907** | 0.242 | 1.871 | 0.604 | 0.966 |
| **50.00** | 0.290 | 3.561 | 0.794 | 1.367 |
| **AFE:** | 31.5 | 162 | 63.4 | 103 |
| **RFE:** | 0.62 | 3.42 | 1.97 | 2.74 |

**Table S5:** Effective fluorescence quantum yields of the dyes in the presence of varying concentrations of dsDNA; absolute and relative fluorescence enhancements (AFE, RFE) calculated from the first five points of the measurement.

| C(DNA) [uM] | 2,6-NMP-4-Morph | 2,6-Ind-4-DMA | 2,6-NdMP-4-DMA | 2,6-NMP-4-OMe |
|---|---|---|---|---|
| **0.000** | 0.094 | 0.061 | 0.035 | 0.103 |
| **0.200** | 0.121 | 0.094 | 0.076 | 0.129 |
| **0.398** | 0.171 | 0.130 | 0.124 | 0.177 |
| **0.596** | 0.232 | 0.166 | 0.171 | 0.217 |
| **0.793** | 0.283 | 0.203 | 0.221 | 0.269 |
| **0.990** | 0.350 | 0.243 | 0.274 | 0.326 |
| **1.185** | 0.411 | 0.276 | 0.334 | 0.393 |
| **1.477** | 0.488 | 0.323 | 0.435 | 0.476 |
| **2.459** | 0.622 | 0.406 | 0.747 | 0.637 |
| **3.440** | 0.721 | 0.464 | 1.018 | 0.730 |
| **4.907** | 0.813 | 0.519 | 1.268 | 0.802 |
| **50.00** | 1.081 | 0.781 | 2.192 | 1.108 |
| **AFE:** | 93.6 | 125 | 169 | 52.8 |
| **RFE:** | 2.64 | 2.94 | 6.66 | 2.07 |

**Table S6.** Effective fluorescence quantum yields of the dyes in the presence of varying concentrations of dsDNA; absolute and relative fluorescence enhancements (AFE, RFE) calculated from the first five points of the measurement.

| C(DNA) [uM] | 2,6-NMP-4-OEt | 2,6-NMP-4-OH | 2,6-NMP-4-2MeOPh | 2,6-Pip-4-2MeOPh |
|---|---|---|---|---|
| **0.000** | 0.100 | 0.096 | 0.097 | 0.035 |
| **0.200** | 0.132 | 0.088 | 0.122 | 0.073 |
| **0.398** | 0.192 | 0.096 | 0.161 | 0.128 |
| **0.596** | 0.253 | 0.120 | 0.204 | 0.173 |
| **0.793** | 0.327 | 0.137 | 0.251 | 0.211 |
| **0.990** | 0.399 | 0.157 | 0.300 | 0.258 |
| **1.185** | 0.470 | 0.174 | 0.347 | 0.297 |
| **1.477** | 0.558 | 0.199 | 0.424 | 0.355 |
| **2.459** | 0.688 | 0.242 | 0.572 | 0.480 |
| **3.440** | 0.755 | 0.271 | 0.681 | 0.602 |
| **4.907** | 0.823 | 0.297 | 0.789 | 0.721 |
| **50.00** | 1.028 | 0.446 | 0.866 | 1.579 |
| **AFE:** | 55.5 | 25.1 | 47.8 | 30.1 |
| **RFE:** | 2.91 | 0.59 | 2.03 | 6.44 |

**Table S7:** Effective fluorescence quantum yields of the dyes in the presence of varying concentrations of dsDNA; absolute and relative fluorescence enhancements (AFE, RFE) calculated from the first five points of the measurement.

| C(DNA) [uM] | 2,4-NMP-6-Ph | 2,4-DMA-6-Me | 2,4-NMP-6-Me | 2,4-DMA-6-BTA |
|---|---|---|---|---|
| **0.000** | 0.043 | 0.027 | 0.085 | 0.129 |
| **0.200** | 0.055 | 0.148 | 0.131 | 0.162 |
| **0.398** | 0.073 | 0.271 | 0.181 | 0.214 |
| **0.596** | 0.095 | 0.390 | 0.236 | 0.268 |
| **0.793** | 0.116 | 0.501 | 0.293 | 0.312 |
| **0.990** | 0.134 | 0.613 | 0.345 | 0.364 |
| **1.185** | 0.156 | 0.708 | 0.402 | 0.407 |
| **1.477** | 0.190 | 0.867 | 0.485 | 0.469 |
| **2.459** | 0.246 | 1.243 | 0.690 | 0.613 |
| **3.440** | 0.280 | 1.558 | 0.855 | 0.759 |
| **4.907** | 0.314 | 1.934 | 1.081 | 0.931 |
| **50.00** | 0.476 | 3.792 | 2.339 | 0.972 |
| **AFE:** | 37.9 | 434 | 103 | 46.2 |
| **RFE:** | 2.19 | 22.3 | 3.11 | 1.85 |

***Table S8:*** *Spectroscopical properties of selected pyrylium dyes compared to those of thiazole-orange-based DNA dyes*[9]*. Extinction coefficients ($\varepsilon^{TE}$, dm$^3$ mol$^{-1}$ cm$^{-1}$) absorption maxima ($\lambda_{abs}$, nm) emission maximum wavelengths in solution and in dsDNA ($\lambda_{em}^{TE}$, $\lambda_{em}^{DNA}$, nm); fluorescence quantum yields in solution and in dsDNA ($\Phi_f^0$, $\Phi_f^{DNA}$, percent) in pH=7.4 TE buffer. Absolute fluorescence enhancements (**AFE**, dm$^6$ cm$^{-1}$ mmol$^{-2}$) and relative fluorescence enhancements (**RFE**, dm$^3$ µmol$^{-1}$).*

| Compounds | ε | $\lambda_{abs}$ | $\lambda_{em}^{TE}/\lambda_{em}^{DNA}$ | $\Phi_f^0$ [%] | $\Phi_f^{DNA}$ [%] | AFE | RFE |
|---|---|---|---|---|---|---|---|
| **2,4,6-DMA** | 82 800 | 534 | 733/699 | 0.061 | 0.29 | 31.5 | 0.62 |
| **2,6-NMP-4-DMA** | 64 500 | 530 | 702/629 | 0.074 | 3.56 | 162 | 3.41 |
| **2,6-NdMP-4-DMA** | 72 000 | 535 | 640/628 | 0.035 | 2.19 | 169 | 6.66 |
| **2,4-DMA-6-Me** | 72 300 | 562 | 656/637 | 0.027 | 3.79 | 434 | 22.3 |
| **SYBR Safe** | 54 500 | 531 | 530/531 | 0.017 | 27.8 | 1706 | 186 |
| **SYBR Green** | 54 200 | 525 | 528/524 | 0.025 | 62.9 | 11409 | 858 |
| **SYTO-9** | 55 100 | 503 | 505/503 | 0.083 | 57 | 5472 | 120 |

**Dependence of fluorescence enhancement on pH, temperature, and ionic strength**

DNA titration experiments were conducted in the following media:

- pH=7.4 TE buffer (Tris-EDTA, 10 mM Tris/Tris-HCl, 1 mM EDTA)
- pH=6.9 TE buffer (Tris-EDTA, 10 mM Tris/Tris-HCl, 1 mM EDTA)
- pH=9.0 TE buffer (Tris-EDTA, 10 mM Tris/Tris-HCl, 1 mM EDTA)
- pH=7.4 TE buffer (Tris-EDTA, 10 mM Tris/Tris-HCl, 1 mM EDTA) + 100 mM NaCl (ionic strength: 0,108 M)

Furthermore, the DNA titration curves were also recorded at 45 and 60 °C. In this case each solutions were prepared separately, thermostated to the appropriate temperature in a water bath, and measured as quickly as possible.

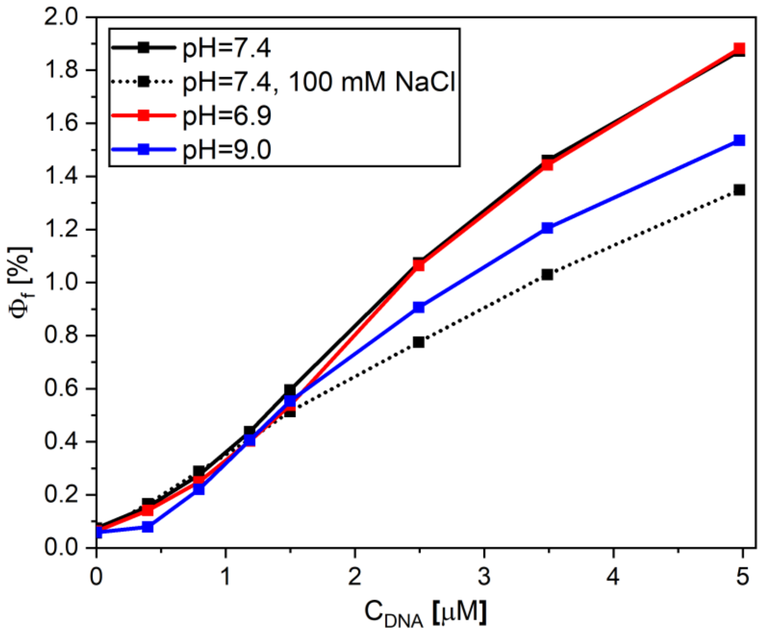


*Figure S161: Fluorescence quantum yield in the presence of plasmid DNA in varying concentrations, measured at different pH-s and ionic strengths.*

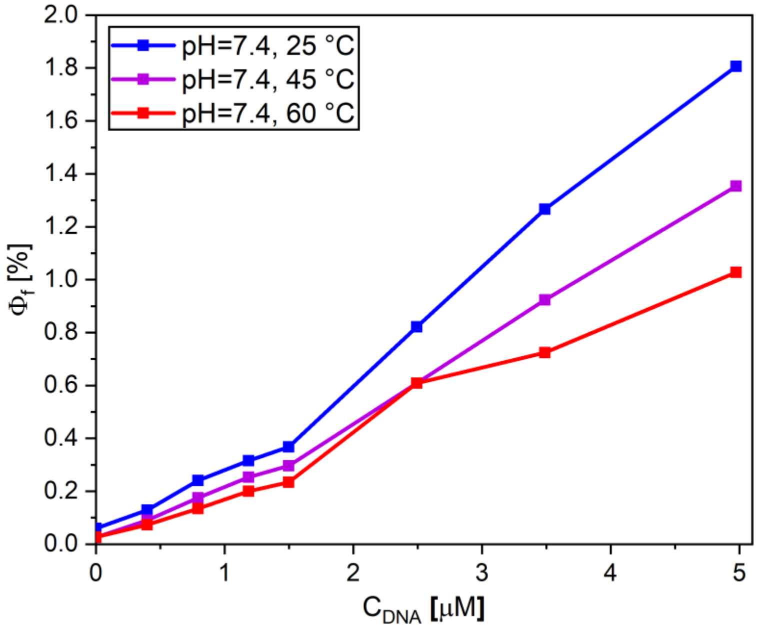


*Figure S162: Fluorescence quantum yield in the presence of plasmid DNA in varying concentrations, measured at different temperatures.*

**Measurement of binding constants**

An attempt was made to determine the binding constant based on the titration curves. For each point of the titration curve, the ratio of the bound dye molecules ($\Theta$) was determined from the effective fluorencence quantum yield measured in the said step ($\Phi_f$), the fluorescence quantum yield of the free dye ($\Phi_f^0$), and the fluorescence quantum yield of the DNA-bound dye, measured in a 50 μM DNA solution ($\Phi_f^{DNA}$):

$$\Theta = \frac{C_{bound\ dye}}{C_{dye}} = \frac{\phi_f - \phi_f^o}{\phi_f^{DNA} - \phi_f^o}$$

This allowed us to determine the average number of bound dye molecules per base pair ($\upsilon$) and the concentration of free dye molecules ($L$), using the dye concentration ($C_{dye}$) and the DNA concentration ($C_{DNA}$, expressed as a concentration of base pairs):

$$\upsilon = \frac{C_{dye} \cdot \Theta}{C_{DNA}}$$

$$L = (1 - \Theta) \cdot C_{dye}$$

According to the model proposed by McGhee and von Hippel[10] $\upsilon/L$ plotted as a function of $\upsilon$ gives a monotonously decreasing curve onto which the following function can be fitted:

$$\frac{\nu}{L} = K_b \cdot (1 - n\upsilon) \cdot \left(\frac{1 - n\upsilon}{1 - (n-1)\upsilon}\right)^{n-1}$$

From this fitting the two parameters in the above equation, the binding constant ($K_b$), and the number of base pairs in one binding site ($n$) can be determined. In the case of these pyrylium dyes the only molecule for which this curve could be fitted was **2,4-DMA-6-Me** ($K_b$=0.25 dm$^3$ μmol$^{-1}$; $n$=1.4). In all other cases of shape of the obtained curve is irregular, implying self-quenching or multimodal binding (Figure S159).

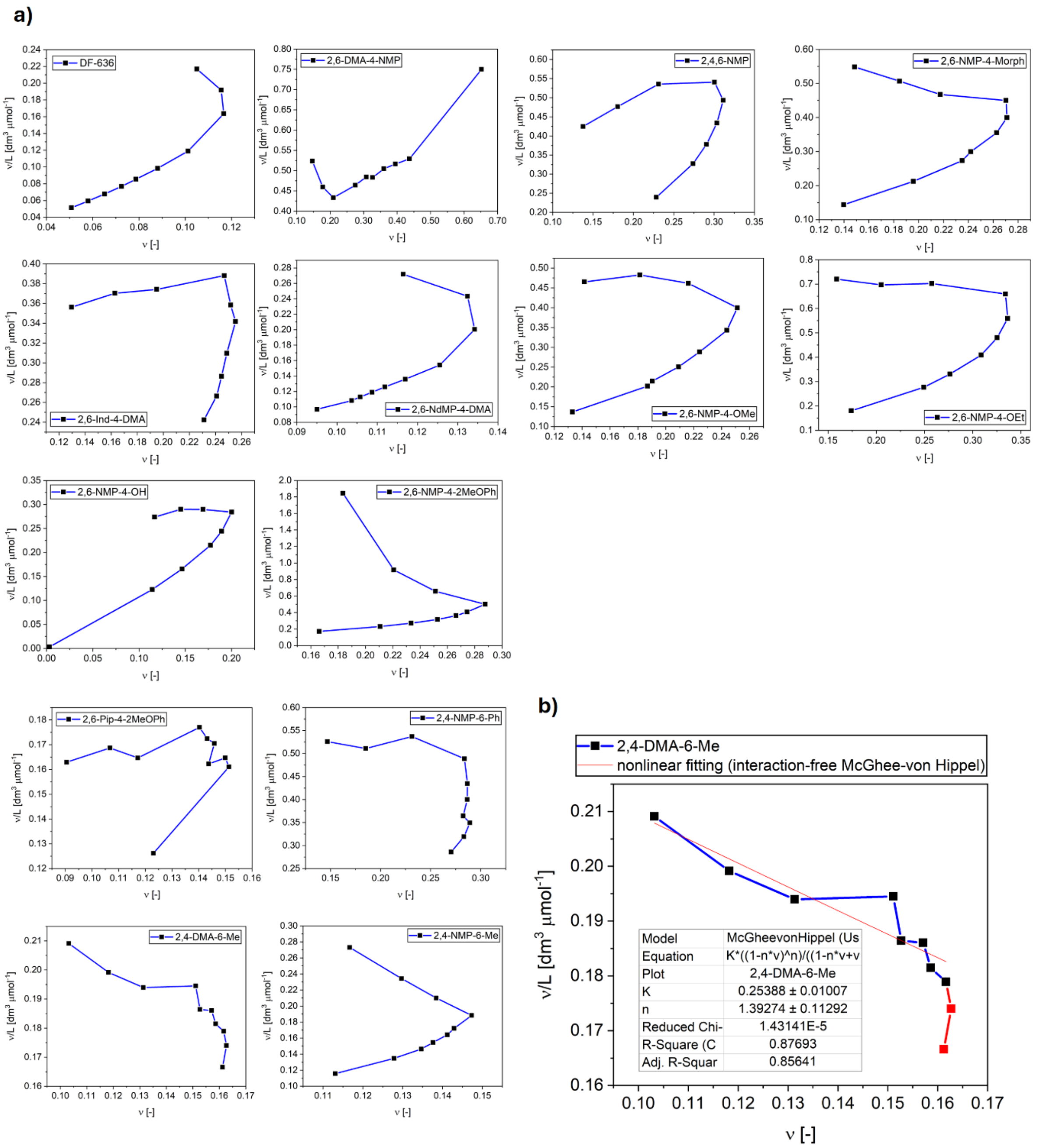


*Figure S163: a) Scatchard plots obtained from the DNA titration experiments. b) Fitting of the McGhee-von Hippel equation on the Scatchard plot of **2,4,-DMA-6-Me**.*

## Determination of LOD (limit of detection) for DNA sensing

*LOD* (limit of detection) for DNA quantitation is defined as the DNA concentration where the effective fluorescence quantum yield of the partially bound dye exceeds the $\Phi_f$ of the non-bound dye by 3$\sigma$, where $\sigma$ is the standard deviation of the measurement of $\Phi_f^0$. It is important to note that since the deviation of measurements depends on the intsrumentation and the concentration of the dye used for DNA quantitation, the measured *LOD* values are sensitive to these parameters as well.

*LOD* values were calculated according to the following formula:

$$LOD = \frac{3\sigma}{\left(\frac{d\phi_f}{dC_{DNA}}\right)_{C_{DNA}=0}} = \frac{3\sigma \cdot \varepsilon}{AFE}$$

*LOD* values were determined for **2,4,6-DMA**, **2,6-NMP-4-DMA**, **2,6-NdMP-4-DMA**, and **2,4-DMA-6-Me** using the AFE values obtained from DNA titration experiments and the standard deviations ($\sigma$) of fluorescence quantum yield ($\Phi_f^0$) measurements, obtained from fivefold measurements of 1 µM dye solutions.

***Table S9****: Standard distribution of quantum yields in pH=7.4 TE buffer from fivefold measurements (σ); absolute fluorescence enhancements (AFE), and limits of detection provided by the dye compounds for the quantitation of dsDNA in our experimental setting.*

| *Compound* | *σ [-]* | *AFE [dm$^6$ cm$^{-1}$ mmol$^{-2}$]* | *LOD [µM]* |
|---|---|---|---|
| **2,4,6-DMA** | $3.7*10^{-5}$ | 31.5 | 0.709 |
| **2,6-NMP-4-DMA** | $5.9*10^{-5}$ | 125.6 | 0.049 |
| **2,6-NdMP-4-DMA** | $9.0*10^{-5}$ | 169.4 | 0.075 |
| **2,4-DMA-6-Me** | $1.8*10^{-5}$ | 433.7 | 0.009 |

### 4.8. Fluorescence lifetime measurements

The steady-state fluorescence spectra and the fluorescence decay curves were measured from a 1 µM solution, on an Edinburgh Instruments FS5 combined steady-state and lifetime spectrometer, which uses the method of time-correlated single-photon counting for measuring the fluorescence decay. The excitation light source was an EPL 450 pulsed diode laser (emitting at 441 nm, with a pulse width of approximately 100 ps) when the fluorescence decay curves were recorded. Mono-, bi- and triexponential curves were fitted to the obtained data and the one with the lowest $\chi^2$ value (biexponential in each case) was selected. In case of **2,6-NdMP-4-DMA** in the presence of 1 µM DNA, the biexponential fit does not describe the decay curve well ($\chi^2$ =2.175) and the reported lifetimes are merely demonstrative.

**Table S10:** Measured fluorescence lifetimes (τ), fractional contributions of the exponential decay components, and the $\chi^2$ values of the biexponential fits.

| **2,6-NdMP-4-DMA** | | | **2,6-NdMP-4-DMA + 1 uM DNA** | | |
|---|---|---|---|---|---|
| $\tau_i$ [ns] | Fractional contribution [%] | $\chi^2$ [-] | $\tau_i$ [ns] | Fractional contribution [%] | $\chi^2$ [-] |
| 0.045 | 92.2 | 1.628 | *0.48* | *41.3* | 2.175 |
| 1.36 | 7.8 | | *1.84* | *58.7* | |

| **2,4,6-DMA in MeOH** | | | **2,4,6-DMA in THF** | | |
|---|---|---|---|---|---|
| $\tau_i$ [ns] | Fractional contribution [%] | $\chi^2$ [-] | $\tau_i$ [ns] | Fractional contribution [%] | $\chi^2$ [-] |
| 0.33 | 92.8 | 1.219 | 1.19 | 46.5 | 1.198 |
| 0.67 | 7.2 | | 1.48 | 53.5 | |

## 5. Testing in agarose gel electrophoresis

### 5.1. Materials and methods

The experiments were performed using 1% agarose gel prepared with 50 mL UltraPure™ TAE Buffer (1X) and stained with either 6 µL of 6 mM or 15 µL of 2 mM gel stain. Thermo Scientific™ DNA Gel

Loading Dye (6X) and GeneRuler 1 kb DNA Ladder were used for sample loading and DNA fragment size estimation. Electrophoresis was carried out at 120 V, 87 mA, and 11 W for 20 minutes. The gels were then illuminated and photographed using UVITEC's Uvidoc HD6 gel documentation system.

### 5.2. Gel electrophoresis images

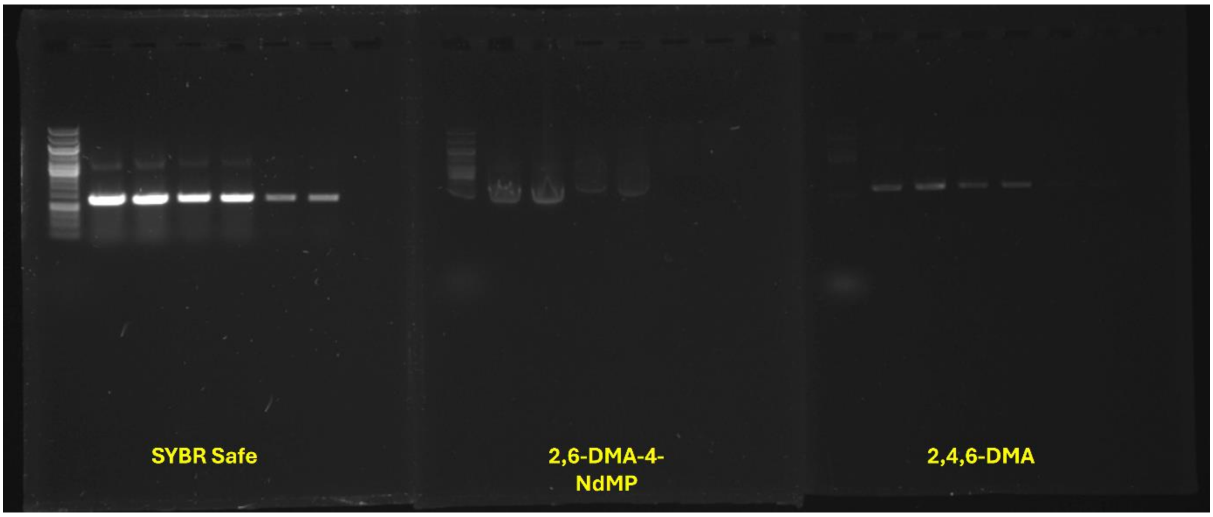


*Figure S165. Comparison of* ***SYBR Safe*** *(control)* ***2,6-DMA-4-NdMP****, and* ***2,4,6-DMA*** *for agarose gel staining. Brightness was adjusted for better visibility.*

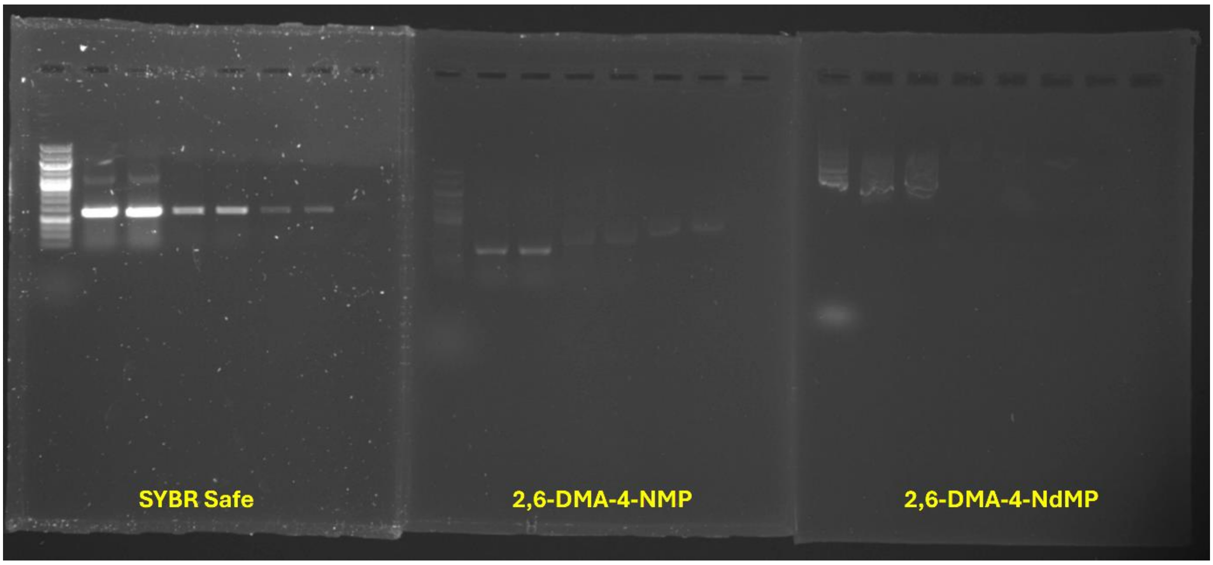


*Figure S166. Comparison of* ***SYBR Safe*** *(control)* ***2,6-DMA-4-NMP****, and* ***2,6-DMA-4-NdMP*** *for agarose gel staining. Brightness was adjusted for better visibility.*

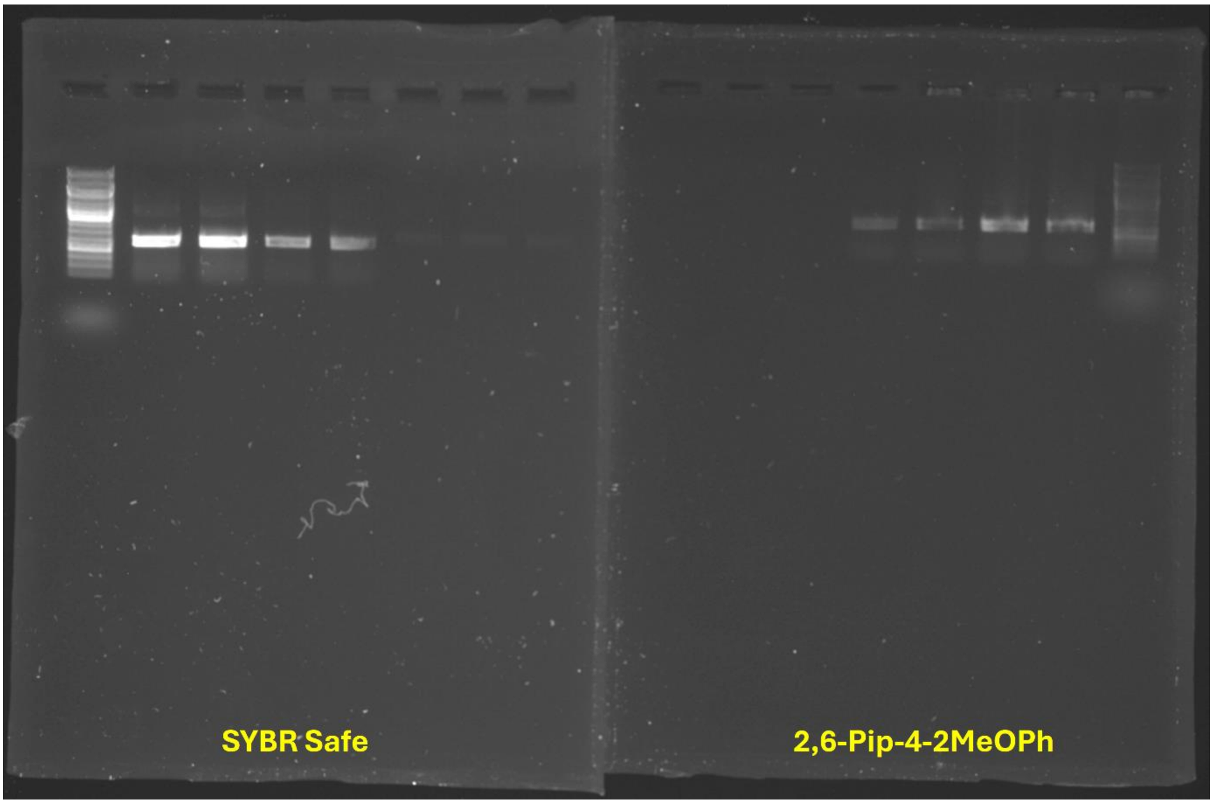


*Figure S167: Comparison of* ***SYBR Safe*** *(control) and* ***2,6-Pip-4-2MeOPh*** *for agarose gel staining. Brightness was adjusted for better visibility.*

### 5.3. Quantification of the gel electrophoresis results

The experiment used for quantification was performed using 0,5% agarose gel prepared with 50 mL UltraPure™ TAE Buffer (1X) and stained with either 6 µL of 5 mM (**SYBR Safe**) or 15 µL of 2 mM (**2,6-Pip-4-2MeOPh**) gel stain. Thermo Scientific™ DNA Gel Loading Dye (6X) and GeneRuler 1 kb DNA Ladder were used for sample loading and DNA fragment size estimation. The DNA samples contained 240, 120, and 60 ng double-stranded DNA (enzymatically cut and purified dsDNA fragment) with 2 parallel measurements taken for each sample. Electrophoresis was carried out at 100 V, 87 mA, and 11 W for 40 minutes. The gels were then illuminated and photographed using UVITEC's Uvidoc HD6 gel documentation system.

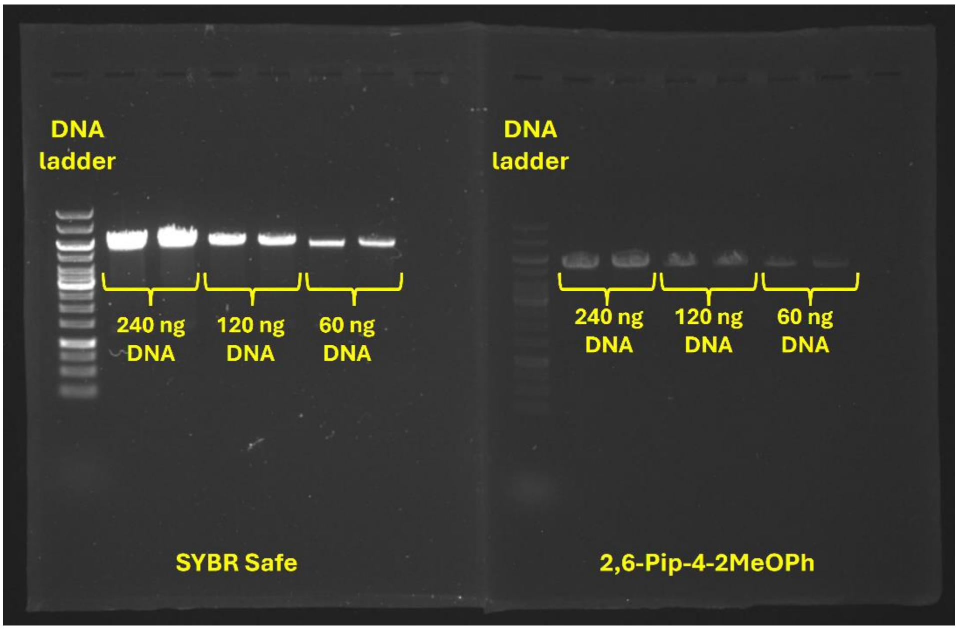


*Figure S168: Comparison of* ***SYBR Safe*** *(control) and* ***2,6-Pip-4-2MeOPh*** *for agarose gel staining. Brightness and contrast were adjusted for optimal visibility.*

The gel electrophoresis image displayed as Figure S168 was analyzed using the Gel Analyzer option of ImageJ. The obtained peaks in corresponding lanes were integrated after baseline correction. *Intensity ratio* shows the ratio of the peak integral of **2,6-Pip-4-2MeOPh** and the average of peak integrals on **SYBR Safe** from two parallel measurements conducted with the same quantity of DNA (Figure S165).

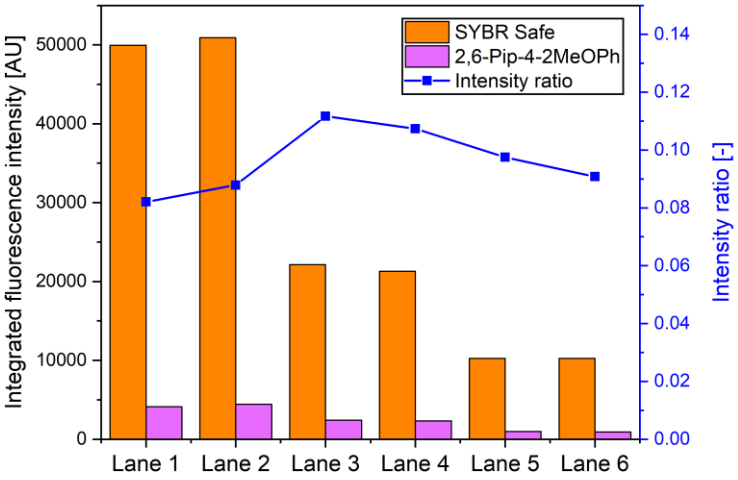


*Figure S169. Integrated peak intensities of the corresponding DNA samples, analyzed using different gel stains (****SYBR Safe*** *and* ***2,6-Pip-4-2MeOPh****).*

***Table S11:*** *Average relative deviations of the peak intensities in the agarose gel staining of DNA samples using different dyes (Figure S168).*

| Compound | Average relative deviation of peak intensity |
|---|---|
| **SYBR Safe** | 0.99% |
| **2,6-Pip-4-2MeOPh** | 2.99% |

# 6. Theoretical studies

## 6.1. Computational methods

Computational modelling of molecular geometry and photochemical properties

Theoretical computations were carried out using the Gaussian16[1], using standard convergence criteria. Optimization and vibrational frequency calculations were performed with the B3LYP method[2] using the 6-31G(d,p) basis set and the IEFPCM method (relative permittivity = 78.3553 for water)[3]. The B3LYP method proved to be the most accurate in the calculation of emission and excitation wavelengths among the applied several DFT methods. The larger basis set does not provide any advantages. Thermodynamic functions were computed at 298.15 K. For wavelength prediction, the vertical excitation was modelled by the TD-B3LYP/6-31G(d,p)[PCM(solvent)] level of theory using the optimized geometries. The emission wavelengths were calculated after optimization using geometries provided by TD-B3LYP/6-31G(d,p)[PCM(solvent)]. The grid size and the convergence criteria for DFT calculation were set as default.

### Triplet state

As a competitive quenching process, the triplet curves were also calculated. As the triplet curve crosses the line of the single excited state, two triplet states were also considered as $T_1$(ISC) and $T_1$(opt). The $T_1$(ISC) is not a stationary point, but the intersection of $S_1$ and $T_1$ curves, where the excited structures slide down on the $S_1$ curve and can switch the electron spin than change to $T_1$.

## 6.2. Molecular orbitals

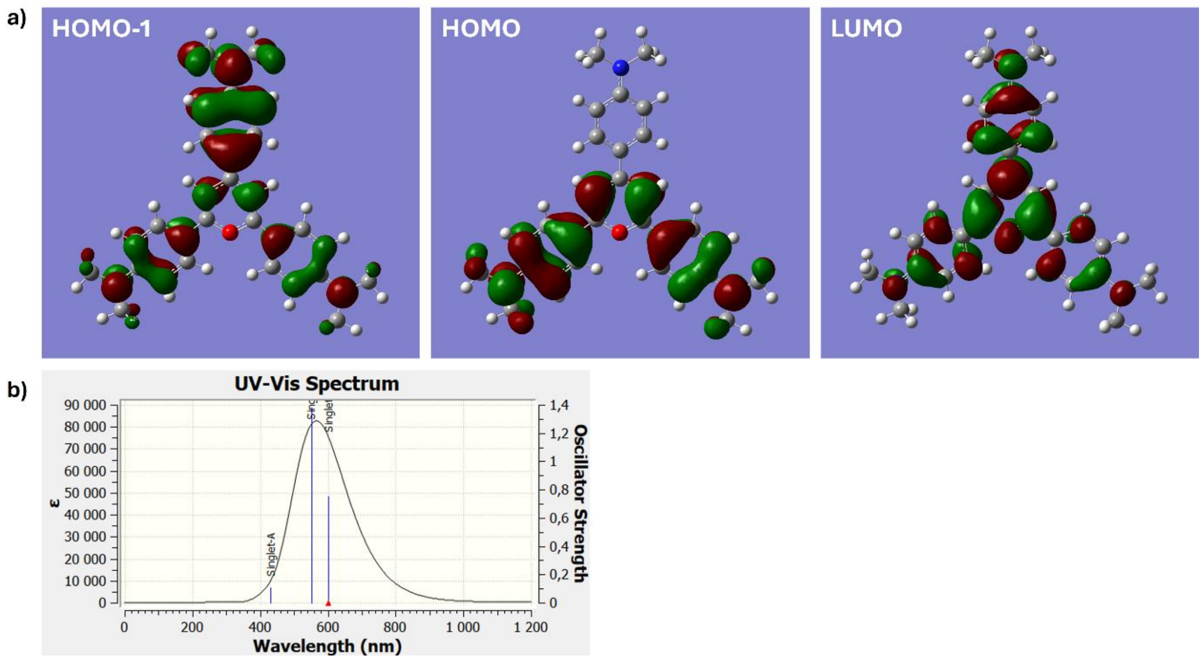


*Figure S170: a) Calculated HOMO-1, HOMO, and LUMO of **2,4,6-DMA**, calculated at a TD-B3LYP/6-31G(d,p)[PCM(water)] level of theory. b) Calculated UV-Vis spectrum of **2,4,6-DMA**. The S0→ S1 excitation corresponds to the HOMO→LUMO transition, while the S0→ S2 excitation corresponds to HOMO-1→LUMO.*

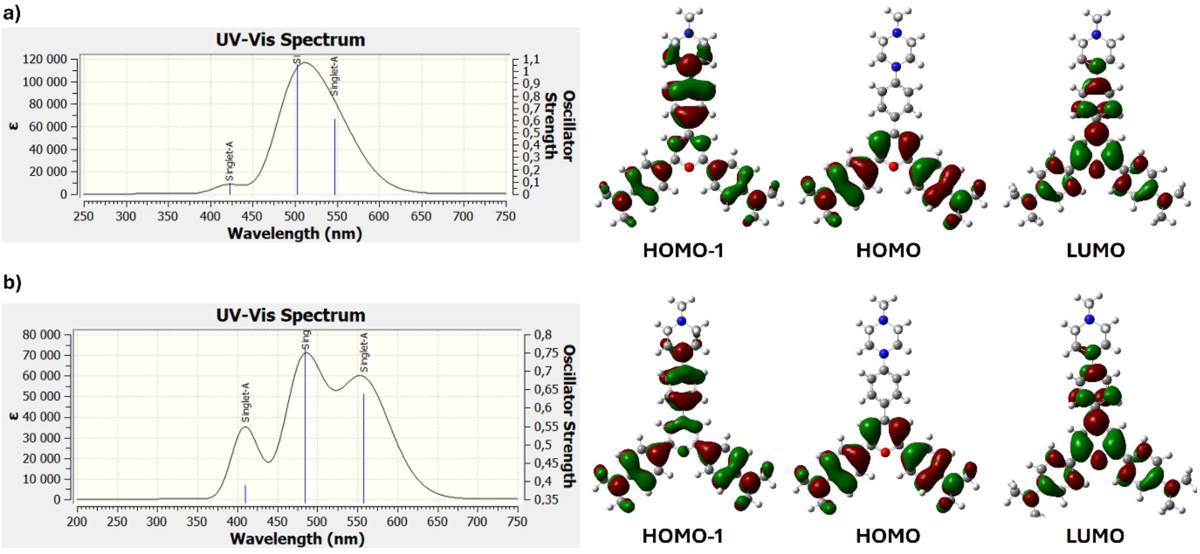


*Figure S171: Calculated UV-Vis spectrum and HOMO-1, HOMO, and LUMO of **2,6-DMA-4-NMP**, not protonated (**a**) or protonated (**b**) on the aliphatic amino group (calculated at a TD-B3LYP/6-31G(d,p)[PCM(water)] level of theory). In each case, S0→ S1 excitation corresponds to the HOMO→LUMO transition, while the S0→ S2 excitation corresponds to HOMO-1→LUMO.*

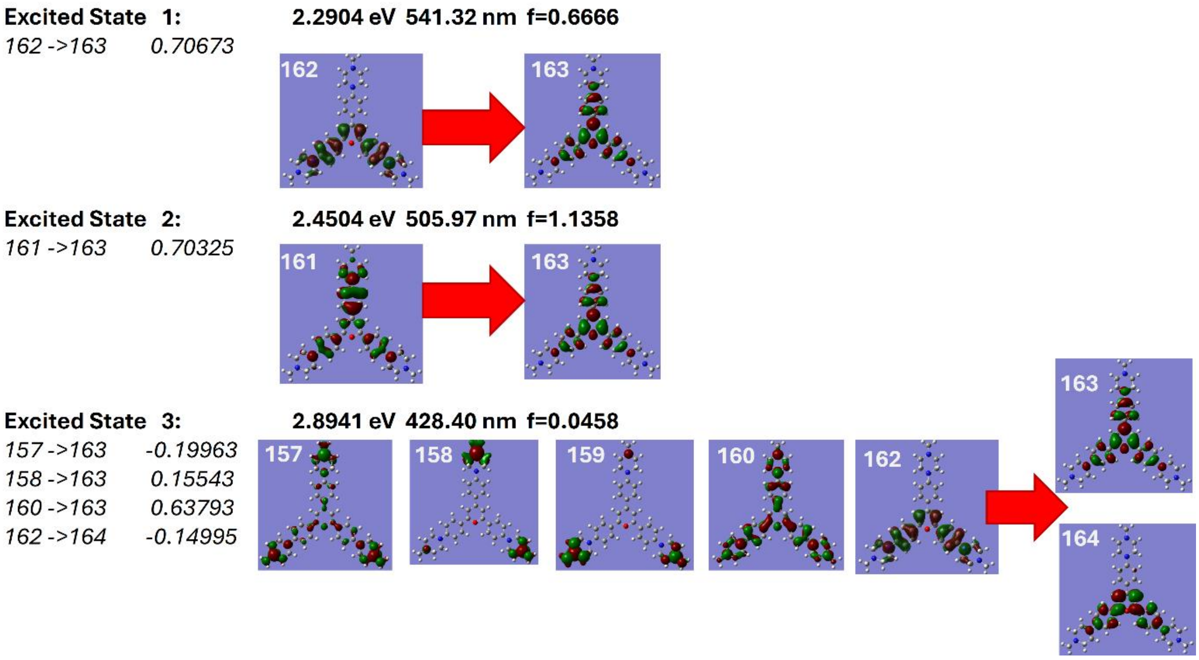


*Figure S172: The first three excited states of **2,4,6-DMA** calculated at a TD-B3LYP/6-31G(d,p)[PCM(water)] level of theory. For each excited state, the excitation energies, calculated wavelengths and oscillator strength (f) are written in bold, the molecular orbitals with the largest coefficient in the CI expansion along with the corresponding transition coefficients are written in italic, and the relevant molecular orbitals are displayed.*

## 6.3. Scanning data

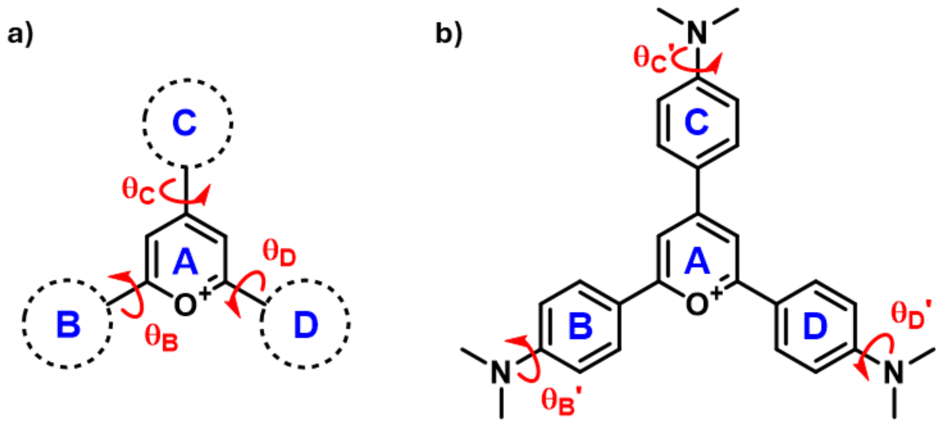


*Figure S173: a) $\theta_B$, $\theta_C$ and $\theta_D$ torsional angles of the model compounds. b) $\theta_B$', $\theta_C$' and $\theta_D$' torsional angles of* ***2,4,6-DMA****.*

The stepwise DFT scanning procedure begins from the initial position at $\theta_B$= 0º (or θc = 0º), which is not necessarily the local minimum. The scan progresses in 5, 10, or 18° increments increments up to 180º along the selected internal coordinate, while all other geometric parameters remain unconstrained.

The following compounds were scanned aroung the $\theta_B$ and $\theta_C$ torsional angles in $S_0$, $S_1$, and $T_1$ states, using B3LYP/6-31G(d,p)[PCM(solvent)] level of theory for ground states and TD-B3LYP/6-31G(d,p)[PCM(solvent)] for excited states. The modeled solvent was water, unless stated otherwise.

**Table S12:** Calculated structures.

| | |
|---|---|
| **2,4,6-DMA**<br>• modeled media: vacuo, toluene, THF, water | 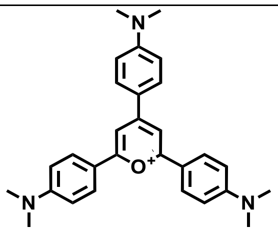 |
| **2,6-NMP-4-DMA** | 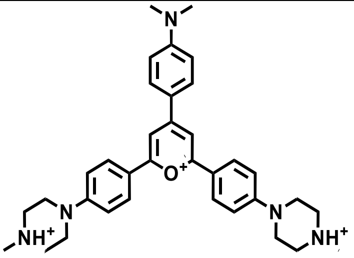 |
| **2,4,6-NMP** | 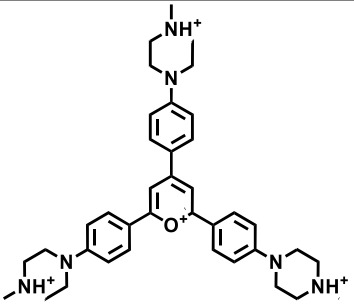 |
| **2,6-NMP-4-Morph** | 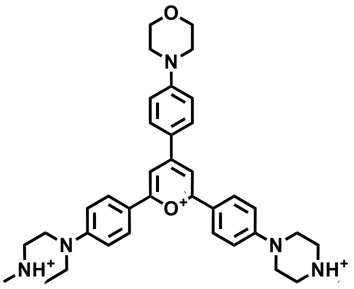 |
| **2,6-NdMP-4-DMA** | 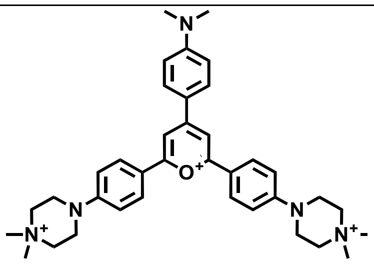 |
| **2,6-NMP-4-OMe** | 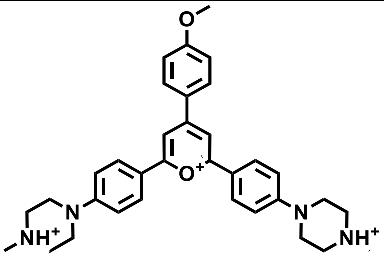 |

| | |
|---|---|
| **2,6-NMP-4-2MeOPh** | 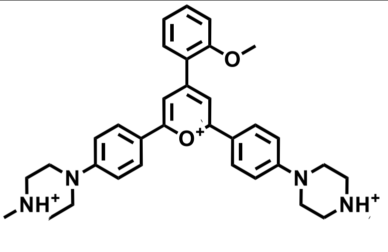 |
| **2,4-DMA-6-Me** | 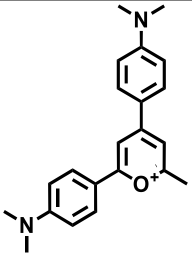 |
| **2,4-NMP-6-Me** | 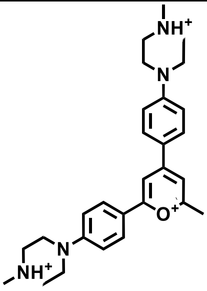 |
| **2,6-Ph-4-DMA** / 4-(4-dimethylaminophenyl)-2,6-diphenylpyrylium | 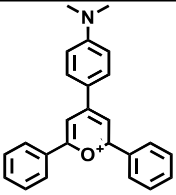 |

Scanning energies (ΔE) are given in kJ/mol, with taking the $S_0$ energy minimum as zero.

Furthermore, for the **2,4,6-DMA** compound the following calculations were conducted:

- Scanning around the $\theta_B$’ and $\theta_C$’ torsional angles in 10° increments in $S_0$, $S_1$, and $T_1$ states using the same method (oscillator strengths were also calculated).
- Scanning around the $\theta_B$ and $\theta_C$ torsional angles using a different implicit solvation (namely, vacuo, toluene, THF, and water), using B3LYP/6-31G(d,p)[PCM(solvent)] level of theory for ground states and TD-B3LYP/6-31G(d,p)[PCM(solvent)] for excited states (oscillator strengths were also calculated).

To model the expected protonation of amino groups at pH=7.4, in all compouds containing aliphatic amino groups these nitrogen atoms were considered to be protonated.

**Table S13:** Scanning data for the $S_0$ states around the $\theta_B$ angle (calculated energies in kJ/mol):

| $\theta_B$ [deg.] | **2,4,6-DMA** | **2,6-NMP-4-DMA** | **2,4,6-NMP** | **2,6-NMP-4-Morph** | **2,6-NdMP-4-DMA** | **2,6-NMP-4-OMe** | **2,6-NMP-4-2MeOPh** | **2,4-DMA-6-Me** | **2,4-NMP-6-Me** |
|---|---|---|---|---|---|---|---|---|---|
| 0 | 0.50 | 0.00 | 0.00 | 0.00 | 0.00 | 0.00 | 0.00 | 0.00 | 0.00 |
| 10 | 0.00 | 1.03 | -0.85 | -0.91 | 0.76 | -0.96 | -0.66 | 0.40 | 0.00 |
| 20 | 0.61 | 1.30 | -0.50 | -0.89 | 1.57 | -0.98 | -0.53 | 1.51 | 0.77 |
| 30 | 2.88 | 2.24 | 0.96 | 0.28 | 2.74 | 0.56 | 0.91 | 4.04 | 2.47 |
| 40 | 7.01 | 4.78 | 4.27 | 3.21 | 4.84 | 3.48 | 3.96 | 8.21 | 6.05 |
| 50 | 12.70 | 8.72 | 9.51 | 7.73 | 8.92 | 8.37 | 9.17 | 14.02 | 11.23 |
| 60 | 19.14 | 13.42 | 14.67 | 12.93 | 13.98 | 13.93 | 14.78 | 20.94 | 17.31 |
| 70 | 25.30 | 18.93 | 20.24 | 18.72 | 19.50 | 19.88 | 20.81 | 27.99 | 23.48 |
| 80 | 29.83 | 22.96 | 25.00 | 23.61 | 23.58 | 24.82 | 25.74 | 33.73 | 28.54 |
| 90 | 31.34 | 24.60 | 26.78 | 25.22 | 25.14 | 26.83 | 27.73 | 36.10 | 30.50 |
| 100 | 29.83 | 22.49 | 25.24 | 23.61 | 23.11 | 24.82 | 25.74 | 33.41 | 28.54 |
| 110 | 25.30 | 18.22 | 20.81 | 18.72 | 18.66 | 19.88 | 20.81 | 27.18 | 23.48 |
| 120 | 19.14 | 12.74 | 14.91 | 12.93 | 13.01 | 13.93 | 14.78 | 19.83 | 17.31 |
| 130 | 12.70 | 7.17 | 9.24 | 7.73 | 7.65 | 8.37 | 9.17 | 12.95 | 11.23 |
| 140 | 7.01 | 2.65 | 4.27 | 3.21 | 3.32 | 3.48 | 3.96 | 7.31 | 6.05 |
| 150 | 2.88 | -0.07 | 0.96 | 0.28 | 0.28 | 0.56 | 0.91 | 3.30 | 2.47 |
| 160 | 0.61 | -1.15 | -0.50 | -0.89 | -0.89 | -0.98 | -0.53 | 0.97 | 0.77 |
| 170 | 0.00 | -1.10 | -0.85 | -0.91 | -0.61 | -0.96 | -0.66 | 0.08 | 0.00 |
| 180 | 0.50 | -0.10 | 0.00 | 0.00 | 0.07 | 0.00 | 0.00 | 0.00 | 0.00 |

**Table S14:** Scanning data for the $S_0$ states around the $\theta_C$ angle (calculated energies in kJ/mol):

| $\theta_C$ [deg.] | **2,4,6-DMA** | **2,6-NMP-4-DMA** | **2,4,6-NMP** | **2,6-NMP-4-Morph** | **2,6-NdMP-4-DMA** | **2,6-NMP-4-OMe** | **2,6-NMP-4-2MeOPh** |
|---|---|---|---|---|---|---|---|
| 0 | 0.00 | 0.00 | 1.00 | 0.00 | 2.70 | 0.00 | 0.00 |
| 10 | -1.01 | -0.86 | 1.00 | -1.01 | 1.44 | -0.51 | -1.06 |
| 20 | -1.60 | -1.14 | 0.00 | -1.51 | 0.01 | -0.69 | -1.85 |
| 30 | -0.56 | 0.28 | 0.41 | -0.42 | 0.00 | 0.88 | -0.80 |
| 40 | 2.85 | 4.17 | 2.91 | 3.01 | 2.17 | 4.69 | 2.08 |
| 50 | 8.46 | 10.44 | 7.43 | 8.66 | 6.39 | 10.78 | 6.80 |
| 60 | 15.61 | 18.53 | 13.73 | 16.11 | 12.04 | 18.50 | 12.98 |
| 70 | 23.21 | 27.14 | 19.79 | 23.18 | 18.12 | 26.78 | 19.58 |
| 80 | 29.54 | 34.31 | 24.59 | 29.23 | 23.08 | 33.80 | 24.95 |
| 90 | 32.30 | 37.11 | 26.61 | 32.01 | 25.11 | 36.77 | 27.29 |
| 100 | 29.94 | 34.46 | 24.59 | 29.23 | 23.08 | 33.65 | 25.46 |
| 110 | 23.62 | 27.48 | 19.79 | 23.18 | 18.12 | 26.60 | 20.16 |
| 120 | 16.15 | 19.15 | 13.73 | 16.11 | 12.04 | 18.36 | 14.05 |
| 130 | 9.19 | 11.39 | 7.43 | 8.66 | 6.39 | 10.70 | 7.69 |
| 140 | 3.81 | 5.30 | 2.91 | 3.01 | 2.17 | 4.69 | 2.71 |
| 150 | 0.52 | 1.42 | 0.41 | -0.42 | 0.00 | 0.89 | 0.06 |
| 160 | -0.56 | -0.12 | 0.00 | -1.51 | 0.01 | -0.69 | -0.98 |
| 170 | -0.25 | -0.15 | 1.00 | -1.01 | 1.44 | -0.50 | -0.43 |
| 180 | 0.00 | 0.01 | 1.00 | 0.00 | 2.70 | 0.00 | 0.16 |

**Table S15:** Scanning data for the $S_0$ states around the $\theta_C$ angle (continuation). Calculated energies in kJ/mol:

| $\theta_C$ [deg.] | **2,6-NdMP-4-DMA** |
|---|---|
| 0 | 0.00 |
| 5 | -0.46 |
| 10 | -0.88 |
| 15 | -1.17 |
| 20 | -1.16 |
| 25 | -0.71 |
| 30 | 0.26 |
| 35 | 1.88 |
| 40 | 4.18 |
| 45 | 6.98 |
| 50 | 10.34 |
| 55 | 14.14 |
| 60 | 18.25 |
| 65 | 22.50 |
| 70 | 26.67 |
| 75 | 30.58 |
| 80 | 33.87 |
| 85 | 36.14 |
| 90 | 37.06 |
| 95 | 36.53 |
| 100 | 34.29 |
| 105 | 31.08 |
| 110 | 27.23 |
| 115 | 23.13 |
| 120 | 19.02 |
| 125 | 15.07 |
| 130 | 11.36 |
| 135 | 8.10 |
| 140 | 5.30 |
| 145 | 3.08 |
| 150 | 1.49 |
| 155 | 0.48 |
| 160 | -0.06 |
| 165 | -0.20 |
| 170 | -0.13 |
| 175 | -0.02 |
| 180 | -0.01 |

| $\theta_C$ [deg.] | **2,6-NMP-4-2MeOPh** |
|---|---|
| 0 | 8.14 |
| 18 | 2.33 |
| 36 | 0.00 |
| 54 | 4.30 |
| 72 | 11.85 |
| 90 | 16.21 |
| 108 | 11.85 |
| 126 | 4.30 |
| 144 | 0.00 |
| 162 | 2.33 |
| 180 | 8.14 |

**Table S16:** Scanning data for the $S_1$ states around the $\theta_B$ angle (calculated energies in kJ/mol):

| $\theta_B$ [deg.] | 2,4,6-DMA | 2,6-NMP-4-DMA | 2,4,6-NMP | 2,6-NdMP-4-DMA | 2,6-NMP-4-OMe | 2,6-NMP-4-2MeOPh | 2,4-DMA-6-Me | 2,4-NMP-6-Me |
|---|---|---|---|---|---|---|---|---|
| 0 | 204.2 | 186.7 | 212.2 | 187.0 | 212.2 | 209.5 | 211.4 | 224.2 |
| 10 | 204.0 | 186.6 | 212.9 | 187.6 | 212.1 | 209.3 | 211.3 | 224.0 |
| 20 | 204.9 | 186.7 | 213.3 | 187.8 | 212.5 | 209.8 | 211.4 | 223.9 |
| 30 | 207.1 | 188.4 | 214.3 | 189.4 | 214.4 | 211.0 | 212.4 | 225.0 |
| 40 | 210.7 | 190.9 | 216.8 | 192.5 | 216.8 | 214.1 | 213.9 | 226.1 |
| 50 | 214.9 | 194.4 | 220.3 | 196.6 | 220.2 | 217.5 | 215.6 | 227.5 |
| 60 | 218.9 | 199.1 | 222.8 | 201.6 | 223.6 | 221.0 | 216.9 | 228.2 |
| 70 | 221.4 | 203.9 | 225.0 | 206.1 | 225.4 | 223.2 | 216.7 | 226.9 |
| 80 | 222.3 | 207.5 | 226.1 | 209.0 | 226.0 | 223.4 | 216.4 | 226.1 |
| 90 | 222.3 | 209.1 | 226.3 | 210.3 | 226.4 | 224.0 | 215.5 | 225.7 |
| 100 | 222.3 | 207.5 | 226.3 | 208.9 | 226.0 | 223.4 | 215.0 | 226.1 |
| 110 | 221.4 | 204.0 | 225.8 | 205.3 | 225.4 | 223.2 | 215.6 | 226.9 |
| 120 | 218.9 | 199.4 | 224.0 | 200.9 | 223.6 | 221.0 | 216.8 | 228.2 |
| 130 | 214.9 | 194.3 | 221.0 | 196.2 | 220.2 | 217.5 | 217.0 | 227.5 |
| 140 | 210.7 | 190.1 | 217.3 | 192.2 | 216.8 | 214.1 | 216.0 | 226.1 |
| 150 | 207.1 | 187.4 | 214.0 | 189.0 | 214.4 | 211.0 | 214.4 | 225.0 |
| 160 | 204.9 | 186.1 | 212.5 | 187.4 | 212.5 | 209.8 | 212.7 | 223.9 |
| 170 | 204.0 | 186.1 | 212.0 | 187.0 | 212.1 | 209.3 | 211.4 | 224.0 |
| 180 | 204.2 | 186.0 | 212.5 | 187.6 | 212.2 | 209.5 | 211.0 | 224.2 |

| $\theta_B$ [deg.] | 2,6-NMP-4-Morph |
|---|---|
| 0 | 186.1 |
| 5 | 186.4 |
| 10 | 186.6 |
| 15 | 186.3 |
| 20 | 186.6 |
| 25 | 187.2 |
| 30 | 188.2 |
| 35 | 189.4 |
| 40 | 191.0 |
| 45 | 193.0 |
| 50 | 195.4 |
| 55 | 197.4 |
| 60 | 199.5 |
| 65 | 202.0 |
| 70 | 204.0 |
| 75 | 205.9 |
| 80 | 207.4 |
| 85 | 208.3 |
| 90 | 208.6 |
| 95 | 208.3 |
| 100 | 207.4 |
| 105 | 205.9 |
| 110 | 204.0 |
| 115 | 202.0 |
| 120 | 199.5 |
| 125 | 197.4 |
| 130 | 195.4 |
| 135 | 193.0 |
| 140 | 191.0 |
| 145 | 189.4 |
| 150 | 188.2 |
| 155 | 187.2 |
| 160 | 186.6 |
| 165 | 186.3 |
| 170 | 186.6 |
| 175 | 186.4 |
| 180 | 186.1 |

**Table S17:** Scanning data for the $S_1$ states around the $\theta_C$ angle (calculated energies in kJ/mol):

| $\theta_B$ [deg.] | **2,4,6-DMA** | **2,6-NMP-4-DMA** | **2,4,6-NMP** | **2,6-NMP-4-OMe** | **2,4-DMA-6-Me** | **2,4-NMP-6-Me** |
|---|---|---|---|---|---|---|
| 0 | 203.3 | 220.3 | 211.6 | 214.6 | 201.1 | 222.6 |
| 10 | 202.8 | 218.8 | 210.7 | 213.9 | 201.4 | 222.4 |
| 20 | 202.8 | 215.8 | 210.2 | 213.4 | 202.6 | 222.4 |
| 30 | 204.2 | 212.7 | 211.1 | 214.3 | 204.7 | 224.1 |
| 40 | 207.5 | 210.1 | 214.1 | 217.0 | 207.4 | 227.1 |
| 50 | 212.4 | 206.7 | 218.8 | 221.5 | 210.5 | 226.8 |
| 60 | 210.2 | 201.1 | 224.5 | 227.0 | 211.7 | 222.8 |
| 70 | 205.3 | 194.0 | 230.2 | 232.8 | 212.7 | 217.1 |
| 80 | 201.4 | 188.5 | 235.2 | 237.3 | 213.7 | 212.5 |
| 90 | 200.2 | 186.5 | 237.5 | 239.1 | 214.8 | 210.9 |
| 100 | 201.9 | 188.1 | 235.6 | 237.3 | 213.7 | 212.6 |
| 110 | 204.9 | 193.2 | 231.1 | 232.8 | 212.7 | 217.5 |
| 120 | 209.4 | 199.6 | 225.1 | 227.0 | 211.7 | 223.3 |
| 130 | 212.6 | 205.5 | 219.5 | 221.5 | 210.5 | 227.0 |
| 140 | 208.2 | 209.3 | 215.1 | 217.0 | 207.4 | 226.8 |
| 150 | 204.9 | 212.3 | 212.5 | 214.3 | 204.7 | 224.4 |
| 160 | 203.5 | 215.7 | 211.1 | 213.4 | 202.6 | 222.6 |
| 170 | 203.4 | 219.0 | 211.3 | 213.9 | 201.4 | 222.4 |
| 180 | 203.5 | 221.0 | 211.4 | 214.6 | 201.1 | 222.7 |

**Table S18:** Scanning data for the $S_1$ states around the $\theta_C$ angle (continuation). Calculated energies in kJ/mol:

| $\theta_C$ [deg.] | **2,6-NMP-4-Morph** | **2,6-NdMP-4-DMA** |
|---|---|---|
| 0 | 217.4 | 220.0 |
| 5 | 216.8 | 219.6 |
| 10 | 215.4 | 218.7 |
| 15 | 213.7 | 217.4 |
| 20 | 212.1 | 215.8 |
| 25 | 210.6 | 214.2 |
| 30 | 209.2 | 212.8 |
| 35 | 208.0 | 211.5 |
| 40 | 206.9 | 210.1 |
| 45 | 205.4 | 208.5 |
| 50 | 203.5 | 206.6 |
| 55 | 201.1 | 204.1 |
| 60 | 198.2 | 200.9 |
| 65 | 194.8 | 197.3 |
| 70 | 191.6 | 193.8 |
| 75 | 188.8 | 190.7 |
| 80 | 186.8 | 188.3 |
| 85 | 185.4 | 186.8 |
| 90 | 185.0 | 186.4 |
| 95 | 185.4 | 186.9 |
| 100 | 186.8 | 188.2 |
| 105 | 188.8 | 190.4 |
| 110 | 191.6 | 193.3 |
| 115 | 194.8 | 196.6 |
| 120 | 198.2 | 200.0 |
| 125 | 201.1 | 203.0 |
| 130 | 203.5 | 205.7 |
| 135 | 205.4 | 207.8 |
| 140 | 206.9 | 209.5 |
| 145 | 208.0 | 211.0 |
| 150 | 209.2 | 212.4 |
| 155 | 210.6 | 214.1 |
| 160 | 212.1 | 215.8 |
| 165 | 213.7 | 217.5 |
| 170 | 215.4 | 219.2 |
| 175 | 216.8 | 219.9 |
| 180 | 217.4 | 220.1 |

| $\theta_C$ [deg.] | **2,6-NMP-4-2MeOPh** |
|---|---|
| 0 | 206.6 |
| 18 | 202.6 |
| 36 | 202.3 |
| 54 | 207.4 |
| 72 | 216.2 |
| 90 | 221.6 |
| 108 | 216.2 |
| 126 | 207.4 |
| 144 | 202.3 |
| 162 | 202.6 |
| 180 | 206.6 |

**Table S19:** Scanning data for the $T_1$ states around the $\theta_B$ angle (calculated energies in kJ/mol):

| $\theta_B$ [deg.] | 2,4,6-DMA | 2,6-NMP-4-DMA | 2,4,6-NMP | 2,6-NMP-4-Morph | 2,6-NdMP-4-DMA | 2,6-NMP-4-OMe | 2,6-NMP-4-2MeOPh | 2,4-DMA-6-Me | 2,4-NMP-6-Me |
|---|---|---|---|---|---|---|---|---|---|
| 0 | 172.9 | 169.0 | 181.4 | 166.0 | 168.4 | 181.2 | 177.1 | 172.39 | 189.0 |
| 10 | 172.8 | 168.8 | 182.3 | 165.9 | 169.1 | 181.7 | 177.0 | 172.70 | 187.3 |
| 20 | 172.9 | 169.0 | 182.8 | 166.2 | 169.0 | 182.7 | 178.2 | 173.85 | 188.9 |
| 30 | 174.4 | 170.5 | 184.9 | 167.4 | 170.3 | 185.0 | 180.1 | 176.38 | 192.3 |
| 40 | 177.5 | 172.3 | 188.8 | 170.1 | 173.1 | 188.7 | 183.9 | 180.51 | 196.1 |
| 50 | 181.9 | 176.0 | 193.2 | 174.1 | 177.0 | 193.8 | 189.4 | 186.27 | 201.0 |
| 60 | 187.3 | 180.7 | 199.0 | 179.1 | 182.0 | 199.8 | 195.7 | 193.15 | 206.3 |
| 70 | 192.6 | 186.0 | 205.1 | 183.9 | 187.3 | 205.7 | 202.1 | 200.28 | 212.0 |
| 80 | 196.8 | 189.9 | 209.9 | 188.3 | 191.1 | 210.8 | 207.4 | 206.21 | 216.5 |
| 90 | 198.6 | 191.6 | 212.0 | 190.0 | 192.5 | 213.1 | 209.7 | 208.78 | 218.2 |
| 100 | 196.8 | 189.8 | 210.6 | 188.3 | 190.7 | 210.8 | 207.4 | 206.72 | 216.5 |
| 110 | 192.6 | 185.9 | 206.1 | 183.9 | 186.6 | 205.7 | 202.1 | 200.94 | 212.0 |
| 120 | 187.3 | 181.0 | 200.1 | 179.1 | 181.4 | 199.8 | 195.7 | 193.73 | 206.3 |
| 130 | 181.9 | 175.8 | 194.3 | 174.1 | 176.4 | 193.8 | 189.4 | 186.87 | 201.0 |
| 140 | 177.5 | 171.6 | 189.0 | 170.1 | 172.3 | 188.7 | 183.9 | 181.16 | 196.1 |
| 150 | 174.4 | 168.7 | 185.3 | 167.4 | 169.4 | 185.0 | 180.1 | 176.92 | 192.3 |
| 160 | 172.9 | 167.5 | 182.4 | 166.2 | 168.4 | 182.7 | 178.2 | 174.19 | 188.9 |
| 170 | 172.8 | 167.5 | 181.5 | 165.9 | 168.2 | 181.7 | 177.0 | 172.85 | 187.3 |
| 180 | 172.9 | 167.7 | 181.5 | 166.0 | 168.9 | 181.2 | 177.1 | 172.39 | 189.0 |

**Table S20:** Scanning data for the $T_1$ states around the $\theta_C$ angle (calculated energies in kJ/mol):

| $\theta_C$ [degree.] | 2,4,6-DMA | 2,6-NMP-4-DMA | 2,4,6-NMP | 2,6-NMP-4-Morph | 2,6-NMP-4-OMe | 2,4-DMA-6-Me | 2,4-NMP-6-Me |
|---|---|---|---|---|---|---|---|
| 0 | 172.9 | 169.6 | 181.7 | 166.8 | 184.4 | 172.08 | 185.7 |
| 10 | 173.7 | 168.5 | 180.7 | 165.8 | 183.7 | 171.75 | 185.3 |
| 20 | 175.0 | 167.5 | 179.7 | 165.1 | 182.7 | 171.90 | 185.0 |
| 30 | 177.5 | 168.0 | 180.0 | 165.8 | 182.9 | 173.82 | 186.2 |
| 40 | 181.6 | 170.4 | 182.3 | 168.2 | 184.7 | 177.67 | 189.4 |
| 50 | 187.5 | 174.3 | 185.9 | 171.9 | 188.2 | 183.46 | 194.1 |
| 60 | 190.7 | 179.3 | 190.5 | 176.9 | 192.7 | 190.42 | 199.9 |
| 70 | 193.2 | 184.3 | 195.6 | 182.2 | 197.4 | 197.78 | 205.4 |
| 80 | 197.2 | 188.5 | 199.6 | 186.3 | 201.2 | 203.81 | 209.8 |
| 90 | 198.6 | 190.2 | 201.3 | 187.9 | 202.8 | 206.26 | 211.8 |
| 100 | 196.8 | 188.7 | 199.8 | 186.3 | 201.2 | 204.00 | 210.1 |
| 110 | 192.6 | 184.7 | 196.0 | 182.2 | 197.4 | 197.75 | 205.7 |
| 120 | 187.3 | 179.5 | 190.6 | 176.9 | 192.7 | 190.35 | 200.4 |
| 130 | 181.9 | 174.6 | 186.2 | 171.9 | 188.2 | 183.33 | 194.7 |
| 140 | 177.5 | 170.9 | 182.6 | 168.2 | 184.7 | 177.60 | 189.8 |
| 150 | 174.4 | 168.6 | 180.7 | 165.8 | 182.9 | 173.76 | 186.7 |
| 160 | 172.9 | 168.1 | 180.2 | 165.1 | 182.7 | 171.88 | 185.9 |
| 170 | 172.8 | 168.9 | 180.9 | 165.8 | 183.7 | 171.76 | 185.5 |
| 180 | 173.2 | 169.4 | 181.4 | 166.8 | 184.4 | 172.08 | 185.7 |

**Table S21:** Scanning data for the $T_1$ states around the $\theta_C$ angle (continuation). Calculated energies in kJ/mol:

| $\theta_C$ [deg.] | **2,6-NdMP-4-DMA** |
|---|---|
| 0 | 169.8 |
| 5 | 169.4 |
| 10 | 168.9 |
| 15 | 168.0 |
| 20 | 167.6 |
| 25 | 167.7 |
| 30 | 168.2 |
| 35 | 169.1 |
| 40 | 170.4 |
| 45 | 172.2 |
| 50 | 174.3 |
| 55 | 176.8 |
| 60 | 179.3 |
| 65 | 181.9 |
| 70 | 184.4 |
| 75 | 186.7 |
| 80 | 188.5 |
| 85 | 189.7 |
| 90 | 190.0 |
| 95 | 189.7 |
| 100 | 188.5 |
| 105 | 186.6 |
| 110 | 184.4 |
| 115 | 181.9 |
| 120 | 179.3 |
| 125 | 176.8 |
| 130 | 174.4 |
| 135 | 172.4 |
| 140 | 170.8 |
| 145 | 169.5 |
| 150 | 168.6 |
| 155 | 168.3 |
| 160 | 168.3 |
| 165 | 168.6 |
| 170 | 169.1 |
| 175 | 169.5 |
| 180 | 169.6 |

| $\theta_C$ [deg.] | **2,6-NMP-4-2MeOPh** |
|---|---|
| 0 | 175.5 |
| 18 | 170.6 |
| 36 | 169.5 |
| 54 | 173.8 |
| 72 | 181.5 |
| 90 | 185.9 |
| 108 | 181.5 |
| 126 | 173.8 |
| 144 | 169.5 |
| 162 | 170.6 |
| 180 | 175.5 |

**Table S22:** Energies, oscillator strengths, and calculated absorption maxima for the scanning of **2,4,6-DMA** around the $\theta_B$ and $\theta_C$ torsional angles in the $S_1$ state:

| $\theta_B$ [degree] | ΔE [kJ/mol] | Oscillator strength [-] | $\lambda_{calc}$ [nm] |
|---|---|---|---|
| 0 | 204.2 | 0.759 | 603 |
| 10 | 204.0 | 0.750 | 602 |
| 20 | 204.9 | 0.726 | 602 |
| 30 | 207.1 | 0.673 | 605 |
| 40 | 210.7 | 0.580 | 610 |
| 50 | 214.9 | 0.442 | 620 |
| 60 | 218.9 | 0.273 | 640 |
| 70 | 221.4 | 0.122 | 667 |
| 80 | 222.3 | 0.025 | 692 |
| 90 | 222.3 | 0.001 | 700 |

| $\theta_C$ [degree] | ΔE [kJ/mol] | Oscillator strength [-] | $\lambda_{calc}$ [nm] |
|---|---|---|---|
| 0 | 204.7 | 0.735 | 604 |
| 10 | 204.2 | 0.743 | 603 |
| 20 | 204.1 | 0.762 | 602 |
| 30 | 205.5 | 0.787 | 600 |
| 40 | 208.8 | 0.814 | 600 |
| 50 | 214.0 | 0.846 | 601 |
| 60 | 216.6 | 0.879 | 604 |
| 70 | 213.4 | 0.147 | 715 |
| 80 | 210.3 | 0.032 | 752 |
| 90 | 209.4 | 0.000 | 760 |

**Table S23:** Energies, oscillator strengths, and calculated absorption maxima for the scanning of **2,4,6-DMA** around the $\theta_B$' and $\theta_C$' torsional angles in the $S_1$ state:

| $\theta_B'$ [degree] | ΔE [kJ/mol] | Oscillator strength [-] | $\lambda_{calc}$ [nm] |
|---|---|---|---|
| 0 | 204.7 | 0.757 | 603 |
| 10 | 205.4 | 0.762 | 604 |
| 20 | 206.9 | 0.748 | 603 |
| 30 | 210.5 | 0.752 | 604 |
| 40 | 213.7 | 0.739 | 602 |
| 50 | 216.9 | 0.721 | 605 |
| 60 | 220.7 | 0.718 | 609 |
| 70 | 225.0 | 0.720 | 614 |
| 80 | 229.5 | 0.727 | 618 |
| 90 | 233.7 | 0.728 | 623 |
| 100 | 236.9 | 0.734 | 626 |
| 110 | 238.7 | 0.732 | 629 |
| 120 | 238.9 | 0.728 | 630 |
| 130 | 237.1 | 0.718 | 627 |
| 140 | 212.8 | 0.722 | 603 |
| 150 | 210.0 | 0.756 | 604 |

| $\theta_C'$ [degree] | ΔE [kJ/mol] | Oscillator strength [-] | $\lambda_{calc}$ [nm] |
|---|---|---|---|
| 0 | 204.7 | 0.757 | 603 |
| 10 | 204.7 | 0.757 | 603 |
| 20 | 205.1 | 0.757 | 606 |
| 30 | 206.6 | 0.758 | 610 |
| 40 | 208.8 | 0.757 | 614 |
| 50 | 210.6 | 0.756 | 618 |
| 60 | 212.7 | 0.758 | 624 |
| 70 | 215.8 | 0.761 | 629 |
| 80 | 219.5 | 0.765 | 634 |
| 90 | 223.4 | 0.767 | 639 |
| 100 | 226.9 | 0.705 | 644 |
| 110 | 229.2 | 0.773 | 647 |
| 120 | 230.2 | 0.775 | 649 |
| 130 | 229.6 | 0.774 | 648 |
| 140 | 227.1 | 0.780 | 643 |
| 150 | 215.5 | 0.751 | 608 |

**Table S24:** Scanning data of **2,4,6-DMA** in the $S_0$, $S_1$, and $T_1$ states around the $\theta_B$ angle, using different PCM solvent models:

- in vacuum

| $\theta_B$ [degree] | ΔE in $S_0$ [kJ/mol] | ΔE in $S_1$ [kJ/mol] | ΔE in $T_1$ [kJ/mol] |
|---|---|---|---|
| 88.7 | 32.49 | 209.08 | 192.97 |
| 98.7 | 30.80 | 207.21 | 194.30 |
| 108.7 | 25.78 | 203.09 | 197.80 |
| 118.7 | 19.20 | 197.66 | 201.92 |
| 128.7 | 12.50 | 191.94 | 205.93 |
| 138.7 | 6.75 | 186.84 | 208.88 |
| 148.7 | 2.62 | 183.06 | 211.27 |
| 158.7 | 0.42 | 180.96 | 213.66 |
| 168.7 | 0.00 | 180.46 | 216.07 |
| 178.7 | 0.71 | 180.90 | 217.86 |

- in toluene

| $\theta_B$ [degree] | ΔE in $S_0$ [kJ/mol] | ΔE in $S_1$ [kJ/mol] | ΔE in $T_1$ [kJ/mol] |
|---|---|---|---|
| 88.7 | 31.84 | 207.79 | 207.96 |
| 98.7 | 30.18 | 206.14 | 208.75 |
| 108.7 | 25.45 | 202.13 | 210.56 |
| 118.7 | 19.10 | 196.74 | 211.66 |
| 128.7 | 12.53 | 190.99 | 212.16 |
| 138.7 | 6.78 | 185.75 | 211.81 |
| 148.7 | 2.68 | 181.88 | 211.36 |
| 158.7 | 0.48 | 179.52 | 211.38 |
| 168.7 | 0.00 | 178.76 | 211.43 |
| 178.7 | 0.63 | 178.99 | 211.26 |

- in THF

| $\theta_B$ [degree] | $\Delta E$ in $S_0$ [kJ/mol] | $\Delta E$ in $S_1$ [kJ/mol] | $\Delta E$ in $T_1$ [kJ/mol] |
|---|---|---|---|
| 88.7 | 31.47 | 205.04 | 217.30 |
| 98.7 | 29.92 | 203.84 | 217.61 |
| 108.7 | 25.33 | 200.15 | 217.74 |
| 118.7 | 19.10 | 195.05 | 216.76 |
| 128.7 | 12.63 | 189.46 | 214.43 |
| 138.7 | 6.91 | 184.17 | 211.55 |
| 148.7 | 2.79 | 180.11 | 208.98 |
| 158.7 | 0.56 | 177.48 | 207.35 |
| 168.7 | 0.00 | 176.28 | 206.76 |
| 178.7 | 0.57 | 176.17 | 207.02 |

- in water

| $\theta_B$ [degree] | $\Delta E$ in $S_0$ [kJ/mol] | $\Delta E$ in $S_1$ [kJ/mol] | $\Delta E$ in $T_1$ [kJ/mol] |
|---|---|---|---|
| 88.7 | 31.34 | 203.37 | 222.28 |
| 98.7 | 29.83 | 202.22 | 222.29 |
| 108.7 | 25.30 | 200.62 | 221.42 |
| 118.7 | 19.14 | 198.58 | 218.94 |
| 128.7 | 12.70 | 193.59 | 214.93 |
| 138.7 | 7.01 | 188.01 | 210.65 |
| 148.7 | 2.88 | 182.68 | 207.08 |
| 158.7 | 0.61 | 178.49 | 204.91 |
| 168.7 | 0.00 | 175.78 | 204.03 |
| 178.7 | 0.50 | 174.46 | 204.18 |

**Table S25:** Scanning data of **2,4,6-DMA** in the $S_0$, $S_1$, and $T_1$ states around the $\theta_C$ angle, using different PCM solvent models:

- in vacuum

| $\theta_C$ [degree] | ΔE in $S_0$ [kJ/mol] | ΔE in $S_1$ [kJ/mol] | ΔE in $T_1$ [kJ/mol] |
|---|---|---|---|
| 88.7 | 34.66 | 176.88 | 200.29 |
| 98.7 | 31.52 | 179.02 | 199.52 |
| 108.7 | 24.46 | 185.84 | 195.89 |
| 118.7 | 16.60 | 196.80 | 191.07 |
| 128.7 | 9.62 | 216.40 | 186.37 |
| 138.7 | 4.45 | 200.60 | 182.76 |
| 148.7 | 1.53 | 194.58 | 180.75 |
| 158.7 | 0.88 | 193.12 | 180.60 |
| 168.7 | 1.72 | 193.09 | 181.78 |
| 178.7 | 2.20 | 193.43 | 182.83 |

- in toluene

| $\theta_C$ [degree] | ΔE in $S_0$ [kJ/mol] | ΔE in $S_1$ [kJ/mol] | ΔE in $T_1$ [kJ/mol] |
|---|---|---|---|
| 88.7 | 33.14 | 193.40 | 200.05 |
| 98.7 | 30.38 | 195.64 | 197.91 |
| 108.7 | 23.96 | 201.53 | 193.78 |
| 118.7 | 16.35 | 209.60 | 188.99 |
| 128.7 | 9.51 | 222.13 | 184.45 |
| 138.7 | 4.44 | 216.81 | 180.99 |
| 148.7 | 1.51 | 213.24 | 178.97 |
| 158.7 | 0.89 | 211.56 | 178.83 |
| 168.7 | 1.60 | 211.33 | 179.76 |
| 178.7 | 2.16 | 211.65 | 180.78 |

- in THF

| $\theta_C$ [degree] | ΔE in $S_0$ [kJ/mol] | ΔE in $S_1$ [kJ/mol] | ΔE in $T_1$ [kJ/mol] |
|---|---|---|---|
| 88.7 | 32.65 | 203.85 | 198.46 |
| 98.7 | 30.20 | 205.67 | 196.82 |
| 108.7 | 24.11 | 210.15 | 192.66 |
| 118.7 | 16.73 | 214.28 | 187.47 |
| 128.7 | 9.95 | 218.33 | 182.68 |
| 138.7 | 4.79 | 212.32 | 178.90 |
| 148.7 | 1.72 | 208.87 | 176.51 |
| 158.7 | 0.95 | 207.41 | 176.09 |
| 168.7 | 1.49 | 207.25 | 176.80 |
| 178.7 | 1.95 | 207.46 | 177.68 |

- in water

| $\theta_C$ [degree] | ΔE in $S_0$ [kJ/mol] | ΔE in $S_1$ [kJ/mol] | ΔE in $T_1$ [kJ/mol] |
|---|---|---|---|
| 88.7 | 32.53 | 209.41 | 197.38 |
| 98.7 | 30.30 | 210.91 | 195.61 |
| 108.7 | 24.26 | 214.32 | 191.34 |
| 118.7 | 16.99 | 216.62 | 186.09 |
| 128.7 | 10.23 | 219.80 | 181.11 |
| 138.7 | 5.00 | 212.89 | 177.18 |
| 148.7 | 1.89 | 205.52 | 174.84 |
| 158.7 | 0.99 | 204.12 | 174.27 |
| 168.7 | 1.45 | 204.18 | 176.68 |
| 178.7 | 1.79 | 204.74 | 175.79 |

**Table S26:** Scanning data of 4-(4-dimethylaminophenyl)-2,6-diphenylpyrylium in the $S_0$, $S_1$, and $T_1$ states around the $\theta_B$ and $\theta_B$ angles, using PCM solvent model with ethanol parameter set:

| $\theta_B$ [deg.] | ΔE in $S_0$ [kJ/mol] | ΔE in $S_1$ [kJ/mol] | ΔE in $T_1$ [kJ/mol] |
|---|---|---|---|
| 0 | 1.41 | 172.97 | 162.36 |
| 10 | 0.55 | 172.56 | 162.84 |
| 20 | 0.00 | 172.66 | 162.00 |
| 30 | 0.50 | 173.83 | 162.47 |
| 40 | 2.61 | 176.32 | 164.90 |
| 50 | 6.96 | 180.06 | 168.66 |
| 60 | 11.21 | 184.71 | 173.39 |
| 70 | 15.81 | 189.47 | 178.35 |
| 80 | 19.46 | 193.20 | 182.27 |
| 90 | 20.76 | 194.53 | 183.69 |
| 100 | 19.18 | 193.08 | 182.09 |
| 110 | 15.49 | 189.50 | 178.31 |
| 120 | 10.78 | 184.93 | 173.51 |
| 130 | 6.21 | 180.29 | 168.74 |
| 140 | 2.61 | 176.42 | 165.41 |
| 150 | 0.50 | 173.87 | 162.47 |
| 160 | 0.00 | 172.67 | 162.00 |
| 170 | 0.55 | 172.57 | 162.84 |
| 180 | 1.41 | 172.97 | 162.36 |

| $\theta_B$ [deg.] | ΔE in $S_0$ [kJ/mol] | ΔE in $S_1$ [kJ/mol] | ΔE in $T_1$ [kJ/mol] |
|---|---|---|---|
| 0 | 0.74 | 225.34 | 163.61 |
| 10 | 0.06 | 222.78 | 162.57 |
| 20 | 0.23 | 218.14 | 161.25 |
| 30 | 2.18 | 213.17 | 161.41 |
| 40 | 6.65 | 208.19 | 163.39 |
| 50 | 13.71 | 201.64 | 166.94 |
| 60 | 22.61 | 192.09 | 171.61 |
| 70 | 32.34 | 181.81 | 176.37 |
| 80 | 41.19 | 174.82 | 180.07 |
| 90 | 45.50 | 172.50 | 181.53 |
| 100 | 41.54 | 174.80 | 180.13 |
| 110 | 32.89 | 181.23 | 176.33 |
| 120 | 23.22 | 190.65 | 171.62 |
| 130 | 14.47 | 200.34 | 167.11 |
| 140 | 7.58 | 207.57 | 163.86 |
| 150 | 3.15 | 212.96 | 162.05 |
| 160 | 1.12 | 218.20 | 161.99 |
| 170 | 0.69 | 223.01 | 163.03 |
| 180 | 0.75 | 225.34 | 163.62 |

### 6.4. 2D scanning data

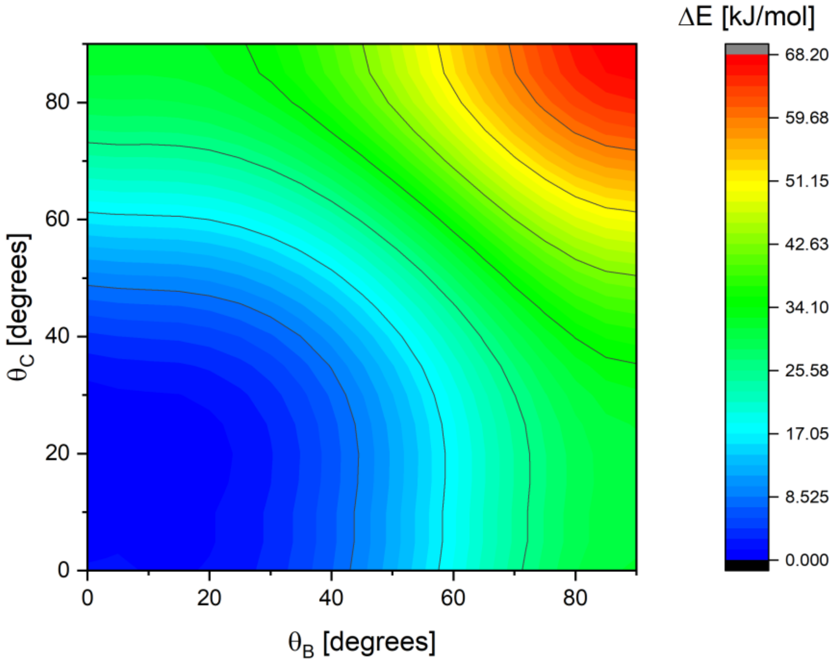


*Figure S174. Scanning data calculated for the simultaneous rotation of the B and C rings in $S_0$*

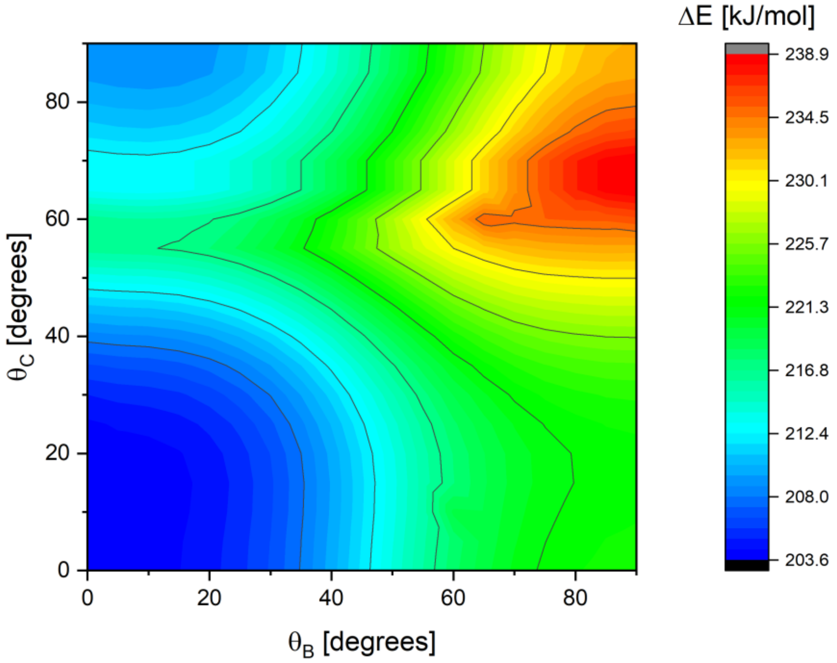


*Figure S175. Scanning data calculated for the simultaneous rotation of the B and C rings in $S_1$*

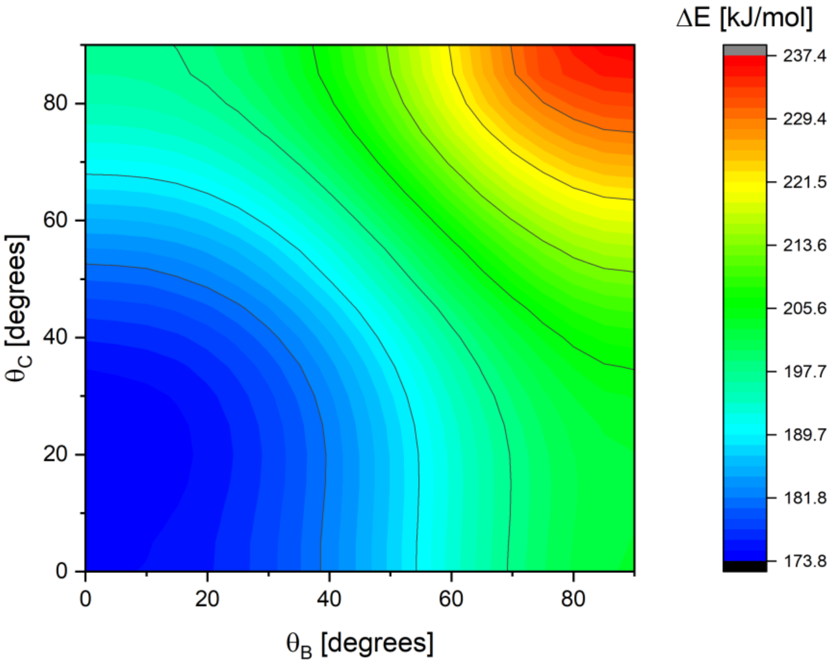


*Figure S176. Scanning data calculated for the simultaneous rotation of the B and C rings in $T_1$*

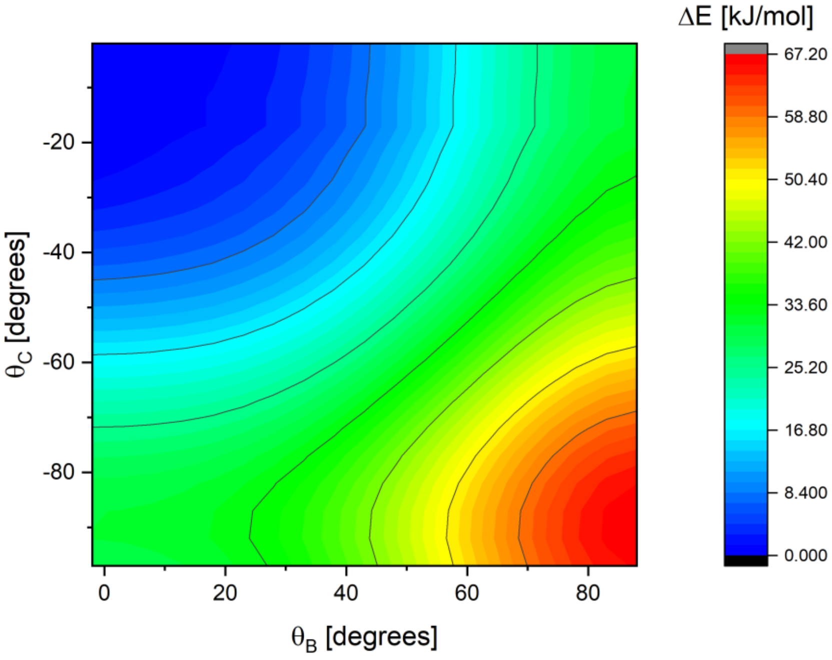


*Figure S177. Scanning data calculated for the simultaneous rotation of the B and D rings in $S_0$*

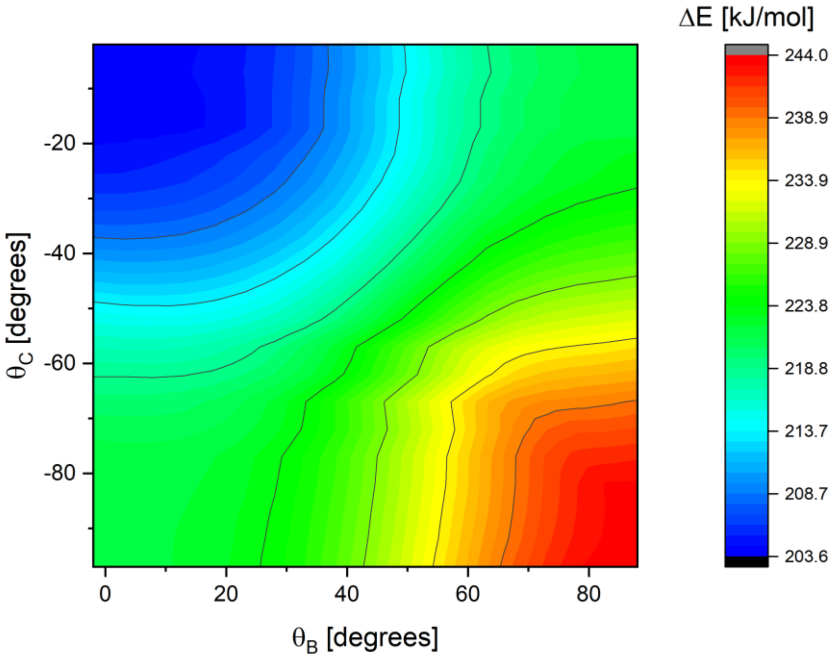


*Figure S178. Scanning data calculated for the simultaneous rotation of the B and D rings in $S_1$*

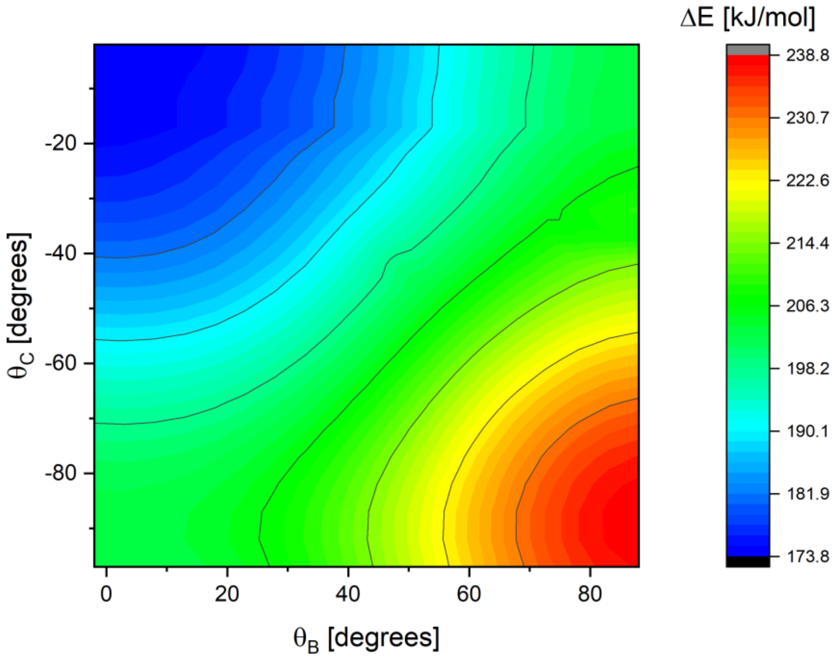


*Figure S179. Scanning data calculated for the simultaneous rotation of the B and D rings in $T_1$*

## 6.5. Coordinates of the optimized structures

### 2,4,6-DMA (vacuo)

**2,4,6-DMA_vacuo_S0**

1011aaa_tris_NMe2_pirrilium_b3lyp631dp.log

Standard orientation:

| Center Number | Atomic Number | Atomic Type | Coordinates (Angstroms) X | Y | Z |
|---|---|---|---|---|---|
| 1 | 6 | 0 | -0.919667 | 1.203954 | 0.012423 |
| 2 | 6 | 0 | 0.459349 | 1.194265 | 0.000694 |
| 3 | 6 | 0 | 0.459383 | -1.194275 | -0.000823 |
| 4 | 6 | 0 | -0.919639 | -1.204003 | -0.012466 |
| 5 | 6 | 0 | -1.662391 | -0.000041 | -0.000022 |
| 6 | 1 | 0 | -1.424057 | 2.155743 | 0.091929 |
| 7 | 1 | 0 | -1.423962 | -2.155836 | -0.091909 |
| 8 | 6 | 0 | -3.116712 | -0.000052 | -0.000028 |
| 9 | 6 | 0 | -3.860467 | -1.121863 | 0.432579 |
| 10 | 6 | 0 | -3.860492 | 1.121740 | -0.432633 |
| 11 | 6 | 0 | -5.240135 | -1.128991 | 0.443594 |
| 12 | 1 | 0 | -3.345515 | -1.998169 | 0.812385 |
| 13 | 6 | 0 | -5.240161 | 1.128830 | -0.443650 |
| 14 | 1 | 0 | -3.345568 | 1.998070 | -0.812421 |
| 15 | 6 | 0 | -5.983151 | -0.000095 | -0.000044 |
| 16 | 1 | 0 | -5.752551 | -2.008875 | 0.809804 |
| 17 | 1 | 0 | -5.752600 | 2.008716 | -0.809826 |
| 18 | 6 | 0 | 1.340954 | 2.340414 | 0.020788 |
| 19 | 6 | 0 | 2.713616 | 2.206959 | 0.325470 |
| 20 | 6 | 0 | 0.864355 | 3.639939 | -0.260715 |
| 21 | 6 | 0 | 3.557727 | 3.298131 | 0.358825 |
| 22 | 1 | 0 | 3.114594 | 1.227993 | 0.561267 |
| 23 | 6 | 0 | 1.696854 | 4.739224 | -0.235516 |
| 24 | 1 | 0 | -0.174840 | 3.792583 | -0.532644 |
| 25 | 6 | 0 | 3.078266 | 4.607743 | 0.079652 |
| 26 | 1 | 0 | 4.598085 | 3.141615 | 0.611746 |
| 27 | 1 | 0 | 1.281889 | 5.709891 | -0.472571 |
| 28 | 8 | 0 | 1.114198 | 0.000013 | -0.000123 |
| 29 | 7 | 0 | -7.348054 | -0.000118 | -0.000077 |
| 30 | 7 | 0 | 3.907049 | 5.692153 | 0.110815 |
| 31 | 6 | 0 | 3.386132 | 7.027229 | -0.164014 |
| 32 | 1 | 0 | 4.193237 | 7.752022 | -0.067597 |
| 33 | 1 | 0 | 2.982548 | 7.102641 | -1.181129 |
| 34 | 1 | 0 | 2.596311 | 7.304278 | 0.544275 |
| 35 | 6 | 0 | 5.324017 | 5.528458 | 0.418310 |
| 36 | 1 | 0 | 5.812724 | 6.500843 | 0.377385 |
| 37 | 1 | 0 | 5.474287 | 5.115425 | 1.423183 |
| 38 | 1 | 0 | 5.819320 | 4.869921 | -0.305171 |
| 39 | 6 | 0 | -8.085802 | -1.180420 | 0.438398 |
| 40 | 1 | 0 | -7.880268 | -1.418588 | 1.489161 |
| 41 | 1 | 0 | -9.153634 | -0.991291 | 0.338514 |
| 42 | 1 | 0 | -7.838979 | -2.057694 | -0.171141 |
| 43 | 6 | 0 | -8.085816 | 1.180268 | -0.438306 |
| 44 | 1 | 0 | -7.880086 | 1.418766 | -1.488951 |
| 45 | 1 | 0 | -9.153651 | 0.991006 | -0.338718 |
| 46 | 1 | 0 | -7.839193 | 2.057383 | 0.171549 |
| 47 | 6 | 0 | 1.341028 | -2.340381 | -0.020873 |
| 48 | 6 | 0 | 2.713669 | -2.206907 | -0.325669 |
| 49 | 6 | 0 | 0.864486 | -3.639899 | 0.260770 |
| 50 | 6 | 0 | 3.557807 | -3.298050 | -0.358971 |
| 51 | 1 | 0 | 3.114579 | -1.227941 | -0.561593 |
| 52 | 6 | 0 | 1.697013 | -4.739161 | 0.235628 |
| 53 | 1 | 0 | -0.174698 | -3.792530 | 0.532754 |
| 54 | 6 | 0 | 3.078413 | -4.607656 | -0.079595 |
| 55 | 1 | 0 | 4.598147 | -3.141543 | -0.611974 |
| 56 | 1 | 0 | 1.282076 | -5.709838 | 0.472700 |
| 57 | 7 | 0 | 3.907228 | -5.692025 | -0.110673 |
| 58 | 6 | 0 | 3.386467 | -7.027039 | 0.164894 |
| 59 | 1 | 0 | 2.596981 | -7.304766 | -0.543507 |
| 60 | 1 | 0 | 4.193777 | -7.751713 | 0.069353 |
| 61 | 1 | 0 | 2.982512 | -7.101741 | 1.181896 |
| 62 | 6 | 0 | 5.324076 | -5.528398 | -0.418822 |
| 63 | 1 | 0 | 5.819770 | -4.869978 | 0.304494 |

| | | | | | |
|---|---|---|---|---|---|
| 64 | 1 | 0 | 5.812708 | -6.500832 | -0.378266 |
| 65 | 1 | 0 | 5.473858 | -5.115272 | -1.423719 |

---------------------------------------------------------------------

**2,4,6-DMA_VACUO_S1**
1011aab_tris_NMe2_pirrilium_TD_b3lyp631dp_optsp.log

Standard orientation:

| Center Number | Atomic Number | Atomic Type | Coordinates (Angstroms) X | Y | Z |
|---|---|---|---|---|---|
| 1 | 6 | 0 | 0.835884 | 1.263069 | 0.004579 |
| 2 | 6 | 0 | -0.503061 | 1.152770 | 0.000961 |
| 3 | 6 | 0 | -0.416355 | -1.216668 | -0.011725 |
| 4 | 6 | 0 | 0.954572 | -1.134640 | -0.007780 |
| 5 | 6 | 0 | 1.677476 | 0.083812 | -0.004270 |
| 6 | 1 | 0 | 1.267415 | 2.256363 | -0.014266 |
| 7 | 1 | 0 | 1.494704 | -2.070921 | 0.034120 |
| 8 | 6 | 0 | 3.121628 | 0.160320 | -0.007249 |
| 9 | 6 | 0 | 3.934420 | -0.957475 | -0.321708 |
| 10 | 6 | 0 | 3.820264 | 1.351196 | 0.308077 |
| 11 | 6 | 0 | 5.315768 | -0.897786 | -0.319758 |
| 12 | 1 | 0 | 3.472148 | -1.897857 | -0.603156 |
| 13 | 6 | 0 | 5.202514 | 1.426091 | 0.314026 |
| 14 | 1 | 0 | 3.270329 | 2.242411 | 0.593193 |
| 15 | 6 | 0 | 6.002541 | 0.301707 | -0.005688 |
| 16 | 1 | 0 | 5.870305 | -1.791534 | -0.576842 |
| 17 | 1 | 0 | 5.667294 | 2.366377 | 0.582930 |
| 18 | 6 | 0 | -1.465879 | 2.304454 | -0.014326 |
| 19 | 6 | 0 | -1.900621 | 2.857352 | -1.231610 |
| 20 | 6 | 0 | -1.963982 | 2.837847 | 1.187389 |
| 21 | 6 | 0 | -2.787442 | 3.917704 | -1.262000 |
| 22 | 1 | 0 | -1.533139 | 2.441376 | -2.162134 |
| 23 | 6 | 0 | -2.861538 | 3.889487 | 1.187852 |
| 24 | 1 | 0 | -1.629123 | 2.421099 | 2.129829 |
| 25 | 6 | 0 | -3.292515 | 4.457644 | -0.044942 |
| 26 | 1 | 0 | -3.118582 | 4.306077 | -2.216006 |
| 27 | 1 | 0 | -3.201842 | 4.297055 | 2.130582 |
| 28 | 8 | 0 | -1.180617 | -0.040300 | -0.007023 |
| 29 | 7 | 0 | 7.380569 | 0.370774 | -0.017726 |
| 30 | 7 | 0 | -4.177334 | 5.504954 | -0.059719 |
| 31 | 6 | 0 | -4.904097 | 5.922738 | 1.141056 |
| 32 | 1 | 0 | -5.821896 | 6.423834 | 0.835101 |
| 33 | 1 | 0 | -5.162027 | 5.061908 | 1.755474 |
| 34 | 1 | 0 | -4.300016 | 6.625681 | 1.726237 |
| 35 | 6 | 0 | -4.452530 | 6.269727 | -1.277920 |
| 36 | 1 | 0 | -4.803156 | 7.261443 | -0.994043 |
| 37 | 1 | 0 | -3.551488 | 6.374694 | -1.879839 |
| 38 | 1 | 0 | -5.234416 | 5.777604 | -1.867757 |
| 39 | 6 | 0 | 8.166344 | -0.833879 | -0.232160 |
| 40 | 1 | 0 | 7.950037 | -1.284043 | -1.208544 |
| 41 | 1 | 0 | 9.225812 | -0.578440 | -0.213712 |
| 42 | 1 | 0 | 7.985103 | -1.594093 | 0.540861 |
| 43 | 6 | 0 | 8.050054 | 1.590333 | 0.402631 |
| 44 | 1 | 0 | 7.841091 | 1.843556 | 1.452116 |
| 45 | 1 | 0 | 9.127203 | 1.464079 | 0.293253 |
| 46 | 1 | 0 | 7.750935 | 2.443280 | -0.218415 |
| 47 | 6 | 0 | -1.234670 | -2.414078 | -0.001673 |
| 48 | 6 | 0 | -2.634112 | -2.350781 | 0.158969 |
| 49 | 6 | 0 | -0.674334 | -3.701125 | -0.155254 |
| 50 | 6 | 0 | -3.422662 | -3.490569 | 0.180671 |
| 51 | 1 | 0 | -3.111168 | -1.385004 | 0.273688 |
| 52 | 6 | 0 | -1.449535 | -4.845603 | -0.132992 |
| 53 | 1 | 0 | 0.393302 | -3.815939 | -0.310773 |
| 54 | 6 | 0 | -2.855400 | -4.780295 | 0.043652 |
| 55 | 1 | 0 | -4.491785 | -3.376940 | 0.308428 |
| 56 | 1 | 0 | -0.959930 | -5.802311 | -0.262669 |
| 57 | 7 | 0 | -3.629313 | -5.919601 | 0.078255 |
| 58 | 6 | 0 | -3.018931 | -7.223896 | -0.125471 |
| 59 | 1 | 0 | -2.249701 | -7.430329 | 0.628946 |
| 60 | 1 | 0 | -3.784164 | -7.995070 | -0.038278 |
| 61 | 1 | 0 | -2.556142 | -7.315818 | -1.117862 |

| | | | | | |
|---|---|---|---|---|---|
| 62 | 6 | 0 | -5.074515 | -5.814461 | 0.190089 |
| 63 | 1 | 0 | -5.517884 | -5.273090 | -0.657396 |
| 64 | 1 | 0 | -5.506462 | -6.814842 | 0.213332 |
| 65 | 1 | 0 | -5.370981 | -5.301062 | 1.113587 |

---------------------------------------------------------------------

**2,4,6-DMA_VACUO_TR**

1011aac_tris_NMe2_pirrilium_b3lyp631dp_TRIPLET.log

Standard orientation:

| Center Number | Atomic Number | Atomic Type | Coordinates (Angstroms) X | Y | Z |
|---|---|---|---|---|---|
| 1 | 6 | 0 | 0.924238 | 1.161842 | 0.053376 |
| 2 | 6 | 0 | -0.462538 | 1.199263 | 0.028048 |
| 3 | 6 | 0 | -0.510174 | -1.196998 | -0.006666 |
| 4 | 6 | 0 | 0.856960 | -1.232307 | 0.014015 |
| 5 | 6 | 0 | 1.652549 | -0.044788 | 0.039931 |
| 6 | 1 | 0 | 1.454418 | 2.105167 | 0.038183 |
| 7 | 1 | 0 | 1.338223 | -2.199894 | 0.065747 |
| 8 | 6 | 0 | 3.112357 | -0.065881 | 0.027282 |
| 9 | 6 | 0 | 3.833042 | -1.162246 | -0.497384 |
| 10 | 6 | 0 | 3.882845 | 1.001795 | 0.542457 |
| 11 | 6 | 0 | 5.215869 | -1.198233 | -0.509552 |
| 12 | 1 | 0 | 3.296533 | -1.996162 | -0.938659 |
| 13 | 6 | 0 | 5.265604 | 0.984081 | 0.536873 |
| 14 | 1 | 0 | 3.387307 | 1.855293 | 0.994627 |
| 15 | 6 | 0 | 5.981820 | -0.122213 | 0.010357 |
| 16 | 1 | 0 | 5.708343 | -2.060531 | -0.940635 |
| 17 | 1 | 0 | 5.796868 | 1.827454 | 0.959138 |
| 18 | 6 | 0 | -1.281248 | 2.377928 | -0.006362 |
| 19 | 6 | 0 | -2.692228 | 2.280853 | -0.150313 |
| 20 | 6 | 0 | -0.737547 | 3.688767 | 0.099507 |
| 21 | 6 | 0 | -3.496922 | 3.398304 | -0.190689 |
| 22 | 1 | 0 | -3.143352 | 1.301898 | -0.247313 |
| 23 | 6 | 0 | -1.532593 | 4.809840 | 0.062735 |
| 24 | 1 | 0 | 0.330129 | 3.827959 | 0.224268 |
| 25 | 6 | 0 | -2.947402 | 4.707252 | -0.084842 |
| 26 | 1 | 0 | -4.564369 | 3.264311 | -0.309988 |
| 27 | 1 | 0 | -1.063353 | 5.780891 | 0.152521 |
| 28 | 8 | 0 | -1.181929 | 0.016890 | -0.000336 |
| 29 | 7 | 0 | 7.356480 | -0.149825 | 0.001102 |
| 30 | 7 | 0 | -3.741119 | 5.820426 | -0.123049 |
| 31 | 6 | 0 | -3.154468 | 7.154134 | -0.019350 |
| 32 | 1 | 0 | -3.946727 | 7.898467 | -0.076911 |
| 33 | 1 | 0 | -2.630130 | 7.285359 | 0.934086 |
| 34 | 1 | 0 | -2.447876 | 7.342405 | -0.835604 |
| 35 | 6 | 0 | -5.188560 | 5.693581 | -0.272137 |
| 36 | 1 | 0 | -5.636059 | 6.685934 | -0.273488 |
| 37 | 1 | 0 | -5.449682 | 5.198521 | -1.214474 |
| 38 | 1 | 0 | -5.624626 | 5.122337 | 0.555184 |
| 39 | 6 | 0 | 8.065597 | -1.303866 | -0.534864 |
| 40 | 1 | 0 | 7.845081 | -1.456118 | -1.598986 |
| 41 | 1 | 0 | 9.138173 | -1.145026 | -0.431655 |
| 42 | 1 | 0 | 7.804831 | -2.222093 | 0.005453 |
| 43 | 6 | 0 | 8.117136 | 0.978586 | 0.520164 |
| 44 | 1 | 0 | 7.913428 | 1.147287 | 1.585069 |
| 45 | 1 | 0 | 9.181356 | 0.775740 | 0.407998 |
| 46 | 1 | 0 | 7.887915 | 1.902901 | -0.024473 |
| 47 | 6 | 0 | -1.408699 | -2.335401 | 0.006955 |
| 48 | 6 | 0 | -2.784313 | -2.178133 | 0.290133 |
| 49 | 6 | 0 | -0.953764 | -3.648278 | -0.259523 |
| 50 | 6 | 0 | -3.648826 | -3.255716 | 0.321444 |
| 51 | 1 | 0 | -3.169211 | -1.189867 | 0.508833 |
| 52 | 6 | 0 | -1.804845 | -4.732790 | -0.232247 |
| 53 | 1 | 0 | 0.085815 | -3.820739 | -0.516131 |
| 54 | 6 | 0 | -3.187938 | -4.574112 | 0.063191 |
| 55 | 1 | 0 | -4.690892 | -3.078251 | 0.553987 |
| 56 | 1 | 0 | -1.403131 | -5.713034 | -0.453398 |
| 57 | 7 | 0 | -4.037664 | -5.648481 | 0.092372 |
| 58 | 6 | 0 | -3.537381 | -6.993861 | -0.167606 |
| 59 | 1 | 0 | -2.762315 | -7.279082 | 0.553485 |

| | | | | | |
|---|---|---|---|---|---|
| 60 | 1 | 0 | -4.358399 | -7.703783 | -0.079866 |
| 61 | 1 | 0 | -3.119487 | -7.079427 | -1.177966 |
| 62 | 6 | 0 | -5.454203 | -5.459948 | 0.383646 |
| 63 | 1 | 0 | -5.930148 | -4.800965 | -0.352321 |
| 64 | 1 | 0 | -5.958074 | -6.424694 | 0.349925 |
| 65 | 1 | 0 | -5.605236 | -5.030946 | 1.381593 |

---------------------------------------------------------------------

## 2,4,6-DMA (toluene)

### 2,4,6-DMA_TOL_S0

1011aaa_tris_NMe2_pirrilium_b3lyp631dp_PCMtol.log

Standard orientation:

| Center Number | Atomic Number | Atomic Type | Coordinates (Angstroms) X | Y | Z |
|---|---|---|---|---|---|
| 1 | 6 | 0 | 0.922589 | 1.204527 | -0.006622 |
| 2 | 6 | 0 | -0.456425 | 1.193951 | 0.002794 |
| 3 | 6 | 0 | -0.456415 | -1.193945 | -0.002662 |
| 4 | 6 | 0 | 0.922599 | -1.204510 | 0.006763 |
| 5 | 6 | 0 | 1.663834 | 0.000011 | 0.000077 |
| 6 | 1 | 0 | 1.428008 | 2.156464 | -0.076946 |
| 7 | 1 | 0 | 1.428025 | -2.156444 | 0.077068 |
| 8 | 6 | 0 | 3.118803 | 0.000014 | 0.000060 |
| 9 | 6 | 0 | 3.861884 | -1.115477 | -0.449796 |
| 10 | 6 | 0 | 3.861895 | 1.115509 | 0.449891 |
| 11 | 6 | 0 | 5.242005 | -1.122110 | -0.461220 |
| 12 | 1 | 0 | 3.346688 | -1.986002 | -0.842350 |
| 13 | 6 | 0 | 5.242016 | 1.122153 | 0.461257 |
| 14 | 1 | 0 | 3.346708 | 1.986034 | 0.842458 |
| 15 | 6 | 0 | 5.984928 | 0.000024 | -0.000001 |
| 16 | 1 | 0 | 5.754593 | -1.995988 | -0.841355 |
| 17 | 1 | 0 | 5.754608 | 1.996040 | 0.841362 |
| 18 | 6 | 0 | -1.339541 | 2.339520 | -0.016382 |
| 19 | 6 | 0 | -2.716107 | 2.201341 | -0.301169 |
| 20 | 6 | 0 | -0.860558 | 3.642571 | 0.244843 |
| 21 | 6 | 0 | -3.562443 | 3.291187 | -0.334097 |
| 22 | 1 | 0 | -3.119894 | 1.219686 | -0.520030 |
| 23 | 6 | 0 | -1.694957 | 4.740833 | 0.218622 |
| 24 | 1 | 0 | 0.182322 | 3.800043 | 0.498433 |
| 25 | 6 | 0 | -3.080547 | 4.604518 | -0.076451 |
| 26 | 1 | 0 | -4.606322 | 3.130018 | -0.568897 |
| 27 | 1 | 0 | -1.277504 | 5.714746 | 0.437235 |
| 28 | 8 | 0 | -1.110453 | 0.000000 | 0.000062 |
| 29 | 7 | 0 | 7.349677 | 0.000018 | -0.000059 |
| 30 | 7 | 0 | -3.910891 | 5.687287 | -0.109360 |
| 31 | 6 | 0 | -3.388463 | 7.026208 | 0.143477 |
| 32 | 1 | 0 | -4.200223 | 7.747213 | 0.059544 |
| 33 | 1 | 0 | -2.962706 | 7.110033 | 1.150624 |
| 34 | 1 | 0 | -2.614414 | 7.299935 | -0.583323 |
| 35 | 6 | 0 | -5.329897 | 5.518347 | -0.404546 |
| 36 | 1 | 0 | -5.816888 | 6.491973 | -0.381976 |
| 37 | 1 | 0 | -5.485770 | 5.083154 | -1.398996 |
| 38 | 1 | 0 | -5.821460 | 4.875414 | 0.335217 |
| 39 | 6 | 0 | 8.087179 | -1.174408 | -0.454190 |
| 40 | 1 | 0 | 7.881557 | -1.398058 | -1.508013 |
| 41 | 1 | 0 | 9.154810 | -0.986671 | -0.350506 |
| 42 | 1 | 0 | 7.838649 | -2.060047 | 0.142217 |
| 43 | 6 | 0 | 8.087258 | 1.174422 | 0.454009 |
| 44 | 1 | 0 | 7.881758 | 1.398066 | 1.507856 |
| 45 | 1 | 0 | 9.154873 | 0.986657 | 0.350207 |
| 46 | 1 | 0 | 7.838691 | 2.060074 | -0.142362 |
| 47 | 6 | 0 | -1.339522 | -2.339522 | 0.016467 |
| 48 | 6 | 0 | -2.716095 | -2.201365 | 0.301232 |
| 49 | 6 | 0 | -0.860523 | -3.642560 | -0.244798 |
| 50 | 6 | 0 | -3.562422 | -3.291219 | 0.334106 |
| 51 | 1 | 0 | -3.119895 | -1.219721 | 0.520118 |
| 52 | 6 | 0 | -1.694913 | -4.740830 | -0.218631 |
| 53 | 1 | 0 | 0.182361 | -3.800014 | -0.498381 |
| 54 | 6 | 0 | -3.080511 | -4.604537 | 0.076419 |
| 55 | 1 | 0 | -4.606307 | -3.130067 | 0.568890 |

| | | | | | |
|---|---|---|---|---|---|
| 56 | 1 | 0 | -1.277449 | -5.714731 | -0.437275 |
| 57 | 7 | 0 | -3.910845 | -5.687314 | 0.109268 |
| 58 | 6 | 0 | -3.388399 | -7.026221 | -0.143614 |
| 59 | 1 | 0 | -2.614371 | -7.299975 | 0.583198 |
| 60 | 1 | 0 | -4.200156 | -7.747235 | -0.059740 |
| 61 | 1 | 0 | -2.962610 | -7.109995 | -1.150751 |
| 62 | 6 | 0 | -5.329858 | -5.518402 | 0.404436 |
| 63 | 1 | 0 | -5.821417 | -4.875448 | -0.335312 |
| 64 | 1 | 0 | -5.816838 | -6.492032 | 0.381822 |
| 65 | 1 | 0 | -5.485752 | -5.083247 | 1.398898 |

---------------------------------------------------------------------

**2,4,6-DMA_TOL_S1**

1011aaa_tris_NMe2_pirrilium_TD_b3lyp631dp_PCMtol_spopt.log

Standard orientation:

| Center Number | Atomic Number | Atomic Type | Coordinates (Angstroms) X | Y | Z |
|---|---|---|---|---|---|
| 1 | 6 | 0 | 0.902582 | -1.199426 | -0.018425 |
| 2 | 6 | 0 | -0.465044 | -1.189362 | -0.023252 |
| 3 | 6 | 0 | -0.465042 | 1.189362 | 0.023248 |
| 4 | 6 | 0 | 0.902583 | 1.199424 | 0.018420 |
| 5 | 6 | 0 | 1.682075 | -0.000002 | -0.000003 |
| 6 | 1 | 0 | 1.393044 | -2.162619 | 0.013510 |
| 7 | 1 | 0 | 1.393047 | 2.162616 | -0.013514 |
| 8 | 6 | 0 | 3.125302 | -0.000002 | -0.000002 |
| 9 | 6 | 0 | 3.881669 | 1.165368 | 0.288848 |
| 10 | 6 | 0 | 3.881670 | -1.165372 | -0.288852 |
| 11 | 6 | 0 | 5.263392 | 1.172750 | 0.292083 |
| 12 | 1 | 0 | 3.375564 | 2.089128 | 0.548245 |
| 13 | 6 | 0 | 5.263392 | -1.172755 | -0.292084 |
| 14 | 1 | 0 | 3.375565 | -2.089133 | -0.548250 |
| 15 | 6 | 0 | 6.008416 | -0.000003 | 0.000001 |
| 16 | 1 | 0 | 5.774577 | 2.095929 | 0.534300 |
| 17 | 1 | 0 | 5.774577 | -2.095935 | -0.534301 |
| 18 | 6 | 0 | -1.347283 | -2.352698 | -0.006217 |
| 19 | 6 | 0 | -2.719525 | -2.216628 | 0.300643 |
| 20 | 6 | 0 | -0.871895 | -3.652820 | -0.291061 |
| 21 | 6 | 0 | -3.566640 | -3.308952 | 0.338265 |
| 22 | 1 | 0 | -3.116659 | -1.235482 | 0.529716 |
| 23 | 6 | 0 | -1.704663 | -4.753016 | -0.256086 |
| 24 | 1 | 0 | 0.166387 | -3.805285 | -0.563740 |
| 25 | 6 | 0 | -3.085252 | -4.617851 | 0.064793 |
| 26 | 1 | 0 | -4.608603 | -3.149692 | 0.583140 |
| 27 | 1 | 0 | -1.289887 | -5.725522 | -0.486090 |
| 28 | 8 | 0 | -1.162959 | 0.000000 | -0.000002 |
| 29 | 7 | 0 | 7.381971 | -0.000004 | 0.000002 |
| 30 | 7 | 0 | -3.915384 | -5.706362 | 0.103188 |
| 31 | 6 | 0 | -3.404438 | -7.039812 | -0.196947 |
| 32 | 1 | 0 | -4.220371 | -7.757167 | -0.131679 |
| 33 | 1 | 0 | -2.986064 | -7.087704 | -1.209019 |
| 34 | 1 | 0 | -2.625716 | -7.338402 | 0.515016 |
| 35 | 6 | 0 | -5.323637 | -5.548527 | 0.449437 |
| 36 | 1 | 0 | -5.800809 | -6.526708 | 0.458971 |
| 37 | 1 | 0 | -5.439897 | -5.099567 | 1.442730 |
| 38 | 1 | 0 | -5.846244 | -4.917234 | -0.279327 |
| 39 | 6 | 0 | 8.114443 | 1.223060 | 0.294310 |
| 40 | 1 | 0 | 7.895912 | 1.595639 | 1.303445 |
| 41 | 1 | 0 | 9.183761 | 1.023696 | 0.233715 |
| 42 | 1 | 0 | 7.877746 | 2.019983 | -0.422154 |
| 43 | 6 | 0 | 8.114441 | -1.223070 | -0.294305 |
| 44 | 1 | 0 | 7.895913 | -1.595647 | -1.303441 |
| 45 | 1 | 0 | 9.183758 | -1.023710 | -0.233704 |
| 46 | 1 | 0 | 7.877737 | -2.019993 | 0.422157 |
| 47 | 6 | 0 | -1.347279 | 2.352699 | 0.006215 |
| 48 | 6 | 0 | -2.719521 | 2.216631 | -0.300645 |
| 49 | 6 | 0 | -0.871889 | 3.652820 | 0.291060 |
| 50 | 6 | 0 | -3.566635 | 3.308957 | -0.338266 |
| 51 | 1 | 0 | -3.116657 | 1.235486 | -0.529718 |
| 52 | 6 | 0 | -1.704656 | 4.753017 | 0.256086 |
| 53 | 1 | 0 | 0.166393 | 3.805284 | 0.563738 |

| | | | | | |
|---|---|---|---|---|---|
| 54 | 6 | 0 | -3.085245 | 4.617854 | -0.064792 |
| 55 | 1 | 0 | -4.608599 | 3.149698 | -0.583140 |
| 56 | 1 | 0 | -1.289879 | 5.725523 | 0.486090 |
| 57 | 7 | 0 | -3.915374 | 5.706367 | -0.103185 |
| 58 | 6 | 0 | -3.404424 | 7.039815 | 0.196952 |
| 59 | 1 | 0 | -2.625704 | 7.338404 | -0.515012 |
| 60 | 1 | 0 | -4.220355 | 7.757172 | 0.131689 |
| 61 | 1 | 0 | -2.986048 | 7.087703 | 1.209023 |
| 62 | 6 | 0 | -5.323628 | 5.548538 | -0.449434 |
| 63 | 1 | 0 | -5.846238 | 4.917246 | 0.279330 |
| 64 | 1 | 0 | -5.800797 | 6.526721 | -0.458967 |
| 65 | 1 | 0 | -5.439890 | 5.099579 | -1.442728 |

---------------------------------------------------------------------

**2,4,6-DMA_TOL_TR**
1011cba_tris_NMe2_pirrilium_b3lyp631dp_PCMtol_TRIPLET.log

Standard orientation:

| Center Number | Atomic Number | Atomic Type | Coordinates (Angstroms) X | Y | Z |
|---|---|---|---|---|---|
| 1 | 6 | 0 | 0.883975 | 1.194074 | 0.017525 |
| 2 | 6 | 0 | -0.494303 | 1.197154 | 0.015878 |
| 3 | 6 | 0 | -0.494366 | -1.197166 | -0.015989 |
| 4 | 6 | 0 | 0.883910 | -1.194158 | -0.017597 |
| 5 | 6 | 0 | 1.651136 | -0.000061 | -0.000029 |
| 6 | 1 | 0 | 1.389814 | 2.150049 | -0.008684 |
| 7 | 1 | 0 | 1.389697 | -2.150158 | 0.008633 |
| 8 | 6 | 0 | 3.111456 | -0.000091 | -0.000016 |
| 9 | 6 | 0 | 3.858375 | -1.095468 | -0.484800 |
| 10 | 6 | 0 | 3.858405 | 1.095262 | 0.484779 |
| 11 | 6 | 0 | 5.244008 | -1.104428 | -0.487821 |
| 12 | 1 | 0 | 3.345904 | -1.955883 | -0.904955 |
| 13 | 6 | 0 | 5.244038 | 1.104174 | 0.487827 |
| 14 | 1 | 0 | 3.345955 | 1.955697 | 0.904918 |
| 15 | 6 | 0 | 5.986987 | -0.000141 | 0.000013 |
| 16 | 1 | 0 | 5.755033 | -1.971236 | -0.887775 |
| 17 | 1 | 0 | 5.755087 | 1.970967 | 0.887784 |
| 18 | 6 | 0 | -1.347287 | 2.357919 | -0.006857 |
| 19 | 6 | 0 | -2.752953 | 2.227606 | -0.155787 |
| 20 | 6 | 0 | -0.834254 | 3.677951 | 0.116690 |
| 21 | 6 | 0 | -3.585720 | 3.325796 | -0.185908 |
| 22 | 1 | 0 | -3.182352 | 1.240167 | -0.263102 |
| 23 | 6 | 0 | -1.655094 | 4.780896 | 0.089830 |
| 24 | 1 | 0 | 0.229320 | 3.840049 | 0.247725 |
| 25 | 6 | 0 | -3.067211 | 4.645690 | -0.062547 |
| 26 | 1 | 0 | -4.649341 | 3.167625 | -0.308793 |
| 27 | 1 | 0 | -1.209118 | 5.761694 | 0.190066 |
| 28 | 8 | 0 | -1.194497 | 0.000012 | -0.000065 |
| 29 | 7 | 0 | 7.363666 | -0.000167 | 0.000025 |
| 30 | 7 | 0 | -3.886019 | 5.738356 | -0.086931 |
| 31 | 6 | 0 | -3.332355 | 7.085001 | 0.037956 |
| 32 | 1 | 0 | -4.146111 | 7.807339 | 0.023456 |
| 33 | 1 | 0 | -2.786100 | 7.201457 | 0.980141 |
| 34 | 1 | 0 | -2.653244 | 7.313851 | -0.791085 |
| 35 | 6 | 0 | -5.328390 | 5.579927 | -0.257311 |
| 36 | 1 | 0 | -5.798716 | 6.561232 | -0.246342 |
| 37 | 1 | 0 | -5.563859 | 5.095183 | -1.211377 |
| 38 | 1 | 0 | -5.759169 | 4.982726 | 0.553766 |
| 39 | 6 | 0 | 8.094114 | -1.161286 | -0.483052 |
| 40 | 1 | 0 | 7.888290 | -1.364076 | -1.542611 |
| 41 | 1 | 0 | 9.163661 | -0.981310 | -0.377440 |
| 42 | 1 | 0 | 7.846307 | -2.064781 | 0.089249 |
| 43 | 6 | 0 | 8.094148 | 1.160939 | 0.483084 |
| 44 | 1 | 0 | 7.888310 | 1.363765 | 1.542633 |
| 45 | 1 | 0 | 9.163691 | 0.980920 | 0.377500 |
| 46 | 1 | 0 | 7.846387 | 2.064429 | -0.089247 |
| 47 | 6 | 0 | -1.347419 | -2.357882 | 0.006762 |
| 48 | 6 | 0 | -2.753085 | -2.227475 | 0.155585 |
| 49 | 6 | 0 | -0.834457 | -3.677954 | -0.116626 |
| 50 | 6 | 0 | -3.585921 | -3.325612 | 0.185743 |
| 51 | 1 | 0 | -3.182433 | -1.240001 | 0.262787 |

| | | | | | |
|---|---|---|---|---|---|
| 52 | 6 | 0 | -1.655364 | -4.780850 | -0.089709 |
| 53 | 1 | 0 | 0.229118 | -3.840129 | -0.247548 |
| 54 | 6 | 0 | -3.067484 | -4.645546 | 0.062554 |
| 55 | 1 | 0 | -4.649542 | -3.167372 | 0.308544 |
| 56 | 1 | 0 | -1.209435 | -5.761682 | -0.189803 |
| 57 | 7 | 0 | -3.886381 | -5.738142 | 0.087029 |
| 58 | 6 | 0 | -3.332842 | -7.084859 | -0.037655 |
| 59 | 1 | 0 | -2.653795 | -7.313673 | 0.791448 |
| 60 | 1 | 0 | -4.146670 | -7.807115 | -0.023099 |
| 61 | 1 | 0 | -2.786554 | -7.201497 | -0.979797 |
| 62 | 6 | 0 | -5.328746 | -5.579544 | 0.257291 |
| 63 | 1 | 0 | -5.759421 | -4.982452 | -0.553922 |
| 64 | 1 | 0 | -5.799168 | -6.560805 | 0.246481 |
| 65 | 1 | 0 | -5.564220 | -5.094595 | 1.211250 |

---------------------------------------------------------------------

# 2,4,6-DMA (THF)

## 2,4,6-DMA_THF_S0

1011aaa_tris_NMe2_pirrilium_b3lyp631dp_PCMthf.log

Standard orientation:

| Center Number | Atomic Number | Atomic Type | Coordinates (Angstroms) X | Y | Z |
|---|---|---|---|---|---|
| 1 | 6 | 0 | 0.923616 | 1.204626 | -0.014614 |
| 2 | 6 | 0 | -0.455170 | 1.193554 | -0.003231 |
| 3 | 6 | 0 | -0.455172 | -1.193555 | 0.003224 |
| 4 | 6 | 0 | 0.923615 | -1.204627 | 0.014608 |
| 5 | 6 | 0 | 1.664536 | -0.000001 | -0.000002 |
| 6 | 1 | 0 | 1.428475 | 2.156496 | -0.088562 |
| 7 | 1 | 0 | 1.428473 | -2.156498 | 0.088555 |
| 8 | 6 | 0 | 3.119828 | -0.000002 | 0.000000 |
| 9 | 6 | 0 | 3.862847 | -1.124497 | -0.427727 |
| 10 | 6 | 0 | 3.862847 | 1.124492 | 0.427730 |
| 11 | 6 | 0 | 5.243263 | -1.131473 | -0.438595 |
| 12 | 1 | 0 | 3.348044 | -2.003518 | -0.800932 |
| 13 | 6 | 0 | 5.243263 | 1.131466 | 0.438603 |
| 14 | 1 | 0 | 3.348043 | 2.003516 | 0.800929 |
| 15 | 6 | 0 | 5.986335 | -0.000005 | 0.000009 |
| 16 | 1 | 0 | 5.755568 | -2.013768 | -0.799241 |
| 17 | 1 | 0 | 5.755567 | 2.013762 | 0.799247 |
| 18 | 6 | 0 | -1.338592 | 2.339478 | -0.022661 |
| 19 | 6 | 0 | -2.718487 | 2.199129 | -0.290623 |
| 20 | 6 | 0 | -0.856666 | 3.644371 | 0.224965 |
| 21 | 6 | 0 | -3.565996 | 3.288604 | -0.319662 |
| 22 | 1 | 0 | -3.125205 | 1.216267 | -0.498167 |
| 23 | 6 | 0 | -1.692057 | 4.742385 | 0.201706 |
| 24 | 1 | 0 | 0.188976 | 3.804298 | 0.465000 |
| 25 | 6 | 0 | -3.081495 | 4.603813 | -0.075069 |
| 26 | 1 | 0 | -4.612485 | 3.125646 | -0.541503 |
| 27 | 1 | 0 | -1.272285 | 5.717549 | 0.410231 |
| 28 | 8 | 0 | -1.109261 | 0.000000 | -0.000002 |
| 29 | 7 | 0 | 7.350837 | -0.000009 | 0.000017 |
| 30 | 7 | 0 | -3.912580 | 5.685840 | -0.102879 |
| 31 | 6 | 0 | -3.386253 | 7.027085 | 0.129740 |
| 32 | 1 | 0 | -4.196600 | 7.748375 | 0.036521 |
| 33 | 1 | 0 | -2.956688 | 7.124524 | 1.134092 |
| 34 | 1 | 0 | -2.613442 | 7.287410 | -0.602894 |
| 35 | 6 | 0 | -5.337836 | 5.513805 | -0.365452 |
| 36 | 1 | 0 | -5.826948 | 6.485645 | -0.324386 |
| 37 | 1 | 0 | -5.516499 | 5.083563 | -1.358206 |
| 38 | 1 | 0 | -5.808455 | 4.864276 | 0.381903 |
| 39 | 6 | 0 | 8.088594 | -1.180707 | -0.437910 |
| 40 | 1 | 0 | 7.874147 | -1.424827 | -1.485244 |
| 41 | 1 | 0 | 9.156158 | -0.986744 | -0.347350 |
| 42 | 1 | 0 | 7.847921 | -2.055961 | 0.176808 |
| 43 | 6 | 0 | 8.088596 | 1.180703 | 0.437904 |
| 44 | 1 | 0 | 7.874120 | 1.424877 | 1.485218 |
| 45 | 1 | 0 | 9.156161 | 0.986719 | 0.347391 |
| 46 | 1 | 0 | 7.847956 | 2.055931 | -0.176865 |
| 47 | 6 | 0 | -1.338595 | -2.339478 | 0.022652 |
| 48 | 6 | 0 | -2.718493 | -2.199123 | 0.290594 |

| | | | | | |
|---|---|---|---|---|---|
| 49 | 6 | 0 | -0.856668 | -3.644374 | -0.224954 |
| 50 | 6 | 0 | -3.566005 | -3.288597 | 0.319633 |
| 51 | 1 | 0 | -3.125214 | -1.216259 | 0.498120 |
| 52 | 6 | 0 | -1.692061 | -4.742386 | -0.201696 |
| 53 | 1 | 0 | 0.188978 | -3.804307 | -0.464969 |
| 54 | 6 | 0 | -3.081503 | -4.603810 | 0.075059 |
| 55 | 1 | 0 | -4.612498 | -3.125634 | 0.541452 |
| 56 | 1 | 0 | -1.272286 | -5.717553 | -0.410195 |
| 57 | 7 | 0 | -3.912592 | -5.685834 | 0.102868 |
| 58 | 6 | 0 | -3.386271 | -7.027078 | -0.129773 |
| 59 | 1 | 0 | -2.613530 | -7.287464 | 0.602915 |
| 60 | 1 | 0 | -4.196643 | -7.748356 | -0.036673 |
| 61 | 1 | 0 | -2.956621 | -7.124466 | -1.134091 |
| 62 | 6 | 0 | -5.337833 | -5.513801 | 0.365524 |
| 63 | 1 | 0 | -5.808512 | -4.864323 | -0.381839 |
| 64 | 1 | 0 | -5.826935 | -6.485650 | 0.324552 |
| 65 | 1 | 0 | -5.516432 | -5.083501 | 1.358263 |

---------------------------------------------------------------------

## 2,4,6-DMA_THF_S1

1011aaa_tris_NMe2_pirrilium_TD_b3lyp631dp_PCMthf_spopt.log

Standard orientation:

| Center Number | Atomic Number | Atomic Type | Coordinates (Angstroms) X | Y | Z |
|---|---|---|---|---|---|
| 1 | 6 | 0 | 0.899509 | 1.198631 | 0.012170 |
| 2 | 6 | 0 | -0.471202 | 1.191992 | 0.015883 |
| 3 | 6 | 0 | -0.471204 | -1.191993 | -0.015895 |
| 4 | 6 | 0 | 0.899508 | -1.198633 | -0.012173 |
| 5 | 6 | 0 | 1.674006 | -0.000002 | -0.000001 |
| 6 | 1 | 0 | 1.393595 | 2.159675 | -0.022082 |
| 7 | 1 | 0 | 1.393593 | -2.159677 | 0.022083 |
| 8 | 6 | 0 | 3.122715 | -0.000003 | 0.000001 |
| 9 | 6 | 0 | 3.877111 | -1.156710 | -0.319291 |
| 10 | 6 | 0 | 3.877112 | 1.156704 | 0.319294 |
| 11 | 6 | 0 | 5.260500 | -1.164388 | -0.322400 |
| 12 | 1 | 0 | 3.370089 | -2.072957 | -0.603277 |
| 13 | 6 | 0 | 5.260501 | 1.164379 | 0.322407 |
| 14 | 1 | 0 | 3.370091 | 2.072953 | 0.603276 |
| 15 | 6 | 0 | 6.005411 | -0.000006 | 0.000007 |
| 16 | 1 | 0 | 5.771345 | -2.081153 | -0.588954 |
| 17 | 1 | 0 | 5.771347 | 2.081145 | 0.588959 |
| 18 | 6 | 0 | -1.346301 | 2.353992 | -0.002474 |
| 19 | 6 | 0 | -2.734026 | 2.214602 | -0.241174 |
| 20 | 6 | 0 | -0.855295 | 3.664154 | 0.216495 |
| 21 | 6 | 0 | -3.579245 | 3.307118 | -0.271710 |
| 22 | 1 | 0 | -3.144891 | 1.229355 | -0.422115 |
| 23 | 6 | 0 | -1.686345 | 4.763972 | 0.187223 |
| 24 | 1 | 0 | 0.195057 | 3.824838 | 0.431877 |
| 25 | 6 | 0 | -3.082882 | 4.623562 | -0.060923 |
| 26 | 1 | 0 | -4.631333 | 3.144575 | -0.465870 |
| 27 | 1 | 0 | -1.259914 | 5.741679 | 0.368466 |
| 28 | 8 | 0 | -1.168721 | 0.000000 | -0.000012 |
| 29 | 7 | 0 | 7.379992 | -0.000008 | 0.000013 |
| 30 | 7 | 0 | -3.910207 | 5.710820 | -0.090852 |
| 31 | 6 | 0 | -3.377020 | 7.054823 | 0.111086 |
| 32 | 1 | 0 | -4.186748 | 7.776121 | 0.020389 |
| 33 | 1 | 0 | -2.929626 | 7.161012 | 1.106293 |
| 34 | 1 | 0 | -2.615491 | 7.295682 | -0.639084 |
| 35 | 6 | 0 | -5.341537 | 5.544392 | -0.323391 |
| 36 | 1 | 0 | -5.823353 | 6.519495 | -0.292026 |
| 37 | 1 | 0 | -5.535510 | 5.094190 | -1.303920 |
| 38 | 1 | 0 | -5.796620 | 4.909750 | 0.445560 |
| 39 | 6 | 0 | 8.112213 | -1.215844 | -0.323050 |
| 40 | 1 | 0 | 7.896643 | -1.563538 | -1.341771 |
| 41 | 1 | 0 | 9.181365 | -1.018574 | -0.253959 |
| 42 | 1 | 0 | 7.872764 | -2.031062 | 0.371700 |
| 43 | 6 | 0 | 8.112214 | 1.215840 | 0.323027 |
| 44 | 1 | 0 | 7.896626 | 1.563584 | 1.341726 |
| 45 | 1 | 0 | 9.181366 | 1.018559 | 0.253969 |
| 46 | 1 | 0 | 7.872785 | 2.031028 | -0.371767 |

| | | | | | |
|---|---|---|---|---|---|
| 47 | 6 | 0 | -1.346304 | -2.353992 | 0.002466 |
| 48 | 6 | 0 | -2.734037 | -2.214594 | 0.241121 |
| 49 | 6 | 0 | -0.855294 | -3.664160 | -0.216452 |
| 50 | 6 | 0 | -3.579258 | -3.307108 | 0.271661 |
| 51 | 1 | 0 | -3.144906 | -1.229341 | 0.422022 |
| 52 | 6 | 0 | -1.686347 | -4.763976 | -0.187176 |
| 53 | 1 | 0 | 0.195065 | -3.824854 | -0.431791 |
| 54 | 6 | 0 | -3.082892 | -4.623558 | 0.060923 |
| 55 | 1 | 0 | -4.631353 | -3.144557 | 0.465780 |
| 56 | 1 | 0 | -1.259910 | -5.741690 | -0.368372 |
| 57 | 7 | 0 | -3.910220 | -5.710813 | 0.090856 |
| 58 | 6 | 0 | -3.377033 | -7.054821 | -0.111053 |
| 59 | 1 | 0 | -2.615562 | -7.295698 | 0.639171 |
| 60 | 1 | 0 | -4.186777 | -7.776110 | -0.020425 |
| 61 | 1 | 0 | -2.929567 | -7.161005 | -1.106228 |
| 62 | 6 | 0 | -5.341549 | -5.544378 | 0.323393 |
| 63 | 1 | 0 | -5.796639 | -4.909776 | -0.445587 |
| 64 | 1 | 0 | -5.823363 | -6.519485 | 0.292079 |
| 65 | 1 | 0 | -5.535517 | -5.094128 | 1.303901 |

---------------------------------------------------------------------

**2,4,6-DMA_THF_TR**

1011caa_tris_NMe2_pirrilium_b3lyp631dp_PCMthf_TRIPLET.log

Standard orientation:

| Center Number | Atomic Number | Atomic Type | Coordinates (Angstroms) X | Y | Z |
|---|---|---|---|---|---|
| 1 | 6 | 0 | 0.886100 | 1.195341 | 0.017014 |
| 2 | 6 | 0 | -0.491514 | 1.197482 | 0.014885 |
| 3 | 6 | 0 | -0.491516 | -1.197482 | -0.014882 |
| 4 | 6 | 0 | 0.886100 | -1.195343 | -0.017011 |
| 5 | 6 | 0 | 1.652630 | -0.000002 | 0.000002 |
| 6 | 1 | 0 | 1.390990 | 2.151847 | -0.007979 |
| 7 | 1 | 0 | 1.390988 | -2.151849 | 0.007980 |
| 8 | 6 | 0 | 3.113599 | -0.000004 | 0.000004 |
| 9 | 6 | 0 | 3.861220 | -1.099516 | -0.475519 |
| 10 | 6 | 0 | 3.861221 | 1.099508 | 0.475527 |
| 11 | 6 | 0 | 5.247668 | -1.108322 | -0.478346 |
| 12 | 1 | 0 | 3.349111 | -1.964551 | -0.886333 |
| 13 | 6 | 0 | 5.247669 | 1.108312 | 0.478357 |
| 14 | 1 | 0 | 3.349112 | 1.964546 | 0.886336 |
| 15 | 6 | 0 | 5.991096 | -0.000007 | 0.000009 |
| 16 | 1 | 0 | 5.758274 | -1.979383 | -0.869859 |
| 17 | 1 | 0 | 5.758275 | 1.979373 | 0.869868 |
| 18 | 6 | 0 | -1.346861 | 2.357626 | -0.007426 |
| 19 | 6 | 0 | -2.752536 | 2.224300 | -0.152229 |
| 20 | 6 | 0 | -0.834935 | 3.678235 | 0.113664 |
| 21 | 6 | 0 | -3.587485 | 3.321046 | -0.181676 |
| 22 | 1 | 0 | -3.181875 | 1.236371 | -0.255729 |
| 23 | 6 | 0 | -1.657629 | 4.780044 | 0.087397 |
| 24 | 1 | 0 | 0.228609 | 3.841914 | 0.242478 |
| 25 | 6 | 0 | -3.070117 | 4.642217 | -0.062722 |
| 26 | 1 | 0 | -4.651176 | 3.160972 | -0.300175 |
| 27 | 1 | 0 | -1.213190 | 5.761482 | 0.187198 |
| 28 | 8 | 0 | -1.190847 | 0.000001 | 0.000002 |
| 29 | 7 | 0 | 7.368523 | -0.000011 | 0.000017 |
| 30 | 7 | 0 | -3.889939 | 5.732693 | -0.089790 |
| 31 | 6 | 0 | -3.339120 | 7.080781 | 0.037073 |
| 32 | 1 | 0 | -4.153386 | 7.801684 | 0.004647 |
| 33 | 1 | 0 | -2.808108 | 7.202252 | 0.987206 |
| 34 | 1 | 0 | -2.646873 | 7.304067 | -0.782029 |
| 35 | 6 | 0 | -5.333620 | 5.571645 | -0.251094 |
| 36 | 1 | 0 | -5.802814 | 6.553251 | -0.253752 |
| 37 | 1 | 0 | -5.571788 | 5.071932 | -1.196272 |
| 38 | 1 | 0 | -5.760205 | 4.986599 | 0.570861 |
| 39 | 6 | 0 | 8.099029 | -1.162833 | -0.479372 |
| 40 | 1 | 0 | 7.889358 | -1.371988 | -1.536953 |
| 41 | 1 | 0 | 9.168500 | -0.980081 | -0.378823 |
| 42 | 1 | 0 | 7.854719 | -2.064183 | 0.097943 |
| 43 | 6 | 0 | 8.099029 | 1.162838 | 0.479340 |
| 44 | 1 | 0 | 7.889341 | 1.372059 | 1.536903 |

| | | | | | |
|---|---|---|---|---|---|
| 45 | 1 | 0 | 9.168501 | 0.980071 | 0.378824 |
| 46 | 1 | 0 | 7.854736 | 2.064156 | -0.098035 |
| 47 | 6 | 0 | -1.346864 | -2.357625 | 0.007428 |
| 48 | 6 | 0 | -2.752540 | -2.224296 | 0.152220 |
| 49 | 6 | 0 | -0.834938 | -3.678235 | -0.113651 |
| 50 | 6 | 0 | -3.587490 | -3.321041 | 0.181665 |
| 51 | 1 | 0 | -3.181878 | -1.236366 | 0.255712 |
| 52 | 6 | 0 | -1.657634 | -4.780042 | -0.087386 |
| 53 | 1 | 0 | 0.228607 | -3.841916 | -0.242455 |
| 54 | 6 | 0 | -3.070123 | -4.642213 | 0.062720 |
| 55 | 1 | 0 | -4.651182 | -3.160965 | 0.300156 |
| 56 | 1 | 0 | -1.213194 | -5.761483 | -0.187178 |
| 57 | 7 | 0 | -3.889946 | -5.732688 | 0.089786 |
| 58 | 6 | 0 | -3.339129 | -7.080777 | -0.037070 |
| 59 | 1 | 0 | -2.646896 | -7.304065 | 0.782043 |
| 60 | 1 | 0 | -4.153397 | -7.801678 | -0.004659 |
| 61 | 1 | 0 | -2.808102 | -7.202248 | -0.987194 |
| 62 | 6 | 0 | -5.333628 | -5.571638 | 0.251085 |
| 63 | 1 | 0 | -5.760209 | -4.986594 | -0.570872 |
| 64 | 1 | 0 | -5.802823 | -6.553244 | 0.253743 |
| 65 | 1 | 0 | -5.571797 | -5.071923 | 1.196261 |

---------------------------------------------------------------------

# 2,4,6-DMA (water)

## 2,4,6-DMA_WATER_S0

1011aaa_tris_NMe2_pirrilium_b3lyp631dp_PCMw.log

Standard orientation:

| Center Number | Atomic Number | Atomic Type | Coordinates (Angstroms) X | Y | Z |
|---|---|---|---|---|---|
| 1 | 6 | 0 | 0.924025 | 1.204646 | -0.015997 |
| 2 | 6 | 0 | -0.454536 | 1.193251 | -0.004361 |
| 3 | 6 | 0 | -0.454541 | -1.193253 | 0.004368 |
| 4 | 6 | 0 | 0.924020 | -1.204651 | 0.016002 |
| 5 | 6 | 0 | 1.665147 | -0.000004 | 0.000003 |
| 6 | 1 | 0 | 1.428111 | 2.156918 | -0.088471 |
| 7 | 1 | 0 | 1.428104 | -2.156925 | 0.088472 |
| 8 | 6 | 0 | 3.120697 | -0.000007 | 0.000004 |
| 9 | 6 | 0 | 3.864091 | -1.132234 | -0.407134 |
| 10 | 6 | 0 | 3.864095 | 1.132217 | 0.407140 |
| 11 | 6 | 0 | 5.244644 | -1.139487 | -0.417523 |
| 12 | 1 | 0 | 3.350495 | -2.019162 | -0.762490 |
| 13 | 6 | 0 | 5.244649 | 1.139464 | 0.417533 |
| 14 | 1 | 0 | 3.350502 | 2.019150 | 0.762488 |
| 15 | 6 | 0 | 5.987974 | -0.000015 | 0.000011 |
| 16 | 1 | 0 | 5.756575 | -2.029002 | -0.760536 |
| 17 | 1 | 0 | 5.756582 | 2.028981 | 0.760536 |
| 18 | 6 | 0 | -1.338246 | 2.339408 | -0.023788 |
| 19 | 6 | 0 | -2.718913 | 2.198059 | -0.287624 |
| 20 | 6 | 0 | -0.855756 | 3.644874 | 0.220301 |
| 21 | 6 | 0 | -3.567172 | 3.287269 | -0.315682 |
| 22 | 1 | 0 | -3.126332 | 1.214822 | -0.492015 |
| 23 | 6 | 0 | -1.691978 | 4.742605 | 0.198050 |
| 24 | 1 | 0 | 0.190638 | 3.805995 | 0.456098 |
| 25 | 6 | 0 | -3.082449 | 4.603104 | -0.073885 |
| 26 | 1 | 0 | -4.614316 | 3.123372 | -0.533719 |
| 27 | 1 | 0 | -1.271669 | 5.718240 | 0.403357 |
| 28 | 8 | 0 | -1.108779 | 0.000001 | 0.000006 |
| 29 | 7 | 0 | 7.352281 | -0.000022 | 0.000024 |
| 30 | 7 | 0 | -3.914083 | 5.684681 | -0.099881 |
| 31 | 6 | 0 | -3.387985 | 7.026133 | 0.132911 |
| 32 | 1 | 0 | -4.199504 | 7.746549 | 0.044009 |
| 33 | 1 | 0 | -2.954455 | 7.122091 | 1.135652 |
| 34 | 1 | 0 | -2.617907 | 7.288283 | -0.601914 |
| 35 | 6 | 0 | -5.340134 | 5.512115 | -0.358635 |
| 36 | 1 | 0 | -5.829185 | 6.483876 | -0.316103 |
| 37 | 1 | 0 | -5.521287 | 5.081751 | -1.350847 |
| 38 | 1 | 0 | -5.808383 | 4.861979 | 0.389636 |
| 39 | 6 | 0 | 8.090197 | -1.187556 | -0.419474 |
| 40 | 1 | 0 | 7.872680 | -1.450094 | -1.461636 |

| | | | | | |
|---|---|---|---|---|---|
| 41 | 1 | 0 | 9.157686 | -0.990653 | -0.335522 |
| 42 | 1 | 0 | 7.852056 | -2.052320 | 0.210834 |
| 43 | 6 | 0 | 8.090201 | 1.187541 | 0.419434 |
| 44 | 1 | 0 | 7.872622 | 1.450192 | 1.461553 |
| 45 | 1 | 0 | 9.157690 | 0.990599 | 0.335579 |
| 46 | 1 | 0 | 7.852124 | 2.052247 | -0.210980 |
| 47 | 6 | 0 | -1.338256 | -2.339405 | 0.023791 |
| 48 | 6 | 0 | -2.718927 | -2.198047 | 0.287601 |
| 49 | 6 | 0 | -0.855768 | -3.644876 | -0.220274 |
| 50 | 6 | 0 | -3.567192 | -3.287252 | 0.315654 |
| 51 | 1 | 0 | -3.126346 | -1.214806 | 0.491974 |
| 52 | 6 | 0 | -1.691996 | -4.742602 | -0.198027 |
| 53 | 1 | 0 | 0.190630 | -3.806007 | -0.456045 |
| 54 | 6 | 0 | -3.082473 | -4.603092 | 0.073877 |
| 55 | 1 | 0 | -4.614339 | -3.123347 | 0.533670 |
| 56 | 1 | 0 | -1.271686 | -5.718242 | -0.403309 |
| 57 | 7 | 0 | -3.914113 | -5.684663 | 0.099862 |
| 58 | 6 | 0 | -3.388017 | -7.026121 | -0.132902 |
| 59 | 1 | 0 | -2.617992 | -7.288290 | 0.601972 |
| 60 | 1 | 0 | -4.199553 | -7.746526 | -0.044067 |
| 61 | 1 | 0 | -2.954421 | -7.122074 | -1.135615 |
| 62 | 6 | 0 | -5.340160 | -5.512092 | 0.358633 |
| 63 | 1 | 0 | -5.808415 | -4.861955 | -0.389633 |
| 64 | 1 | 0 | -5.829216 | -6.483851 | 0.316107 |
| 65 | 1 | 0 | -5.521301 | -5.081726 | 1.350847 |

---------------------------------------------------------------------

**2,4,6-DMA_WATER_S1**

1011aaa_tris_NMe2_pirrilium_TD_b3lyp631dp_PCMw_spopt.log

Standard orientation:

| Center Number | Atomic Number | Atomic Type | Coordinates (Angstroms) X | Y | Z |
|---|---|---|---|---|---|
| 1 | 6 | 0 | -0.900250 | 1.198561 | -0.012272 |
| 2 | 6 | 0 | 0.472269 | 1.193409 | -0.013420 |
| 3 | 6 | 0 | 0.472275 | -1.193409 | 0.013416 |
| 4 | 6 | 0 | -0.900245 | -1.198567 | 0.012264 |
| 5 | 6 | 0 | -1.671921 | -0.000005 | -0.000005 |
| 6 | 1 | 0 | -1.396560 | 2.158661 | 0.018742 |
| 7 | 1 | 0 | -1.396551 | -2.158669 | -0.018750 |
| 8 | 6 | 0 | -3.123626 | -0.000008 | -0.000008 |
| 9 | 6 | 0 | -3.876926 | -1.149925 | 0.341784 |
| 10 | 6 | 0 | -3.876930 | 1.149906 | -0.341800 |
| 11 | 6 | 0 | -5.261327 | -1.157736 | 0.344750 |
| 12 | 1 | 0 | -3.369711 | -2.060533 | 0.643557 |
| 13 | 6 | 0 | -5.261331 | 1.157710 | -0.344775 |
| 14 | 1 | 0 | -3.369719 | 2.060519 | -0.643565 |
| 15 | 6 | 0 | -6.006285 | -0.000017 | -0.000020 |
| 16 | 1 | 0 | -5.771682 | -2.069568 | 0.628923 |
| 17 | 1 | 0 | -5.771690 | 2.069542 | -0.628941 |
| 18 | 6 | 0 | 1.344725 | 2.353864 | 0.006615 |
| 19 | 6 | 0 | 2.739367 | 2.211536 | 0.208850 |
| 20 | 6 | 0 | 0.848435 | 3.669355 | -0.174842 |
| 21 | 6 | 0 | 3.584801 | 3.303060 | 0.236983 |
| 22 | 1 | 0 | 3.155596 | 1.223864 | 0.361335 |
| 23 | 6 | 0 | 1.680068 | 4.767835 | -0.148718 |
| 24 | 1 | 0 | -0.207490 | 3.834940 | -0.355448 |
| 25 | 6 | 0 | 3.083605 | 4.623539 | 0.059716 |
| 26 | 1 | 0 | 4.641469 | 3.137575 | 0.401145 |
| 27 | 1 | 0 | 1.248818 | 5.748653 | -0.298883 |
| 28 | 8 | 0 | 1.169274 | 0.000002 | -0.000001 |
| 29 | 7 | 0 | -7.381492 | -0.000024 | -0.000037 |
| 30 | 7 | 0 | 3.911181 | 5.708832 | 0.085181 |
| 31 | 6 | 0 | 3.374985 | 7.055906 | -0.090157 |
| 32 | 1 | 0 | 4.189799 | 7.773889 | -0.023925 |
| 33 | 1 | 0 | 2.894095 | 7.167962 | -1.068620 |
| 34 | 1 | 0 | 2.640346 | 7.294315 | 0.687107 |
| 35 | 6 | 0 | 5.347192 | 5.540048 | 0.289039 |
| 36 | 1 | 0 | 5.827023 | 6.516168 | 0.264329 |
| 37 | 1 | 0 | 5.558401 | 5.074824 | 1.258700 |
| 38 | 1 | 0 | 5.788283 | 4.917750 | -0.497701 |
| 39 | 6 | 0 | -8.113490 | -1.209412 | 0.347175 |
| 40 | 1 | 0 | -7.896564 | -1.538170 | 1.371908 |

| | | | | | |
|---|---|---|---|---|---|
| 41 | 1 | 0 | -9.182629 | -1.012986 | 0.276004 |
| 42 | 1 | 0 | -7.875232 | -2.038232 | -0.331800 |
| 43 | 6 | 0 | -8.113493 | 1.209401 | -0.347112 |
| 44 | 1 | 0 | -7.896520 | 1.538294 | -1.371789 |
| 45 | 1 | 0 | -9.182632 | 1.012946 | -0.276028 |
| 46 | 1 | 0 | -7.875284 | 2.038140 | 0.331983 |
| 47 | 6 | 0 | 1.344735 | -2.353860 | -0.006617 |
| 48 | 6 | 0 | 2.739378 | -2.211525 | -0.208840 |
| 49 | 6 | 0 | 0.848450 | -3.669354 | 0.174829 |
| 50 | 6 | 0 | 3.584818 | -3.303045 | -0.236970 |
| 51 | 1 | 0 | 3.155605 | -1.223850 | -0.361317 |
| 52 | 6 | 0 | 1.680089 | -4.767830 | 0.148708 |
| 53 | 1 | 0 | -0.207476 | -3.834945 | 0.355424 |
| 54 | 6 | 0 | 3.083626 | -4.623527 | -0.059713 |
| 55 | 1 | 0 | 4.641486 | -3.137554 | -0.401122 |
| 56 | 1 | 0 | 1.248842 | -5.748651 | 0.298863 |
| 57 | 7 | 0 | 3.911208 | -5.708817 | -0.085175 |
| 58 | 6 | 0 | 3.375016 | -7.055893 | 0.090156 |
| 59 | 1 | 0 | 2.640389 | -7.294307 | -0.687117 |
| 60 | 1 | 0 | 4.189835 | -7.773872 | 0.023935 |
| 61 | 1 | 0 | 2.894114 | -7.167952 | 1.068612 |
| 62 | 6 | 0 | 5.347219 | -5.540025 | -0.289025 |
| 63 | 1 | 0 | 5.788303 | -4.917730 | 0.497721 |
| 64 | 1 | 0 | 5.827054 | -6.516143 | -0.264318 |
| 65 | 1 | 0 | 5.558431 | -5.074794 | -1.258682 |

---------------------------------------------------------------------

## 2,4,6-DMA_WATER_TR

1011cda_tris_NMe2_pirrilium_b3lyp631dp_PCMw_TRIPLET.log

Standard orientation:

| Center Number | Atomic Number | Atomic Type | Coordinates (Angstroms) X | Y | Z |
|---|---|---|---|---|---|
| 1 | 6 | 0 | 0.888590 | 1.196077 | 0.015091 |
| 2 | 6 | 0 | -0.488584 | 1.197721 | 0.012654 |
| 3 | 6 | 0 | -0.488590 | -1.197720 | -0.012650 |
| 4 | 6 | 0 | 0.888585 | -1.196082 | -0.015082 |
| 5 | 6 | 0 | 1.655171 | -0.000004 | 0.000006 |
| 6 | 1 | 0 | 1.392324 | 2.153193 | -0.009238 |
| 7 | 1 | 0 | 1.392315 | -2.153200 | 0.009249 |
| 8 | 6 | 0 | 3.116374 | -0.000008 | 0.000008 |
| 9 | 6 | 0 | 3.864961 | -1.109299 | -0.452216 |
| 10 | 6 | 0 | 3.864965 | 1.109280 | 0.452234 |
| 11 | 6 | 0 | 5.251790 | -1.118085 | -0.454746 |
| 12 | 1 | 0 | 3.354011 | -1.984096 | -0.842938 |
| 13 | 6 | 0 | 5.251794 | 1.118058 | 0.454769 |
| 14 | 1 | 0 | 3.354018 | 1.984081 | 0.842950 |
| 15 | 6 | 0 | 5.995721 | -0.000017 | 0.000016 |
| 16 | 1 | 0 | 5.761889 | -1.998066 | -0.826639 |
| 17 | 1 | 0 | 5.761896 | 1.998040 | 0.826657 |
| 18 | 6 | 0 | -1.345982 | 2.357054 | -0.008940 |
| 19 | 6 | 0 | -2.752425 | 2.220596 | -0.142350 |
| 20 | 6 | 0 | -0.834916 | 3.678792 | 0.102205 |
| 21 | 6 | 0 | -3.589701 | 3.315736 | -0.169420 |
| 22 | 1 | 0 | -3.181805 | 1.231834 | -0.237626 |
| 23 | 6 | 0 | -1.659724 | 4.779237 | 0.078127 |
| 24 | 1 | 0 | 0.229400 | 3.844949 | 0.220626 |
| 25 | 6 | 0 | -3.073408 | 4.638434 | -0.059961 |
| 26 | 1 | 0 | -4.653968 | 3.153086 | -0.278097 |
| 27 | 1 | 0 | -1.216016 | 5.761699 | 0.170303 |
| 28 | 8 | 0 | -1.187424 | 0.000002 | 0.000001 |
| 29 | 7 | 0 | 7.373449 | -0.000024 | 0.000028 |
| 30 | 7 | 0 | -3.894905 | 5.726922 | -0.085077 |
| 31 | 6 | 0 | -3.345530 | 7.076759 | 0.032101 |
| 32 | 1 | 0 | -4.161199 | 7.795850 | -0.001420 |
| 33 | 1 | 0 | -2.811042 | 7.203959 | 0.979421 |
| 34 | 1 | 0 | -2.656662 | 7.295641 | -0.790809 |
| 35 | 6 | 0 | -5.340045 | 5.563192 | -0.233520 |
| 36 | 1 | 0 | -5.810017 | 6.544281 | -0.235761 |
| 37 | 1 | 0 | -5.585059 | 5.059491 | -1.174668 |
| 38 | 1 | 0 | -5.758143 | 4.980505 | 0.594249 |
| 39 | 6 | 0 | 8.104068 | -1.170739 | -0.460199 |
| 40 | 1 | 0 | 7.889835 | -1.400565 | -1.512525 |

| | | | | | |
|---|---|---|---|---|---|
| 41 | 1 | 0 | 9.173538 | -0.983826 | -0.368307 |
| 42 | 1 | 0 | 7.864124 | -2.061736 | 0.134853 |
| 43 | 6 | 0 | 8.104074 | 1.170730 | 0.460146 |
| 44 | 1 | 0 | 7.889816 | 1.400662 | 1.512442 |
| 45 | 1 | 0 | 9.173543 | 0.983790 | 0.368305 |
| 46 | 1 | 0 | 7.864158 | 2.061674 | -0.135000 |
| 47 | 6 | 0 | -1.345992 | -2.357049 | 0.008942 |
| 48 | 6 | 0 | -2.752435 | -2.220585 | 0.142348 |
| 49 | 6 | 0 | -0.834932 | -3.678789 | -0.102203 |
| 50 | 6 | 0 | -3.589716 | -3.315722 | 0.169416 |
| 51 | 1 | 0 | -3.181812 | -1.231822 | 0.237623 |
| 52 | 6 | 0 | -1.659744 | -4.779231 | -0.078127 |
| 53 | 1 | 0 | 0.229384 | -3.844951 | -0.220621 |
| 54 | 6 | 0 | -3.073428 | -4.638422 | 0.059958 |
| 55 | 1 | 0 | -4.653982 | -3.153067 | 0.278090 |
| 56 | 1 | 0 | -1.216040 | -5.761695 | -0.170302 |
| 57 | 7 | 0 | -3.894930 | -5.726907 | 0.085072 |
| 58 | 6 | 0 | -3.345561 | -7.076746 | -0.032109 |
| 59 | 1 | 0 | -2.656695 | -7.295634 | 0.790802 |
| 60 | 1 | 0 | -4.161233 | -7.795834 | 0.001408 |
| 61 | 1 | 0 | -2.811071 | -7.203945 | -0.979428 |
| 62 | 6 | 0 | -5.340069 | -5.563172 | 0.233514 |
| 63 | 1 | 0 | -5.758165 | -4.980487 | -0.594259 |
| 64 | 1 | 0 | -5.810045 | -6.544259 | 0.235760 |
| 65 | 1 | 0 | -5.585082 | -5.059465 | 1.174659 |

---------------------------------------------------------------------

# 2,6-NMP-4-DMA

## 2,6-NMP-4-DMA_WATER_+2H+_S0

1039aaa_bis_NMP_NMe2_pirrilium_+2H+_b3lyp631dp_PCMw.log

Standard orientation:

| Center Number | Atomic Number | Atomic Type | Coordinates (Angstroms) X | Y | Z |
|---|---|---|---|---|---|
| 1 | 6 | 0 | -1.213190 | 2.111723 | -0.000635 |
| 2 | 6 | 0 | -1.182493 | 0.738351 | -0.010299 |
| 3 | 6 | 0 | 1.192872 | 0.761923 | -0.061086 |
| 4 | 6 | 0 | 1.195368 | 2.136245 | -0.027952 |
| 5 | 6 | 0 | -0.015918 | 2.873112 | -0.003247 |
| 6 | 1 | 0 | -2.175811 | 2.599177 | -0.042173 |
| 7 | 1 | 0 | 2.147773 | 2.642111 | 0.026350 |
| 8 | 6 | 0 | -0.031065 | 4.321307 | 0.016797 |
| 9 | 6 | 0 | 1.108218 | 5.084201 | -0.341394 |
| 10 | 6 | 0 | -1.186424 | 5.049680 | 0.394929 |
| 11 | 6 | 0 | 1.100818 | 6.462285 | -0.333434 |
| 12 | 1 | 0 | 2.013665 | 4.587262 | -0.671521 |
| 13 | 6 | 0 | -1.208721 | 6.427288 | 0.423373 |
| 14 | 1 | 0 | -2.080977 | 4.525003 | 0.711579 |
| 15 | 6 | 0 | -0.062101 | 7.188689 | 0.054604 |
| 16 | 1 | 0 | 1.995905 | 6.988347 | -0.637869 |
| 17 | 1 | 0 | -2.114826 | 6.925712 | 0.741279 |
| 18 | 6 | 0 | -2.327571 | -0.160004 | -0.018665 |
| 19 | 6 | 0 | -2.192903 | -1.505312 | -0.406306 |
| 20 | 6 | 0 | -3.609766 | 0.290159 | 0.358819 |
| 21 | 6 | 0 | -3.285407 | -2.359591 | -0.434147 |
| 22 | 1 | 0 | -1.227084 | -1.880334 | -0.723963 |
| 23 | 6 | 0 | -4.700356 | -0.558952 | 0.341900 |
| 24 | 1 | 0 | -3.752889 | 1.306589 | 0.708319 |
| 25 | 6 | 0 | -4.569515 | -1.909361 | -0.059185 |
| 26 | 1 | 0 | -3.133702 | -3.373382 | -0.781361 |
| 27 | 1 | 0 | -5.655760 | -0.179849 | 0.683956 |
| 28 | 8 | 0 | 0.011388 | 0.092233 | -0.046361 |
| 29 | 7 | 0 | -0.077234 | 8.548880 | 0.072270 |
| 30 | 7 | 0 | -5.677477 | -2.762569 | -0.034233 |
| 31 | 6 | 0 | -6.967935 | -2.269744 | -0.523155 |
| 32 | 1 | 0 | -7.104761 | -1.222108 | -0.261716 |
| 33 | 1 | 0 | -7.017106 | -2.336074 | -1.621912 |
| 34 | 6 | 0 | -5.495312 | -4.185996 | -0.299642 |

| | | | | | |
|---|---|---|---|---|---|
| 35 | 1 | 0 | -5.422809 | -4.400920 | -1.377965 |
| 36 | 1 | 0 | -4.574458 | -4.535258 | 0.169312 |
| 37 | 6 | 0 | 1.114384 | 9.307428 | -0.301662 |
| 38 | 1 | 0 | 1.411040 | 9.103099 | -1.336777 |
| 39 | 1 | 0 | 0.900050 | 10.370937 | -0.213355 |
| 40 | 1 | 0 | 1.959984 | 9.072998 | 0.354639 |
| 41 | 6 | 0 | -1.286357 | 9.270563 | 0.462751 |
| 42 | 1 | 0 | -1.581516 | 9.030756 | 1.490611 |
| 43 | 1 | 0 | -1.095013 | 10.340512 | 0.405146 |
| 44 | 1 | 0 | -2.124790 | 9.036254 | -0.202818 |
| 45 | 6 | 0 | 2.353676 | -0.114825 | -0.079697 |
| 46 | 6 | 0 | 2.232759 | -1.488287 | 0.217818 |
| 47 | 6 | 0 | 3.634928 | 0.376164 | -0.392198 |
| 48 | 6 | 0 | 3.335520 | -2.322399 | 0.215884 |
| 49 | 1 | 0 | 1.265934 | -1.898057 | 0.484678 |
| 50 | 6 | 0 | 4.743011 | -0.456090 | -0.402762 |
| 51 | 1 | 0 | 3.772431 | 1.415275 | -0.669883 |
| 52 | 6 | 0 | 4.625068 | -1.829251 | -0.091529 |
| 53 | 1 | 0 | 3.197981 | -3.359611 | 0.496386 |
| 54 | 1 | 0 | 5.697418 | -0.033601 | -0.689009 |
| 55 | 7 | 0 | 5.736381 | -2.672982 | -0.041655 |
| 56 | 6 | 0 | 7.078575 | -2.121690 | -0.192671 |
| 57 | 1 | 0 | 7.137976 | -1.149755 | 0.298410 |
| 58 | 1 | 0 | 7.350939 | -1.978201 | -1.250610 |
| 59 | 6 | 0 | 5.636672 | -4.039331 | -0.558310 |
| 60 | 1 | 0 | 5.809493 | -4.054487 | -1.646411 |
| 61 | 1 | 0 | 4.641544 | -4.445838 | -0.388999 |
| 62 | 6 | 0 | 8.104560 | -3.011774 | 0.501881 |
| 63 | 1 | 0 | 9.116519 | -2.645761 | 0.326624 |
| 64 | 1 | 0 | 7.916964 | -3.063050 | 1.575927 |
| 65 | 6 | 0 | 6.627043 | -4.963384 | 0.136482 |
| 66 | 1 | 0 | 6.429136 | -5.027434 | 1.207953 |
| 67 | 1 | 0 | 6.603664 | -5.961536 | -0.302077 |
| 68 | 6 | 0 | -6.625410 | -4.998112 | 0.323788 |
| 69 | 1 | 0 | -6.532946 | -6.052944 | 0.063622 |
| 70 | 1 | 0 | -6.635189 | -4.890249 | 1.409852 |
| 71 | 6 | 0 | -8.123883 | -3.041429 | 0.097677 |
| 72 | 1 | 0 | -8.149396 | -2.917602 | 1.181808 |
| 73 | 1 | 0 | -9.075854 | -2.725395 | -0.330131 |
| 74 | 7 | 0 | -7.974175 | -4.523138 | -0.164633 |
| 75 | 7 | 0 | 8.034934 | -4.432115 | -0.009700 |
| 76 | 6 | 0 | -9.100421 | -5.321990 | 0.426353 |
| 77 | 1 | 0 | -10.039569 | -4.952605 | 0.016977 |
| 78 | 1 | 0 | -8.958556 | -6.368607 | 0.160692 |
| 79 | 1 | 0 | -9.082376 | -5.198768 | 1.508484 |
| 80 | 6 | 0 | 9.048112 | -5.321183 | 0.651978 |
| 81 | 1 | 0 | 8.973089 | -6.316051 | 0.215311 |
| 82 | 1 | 0 | 10.039674 | -4.906219 | 0.477037 |
| 83 | 1 | 0 | 8.832530 | -5.357270 | 1.719025 |
| 84 | 1 | 0 | -8.005348 | -4.664474 | -1.180598 |
| 85 | 1 | 0 | 8.257900 | -4.415463 | -1.010626 |

---------------------------------------------------------------------

**2,6-NMP-4-DMA_WATER_+2H+_S1**
1039caa_bis_NMP_NMe2_pirrilium_+2H+_TD_b3lyp631dp_PCMw_spopt.log

Standard orientation:

| Center Number | Atomic Number | Atomic Type | Coordinates (Angstroms) X | Y | Z |
|---|---|---|---|---|---|
| 1 | 6 | 0 | -1.211820 | 2.129543 | -0.011699 |
| 2 | 6 | 0 | -1.206220 | 0.756274 | 0.002942 |
| 3 | 6 | 0 | 1.204366 | 0.764605 | -0.022548 |
| 4 | 6 | 0 | 1.206227 | 2.135667 | -0.016561 |
| 5 | 6 | 0 | -0.006357 | 2.862783 | -0.014353 |
| 6 | 1 | 0 | -2.160290 | 2.651044 | -0.021890 |
| 7 | 1 | 0 | 2.154276 | 2.658194 | 0.002141 |
| 8 | 6 | 0 | -0.010272 | 4.348786 | 0.004389 |
| 9 | 6 | 0 | -0.010956 | 5.090058 | -1.198703 |
| 10 | 6 | 0 | -0.012783 | 5.054434 | 1.228974 |
| 11 | 6 | 0 | -0.007454 | 6.466873 | -1.192924 |
| 12 | 1 | 0 | -0.020462 | 4.562240 | -2.145498 |
| 13 | 6 | 0 | -0.022810 | 6.430535 | 1.264410 |
| 14 | 1 | 0 | -0.000842 | 4.498575 | 2.159536 |

| | | | | | |
|---|---|---|---|---|---|
| 15 | 6 | 0 | -0.016743 | 7.180573 | 0.046602 |
| 16 | 1 | 0 | -0.033566 | 7.000550 | -2.132694 |
| 17 | 1 | 0 | 0.000566 | 6.935906 | 2.219752 |
| 18 | 6 | 0 | -2.359643 | -0.132971 | 0.011263 |
| 19 | 6 | 0 | -2.202359 | -1.527574 | -0.091583 |
| 20 | 6 | 0 | -3.678814 | 0.360873 | 0.126300 |
| 21 | 6 | 0 | -3.299166 | -2.386329 | -0.092582 |
| 22 | 1 | 0 | -1.209428 | -1.947294 | -0.195797 |
| 23 | 6 | 0 | -4.769219 | -0.491436 | 0.135907 |
| 24 | 1 | 0 | -3.859819 | 1.424522 | 0.235952 |
| 25 | 6 | 0 | -4.606330 | -1.888271 | 0.022518 |
| 26 | 1 | 0 | -3.120303 | -3.449637 | -0.200315 |
| 27 | 1 | 0 | -5.761644 | -0.072586 | 0.265881 |
| 28 | 8 | 0 | 0.002618 | 0.075310 | -0.001092 |
| 29 | 7 | 0 | -0.019679 | 8.535901 | 0.067031 |
| 30 | 7 | 0 | -5.746089 | -2.727298 | 0.076388 |
| 31 | 6 | 0 | -6.822179 | -2.449513 | -0.878564 |
| 32 | 1 | 0 | -6.978956 | -1.375114 | -0.971744 |
| 33 | 1 | 0 | -6.572019 | -2.829152 | -1.884411 |
| 34 | 6 | 0 | -5.536861 | -4.161036 | 0.234267 |
| 35 | 1 | 0 | -5.203585 | -4.647168 | -0.698536 |
| 36 | 1 | 0 | -4.768708 | -4.339835 | 0.989579 |
| 37 | 6 | 0 | 0.199577 | 9.338240 | -1.140765 |
| 38 | 1 | 0 | -0.752877 | 9.510215 | -1.651564 |
| 39 | 1 | 0 | 0.617146 | 10.297762 | -0.839966 |
| 40 | 1 | 0 | 0.898814 | 8.845759 | -1.812263 |
| 41 | 6 | 0 | -0.242297 | 9.300247 | 1.298698 |
| 42 | 1 | 0 | 0.709094 | 9.458932 | 1.815763 |
| 43 | 1 | 0 | -0.662182 | 10.267480 | 1.027271 |
| 44 | 1 | 0 | -0.940965 | 8.785507 | 1.953911 |
| 45 | 6 | 0 | 2.366134 | -0.116011 | -0.026141 |
| 46 | 6 | 0 | 2.246157 | -1.480101 | 0.312347 |
| 47 | 6 | 0 | 3.648919 | 0.354153 | -0.362923 |
| 48 | 6 | 0 | 3.350635 | -2.318922 | 0.329940 |
| 49 | 1 | 0 | 1.278704 | -1.877128 | 0.595587 |
| 50 | 6 | 0 | 4.755813 | -0.486713 | -0.358851 |
| 51 | 1 | 0 | 3.789606 | 1.385618 | -0.668307 |
| 52 | 6 | 0 | 4.633140 | -1.844599 | -0.008216 |
| 53 | 1 | 0 | 3.217881 | -3.349820 | 0.640235 |
| 54 | 1 | 0 | 5.712312 | -0.076397 | -0.658985 |
| 55 | 7 | 0 | 5.744980 | -2.716616 | 0.053611 |
| 56 | 6 | 0 | 7.078903 | -2.150928 | -0.104879 |
| 57 | 1 | 0 | 7.156336 | -1.222296 | 0.463521 |
| 58 | 1 | 0 | 7.316365 | -1.917210 | -1.157080 |
| 59 | 6 | 0 | 5.631036 | -4.009093 | -0.626265 |
| 60 | 1 | 0 | 5.779908 | -3.901676 | -1.714186 |
| 61 | 1 | 0 | 4.637825 | -4.431750 | -0.480090 |
| 62 | 6 | 0 | 8.128250 | -3.098423 | 0.465966 |
| 63 | 1 | 0 | 9.135247 | -2.717201 | 0.293412 |
| 64 | 1 | 0 | 7.975010 | -3.256691 | 1.534722 |
| 65 | 6 | 0 | 6.634852 | -5.005572 | -0.063696 |
| 66 | 1 | 0 | 6.461461 | -5.187715 | 0.998305 |
| 67 | 1 | 0 | 6.601829 | -5.949148 | -0.609487 |
| 68 | 6 | 0 | -6.812770 | -4.826510 | 0.738857 |
| 69 | 1 | 0 | -6.694179 | -5.908832 | 0.799650 |
| 70 | 1 | 0 | -7.099849 | -4.433333 | 1.715394 |
| 71 | 6 | 0 | -8.132750 | -3.062060 | -0.404986 |
| 72 | 1 | 0 | -8.442972 | -2.643218 | 0.553984 |
| 73 | 1 | 0 | -8.924598 | -2.920842 | -1.141432 |
| 74 | 7 | 0 | -7.973032 | -4.550576 | -0.190436 |
| 75 | 7 | 0 | 8.040939 | -4.460833 | -0.182568 |
| 76 | 6 | 0 | -9.243586 | -5.189926 | 0.287869 |
| 77 | 1 | 0 | -10.025225 | -4.998951 | -0.446275 |
| 78 | 1 | 0 | -9.076532 | -6.261333 | 0.390259 |
| 79 | 1 | 0 | -9.509982 | -4.752833 | 1.249423 |
| 80 | 6 | 0 | 9.064922 | -5.410699 | 0.366241 |
| 81 | 1 | 0 | 8.975802 | -6.359939 | -0.160025 |
| 82 | 1 | 0 | 10.054438 | -4.983601 | 0.209484 |
| 83 | 1 | 0 | 8.874539 | -5.546912 | 1.430059 |
| 84 | 1 | 0 | -7.745864 | -4.971109 | -1.098243 |
| 85 | 1 | 0 | 8.241670 | -4.341851 | -1.181979 |

---------------------------------------------------------------------

**2,6-NMP-4-DMA_WATER_+2H+_TR**

1039daa_bis_NMP_NMe2_pirrilium_+2H+_b3lyp631dp_PCMw_TRIPLET.log

Standard orientation:

| Center Number | Atomic Number | Atomic Type | Coordinates (Angstroms) X | Y | Z |
|---|---|---|---|---|---|
| 1 | 6 | 0 | -1.212554 | 2.120343 | -0.016933 |
| 2 | 6 | 0 | -1.200446 | 0.749778 | -0.037273 |
| 3 | 6 | 0 | 1.200704 | 0.759105 | -0.087540 |
| 4 | 6 | 0 | 1.203489 | 2.129708 | -0.065322 |
| 5 | 6 | 0 | -0.007197 | 2.874251 | -0.029913 |
| 6 | 1 | 0 | -2.172112 | 2.618857 | -0.041299 |
| 7 | 1 | 0 | 2.159794 | 2.633016 | -0.018997 |
| 8 | 6 | 0 | -0.011329 | 4.333969 | -0.000015 |
| 9 | 6 | 0 | 1.105064 | 5.090617 | -0.456167 |
| 10 | 6 | 0 | -1.130589 | 5.065711 | 0.488741 |
| 11 | 6 | 0 | 1.115190 | 6.465775 | -0.419366 |
| 12 | 1 | 0 | 1.964395 | 4.582428 | -0.876233 |
| 13 | 6 | 0 | -1.145230 | 6.441147 | 0.514047 |
| 14 | 1 | 0 | -1.988187 | 4.536476 | 0.885755 |
| 15 | 6 | 0 | -0.016174 | 7.190062 | 0.063873 |
| 16 | 1 | 0 | 1.976760 | 6.993074 | -0.807433 |
| 17 | 1 | 0 | -2.008407 | 6.947510 | 0.925867 |
| 18 | 6 | 0 | -2.354293 | -0.139288 | -0.047281 |
| 19 | 6 | 0 | -2.217075 | -1.502729 | -0.367010 |
| 20 | 6 | 0 | -3.650367 | 0.323756 | 0.269573 |
| 21 | 6 | 0 | -3.313237 | -2.358613 | -0.386740 |
| 22 | 1 | 0 | -1.242265 | -1.894171 | -0.632118 |
| 23 | 6 | 0 | -4.741774 | -0.526659 | 0.262169 |
| 24 | 1 | 0 | -3.805853 | 1.356213 | 0.563599 |
| 25 | 6 | 0 | -4.602264 | -1.893456 | -0.067736 |
| 26 | 1 | 0 | -3.152771 | -3.390448 | -0.674360 |
| 27 | 1 | 0 | -5.708390 | -0.134297 | 0.557943 |
| 28 | 8 | 0 | 0.002722 | 0.070185 | -0.076302 |
| 29 | 7 | 0 | -0.017810 | 8.555003 | 0.094463 |
| 30 | 7 | 0 | -5.721135 | -2.747123 | -0.014163 |
| 31 | 6 | 0 | -6.965169 | -2.308806 | -0.649626 |
| 32 | 1 | 0 | -7.109973 | -1.238278 | -0.512777 |
| 33 | 1 | 0 | -6.939975 | -2.495434 | -1.736384 |
| 34 | 6 | 0 | -5.521452 | -4.183440 | -0.160318 |
| 35 | 1 | 0 | -5.383682 | -4.483279 | -1.212862 |
| 36 | 1 | 0 | -4.629678 | -4.490982 | 0.388426 |
| 37 | 6 | 0 | 1.200116 | 9.336815 | -0.131133 |
| 38 | 1 | 0 | 1.347592 | 9.506804 | -1.203516 |
| 39 | 1 | 0 | 1.087200 | 10.300518 | 0.364225 |
| 40 | 1 | 0 | 2.068022 | 8.826649 | 0.283111 |
| 41 | 6 | 0 | -1.237226 | 9.323492 | 0.354688 |
| 42 | 1 | 0 | -1.383036 | 9.447997 | 1.433551 |
| 43 | 1 | 0 | -1.127638 | 10.307429 | -0.099994 |
| 44 | 1 | 0 | -2.104556 | 8.828970 | -0.079222 |
| 45 | 6 | 0 | 2.361752 | -0.120846 | -0.087750 |
| 46 | 6 | 0 | 2.233646 | -1.492066 | 0.219660 |
| 47 | 6 | 0 | 3.652690 | 0.355001 | -0.387228 |
| 48 | 6 | 0 | 3.335912 | -2.331717 | 0.247382 |
| 49 | 1 | 0 | 1.259702 | -1.894430 | 0.471207 |
| 50 | 6 | 0 | 4.758224 | -0.484803 | -0.371143 |
| 51 | 1 | 0 | 3.801342 | 1.391204 | -0.671796 |
| 52 | 6 | 0 | 4.628997 | -1.850643 | -0.045831 |
| 53 | 1 | 0 | 3.191748 | -3.367081 | 0.535326 |
| 54 | 1 | 0 | 5.720543 | -0.069449 | -0.643650 |
| 55 | 7 | 0 | 5.739847 | -2.712296 | 0.035381 |
| 56 | 6 | 0 | 7.080168 | -2.153399 | -0.091931 |
| 57 | 1 | 0 | 7.138656 | -1.205921 | 0.445972 |
| 58 | 1 | 0 | 7.355761 | -1.957753 | -1.142295 |
| 59 | 6 | 0 | 5.644039 | -4.036718 | -0.581554 |
| 60 | 1 | 0 | 5.822976 | -3.980436 | -1.668423 |
| 61 | 1 | 0 | 4.647637 | -4.453558 | -0.443788 |
| 62 | 6 | 0 | 8.106249 | -3.079339 | 0.552898 |
| 63 | 1 | 0 | 9.119089 | -2.704212 | 0.402879 |
| 64 | 1 | 0 | 7.914772 | -3.196839 | 1.620791 |
| 65 | 6 | 0 | 6.631141 | -5.005373 | 0.054844 |
| 66 | 1 | 0 | 6.427248 | -5.140977 | 1.118409 |
| 67 | 1 | 0 | 6.613413 | -5.972299 | -0.449127 |
| 68 | 6 | 0 | -6.688737 | -4.946403 | 0.457124 |
| 69 | 1 | 0 | -6.589835 | -6.018871 | 0.285614 |

| | | | | | |
|---|---|---|---|---|---|
| 70 | 1 | 0 | -6.758921 | -4.752119 | 1.528669 |
| 71 | 6 | 0 | -8.166089 | -3.012605 | -0.032871 |
| 72 | 1 | 0 | -8.258985 | -2.783011 | 1.029940 |
| 73 | 1 | 0 | -9.087645 | -2.742811 | -0.549941 |
| 74 | 7 | 0 | -8.008163 | -4.512626 | -0.139417 |
| 75 | 7 | 0 | 8.039477 | -4.464443 | -0.046737 |
| 76 | 6 | 0 | -9.164218 | -5.252171 | 0.468081 |
| 77 | 1 | 0 | -10.082489 | -4.920062 | -0.013889 |
| 78 | 1 | 0 | -9.016573 | -6.318616 | 0.303840 |
| 79 | 1 | 0 | -9.193213 | -5.033060 | 1.534756 |
| 80 | 6 | 0 | 9.048001 | -5.393242 | 0.563064 |
| 81 | 1 | 0 | 8.975057 | -6.358995 | 0.064870 |
| 82 | 1 | 0 | 10.040946 | -4.969079 | 0.420947 |
| 83 | 1 | 0 | 8.826037 | -5.494897 | 1.624534 |
| 84 | 1 | 0 | -7.989821 | -4.748424 | -1.137419 |
| 85 | 1 | 0 | 8.268231 | -4.381723 | -1.043760 |

---------------------------------------------------------------------

# 2,4,6-NMP

## 2,4,6-NMP_WATER_+3H+_S0

1099aaa_tris_NMP_pirrilium_+3H+_b3lyp631dp_PCMw_f2.log

Standard orientation:

| Center Number | Atomic Number | Atomic Type | Coordinates (Angstroms) X | Y | Z |
|---|---|---|---|---|---|
| 1 | 6 | 0 | -1.046051 | 1.182709 | -0.061125 |
| 2 | 6 | 0 | 0.331470 | 1.182533 | -0.021776 |
| 3 | 6 | 0 | 0.355171 | -1.197647 | 0.008841 |
| 4 | 6 | 0 | -1.021465 | -1.227092 | -0.055201 |
| 5 | 6 | 0 | -1.770755 | -0.029990 | -0.085007 |
| 6 | 1 | 0 | -1.563279 | 2.129900 | -0.021930 |
| 7 | 1 | 0 | -1.513533 | -2.185597 | -0.130234 |
| 8 | 6 | 0 | -3.229052 | -0.044615 | -0.134216 |
| 9 | 6 | 0 | -3.970843 | -1.161823 | 0.309812 |
| 10 | 6 | 0 | -3.962567 | 1.055309 | -0.624300 |
| 11 | 6 | 0 | -5.351981 | -1.176013 | 0.280280 |
| 12 | 1 | 0 | -3.462308 | -2.018861 | 0.737321 |
| 13 | 6 | 0 | -5.346223 | 1.044988 | -0.676229 |
| 14 | 1 | 0 | -3.445978 | 1.920486 | -1.025274 |
| 15 | 6 | 0 | -6.084651 | -0.069072 | -0.213389 |
| 16 | 1 | 0 | -5.866030 | -2.038598 | 0.685798 |
| 17 | 1 | 0 | -5.848257 | 1.898690 | -1.112512 |
| 18 | 6 | 0 | 1.205605 | 2.341068 | 0.015611 |
| 19 | 6 | 0 | 2.567450 | 2.215260 | 0.350514 |
| 20 | 6 | 0 | 0.720108 | 3.633398 | -0.278652 |
| 21 | 6 | 0 | 3.402389 | 3.319583 | 0.401453 |
| 22 | 1 | 0 | 2.971611 | 1.243573 | 0.607982 |
| 23 | 6 | 0 | 1.548576 | 4.737778 | -0.234694 |
| 24 | 1 | 0 | -0.310621 | 3.778276 | -0.581927 |
| 25 | 6 | 0 | 2.916872 | 4.614002 | 0.108313 |
| 26 | 1 | 0 | 4.430160 | 3.169615 | 0.705309 |
| 27 | 1 | 0 | 1.137406 | 5.700912 | -0.510876 |
| 28 | 8 | 0 | 0.993686 | -0.000941 | 0.016896 |
| 29 | 7 | 0 | -7.474953 | -0.073715 | -0.198698 |
| 30 | 7 | 0 | 3.749606 | 5.731666 | 0.109766 |
| 31 | 6 | 0 | 3.256232 | 7.010445 | 0.626074 |
| 32 | 1 | 0 | 2.194467 | 7.128282 | 0.420172 |
| 33 | 1 | 0 | 3.379071 | 7.054445 | 1.720021 |
| 34 | 6 | 0 | 5.186712 | 5.574473 | 0.309583 |
| 35 | 1 | 0 | 5.448030 | 5.499102 | 1.376793 |
| 36 | 1 | 0 | 5.533353 | 4.664939 | -0.181643 |
| 37 | 6 | 0 | -8.225637 | -1.302567 | -0.456617 |
| 38 | 1 | 0 | -7.656997 | -2.179024 | -0.154073 |
| 39 | 1 | 0 | -8.426310 | -1.404974 | -1.534666 |
| 40 | 6 | 0 | -8.234826 | 1.119609 | -0.554668 |
| 41 | 1 | 0 | -8.415154 | 1.180028 | -1.639368 |
| 42 | 1 | 0 | -7.689138 | 2.015173 | -0.258182 |
| 43 | 6 | 0 | 1.252809 | -2.337542 | 0.045179 |

| | | | | | |
|---|---|---|---|---|---|
| 44 | 6 | 0 | 2.634413 | -2.188445 | -0.200477 |
| 45 | 6 | 0 | 0.776134 | -3.631511 | 0.330800 |
| 46 | 6 | 0 | 3.489715 | -3.273398 | -0.171759 |
| 47 | 1 | 0 | 3.035304 | -1.212679 | -0.447366 |
| 48 | 6 | 0 | 1.628623 | -4.721735 | 0.367650 |
| 49 | 1 | 0 | -0.269436 | -3.792236 | 0.568369 |
| 50 | 6 | 0 | 3.011779 | -4.575912 | 0.110631 |
| 51 | 1 | 0 | 4.533343 | -3.110791 | -0.410576 |
| 52 | 1 | 0 | 1.213610 | -5.684812 | 0.633976 |
| 53 | 7 | 0 | 3.874294 | -5.669374 | 0.092967 |
| 54 | 6 | 0 | 3.350438 | -7.023581 | 0.241762 |
| 55 | 1 | 0 | 2.382681 | -7.105283 | -0.253205 |
| 56 | 1 | 0 | 3.209401 | -7.295499 | 1.299720 |
| 57 | 6 | 0 | 5.239100 | -5.541968 | 0.608524 |
| 58 | 1 | 0 | 5.255431 | -5.710260 | 1.696929 |
| 59 | 1 | 0 | 5.627950 | -4.540767 | 0.436016 |
| 60 | 6 | 0 | 4.263130 | -8.032518 | -0.448329 |
| 61 | 1 | 0 | 3.915823 | -9.050818 | -0.271568 |
| 62 | 1 | 0 | 4.313420 | -7.846818 | -1.522614 |
| 63 | 6 | 0 | 6.183387 | -6.517400 | -0.080777 |
| 64 | 1 | 0 | 6.248882 | -6.321063 | -1.152298 |
| 65 | 1 | 0 | 7.178960 | -6.473184 | 0.361966 |
| 66 | 6 | 0 | 5.948365 | 6.724050 | -0.342418 |
| 67 | 1 | 0 | 7.014944 | 6.651161 | -0.127866 |
| 68 | 1 | 0 | 5.793398 | 6.734652 | -1.422544 |
| 69 | 6 | 0 | 3.975651 | 8.183706 | -0.025659 |
| 70 | 1 | 0 | 3.802987 | 8.209948 | -1.102895 |
| 71 | 1 | 0 | 3.659917 | 9.128007 | 0.418848 |
| 72 | 7 | 0 | 5.469252 | 8.062007 | 0.169682 |
| 73 | 7 | 0 | 5.679312 | -7.934229 | 0.067101 |
| 74 | 6 | 0 | 6.220727 | 9.202656 | -0.453693 |
| 75 | 1 | 0 | 5.852315 | 10.134738 | -0.027663 |
| 76 | 1 | 0 | 7.279861 | 9.079446 | -0.232266 |
| 77 | 1 | 0 | 6.052359 | 9.182693 | -1.529669 |
| 78 | 6 | 0 | 6.589470 | -8.931522 | -0.588953 |
| 79 | 1 | 0 | 7.581467 | -8.835291 | -0.150020 |
| 80 | 1 | 0 | 6.193450 | -9.930274 | -0.411493 |
| 81 | 1 | 0 | 6.623645 | -8.719034 | -1.656586 |
| 82 | 6 | 0 | -9.536936 | -1.318593 | 0.317792 |
| 83 | 1 | 0 | -10.130608 | -2.197454 | 0.063159 |
| 84 | 1 | 0 | -9.365579 | -1.290566 | 1.395232 |
| 85 | 6 | 0 | -9.556811 | 1.158123 | 0.207651 |
| 86 | 1 | 0 | -9.386128 | 1.223524 | 1.283283 |
| 87 | 1 | 0 | -10.165810 | 2.002677 | -0.116641 |
| 88 | 7 | 0 | -10.362599 | -0.098954 | -0.020167 |
| 89 | 6 | 0 | -11.663415 | -0.076410 | 0.728496 |
| 90 | 1 | 0 | -12.231785 | 0.796007 | 0.409941 |
| 91 | 1 | 0 | -12.209887 | -0.989467 | 0.496867 |
| 92 | 1 | 0 | -11.447350 | -0.022164 | 1.794803 |
| 93 | 1 | 0 | 5.663120 | -8.155410 | 1.068593 |
| 94 | 1 | 0 | -10.588898 | -0.146872 | -1.019605 |
| 95 | 1 | 0 | 5.654163 | 8.095241 | 1.178580 |

---------------------------------------------------------------------

**2,4,6-NMP_WATER_+3H+_S1**
1099aaa_tris_NMP_pirrilium_+3H+_TD_b3lyp631dp_PCMw_spopt_uj_f.log

Standard orientation:

| Center Number | Atomic Number | Atomic Type | Coordinates (Angstroms) X | Y | Z |
|---|---|---|---|---|---|
| 1 | 6 | 0 | 1.005574 | 1.179297 | -0.040783 |
| 2 | 6 | 0 | -0.366326 | 1.191251 | -0.069733 |
| 3 | 6 | 0 | -0.401161 | -1.192588 | -0.097416 |
| 4 | 6 | 0 | 0.973929 | -1.218134 | -0.064556 |
| 5 | 6 | 0 | 1.755911 | -0.033079 | -0.033481 |
| 6 | 1 | 0 | 1.519822 | 2.129955 | -0.066706 |
| 7 | 1 | 0 | 1.456677 | -2.184734 | -0.022291 |
| 8 | 6 | 0 | 3.210644 | -0.051071 | -0.003722 |
| 9 | 6 | 0 | 3.949900 | -1.194091 | -0.394939 |
| 10 | 6 | 0 | 3.962628 | 1.069783 | 0.411034 |
| 11 | 6 | 0 | 5.333658 | -1.210955 | -0.377598 |
| 12 | 1 | 0 | 3.435571 | -2.073993 | -0.766201 |

| | | | | | |
|---|---|---|---|---|---|
| 13 | 6 | 0 | 5.351761 | 1.054249 | 0.443324 |
| 14 | 1 | 0 | 3.458826 | 1.964180 | 0.761507 |
| 15 | 6 | 0 | 6.074109 | -0.086562 | 0.046068 |
| 16 | 1 | 0 | 5.848494 | -2.095997 | -0.735535 |
| 17 | 1 | 0 | 5.866180 | 1.934999 | 0.808245 |
| 18 | 6 | 0 | -1.221259 | 2.363877 | -0.122094 |
| 19 | 6 | 0 | -2.613549 | 2.236835 | -0.344704 |
| 20 | 6 | 0 | -0.706445 | 3.674778 | 0.041064 |
| 21 | 6 | 0 | -3.439663 | 3.341572 | -0.413559 |
| 22 | 1 | 0 | -3.041407 | 1.254722 | -0.497688 |
| 23 | 6 | 0 | -1.520738 | 4.782862 | -0.029304 |
| 24 | 1 | 0 | 0.344826 | 3.829215 | 0.252738 |
| 25 | 6 | 0 | -2.919835 | 4.654558 | -0.262514 |
| 26 | 1 | 0 | -4.487750 | 3.184227 | -0.631755 |
| 27 | 1 | 0 | -1.077872 | 5.754939 | 0.143988 |
| 28 | 8 | 0 | -1.077755 | 0.009833 | -0.091252 |
| 29 | 7 | 0 | 7.484588 | -0.120048 | 0.014407 |
| 30 | 7 | 0 | -3.735413 | 5.764876 | -0.318345 |
| 31 | 6 | 0 | -3.253280 | 7.078383 | -0.745130 |
| 32 | 1 | 0 | -2.169790 | 7.137127 | -0.688511 |
| 33 | 1 | 0 | -3.525308 | 7.233238 | -1.799638 |
| 34 | 6 | 0 | -5.193068 | 5.669993 | -0.322242 |
| 35 | 1 | 0 | -5.582805 | 5.712251 | -1.349247 |
| 36 | 1 | 0 | -5.512672 | 4.727189 | 0.117465 |
| 37 | 6 | 0 | 8.153492 | -1.270633 | 0.626662 |
| 38 | 1 | 0 | 7.610080 | -2.188941 | 0.407602 |
| 39 | 1 | 0 | 8.189980 | -1.170751 | 1.725031 |
| 40 | 6 | 0 | 8.215691 | 1.117673 | 0.256457 |
| 41 | 1 | 0 | 8.244508 | 1.388235 | 1.325535 |
| 42 | 1 | 0 | 7.734733 | 1.939760 | -0.276941 |
| 43 | 6 | 0 | -1.286065 | -2.340535 | -0.111190 |
| 44 | 6 | 0 | -2.680108 | -2.187976 | 0.087703 |
| 45 | 6 | 0 | -0.803979 | -3.653754 | -0.332569 |
| 46 | 6 | 0 | -3.535866 | -3.270957 | 0.069845 |
| 47 | 1 | 0 | -3.086096 | -1.204370 | 0.286079 |
| 48 | 6 | 0 | -1.651926 | -4.741732 | -0.351109 |
| 49 | 1 | 0 | 0.247449 | -3.825558 | -0.531166 |
| 50 | 6 | 0 | -3.050557 | -4.586510 | -0.153014 |
| 51 | 1 | 0 | -4.585581 | -3.097122 | 0.269019 |
| 52 | 1 | 0 | -1.230171 | -5.713586 | -0.570249 |
| 53 | 7 | 0 | -3.906560 | -5.674000 | -0.158407 |
| 54 | 6 | 0 | -3.401170 | -7.043243 | -0.219907 |
| 55 | 1 | 0 | -2.424593 | -7.104439 | 0.257947 |
| 56 | 1 | 0 | -3.287561 | -7.375384 | -1.262634 |
| 57 | 6 | 0 | -5.291990 | -5.556208 | -0.616728 |
| 58 | 1 | 0 | -5.341465 | -5.773143 | -1.694760 |
| 59 | 1 | 0 | -5.662873 | -4.543349 | -0.481444 |
| 60 | 6 | 0 | -4.310157 | -8.000396 | 0.547953 |
| 61 | 1 | 0 | -3.972104 | -9.028842 | 0.418393 |
| 62 | 1 | 0 | -4.331133 | -7.756714 | 1.611503 |
| 63 | 6 | 0 | -6.221094 | -6.488755 | 0.149689 |
| 64 | 1 | 0 | -6.255160 | -6.236285 | 1.210922 |
| 65 | 1 | 0 | -7.227957 | -6.452519 | -0.266810 |
| 66 | 6 | 0 | -5.812662 | 6.774018 | 0.534687 |
| 67 | 1 | 0 | -6.899281 | 6.756354 | 0.446646 |
| 68 | 1 | 0 | -5.531019 | 6.663214 | 1.583101 |
| 69 | 6 | 0 | -3.830591 | 8.196105 | 0.115117 |
| 70 | 1 | 0 | -3.518767 | 8.102282 | 1.156762 |
| 71 | 1 | 0 | -3.530236 | 9.171330 | -0.268999 |
| 72 | 7 | 0 | -5.340040 | 8.142488 | 0.106063 |
| 73 | 7 | 0 | -5.735731 | -7.916230 | 0.059527 |
| 74 | 6 | 0 | -5.947935 | 9.233779 | 0.941031 |
| 75 | 1 | 0 | -5.601245 | 10.192750 | 0.558719 |
| 76 | 1 | 0 | -7.031832 | 9.164598 | 0.862715 |
| 77 | 1 | 0 | -5.630405 | 9.097120 | 1.973798 |
| 78 | 6 | 0 | -6.643043 | -8.868788 | 0.784953 |
| 79 | 1 | 0 | -7.640793 | -8.786279 | 0.356418 |
| 80 | 1 | 0 | -6.258258 | -9.878915 | 0.653991 |
| 81 | 1 | 0 | -6.658109 | -8.600338 | 1.840234 |
| 82 | 6 | 0 | 9.562630 | -1.434722 | 0.074988 |
| 83 | 1 | 0 | 10.089563 | -2.247585 | 0.576020 |
| 84 | 1 | 0 | 9.551572 | -1.611955 | -1.001943 |
| 85 | 6 | 0 | 9.634219 | 1.015204 | -0.294501 |
| 86 | 1 | 0 | 9.624246 | 0.865199 | -1.375327 |
| 87 | 1 | 0 | 10.211821 | 1.908588 | -0.053983 |

| | | | | | |
|---|---|---|---|---|---|
| 88 | 7 | 0 | 10.363305 | -0.171091 | 0.293992 |
| 89 | 6 | 0 | 11.764876 | -0.293222 | -0.228020 |
| 90 | 1 | 0 | 12.302952 | 0.625557 | 0.001499 |
| 91 | 1 | 0 | 12.241894 | -1.141699 | 0.260757 |
| 92 | 1 | 0 | 11.722094 | -0.450336 | -1.304998 |
| 93 | 1 | 0 | -5.745294 | -8.190764 | -0.929232 |
| 94 | 1 | 0 | 10.431026 | -0.022551 | 1.306846 |
| 95 | 1 | 0 | -5.653102 | 8.296647 | -0.858635 |

---------------------------------------------------------------------

**2,4,6-NMP_WATER_+3H+_TR**

1099daa_tris_NMP_pirrilium_+3H+_b3lyp631dp_PCMw_TRIPLET.log

Standard orientation:

| Center Number | Atomic Number | Atomic Type | Coordinates (Angstroms) X | Y | Z |
|---|---|---|---|---|---|
| 1 | 6 | 0 | 0.988733 | -1.164251 | 0.055610 |
| 2 | 6 | 0 | -0.390527 | -1.197605 | 0.073238 |
| 3 | 6 | 0 | -0.448329 | 1.193431 | 0.087803 |
| 4 | 6 | 0 | 0.930471 | 1.226297 | 0.074414 |
| 5 | 6 | 0 | 1.722516 | 0.049622 | 0.047431 |
| 6 | 1 | 0 | 1.517735 | -2.107161 | 0.085402 |
| 7 | 1 | 0 | 1.410130 | 2.195408 | 0.049149 |
| 8 | 6 | 0 | 3.185106 | 0.082741 | 0.023259 |
| 9 | 6 | 0 | 3.909030 | 1.203507 | 0.489628 |
| 10 | 6 | 0 | 3.943758 | -1.000304 | -0.462984 |
| 11 | 6 | 0 | 5.294395 | 1.235698 | 0.473088 |
| 12 | 1 | 0 | 3.383393 | 2.051011 | 0.917463 |
| 13 | 6 | 0 | 5.334658 | -0.970039 | -0.492752 |
| 14 | 1 | 0 | 3.445830 | -1.874254 | -0.870548 |
| 15 | 6 | 0 | 6.043665 | 0.149647 | -0.023438 |
| 16 | 1 | 0 | 5.802993 | 2.100208 | 0.886728 |
| 17 | 1 | 0 | 5.858485 | -1.820902 | -0.911668 |
| 18 | 6 | 0 | -1.213542 | -2.376079 | 0.126848 |
| 19 | 6 | 0 | -2.627770 | -2.273908 | 0.212154 |
| 20 | 6 | 0 | -0.661118 | -3.687436 | 0.103145 |
| 21 | 6 | 0 | -3.432162 | -3.390806 | 0.276310 |
| 22 | 1 | 0 | -3.087838 | -1.295836 | 0.254394 |
| 23 | 6 | 0 | -1.455139 | -4.805600 | 0.174456 |
| 24 | 1 | 0 | 0.408234 | -3.831141 | 0.010042 |
| 25 | 6 | 0 | -2.874682 | -4.699197 | 0.266523 |
| 26 | 1 | 0 | -4.499281 | -3.247474 | 0.383057 |
| 27 | 1 | 0 | -0.978480 | -5.775183 | 0.114277 |
| 28 | 8 | 0 | -1.115920 | -0.017861 | 0.066985 |
| 29 | 7 | 0 | 7.457196 | 0.200354 | 0.008020 |
| 30 | 7 | 0 | -3.668048 | -5.821222 | 0.329320 |
| 31 | 6 | 0 | -3.198271 | -7.097403 | 0.871585 |
| 32 | 1 | 0 | -2.113783 | -7.125560 | 0.930091 |
| 33 | 1 | 0 | -3.575552 | -7.204312 | 1.898565 |
| 34 | 6 | 0 | -5.122754 | -5.773494 | 0.187861 |
| 35 | 1 | 0 | -5.604457 | -5.788208 | 1.174804 |
| 36 | 1 | 0 | -5.426604 | -4.860989 | -0.320604 |
| 37 | 6 | 0 | 8.099426 | 1.376582 | -0.584279 |
| 38 | 1 | 0 | 7.548907 | 2.280721 | -0.326159 |
| 39 | 1 | 0 | 8.115238 | 1.310245 | -1.685745 |
| 40 | 6 | 0 | 8.197521 | -1.018754 | -0.292730 |
| 41 | 1 | 0 | 8.213887 | -1.249285 | -1.371837 |
| 42 | 1 | 0 | 7.733459 | -1.866069 | 0.216089 |
| 43 | 6 | 0 | -1.329453 | 2.333922 | 0.099304 |
| 44 | 6 | 0 | -2.731548 | 2.174994 | -0.043798 |
| 45 | 6 | 0 | -0.846451 | 3.657715 | 0.265595 |
| 46 | 6 | 0 | -3.589113 | 3.254399 | -0.021704 |
| 47 | 1 | 0 | -3.142222 | 1.185575 | -0.196362 |
| 48 | 6 | 0 | -1.696955 | 4.740960 | 0.289663 |
| 49 | 1 | 0 | 0.211115 | 3.841926 | 0.412082 |
| 50 | 6 | 0 | -3.102720 | 4.578359 | 0.148859 |
| 51 | 1 | 0 | -4.644689 | 3.069357 | -0.172697 |
| 52 | 1 | 0 | -1.268953 | 5.718744 | 0.464547 |
| 53 | 7 | 0 | -3.960661 | 5.662891 | 0.156348 |
| 54 | 6 | 0 | -3.457297 | 7.034694 | 0.205518 |
| 55 | 1 | 0 | -2.487592 | 7.097144 | -0.285429 |
| 56 | 1 | 0 | -3.330801 | 7.370041 | 1.245270 |
| 57 | 6 | 0 | -5.341421 | 5.546671 | 0.631696 |

| | | | | | |
|---|---|---|---|---|---|
| 58 | 1 | 0 | -5.379219 | 5.765473 | 1.709240 |
| 59 | 1 | 0 | -5.715267 | 4.534513 | 0.501093 |
| 60 | 6 | 0 | -4.376371 | 7.987055 | -0.554947 |
| 61 | 1 | 0 | -4.037435 | 9.016315 | -0.434781 |
| 62 | 1 | 0 | -4.410587 | 7.738364 | -1.617032 |
| 63 | 6 | 0 | -6.279661 | 6.475998 | -0.127614 |
| 64 | 1 | 0 | -6.325732 | 6.219685 | -1.187511 |
| 65 | 1 | 0 | -7.281710 | 6.439806 | 0.300236 |
| 66 | 6 | 0 | -5.622813 | -6.934341 | -0.671929 |
| 67 | 1 | 0 | -6.712849 | -6.946322 | -0.693846 |
| 68 | 1 | 0 | -5.240096 | -6.865072 | -1.691581 |
| 69 | 6 | 0 | -3.657203 | -8.273637 | 0.018220 |
| 70 | 1 | 0 | -3.242009 | -8.224783 | -0.989650 |
| 71 | 1 | 0 | -3.375035 | -9.217653 | 0.484439 |
| 72 | 7 | 0 | -5.160371 | -8.264390 | -0.126938 |
| 73 | 7 | 0 | -5.795670 | 7.904485 | -0.048885 |
| 74 | 6 | 0 | -5.655155 | -9.416544 | -0.955282 |
| 75 | 1 | 0 | -5.322722 | -10.342435 | -0.487535 |
| 76 | 1 | 0 | -6.742939 | -9.376044 | -0.984470 |
| 77 | 1 | 0 | -5.241869 | -9.325266 | -1.958689 |
| 78 | 6 | 0 | -6.711652 | 8.852937 | -0.768521 |
| 79 | 1 | 0 | -7.705227 | 8.770083 | -0.330518 |
| 80 | 1 | 0 | -6.327437 | 9.864168 | -0.644448 |
| 81 | 1 | 0 | -6.735991 | 8.581016 | -1.822734 |
| 82 | 6 | 0 | 9.517267 | 1.542142 | -0.055840 |
| 83 | 1 | 0 | 10.025643 | 2.374465 | -0.544125 |
| 84 | 1 | 0 | 9.524404 | 1.689769 | 1.025568 |
| 85 | 6 | 0 | 9.623147 | -0.918060 | 0.239312 |
| 86 | 1 | 0 | 9.628168 | -0.801313 | 1.324293 |
| 87 | 1 | 0 | 10.207710 | -1.796163 | -0.038049 |
| 88 | 7 | 0 | 10.328811 | 0.294636 | -0.323980 |
| 89 | 6 | 0 | 11.736883 | 0.418092 | 0.179879 |
| 90 | 1 | 0 | 12.281864 | -0.486972 | -0.084870 |
| 91 | 1 | 0 | 12.196692 | 1.286245 | -0.290464 |
| 92 | 1 | 0 | 11.709793 | 0.542377 | 1.261657 |
| 93 | 1 | 0 | -5.794159 | 8.183477 | 0.938735 |
| 94 | 1 | 0 | 10.383366 | 0.176065 | -1.341663 |
| 95 | 1 | 0 | -5.564761 | -8.376211 | 0.809234 |

---------------------------------------------------------------------

## 2,6-NMP-4-Morph

### 2,6-NMP-4-Morph_WATER_+2H+_S0
1121aaa_bis_NMP_MO_pirrilium+2H+_b3lyp631dp_PCMw.log

Standard orientation:

| Center Number | Atomic Number | Atomic Type | Coordinates (Angstroms) X | Y | Z |
|---|---|---|---|---|---|
| 1 | 6 | 0 | 1.383946 | 1.169510 | -0.003665 |
| 2 | 6 | 0 | 0.008598 | 1.181125 | -0.022573 |
| 3 | 6 | 0 | -0.037813 | -1.196099 | -0.052933 |
| 4 | 6 | 0 | 1.336343 | -1.239215 | -0.008570 |
| 5 | 6 | 0 | 2.105121 | -0.049832 | 0.011060 |
| 6 | 1 | 0 | 1.904201 | 2.114692 | -0.048280 |
| 7 | 1 | 0 | 1.815276 | -2.204535 | 0.059738 |
| 8 | 6 | 0 | 3.555908 | -0.079144 | 0.044561 |
| 9 | 6 | 0 | 4.287226 | -1.235416 | -0.317836 |
| 10 | 6 | 0 | 4.315308 | 1.043074 | 0.447694 |
| 11 | 6 | 0 | 5.665436 | -1.269624 | -0.292884 |
| 12 | 1 | 0 | 3.768450 | -2.120543 | -0.669044 |
| 13 | 6 | 0 | 5.694939 | 1.019587 | 0.489472 |
| 14 | 1 | 0 | 3.817041 | 1.944205 | 0.787855 |
| 15 | 6 | 0 | 6.428984 | -0.136669 | 0.105098 |
| 16 | 1 | 0 | 6.155005 | -2.178501 | -0.615418 |
| 17 | 1 | 0 | 6.206151 | 1.898998 | 0.856151 |
| 18 | 6 | 0 | -0.856283 | 2.349771 | -0.043941 |
| 19 | 6 | 0 | -2.219933 | 2.239089 | -0.373625 |
| 20 | 6 | 0 | -0.358969 | 3.633778 | 0.263870 |
| 21 | 6 | 0 | -3.046589 | 3.351351 | -0.406672 |

| | | | | | |
|---|---|---|---|---|---|
| 22 | 1 | 0 | -2.633073 | 1.273669 | -0.640439 |
| 23 | 6 | 0 | -1.179265 | 4.745571 | 0.238858 |
| 24 | 1 | 0 | 0.674679 | 3.765804 | 0.563289 |
| 25 | 6 | 0 | -2.549641 | 4.636892 | -0.098241 |
| 26 | 1 | 0 | -4.077224 | 3.213422 | -0.706736 |
| 27 | 1 | 0 | -0.759624 | 5.702077 | 0.525617 |
| 28 | 8 | 0 | -0.669870 | 0.005274 | -0.052165 |
| 29 | 7 | 0 | 7.804514 | -0.151544 | 0.093462 |
| 30 | 7 | 0 | -3.374396 | 5.762824 | -0.077259 |
| 31 | 6 | 0 | -2.875611 | 7.037774 | -0.597996 |
| 32 | 1 | 0 | -1.812005 | 7.148751 | -0.397075 |
| 33 | 1 | 0 | -3.003610 | 7.082956 | -1.691438 |
| 34 | 6 | 0 | -4.813031 | 5.614838 | -0.271643 |
| 35 | 1 | 0 | -5.080255 | 5.541027 | -1.337740 |
| 36 | 1 | 0 | -5.163078 | 4.707239 | 0.221073 |
| 37 | 6 | 0 | 8.563025 | -1.406587 | 0.208704 |
| 38 | 1 | 0 | 8.024458 | -2.227109 | -0.260971 |
| 39 | 1 | 0 | 8.697665 | -1.654350 | 1.272109 |
| 40 | 6 | 0 | 8.580379 | 1.022928 | 0.510518 |
| 41 | 1 | 0 | 8.701908 | 1.033500 | 1.603977 |
| 42 | 1 | 0 | 8.062207 | 1.934979 | 0.214005 |
| 43 | 6 | 0 | -0.946745 | -2.330502 | -0.073168 |
| 44 | 6 | 0 | -2.317861 | -2.173082 | 0.218430 |
| 45 | 6 | 0 | -0.489282 | -3.624500 | -0.384967 |
| 46 | 6 | 0 | -3.181498 | -3.252750 | 0.211120 |
| 47 | 1 | 0 | -2.702999 | -1.196072 | 0.485051 |
| 48 | 6 | 0 | -1.350716 | -4.709276 | -0.400978 |
| 49 | 1 | 0 | 0.546607 | -3.789535 | -0.659534 |
| 50 | 6 | 0 | -2.722095 | -4.555412 | -0.095599 |
| 51 | 1 | 0 | -4.215838 | -3.086410 | 0.486163 |
| 52 | 1 | 0 | -0.952220 | -5.673518 | -0.688071 |
| 53 | 7 | 0 | -3.593625 | -5.644106 | -0.053246 |
| 54 | 6 | 0 | -3.078655 | -6.999545 | -0.217798 |
| 55 | 1 | 0 | -2.105598 | -7.088828 | 0.265841 |
| 56 | 1 | 0 | -2.950479 | -7.266284 | -1.278895 |
| 57 | 6 | 0 | -4.961506 | -5.505882 | -0.557787 |
| 58 | 1 | 0 | -4.988965 | -5.668475 | -1.647078 |
| 59 | 1 | 0 | -5.342209 | -4.502930 | -0.376184 |
| 60 | 6 | 0 | -3.989460 | -8.007901 | 0.475344 |
| 61 | 1 | 0 | -3.650457 | -9.027199 | 0.288256 |
| 62 | 1 | 0 | -4.027650 | -7.828574 | 1.551221 |
| 63 | 6 | 0 | -5.905319 | -6.479366 | 0.134735 |
| 64 | 1 | 0 | -5.959852 | -6.288303 | 1.207818 |
| 65 | 1 | 0 | -6.904641 | -6.427929 | -0.298801 |
| 66 | 6 | 0 | -5.564415 | 6.769060 | 0.383693 |
| 67 | 1 | 0 | -6.632535 | 6.703122 | 0.174661 |
| 68 | 1 | 0 | -5.403519 | 6.778988 | 1.462963 |
| 69 | 6 | 0 | -3.583980 | 8.215915 | 0.057005 |
| 70 | 1 | 0 | -3.406180 | 8.240790 | 1.133458 |
| 71 | 1 | 0 | -3.264557 | 9.158340 | -0.388901 |
| 72 | 7 | 0 | -5.079287 | 8.103760 | -0.131232 |
| 73 | 7 | 0 | -5.410314 | -7.898143 | -0.025100 |
| 74 | 6 | 0 | -5.820657 | 9.249452 | 0.494814 |
| 75 | 1 | 0 | -5.448357 | 10.178958 | 0.066562 |
| 76 | 1 | 0 | -6.881595 | 9.132834 | 0.278566 |
| 77 | 1 | 0 | -5.647434 | 9.229238 | 1.570022 |
| 78 | 6 | 0 | -6.319625 | -8.893904 | 0.634243 |
| 79 | 1 | 0 | -7.315051 | -8.790663 | 0.204756 |
| 80 | 1 | 0 | -5.930292 | -9.893811 | 0.448734 |
| 81 | 1 | 0 | -6.343321 | -8.686404 | 1.703151 |
| 82 | 6 | 0 | 9.929095 | -1.280039 | -0.459448 |
| 83 | 1 | 0 | 10.516797 | -2.180886 | -0.266031 |
| 84 | 1 | 0 | 9.803311 | -1.166913 | -1.547372 |
| 85 | 6 | 0 | 9.951576 | 1.028276 | -0.165134 |
| 86 | 1 | 0 | 9.827376 | 1.192328 | -1.246767 |
| 87 | 1 | 0 | 10.556176 | 1.841218 | 0.244564 |
| 88 | 8 | 0 | 10.663460 | -0.180548 | 0.065603 |
| 89 | 1 | 0 | -5.404929 | -8.113560 | -1.027931 |
| 90 | 1 | 0 | -5.268364 | 8.137380 | -1.139331 |

---------------------------------------------------------------------

**2,6-NMP-4-Morph_WATER_+2H+_S1**
1121aaa_bis_NMP_MO_pirrilium+2H+_TD_b3lyp631dp_PCMw_spopt.log

Standard orientation:

| Center Number | Atomic Number | Atomic Type | Coordinates (Angstroms) X | Y | Z |
|---|---|---|---|---|---|
| 1 | 6 | 0 | 1.716379 | 0.474868 | 0.077904 |
| 2 | 6 | 0 | 0.447021 | 0.996870 | 0.079889 |
| 3 | 6 | 0 | -0.474272 | -1.231163 | 0.053547 |
| 4 | 6 | 0 | 0.792011 | -1.759282 | 0.072161 |
| 5 | 6 | 0 | 1.928946 | -0.921066 | 0.079339 |
| 6 | 1 | 0 | 2.562218 | 1.150301 | 0.066757 |
| 7 | 1 | 0 | 0.912034 | -2.834994 | 0.091032 |
| 8 | 6 | 0 | 3.302513 | -1.488444 | 0.084875 |
| 9 | 6 | 0 | 3.978523 | -1.759918 | -1.124663 |
| 10 | 6 | 0 | 3.965045 | -1.775566 | 1.297731 |
| 11 | 6 | 0 | 5.249762 | -2.290455 | -1.136113 |
| 12 | 1 | 0 | 3.488826 | -1.538457 | -2.066143 |
| 13 | 6 | 0 | 5.239783 | -2.297825 | 1.314700 |
| 14 | 1 | 0 | 3.454694 | -1.593136 | 2.236592 |
| 15 | 6 | 0 | 5.927896 | -2.567539 | 0.091024 |
| 16 | 1 | 0 | 5.735929 | -2.452221 | -2.087180 |
| 17 | 1 | 0 | 5.690378 | -2.539989 | 2.266486 |
| 18 | 6 | 0 | 0.071249 | 2.404413 | 0.076640 |
| 19 | 6 | 0 | -1.265149 | 2.798961 | -0.116890 |
| 20 | 6 | 0 | 1.025631 | 3.428328 | 0.268970 |
| 21 | 6 | 0 | -1.632419 | 4.142496 | -0.136400 |
| 22 | 1 | 0 | -2.026196 | 2.045966 | -0.280987 |
| 23 | 6 | 0 | 0.661379 | 4.763653 | 0.260300 |
| 24 | 1 | 0 | 2.064714 | 3.181492 | 0.457999 |
| 25 | 6 | 0 | -0.678072 | 5.154806 | 0.051454 |
| 26 | 1 | 0 | -2.671941 | 4.388599 | -0.317574 |
| 27 | 1 | 0 | 1.419729 | 5.515424 | 0.451955 |
| 28 | 8 | 0 | -0.646411 | 0.144349 | 0.064373 |
| 29 | 7 | 0 | 7.188525 | -3.085340 | 0.099375 |
| 30 | 7 | 0 | -1.014641 | 6.530095 | 0.085859 |
| 31 | 6 | 0 | -0.261390 | 7.419222 | -0.803081 |
| 32 | 1 | 0 | 0.795426 | 7.153502 | -0.798616 |
| 33 | 1 | 0 | -0.612213 | 7.333677 | -1.846168 |
| 34 | 6 | 0 | -2.427192 | 6.888417 | 0.123013 |
| 35 | 1 | 0 | -2.923424 | 6.763430 | -0.854750 |
| 36 | 1 | 0 | -2.949439 | 6.250143 | 0.838722 |
| 37 | 6 | 0 | 7.885474 | -3.595561 | -1.087328 |
| 38 | 1 | 0 | 7.234070 | -3.617407 | -1.954710 |
| 39 | 1 | 0 | 8.188688 | -4.621485 | -0.858978 |
| 40 | 6 | 0 | 8.076576 | -3.072564 | 1.269011 |
| 41 | 1 | 0 | 8.366458 | -4.103487 | 1.488142 |
| 42 | 1 | 0 | 7.585681 | -2.642735 | 2.136062 |
| 43 | 6 | 0 | -1.735068 | -1.962045 | 0.038864 |
| 44 | 6 | 0 | -2.961290 | -1.300403 | 0.262943 |
| 45 | 6 | 0 | -1.785673 | -3.349042 | -0.197158 |
| 46 | 6 | 0 | -4.164484 | -1.990621 | 0.262302 |
| 47 | 1 | 0 | -2.964882 | -0.236914 | 0.468586 |
| 48 | 6 | 0 | -2.990857 | -4.041349 | -0.208311 |
| 49 | 1 | 0 | -0.876850 | -3.903028 | -0.406416 |
| 50 | 6 | 0 | -4.209876 | -3.377281 | 0.023139 |
| 51 | 1 | 0 | -5.079147 | -1.448865 | 0.479404 |
| 52 | 1 | 0 | -2.971313 | -5.102846 | -0.423940 |
| 53 | 7 | 0 | -5.451877 | -4.055711 | 0.061612 |
| 54 | 6 | 0 | -5.439818 | -5.513309 | 0.051099 |
| 55 | 1 | 0 | -4.668407 | -5.880077 | 0.731144 |
| 56 | 1 | 0 | -5.224405 | -5.928576 | -0.948381 |
| 57 | 6 | 0 | -6.530304 | -3.524116 | -0.776132 |
| 58 | 1 | 0 | -6.394125 | -3.809706 | -1.833643 |
| 59 | 1 | 0 | -6.539785 | -2.435301 | -0.737290 |
| 60 | 6 | 0 | -6.768923 | -6.057848 | 0.562941 |
| 61 | 1 | 0 | -6.799876 | -7.145793 | 0.493676 |
| 62 | 1 | 0 | -6.948405 | -5.751137 | 1.594603 |
| 63 | 6 | 0 | -7.885636 | -4.008938 | -0.281744 |
| 64 | 1 | 0 | -8.081458 | -3.672340 | 0.737913 |
| 65 | 1 | 0 | -8.689194 | -3.675040 | -0.939269 |
| 66 | 6 | 0 | -2.596136 | 8.323459 | 0.609567 |
| 67 | 1 | 0 | -3.642836 | 8.627643 | 0.578017 |
| 68 | 1 | 0 | -2.209696 | 8.443131 | 1.623215 |
| 69 | 6 | 0 | -0.368736 | 8.864893 | -0.338641 |
| 70 | 1 | 0 | 0.046949 | 8.992644 | 0.662324 |
| 71 | 1 | 0 | 0.131035 | 9.540656 | -1.033557 |

| | | | | | |
|---|---|---|---|---|---|
| 72 | 7 | 0 | -1.819380 | 9.285242 | -0.260510 |
| 73 | 7 | 0 | -7.923635 | -5.520366 | -0.250418 |
| 74 | 6 | 0 | -1.975137 | 10.707529 | 0.191710 |
| 75 | 1 | 0 | -1.431021 | 11.352665 | -0.496682 |
| 76 | 1 | 0 | -3.034753 | 10.959065 | 0.186997 |
| 77 | 1 | 0 | -1.568979 | 10.797751 | 1.198259 |
| 78 | 6 | 0 | -9.245450 | -6.041569 | 0.231305 |
| 79 | 1 | 0 | -10.027529 | -5.661025 | -0.424382 |
| 80 | 1 | 0 | -9.221837 | -7.129907 | 0.199682 |
| 81 | 1 | 0 | -9.402124 | -5.694611 | 1.251911 |
| 82 | 6 | 0 | 9.142715 | -2.746320 | -1.351651 |
| 83 | 1 | 0 | 9.701773 | -3.184058 | -2.180154 |
| 84 | 1 | 0 | 8.847889 | -1.721245 | -1.616176 |
| 85 | 6 | 0 | 9.329996 | -2.238492 | 0.925710 |
| 86 | 1 | 0 | 9.036984 | -1.190754 | 0.770859 |
| 87 | 1 | 0 | 10.028416 | -2.295447 | 1.762295 |
| 88 | 8 | 0 | 9.990406 | -2.754324 | -0.215564 |
| 89 | 1 | 0 | -7.799652 | -5.851281 | -1.213390 |
| 90 | 1 | 0 | -2.207409 | 9.224931 | -1.208305 |

---------------------------------------------------------------------

**2,6-NMP-4-Morph_WATER_+2H+_TR**
1121aad_bis_NMP_MO_pirrilium+2H+_b3lyp631dp_PCMw_TRIPLET.log

Standard orientation:

| Center Number | Atomic Number | Atomic Type | Coordinates (Angstroms) X | Y | Z |
|---|---|---|---|---|---|
| 1 | 6 | 0 | 1.448822 | 1.101215 | 0.148525 |
| 2 | 6 | 0 | 0.081456 | 1.184807 | 0.088252 |
| 3 | 6 | 0 | -0.071963 | -1.211038 | 0.035480 |
| 4 | 6 | 0 | 1.293265 | -1.308712 | 0.099886 |
| 5 | 6 | 0 | 2.118127 | -0.152047 | 0.162129 |
| 6 | 1 | 0 | 2.014843 | 2.022584 | 0.139433 |
| 7 | 1 | 0 | 1.727580 | -2.297529 | 0.157211 |
| 8 | 6 | 0 | 3.572536 | -0.247535 | 0.243296 |
| 9 | 6 | 0 | 4.267677 | -1.416106 | -0.175076 |
| 10 | 6 | 0 | 4.362643 | 0.820081 | 0.751701 |
| 11 | 6 | 0 | 5.637960 | -1.519218 | -0.092073 |
| 12 | 1 | 0 | 3.718750 | -2.245001 | -0.605071 |
| 13 | 6 | 0 | 5.734930 | 0.740058 | 0.823583 |
| 14 | 1 | 0 | 3.882453 | 1.712746 | 1.133612 |
| 15 | 6 | 0 | 6.423757 | -0.435338 | 0.399876 |
| 16 | 1 | 0 | 6.107944 | -2.420675 | -0.461692 |
| 17 | 1 | 0 | 6.277327 | 1.564947 | 1.266251 |
| 18 | 6 | 0 | -0.724287 | 2.397875 | 0.048998 |
| 19 | 6 | 0 | -2.104162 | 2.344894 | -0.222970 |
| 20 | 6 | 0 | -0.160449 | 3.671553 | 0.286274 |
| 21 | 6 | 0 | -2.880701 | 3.498408 | -0.272705 |
| 22 | 1 | 0 | -2.572915 | 1.389578 | -0.425441 |
| 23 | 6 | 0 | -0.930645 | 4.819786 | 0.244797 |
| 24 | 1 | 0 | 0.889644 | 3.768926 | 0.539240 |
| 25 | 6 | 0 | -2.313119 | 4.763505 | -0.039425 |
| 26 | 1 | 0 | -3.931551 | 3.400462 | -0.517110 |
| 27 | 1 | 0 | -0.462647 | 5.770770 | 0.474705 |
| 28 | 8 | 0 | -0.678121 | 0.030480 | 0.036474 |
| 29 | 7 | 0 | 7.792245 | -0.517175 | 0.479929 |
| 30 | 7 | 0 | -3.076687 | 5.948378 | -0.029191 |
| 31 | 6 | 0 | -2.584320 | 7.092336 | -0.799803 |
| 32 | 1 | 0 | -1.499479 | 7.161494 | -0.730544 |
| 33 | 1 | 0 | -2.834248 | 6.986221 | -1.869250 |
| 34 | 6 | 0 | -4.529719 | 5.850285 | -0.084123 |
| 35 | 1 | 0 | -4.901084 | 5.651737 | -1.103520 |
| 36 | 1 | 0 | -4.869010 | 5.032056 | 0.553613 |
| 37 | 6 | 0 | 8.553430 | -1.762875 | 0.353679 |
| 38 | 1 | 0 | 7.894298 | -2.623369 | 0.286979 |
| 39 | 1 | 0 | 9.158606 | -1.862494 | 1.261313 |
| 40 | 6 | 0 | 8.674283 | 0.651286 | 0.562987 |
| 41 | 1 | 0 | 9.270101 | 0.573277 | 1.477601 |
| 42 | 1 | 0 | 8.101771 | 1.574347 | 0.580098 |
| 43 | 6 | 0 | -1.026304 | -2.311499 | -0.006717 |
| 44 | 6 | 0 | -2.392783 | -2.102496 | 0.275329 |

| | | | | | |
|---|---|---|---|---|---|
| 45 | 6 | 0 | -0.626904 | -3.622847 | -0.327409 |
| 46 | 6 | 0 | -3.301267 | -3.149384 | 0.259982 |
| 47 | 1 | 0 | -2.738038 | -1.110624 | 0.541671 |
| 48 | 6 | 0 | -1.535167 | -4.672457 | -0.354620 |
| 49 | 1 | 0 | 0.403330 | -3.830206 | -0.596526 |
| 50 | 6 | 0 | -2.896513 | -4.463592 | -0.053831 |
| 51 | 1 | 0 | -4.331729 | -2.946013 | 0.529487 |
| 52 | 1 | 0 | -1.176131 | -5.652704 | -0.642403 |
| 53 | 7 | 0 | -3.827167 | -5.519987 | -0.016408 |
| 54 | 6 | 0 | -3.348157 | -6.889070 | -0.162072 |
| 55 | 1 | 0 | -2.419465 | -7.018093 | 0.396137 |
| 56 | 1 | 0 | -3.143774 | -7.154227 | -1.213516 |
| 57 | 6 | 0 | -5.127079 | -5.330525 | -0.663371 |
| 58 | 1 | 0 | -5.052998 | -5.493355 | -1.751533 |
| 59 | 1 | 0 | -5.484596 | -4.312438 | -0.516984 |
| 60 | 6 | 0 | -4.350169 | -7.867780 | 0.440053 |
| 61 | 1 | 0 | -4.035831 | -8.898973 | 0.276544 |
| 62 | 1 | 0 | -4.477200 | -7.689770 | 1.509104 |
| 63 | 6 | 0 | -6.172003 | -6.266494 | -0.070990 |
| 64 | 1 | 0 | -6.325867 | -6.071365 | 0.991790 |
| 65 | 1 | 0 | -7.121207 | -6.181173 | -0.601494 |
| 66 | 6 | 0 | -5.165757 | 7.122443 | 0.467130 |
| 67 | 1 | 0 | -6.250855 | 7.095193 | 0.361487 |
| 68 | 1 | 0 | -4.904319 | 7.267374 | 1.516659 |
| 69 | 6 | 0 | -3.159425 | 8.394469 | -0.260480 |
| 70 | 1 | 0 | -2.859285 | 8.562760 | 0.775177 |
| 71 | 1 | 0 | -2.856402 | 9.243430 | -0.874564 |
| 72 | 7 | 0 | -4.670935 | 8.346494 | -0.269394 |
| 73 | 7 | 0 | -5.716621 | -7.704349 | -0.183790 |
| 74 | 6 | 0 | -5.278915 | 9.608560 | 0.269651 |
| 75 | 1 | 0 | -4.921746 | 10.448294 | -0.324849 |
| 76 | 1 | 0 | -6.362726 | 9.529264 | 0.198680 |
| 77 | 1 | 0 | -4.973590 | 9.722847 | 1.308941 |
| 78 | 6 | 0 | -6.714872 | -8.663441 | 0.395456 |
| 79 | 1 | 0 | -7.668786 | -8.518552 | -0.109486 |
| 80 | 1 | 0 | -6.352907 | -9.678294 | 0.236733 |
| 81 | 1 | 0 | -6.813298 | -8.458583 | 1.460689 |
| 82 | 6 | 0 | 9.492643 | -1.677928 | -0.864288 |
| 83 | 1 | 0 | 10.125633 | -2.566816 | -0.892758 |
| 84 | 1 | 0 | 8.899259 | -1.627686 | -1.788137 |
| 85 | 6 | 0 | 9.610336 | 0.653238 | -0.666236 |
| 86 | 1 | 0 | 9.014595 | 0.812678 | -1.575998 |
| 87 | 1 | 0 | 10.328468 | 1.468805 | -0.560844 |
| 88 | 8 | 0 | 10.347907 | -0.552898 | -0.748320 |
| 89 | 1 | 0 | -5.631343 | -7.922839 | -1.182809 |
| 90 | 1 | 0 | -4.968822 | 8.260302 | -1.247138 |

---------------------------------------------------------------------

# 2,6-NdMP-4-DMA

## 2,6-NdMP-4-DMA_WATER_S0

1071aaa_bis_NMP+Me_NMe2_pirrilium_b3lyp631dp_PCMw_f.log

Standard orientation:

| Center Number | Atomic Number | Atomic Type | Coordinates (Angstroms) X | Y | Z |
|---|---|---|---|---|---|
| 1 | 6 | 0 | 1.208651 | 2.359647 | -0.036228 |
| 2 | 6 | 0 | 1.187169 | 0.985707 | -0.042248 |
| 3 | 6 | 0 | -1.188611 | 0.992730 | 0.009387 |
| 4 | 6 | 0 | -1.200037 | 2.367357 | -0.004604 |
| 5 | 6 | 0 | 0.006421 | 3.112254 | -0.019639 |
| 6 | 1 | 0 | 2.168191 | 2.853008 | 0.009885 |
| 7 | 1 | 0 | -2.155414 | 2.868242 | -0.052604 |
| 8 | 6 | 0 | 0.011127 | 4.561011 | -0.015682 |
| 9 | 6 | 0 | -1.130685 | 5.308964 | 0.364626 |
| 10 | 6 | 0 | 1.158372 | 5.303999 | -0.389281 |
| 11 | 6 | 0 | -1.132865 | 6.687098 | 0.381780 |
| 12 | 1 | 0 | -2.029912 | 4.799494 | 0.692755 |
| 13 | 6 | 0 | 1.170846 | 6.682196 | -0.392952 |

| | | | | | |
|---|---|---|---|---|---|
| 14 | 1 | 0 | 2.053940 | 4.791019 | -0.721968 |
| 15 | 6 | 0 | 0.021949 | 7.428569 | -0.001474 |
| 16 | 1 | 0 | -2.029224 | 7.201210 | 0.702421 |
| 17 | 1 | 0 | 2.071044 | 7.192729 | -0.708495 |
| 18 | 6 | 0 | 2.337796 | 0.095784 | -0.044110 |
| 19 | 6 | 0 | 2.218028 | -1.249939 | 0.347847 |
| 20 | 6 | 0 | 3.613326 | 0.553622 | -0.433549 |
| 21 | 6 | 0 | 3.316994 | -2.094992 | 0.369203 |
| 22 | 1 | 0 | 1.258231 | -1.632313 | 0.675017 |
| 23 | 6 | 0 | 4.710881 | -0.286533 | -0.425192 |
| 24 | 1 | 0 | 3.746402 | 1.570798 | -0.785036 |
| 25 | 6 | 0 | 4.596053 | -1.638146 | -0.020272 |
| 26 | 1 | 0 | 3.173888 | -3.107813 | 0.722075 |
| 27 | 1 | 0 | 5.657896 | 0.104684 | -0.775460 |
| 28 | 8 | 0 | -0.002539 | 0.331225 | -0.012336 |
| 29 | 7 | 0 | 0.027443 | 8.789147 | 0.006353 |
| 30 | 7 | 0 | 5.703976 | -2.486933 | -0.052098 |
| 31 | 6 | 0 | 7.021843 | -1.974336 | 0.329487 |
| 32 | 1 | 0 | 7.149219 | -0.948343 | -0.010350 |
| 33 | 1 | 0 | 7.133622 | -1.958808 | 1.423255 |
| 34 | 6 | 0 | 5.542032 | -3.900312 | 0.273617 |
| 35 | 1 | 0 | 5.490646 | -4.073964 | 1.358726 |
| 36 | 1 | 0 | 4.610923 | -4.272705 | -0.155912 |
| 37 | 6 | 0 | -1.166278 | 9.531778 | 0.404476 |
| 38 | 1 | 0 | -1.454768 | 9.302895 | 1.436756 |
| 39 | 1 | 0 | -0.959003 | 10.598296 | 0.338393 |
| 40 | 1 | 0 | -2.014999 | 9.306601 | -0.251144 |
| 41 | 6 | 0 | 1.227805 | 9.526663 | -0.381158 |
| 42 | 1 | 0 | 1.515962 | 9.307715 | -1.415658 |
| 43 | 1 | 0 | 1.029120 | 10.593978 | -0.302715 |
| 44 | 1 | 0 | 2.073687 | 9.286776 | 0.272953 |
| 45 | 6 | 0 | -2.343728 | 0.108794 | 0.012301 |
| 46 | 6 | 0 | -2.215255 | -1.258920 | -0.307084 |
| 47 | 6 | 0 | -3.628587 | 0.586789 | 0.329987 |
| 48 | 6 | 0 | -3.313146 | -2.099428 | -0.321465 |
| 49 | 1 | 0 | -1.245311 | -1.659980 | -0.575942 |
| 50 | 6 | 0 | -4.731859 | -0.251333 | 0.323833 |
| 51 | 1 | 0 | -3.773131 | 1.620265 | 0.624620 |
| 52 | 6 | 0 | -4.607252 | -1.619046 | -0.011103 |
| 53 | 1 | 0 | -3.165235 | -3.131357 | -0.614723 |
| 54 | 1 | 0 | -5.688140 | 0.162886 | 0.614984 |
| 55 | 7 | 0 | -5.715935 | -2.463830 | -0.077053 |
| 56 | 6 | 0 | -7.054963 | -1.921105 | 0.126454 |
| 57 | 1 | 0 | -7.131932 | -0.943184 | -0.350843 |
| 58 | 1 | 0 | -7.290479 | -1.781617 | 1.191860 |
| 59 | 6 | 0 | -5.601504 | -3.853172 | 0.370971 |
| 60 | 1 | 0 | -5.696166 | -3.920450 | 1.464626 |
| 61 | 1 | 0 | -4.624009 | -4.259779 | 0.118341 |
| 62 | 6 | 0 | -8.100699 | -2.788960 | -0.566257 |
| 63 | 1 | 0 | -9.106024 | -2.420328 | -0.359722 |
| 64 | 1 | 0 | -7.933145 | -2.785043 | -1.644182 |
| 65 | 6 | 0 | -6.623121 | -4.741679 | -0.325492 |
| 66 | 1 | 0 | -6.442716 | -4.751260 | -1.401498 |
| 67 | 1 | 0 | -6.575996 | -5.762009 | 0.056925 |
| 68 | 6 | 0 | 6.642706 | -4.734465 | -0.373922 |
| 69 | 1 | 0 | 6.560891 | -5.779967 | -0.074108 |
| 70 | 1 | 0 | 6.570025 | -4.666673 | -1.460235 |
| 71 | 6 | 0 | 8.136260 | -2.778303 | -0.325620 |
| 72 | 1 | 0 | 8.078119 | -2.689266 | -1.411383 |
| 73 | 1 | 0 | 9.114846 | -2.433739 | 0.011593 |
| 74 | 7 | 0 | 8.043694 | -4.264791 | -0.015760 |
| 75 | 7 | 0 | -8.050504 | -4.247530 | -0.139479 |
| 76 | 6 | 0 | 9.040182 | -5.005472 | -0.870245 |
| 77 | 1 | 0 | 10.040523 | -4.652032 | -0.623064 |
| 78 | 1 | 0 | 8.955568 | -6.071302 | -0.660556 |
| 79 | 1 | 0 | 8.820552 | -4.809456 | -1.918876 |
| 80 | 6 | 0 | -8.966528 | -5.045896 | -1.031186 |
| 81 | 1 | 0 | -8.928160 | -6.090997 | -0.726386 |
| 82 | 1 | 0 | -9.979717 | -4.660273 | -0.923427 |
| 83 | 1 | 0 | -8.632951 | -4.941911 | -2.062673 |
| 84 | 6 | 0 | 8.360639 | -4.542637 | 1.431974 |
| 85 | 1 | 0 | 8.294149 | -5.616825 | 1.597479 |
| 86 | 1 | 0 | 9.371639 | -4.192702 | 1.635628 |
| 87 | 1 | 0 | 7.655070 | -4.026927 | 2.077516 |
| 88 | 6 | 0 | -8.509269 | -4.418603 | 1.287041 |

| | | | | | |
|---|---|---|---|---|---|
| 89 | 1 | 0 | -9.541833 | -4.079746 | 1.359738 |
| 90 | 1 | 0 | -8.442003 | -5.474668 | 1.544979 |
| 91 | 1 | 0 | -7.882427 | -3.835522 | 1.956070 |

---------------------------------------------------------------------

**2,6-NdMP-4-DMA_WATER_S1**
1071baa_bis_NMP+Me_NMe2_pirrilium_TD_b3lyp631dp_PCMw_spopt.log

Standard orientation:

---------------------------------------------------------------------

| Center Number | Atomic Number | Atomic Type | Coordinates (Angstroms) X | Y | Z |
|---|---|---|---|---|---|
| 1 | 6 | 0 | 1.205927 | 2.382279 | -0.048221 |
| 2 | 6 | 0 | 1.206311 | 1.010773 | -0.087511 |
| 3 | 6 | 0 | -1.204472 | 1.003300 | -0.083825 |
| 4 | 6 | 0 | -1.212312 | 2.375492 | -0.070549 |
| 5 | 6 | 0 | -0.005601 | 3.108946 | -0.044471 |
| 6 | 1 | 0 | 2.152454 | 2.906342 | -0.010560 |
| 7 | 1 | 0 | -2.162041 | 2.894847 | -0.088791 |
| 8 | 6 | 0 | -0.009839 | 4.594666 | -0.011518 |
| 9 | 6 | 0 | -0.026804 | 5.290110 | 1.218724 |
| 10 | 6 | 0 | 0.003173 | 5.346009 | -1.208374 |
| 11 | 6 | 0 | -0.037070 | 6.665929 | 1.265243 |
| 12 | 1 | 0 | -0.025863 | 4.726582 | 2.144729 |
| 13 | 6 | 0 | 0.007215 | 6.722673 | -1.191346 |
| 14 | 1 | 0 | 0.004343 | 4.826006 | -2.159536 |
| 15 | 6 | 0 | -0.016036 | 7.426045 | 0.053839 |
| 16 | 1 | 0 | -0.025043 | 7.163626 | 2.224818 |
| 17 | 1 | 0 | -0.008142 | 7.263989 | -2.126963 |
| 18 | 6 | 0 | 2.365916 | 0.128922 | -0.101320 |
| 19 | 6 | 0 | 2.230104 | -1.253773 | 0.117786 |
| 20 | 6 | 0 | 3.668626 | 0.620028 | -0.339119 |
| 21 | 6 | 0 | 3.334127 | -2.102646 | 0.119360 |
| 22 | 1 | 0 | 1.249814 | -1.669572 | 0.317019 |
| 23 | 6 | 0 | 4.766677 | -0.222918 | -0.348801 |
| 24 | 1 | 0 | 3.826540 | 1.671557 | -0.553511 |
| 25 | 6 | 0 | 4.627772 | -1.608025 | -0.115794 |
| 26 | 1 | 0 | 3.171978 | -3.154232 | 0.322925 |
| 27 | 1 | 0 | 5.741705 | 0.193224 | -0.577966 |
| 28 | 8 | 0 | 0.003112 | 0.322077 | -0.097399 |
| 29 | 7 | 0 | -0.018396 | 8.781159 | 0.086115 |
| 30 | 7 | 0 | 5.765044 | -2.446632 | -0.178595 |
| 31 | 6 | 0 | 6.935991 | -2.053782 | 0.609269 |
| 32 | 1 | 0 | 7.108906 | -0.981310 | 0.520451 |
| 33 | 1 | 0 | 6.789900 | -2.261266 | 1.681046 |
| 34 | 6 | 0 | 5.557816 | -3.888985 | -0.147889 |
| 35 | 1 | 0 | 5.289076 | -4.261420 | 0.853609 |
| 36 | 1 | 0 | 4.736595 | -4.153872 | -0.817681 |
| 37 | 6 | 0 | -0.256982 | 9.533764 | 1.322117 |
| 38 | 1 | 0 | 0.685945 | 9.678230 | 1.858469 |
| 39 | 1 | 0 | -0.663410 | 10.507604 | 1.054098 |
| 40 | 1 | 0 | -0.972024 | 9.017513 | 1.958355 |
| 41 | 6 | 0 | 0.216807 | 9.595107 | -1.110743 |
| 42 | 1 | 0 | -0.730901 | 9.784270 | -1.624328 |
| 43 | 1 | 0 | 0.643722 | 10.546138 | -0.795956 |
| 44 | 1 | 0 | 0.913989 | 9.103069 | -1.784510 |
| 45 | 6 | 0 | -2.358437 | 0.113740 | -0.103638 |
| 46 | 6 | 0 | -2.209320 | -1.263483 | -0.373154 |
| 47 | 6 | 0 | -3.662311 | 0.583498 | 0.143247 |
| 48 | 6 | 0 | -3.303129 | -2.115782 | -0.403478 |
| 49 | 1 | 0 | -1.225395 | -1.662017 | -0.588689 |
| 50 | 6 | 0 | -4.759108 | -0.270079 | 0.122848 |
| 51 | 1 | 0 | -3.831715 | 1.626839 | 0.387050 |
| 52 | 6 | 0 | -4.605116 | -1.642052 | -0.151748 |
| 53 | 1 | 0 | -3.145210 | -3.159314 | -0.654465 |
| 54 | 1 | 0 | -5.734475 | 0.143321 | 0.348888 |
| 55 | 7 | 0 | -5.703267 | -2.532348 | -0.218963 |
| 56 | 6 | 0 | -7.046791 | -1.968886 | -0.167391 |
| 57 | 1 | 0 | -7.104675 | -1.103774 | -0.831841 |
| 58 | 1 | 0 | -7.327826 | -1.620622 | 0.839462 |
| 59 | 6 | 0 | -5.607308 | -3.769952 | 0.559694 |
| 60 | 1 | 0 | -5.753206 | -3.582516 | 1.635099 |
| 61 | 1 | 0 | -4.614944 | -4.207632 | 0.451964 |
| 62 | 6 | 0 | -8.060928 | -2.973867 | -0.701491 |

| | | | | | |
|---|---|---|---|---|---|
| 63 | 1 | 0 | -9.077436 | -2.585322 | -0.627946 |
| 64 | 1 | 0 | -7.838873 | -3.206514 | -1.743988 |
| 65 | 6 | 0 | -6.592946 | -4.810089 | 0.045749 |
| 66 | 1 | 0 | -6.361595 | -5.068262 | -0.988902 |
| 67 | 1 | 0 | -6.569340 | -5.712881 | 0.657448 |
| 68 | 6 | 0 | 6.786301 | -4.610299 | -0.690706 |
| 69 | 1 | 0 | 6.667086 | -5.692669 | -0.628975 |
| 70 | 1 | 0 | 6.954060 | -4.325239 | -1.730393 |
| 71 | 6 | 0 | 8.192100 | -2.735280 | 0.083827 |
| 72 | 1 | 0 | 8.382200 | -2.427002 | -0.945338 |
| 73 | 1 | 0 | 9.060109 | -2.494344 | 0.698922 |
| 74 | 7 | 0 | 8.063840 | -4.252551 | 0.055020 |
| 75 | 7 | 0 | -8.028242 | -4.301161 | 0.042714 |
| 76 | 6 | 0 | 9.239794 | -4.829833 | -0.688670 |
| 77 | 1 | 0 | 10.153059 | -4.556113 | -0.161597 |
| 78 | 1 | 0 | 9.134095 | -5.913730 | -0.720621 |
| 79 | 1 | 0 | 9.252601 | -4.424348 | -1.699345 |
| 80 | 6 | 0 | -8.896854 | -5.292819 | -0.685913 |
| 81 | 1 | 0 | -8.845945 | -6.248500 | -0.165178 |
| 82 | 1 | 0 | -9.921100 | -4.921643 | -0.690684 |
| 83 | 1 | 0 | -8.530706 | -5.400612 | -1.705759 |
| 84 | 6 | 0 | 8.059502 | -4.824484 | 1.449975 |
| 85 | 1 | 0 | 7.972350 | -5.907736 | 1.377576 |
| 86 | 1 | 0 | 8.996974 | -4.554559 | 1.934532 |
| 87 | 1 | 0 | 7.223246 | -4.424954 | 2.016945 |
| 88 | 6 | 0 | -8.558406 | -4.151576 | 1.446157 |
| 89 | 1 | 0 | -9.579857 | -3.778013 | 1.388282 |
| 90 | 1 | 0 | -8.542648 | -5.129568 | 1.925212 |
| 91 | 1 | 0 | -7.942455 | -3.455533 | 2.008787 |

---------------------------------------------------------------------

## 2,6-NdMP-4-DMA_WATER_TR

1071daa_bis_NMP+Me_NMe2_pirrilium_b3lyp631dp_PCMw_TRIPLET_FRQ.log

Standard orientation:

| Center Number | Atomic Number | Atomic Type | Coordinates (Angstroms) X | Y | Z |
|---|---|---|---|---|---|
| 1 | 6 | 0 | 1.205704 | 2.376814 | -0.054674 |
| 2 | 6 | 0 | 1.204045 | 1.005971 | -0.084485 |
| 3 | 6 | 0 | -1.197963 | 0.997322 | -0.054689 |
| 4 | 6 | 0 | -1.210437 | 2.367684 | -0.028749 |
| 5 | 6 | 0 | -0.004756 | 3.120994 | -0.028346 |
| 6 | 1 | 0 | 2.160410 | 2.882114 | -0.001359 |
| 7 | 1 | 0 | -2.169626 | 2.866393 | -0.061129 |
| 8 | 6 | 0 | -0.010394 | 4.581102 | -0.005214 |
| 9 | 6 | 0 | -1.133646 | 5.313571 | 0.472845 |
| 10 | 6 | 0 | 1.106603 | 5.336720 | -0.461166 |
| 11 | 6 | 0 | -1.152278 | 6.689132 | 0.486421 |
| 12 | 1 | 0 | -1.991739 | 4.785047 | 0.869790 |
| 13 | 6 | 0 | 1.112761 | 6.712195 | -0.436068 |
| 14 | 1 | 0 | 1.969285 | 4.827317 | -0.872847 |
| 15 | 6 | 0 | -0.023370 | 7.437310 | 0.034687 |
| 16 | 1 | 0 | -2.018940 | 7.196622 | 0.889372 |
| 17 | 1 | 0 | 1.974778 | 7.238547 | -0.824369 |
| 18 | 6 | 0 | 2.364199 | 0.125247 | -0.089927 |
| 19 | 6 | 0 | 2.225966 | -1.258203 | 0.127615 |
| 20 | 6 | 0 | 3.669163 | 0.616751 | -0.316712 |
| 21 | 6 | 0 | 3.328208 | -2.106777 | 0.137259 |
| 22 | 1 | 0 | 1.244327 | -1.673729 | 0.320295 |
| 23 | 6 | 0 | 4.767065 | -0.224929 | -0.315179 |
| 24 | 1 | 0 | 3.829837 | 1.667827 | -0.530773 |
| 25 | 6 | 0 | 4.626009 | -1.611676 | -0.084880 |
| 26 | 1 | 0 | 3.164167 | -3.157732 | 0.341670 |
| 27 | 1 | 0 | 5.743134 | 0.193033 | -0.534988 |
| 28 | 8 | 0 | 0.004906 | 0.317770 | -0.087535 |
| 29 | 7 | 0 | -0.030094 | 8.802371 | 0.051516 |
| 30 | 7 | 0 | 5.757134 | -2.450794 | -0.134582 |
| 31 | 6 | 0 | 6.947288 | -2.045575 | 0.617206 |
| 32 | 1 | 0 | 7.115553 | -0.974104 | 0.512682 |
| 33 | 1 | 0 | 6.826182 | -2.241190 | 1.693985 |
| 34 | 6 | 0 | 5.556429 | -3.894158 | -0.097031 |
| 35 | 1 | 0 | 5.306381 | -4.262656 | 0.910199 |
| 36 | 1 | 0 | 4.725441 | -4.164341 | -0.752004 |

| | | | | | |
|---|---|---|---|---|---|
| 37 | 6 | 0 | -1.253614 | 9.568339 | 0.300226 |
| 38 | 1 | 0 | -1.406606 | 9.697618 | 1.377505 |
| 39 | 1 | 0 | -1.144463 | 10.550266 | -0.158727 |
| 40 | 1 | 0 | -2.116625 | 9.068660 | -0.136516 |
| 41 | 6 | 0 | 1.185429 | 9.586428 | -0.179420 |
| 42 | 1 | 0 | 1.335476 | 9.744272 | -1.253303 |
| 43 | 1 | 0 | 1.067189 | 10.555380 | 0.304204 |
| 44 | 1 | 0 | 2.054093 | 9.084654 | 0.243433 |
| 45 | 6 | 0 | -2.353081 | 0.109659 | -0.077152 |
| 46 | 6 | 0 | -2.216165 | -1.254441 | -0.411018 |
| 47 | 6 | 0 | -3.646589 | 0.569689 | 0.235156 |
| 48 | 6 | 0 | -3.311013 | -2.103802 | -0.442478 |
| 49 | 1 | 0 | -1.240855 | -1.644541 | -0.676121 |
| 50 | 6 | 0 | -4.744916 | -0.279303 | 0.213097 |
| 51 | 1 | 0 | -3.803324 | 1.599851 | 0.536619 |
| 52 | 6 | 0 | -4.605691 | -1.640165 | -0.128863 |
| 53 | 1 | 0 | -3.158557 | -3.133592 | -0.745368 |
| 54 | 1 | 0 | -5.708590 | 0.125465 | 0.495796 |
| 55 | 7 | 0 | -5.709391 | -2.513248 | -0.200292 |
| 56 | 6 | 0 | -7.046001 | -1.961035 | -0.013075 |
| 57 | 1 | 0 | -7.138817 | -1.031002 | -0.577592 |
| 58 | 1 | 0 | -7.262327 | -1.722283 | 1.040063 |
| 59 | 6 | 0 | -5.575214 | -3.841090 | 0.404363 |
| 60 | 1 | 0 | -5.645869 | -3.789908 | 1.501782 |
| 61 | 1 | 0 | -4.598745 | -4.266031 | 0.174895 |
| 62 | 6 | 0 | -8.099839 | -2.898821 | -0.591299 |
| 63 | 1 | 0 | -9.104648 | -2.517409 | -0.405755 |
| 64 | 1 | 0 | -7.950800 | -3.010085 | -1.666429 |
| 65 | 6 | 0 | -6.602972 | -4.807429 | -0.168003 |
| 66 | 1 | 0 | -6.443689 | -4.934626 | -1.239869 |
| 67 | 1 | 0 | -6.543709 | -5.779654 | 0.322901 |
| 68 | 6 | 0 | 6.777316 | -4.613482 | -0.659658 |
| 69 | 1 | 0 | 6.661814 | -5.695759 | -0.589990 |
| 70 | 1 | 0 | 6.923934 | -4.333943 | -1.704018 |
| 71 | 6 | 0 | 8.192798 | -2.730149 | 0.070900 |
| 72 | 1 | 0 | 8.361251 | -2.431107 | -0.964698 |
| 73 | 1 | 0 | 9.072533 | -2.480899 | 0.665652 |
| 74 | 7 | 0 | 8.067263 | -4.247606 | 0.059335 |
| 75 | 7 | 0 | -8.030301 | -4.302830 | -0.009796 |
| 76 | 6 | 0 | 9.230855 | -4.829571 | -0.700346 |
| 77 | 1 | 0 | 10.152913 | -4.545152 | -0.194681 |
| 78 | 1 | 0 | 9.129325 | -5.914162 | -0.715979 |
| 79 | 1 | 0 | 9.221991 | -4.437145 | -1.716235 |
| 80 | 6 | 0 | -8.957978 | -5.200480 | -0.786671 |
| 81 | 1 | 0 | -8.886529 | -6.209542 | -0.382313 |
| 82 | 1 | 0 | -9.975484 | -4.825610 | -0.680338 |
| 83 | 1 | 0 | -8.661523 | -5.192735 | -1.834486 |
| 84 | 6 | 0 | 8.089253 | -4.807220 | 1.459244 |
| 85 | 1 | 0 | 8.000664 | -5.891023 | 1.397734 |
| 86 | 1 | 0 | 9.035827 | -4.533548 | 1.923491 |
| 87 | 1 | 0 | 7.264082 | -4.402725 | 2.038826 |
| 88 | 6 | 0 | -8.459053 | -4.317825 | 1.435454 |
| 89 | 1 | 0 | -9.486510 | -3.961057 | 1.491668 |
| 90 | 1 | 0 | -8.395999 | -5.341598 | 1.802193 |
| 91 | 1 | 0 | -7.812859 | -3.674353 | 2.025915 |

---------------------------------------------------------------------

# 2,6-NMP-4-OMe

## 2,6-NMP-4-OMe_WATER_+2H+S0

1157aaa_26_NMP+2H+_4OMe_pirrilium_b3lyp631dp_PCMw.log

Standard orientation:

| Center Number | Atomic Number | Atomic Type | Coordinates (Angstroms) X | Y | Z |
|---|---|---|---|---|---|
| 1 | 6 | 0 | -1.147500 | 2.430298 | 0.031715 |
| 2 | 6 | 0 | -1.180451 | 1.051784 | -0.000132 |
| 3 | 6 | 0 | 1.198975 | 0.971561 | -0.033738 |

| | | | | | |
|---|---|---|---|---|---|
| 4 | 6 | 0 | 1.260603 | 2.348436 | 0.020700 |
| 5 | 6 | 0 | 0.081676 | 3.123441 | 0.047631 |
| 6 | 1 | 0 | -2.081486 | 2.971175 | -0.005971 |
| 7 | 1 | 0 | 2.229435 | 2.820441 | 0.092584 |
| 8 | 6 | 0 | 0.131161 | 4.585437 | 0.090320 |
| 9 | 6 | 0 | 1.245136 | 5.290231 | -0.405538 |
| 10 | 6 | 0 | -0.938122 | 5.339348 | 0.627015 |
| 11 | 6 | 0 | 1.301632 | 6.678131 | -0.380813 |
| 12 | 1 | 0 | 2.072105 | 4.752990 | -0.856797 |
| 13 | 6 | 0 | -0.888735 | 6.719668 | 0.670862 |
| 14 | 1 | 0 | -1.801880 | 4.838066 | 1.049677 |
| 15 | 6 | 0 | 0.231020 | 7.406342 | 0.163712 |
| 16 | 1 | 0 | 2.168673 | 7.180919 | -0.789552 |
| 17 | 1 | 0 | -1.701715 | 7.294964 | 1.100005 |
| 18 | 6 | 0 | -2.358940 | 0.206051 | -0.029529 |
| 19 | 6 | 0 | -2.266364 | -1.161894 | -0.351130 |
| 20 | 6 | 0 | -3.639403 | 0.725531 | 0.259422 |
| 21 | 6 | 0 | -3.390211 | -1.970160 | -0.393638 |
| 22 | 1 | 0 | -1.304941 | -1.591998 | -0.605019 |
| 23 | 6 | 0 | -4.763297 | -0.076336 | 0.224169 |
| 24 | 1 | 0 | -3.759926 | 1.762653 | 0.551215 |
| 25 | 6 | 0 | -4.673024 | -1.451042 | -0.104586 |
| 26 | 1 | 0 | -3.264449 | -3.003852 | -0.687823 |
| 27 | 1 | 0 | -5.715660 | 0.361855 | 0.495514 |
| 28 | 8 | 0 | -0.012782 | 0.363000 | -0.036686 |
| 29 | 7 | 0 | -5.809016 | -2.257151 | -0.097818 |
| 30 | 6 | 0 | -7.082161 | -1.734998 | -0.599861 |
| 31 | 1 | 0 | -7.174571 | -0.671177 | -0.392020 |
| 32 | 1 | 0 | -7.140049 | -1.855877 | -1.693185 |
| 33 | 6 | 0 | -5.687145 | -3.698308 | -0.294492 |
| 34 | 1 | 0 | -5.625460 | -3.963661 | -1.361537 |
| 35 | 1 | 0 | -4.782639 | -4.064955 | 0.191257 |
| 36 | 6 | 0 | 1.288131 | 9.517530 | -0.244347 |
| 37 | 1 | 0 | 1.439009 | 9.351681 | -1.315893 |
| 38 | 1 | 0 | 2.206502 | 9.275141 | 0.299892 |
| 39 | 6 | 0 | 2.317433 | 0.048257 | -0.062752 |
| 40 | 6 | 0 | 2.138018 | -1.327862 | 0.194241 |
| 41 | 6 | 0 | 3.621901 | 0.494494 | -0.350802 |
| 42 | 6 | 0 | 3.204278 | -2.206420 | 0.174992 |
| 43 | 1 | 0 | 1.153556 | -1.705566 | 0.443186 |
| 44 | 6 | 0 | 4.693306 | -0.381403 | -0.378212 |
| 45 | 1 | 0 | 3.805624 | 1.534038 | -0.597872 |
| 46 | 6 | 0 | 4.517503 | -1.758789 | -0.108494 |
| 47 | 1 | 0 | 3.019010 | -3.244213 | 0.422641 |
| 48 | 1 | 0 | 5.665278 | 0.010404 | -0.647094 |
| 49 | 7 | 0 | 5.592295 | -2.643377 | -0.080111 |
| 50 | 6 | 0 | 6.957999 | -2.149905 | -0.227895 |
| 51 | 1 | 0 | 7.057902 | -1.177915 | 0.255202 |
| 52 | 1 | 0 | 7.237773 | -2.028449 | -1.286085 |
| 53 | 6 | 0 | 5.438463 | -4.010922 | -0.580862 |
| 54 | 1 | 0 | 5.611761 | -4.042362 | -1.668146 |
| 55 | 1 | 0 | 4.428318 | -4.376476 | -0.409328 |
| 56 | 6 | 0 | 7.943944 | -3.074960 | 0.478665 |
| 57 | 1 | 0 | 8.970242 | -2.751745 | 0.302085 |
| 58 | 1 | 0 | 7.751948 | -3.107405 | 1.552533 |
| 59 | 6 | 0 | 6.390503 | -4.968067 | 0.123452 |
| 60 | 1 | 0 | 6.186271 | -5.019777 | 1.194277 |
| 61 | 1 | 0 | 6.328998 | -5.966643 | -0.310461 |
| 62 | 6 | 0 | -6.848764 | -4.431919 | 0.368190 |
| 63 | 1 | 0 | -6.800992 | -5.500339 | 0.156147 |
| 64 | 1 | 0 | -6.847336 | -4.273603 | 1.447877 |
| 65 | 6 | 0 | -8.264917 | -2.428272 | 0.063231 |
| 66 | 1 | 0 | -8.275992 | -2.256934 | 1.140995 |
| 67 | 1 | 0 | -9.206393 | -2.090500 | -0.371102 |
| 68 | 7 | 0 | -8.179301 | -3.923930 | -0.134687 |
| 69 | 7 | 0 | 7.818367 | -4.494716 | -0.020278 |
| 70 | 6 | 0 | -9.332634 | -4.648832 | 0.496994 |
| 71 | 1 | 0 | -10.258353 | -4.262472 | 0.073035 |
| 72 | 1 | 0 | -9.232722 | -5.711322 | 0.280272 |
| 73 | 1 | 0 | -9.305093 | -4.475875 | 1.572063 |
| 74 | 6 | 0 | 8.791766 | -5.418140 | 0.653035 |
| 75 | 1 | 0 | 8.678586 | -6.412449 | 0.223509 |
| 76 | 1 | 0 | 9.799678 | -5.044202 | 0.479853 |
| 77 | 1 | 0 | 8.570542 | -5.436803 | 1.719264 |
| 78 | 1 | 0 | 1.025720 | 10.560432 | -0.070491 |

| | | | | | |
|---|---|---|---|---|---|
| 79 | 8 | 0 | 0.180705 | 8.753026 | 0.245462 |
| 80 | 1 | 0 | -8.224163 | -4.107820 | -1.143495 |
| 81 | 1 | 0 | 8.046402 | -4.494241 | -1.020323 |

---------------------------------------------------------------------

## 2,6-NMP-4-OMe_WATER_+2H+S1

1157baa_26_NMP+2H+_4OMe_pirrilium_TD_b3lyp631dp_PCMw_spopt.log

Standard orientation:

| Center Number | Atomic Number | Atomic Type | Coordinates (Angstroms) X | Y | Z |
|---|---|---|---|---|---|
| 1 | 6 | 0 | -1.178398 | 2.365234 | 0.015259 |
| 2 | 6 | 0 | -1.194229 | 0.992279 | -0.037943 |
| 3 | 6 | 0 | 1.189943 | 0.955947 | -0.059838 |
| 4 | 6 | 0 | 1.217916 | 2.329199 | -0.002962 |
| 5 | 6 | 0 | 0.032338 | 3.112622 | 0.040294 |
| 6 | 1 | 0 | -2.128095 | 2.881569 | -0.001753 |
| 7 | 1 | 0 | 2.184980 | 2.810130 | 0.049067 |
| 8 | 6 | 0 | 0.053646 | 4.568622 | 0.098512 |
| 9 | 6 | 0 | 1.191717 | 5.309456 | -0.287331 |
| 10 | 6 | 0 | -1.069369 | 5.310170 | 0.543578 |
| 11 | 6 | 0 | 1.221309 | 6.700101 | -0.238416 |
| 12 | 1 | 0 | 2.070089 | 4.798173 | -0.666395 |
| 13 | 6 | 0 | -1.051428 | 6.692934 | 0.596190 |
| 14 | 1 | 0 | -1.961807 | 4.795945 | 0.883736 |
| 15 | 6 | 0 | 0.094487 | 7.407108 | 0.204934 |
| 16 | 1 | 0 | 2.116540 | 7.219943 | -0.556675 |
| 17 | 1 | 0 | -1.915019 | 7.246833 | 0.949578 |
| 18 | 6 | 0 | -2.367504 | 0.141568 | -0.105226 |
| 19 | 6 | 0 | -2.242137 | -1.254176 | -0.306965 |
| 20 | 6 | 0 | -3.680213 | 0.662266 | 0.023587 |
| 21 | 6 | 0 | -3.348756 | -2.077920 | -0.383147 |
| 22 | 1 | 0 | -1.259216 | -1.688110 | -0.435626 |
| 23 | 6 | 0 | -4.789374 | -0.150178 | -0.053990 |
| 24 | 1 | 0 | -3.836616 | 1.717641 | 0.211910 |
| 25 | 6 | 0 | -4.661703 | -1.552678 | -0.261412 |
| 26 | 1 | 0 | -3.191342 | -3.129884 | -0.582477 |
| 27 | 1 | 0 | -5.763401 | 0.298336 | 0.092558 |
| 28 | 8 | 0 | -0.012576 | 0.279726 | -0.065928 |
| 29 | 7 | 0 | -5.775300 | -2.366331 | -0.318012 |
| 30 | 6 | 0 | -7.073462 | -1.890652 | -0.794992 |
| 31 | 1 | 0 | -7.132645 | -0.806195 | -0.757500 |
| 32 | 1 | 0 | -7.196427 | -2.179941 | -1.849301 |
| 33 | 6 | 0 | -5.683180 | -3.823357 | -0.293528 |
| 34 | 1 | 0 | -5.701691 | -4.233464 | -1.313654 |
| 35 | 1 | 0 | -4.752247 | -4.136228 | 0.175641 |
| 36 | 6 | 0 | 1.146983 | 9.538055 | -0.088474 |
| 37 | 1 | 0 | 1.402352 | 9.381460 | -1.142435 |
| 38 | 1 | 0 | 2.017081 | 9.304234 | 0.535015 |
| 39 | 6 | 0 | 2.337713 | 0.070209 | -0.089054 |
| 40 | 6 | 0 | 2.184873 | -1.326188 | 0.092888 |
| 41 | 6 | 0 | 3.650385 | 0.554882 | -0.309628 |
| 42 | 6 | 0 | 3.267045 | -2.182166 | 0.060837 |
| 43 | 1 | 0 | 1.201660 | -1.733849 | 0.289343 |
| 44 | 6 | 0 | 4.737780 | -0.292648 | -0.341787 |
| 45 | 1 | 0 | 3.821138 | 1.608563 | -0.496513 |
| 46 | 6 | 0 | 4.582582 | -1.694211 | -0.160059 |
| 47 | 1 | 0 | 3.093053 | -3.234013 | 0.247657 |
| 48 | 1 | 0 | 5.708833 | 0.131192 | -0.560070 |
| 49 | 7 | 0 | 5.669246 | -2.548803 | -0.181333 |
| 50 | 6 | 0 | 7.039875 | -2.045722 | -0.237943 |
| 51 | 1 | 0 | 7.101741 | -1.069216 | 0.239591 |
| 52 | 1 | 0 | 7.373992 | -1.933491 | -1.279962 |
| 53 | 6 | 0 | 5.550522 | -3.934732 | -0.637993 |
| 54 | 1 | 0 | 5.770809 | -3.983904 | -1.715189 |
| 55 | 1 | 0 | 4.536652 | -4.303794 | -0.506371 |
| 56 | 6 | 0 | 7.993364 | -2.956423 | 0.532745 |
| 57 | 1 | 0 | 9.022512 | -2.619721 | 0.405423 |
| 58 | 1 | 0 | 7.747256 | -2.976268 | 1.595740 |
| 59 | 6 | 0 | 6.479719 | -4.864923 | 0.131444 |
| 60 | 1 | 0 | 6.224269 | -4.898504 | 1.191973 |
| 61 | 1 | 0 | 6.442602 | -5.871729 | -0.285079 |
| 62 | 6 | 0 | -6.810799 | -4.423653 | 0.545721 |

| | | | | | |
|---|---|---|---|---|---|
| 63 | 1 | 0 | -6.791624 | -5.512155 | 0.484912 |
| 64 | 1 | 0 | -6.731309 | -4.115229 | 1.589564 |
| 65 | 6 | 0 | -8.218977 | -2.451924 | 0.038969 |
| 66 | 1 | 0 | -8.160036 | -2.119598 | 1.076737 |
| 67 | 1 | 0 | -9.180949 | -2.160987 | -0.383673 |
| 68 | 7 | 0 | -8.164433 | -3.961239 | 0.062354 |
| 69 | 7 | 0 | 7.908129 | -4.381938 | 0.044636 |
| 70 | 6 | 0 | -9.284699 | -4.554109 | 0.868913 |
| 71 | 1 | 0 | -10.229231 | -4.212845 | 0.447511 |
| 72 | 1 | 0 | -9.214364 | -5.639174 | 0.810591 |
| 73 | 1 | 0 | -9.184044 | -4.220168 | 1.900572 |
| 74 | 6 | 0 | 8.857516 | -5.290532 | 0.772882 |
| 75 | 1 | 0 | 8.774401 | -6.288282 | 0.344472 |
| 76 | 1 | 0 | 9.868522 | -4.907320 | 0.644028 |
| 77 | 1 | 0 | 8.586538 | -5.304743 | 1.827523 |
| 78 | 1 | 0 | 0.861925 | 10.579041 | 0.063718 |
| 79 | 8 | 0 | 0.010691 | 8.761889 | 0.292203 |
| 80 | 1 | 0 | -8.283451 | -4.293324 | -0.900862 |
| 81 | 1 | 0 | 8.185244 | -4.392431 | -0.943417 |

---------------------------------------------------------------------

**2,6-NMP-4-OMe_WATER_+2H+TR**

1157caa_26_NMP+2H+_4OMe_pirrilium_b3lyp631dp_PCMw_TRIPLET.log

Standard orientation:

| Center Number | Atomic Number | Atomic Type | Coordinates (Angstroms) X | Y | Z |
|---|---|---|---|---|---|
| 1 | 6 | 0 | -1.192802 | 2.330015 | 0.034231 |
| 2 | 6 | 0 | -1.203818 | 0.950722 | -0.018911 |
| 3 | 6 | 0 | 1.188087 | 0.933931 | -0.046584 |
| 4 | 6 | 0 | 1.197505 | 2.312617 | 0.001872 |
| 5 | 6 | 0 | 0.007777 | 3.083479 | 0.051726 |
| 6 | 1 | 0 | -2.144810 | 2.843466 | 0.023923 |
| 7 | 1 | 0 | 2.158188 | 2.808386 | 0.038953 |
| 8 | 6 | 0 | 0.016700 | 4.547424 | 0.106543 |
| 9 | 6 | 0 | 1.108921 | 5.296966 | -0.371895 |
| 10 | 6 | 0 | -1.073298 | 5.272688 | 0.641875 |
| 11 | 6 | 0 | 1.127667 | 6.689681 | -0.324398 |
| 12 | 1 | 0 | 1.957864 | 4.792443 | -0.821962 |
| 13 | 6 | 0 | -1.068458 | 6.657338 | 0.691439 |
| 14 | 1 | 0 | -1.927772 | 4.745550 | 1.053995 |
| 15 | 6 | 0 | 0.033141 | 7.383460 | 0.208794 |
| 16 | 1 | 0 | 1.988320 | 7.219073 | -0.714416 |
| 17 | 1 | 0 | -1.907016 | 7.201403 | 1.113924 |
| 18 | 6 | 0 | -2.368388 | 0.110115 | -0.088846 |
| 19 | 6 | 0 | -2.243882 | -1.295903 | -0.247695 |
| 20 | 6 | 0 | -3.688352 | 0.636150 | -0.009295 |
| 21 | 6 | 0 | -3.348108 | -2.116306 | -0.330514 |
| 22 | 1 | 0 | -1.259137 | -1.735060 | -0.334956 |
| 23 | 6 | 0 | -4.793873 | -0.174187 | -0.096053 |
| 24 | 1 | 0 | -3.848306 | 1.696413 | 0.143294 |
| 25 | 6 | 0 | -4.665326 | -1.584828 | -0.262815 |
| 26 | 1 | 0 | -3.188041 | -3.173588 | -0.495818 |
| 27 | 1 | 0 | -5.769760 | 0.281200 | 0.009354 |
| 28 | 8 | 0 | -0.011576 | 0.245275 | -0.043116 |
| 29 | 7 | 0 | -5.775319 | -2.395455 | -0.338561 |
| 30 | 6 | 0 | -7.071240 | -1.921477 | -0.827955 |
| 31 | 1 | 0 | -7.126016 | -0.836605 | -0.810152 |
| 32 | 1 | 0 | -7.187675 | -2.229242 | -1.876763 |
| 33 | 6 | 0 | -5.693406 | -3.854490 | -0.288496 |
| 34 | 1 | 0 | -5.713040 | -4.276549 | -1.302692 |
| 35 | 1 | 0 | -4.766330 | -4.167406 | 0.187123 |
| 36 | 6 | 0 | 1.035355 | 9.525539 | -0.167262 |
| 37 | 1 | 0 | 1.207595 | 9.371388 | -1.238502 |
| 38 | 1 | 0 | 1.955466 | 9.300891 | 0.383940 |
| 39 | 6 | 0 | 2.343269 | 0.073464 | -0.075656 |
| 40 | 6 | 0 | 2.208206 | -1.333365 | 0.046857 |
| 41 | 6 | 0 | 3.658635 | 0.581445 | -0.237451 |
| 42 | 6 | 0 | 3.301891 | -2.171863 | 0.012608 |
| 43 | 1 | 0 | 1.225936 | -1.762447 | 0.194932 |
| 44 | 6 | 0 | 4.756024 | -0.250010 | -0.273653 |
| 45 | 1 | 0 | 3.824177 | 1.643777 | -0.371645 |
| 46 | 6 | 0 | 4.617546 | -1.660385 | -0.150723 |

| | | | | | |
|---|---|---|---|---|---|
| 47 | 1 | 0 | 3.134805 | -3.232334 | 0.149450 |
| 48 | 1 | 0 | 5.726155 | 0.196722 | -0.444592 |
| 49 | 7 | 0 | 5.716429 | -2.499070 | -0.168681 |
| 50 | 6 | 0 | 7.079938 | -1.972527 | -0.206950 |
| 51 | 1 | 0 | 7.124173 | -1.007791 | 0.295600 |
| 52 | 1 | 0 | 7.416415 | -1.827958 | -1.243907 |
| 53 | 6 | 0 | 5.624022 | -3.877328 | -0.656014 |
| 54 | 1 | 0 | 5.845373 | -3.902295 | -1.733355 |
| 55 | 1 | 0 | 4.617850 | -4.268631 | -0.530982 |
| 56 | 6 | 0 | 8.045359 | -2.884076 | 0.546322 |
| 57 | 1 | 0 | 9.069174 | -2.526979 | 0.432231 |
| 58 | 1 | 0 | 7.794805 | -2.933545 | 1.607413 |
| 59 | 6 | 0 | 6.566674 | -4.806787 | 0.097530 |
| 60 | 1 | 0 | 6.309067 | -4.865203 | 1.156501 |
| 61 | 1 | 0 | 6.546859 | -5.805908 | -0.338212 |
| 62 | 6 | 0 | -6.827747 | -4.432666 | 0.556743 |
| 63 | 1 | 0 | -6.812769 | -5.522084 | 0.515851 |
| 64 | 1 | 0 | -6.751547 | -4.105963 | 1.595274 |
| 65 | 6 | 0 | -8.222580 | -2.463789 | 0.010457 |
| 66 | 1 | 0 | -8.166818 | -2.114920 | 1.042972 |
| 67 | 1 | 0 | -9.180591 | -2.173613 | -0.421332 |
| 68 | 7 | 0 | -8.176491 | -3.972585 | 0.058167 |
| 69 | 7 | 0 | 7.987494 | -4.299570 | 0.026167 |
| 70 | 6 | 0 | -9.304433 | -4.546690 | 0.867749 |
| 71 | 1 | 0 | -10.244591 | -4.208576 | 0.434133 |
| 72 | 1 | 0 | -9.238279 | -5.632814 | 0.828137 |
| 73 | 1 | 0 | -9.208869 | -4.195691 | 1.894180 |
| 74 | 6 | 0 | 8.948187 | -5.207112 | 0.740365 |
| 75 | 1 | 0 | 8.882618 | -6.197629 | 0.292567 |
| 76 | 1 | 0 | 9.953581 | -4.805809 | 0.623648 |
| 77 | 1 | 0 | 8.673187 | -5.245932 | 1.793329 |
| 78 | 1 | 0 | 0.753882 | 10.564094 | 0.007391 |
| 79 | 8 | 0 | -0.058667 | 8.739052 | 0.302956 |
| 80 | 1 | 0 | -8.291839 | -4.320127 | -0.900150 |
| 81 | 1 | 0 | 8.269718 | -4.285430 | -0.960704 |

## 2,6-NMP-4-2MeOPh

### 2,6-NMP-4-2MeOPh_WATER_+2H+_S0

1167aaa_26_NMP+2H+_4oOMe_pirrilium_b3lyp631dp_PCMw.log

Standard orientation:

| Center Number | Atomic Number | Atomic Type | Coordinates (Angstroms) X | Y | Z |
|---|---|---|---|---|---|
| 1 | 6 | 0 | 1.178171 | 2.660020 | -0.143796 |
| 2 | 6 | 0 | 1.241781 | 1.283992 | -0.051756 |
| 3 | 6 | 0 | -1.134059 | 1.155565 | -0.045352 |
| 4 | 6 | 0 | -1.226831 | 2.529397 | -0.148603 |
| 5 | 6 | 0 | -0.064558 | 3.324626 | -0.199443 |
| 6 | 1 | 0 | 2.098889 | 3.224409 | -0.118861 |
| 7 | 1 | 0 | -2.200273 | 2.986879 | -0.217588 |
| 8 | 6 | 0 | -0.109212 | 4.788378 | -0.342743 |
| 9 | 6 | 0 | -1.135374 | 5.589932 | 0.233386 |
| 10 | 6 | 0 | 0.902246 | 5.436355 | -1.078746 |
| 11 | 6 | 0 | -1.117001 | 6.979388 | 0.057827 |
| 12 | 6 | 0 | 0.910332 | 6.813536 | -1.260743 |
| 13 | 1 | 0 | 1.673829 | 4.836595 | -1.549163 |
| 14 | 6 | 0 | -0.103778 | 7.581509 | -0.685717 |
| 15 | 1 | 0 | -1.889627 | 7.594608 | 0.500130 |
| 16 | 1 | 0 | 1.692562 | 7.280615 | -1.848781 |
| 17 | 6 | 0 | 2.438904 | 0.470641 | 0.028481 |
| 18 | 6 | 0 | 2.385967 | -0.869529 | 0.459143 |
| 19 | 6 | 0 | 3.701319 | 0.996197 | -0.320903 |
| 20 | 6 | 0 | 3.530636 | -1.643163 | 0.549148 |
| 21 | 1 | 0 | 1.439158 | -1.301024 | 0.762095 |
| 22 | 6 | 0 | 4.845313 | 0.226858 | -0.244509 |
| 23 | 1 | 0 | 3.789990 | 2.009470 | -0.696226 |

| | | | | | |
|---|---|---|---|---|---|
| 24 | 6 | 0 | 4.796623 | -1.119887 | 0.194124 |
| 25 | 1 | 0 | 3.435110 | -2.651299 | 0.930274 |
| 26 | 1 | 0 | 5.779257 | 0.667092 | -0.570010 |
| 27 | 8 | 0 | 0.089205 | 0.573397 | -0.000704 |
| 28 | 7 | 0 | 5.948643 | -1.896984 | 0.229686 |
| 29 | 6 | 0 | 7.244050 | -1.308271 | 0.571085 |
| 30 | 1 | 0 | 7.282300 | -0.257564 | 0.294009 |
| 31 | 1 | 0 | 7.410303 | -1.359327 | 1.658838 |
| 32 | 6 | 0 | 5.888625 | -3.318287 | 0.554990 |
| 33 | 1 | 0 | 5.933880 | -3.488524 | 1.641701 |
| 34 | 1 | 0 | 4.955840 | -3.748441 | 0.191442 |
| 35 | 6 | 0 | -2.234873 | 0.212733 | -0.003211 |
| 36 | 6 | 0 | -2.028434 | -1.166384 | -0.221355 |
| 37 | 6 | 0 | -3.551074 | 0.644066 | 0.253155 |
| 38 | 6 | 0 | -3.080461 | -2.061852 | -0.198137 |
| 39 | 1 | 0 | -1.033370 | -1.533458 | -0.443234 |
| 40 | 6 | 0 | -4.607875 | -0.248817 | 0.286050 |
| 41 | 1 | 0 | -3.755215 | 1.686276 | 0.471245 |
| 42 | 6 | 0 | -4.405943 | -1.629271 | 0.051260 |
| 43 | 1 | 0 | -2.873414 | -3.102033 | -0.416543 |
| 44 | 1 | 0 | -5.589970 | 0.133336 | 0.530558 |
| 45 | 7 | 0 | -5.466691 | -2.529964 | 0.023396 |
| 46 | 6 | 0 | -6.842323 | -2.056779 | 0.143627 |
| 47 | 1 | 0 | -6.950109 | -1.090869 | -0.349668 |
| 48 | 1 | 0 | -7.141207 | -1.930864 | 1.195975 |
| 49 | 6 | 0 | -5.300405 | -3.890377 | 0.538916 |
| 50 | 1 | 0 | -5.491943 | -3.914627 | 1.623288 |
| 51 | 1 | 0 | -4.282121 | -4.242075 | 0.388224 |
| 52 | 6 | 0 | -7.801932 | -3.003024 | -0.571053 |
| 53 | 1 | 0 | -8.835879 | -2.694602 | -0.413819 |
| 54 | 1 | 0 | -7.591797 | -3.040368 | -1.641318 |
| 55 | 6 | 0 | -6.225387 | -4.868582 | -0.172842 |
| 56 | 1 | 0 | -6.001464 | -4.927605 | -1.239397 |
| 57 | 1 | 0 | -6.156499 | -5.861952 | 0.271724 |
| 58 | 6 | 0 | 7.005648 | -4.082765 | -0.152344 |
| 59 | 1 | 0 | 7.022449 | -5.124388 | 0.170895 |
| 60 | 1 | 0 | 6.884552 | -4.039837 | -1.236061 |
| 61 | 6 | 0 | 8.376550 | -2.013689 | -0.162674 |
| 62 | 1 | 0 | 8.270651 | -1.919450 | -1.244801 |
| 63 | 1 | 0 | 9.346283 | -1.622154 | 0.147023 |
| 64 | 7 | 0 | 8.363522 | -3.492188 | 0.149305 |
| 65 | 7 | 0 | -7.662753 | -4.416442 | -0.058522 |
| 66 | 6 | 0 | 9.465512 | -4.227706 | -0.557008 |
| 67 | 1 | 0 | 10.417996 | -3.792712 | -0.257995 |
| 68 | 1 | 0 | 9.421629 | -5.277096 | -0.269695 |
| 69 | 1 | 0 | 9.320284 | -4.121242 | -1.631143 |
| 70 | 6 | 0 | -8.608849 | -5.360431 | -0.742153 |
| 71 | 1 | 0 | -8.487539 | -6.349569 | -0.302997 |
| 72 | 1 | 0 | -9.625556 | -5.001171 | -0.590068 |
| 73 | 1 | 0 | -8.368099 | -5.383206 | -1.804069 |
| 74 | 1 | 0 | -0.113063 | 8.659295 | -0.815061 |
| 75 | 8 | 0 | -2.079241 | 4.948347 | 0.965564 |
| 76 | 6 | 0 | -3.107335 | 5.712474 | 1.604635 |
| 77 | 1 | 0 | -2.682615 | 6.419058 | 2.324573 |
| 78 | 1 | 0 | -3.714473 | 6.251481 | 0.870568 |
| 79 | 1 | 0 | -3.727648 | 4.985967 | 2.128220 |
| 80 | 1 | 0 | -7.908777 | -4.411637 | 0.937354 |
| 81 | 1 | 0 | 8.531115 | -3.594023 | 1.156015 |

---------------------------------------------------------------------

**2,6-NMP-4-2MeOPh_WATER_+2H+_S1**

1167baa_26_NMP+2H+_4oOMe_pirrilium_TD_b3lyp631dp_PCMw_spopt.log

Standard orientation:

| Center Number | Atomic Number | Atomic Type | Coordinates (Angstroms) X | Y | Z |
|---|---|---|---|---|---|
| 1 | 6 | 0 | 1.206272 | 2.606290 | -0.128576 |
| 2 | 6 | 0 | 1.260772 | 1.239681 | -0.024379 |
| 3 | 6 | 0 | -1.117447 | 1.133903 | -0.002585 |
| 4 | 6 | 0 | -1.188003 | 2.506268 | -0.106582 |
| 5 | 6 | 0 | -0.028816 | 3.319953 | -0.182675 |
| 6 | 1 | 0 | 2.140096 | 3.151426 | -0.117507 |
| 7 | 1 | 0 | -2.162643 | 2.965707 | -0.155845 |

| | | | | | |
|---|---|---|---|---|---|
| 8 | 6 | 0 | -0.048394 | 4.772636 | -0.347649 |
| 9 | 6 | 0 | -1.100646 | 5.603772 | 0.143422 |
| 10 | 6 | 0 | 1.020502 | 5.418297 | -1.007918 |
| 11 | 6 | 0 | -1.055407 | 6.988964 | -0.037560 |
| 12 | 6 | 0 | 1.064209 | 6.797033 | -1.184882 |
| 13 | 1 | 0 | 1.819048 | 4.813322 | -1.424016 |
| 14 | 6 | 0 | 0.021306 | 7.584859 | -0.698289 |
| 15 | 1 | 0 | -1.854586 | 7.612620 | 0.342308 |
| 16 | 1 | 0 | 1.899791 | 7.248843 | -1.709449 |
| 17 | 6 | 0 | 2.461644 | 0.429981 | 0.087682 |
| 18 | 6 | 0 | 2.387817 | -0.928906 | 0.476618 |
| 19 | 6 | 0 | 3.747542 | 0.960498 | -0.182326 |
| 20 | 6 | 0 | 3.521448 | -1.706465 | 0.607609 |
| 21 | 1 | 0 | 1.424730 | -1.364164 | 0.710726 |
| 22 | 6 | 0 | 4.884371 | 0.193907 | -0.056473 |
| 23 | 1 | 0 | 3.857190 | 1.981723 | -0.527676 |
| 24 | 6 | 0 | 4.811939 | -1.170406 | 0.347886 |
| 25 | 1 | 0 | 3.405032 | -2.724366 | 0.955282 |
| 26 | 1 | 0 | 5.833117 | 0.642553 | -0.319345 |
| 27 | 8 | 0 | 0.104532 | 0.493342 | 0.030111 |
| 28 | 7 | 0 | 5.949032 | -1.938760 | 0.462688 |
| 29 | 6 | 0 | 7.277445 | -1.374657 | 0.696088 |
| 30 | 1 | 0 | 7.287913 | -0.301422 | 0.529041 |
| 31 | 1 | 0 | 7.550545 | -1.535223 | 1.749000 |
| 32 | 6 | 0 | 5.920152 | -3.385668 | 0.661044 |
| 33 | 1 | 0 | 6.096568 | -3.629323 | 1.717688 |
| 34 | 1 | 0 | 4.951481 | -3.793439 | 0.381001 |
| 35 | 6 | 0 | -2.239603 | 0.219343 | 0.040605 |
| 36 | 6 | 0 | -2.050890 | -1.176642 | -0.108770 |
| 37 | 6 | 0 | -3.566560 | 0.675062 | 0.234937 |
| 38 | 6 | 0 | -3.113718 | -2.057434 | -0.079185 |
| 39 | 1 | 0 | -1.054666 | -1.564619 | -0.279603 |
| 40 | 6 | 0 | -4.633466 | -0.198771 | 0.269357 |
| 41 | 1 | 0 | -3.764780 | 1.727856 | 0.398856 |
| 42 | 6 | 0 | -4.443721 | -1.597984 | 0.110058 |
| 43 | 1 | 0 | -2.911054 | -3.108158 | -0.242608 |
| 44 | 1 | 0 | -5.617024 | 0.205831 | 0.467300 |
| 45 | 7 | 0 | -5.512606 | -2.477987 | 0.118224 |
| 46 | 6 | 0 | -6.893676 | -2.004408 | 0.157279 |
| 47 | 1 | 0 | -6.972023 | -1.031354 | -0.324995 |
| 48 | 1 | 0 | -7.244374 | -1.895594 | 1.194465 |
| 49 | 6 | 0 | -5.371916 | -3.855796 | 0.591099 |
| 50 | 1 | 0 | -5.611058 | -3.902261 | 1.664686 |
| 51 | 1 | 0 | -4.348320 | -4.204484 | 0.480628 |
| 52 | 6 | 0 | -7.817204 | -2.938550 | -0.621241 |
| 53 | 1 | 0 | -8.855454 | -2.625379 | -0.508814 |
| 54 | 1 | 0 | -7.556929 | -2.956935 | -1.680779 |
| 55 | 6 | 0 | -6.267148 | -4.812477 | -0.186886 |
| 56 | 1 | 0 | -5.994284 | -4.848343 | -1.243022 |
| 57 | 1 | 0 | -6.216482 | -5.815311 | 0.237810 |
| 58 | 6 | 0 | 6.950590 | -4.079103 | -0.231573 |
| 59 | 1 | 0 | 6.997490 | -5.143369 | 0.002081 |
| 60 | 1 | 0 | 6.705473 | -3.950034 | -1.286787 |
| 61 | 6 | 0 | 8.320455 | -2.007879 | -0.217479 |
| 62 | 1 | 0 | 8.101494 | -1.815982 | -1.268955 |
| 63 | 1 | 0 | 9.317324 | -1.636514 | 0.022464 |
| 64 | 7 | 0 | 8.335673 | -3.508075 | -0.038889 |
| 65 | 7 | 0 | -7.706796 | -4.359595 | -0.125245 |
| 66 | 6 | 0 | 9.340368 | -4.170864 | -0.939582 |
| 67 | 1 | 0 | 10.323584 | -3.754932 | -0.724455 |
| 68 | 1 | 0 | 9.329189 | -5.240996 | -0.738396 |
| 69 | 1 | 0 | 9.060112 | -3.974231 | -1.973664 |
| 70 | 6 | 0 | -8.626790 | -5.290998 | -0.861841 |
| 71 | 1 | 0 | -8.527055 | -6.285696 | -0.429810 |
| 72 | 1 | 0 | -9.647496 | -4.929335 | -0.745955 |
| 73 | 1 | 0 | -8.342635 | -5.302037 | -1.913127 |
| 74 | 1 | 0 | 0.035109 | 8.662227 | -0.829881 |
| 75 | 8 | 0 | -2.112591 | 4.978883 | 0.811549 |
| 76 | 6 | 0 | -3.160508 | 5.766665 | 1.377054 |
| 77 | 1 | 0 | -2.770538 | 6.475579 | 2.115338 |
| 78 | 1 | 0 | -3.713476 | 6.311237 | 0.604122 |
| 79 | 1 | 0 | -3.828014 | 5.059672 | 1.869536 |
| 80 | 1 | 0 | -7.996308 | -4.370451 | 0.859174 |
| 81 | 1 | 0 | 8.623739 | -3.702128 | 0.926728 |

---------------------------------------------------------------------

**2,6-NMP-4-2MeOPh_WATER_+2H+_TR**
1167caa_26_NMP+2H+_4oOMe_pirrilium_b3lyp631dp_PCMw_TRIPLET.log

Standard orientation:

| Center Number | Atomic Number | Atomic Type | Coordinates (Angstroms) X | Y | Z |
|---|---|---|---|---|---|
| 1 | 6 | 0 | 1.197126 | 2.584056 | -0.158370 |
| 2 | 6 | 0 | 1.256705 | 1.207932 | -0.065527 |
| 3 | 6 | 0 | -1.130944 | 1.112172 | -0.049769 |
| 4 | 6 | 0 | -1.191393 | 2.489071 | -0.137183 |
| 5 | 6 | 0 | -0.028000 | 3.297100 | -0.212030 |
| 6 | 1 | 0 | 2.129682 | 3.132421 | -0.149310 |
| 7 | 1 | 0 | -2.162616 | 2.958645 | -0.165460 |
| 8 | 6 | 0 | -0.048010 | 4.757293 | -0.374022 |
| 9 | 6 | 0 | -1.065764 | 5.585626 | 0.179954 |
| 10 | 6 | 0 | 0.982746 | 5.397090 | -1.090366 |
| 11 | 6 | 0 | -1.027040 | 6.972103 | 0.004218 |
| 12 | 6 | 0 | 1.024465 | 6.778642 | -1.260809 |
| 13 | 1 | 0 | 1.753851 | 4.787789 | -1.551095 |
| 14 | 6 | 0 | 0.014217 | 7.566894 | -0.712457 |
| 15 | 1 | 0 | -1.801577 | 7.596647 | 0.431156 |
| 16 | 1 | 0 | 1.831940 | 7.230129 | -1.828066 |
| 17 | 6 | 0 | 2.449846 | 0.411110 | 0.043940 |
| 18 | 6 | 0 | 2.372713 | -0.990272 | 0.253742 |
| 19 | 6 | 0 | 3.750445 | 0.978205 | -0.048433 |
| 20 | 6 | 0 | 3.504477 | -1.769425 | 0.369728 |
| 21 | 1 | 0 | 1.403102 | -1.459564 | 0.354761 |
| 22 | 6 | 0 | 4.883376 | 0.209002 | 0.069024 |
| 23 | 1 | 0 | 3.875085 | 2.037452 | -0.237478 |
| 24 | 6 | 0 | 4.802506 | -1.197651 | 0.283167 |
| 25 | 1 | 0 | 3.379194 | -2.824809 | 0.572838 |
| 26 | 1 | 0 | 5.843475 | 0.694297 | -0.048470 |
| 27 | 8 | 0 | 0.091557 | 0.462672 | -0.041367 |
| 28 | 7 | 0 | 5.939986 | -1.969222 | 0.381884 |
| 29 | 6 | 0 | 7.219323 | -1.437854 | 0.853683 |
| 30 | 1 | 0 | 7.241310 | -0.353220 | 0.793781 |
| 31 | 1 | 0 | 7.347130 | -1.700902 | 1.913525 |
| 32 | 6 | 0 | 5.903666 | -3.430166 | 0.389350 |
| 33 | 1 | 0 | 5.938358 | -3.813721 | 1.418687 |
| 34 | 1 | 0 | 4.986411 | -3.791144 | -0.071225 |
| 35 | 6 | 0 | -2.256487 | 0.217650 | 0.004353 |
| 36 | 6 | 0 | -2.080081 | -1.187244 | -0.100316 |
| 37 | 6 | 0 | -3.586280 | 0.688813 | 0.171734 |
| 38 | 6 | 0 | -3.147353 | -2.056778 | -0.046688 |
| 39 | 1 | 0 | -1.086480 | -1.588197 | -0.251738 |
| 40 | 6 | 0 | -4.657084 | -0.173622 | 0.227548 |
| 41 | 1 | 0 | -3.780005 | 1.747391 | 0.296359 |
| 42 | 6 | 0 | -4.477522 | -1.581959 | 0.120444 |
| 43 | 1 | 0 | -2.949943 | -3.113302 | -0.172298 |
| 44 | 1 | 0 | -5.638460 | 0.245706 | 0.403000 |
| 45 | 7 | 0 | -5.549563 | -2.450144 | 0.159757 |
| 46 | 6 | 0 | -6.931508 | -1.972552 | 0.192505 |
| 47 | 1 | 0 | -7.006621 | -0.999234 | -0.288464 |
| 48 | 1 | 0 | -7.281158 | -1.864875 | 1.229110 |
| 49 | 6 | 0 | -5.416457 | -3.831292 | 0.629277 |
| 50 | 1 | 0 | -5.658070 | -3.874709 | 1.701383 |
| 51 | 1 | 0 | -4.394850 | -4.185177 | 0.521058 |
| 52 | 6 | 0 | -7.854932 | -2.903580 | -0.589669 |
| 53 | 1 | 0 | -8.891896 | -2.585081 | -0.481160 |
| 54 | 1 | 0 | -7.591192 | -2.922808 | -1.648365 |
| 55 | 6 | 0 | -6.314371 | -4.782597 | -0.152290 |
| 56 | 1 | 0 | -6.039015 | -4.818491 | -1.207787 |
| 57 | 1 | 0 | -6.267048 | -5.785983 | 0.271285 |
| 58 | 6 | 0 | 7.054274 | -4.004910 | -0.435560 |
| 59 | 1 | 0 | 7.073130 | -5.092101 | -0.355184 |
| 60 | 1 | 0 | 6.969255 | -3.718860 | -1.485253 |
| 61 | 6 | 0 | 8.386768 | -1.974954 | 0.034074 |
| 62 | 1 | 0 | 8.316257 | -1.668212 | -1.010901 |
| 63 | 1 | 0 | 9.335931 | -1.637489 | 0.450951 |
| 64 | 7 | 0 | 8.388579 | -3.485538 | 0.043660 |
| 65 | 7 | 0 | -7.752424 | -4.325004 | -0.093525 |
| 66 | 6 | 0 | 9.532220 | -4.056703 | -0.745628 |

| | | | | | |
|---|---|---|---|---|---|
| 67 | 1 | 0 | 10.462838 | -3.673542 | -0.329400 |
| 68 | 1 | 0 | 9.498628 | -5.141948 | -0.662278 |
| 69 | 1 | 0 | 9.424324 | -3.750882 | -1.785294 |
| 70 | 6 | 0 | -8.674004 | -5.252868 | -0.833107 |
| 71 | 1 | 0 | -8.579497 | -6.247763 | -0.400405 |
| 72 | 1 | 0 | -9.693516 | -4.887076 | -0.720092 |
| 73 | 1 | 0 | -8.386870 | -5.265269 | -1.883531 |
| 74 | 1 | 0 | 0.025160 | 8.645061 | -0.838983 |
| 75 | 8 | 0 | -2.041620 | 4.954696 | 0.897509 |
| 76 | 6 | 0 | -3.061922 | 5.739151 | 1.514698 |
| 77 | 1 | 0 | -2.638760 | 6.445024 | 2.237636 |
| 78 | 1 | 0 | -3.650255 | 6.287581 | 0.770998 |
| 79 | 1 | 0 | -3.706192 | 5.030083 | 2.034557 |
| 80 | 1 | 0 | -8.045256 | -4.335565 | 0.889948 |
| 81 | 1 | 0 | 8.514341 | -3.792804 | 1.014338 |

---------------------------------------------------------------------

## 2,4-DMA-6-Me

### 2,4-DMA-6-Me_WATER_S0

1051aaa_bis_NMe2_pirrilium_Me_b3lyp631dp_PCMw.log

Standard orientation:

| Center Number | Atomic Number | Atomic Type | Coordinates (Angstroms) X | Y | Z |
|---|---|---|---|---|---|
| 1 | 6 | 0 | -0.056515 | 0.308011 | 0.019617 |
| 2 | 6 | 0 | 1.181059 | 0.930564 | 0.044868 |
| 3 | 6 | 0 | 0.110039 | 3.047173 | 0.112306 |
| 4 | 6 | 0 | -1.119631 | 2.468430 | 0.088089 |
| 5 | 6 | 0 | -1.255643 | 1.044488 | 0.043916 |
| 6 | 1 | 0 | -0.080486 | -0.768514 | -0.055556 |
| 7 | 1 | 0 | -1.982928 | 3.116950 | 0.134566 |
| 8 | 6 | 0 | -2.556813 | 0.405315 | 0.023055 |
| 9 | 6 | 0 | -3.735035 | 1.124403 | -0.295137 |
| 10 | 6 | 0 | -2.718210 | -0.970898 | 0.319526 |
| 11 | 6 | 0 | -4.976060 | 0.524053 | -0.328249 |
| 12 | 1 | 0 | -3.678115 | 2.174476 | -0.558887 |
| 13 | 6 | 0 | -3.951847 | -1.585491 | 0.305977 |
| 14 | 1 | 0 | -1.860782 | -1.570684 | 0.604161 |
| 15 | 6 | 0 | -5.131780 | -0.858470 | -0.024395 |
| 16 | 1 | 0 | -5.835597 | 1.123218 | -0.598222 |
| 17 | 1 | 0 | -4.012068 | -2.634997 | 0.562136 |
| 18 | 6 | 0 | 2.478781 | 0.299404 | 0.014502 |
| 19 | 6 | 0 | 3.664801 | 1.069537 | -0.028165 |
| 20 | 6 | 0 | 2.621205 | -1.107960 | 0.028371 |
| 21 | 6 | 0 | 4.911127 | 0.479894 | -0.059229 |
| 22 | 1 | 0 | 3.598802 | 2.151029 | -0.040300 |
| 23 | 6 | 0 | 3.860614 | -1.711125 | -0.001853 |
| 24 | 1 | 0 | 1.747819 | -1.749025 | 0.070927 |
| 25 | 6 | 0 | 5.055602 | -0.936804 | -0.048388 |
| 26 | 1 | 0 | 5.785273 | 1.116436 | -0.094422 |
| 27 | 1 | 0 | 3.911086 | -2.791738 | 0.013629 |
| 28 | 8 | 0 | 1.232802 | 2.288332 | 0.092186 |
| 29 | 7 | 0 | -6.353458 | -1.458638 | -0.046859 |
| 30 | 7 | 0 | 6.283187 | -1.526498 | -0.080312 |
| 31 | 6 | 0 | 6.404627 | -2.981922 | -0.073466 |
| 32 | 1 | 0 | 7.458922 | -3.250381 | -0.110976 |
| 33 | 1 | 0 | 5.973401 | -3.416994 | 0.835609 |
| 34 | 1 | 0 | 5.907084 | -3.429973 | -0.941278 |
| 35 | 6 | 0 | 7.494132 | -0.710836 | -0.120709 |
| 36 | 1 | 0 | 8.363098 | -1.366353 | -0.135904 |
| 37 | 1 | 0 | 7.524987 | -0.081545 | -1.017757 |
| 38 | 1 | 0 | 7.570847 | -0.062399 | 0.759697 |
| 39 | 6 | 0 | -7.550517 | -0.686377 | -0.370412 |
| 40 | 1 | 0 | -7.495239 | -0.265051 | -1.380774 |
| 41 | 1 | 0 | -8.418844 | -1.341050 | -0.322632 |
| 42 | 1 | 0 | -7.701460 | 0.134731 | 0.339590 |
| 43 | 6 | 0 | -6.484976 | -2.880964 | 0.259473 |
| 44 | 1 | 0 | -6.156096 | -3.104265 | 1.281065 |
| 45 | 1 | 0 | -7.531061 | -3.168117 | 0.168898 |

| | | | | | |
|---|---|---|---|---|---|
| 46 | 1 | 0 | -5.900577 | -3.495296 | -0.434713 |
| 47 | 6 | 0 | 0.412629 | 4.504035 | 0.174341 |
| 48 | 1 | 0 | 0.996422 | 4.732647 | 1.071865 |
| 49 | 1 | 0 | 1.008927 | 4.804361 | -0.693342 |
| 50 | 1 | 0 | -0.509482 | 5.085059 | 0.191112 |

---------------------------------------------------------------------

**2,4-DMA-6-Me_WATER_S1**

1051aaa_bis_NMe2_pirrilium_Me_TD_b3lyp631dp_PCMw_spopt.log

Standard orientation:

| Center Number | Atomic Number | Atomic Type | Coordinates (Angstroms) X | Y | Z |
|---|---|---|---|---|---|
| 1 | 6 | 0 | 0.042567 | 0.332759 | -0.016793 |
| 2 | 6 | 0 | -1.176723 | 0.948887 | 0.009544 |
| 3 | 6 | 0 | -0.120219 | 3.078217 | -0.012554 |
| 4 | 6 | 0 | 1.107405 | 2.492727 | -0.012855 |
| 5 | 6 | 0 | 1.269390 | 1.073329 | -0.020398 |
| 6 | 1 | 0 | 0.059843 | -0.748133 | -0.014666 |
| 7 | 1 | 0 | 1.965185 | 3.152686 | -0.039677 |
| 8 | 6 | 0 | 2.562436 | 0.418834 | -0.017075 |
| 9 | 6 | 0 | 3.756068 | 1.119657 | 0.301541 |
| 10 | 6 | 0 | 2.716456 | -0.957562 | -0.333922 |
| 11 | 6 | 0 | 4.995151 | 0.509473 | 0.300096 |
| 12 | 1 | 0 | 3.709360 | 2.166340 | 0.581510 |
| 13 | 6 | 0 | 3.949083 | -1.582060 | -0.340132 |
| 14 | 1 | 0 | 1.852295 | -1.551215 | -0.611018 |
| 15 | 6 | 0 | 5.137674 | -0.868998 | -0.023656 |
| 16 | 1 | 0 | 5.863681 | 1.100355 | 0.561797 |
| 17 | 1 | 0 | 3.996047 | -2.631625 | -0.601137 |
| 18 | 6 | 0 | -2.487364 | 0.297432 | 0.026599 |
| 19 | 6 | 0 | -3.661350 | 1.039569 | -0.242152 |
| 20 | 6 | 0 | -2.630802 | -1.081484 | 0.310225 |
| 21 | 6 | 0 | -4.906979 | 0.443443 | -0.243614 |
| 22 | 1 | 0 | -3.582628 | 2.097767 | -0.456916 |
| 23 | 6 | 0 | -3.866731 | -1.692501 | 0.308987 |
| 24 | 1 | 0 | -1.761898 | -1.682931 | 0.550845 |
| 25 | 6 | 0 | -5.051047 | -0.947395 | 0.029180 |
| 26 | 1 | 0 | -5.775446 | 1.052175 | -0.456754 |
| 27 | 1 | 0 | -3.925199 | -2.748707 | 0.534751 |
| 28 | 8 | 0 | -1.273479 | 2.324747 | -0.000221 |
| 29 | 7 | 0 | 6.364445 | -1.480502 | -0.026944 |
| 30 | 7 | 0 | -6.277300 | -1.545999 | 0.029517 |
| 31 | 6 | 0 | -6.411486 | -2.971794 | 0.321030 |
| 32 | 1 | 0 | -7.462625 | -3.246195 | 0.273806 |
| 33 | 1 | 0 | -5.860098 | -3.574601 | -0.408595 |
| 34 | 1 | 0 | -6.035132 | -3.204203 | 1.323030 |
| 35 | 6 | 0 | -7.482347 | -0.771327 | -0.258627 |
| 36 | 1 | 0 | -8.346015 | -1.430909 | -0.221673 |
| 37 | 1 | 0 | -7.620328 | 0.027347 | 0.478441 |
| 38 | 1 | 0 | -7.428963 | -0.321651 | -1.255808 |
| 39 | 6 | 0 | 7.566631 | -0.723684 | 0.299494 |
| 40 | 1 | 0 | 7.522629 | -0.317046 | 1.317453 |
| 41 | 1 | 0 | 8.431130 | -1.382017 | 0.233409 |
| 42 | 1 | 0 | 7.717188 | 0.110995 | -0.396110 |
| 43 | 6 | 0 | 6.480370 | -2.895772 | -0.354845 |
| 44 | 1 | 0 | 6.121605 | -3.104077 | -1.370474 |
| 45 | 1 | 0 | 7.526565 | -3.191092 | -0.296229 |
| 46 | 1 | 0 | 5.908221 | -3.518387 | 0.344022 |
| 47 | 6 | 0 | -0.418918 | 4.536936 | -0.028366 |
| 48 | 1 | 0 | -1.012880 | 4.801593 | -0.911044 |
| 49 | 1 | 0 | -1.005006 | 4.821733 | 0.853276 |
| 50 | 1 | 0 | 0.504703 | 5.117215 | -0.038725 |

---------------------------------------------------------------------

**2,4-DMA-6-Me_VACUO_TR**

1051dba_bis_NMe2_pirrilium_Me_b3lyp631dp_TRIPLET.log

Standard orientation:

| Center Number | Atomic Number | Atomic Type | Coordinates (Angstroms) X | Y | Z |
|---|---|---|---|---|---|
| 1 | 6 | 0 | 0.051304 | 0.333819 | -0.054969 |

| | | | | | |
|---|---|---|---|---|---|
| 2 | 6 | 0 | -1.186541 | 0.948755 | -0.042695 |
| 3 | 6 | 0 | -0.112743 | 3.081159 | -0.092676 |
| 4 | 6 | 0 | 1.107699 | 2.500555 | -0.083692 |
| 5 | 6 | 0 | 1.261928 | 1.066059 | -0.068238 |
| 6 | 1 | 0 | 0.074092 | -0.746866 | -0.015619 |
| 7 | 1 | 0 | 1.969695 | 3.153460 | -0.130917 |
| 8 | 6 | 0 | 2.554332 | 0.412181 | -0.037930 |
| 9 | 6 | 0 | 3.735339 | 1.113193 | 0.332480 |
| 10 | 6 | 0 | 2.723422 | -0.959068 | -0.379834 |
| 11 | 6 | 0 | 4.972797 | 0.507849 | 0.360580 |
| 12 | 1 | 0 | 3.669871 | 2.152059 | 0.634058 |
| 13 | 6 | 0 | 3.953663 | -1.578376 | -0.355702 |
| 14 | 1 | 0 | 1.870264 | -1.542097 | -0.707907 |
| 15 | 6 | 0 | 5.128438 | -0.864840 | 0.015482 |
| 16 | 1 | 0 | 5.830090 | 1.094451 | 0.665171 |
| 17 | 1 | 0 | 4.015936 | -2.620433 | -0.642042 |
| 18 | 6 | 0 | -2.472031 | 0.295832 | -0.010150 |
| 19 | 6 | 0 | -3.668282 | 1.060737 | -0.036489 |
| 20 | 6 | 0 | -2.612011 | -1.116941 | 0.048324 |
| 21 | 6 | 0 | -4.910193 | 0.464851 | -0.006967 |
| 22 | 1 | 0 | -3.599732 | 2.139921 | -0.080942 |
| 23 | 6 | 0 | -3.846748 | -1.722635 | 0.077827 |
| 24 | 1 | 0 | -1.734412 | -1.752678 | 0.074656 |
| 25 | 6 | 0 | -5.046197 | -0.951268 | 0.051084 |
| 26 | 1 | 0 | -5.788667 | 1.096618 | -0.029016 |
| 27 | 1 | 0 | -3.894014 | -2.802743 | 0.124515 |
| 28 | 8 | 0 | -1.270972 | 2.328855 | -0.066517 |
| 29 | 7 | 0 | 6.356915 | -1.472467 | 0.041001 |
| 30 | 7 | 0 | -6.275724 | -1.548293 | 0.080798 |
| 31 | 6 | 0 | -6.394512 | -3.003731 | 0.138921 |
| 32 | 1 | 0 | -7.448186 | -3.276088 | 0.150897 |
| 33 | 1 | 0 | -5.928254 | -3.474188 | -0.733881 |
| 34 | 1 | 0 | -5.926050 | -3.403574 | 1.045201 |
| 35 | 6 | 0 | -7.494113 | -0.740752 | 0.055832 |
| 36 | 1 | 0 | -8.359906 | -1.399399 | 0.089507 |
| 37 | 1 | 0 | -7.540256 | -0.067707 | 0.919059 |
| 38 | 1 | 0 | -7.553084 | -0.141644 | -0.859566 |
| 39 | 6 | 0 | 7.552523 | -0.715613 | 0.404480 |
| 40 | 1 | 0 | 7.486508 | -0.338176 | 1.431336 |
| 41 | 1 | 0 | 8.422500 | -1.365949 | 0.334567 |
| 42 | 1 | 0 | 7.702837 | 0.133299 | -0.271393 |
| 43 | 6 | 0 | 6.491332 | -2.887166 | -0.295848 |
| 44 | 1 | 0 | 6.184560 | -3.079627 | -1.330420 |
| 45 | 1 | 0 | 7.532911 | -3.183781 | -0.187196 |
| 46 | 1 | 0 | 5.885036 | -3.510779 | 0.370387 |
| 47 | 6 | 0 | -0.416485 | 4.539265 | -0.139360 |
| 48 | 1 | 0 | -1.007867 | 4.783105 | -1.029330 |
| 49 | 1 | 0 | -1.005886 | 4.839210 | 0.734631 |
| 50 | 1 | 0 | 0.503908 | 5.124877 | -0.158985 |

---------------------------------------------------------------------

## 2,4-NMP-6-Me

### 2,4-NMP-6-Me_water_+2H+_S0

1003aaa_bis_PIPERAZIN_pirrilium_6Me_+2H+_b3lyp631dp_PCMw.log

Standard orientation:

| Center Number | Atomic Number | Atomic Type | Coordinates (Angstroms) X | Y | Z |
|---|---|---|---|---|---|
| 1 | 6 | 0 | 1.106977 | 3.218116 | -0.008113 |
| 2 | 6 | 0 | -0.126634 | 3.797398 | -0.026190 |
| 3 | 6 | 0 | -1.185941 | 1.679761 | -0.133014 |
| 4 | 6 | 0 | 0.049442 | 1.055392 | -0.106384 |
| 5 | 6 | 0 | 1.240912 | 1.799897 | -0.046782 |
| 6 | 1 | 0 | 1.972134 | 3.865971 | 0.011465 |
| 7 | 1 | 0 | 0.076047 | -0.023792 | -0.077596 |
| 8 | 6 | 0 | 2.546636 | 1.156215 | -0.006646 |
| 9 | 6 | 0 | 2.730604 | -0.173868 | -0.449682 |
| 10 | 6 | 0 | 3.684254 | 1.827542 | 0.488174 |

| | | | | | |
|---|---|---|---|---|---|
| 11 | 6 | 0 | 3.964095 | -0.791910 | -0.400452 |
| 12 | 1 | 0 | 1.904172 | -0.722562 | -0.887579 |
| 13 | 6 | 0 | 4.921926 | 1.211563 | 0.558748 |
| 14 | 1 | 0 | 3.597785 | 2.834205 | 0.881278 |
| 15 | 6 | 0 | 5.100222 | -0.118901 | 0.113418 |
| 16 | 1 | 0 | 4.054306 | -1.793603 | -0.801865 |
| 17 | 1 | 0 | 5.742058 | 1.762564 | 0.999791 |
| 18 | 8 | 0 | -1.240651 | 3.032803 | -0.092240 |
| 19 | 7 | 0 | 6.343486 | -0.741514 | 0.137061 |
| 20 | 6 | 0 | 6.449017 | -2.167981 | 0.452982 |
| 21 | 1 | 0 | 5.573542 | -2.710480 | 0.103121 |
| 22 | 1 | 0 | 6.501878 | -2.310991 | 1.543470 |
| 23 | 6 | 0 | 7.534677 | 0.001645 | 0.537272 |
| 24 | 1 | 0 | 7.650297 | 0.028601 | 1.631902 |
| 25 | 1 | 0 | 7.468190 | 1.031304 | 0.185757 |
| 26 | 6 | 0 | -2.484663 | 1.041834 | -0.174825 |
| 27 | 6 | 0 | -3.666038 | 1.777087 | 0.065279 |
| 28 | 6 | 0 | -2.619004 | -0.333016 | -0.455039 |
| 29 | 6 | 0 | -4.906378 | 1.170894 | 0.046716 |
| 30 | 1 | 0 | -3.603968 | 2.834484 | 0.293296 |
| 31 | 6 | 0 | -3.858625 | -0.944902 | -0.485080 |
| 32 | 1 | 0 | -1.748840 | -0.935252 | -0.690677 |
| 33 | 6 | 0 | -5.043772 | -0.214683 | -0.220994 |
| 34 | 1 | 0 | -5.772250 | 1.777278 | 0.279009 |
| 35 | 1 | 0 | -3.899596 | -1.993235 | -0.748544 |
| 36 | 7 | 0 | -6.287113 | -0.830224 | -0.187863 |
| 37 | 6 | 0 | -6.421079 | -2.273524 | -0.361161 |
| 38 | 1 | 0 | -5.544919 | -2.784293 | 0.035744 |
| 39 | 1 | 0 | -6.514509 | -2.546209 | -1.423257 |
| 40 | 6 | 0 | -7.504463 | -0.111958 | -0.567169 |
| 41 | 1 | 0 | -7.698030 | -0.235543 | -1.643963 |
| 42 | 1 | 0 | -7.403981 | 0.954898 | -0.383880 |
| 43 | 6 | 0 | -7.609376 | -2.807544 | 0.433963 |
| 44 | 1 | 0 | -7.762380 | -3.868030 | 0.230688 |
| 45 | 1 | 0 | -7.462695 | -2.659242 | 1.504981 |
| 46 | 6 | 0 | -8.704838 | -0.594533 | 0.236980 |
| 47 | 1 | 0 | -8.572200 | -0.409719 | 1.304291 |
| 48 | 1 | 0 | -9.620977 | -0.113111 | -0.106614 |
| 49 | 7 | 0 | -8.886255 | -2.085500 | 0.075174 |
| 50 | 6 | 0 | -10.060461 | -2.600215 | 0.855934 |
| 51 | 1 | 0 | -10.950044 | -2.057332 | 0.540318 |
| 52 | 1 | 0 | -10.173046 | -3.663725 | 0.650081 |
| 53 | 1 | 0 | -9.870125 | -2.435398 | 1.915788 |
| 54 | 6 | 0 | 7.666861 | -2.794779 | -0.212379 |
| 55 | 1 | 0 | 7.782333 | -3.835968 | 0.090947 |
| 56 | 1 | 0 | 7.604226 | -2.734158 | -1.300228 |
| 57 | 6 | 0 | 8.781377 | -0.586802 | -0.116335 |
| 58 | 1 | 0 | 8.731784 | -0.502567 | -1.203385 |
| 59 | 1 | 0 | 9.679222 | -0.086259 | 0.247138 |
| 60 | 7 | 0 | 8.923889 | -2.059015 | 0.189523 |
| 61 | 6 | 0 | 10.152987 | -2.644810 | -0.443114 |
| 62 | 1 | 0 | 11.022325 | -2.101755 | -0.075180 |
| 63 | 1 | 0 | 10.218305 | -3.695862 | -0.165981 |
| 64 | 1 | 0 | 10.068326 | -2.540814 | -1.523863 |
| 65 | 6 | 0 | -0.433637 | 5.252902 | 0.005813 |
| 66 | 1 | 0 | -0.994024 | 5.541684 | -0.889111 |
| 67 | 1 | 0 | -1.054880 | 5.487698 | 0.876173 |
| 68 | 1 | 0 | 0.486053 | 5.835461 | 0.055987 |
| 69 | 1 | 0 | 9.030443 | -2.159152 | 1.204793 |
| 70 | 1 | 0 | -9.080254 | -2.268672 | -0.915159 |

---------------------------------------------------------------------

**2,4-NMP-6-Me_water_+2H+_S1**
1003baa_bis_PIPERAZIN_pirrilium_6Me_+2H+_TD_b3lyp631dp_PCMw.log

Standard orientation:

| Center Number | Atomic Number | Atomic Type | Coordinates (Angstroms) X | Y | Z |
|---|---|---|---|---|---|
| 1 | 6 | 0 | 1.035411 | 3.194340 | 0.098071 |
| 2 | 6 | 0 | -0.202421 | 3.760651 | 0.063560 |
| 3 | 6 | 0 | -1.214144 | 1.623341 | -0.142563 |
| 4 | 6 | 0 | 0.011382 | 1.024348 | -0.113218 |
| 5 | 6 | 0 | 1.224192 | 1.782396 | 0.016069 |

| | | | | | |
|---|---|---|---|---|---|
| 6 | 1 | 0 | 1.879506 | 3.868484 | 0.165748 |
| 7 | 1 | 0 | 0.047125 | -0.055053 | -0.154815 |
| 8 | 6 | 0 | 2.524427 | 1.149429 | 0.067392 |
| 9 | 6 | 0 | 2.720303 | -0.210795 | -0.296548 |
| 10 | 6 | 0 | 3.683443 | 1.854933 | 0.482550 |
| 11 | 6 | 0 | 3.964134 | -0.810321 | -0.256182 |
| 12 | 1 | 0 | 1.888924 | -0.803394 | -0.660588 |
| 13 | 6 | 0 | 4.931141 | 1.259148 | 0.533186 |
| 14 | 1 | 0 | 3.602399 | 2.883829 | 0.814212 |
| 15 | 6 | 0 | 5.111786 | -0.094378 | 0.161484 |
| 16 | 1 | 0 | 4.052484 | -1.836530 | -0.593320 |
| 17 | 1 | 0 | 5.763194 | 1.845488 | 0.902770 |
| 18 | 8 | 0 | -1.336430 | 2.991970 | -0.047172 |
| 19 | 7 | 0 | 6.369764 | -0.701448 | 0.165568 |
| 20 | 6 | 0 | 6.517194 | -2.089946 | 0.601254 |
| 21 | 1 | 0 | 5.626621 | -2.667520 | 0.361647 |
| 22 | 1 | 0 | 6.643476 | -2.135277 | 1.695519 |
| 23 | 6 | 0 | 7.569590 | 0.088771 | 0.411494 |
| 24 | 1 | 0 | 7.760699 | 0.216482 | 1.488967 |
| 25 | 1 | 0 | 7.457379 | 1.081699 | -0.024462 |
| 26 | 6 | 0 | -2.516354 | 0.958996 | -0.254099 |
| 27 | 6 | 0 | -3.711736 | 1.695161 | -0.079732 |
| 28 | 6 | 0 | -2.629473 | -0.422007 | -0.541125 |
| 29 | 6 | 0 | -4.949100 | 1.091059 | -0.172352 |
| 30 | 1 | 0 | -3.658147 | 2.751453 | 0.148901 |
| 31 | 6 | 0 | -3.858263 | -1.039883 | -0.628713 |
| 32 | 1 | 0 | -1.744281 | -1.019013 | -0.724363 |
| 33 | 6 | 0 | -5.064240 | -0.301023 | -0.446028 |
| 34 | 1 | 0 | -5.828151 | 1.693704 | 0.012876 |
| 35 | 1 | 0 | -3.887293 | -2.088668 | -0.891612 |
| 36 | 7 | 0 | -6.291519 | -0.911308 | -0.537021 |
| 37 | 6 | 0 | -6.483832 | -2.360295 | -0.516989 |
| 38 | 1 | 0 | -5.567054 | -2.870444 | -0.233009 |
| 39 | 1 | 0 | -6.772455 | -2.710923 | -1.516197 |
| 40 | 6 | 0 | -7.537143 | -0.194152 | -0.805083 |
| 41 | 1 | 0 | -7.880896 | -0.474551 | -1.810273 |
| 42 | 1 | 0 | -7.382317 | 0.880776 | -0.814018 |
| 43 | 6 | 0 | -7.540756 | -2.748674 | 0.520045 |
| 44 | 1 | 0 | -7.747635 | -3.817818 | 0.463227 |
| 45 | 1 | 0 | -7.210818 | -2.496105 | 1.528959 |
| 46 | 6 | 0 | -8.607620 | -0.524298 | 0.231205 |
| 47 | 1 | 0 | -8.307654 | -0.208718 | 1.231914 |
| 48 | 1 | 0 | -9.553416 | -0.050194 | -0.031980 |
| 49 | 7 | 0 | -8.841979 | -2.015573 | 0.292645 |
| 50 | 6 | 0 | -9.872712 | -2.375927 | 1.327090 |
| 51 | 1 | 0 | -10.795771 | -1.849763 | 1.089155 |
| 52 | 1 | 0 | -10.031500 | -3.452581 | 1.294532 |
| 53 | 1 | 0 | -9.500614 | -2.072645 | 2.304530 |
| 54 | 6 | 0 | 7.697247 | -2.762098 | -0.088350 |
| 55 | 1 | 0 | 7.852452 | -3.769484 | 0.298935 |
| 56 | 1 | 0 | 7.557640 | -2.802628 | -1.169987 |
| 57 | 6 | 0 | 8.781360 | -0.541767 | -0.269488 |
| 58 | 1 | 0 | 8.660028 | -0.553250 | -1.353923 |
| 59 | 1 | 0 | 9.692959 | -0.001833 | -0.011250 |
| 60 | 7 | 0 | 8.967417 | -1.979942 | 0.154079 |
| 61 | 6 | 0 | 10.154819 | -2.611352 | -0.513387 |
| 62 | 1 | 0 | 11.040843 | -2.027247 | -0.269053 |
| 63 | 1 | 0 | 10.258635 | -3.630099 | -0.143029 |
| 64 | 1 | 0 | 9.985456 | -2.613639 | -1.589323 |
| 65 | 6 | 0 | -0.525089 | 5.212529 | 0.123263 |
| 66 | 1 | 0 | -1.063835 | 5.525949 | -0.778442 |
| 67 | 1 | 0 | -1.173882 | 5.428010 | 0.980114 |
| 68 | 1 | 0 | 0.386385 | 5.804555 | 0.213503 |
| 69 | 1 | 0 | 9.149830 | -1.988673 | 1.163722 |
| 70 | 1 | 0 | -9.215929 | -2.311350 | -0.615944 |

---------------------------------------------------------------------

**2,4-NMP-6-Me_water_+2H+_TR**

1003caa_bis_PIPERAZIN_pirrilium_6Me_+2H+_b3lyp631dp_PCMw_TRIPLET.log

Standard orientation:

---------------------------------------------------------------------

| Center Number | Atomic Number | Atomic Type | Coordinates (Angstroms) X | Y | Z |
|---|---|---|---|---|---|

---------------------------------------------------------------------

| | | | | | |
|---|---|---|---|---|---|
| 1 | 6 | 0 | 1.043848 | 3.207899 | 0.080965 |
| 2 | 6 | 0 | -0.189728 | 3.774841 | 0.062796 |
| 3 | 6 | 0 | -1.218397 | 1.631502 | -0.077503 |
| 4 | 6 | 0 | 0.028036 | 1.034844 | -0.075079 |
| 5 | 6 | 0 | 1.224687 | 1.783221 | 0.005920 |
| 6 | 1 | 0 | 1.895910 | 3.874568 | 0.118700 |
| 7 | 1 | 0 | 0.071038 | -0.045249 | -0.107525 |
| 8 | 6 | 0 | 2.533646 | 1.150041 | 0.030204 |
| 9 | 6 | 0 | 2.737716 | -0.171790 | -0.439045 |
| 10 | 6 | 0 | 3.675357 | 1.823522 | 0.520748 |
| 11 | 6 | 0 | 3.982854 | -0.771975 | -0.418828 |
| 12 | 1 | 0 | 1.914071 | -0.728181 | -0.873482 |
| 13 | 6 | 0 | 4.925962 | 1.223822 | 0.556243 |
| 14 | 1 | 0 | 3.580870 | 2.824209 | 0.928268 |
| 15 | 6 | 0 | 5.116423 | -0.092598 | 0.084822 |
| 16 | 1 | 0 | 4.083731 | -1.766545 | -0.838023 |
| 17 | 1 | 0 | 5.748485 | 1.781308 | 0.986960 |
| 18 | 8 | 0 | -1.330899 | 3.013734 | -0.014155 |
| 19 | 7 | 0 | 6.377991 | -0.706240 | 0.064631 |
| 20 | 6 | 0 | 6.494229 | -2.112012 | 0.455826 |
| 21 | 1 | 0 | 5.619346 | -2.675393 | 0.136953 |
| 22 | 1 | 0 | 6.555382 | -2.205016 | 1.552521 |
| 23 | 6 | 0 | 7.559166 | 0.061841 | 0.439667 |
| 24 | 1 | 0 | 7.676628 | 0.132017 | 1.533366 |
| 25 | 1 | 0 | 7.480728 | 1.077710 | 0.050473 |
| 26 | 6 | 0 | -2.488360 | 0.962027 | -0.146499 |
| 27 | 6 | 0 | -3.700745 | 1.714351 | -0.167324 |
| 28 | 6 | 0 | -2.601025 | -0.460395 | -0.200749 |
| 29 | 6 | 0 | -4.925538 | 1.100329 | -0.253631 |
| 30 | 1 | 0 | -3.653048 | 2.793078 | -0.105167 |
| 31 | 6 | 0 | -3.820405 | -1.081078 | -0.274924 |
| 32 | 1 | 0 | -1.714067 | -1.081144 | -0.198415 |
| 33 | 6 | 0 | -5.034934 | -0.323451 | -0.315843 |
| 34 | 1 | 0 | -5.810710 | 1.721802 | -0.231810 |
| 35 | 1 | 0 | -3.840198 | -2.159530 | -0.352256 |
| 36 | 7 | 0 | -6.252941 | -0.940516 | -0.414117 |
| 37 | 6 | 0 | -6.444546 | -2.385491 | -0.251747 |
| 38 | 1 | 0 | -5.577622 | -2.837943 | 0.222355 |
| 39 | 1 | 0 | -6.575958 | -2.849801 | -1.236515 |
| 40 | 6 | 0 | -7.459800 | -0.270773 | -0.911196 |
| 41 | 1 | 0 | -7.652742 | -0.639584 | -1.927003 |
| 42 | 1 | 0 | -7.312738 | 0.802047 | -0.987010 |
| 43 | 6 | 0 | -7.640088 | -2.679272 | 0.655122 |
| 44 | 1 | 0 | -7.823576 | -3.753498 | 0.688807 |
| 45 | 1 | 0 | -7.468227 | -2.308652 | 1.666617 |
| 46 | 6 | 0 | -8.662641 | -0.527163 | -0.009816 |
| 47 | 1 | 0 | -8.510365 | -0.115421 | 0.989222 |
| 48 | 1 | 0 | -9.560834 | -0.092433 | -0.448675 |
| 49 | 7 | 0 | -8.897686 | -2.009178 | 0.157211 |
| 50 | 6 | 0 | -10.076064 | -2.296438 | 1.048038 |
| 51 | 1 | 0 | -10.953775 | -1.813306 | 0.621590 |
| 52 | 1 | 0 | -10.219823 | -3.374901 | 1.088922 |
| 53 | 1 | 0 | -9.866043 | -1.901386 | 2.040873 |
| 54 | 6 | 0 | 7.711986 | -2.759753 | -0.188708 |
| 55 | 1 | 0 | 7.838639 | -3.786541 | 0.156087 |
| 56 | 1 | 0 | 7.642519 | -2.743892 | -1.277807 |
| 57 | 6 | 0 | 8.812707 | -0.539910 | -0.188216 |
| 58 | 1 | 0 | 8.763564 | -0.498831 | -1.277658 |
| 59 | 1 | 0 | 9.706755 | -0.019199 | 0.156029 |
| 60 | 7 | 0 | 8.966311 | -1.998882 | 0.175125 |
| 61 | 6 | 0 | 10.194777 | -2.602873 | -0.440520 |
| 62 | 1 | 0 | 11.063613 | -2.038653 | -0.104558 |
| 63 | 1 | 0 | 10.269953 | -3.640408 | -0.118447 |
| 64 | 1 | 0 | 10.101219 | -2.547632 | -1.524237 |
| 65 | 6 | 0 | -0.504440 | 5.229978 | 0.106555 |
| 66 | 1 | 0 | -1.061520 | 5.529064 | -0.788584 |
| 67 | 1 | 0 | -1.134442 | 5.459890 | 0.973274 |
| 68 | 1 | 0 | 0.410614 | 5.820173 | 0.167777 |
| 69 | 1 | 0 | 9.078561 | -2.058085 | 1.193057 |
| 70 | 1 | 0 | -9.122569 | -2.400285 | -0.765081 |

---------------------------------------------------------------------

# 2,6-Ph-4-DMA (EtOH)

## 2,6-Ph-4-DMA_ETOH_S0

2002aaa_24Ph_4DMAPh_pyrrilium_B3LYP631dp_PCMEtOH.log

Standard orientation:

| Center Number | Atomic Number | Atomic Type | Coordinates (Angstroms) X | Y | Z |
|---|---|---|---|---|---|
| 1 | 6 | 0 | 1.554293 | 1.183907 | 0.024603 |
| 2 | 6 | 0 | 0.184026 | 1.203888 | 0.036617 |
| 3 | 6 | 0 | -0.571892 | 0.000008 | 0.000001 |
| 4 | 6 | 0 | 0.184017 | -1.203879 | -0.036612 |
| 5 | 6 | 0 | 1.554284 | -1.183909 | -0.024595 |
| 6 | 1 | 0 | -0.306783 | 2.161958 | 0.120885 |
| 7 | 1 | 0 | -0.306802 | -2.161944 | -0.120881 |
| 8 | 6 | 0 | -2.014474 | 0.000011 | -0.000000 |
| 9 | 6 | 0 | -2.762361 | -1.178986 | 0.256721 |
| 10 | 6 | 0 | -2.762360 | 1.179006 | -0.256726 |
| 11 | 6 | 0 | -4.138287 | -1.187613 | 0.264818 |
| 12 | 1 | 0 | -2.253632 | -2.106853 | 0.491698 |
| 13 | 6 | 0 | -4.138286 | 1.187634 | -0.264826 |
| 14 | 1 | 0 | -2.253632 | 2.106873 | -0.491708 |
| 15 | 6 | 0 | -4.882710 | 0.000011 | -0.000002 |
| 16 | 1 | 0 | -4.651244 | -2.113519 | 0.487998 |
| 17 | 1 | 0 | -4.651240 | 2.113539 | -0.488014 |
| 18 | 6 | 0 | 2.448481 | -2.343567 | -0.068697 |
| 19 | 6 | 0 | 1.984594 | -3.616157 | 0.313036 |
| 20 | 6 | 0 | 3.781735 | -2.196641 | -0.495036 |
| 21 | 6 | 0 | 2.834408 | -4.716449 | 0.258423 |
| 22 | 1 | 0 | 0.971304 | -3.743624 | 0.677416 |
| 23 | 6 | 0 | 4.624856 | -3.302890 | -0.547857 |
| 24 | 1 | 0 | 4.146612 | -1.222257 | -0.797944 |
| 25 | 6 | 0 | 4.154852 | -4.564151 | -0.173464 |
| 26 | 1 | 0 | 2.468458 | -5.691880 | 0.561672 |
| 27 | 1 | 0 | 5.648866 | -3.180839 | -0.885551 |
| 28 | 1 | 0 | 4.815180 | -5.424595 | -0.214671 |
| 29 | 6 | 0 | 2.448498 | 2.343559 | 0.068703 |
| 30 | 6 | 0 | 1.984623 | 3.616149 | -0.313045 |
| 31 | 6 | 0 | 3.781746 | 2.196629 | 0.495057 |
| 32 | 6 | 0 | 2.834443 | 4.716436 | -0.258433 |
| 33 | 1 | 0 | 0.971337 | 3.743617 | -0.677438 |
| 34 | 6 | 0 | 4.624874 | 3.302873 | 0.547878 |
| 35 | 1 | 0 | 4.146615 | 1.222244 | 0.797977 |
| 36 | 6 | 0 | 4.154881 | 4.564134 | 0.173469 |
| 37 | 1 | 0 | 2.468502 | 5.691866 | -0.561694 |
| 38 | 1 | 0 | 5.648879 | 3.180819 | 0.885585 |
| 39 | 1 | 0 | 4.815214 | 5.424574 | 0.214677 |
| 40 | 7 | 0 | -6.239995 | 0.000007 | -0.000001 |
| 41 | 6 | 0 | -6.981979 | 1.235976 | -0.246161 |
| 42 | 1 | 0 | -6.778573 | 1.631972 | -1.247424 |
| 43 | 1 | 0 | -8.048005 | 1.030430 | -0.170503 |
| 44 | 1 | 0 | -6.728492 | 2.004846 | 0.491477 |
| 45 | 6 | 0 | -6.981964 | -1.235976 | 0.246139 |
| 46 | 1 | 0 | -6.778531 | -1.631997 | 1.247386 |
| 47 | 1 | 0 | -8.047993 | -1.030435 | 0.170513 |
| 48 | 1 | 0 | -6.728486 | -2.004824 | -0.491526 |
| 49 | 8 | 0 | 2.213824 | -0.000003 | 0.000003 |

## 2,6-Ph-4-DMA_ETOH_S1

2002aca_24Ph_4DMAPh_pyrrilium_TDB3LYP631dp_PCMEtOH_opt.log

Standard orientation:

| Center Number | Atomic Number | Atomic Type | Coordinates (Angstroms) X | Y | Z |
|---|---|---|---|---|---|
| 1 | 6 | 0 | 1.553748 | -1.204162 | -0.007833 |
| 2 | 6 | 0 | 0.182305 | -1.208201 | -0.018143 |

| | | | | | |
|---|---|---|---|---|---|
| 3 | 6 | 0 | -0.548356 | 0.000035 | -0.000039 |
| 4 | 6 | 0 | 0.182347 | 1.208246 | 0.018083 |
| 5 | 6 | 0 | 1.553790 | 1.204159 | 0.007810 |
| 6 | 1 | 0 | -0.338419 | -2.156568 | -0.052634 |
| 7 | 1 | 0 | -0.338346 | 2.156631 | 0.052561 |
| 8 | 6 | 0 | -2.035708 | 0.000060 | -0.000057 |
| 9 | 6 | 0 | -2.758331 | 0.020865 | -1.213650 |
| 10 | 6 | 0 | -2.758361 | -0.020762 | 1.213518 |
| 11 | 6 | 0 | -4.135350 | 0.027491 | -1.228465 |
| 12 | 1 | 0 | -2.216441 | 0.025671 | -2.152528 |
| 13 | 6 | 0 | -4.135380 | -0.027376 | 1.228300 |
| 14 | 1 | 0 | -2.216496 | -0.025623 | 2.152409 |
| 15 | 6 | 0 | -4.866896 | 0.000094 | -0.000083 |
| 16 | 1 | 0 | -4.654910 | 0.017988 | -2.176438 |
| 17 | 1 | 0 | -4.654995 | -0.017970 | 2.176254 |
| 18 | 6 | 0 | 2.440352 | 2.365185 | 0.031223 |
| 19 | 6 | 0 | 1.952967 | 3.662699 | -0.231666 |
| 20 | 6 | 0 | 3.811611 | 2.212516 | 0.320351 |
| 21 | 6 | 0 | 2.803634 | 4.762361 | -0.191872 |
| 22 | 1 | 0 | 0.909542 | 3.813141 | -0.487192 |
| 23 | 6 | 0 | 4.657719 | 3.319072 | 0.355074 |
| 24 | 1 | 0 | 4.205354 | 1.225632 | 0.531415 |
| 25 | 6 | 0 | 4.161466 | 4.599224 | 0.101779 |
| 26 | 1 | 0 | 2.406877 | 5.751264 | -0.400721 |
| 27 | 1 | 0 | 5.709842 | 3.178772 | 0.584440 |
| 28 | 1 | 0 | 4.822948 | 5.459426 | 0.127923 |
| 29 | 6 | 0 | 2.440270 | -2.365219 | -0.031223 |
| 30 | 6 | 0 | 1.952830 | -3.662716 | 0.231653 |
| 31 | 6 | 0 | 3.811542 | -2.212601 | -0.320313 |
| 32 | 6 | 0 | 2.803458 | -4.762409 | 0.191881 |
| 33 | 1 | 0 | 0.909392 | -3.813120 | 0.487150 |
| 34 | 6 | 0 | 4.657610 | -3.319187 | -0.355014 |
| 35 | 1 | 0 | 4.205326 | -1.225731 | -0.531365 |
| 36 | 6 | 0 | 4.161304 | -4.599321 | -0.101734 |
| 37 | 1 | 0 | 2.406659 | -5.751298 | 0.400718 |
| 38 | 1 | 0 | 5.709745 | -3.178925 | -0.584352 |
| 39 | 1 | 0 | 4.822755 | -5.459547 | -0.127861 |
| 40 | 7 | 0 | -6.223252 | 0.000031 | 0.000019 |
| 41 | 6 | 0 | -7.006036 | -0.239752 | 1.216594 |
| 42 | 1 | 0 | -7.169790 | 0.703858 | 1.746440 |
| 43 | 1 | 0 | -7.970453 | -0.652370 | 0.924786 |
| 44 | 1 | 0 | -6.502750 | -0.950018 | 1.868291 |
| 45 | 6 | 0 | -7.006440 | 0.239739 | -1.216325 |
| 46 | 1 | 0 | -7.170103 | -0.703853 | -1.746232 |
| 47 | 1 | 0 | -7.970887 | 0.652050 | -0.924181 |
| 48 | 1 | 0 | -6.503590 | 0.950234 | -1.868094 |
| 49 | 8 | 0 | 2.238117 | -0.000013 | -0.000003 |

---------------------------------------------------------------------

## 2,6-Ph-4-DMA_ETOH_TR

2002cca_24Ph_4DMAPh_pyrrilium_B3LYP631dp_PCMEtOH_TRIPLET.log

Standard orientation:

| Center Number | Atomic Number | Atomic Type | Coordinates (Angstroms) X | Y | Z |
|---|---|---|---|---|---|
| 1 | 6 | 0 | 1.566475 | 1.198422 | 0.003326 |
| 2 | 6 | 0 | 0.196836 | 1.206719 | 0.015998 |
| 3 | 6 | 0 | -0.560339 | 0.002597 | -0.017988 |
| 4 | 6 | 0 | 0.195728 | -1.202233 | -0.040086 |
| 5 | 6 | 0 | 1.565496 | -1.195333 | -0.030376 |
| 6 | 1 | 0 | -0.289372 | 2.169389 | 0.074974 |
| 7 | 1 | 0 | -0.292076 | -2.164721 | -0.089282 |
| 8 | 6 | 0 | -2.023889 | 0.001968 | -0.001271 |
| 9 | 6 | 0 | -2.773311 | -1.210054 | -0.006245 |
| 10 | 6 | 0 | -2.775605 | 1.212698 | 0.002263 |
| 11 | 6 | 0 | -4.148705 | -1.220540 | -0.002415 |
| 12 | 1 | 0 | -2.266540 | -2.165868 | -0.003275 |
| 13 | 6 | 0 | -4.150974 | 1.220517 | 0.011341 |
| 14 | 1 | 0 | -2.271012 | 2.169474 | -0.009938 |
| 15 | 6 | 0 | -4.888716 | -0.000769 | 0.009085 |
| 16 | 1 | 0 | -4.661386 | -2.173221 | 0.003568 |
| 17 | 1 | 0 | -4.665487 | 2.172147 | 0.005649 |
| 18 | 6 | 0 | 2.450084 | -2.359526 | -0.060160 |

| | | | | | |
|---|---|---|---|---|---|
| 19 | 6 | 0 | 1.965349 | -3.651966 | 0.229863 |
| 20 | 6 | 0 | 3.814337 | -2.210535 | -0.382069 |
| 21 | 6 | 0 | 2.813332 | -4.753312 | 0.184270 |
| 22 | 1 | 0 | 0.928527 | -3.796898 | 0.513566 |
| 23 | 6 | 0 | 4.657707 | -3.318441 | -0.422754 |
| 24 | 1 | 0 | 4.204321 | -1.226758 | -0.613784 |
| 25 | 6 | 0 | 4.163984 | -4.594330 | -0.142555 |
| 26 | 1 | 0 | 2.421106 | -5.739008 | 0.414930 |
| 27 | 1 | 0 | 5.704412 | -3.183642 | -0.677981 |
| 28 | 1 | 0 | 4.823610 | -5.455857 | -0.173517 |
| 29 | 6 | 0 | 2.451887 | 2.361698 | 0.041660 |
| 30 | 6 | 0 | 1.969959 | 3.654943 | -0.249155 |
| 31 | 6 | 0 | 3.813612 | 2.210978 | 0.373188 |
| 32 | 6 | 0 | 2.818150 | 4.755768 | -0.194455 |
| 33 | 1 | 0 | 0.935458 | 3.800533 | -0.540951 |
| 34 | 6 | 0 | 4.657254 | 3.318294 | 0.422772 |
| 35 | 1 | 0 | 4.201132 | 1.226336 | 0.605516 |
| 36 | 6 | 0 | 4.166203 | 4.595155 | 0.142092 |
| 37 | 1 | 0 | 2.428313 | 5.742229 | -0.425897 |
| 38 | 1 | 0 | 5.701967 | 3.182375 | 0.685457 |
| 39 | 1 | 0 | 4.826064 | 5.456223 | 0.180046 |
| 40 | 7 | 0 | -6.254411 | -0.002658 | 0.016289 |
| 41 | 6 | 0 | -7.009650 | 1.251043 | 0.105173 |
| 42 | 1 | 0 | -6.871710 | 1.842099 | -0.805590 |
| 43 | 1 | 0 | -8.065386 | 1.020671 | 0.220289 |
| 44 | 1 | 0 | -6.676352 | 1.833999 | 0.966489 |
| 45 | 6 | 0 | -7.005904 | -1.259048 | -0.064850 |
| 46 | 1 | 0 | -6.854241 | -1.850605 | 0.843375 |
| 47 | 1 | 0 | -8.063889 | -1.032751 | -0.166668 |
| 48 | 1 | 0 | -6.680730 | -1.839581 | -0.930944 |
| 49 | 8 | 0 | 2.250062 | 0.001392 | -0.019080 |

---------------------------------------------------------------------

# 2,6-DMA-4-NMP

## 2,6-DMA-4-NMP_water_S0

1211aaa_2_6_NMe2_4_NMP_pirrilium_b3lyp631dp_PCMw.log

Standard orientation:

| Center Number | Atomic Number | Atomic Type | Coordinates (Angstroms) X | Y | Z |
|---|---|---|---|---|---|
| 1 | 6 | 0 | 0.261950 | 1.210432 | 0.054712 |
| 2 | 6 | 0 | 1.641045 | 1.192970 | 0.019700 |
| 3 | 6 | 0 | 1.629956 | -1.194473 | 0.011157 |
| 4 | 6 | 0 | 0.250386 | -1.199148 | 0.020390 |
| 5 | 6 | 0 | -0.481946 | 0.009034 | 0.049478 |
| 6 | 1 | 0 | -0.238047 | 2.164198 | 0.135955 |
| 7 | 1 | 0 | -0.261165 | -2.147968 | -0.045291 |
| 8 | 6 | 0 | -1.939641 | 0.015436 | 0.072221 |
| 9 | 6 | 0 | -2.680865 | -1.103213 | 0.508673 |
| 10 | 6 | 0 | -2.684838 | 1.144603 | -0.337299 |
| 11 | 6 | 0 | -4.063694 | -1.100776 | 0.541305 |
| 12 | 1 | 0 | -2.167521 | -1.982303 | 0.883672 |
| 13 | 6 | 0 | -4.065396 | 1.156283 | -0.322544 |
| 14 | 1 | 0 | -2.174898 | 2.021968 | -0.720575 |
| 15 | 6 | 0 | -4.811759 | 0.028166 | 0.113776 |
| 16 | 1 | 0 | -4.565077 | -1.973855 | 0.936720 |
| 17 | 1 | 0 | -4.570976 | 2.039949 | -0.689574 |
| 18 | 6 | 0 | 2.529537 | 2.334469 | 0.022444 |
| 19 | 6 | 0 | 3.914963 | 2.187113 | 0.257947 |
| 20 | 6 | 0 | 2.047447 | 3.642295 | -0.211114 |
| 21 | 6 | 0 | 4.768024 | 3.272506 | 0.268673 |
| 22 | 1 | 0 | 4.322319 | 1.202179 | 0.453979 |
| 23 | 6 | 0 | 2.888483 | 4.736206 | -0.206153 |
| 24 | 1 | 0 | 0.997054 | 3.808009 | -0.424732 |
| 25 | 6 | 0 | 4.283897 | 4.590588 | 0.036674 |
| 26 | 1 | 0 | 5.818691 | 3.104272 | 0.465301 |
| 27 | 1 | 0 | 2.468188 | 5.713721 | -0.402198 |
| 28 | 8 | 0 | 2.288101 | -0.003705 | 0.004888 |
| 29 | 7 | 0 | -6.191856 | 0.023114 | 0.089239 |
| 30 | 7 | 0 | 5.120266 | 5.668231 | 0.045064 |
| 31 | 6 | 0 | 4.595368 | 7.012060 | -0.177593 |
| 32 | 1 | 0 | 5.412232 | 7.728516 | -0.108268 |

| | | | | | |
|---|---|---|---|---|---|
| 33 | 1 | 0 | 4.139825 | 7.108440 | -1.170399 |
| 34 | 1 | 0 | 3.843453 | 7.278807 | 0.574144 |
| 35 | 6 | 0 | 6.550597 | 5.490091 | 0.275877 |
| 36 | 1 | 0 | 7.042541 | 6.459828 | 0.222508 |
| 37 | 1 | 0 | 6.749320 | 5.060160 | 1.264855 |
| 38 | 1 | 0 | 7.001074 | 4.837239 | -0.480796 |
| 39 | 6 | 0 | -6.942602 | -1.171898 | 0.485210 |
| 40 | 1 | 0 | -7.056460 | -1.213006 | 1.579551 |
| 41 | 1 | 0 | -6.401928 | -2.062671 | 0.164499 |
| 42 | 6 | 0 | -6.958115 | 1.263492 | 0.276939 |
| 43 | 1 | 0 | -6.418053 | 2.109920 | -0.141843 |
| 44 | 1 | 0 | -7.090747 | 1.448813 | 1.353946 |
| 45 | 6 | 0 | 2.507586 | -2.343942 | -0.020498 |
| 46 | 6 | 0 | 3.886195 | -2.208487 | -0.299197 |
| 47 | 6 | 0 | 2.021538 | -3.647830 | 0.226580 |
| 48 | 6 | 0 | 4.728880 | -3.301310 | -0.338038 |
| 49 | 1 | 0 | 4.295928 | -1.226886 | -0.506632 |
| 50 | 6 | 0 | 2.852312 | -4.749109 | 0.193703 |
| 51 | 1 | 0 | 0.977050 | -3.804743 | 0.473254 |
| 52 | 6 | 0 | 4.240611 | -4.615442 | -0.092810 |
| 53 | 1 | 0 | 5.774324 | -3.141982 | -0.567207 |
| 54 | 1 | 0 | 2.429816 | -5.723155 | 0.401933 |
| 55 | 7 | 0 | 5.066761 | -5.700383 | -0.129106 |
| 56 | 6 | 0 | 4.537143 | -7.040021 | 0.107183 |
| 57 | 1 | 0 | 3.757721 | -7.297006 | -0.619476 |
| 58 | 1 | 0 | 5.344282 | -7.763928 | 0.007723 |
| 59 | 1 | 0 | 4.114608 | -7.135695 | 1.114589 |
| 60 | 6 | 0 | 6.491113 | -5.533980 | -0.401863 |
| 61 | 1 | 0 | 6.969502 | -4.886890 | 0.342542 |
| 62 | 1 | 0 | 6.976076 | -6.507993 | -0.365247 |
| 63 | 1 | 0 | 6.664142 | -5.103329 | -1.395343 |
| 64 | 6 | 0 | -8.328137 | 1.176012 | -0.389235 |
| 65 | 1 | 0 | -8.886223 | 2.089735 | -0.158920 |
| 66 | 1 | 0 | -8.198699 | 1.131834 | -1.488305 |
| 67 | 6 | 0 | -8.320060 | -1.196132 | -0.177568 |
| 68 | 1 | 0 | -8.192035 | -1.343939 | -1.267811 |
| 69 | 1 | 0 | -8.873263 | -2.059077 | 0.207883 |
| 70 | 6 | 0 | -10.420917 | -0.032486 | -0.433821 |
| 71 | 1 | 0 | -10.957287 | -0.885606 | -0.007056 |
| 72 | 1 | 0 | -10.963133 | 0.880559 | -0.169256 |
| 73 | 1 | 0 | -10.438103 | -0.133750 | -1.534811 |
| 74 | 7 | 0 | -9.069990 | 0.022497 | 0.113679 |

---------------------------------------------------------------------

**2,6-DMA-4-NMP_+H+_water_S0**

1215aaa_2_6_NMe2_4_NMPH_pirrilium_b3lyp631dp_PCMw.log

Standard orientation:

| Center Number | Atomic Number | Atomic Type | Coordinates (Angstroms) X | Y | Z |
|---|---|---|---|---|---|
| 1 | 6 | 0 | 0.290984 | -1.211448 | -0.071151 |
| 2 | 6 | 0 | 1.672553 | -1.195130 | -0.030956 |
| 3 | 6 | 0 | 1.662428 | 1.195085 | -0.029527 |
| 4 | 6 | 0 | 0.280418 | 1.199299 | -0.050401 |
| 5 | 6 | 0 | -0.444613 | -0.009134 | -0.076110 |
| 6 | 1 | 0 | -0.214390 | -2.162914 | -0.148391 |
| 7 | 1 | 0 | -0.236399 | 2.145982 | 0.006201 |
| 8 | 6 | 0 | -1.909751 | -0.014307 | -0.102606 |
| 9 | 6 | 0 | -2.639551 | 1.068405 | -0.628219 |
| 10 | 6 | 0 | -2.650860 | -1.103858 | 0.401403 |
| 11 | 6 | 0 | -4.026358 | 1.067519 | -0.658717 |
| 12 | 1 | 0 | -2.119517 | 1.911004 | -1.071372 |
| 13 | 6 | 0 | -4.034301 | -1.105917 | 0.395412 |
| 14 | 1 | 0 | -2.139389 | -1.945890 | 0.855278 |
| 15 | 6 | 0 | -4.763840 | -0.016988 | -0.135639 |
| 16 | 1 | 0 | -4.528910 | 1.906909 | -1.121843 |
| 17 | 1 | 0 | -4.550963 | -1.944563 | 0.846380 |
| 18 | 6 | 0 | 2.559607 | -2.334811 | -0.026967 |
| 19 | 6 | 0 | 3.948874 | -2.186745 | -0.243284 |
| 20 | 6 | 0 | 2.073577 | -3.644205 | 0.193892 |
| 21 | 6 | 0 | 4.801184 | -3.271764 | -0.247123 |
| 22 | 1 | 0 | 4.359167 | -1.201305 | -0.430011 |
| 23 | 6 | 0 | 2.914063 | -4.737684 | 0.195991 |

| | | | | | |
|---|---|---|---|---|---|
| 24 | 1 | 0 | 1.020306 | -3.811017 | 0.391427 |
| 25 | 6 | 0 | 4.313231 | -4.591152 | -0.027009 |
| 26 | 1 | 0 | 5.854390 | -3.103301 | -0.429074 |
| 27 | 1 | 0 | 2.491049 | -5.716027 | 0.381434 |
| 28 | 8 | 0 | 2.315940 | 0.002689 | -0.016101 |
| 29 | 7 | 0 | -6.160348 | -0.010747 | -0.093554 |
| 30 | 7 | 0 | 5.148730 | -5.668020 | -0.028590 |
| 31 | 6 | 0 | 4.620825 | -7.013276 | 0.180800 |
| 32 | 1 | 0 | 5.439001 | -7.728971 | 0.121096 |
| 33 | 1 | 0 | 4.150680 | -7.112990 | 1.166255 |
| 34 | 1 | 0 | 3.880629 | -7.276808 | -0.583416 |
| 35 | 6 | 0 | 6.582431 | -5.490036 | -0.240830 |
| 36 | 1 | 0 | 7.072796 | -6.460320 | -0.184448 |
| 37 | 1 | 0 | 6.793281 | -5.057058 | -1.225804 |
| 38 | 1 | 0 | 7.023114 | -4.840133 | 0.523865 |
| 39 | 6 | 0 | -6.899927 | 1.178376 | -0.501356 |
| 40 | 1 | 0 | -7.035410 | 1.226392 | -1.593749 |
| 41 | 1 | 0 | -6.361451 | 2.075738 | -0.195124 |
| 42 | 6 | 0 | -6.904889 | -1.241180 | -0.364950 |
| 43 | 1 | 0 | -6.353954 | -2.112779 | -0.017312 |
| 44 | 1 | 0 | -7.059005 | -1.369239 | -1.448446 |
| 45 | 6 | 0 | 2.538827 | 2.342331 | 0.009692 |
| 46 | 6 | 0 | 3.918824 | 2.204792 | 0.284443 |
| 47 | 6 | 0 | 2.051070 | 3.649148 | -0.222630 |
| 48 | 6 | 0 | 4.760362 | 3.297118 | 0.333039 |
| 49 | 1 | 0 | 4.329593 | 1.221556 | 0.481355 |
| 50 | 6 | 0 | 2.880911 | 4.749900 | -0.180122 |
| 51 | 1 | 0 | 1.005856 | 3.808698 | -0.463984 |
| 52 | 6 | 0 | 4.270313 | 4.613942 | 0.102602 |
| 53 | 1 | 0 | 5.806288 | 3.136367 | 0.558726 |
| 54 | 1 | 0 | 2.457459 | 5.725925 | -0.376443 |
| 55 | 7 | 0 | 5.094915 | 5.698144 | 0.149733 |
| 56 | 6 | 0 | 4.564043 | 7.040562 | -0.070205 |
| 57 | 1 | 0 | 3.786421 | 7.288535 | 0.661316 |
| 58 | 1 | 0 | 5.371224 | 7.763415 | 0.035461 |
| 59 | 1 | 0 | 4.139409 | 7.146698 | -1.075495 |
| 60 | 6 | 0 | 6.519947 | 5.530639 | 0.420753 |
| 61 | 1 | 0 | 6.998658 | 4.892693 | -0.331062 |
| 62 | 1 | 0 | 7.003320 | 6.505698 | 0.395031 |
| 63 | 1 | 0 | 6.692856 | 5.089220 | 1.409351 |
| 64 | 6 | 0 | -8.247277 | -1.238882 | 0.353477 |
| 65 | 1 | 0 | -8.834448 | -2.119268 | 0.089393 |
| 66 | 1 | 0 | -8.119910 | -1.193647 | 1.436518 |
| 67 | 6 | 0 | -8.251568 | 1.235328 | 0.203916 |
| 68 | 1 | 0 | -8.125538 | 1.316733 | 1.284613 |
| 69 | 1 | 0 | -8.842962 | 2.077096 | -0.158023 |
| 70 | 6 | 0 | -10.381726 | 0.019164 | 0.661819 |
| 71 | 1 | 0 | -10.932446 | 0.889838 | 0.309288 |
| 72 | 1 | 0 | -10.924957 | -0.894335 | 0.424589 |
| 73 | 1 | 0 | -10.205415 | 0.089419 | 1.734485 |
| 74 | 7 | 0 | -9.053946 | -0.021613 | -0.036533 |
| 75 | 1 | 0 | -9.242286 | -0.083759 | -1.043276 |

---------------------------------------------------------------------

## 6.6. Computed energies

**Table S27A:** Computed energies (*E*), zero point energies (*ZPE*), internal energies (*U*), enthalpies (*H*) and Gibbs free energies (*G*) given in Hartree as well as entropies (*S*) given in J mol$^{-1}$ K$^{-1}$ at B3LYP/6-31G(d,p) and TD-B3LYP/6-31G(d,p) levels of theory with the consideration of PCM solvent method using the parameter set of water or in other solvents. (N of Im frq) = Number of imaginary frequencies

| Mol | form | solvent | state | E(S1) | E(S0) | ZPE | U | H | G | S | N of Im frq |
|---|---|---|---|---|---|---|---|---|---|---|---|
| 2,4-NMP-6-Me | np | water | GS | - | -1382.17672122 | -1381.587321 | -1381.557087 | -1381.556142 | -1381.652114 | 201.990 | 0 |
| | np | water | LE | -1382.08547201 | - | - | - | - | - | - | nc |
| | np | water | S1(opt) | -1382.10569713 | -1382.15661229 | -1381.518303 | -1381.488050 | -1381.487106 | -1381.582220 | 200.183 | 0 |
| | np | water | T1 | - | -1382.11167474 | -1381.525077 | -1381.494612 | -1381.493668 | -1381.590992 | 204.835 | 0 |
| | +2H+ | water | GS | - | -1383.09104481 | -1382.469315 | -1382.439309 | -1382.438365 | -1382.531718 | 196.478 | 0 |
| | +2H+ | water | LE | -1382.99747121 | - | - | - | - | - | - | nc |
| | +2H+ | water | S1(opt) | -1383.00560400 | -1383.08753451 | -1382.386934 | -1382.356538 | -1382.355593 | -1382.449987 | 198.669 | 0 |
| | +2H+ | water | T1 | - | -1383.01963856 | -1382.400722 | -1382.370346 | -1382.369402 | -1382.465527 | 202.314 | 0 |
| 2,4,6-DMA | np | vacuo | GS | - | -1363.69153013 | -1363.140203 | -1363.108104 | -1363.107160 | -1363.206872 | 209.861 | 0 |
| | np | vacuo | LE | -1363.65486436 | - | - | - | - | - | - | nc |
| | np | vacuo | S1(opt) | -1363.61795031 | -1363.67206612 | | | | | | nc |
| | np | vacuo | T1 | - | -1363.62271921 | -1363.074778 | -1363.042193 | -1363.041249 | -1363.143645 | 215.512 | 0 |
| | np | THF | GS | - | -1363.73282549 | -1363.181942 | -1363.149877 | -1363.148933 | -1363.248279 | 209.091 | 0 |
| | np | THF | LE | -1363.65188541 | - | - | - | - | - | - | nc |
| | np | THF | S1(opt) | -1363.65399544 | -1363.73074311 | - | - | - | - | - | nc |
| | np | THF | T1 | - | | | | | | | 0 |
| | np | Toluene | GS | - | -1363.71920679 | -1363.167532 | -1363.135712 | -1363.134768 | -1363.233021 | 206.790 | 0 |
| | np | Toluene | LE | -1363.63850237 | - | - | - | - | - | - | nc |
| | np | Toluene | S1(opt) | -1363.63850232 | -1363.71680124 | - | - | - | - | - | nc |
| | np | Toluene | T1 | - | | | | | | | 0 |
| | np | water | GS | - | -1363.73907004 | -1363.188451 | -1363.156293 | -1363.155349 | -1363.255071 | 209.882 | 0 |
| | np | water | LE | -1363.65919723 | - | - | - | - | - | - | nc |
| | np | water | S1(opt) | -1363.66128568 | -1363.73702263 | - | - | - | - | - | nc |
| | np | water | T1 | - | | | | | | | 0 |

np = non-protonated, nc: not calculated.

**Table S27B:** Computed energies (*E*), zero point energies (*ZPE*), internal energies (*U*), enthalpies (*H*) and Gibbs free energies (*G*) given in Hartree as well as entropies (*S*) given in J mol$^{-1}$ K$^{-1}$ at B3LYP/6-31G(d,p) and TD-B3LYP/6-31G(d,p) levels of theory with the consideration of PCM solvent method using the parameter set of water or in other solvents. (N of Im frq) = Number of imaginary frequencies

| Mol | form | solvent | state | E($S_1$) | E($S_0$) | ZPE | U | H | G | S | N of Im frq |
|---|---|---|---|---|---|---|---|---|---|---|---|
| 2,6-NMP-4-DMA | np | water | GS | | -1707.90169245 | -1707.186157 | -1707.148359 | -1707.147415 | -1707.261163 | 239.404 | 0 |
| | np | water | LE | -1707.82049511 | - | - | - | - | - | - | nc |
| | np | water | S1(opt) | -1707.82383649 | -1707.89824481 | - | - | - | - | - | nc |
| | np | water | T1 | | -1707.83549769 | -1707.123154 | -1707.084888 | -1707.083944 | -1707.200839 | 246.026 | 0 |
| | +2H+ | water | GS | | -1708.81765503 | -1708.069881 | -1708.033137 | -1708.032193 | -1708.140851 | 228.690 | 0 |
| | +2H+ | water | LE | -1708.71723543 | - | - | - | - | - | - | nc |
| | +2H+ | water | S1(opt) | -1708.74620132 | -1708.79502162 | -1708.001098 | -1707.963300 | -1707.962355 | -1708.074539 | 236.110 | 0 |
| | +2H+ | water | T1 | | -1708.75340622 | -1708.008261 | -1707.970477 | -1707.969532 | -1708.082066 | 236.847 | 0 |
| 2,4-DMA-6-Me | np | water | GS | | -1038.01452293 | -1037.589979 | -1037.565432 | -1037.564487 | -1037.646306 | 172.202 | 0 |
| | np | water | LE | -1037.92579445 | - | - | - | - | - | - | nc |
| | np | water | S1(opt) | -1037.93395932 | -1038.01203006 | | | | | | nc |
| | np | water | T1 | | -1037.89732344 | | | | | | 0 |
| | +2H+ | water | GS | | -1383.09033070 | -1382.468614 | -1382.438546 | -1382.437602 | -1382.531656 | 197.955 | 0 |
| | +2H+ | water | LE | -1382.99855333 | - | - | - | - | - | - | nc |
| | +2H+ | water | S1(opt) | -1383.00644221 | -1383.08721656 | | | | | | nc |
| | +2H+ | water | | | | | | | | | 0 |
| 2,6-NdMP-4-DMA | np | water | GS | | -1787.42783215 | -1786.624759 | -1786.584620 | -1786.583675 | -1786.700532 | 245.946 | 0 |
| | np | water | LE | -1787.33629308 | | | | | | | nc |
| | np | water | S1(opt) | -1787.35632041 | -1787.40535263 | | | | | | nc |
| | np | water | T1 | | -1787.36347262 | | | | | | 0 |

np = non-protonated, n.c.: not calculated.

**Table S27C:** Computed energies ($E$), zero point energies ($ZPE$), internal energies ($U$), enthalpies ($H$) and Gibbs free energies ($G$) given in Hartree as well as entropies ($S$) given in J mol$^{-1}$ K$^{-1}$ at B3LYP/6-31G(d,p) and TD-B3LYP/6-31G(d,p) levels of theory with the consideration of PCM solvent method using the parameter set of water or in other solvents. (N of Im frq) = Number of imaginary frequencies

| Mol | form_ring(s) | solvent | state | E(S$_1$) | E(S$_0$) | ZPE | U | H | G | S | N of Im frq |
|---|---|---|---|---|---|---|---|---|---|---|---|
| 2,4,6-NMP | np | water | GS | - | -1879.98296874 | -1879.185033 | -1879.144385 | -1879.143441 | -1879.264437 | 254.659 | 0 |
| | np | water | LE | -1879.89879763 | | | | | | | nc |
| | np | water | S1(opt) | -1879.90635432 | -1879.97954174 | | | | | | nc |
| | np | water | T1 | | -1879.91818652 | | | | | | 0 |
| | +2H+_B,D | water | GS | | -1880.89878525 | -1880.068359 | -1880.027886 | -1880.026942 | -1880.145872 | 250.310 | 0 |
| | +2H+_B,D | water | LE | -1880.80802814 | | | | | | | nc |
| | +2H+_B,D | water | S1(opt) | -1880.83327244 | -1880.86566971 | | | | | | nc |
| | +2H+_B,D | water | T1 | | -1880.83575889 | -1880.008994 | -1879.968997 | -1879.968053 | -1880.086514 | 249.324 | 0 |
| | +1H+_B | water | GS | | -1880.44120881 | -1879.626972 | -1879.586429 | -1879.585485 | -1879.705391 | 252.363 | 0 |
| | +1H+_B | water | LE | -1880.360372 | | | | | | | nc |
| | +1H+_B | water | S1(opt) | -1880.36501942 | -1880.43581898 | | | | | | nc |
| | +1H+_C | water | GS | | -1880.44110310 | -1879.627204 | -1879.586590 | -1879.585646 | -1879.706019 | 253.347 | 0 |
| | +1H+_C | water | LE | -1880.36320321 | | | | | | | nc |
| | +1H+_C | water | S1(opt) | -1880.36685708 | -1880.43782720 | | | | | | nc |
| | +2H+_B,C | water | GS | | -1880.89869970 | -1880.068489 | -1880.027992 | -1880.027048 | -1880.146306 | 251.000 | 0 |
| | +2H+_B,C | water | LE | -1880.818826 | | | | | | | nc |
| | +2H+_B,C | water | S1(opt) | -1880.82466034 | -1880.89169955 | | | | | | nc |
| | +3H+ | water | GS | | -1881.35558810 | -1880.508931 | -1880.468607 | -1880.467662 | -1880.585301 | 247.592 | 0 |
| | +3H+ | water | LE | -1881.26650198 | | | | | | | nc |
| | +3H+ | water | S1(opt) | -1881.27441672 | -1881.35227082 | | | | | | nc |
| | +3H+ | water | T1 | | -1881.28615149 | | | | | | 0 |
| 2,6-NMP-4-Morf | np | water | GS | | -1860.53302894 | -1859.775236 | -1859.736130 | -1859.735185 | -1859.853021 | 248.005 | 0 |
| | np | water | LE | -1860.44908210 | | | | | | | nc |
| | np | water | S1(opt) | -1860.45667533 | -1860.52961253 | | | | | | nc |
| | | | | | | | | | | | 0 |
| | +2H+ | water | GS | | -1861.44874306 | -1860.658540 | -1860.619585 | -1860.618640 | -1860.734638 | 244.138 | 0 |
| | +2H+ | water | LE | -1861.35748222 | | | | | | | nc |
| | +2H+ | water | S1(opt) | -1861.37762929 | -1861.42612372 | | | | | | nc |
| | | | | | | | | | | | 0 |
| | | | | | | | | | | | 0 |

np = non-protonated, n.c.: not calculated.

**Table S27D:** Computed energies (*E*), zero point energies (*ZPE*), internal energies (*U*), enthalpies (*H*) and Gibbs free energies (*G*) given in Hartree as well as entropies (*S*) given in J mol$^{-1}$ K$^{-1}$ at B3LYP/6-31G(d,p) and TD-B3LYP/6-31G(d,p) levels of theory with the consideration of PCM solvent method using the parameter set of water or in other solvents. (N of Im frq) = Number of imaginary frequencies

| Mol | form | solvent | state | E($S_1$) | E($S_0$) | ZPE | U | H | G | S | N of Im frq |
|---|---|---|---|---|---|---|---|---|---|---|---|
| 2,6-NMP-4-OMe | np | water | GS | | -1688.44827170 | -1687.773107 | -1687.737263 | -1687.736318 | -1687.845151 | 229.057 | 0 |
| | np | water | LE | -1688.36597933 | | | | | | | |
| | np | water | S1(opt) | -1688.37349495 | -1688.44500718 | | | | | | 0 |
| | np | water | T1 | | -1688.38567631 | | | | | | 0 |
| | +2H+ | water | GS | | -1689.36324367 | -1688.655732 | -1688.620029 | -1688.619085 | -1688.726312 | 225.678 | 0 |
| | +2H+ | water | LE | -1689.27435943 | | | | | | | |
| | +2H+ | water | S1(opt) | -1689.28179622 | -1689.36026481 | | | | | | 0 |
| | +2H+ | water | T1 | | -1689.29352331 | | | | | | 0 |
| 2,6-NMP-4-2MeOPh | np | water | GS | | -1688.44389131 | -1687.768730 | -1687.732877 | -1687.731933 | -1687.840907 | 229.356 | 0 |
| | np | water | LE | -1688.36247551 | | | | | | | |
| | np | water | S1(opt) | -1688.37019027 | -1688.44081596 | | | | | | 0 |
| | np | water | T1 | | -1688.38303492 | | | | | | 0 |
| | +2H+ | water | GS | | -1689.35876320 | | | | | | 0 |
| | +2H+ | water | LE | -1689.27120542 | | | | | | | |
| | +2H+ | water | S1(opt) | -1689.27866953 | -1689.35567952 | | | | | | 0 |
| | +2H+ | water | T1 | | -1689.29100162 | | | | | | 0 |
| 2,4-Ph-6-DMA | np | water | GS | | -1095.77275620 | -1095.367881 | -1095.344816 | -1095.343871 | -1095.423054 | 166.653 | 0 |
| | np | water | LE | -1095.67751927 | | | | | | | |
| | np | water | S1(opt) | -1095.70630542 | -1095.74936829 | | | | | | 0 |
| | np | water | T1 | | -1095.71170000 | | | | | | 0 |
| | np | EtOH | GS | | -1095.77114631 | -1095.366271 | -1095.343204 | -1095.342260 | -1095.421422 | 166.612 | 0 |
| | np | EtOH | LE | -1095.67612134 | | | | | | | |
| | np | EtOH | S1(opt) | -1095.70537789 | -1095.74777005 | | | | | | 0 |
| | np | EtOH | T1 | | -1095.70966727 | | | | | | 0 |
| | np | vacuo | GS | | -1095.71964009 | -1095.314375 | -1095.291413 | -1095.290469 | -1095.368968 | 165.214 | 0 |
| | np | vacuo | LE | -1095.61959534 | | | | | | | |
| | np | vacuo | S1(opt) | -1095.66835788 | -1095.69622485 | | | | | | 0 |
| | np | vacuo | T1 | | -1095.64970000 | | | | | | 0 |

np = non-protonated, n.c.: not calculated.

## 7. References


[1] M. J. Strauss, K. X. Liu, M. E. Greaves, J. C. Dahl, S.-T. Kim, Y.-J. Wu, M. A. Schmidt, P. M. Scola, S. L. Buchwald, *J. Am. Chem. Soc.* **2024**, *146*, 18616–18625.

[2] B. Liu, X. Li, Z. Liu, B. He, H. Xu, J. Cao, F. Zeng, H. Feng, Y. Ren, H. Li, T. Wang, J. Li, Y. Ye, L. Zhao, C. Ran, Y. Li, *J. Med. Chem.* **2024**, *67*, 9104–9123.

[3] A. Nanni, D. Kong, C. Zhu, M. Rueping, *Green Chem.* **2024**, *26*, 8341–8347.

[4] K. Polidano, B. G. Reed-Berendt, A. Basset, A. J. A. Watson, J. M. J. Williams, L. C. Morrill, *Org. Lett.* **2017**, *19*, 6716–6719.

[5] S. Zhu, W. Wu, D. Hong, F. Chai, Z. Huang, X. Zhu, S. Zhou, S. Wang, *Inorg. Chem.* **2024**, *63*, 14860–14875.

[6] T. P. Vasilyeva, D. V Vorobyeva, S. N. Osipov, *Russ. Chem. Bull.* **2016**, *65*, 2211–2215.

[7] D. Samanta, S. B. Ebrahimi, C. D. Kusmierz, H. F. Cheng, C. A. Mirkin, *J. Am. Chem. Soc.* **2020**, *142*, 13350–13355.

[8] A. Dragan, A. E. Graham, C. D. Geddes, *J. Fluoresc.* **2014**, *24*, 397–402.

[9] F. Domahidy, B. Kovács, L. Cseri, G. Katona, B. Rózsa, Z. Mucsi, E. Kovács, *ChemPhotoChem* **2024**, *8*, e202400080.

[10] J. D. McGhee, P. H. von Hippel, *J. Mol. Biol.* **1974**, *86*, 469–489.
